# Design, synthesis, and antitumor activity of novel thioheterocyclic nucleoside derivatives by suppressing the c-MYC pathway

**DOI:** 10.1016/j.apsb.2025.05.008

**Published:** 2025-05-19

**Authors:** Xian-Jia Li, Ke-Xin Huang, Ke-Xin Wang, Ru Liu, Dong-Chao Wang, Yu-Ru Liang, Er-Jun Hao, Yang Wang, Hai-Ming Guo

**Affiliations:** a State Key Laboratory of Antiviral Drugs, Pingyuan Laboratory, Key Laboratory of Green Chemical Media and Reactions, Ministry of Education, School of Chemistry and Chemical Engineering, Henan Normal University, Xinxiang 453007, China; b School of Pharmacy, Fudan University, Shanghai 201203, China; c Institute of Translation Medicine, Shanghai Jiao Tong University, Shanghai 200240, China; d School of Biological and Chemical Engineering, Nanyang Institute of Technology, Nanyang 473004, China

**Keywords:** Thioheterocyclic nucleoside, Structure–activity relationships, Anti-proliferation activity, Antitumor activity, ROS, Autophagy, Target prediction, The c-MYC pathway

## Abstract

Eightly-four novel thioheterocyclic nucleoside derivatives were designed, synthesized, and evaluated for antitumor activity *in vitro* and *in vivo*. Most of the compounds inhibited the growth of HCT116 and HeLa cancer cells *in vitro*, among them **33a** and **36b** exhibited potent activity against HCT116 cells (IC_50_ = 0.27 and 0.49 μmol/L, respectively). Both compounds **33a** and **36b** inhibited cell metastasis, arrested the cell cycle in the G_2_/M phase, and induced apoptosis *in vitro*. Mechanistic studies revealed that **33a** and **36b** increased ROS levels, led to DNA damage, ER stress, and mitochondrial dysfunction, and inhibited autophagy in HCT116 cells. Biological information analysis, RNA-sequencing, Gene Set Enrichment Analysis (GSEA), drug affinity responsive target stability (DARTS) assay, cellular thermal shift assay (CETSA), and SPR experiments identified that compounds **33a** and **36b** showed antitumor activity by suppressing the c-MYC pathway. *c-MYC* silencing assays indicated that c-MYC proteins participated in **33a**-mediated anticancer activities in HCT116 cells. More importantly, compound **33a** presented favorable pharmacokinetic properties in mice (*T*_1/2_ = 6.8 h) and showed significant antitumor efficacy *in vivo* without obvious toxicity, showing promising potential for further clinical development.

## Graphical abstract

The thioheterocyclic nucleoside derivative **33a** exhibited antitumor activity by inhibiting the c-MYC pathway without apparent toxicity.

**Figure:**
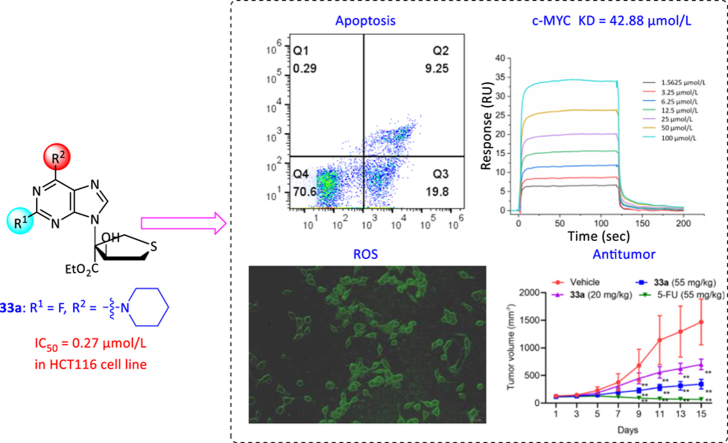

## 1 Introduction

**Figure 1:**
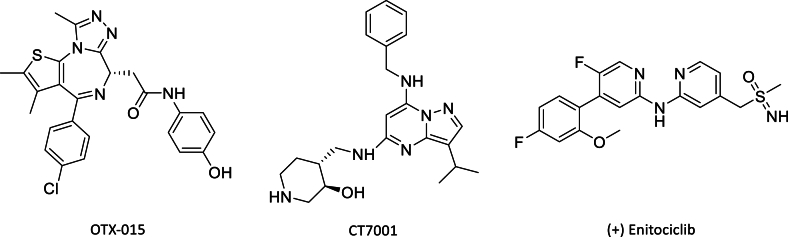
c-MYC inhibitors in clinical trials.

Cancer cells undergo uncontrolled and abnormal growth. Normal cells have regulatory mechanisms that ensure they grow, divide, and die in a controlled manner. Cancer cells often evade these normal regulatory signals, however, leading to uncontrolled proliferation^1^. Abnormal activation of *c-MYC* is one of the most common features of human cancers. As a transcription factor, c-MYC promotes cancer cell proliferation and inhibits apoptosis^2^^,^^3^. Dysregulation or overexpression of c-MYC can lead to uncontrolled cell growth and contribute to the development and progression of tumors^4^. At present, most compounds under evaluation in clinical trials target c-MYC transcription through bromodomain-containing protein 4 (BRD4)^5^, cyclin-dependent kinase 7 (CDK7)^6^, and cyclin-dependent kinase 9 (CDK9) inhibition^7^, but few bind to c-MYC directly (Fig. 1)^8^.

**Figure 2:**
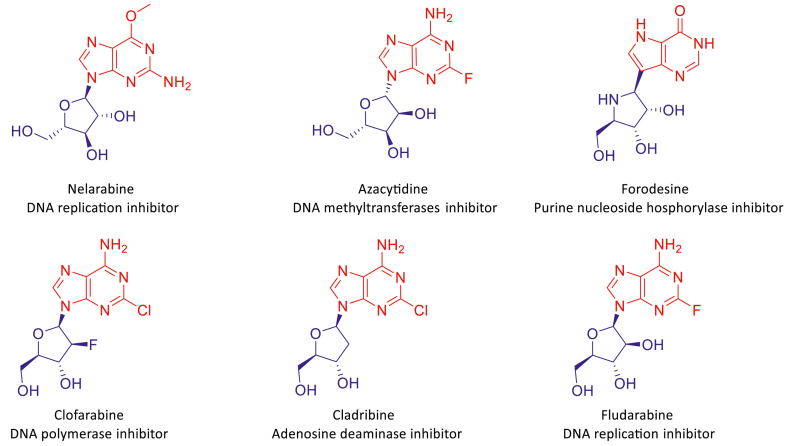
Purine nucleosides as antitumor agents for clinical use.

Nucleoside heterocyclic analogs, especially purine nucleoside analogs such as nelarabine^9^, azacytidine^10^, forodesine^11^, clofarabine^12^, fludarabine^13^, and cladribine^14^, have been widely used in clinical applications (Fig. 2). Substitution at the 2- and 6-positions of these purine nucleosides with moieties such as aryl, halogen, and alkyl, is crucial for their effective antitumor activity^15^.

Compared with oxygen, a sulfur atom in a compound's structure can lead to significant differences in chemical reactivity, protein molecular recognition, and metabolic stability^16^. Therefore, structural modifications of nucleosides to sulfur-containing heterocycles may lead to efficient antitumor drugs^17^. Asymmetric synthetic approaches to chiral sulfur-containing heterocyclic nucleosides have been reported by our group^18^^,^^19^. Based on these synthetic methods, 84 novel compounds were designed and synthesized. Their antitumor activities were evaluated *in vitro* and *in vivo*, finding compound **33a** to effectively bind to c-MYC suggesting its potential as an antitumor candidate.

## 2 Results and discussion

### 2.1 Chemistry

**Scheme 1:**
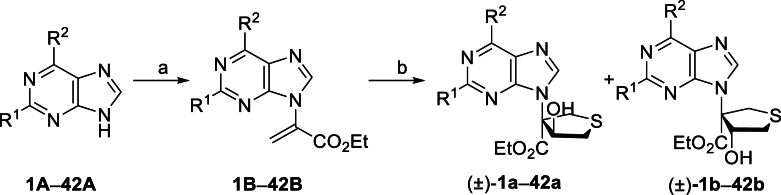
Synthesis of **1a**/**1b**‒**42a**/**42b**. Reagents and conditions: (a) PPh_3_, NaOAc, HOAc, Ethyl propiolate, toluene, 110 °C, 12 h. (b) 1,4-Dithiane-2,5-diol, Et_3_N (20 mol%), CH_2_Cl_2_, rt, 4 h.

The Ni(II)/trisoxazoline-catalyzed asymmetric sulfa-Michael/aldol cascade reaction has been successfully developed in our group. To investigate the structure–activity relationship (SAR) of the product stereoisomers, a series of thioheterocyclic nucleosides were synthesized as racemic mixtures under achiral conditions (Scheme 1). In brief, under catalysis with triphenylphosphine, substrates **1A**–**42A**, possessing halogen, alkoxyl, phenyl, amine, or alkyl sulfide groups at the purine C6 position, produced **1B**–**42B** that underwent a sulfa-Michael/aldol cascade reaction with 1,4-dithiane-2,5-diol to afford products **1a**‒**42a** in 43%–71% yields, and their diastereoisomers **1b**–**42b** in 31%–58% yields.

### 2.2 *In vitro* anticancer activities and structure–activity relationships

Cervical cancer cell line (HeLa) and colon cancer cell line (HCT116) were selected to screen the anti-proliferative activity of the target compounds, and 5-fluorouracil (5-FU) and cisplatin was used as a positive control. Tumor cell anti-proliferation activities were determined by cell counting kit-8 (CCK-8) assay.

As expected, compounds **1a** and **1b** without substituents at the 2- or 6-positions of purine had no anticancer activity under 50 μmol/L concentration, consistent with previous SAR of nucleoside clinical drugs. With the 2-position unsubstituted, the influence of substitution at the purine 6-position was assessed (Table 1). Among these products, compounds **5a**/**5b** and **8a**/**8b**, with piperidine and propylthio substitution at the purine 6-position, showed antitumor activity comparable with that of the positive agent on both cell lines. Based on this SAR, further structural modifications at the purine 6-position were carried out to improve the antitumor activity.

**Table 1:**
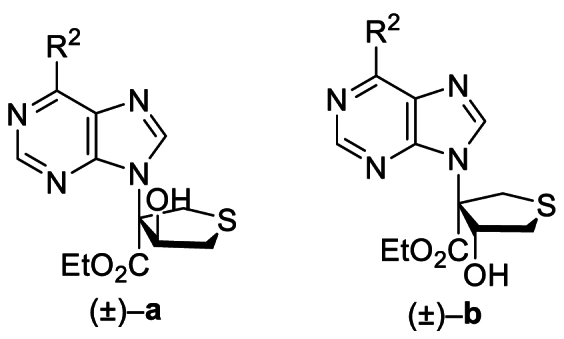
Anticancer activities of thioheterocyclic nucleosides^a^.

Compd.	R^2^	IC_50_ (μmol/L)	
		HeLa	HCT116
**1a**	H	>50	>50
**1b**	H	>50	>50
**2a**	Cl	>50	48.92 ± 2.12
**2b**	Cl	>50	>50
**3a**	Br	>50	8.97 ± 1.03
**3b**	Br	>50	>50
**4a**	N(Me)_2_	9.81 ± 1.12	17.42 ± 1.01
**4b**	N(Me)_2_	11.95 ± 1.57	49.48 ± 2.02
**5a**		2.65 ± 0.52	1.84 ± 0.14
**5b**		4.61 ± 0.87	1.77 ± 0.12
**6a**	OEt	>50	>50
**6b**	OEt	>50	>50
**7a**	OCH_3_	>50	>50
**7b**	OCH_3_	>50	>50
**8a**	S(CH_2_)_2_CH_3_	2.77 ± 0.41	3.43 ± 0.23
**8b**	S(CH_2_)_2_CH_3_	2.55 ± 0.12	2.02 ± 0.07
5-FU	–	4.93 ± 1.02	9.84 ± 2.32
Cisplatin	–	11.25 ± 0.81	5.29 ± 0.25

‒, not applicable.

a Data are mean ± SD values from three independent experiments.

In modifications to compounds **5a**/**5b**, replacing piperidine at the purine 6-position with morpholine (**9a**/**9b**), thiomorpholine (**10a**/**10b**), cycloheximide (**19a**/**19b**) or pyrrolidine (**20a/20b**) reduced antitumor activity slightly. Methylpiperazine substitution (**18a/18b**), especially, was inactive under 50 μmol/L. Similar activity to **5a**/**5b** was observed whether substitution at the piperidine 4-position was electron-donating (Me) (**11a**/**11b**), electron-withdrawing (F, CF_3_ or Cl) (**12a/12b** to **14a/14b**), or bulky (**16a**/**16b** and **17a**/**17b**), indicating that anticancer activity was quite tolerant to modifications at the piperidine *para*-position (Table 2).

**Table 2:**
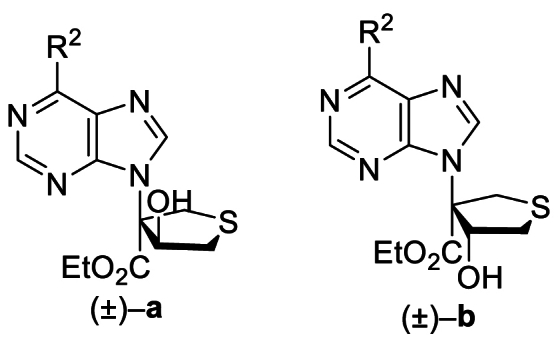
Inhibitory effects of compounds **9a/9b** to **20a/20b** on cancer cell proliferation^a^.

Compd.	R^2^	IC_50_ (μmol/L)	
		HeLa	HCT116
**9a**		10.79 ± 1.03	>50
**9b**		10.22 ± 1.05	17.14 ± 1.12
**10a**		45.77 ± 2.12	18.75 ± 1.04
**10b**		10.23 ± 1.93	10.09 ± 1.01
**11a**		21.78 ± 2.42	1.44 ± 0.13
**11b**		23.39 ± 2.76	0.91 ± 0.27
**12a**		44.45 ± 3.12	1.64 ± 0.27
**12b**		40.88 ± 3.08	3.92 ± 0.87
**13a**		42.00 ± 2.12	5.89 ± 1.02
**13b**		38.73 ± 2.09	2.37 ± 0.36
**14a**		12.12 ± 1.32	15.02 ± 2.16
**14b**		14.27 ± 1.58	18.00 ± 2.37
**15a**		13.71 ± 1.33	43.93 ± 2.11
**15b**		7.71 ± 1.02	32.04 ± 2.09
**16a**		3.34 ± 0.36	1.20 ± 0.09
**16b**		2.86 ± 0.25	4.15 ± 0.82
**17a**		16.99 ± 1.03	4.40 ± 1.02
**17b**		17.00 ± 1.12	4.49 ± 1.13
**18a**		>50	>50
**18b**		>50	>50
**19a**		22.79 ± 1.22	1.19 ± 0.09
**19b**		46.11 ± 2.46	3.05 ± 0.67
**20a**		11.41 ± 1.42	39.14 ± 1.35
**20b**		12.15 ± 1.28	22.78 ± 1.04
5-FU	–	4.93 ± 1.02	9.84 ± 2.32
Cisplatin	–	11.25 ± 0.81	5.29 ± 0.25

‒, not applicable.

a Data are mean ± SD values from three independent experiments.

In modifications to compounds **8a**/**8b**, replacing propylthio at the purine 6-position with benzylthio (**27a**/**27b**), arylthiol (**28a**/**28b** and **29a**/**29b**), and alkylthio (**21a**/**21b** to **26a**/**26b**) maintained anti-cancer activity, which indicated that thio-substitution had a favorable influence on antitumor activity. Propylthio compounds **8a**/**8b** modified by adding fluorine (**30a**/**30b**) or chlorine (**31a**/**31b**) at the propyl 3′-position showed reduced anti-proliferative activity (Table 3).

**Table 3:**
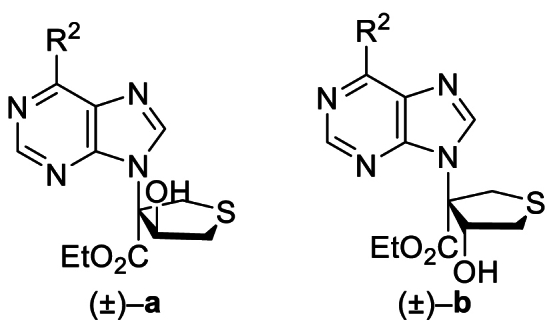
Inhibitory effects of compounds **21a/21b** to **31a/31b** on cancer cell proliferation^a^.

Compd.	R^2^	IC_50_ (μmol/L)	
		HeLa	HCT116
**21a**	SCH_3_	30.97 ± 2.18	44.06 ± 1.36
**21b**	SCH_3_	8.83 ± 0.78	32.73 ± 1.17
**22a**	SCH_2_CH_3_	5.46 ± 1.02	7.56 ± 1.09
**22b**	SCH_2_CH_3_	3.27 ± 0.82	2.42 ± 0.82
**23a**	SCH(CH_3_)_2_	8.45 ± 1.23	4.85 ± 1.02
**23b**	SCH(CH_3_)_2_	3.43 ± 0.76	4.01 ± 0.88
**24a**	SCH_2_C ≡ CH	15.89 ± 2.12	1.81 ± 0.12
**24b**	SCH_2_C ≡ CH	14.93 ± 2.01	1.66 ± 0.13
**25a**	S(CH_2_)_3_CH_3_	9.89 ± 1.02	9.07 ± 0.49
**25b**	S(CH_2_)_3_CH_3_	6.32 ± 0.82	2.84 ± 0.27
**26a**	S(CH_2_)_4_CH_3_	6.07 ± 0.78	11.94 ± 1.32
**26b**	S(CH_2_)_4_CH_3_	>50	11.79 ± 1.27
**27a**	S-Bn	22.31 ± 2.09	14.43 ± 1.64
**27b**	S-Bn	23.88 ± 2.38	5.65 ± 1.40
**28a**	S-Ph-F-*m*	9.47 ± 1.02	12.83 ± 1.08
**28b**	S-Ph-F-*m*	25.77 ± 1.35	15.68 ± 2.00
**29a**	S-Ph-^*t*^Bu-*p*	21.21 ± 2.37	7.60 ± 1.22
**29b**	S-Ph-^*t*^Bu-*p*	15.09 ± 2.08	6.78 ± 1.22
**30a**	S(CH_2_)_2_CH_2_F	10.78 ± 1.07	19.06 ± 1.37
**30b**	S(CH_2_)_2_CH_2_F	12.06 ± 1.12	10.88 ± 1.01
**31a**	S(CH_2_)_2_CH_2_Cl	>50	>50
**31b**	S(CH_2_)_2_CH_2_Cl	>50	>50
5-FU	–	4.93 ± 1.02	9.84 ± 2.32
Cisplatin	–	11.25 ± 0.81	5.29 ± 0.25

‒, not applicable.

a Data are mean ± SD values from three independent experiments.

Based on these results, piperidine or propylthio at the purine 6-position was retained, and the influence of substitution at the purine 2-position was assessed. Anti-proliferative activity was maintained or slightly improved by replacing H with F, Cl, or NH_2_ (**32a**/**32b**‒**37a**/**37b**) (Table 4). On the other hand, compounds **38a**/**38b**‒**42a**/**42b**, which retained F, Cl, and NH_2_ at the purine 2-position but substituted Cl, H, NH_2,_ and morpholine at the purine 6-position, demonstrated no improvement in activity, indicating that piperidine or propylthio at the purine 6-position was crucial for the antitumor activity of these thioheterocyclic nucleosides (Supporting Information Table S3). Considering the cellular anti-proliferative data obtained above, and that the NH_2_ group is a genotoxic structural alert moiety, **5a**/**5b**, **8a**/**8b**, **33a**/**33b**, and **36a**/**36b** bearing H or F at the purine 2-position were submitted to further investigation.

**Table 4:**
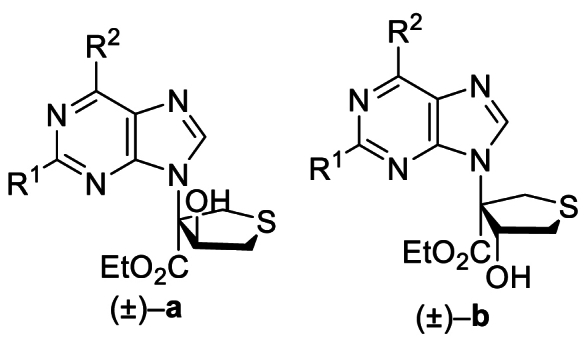
Inhibitory effects of compounds **32a/32b** to **37a/37b** on cancer cell proliferation^a^.

Compd.	R^1^	R^2^	IC_50_ (μmol/L)	
			HeLa	HCT116
**32a**	NH_2_		5.35 ± 1.05	0.72 ± 0.08
**32b**	NH_2_		5.37 ± 1.09	0.81 ± 0.12
**33a**	F		8.73 ± 1.02	0.27 ± 0.01
**33b**	F		8.15 ± 1.08	0.46 ± 0.04
**34a**	Cl		4.86 ± 0.65	7.20 ± 0.39
**34b**	Cl		4.05 ± 0.37	4.30 ± 0.51
**35a**	NH_2_	S(CH_2_)_2_CH_3_	9.44 ± 1.27	0.73 ± 0.11
**35b**	NH_2_	S(CH_2_)_2_CH_3_	8.49 ± 1.02	0.65 ± 0.21
**36a**	F	S(CH_2_)_2_CH_3_	9.68 ± 1.12	0.72 ± 0.12
**36b**	F	S(CH_2_)_2_CH_3_	6.35 ± 1.08	0.49 ± 0.08
**37a**	Cl	S(CH_2_)_2_CH_3_	1.58 ± 0.36	3.81 ± 0.13
**37b**	Cl	S(CH_2_)_2_CH_3_	5.98 ± 1.25	2.70 ± 0.04
5-FU	–	–	4.93 ± 1.02	9.84 ± 2.32
Cisplatin	–	–	11.25 ± 0.81	5.29 ± 0.25

‒, not applicable.

a Data are mean ± SD values from three independent experiments.

To evaluate their spectrum of anti-proliferative activity, further CCK-8 assays were carried out with the selected compounds (**5a**/**5b**, **8a**/**8b**, **33a**/**33b**, and **36a**/**36b**) on MCF-7 (human breast cancer), A375 (human malignant melanoma), DU145 (human prostate cancer), and CT26 (mouse colon cancer) cell lines. The selected compounds had moderate to potent antitumor activity on the four cell lines, indicating their broad spectrum of antitumor activity (Table 5).

**Table 5:**
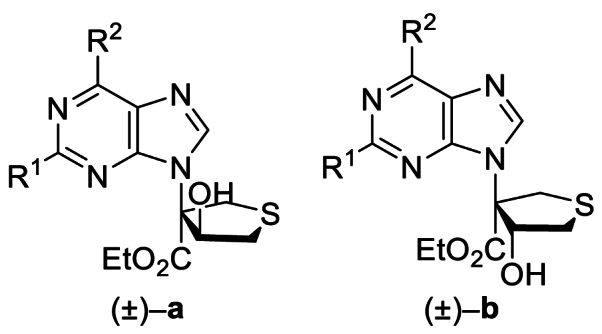
Inhibitory effects of compounds **5a/5b, 8a/8b, 33a/33b,** and **36a/36b** on cancer cell proliferation^a^.

Compd.	R^1^	R^2^	IC_50_ (μmol/L)			
			MCF-7	A375	DU145	CT26
**5a**	H		11.21 ± 1.04	11.75 ± 1.12	42.83 ± 2.12	2.33 ± 0.14
**5b**	H		35.21 ± 2.14	7.52 ± 1.03	42.52 ± 2.11	2.42 ± 0.11
**33a**	F		8.45 ± 1.03	2.34 ± 0.12	21.11 ± 1.21	0.57 ± 0.13
**33b**	F		7.47 ± 1.17	14.67 ± 0.09	18.56 ± 2.12	0.79 ± 0.14
**8a**	H	S(CH_2_)_2_CH_3_	28.25 ± 2.16	4.52 ± 1.12	29.30 ± 1.37	2.58 ± 0.15
**8b**	H	S(CH_2_)_2_CH_3_	25.39 ± 2.45	2.82 ± 0.03	10.97 ± 1.04	2.37 ± 0.08
**36a**	F	S(CH_2_)_2_CH_3_	11.26 ± 1.27	4.27 ± 1.02	9.48 ± 1.11	0.94 ± 0.11
**36b**	F	S(CH_2_)_2_CH_3_	14.14 ± 1.24	1.14 ± 0.17	6.59 ± 1.36	0.85 ± 0.09
5-FU	–	–	>50	8.62 ± 1.21	21.38 ± 2.17	3.12 ± 0.13
Cisplatin	–	–	6.32 ± 0.74	9.42 ± 0.87	9.65 ± 0.48	22.48 ± 1.28

‒, not applicable.

a Data are mean ± SD values from three independent experiments.

The stereochemistry of chiral compounds usually has a substantial effect on their biological activity^20^. To assess the activity of their stereoisomers, compounds **33a** and **36b** were resolved *via* HPLC to afford two pairs of enantiomers (**33aa**/**33 ab** and **36ba**/**36bb**), and the absolute configurations of compounds (3*S*,4*S*)-**33aa** and (3*S*,4*R*)-**36ba** were determined by single crystal X-ray diffraction analysis (Supporting Information Figs. S1 and S2, Tables S1 and S2). No significant difference was observed between the antitumor activities of the enantiomers (Table 6). Therefore, racemic mixtures **33a** and **36b** were used in further antitumor mechanism investigations. Moreover, most compounds showed higher antitumor activity against HCT116 than HeLa cells, so the former was selected for further experiments.

**Table 6:**
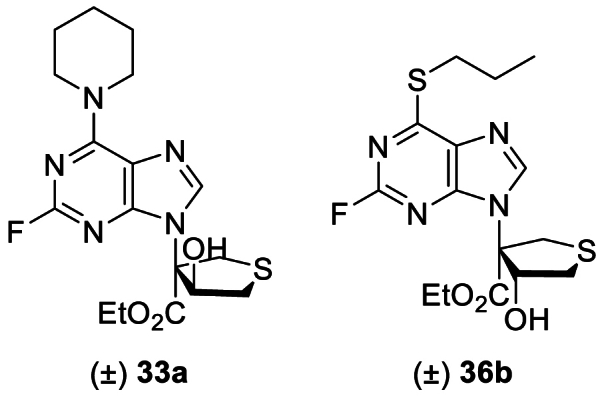
Inhibitory effects of compounds **33a** (*rac*-, 3*S*,4*S*- and 3*R*,4*R-*) and **36b** (*rac*-, 3*S*,4*R*- and 3*R*,4*S*-) on cancer cell proliferation^a^.

Compd.	Configuration	IC_50_ (μmol/L)	
		HeLa	HCT116
**33a**	*rac*	8.73 ± 1.02	0.27 ± 0.01
**33aa**	3*S*,4*S*	6.17 ± 1.01	0.58 ± 0.17
**33 ab**	3*R*,4*R*	10.05 ± 1.02	0.21 ± 0.13
**36b**	*rac*	6.35 ± 1.08	0.49 ± 0.08
**36ba**	3*S*,4*R*	5.52 ± 1.12	0.89 ± 0.14
**36bb**	3*R*,4*S*	9.49 ± 1.18	0.37 ± 0.08
5-FU	–	4.93 ± 1.02	9.84 ± 2.32
Cisplatin	–	11.25 ± 0.81	5.29 ± 0.25

‒, not applicable.

a Data are mean ± SD values from three independent experiments.

### 2.3 Proliferation inhibition of compounds **33a** and **36b**

**Figure 3:**
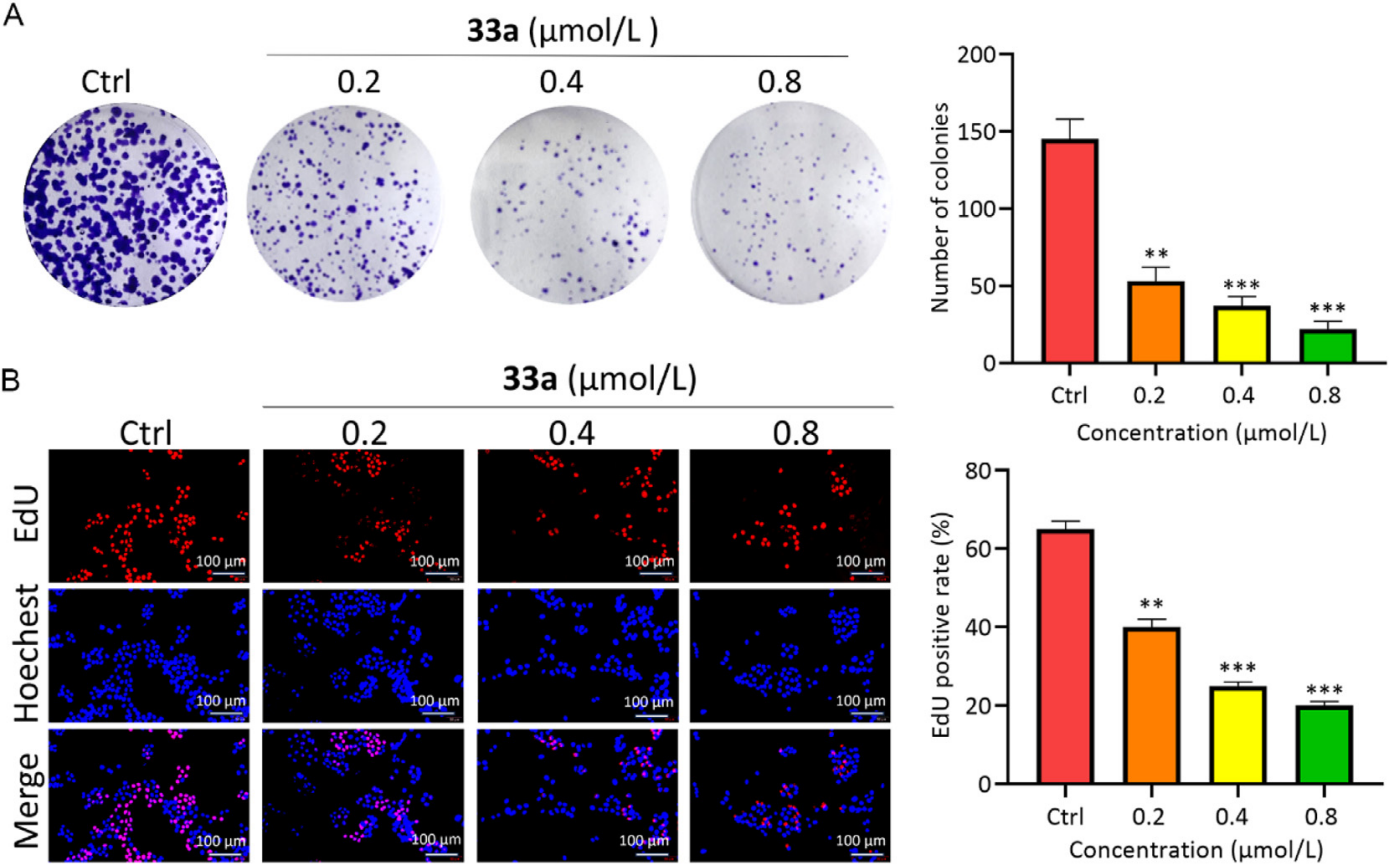
Proliferation inhibition of compound **33a**. (A) Colony formation analysis of HCT116 cell line with **33a** (cells were incubated for 48 h, followed by incubation in fresh media for an additional 14 days). (B) EdU assay analysis of HCT116 cell line with **33a** for 48 h. Data are mean ± SD (*n* = 3), ∗*P* < 0.01, ∗∗∗*P* < 0.001, and ∗∗∗∗*P* < 0.0001 compared with the control group.

Colony formation and EdU assay were applied to assess proliferation inhibition. Both **33a** and **36b** could reduce the colony formation number in a dose-dependent manner compared with the control group (Fig. 3A and Supporting Information Fig. S3A). Moreover, the EdU assay, a cell proliferation detection test, similarly demonstrated the anti-proliferation effects of **33a** and **36b** on HCT116 cells, with significantly fewer EdU-positive cells than in the control group (Fig. 3B and Fig. S3B).

### 2.4 Migration and invasion inhibition of compounds **33a** and **36b**

**Figure 4:**
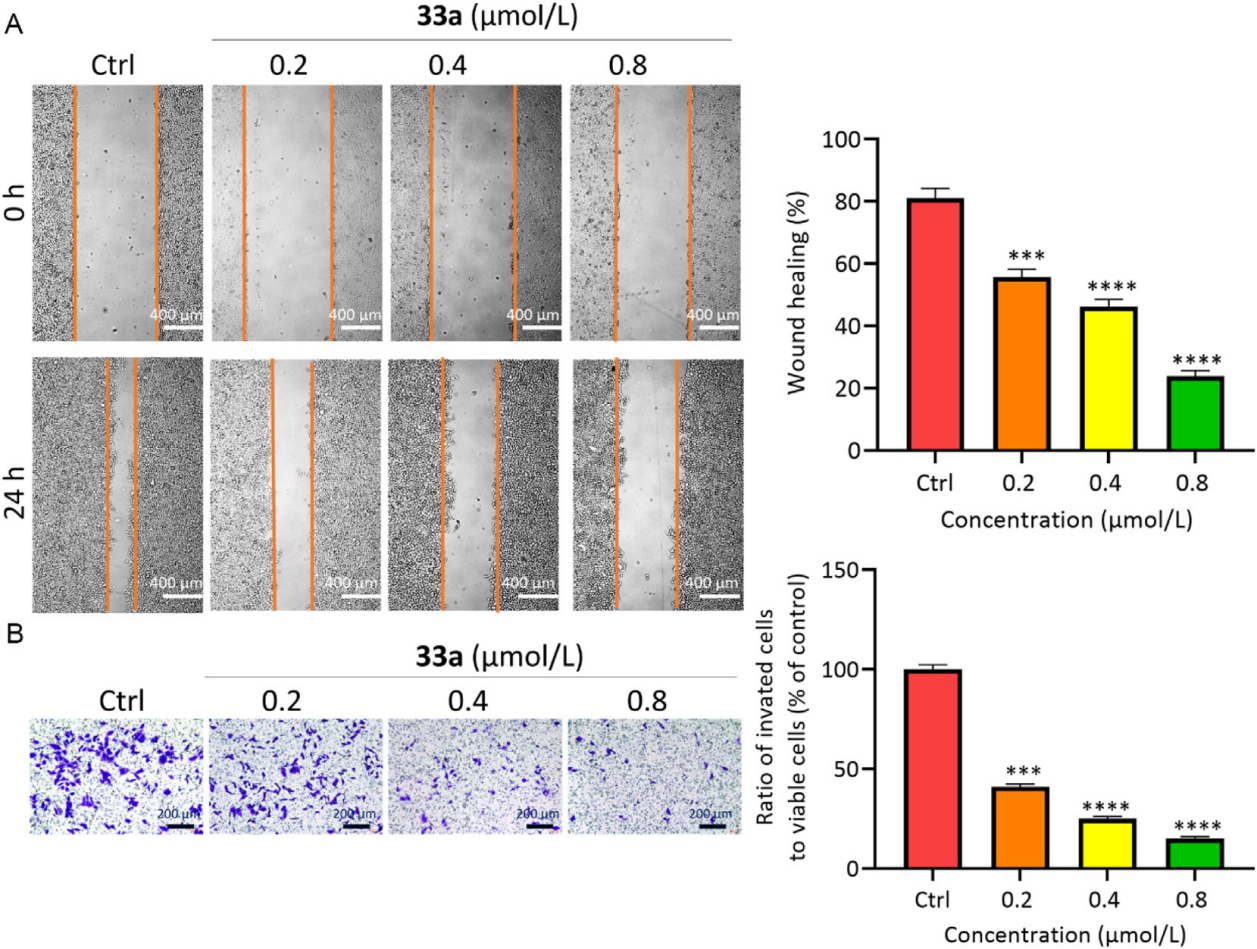
Migration and invasion inhibition of compound **33a**. (A) Wound healing assays of HCT116 cell line with **33a** for 24 h. Scale bar = 400 μm. (B) Transwell assay of HCT116 cell line with **33a** for 24 h. Scale bar = 200 μm. Data are mean ± SD (*n* = 3), ∗∗∗*P* < 0.001, and ∗∗∗∗*P* < 0. 0001 compared with the control group.

Compounds **33a** and **36b** could significantly delay the wound healing ability of HCT116 cells in a dose-dependent manner (Fig. 4A and Supporting Information Fig. S4A). In the invasion test, compounds **33a** and **36b** distinctly reduced the number of HCT116 cells passing through the matrix membrane, also in a dose-dependent manner (Fig. 4B, Fig. S4B). These results showed that compounds **33a** and **36b** could notably inhibit the invasion and migration of HCT116 cells.

### 2.5 Cell cycle arrest and apoptosis induced by compounds **33a** and **36b**

**Figure 5:**
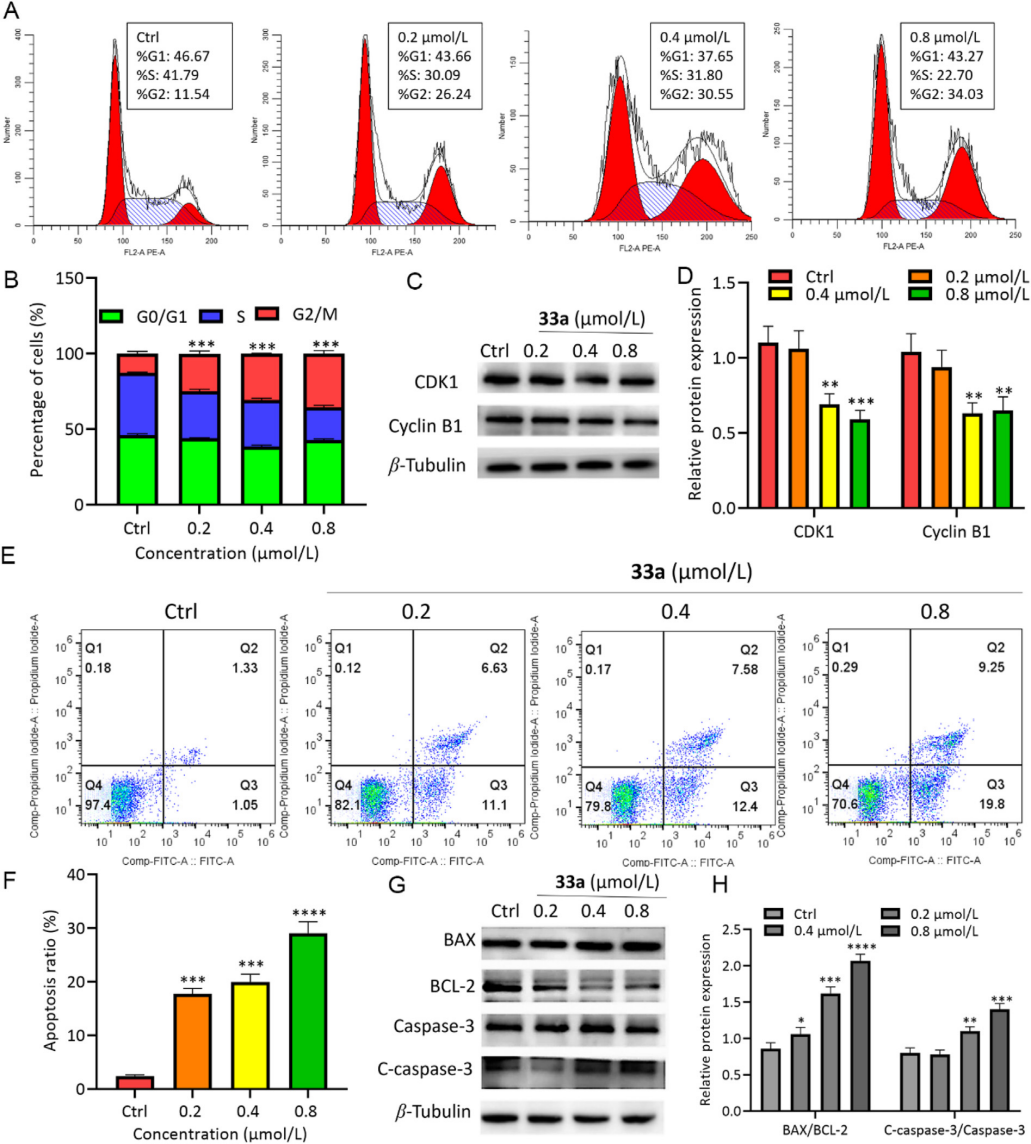
Cell cycle arrest and apoptosis induced by compound **33a** in HCT116 for 48 h. (A,B) G2/M block induced by compound **33a** treated for 48 h. (C,D) Western blot analysis of cell cycle-related proteins. (E,F) Apoptosis induced by compound **33a**. (G–H) Western blot analysis of cell apoptosis-related proteins. Data are mean ± SD (*n* = 3), ∗*P* < 0.05, ∗∗*P* < 0.01, ∗∗∗*P* < 0.001, and ∗∗∗∗*P* < 0.0001 compared with the control group.

Considering that heterocyclic nucleosides could inhibit the growth of tumor cells by interfering with the cell cycle process^21^, flow cytometry was conducted to explore the cell cycle of HCT116 when treated with **33a** and **36b**. As shown in Fig. 5 and Supporting Information Fig. S5, compounds **33a** and **36b** increased the percentage of G2/M cells in a dose-dependent manner (Fig. 5A and B, Fig. S5A and S5B). G2/M transition is mainly regulated by cyclin B1-CDK1^22^. Western blotting showed that the expression of cyclin B1 and CDK1 in HCT116 cells decreased in a dose-dependent manner under treatment with **33a** and **36b** (Fig. 5C and D, and Fig. S5C and S5D). These results suggest that compounds **33a** and **36b** significantly promoted cell cycle arrest at the G2/M phase of HCT116.

Based on these observations, Annexin-V and PI staining were used to detect the proportion of apoptotic cells by flow cytometry. As shown in Fig. 5E and F, and Fig. S5E and S5F, compounds **33a** and **36b** induced apoptosis in a dose-dependent manner. In addition, apoptosis markers BCL-2, BAX, and Cleaved caspase-3 were determined by western blotting, which indicated increased BAX and Cleaved caspase-3 protein levels, and a significant decrease in the expression of BCL-2 (Fig. 5G and H, and Fig. S5G and S5H). Immunofluorescence experiments revealed similar results for BCL-2 and BAX (Supporting Information Fig. S6). These results suggest that compounds **33a** and **36b** could significantly promote the apoptosis of HCT116.

### 2.6 ROS production, DNA damage, ER stress, and mitochondrial cell death pathways induced by compounds **33a** and **36b**

**Figure 6:**
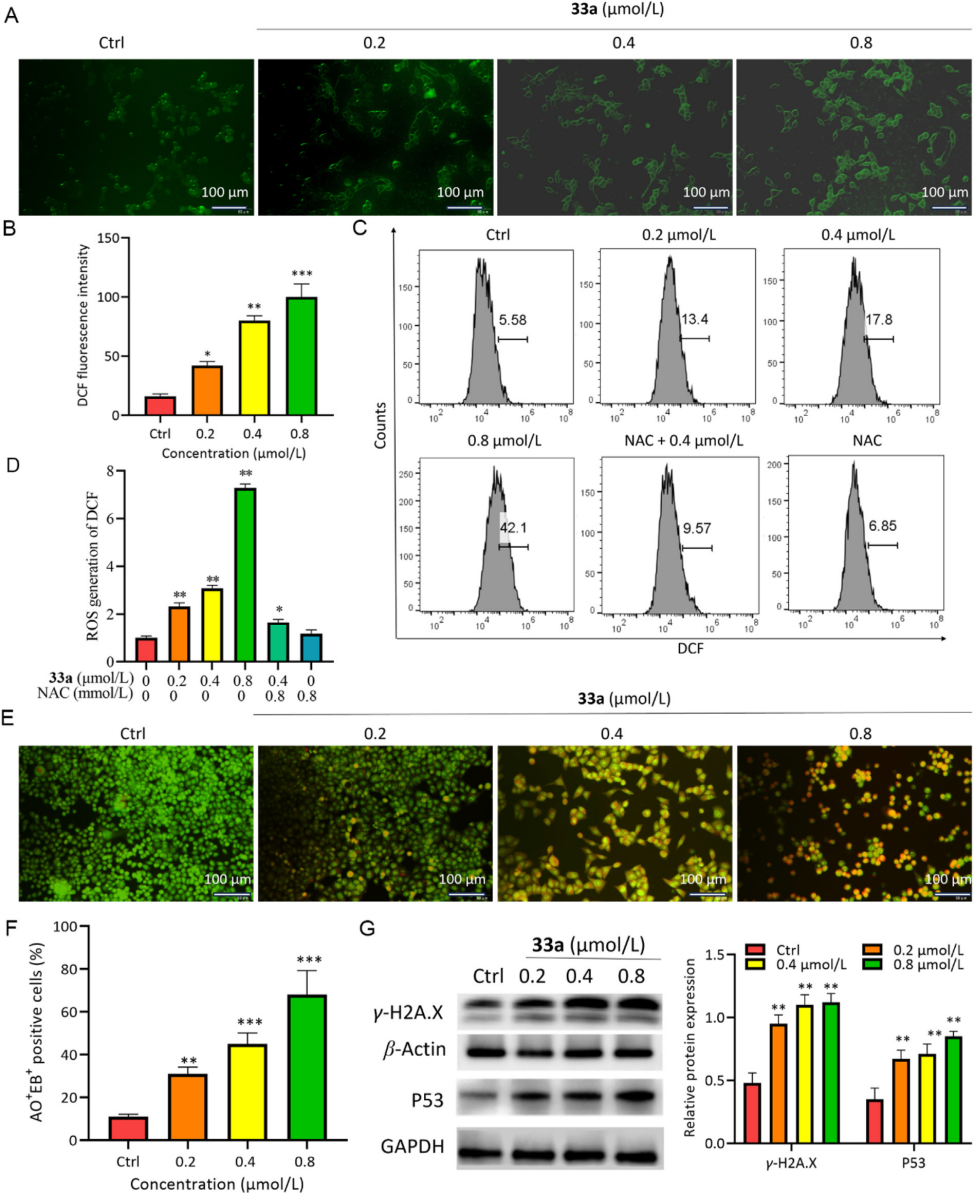
ROS production and DNA damage induced by compound **33a** over 48 h. (A,B) ROS levels were examined by DCFH-DA. Scale bar = 100 μm. (C,D) ROS levels were examined by DCFH-DA using flow cytometry. (E,F) AO staining of **33a**-treated HCT116 cells using fluorescence microscopy. Scale bar = 100 μm. (G–H) Western blot analysis of the expression levels of *γ*-H2AX and P53. Data are mean ± SD (*n* = 3), ∗*P* < 0.05, ∗∗*P* < 0.01, ∗∗∗*P* < 0.001, and ∗∗∗∗*P* < 0.0001 compared with the control group.

Elevated ROS levels are reported to induce apoptosis through DNA damage, endoplasmic reticulum (ER) stress, and mitochondrial dysfunction^23^. ROS assay results indicated that compounds **33a** and **36b** significantly increased ROS levels in a dose-dependent manner compared to the control group (Fig. 6A and B, and Supporting Information Fig. S7A). As expected, these ROS levels were reduced in HCT116 cells pretreated with the ROS-specific scavenger N-acetylcysteine (NAC) (Fig. 6C and D), compounds **33a** and **36b** decreased HCT116 cell viability, an effect partially or completely reversed by different concentrations of NAC(Supporting Information Fig. S8A and Fig. S8D). AO staining was used to detect DNA damage caused by compounds **33a** and **36b**, as shown in Fig. 6E and F, and Fig. S7B, and the fluorescence changed from green to orange on increasing the concentration of compounds **33a** and **36b**, indicating that DNA damage occurred. Western blot analysis results suggest that DNA damage-related proteins *γ*-H2A.X, P53, and DNA damage repair-related proteins PARP-1 were significantly elevated in **33a** and **36b**-treated HCT116 cells compared with the control group (Fig. 6G, Fig. S7C, Fig. S8B and S8C, S8E and S8F). Furthermore, pretreatment with NAC alleviated the increase in *γ*-H2A.X and PARP-1 protein expression in HCT116 cells under compounds **33a** and **36b** treatment. These findings suggest that compounds **33a** and **36b** induced DNA damage by modulating the production of ROS.

**Figure 7:**
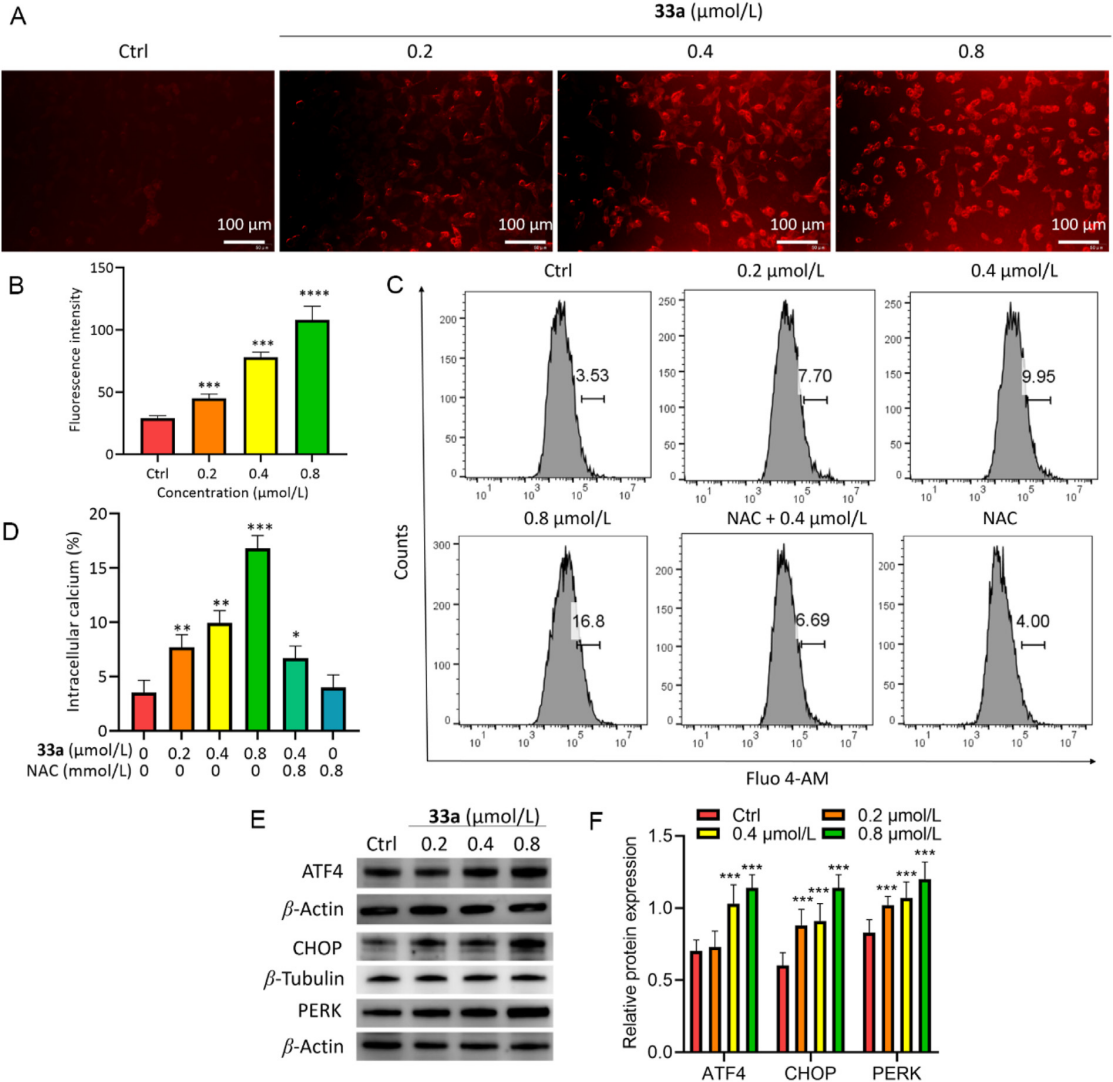
ER Stress induced by compound **33a** over 48 h. (A,B) ER stress was evaluated by ER-Tracker Red using fluorescence microscopy. Scale bar = 100 μm. (C,D) Calcium fluorescence intensity of Fluo-4 AM by flow cytometry. (E,F) Western blot analysis of ATF4, CHOP, and PERK, expression. Data are mean ± SD (*n* = 3), ∗*P* < 0.05, ∗∗*P* < 0.01, ∗∗∗*P* < 0.001, and ∗∗∗∗*P* < 0.0001 compared with the control group.

Next, the effect of compounds **33a** and **36b** on ER stress was investigated using ER-Tracker Red in HCT116 cells, revealing ER stress activation on treatment with compounds **33a** and **36b** (Fig. 7A and B, and Fig. S7D). The ER contains a large amount of Ca^2+^, which plays a critical role in intracellular Ca^2+^ homeostasis^24^. The calcium fluorescence intensity was examined by using the Fluo-4 AM kit. As shown in Fig. 7C and D, the fluorescence intensity increased significantly when treated with compound **33a**, which was decreased in the NAC and **33a**-treated group, indicating that **33a** increased intracellular Ca^2+^ concentration, which was inhibited by NAC. In addition, ER stress-related proteins PERK, ATF4, and CHOP increased rapidly in **33a** and **36b**-treated HCT116 cells (Fig. 7E and F, and Fig. S7E).

**Figure 8:**
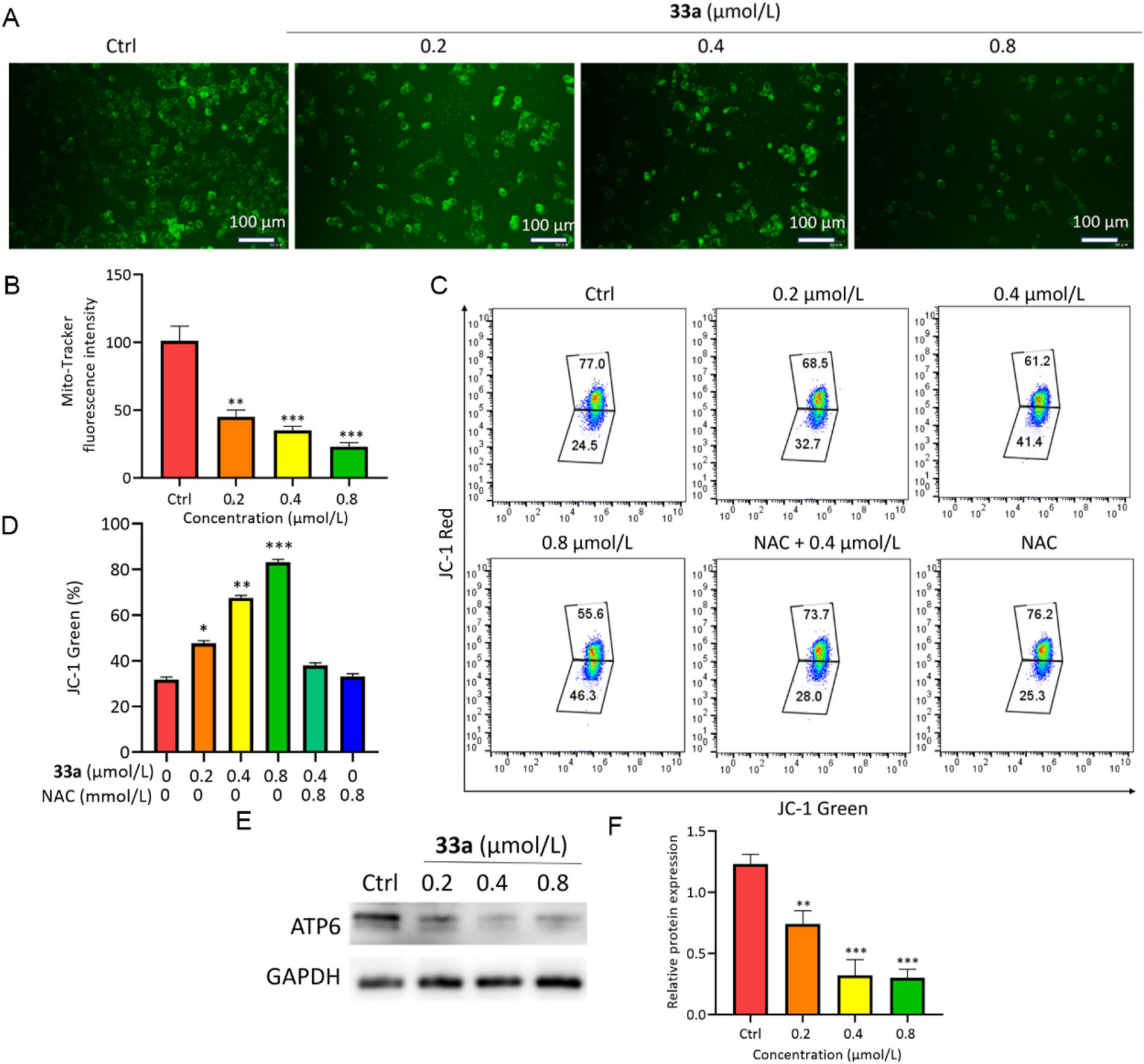
Mitochondrial cell death pathways induced by compound **33a** over 48 h. (A,B) Mitochondrial damage found by Mito-Tracker Green probe. Scale bar = 100 μm. (C,D) Mitochondrial membrane potential. (E,F) Western blot analysis of ATP6 expression level. Data are mean ± SD (*n* = 3), ∗*P* < 0.05, ∗∗*P* < 0.01, ∗∗∗*P* < 0.001, and ∗∗∗∗*P* < 0.0001 compared with the control group.

Mitochondrial structure changes were also evaluated using Mito-Tracker Green fluorescent probes, showing a gradual decrease in the fluorescence intensity of HCT116 cells on increasing the concentration of **33a** and **36b** and indicating damage to the cellular mitochondria (Fig. 8A and B, and Fig. S7F). To further examine the effect of **33a** on mitochondria, the mitochondrial membrane potential was measured by flow cytometry using a JC-1 kit. As shown in Fig. 8C and D, after exposure to 0.2, 0.4, and 0.8 μmol/L **33a**, red fluorescence decreased and green fluorescence increased in the HCT116 cells. Western blotting results indicated that the expression of mitochondrial-related protein ATP6 in HCT116 cells treated with compounds **33a** and **36b** was down-regulated compared to the control group (Fig. 8E and F, and Fig. S7G).

Western blot analysis results indicate that the levels of *γ*-H2A.X, PARP-1, and PERK were significantly elevated in HCT116 cells treated with **33a** and **36b** compared to the control group, whereas ATP6 was significantly downregulated (Fig. S8B and S8C, S8E and S8F). Furthermore, pretreatment with NAC alleviated the increase in *γ*-H2AX, PARP-1, and PERK protein expression and increased ATP6 protein levels in HCT116 cells under compounds **33a** and **36b** treatment. Based on these results, compounds **33a** and **36b** induce ROS-mediated DNA damage, ER stress, and mitochondrial dysfunction, ultimately initiating apoptosis in HCT116 cells.

As shown in Fig. S7, compounds **33a** and **36b** decreased HCT116 viability, which was reversed partially or fully by ROS scavenger NAC (Figs. S8A and S7D). Pretreatment with endoplasmic reticulum stress (ERS) inhibitor 4-PBA increased the viability of HCT116 cells treated with **33a** and **36b**, suggesting ROS-mediated ERS induction (Fig. S8G). Similarly, mitochondrial protector elamipretide increased viability, implying compounds **33a** and **36b** disrupt mitochondrial function through ROS modulation (Fig. S8H). The results above indicate compounds **33a** and **36b** induced ROS-mediated DNA damage, ERS, and mitochondrial dysfunction, and inhibited cell proliferation in HCT116 cells.

### 2.7 Autophagy inhibited by compounds **33a** and **36b**

**Figure 9:**
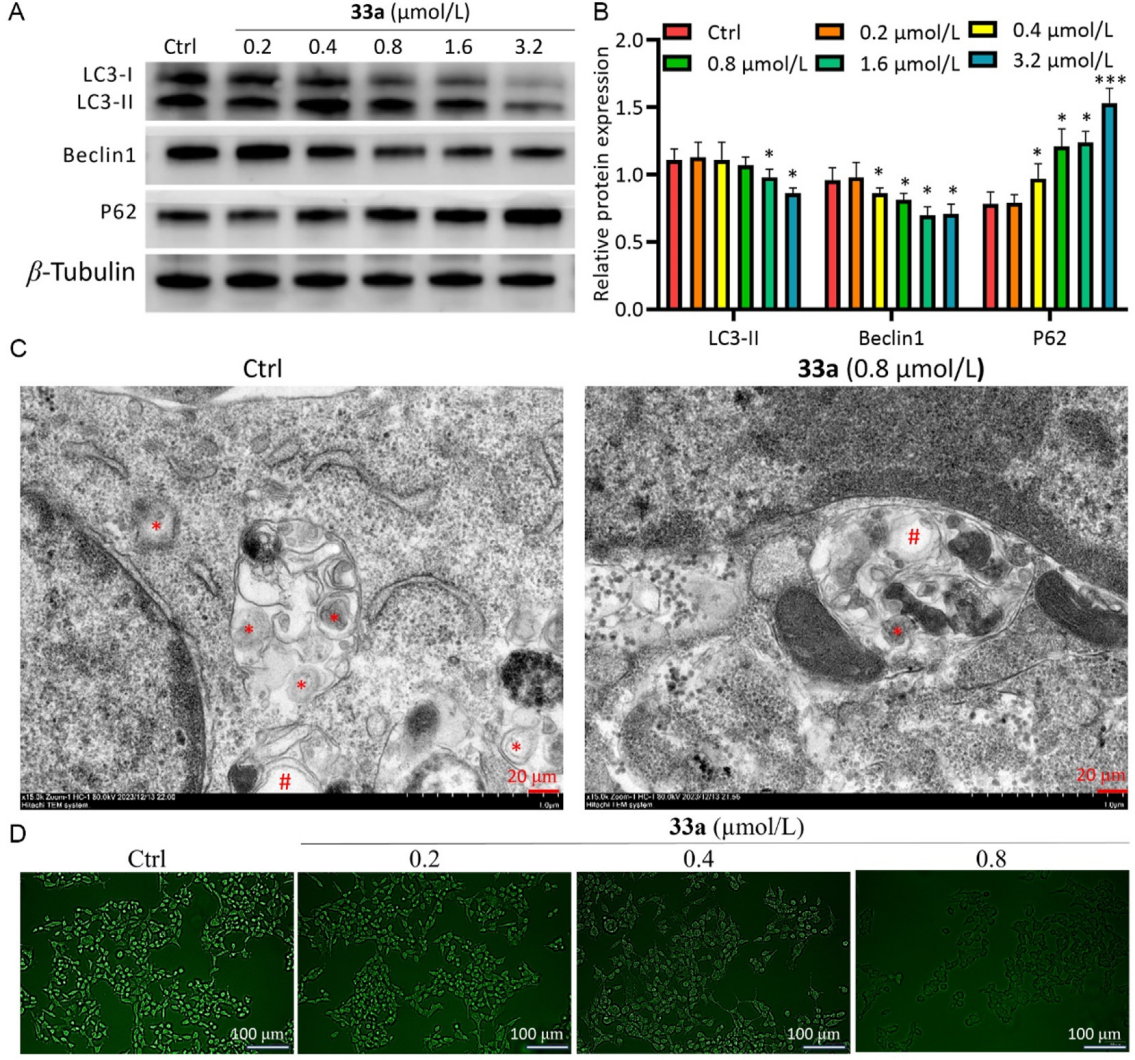
Autophagy inhibition of **33a**-treated HCT116 cells over 48 h. (A,B) Autophagy is inhibited by compound **33a**. (C) Autophagic structures including autolysosomes (∗) and autophagic vacuoles (#) detected by transmission electron microscopy in **33a**-treated cells. Scale bar = 20 μm. (D) MDC staining. Scale bar = 100 μm. Data are mean ± SD (*n* = 3), ∗*P* < 0.05, ∗∗*P* < 0.01, and ∗∗∗*P* < 0.001 compared with the control group.

One of the most important forms of programmed cell death is autophagy, whose participation in tumor progression and therapy has been widely investigated^25^. P62, LC3, and Beclin1 are recognized as markers of autophagy^26^^,^^27^^,^^28^. Western blotting results showed that P62 protein was up-regulated and LC3-II and Beclin1 protein were down-regulated in a dose-dependent manner under compound **33a** treatment. Moreover, it was found by transmission electron microscopy and MDC staining that compound **33a** reduced the number of autophagosomes compared with the control group, indicating that **33a** could inhibit autophagy in HCT116 (Fig. 9). However, compound **36b** behaved differently to **33a** in that **36b** could up-regulate the protein levels of P62 but showed no obvious effect on LC3-II and Beclin1 (Supporting Information Fig. S9).

### 2.8 Target prediction for colon cancer

**Figure 10:**
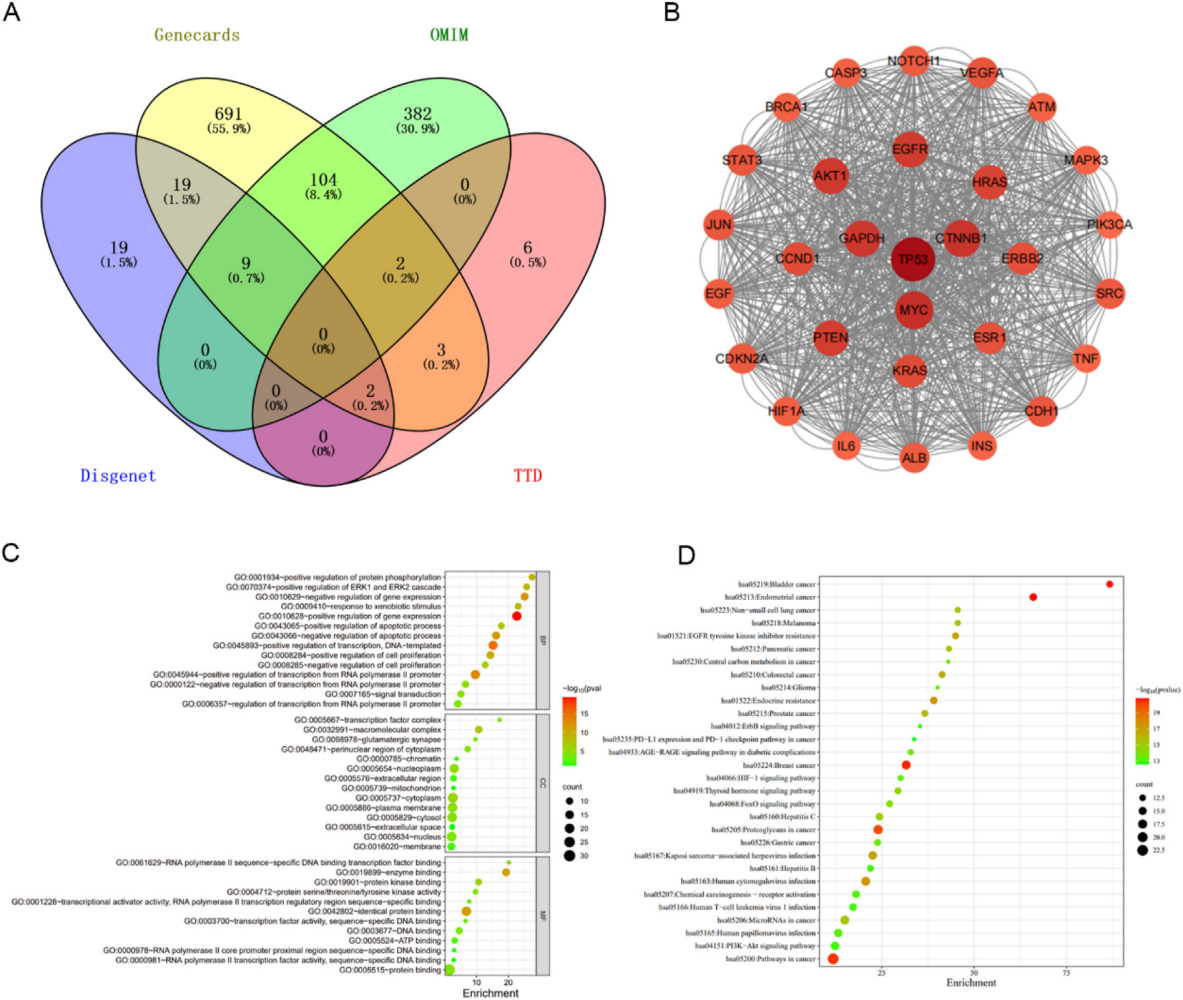
Biological information analysis of colon cancer. (A) Predicted targets. (B) PPI of the hub targets. (C) Gene ontology (GO) biological process of the hub targets. (D) Kyoto Encyclopedia of Genes and Genomes (KEGG) analysis.

To predict the potential target of compounds **33a** and **36b**, biological information analysis was conducted focusing on colon cancer using four datasets (Disgenet, OMIM, TTD, and GeneCards databases). A total of 1237 colon cancer-related targets were identified (Fig. 10A), and these potential targets were submitted to the STRING database and Cytoscape3.8.2 software to calculate the degree value. Three proteins P53, c-MYC, and *β*-Catenin exhibited higher degree values, larger areas, and darker node color (Fig. 10B). Furthermore, GO biological process results showed that the key targets mainly focused on regulating apoptosis and mitochondrial tissue dysfunction (Fig. 10C and D), so P53, c-MYC, and *β*-Catenin were considered to be the essential nodes.

### 2.9 Analysis of gene expression changes in **33a** treatment

**Figure 11:**
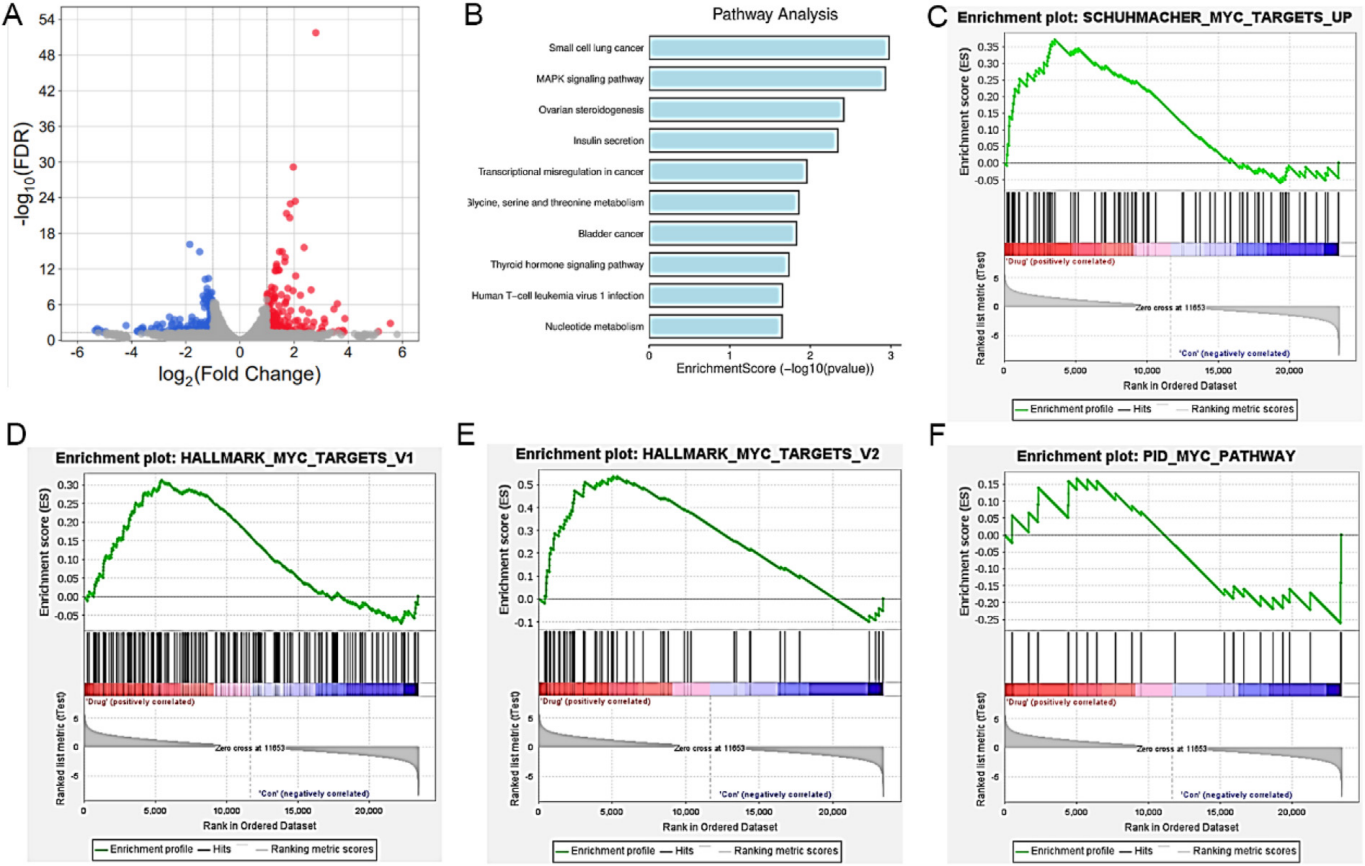
Pathway enrichment analysis of HCT116 cells treated with compound **33a**. (A) Functional annotation and enrichment of DEGs. Volcano plot displaying the upregulated DEGs (red) and downregulated DEGs (blue). (B) KEGG pathway enrichment analysis. (C–F) GSEA analysis results.

To identify the pathways involved in **33a**-induced antiproliferation and apoptosis, RNA-sequencing (RNA-seq) analysis was used to explore differentially expressed genes between HCT116 cells treated with compound **33a** (0.8 μmol/Lol/L) and the control. A total of 265 genes were significantly upregulated after 48 h of compound **33a** treatment, while 221 genes were significantly downregulated. Among the top 10 genes with the most significant fold change (Supporting Information Table S4), *c-MYC* and *DCLK1* were down-regulated, whereas 8 genes including *NGFR*, *SLC44A2*, *TP53*, *C5AR1*, *SLC16A6*, *SDSL*, *PNCK*, and *SULF2* were up-regulated (Fig. 11A). However, there is not much correlation between *SDSL* gene expression and the occurrence and progression of cancer gene^29^, and Expression Profiling Interactive Analysis (http://gepia.cancer-pku.cn/) indicated SULF2, SLC44A2 and SLC16A6 were no obvious change between colon cancer tissue and normal tissue (Supporting Information Fig. S10). Thus, NGFR, C5AR1, PNCK, DCLK1, TP53, and c-MYC were further verified by Western blot analysis which revealed that only P53 and c-MYC showed corresponding changes when treated with **33a** (Supporting Information Figs. S11 and S12).

To analyze the inhibitory effect of **33a**, KEGG pathway analysis on downregulated differentially expressed genes (DEGs) was further performed to give 10 signaling pathways, including MAPK signaling pathway, transcriptional misregulation in cancer, thyroid hormone signaling pathway, and nucleotide metabolism (Fig. 11B). Furthermore, gene set enrichment analysis (GSEA) of downregulated DEGs were conducted and found that different DEGs were enriched in HALLMARK_MYC_TARGETS_V1, HALLMARK_MYC_TARGETS_V2, PID_MYC_PATHWAY and SCHUHMACHER_MYC_TARGETS_UP (Fig. 11C‒F), suggesting that compound **33a** may influence cell survival by inhibiting *c-MYC* and its downstream effector molecules. Taken together, the c-MYC signaling pathway was considered to be crucial for **33a**-induced antitumor activities.

c-MYC coordinately regulates nucleotide synthesis enzymes and other metabolic enzymes to achieve this increased nucleotide production^30^. Further analysis from KEGG pathway analysis and transcriptome sequencing data indicated that **33a** exerts influences on RNA polymerase and nucleic acid metabolism (Supporting Information Fig. S13A and S13B). The uridine phosphorylase 1 (UPP1) protein, a pivotal regulator in intracellular nucleotide metabolism, facilitates the conversion of 5-FU within cells into several active metabolites known as fluoronucleotides. Subsequently, these fluoronucleotides are erroneously incorporated into both DNA and RNA structures, leading to the suppression of thymidylate synthase^31^. Notably, 5-FU itself does not affect the level of UPP1 protein. However, unlike the anticancer mechanism of 5-FU, the expression of UPP1 in HCT116 cells decreased in a dose-dependent manner when treated with **33a** in this study (Supporting Information Fig. S14C and S14D). These findings suggested that **33a** can decrease UPP1 protein levels.

### 2.10 Interaction identification of compounds **33a** and **36b**

**Figure 12:**
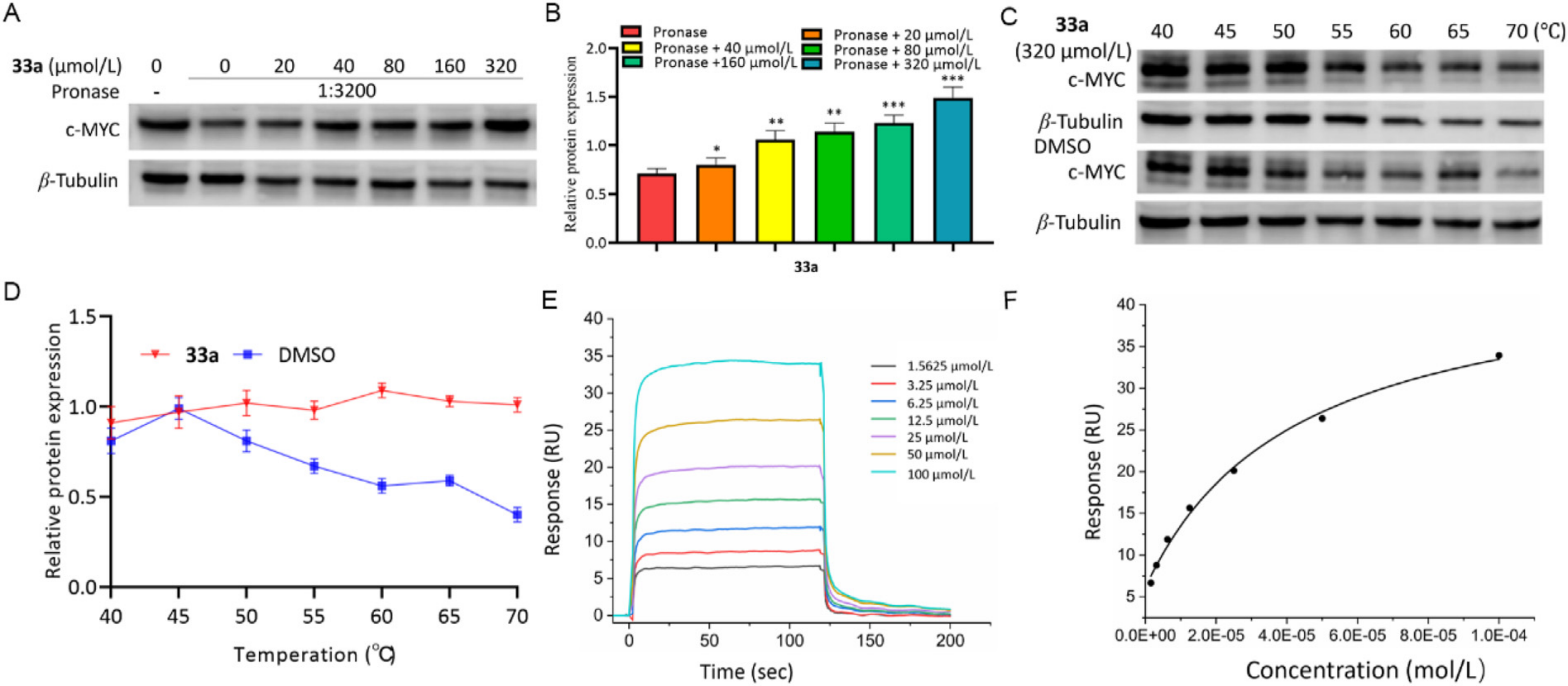
Interaction of compound **33a** and c-MYC. (A,B) c-MYC protected by compound **33a** against proteolysis in DARTS assay. (C,D) Compound **33a** binding with c-MYC in CETSA experiment. (E,F) KD value between compound **33a** and c-MYC determined by SPR analysis. Data are mean ± SD (*n* = 3), ∗*P* < 0.05, ∗∗*P* < 0.01, and ∗∗∗*P* < 0.001 compared with the control group.

Based on the biological information analysis and RNA-seq analysis above, the affinities of compounds **33a and 33b** with NGFR, C5AR1, PNCK, DCLK1, P53, c-MYC, and *β*-Catenin were determined by drug affinity responsive target stability (DARTS) assay. Compounds **33a** and **36b** were shown to increase the stability of c-MYC in the process of protease hydrolysis with a gradual increase in concentration, whereas no obvious effect on NGFR, C5AR1, PNCK, DCLK1, P53, and *β*-Catenin was observed (Fig. 12A and B, Fig. S14A‒S14D). Then, cellular thermal shift assay (CETSA) showed that compounds **33a** and **36b** could increase the thermal stability of c-MYC in HCT116 cells from 40 to 70 °C (Fig. 12C and D, and Fig. S14E and S14F). Subsequently, SPR experiments indicated KD values of 42.88 and 205.80 μmol/L for the concentration-dependent interactions of **33a** and **36b** with c-MYC, respectively (Fig. 12E and F, and Fig. S14G‒S14H).

**Figure 13:**
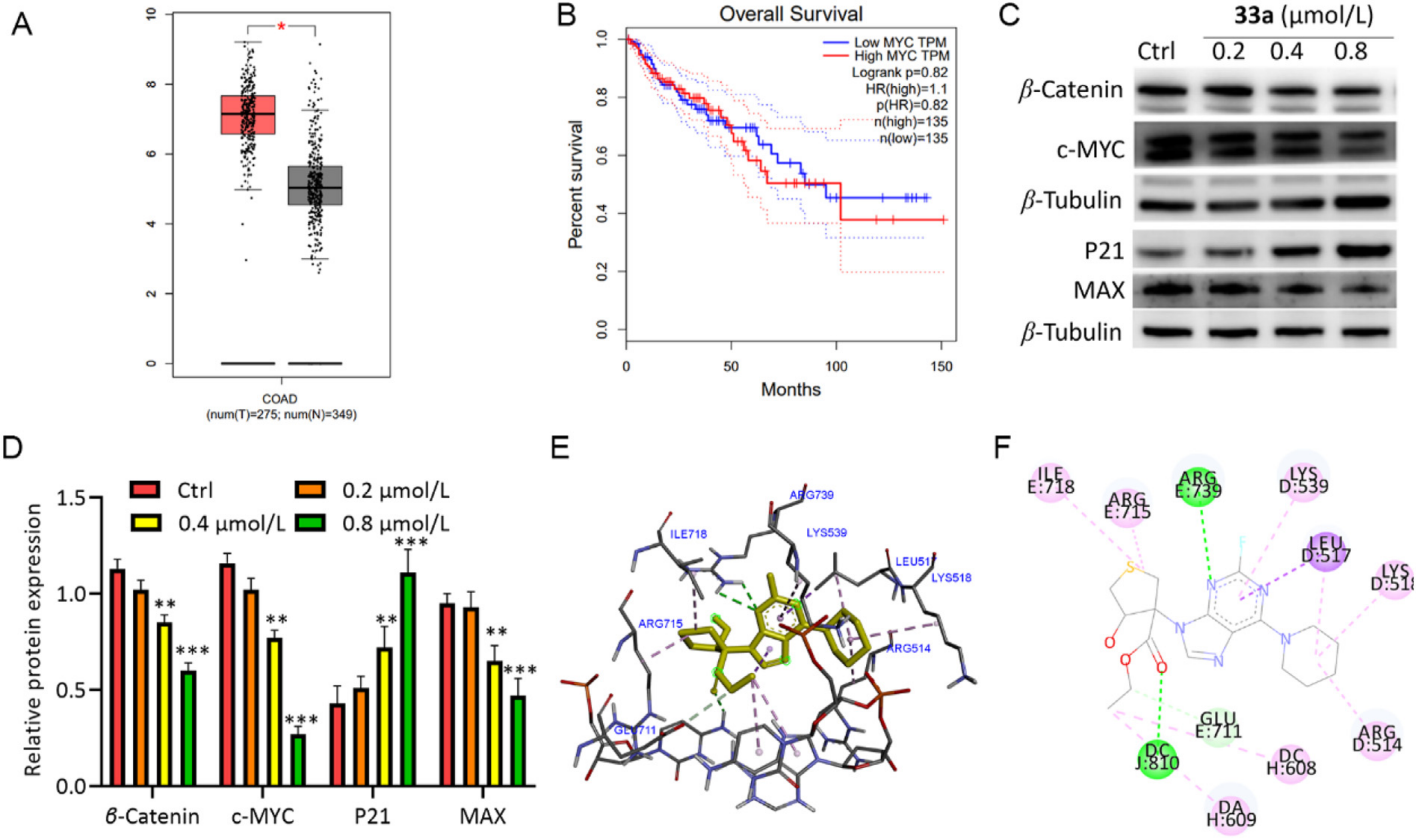
Interaction of compound **33a** and c-MYC (PDB code: 1NKP). (A) The expression of c-MYC in colon cancer tissue and normal tissue. (B) Patient survival rate. (C,D) Expression levels of *β*-Catenin, c-MYC, P21, and MAX. (E,F) 3D and 2D (hydrogen bonding (green) and hydrophobic bond (purple)) interactions of compound **33a** with c-MYC. Data are mean ± SD (*n* = 3), ∗*P* < 0.05, ∗∗*P* < 0.01, and ∗∗∗*P* < 0.001 compared with the control group.

Gene Expression Profiling Interactive Analysis (http://gepia.cancer-pku.cn/) indicated significantly higher expression of c-MYC in colon cancer tissue than in normal tissue (Fig. 13A). Higher c-MYC expression correlates with a lower survival rate of patients with colon cancer (Fig. 13B). Next, western blotting experiments were conducted to analyze the effect of compounds **33a** and **36b** on c-MYC and the downstream protein levels of *β*-Catenin, P21, and MAX. As shown in Fig. 13C and D, and Fig. S14I and S14J, c-MYC, *β*-Catenin, and MAX were significantly decreased, whereas the expression of p21 increased significantly in a dose-dependent manner, which indicated that compounds **33a** and **36b** could bind with c-MYC and block the c-MYC signal path in colon cancer.

Molecular docking was carried out to further explore the binding mode between compounds **33a** and **36b** with c-MYC, showing interaction of both compounds with c-MYC *via* hydrogen bonding or hydrophobic bonds at the Ile718, Arg715, Arg739, Arg514, Leu517, Lys539, Glu711 or His506, Lys502, Leu509, and Thr505 sites (Fig. 13E and F, and Fig. S14K and S14L). These results further confirmed the binding mode between compounds **33a** and **36b** and c-MYC.

### 2.11 Silencing of c-MYC inhibited proliferation and induced apoptosis

**Figure 14:**
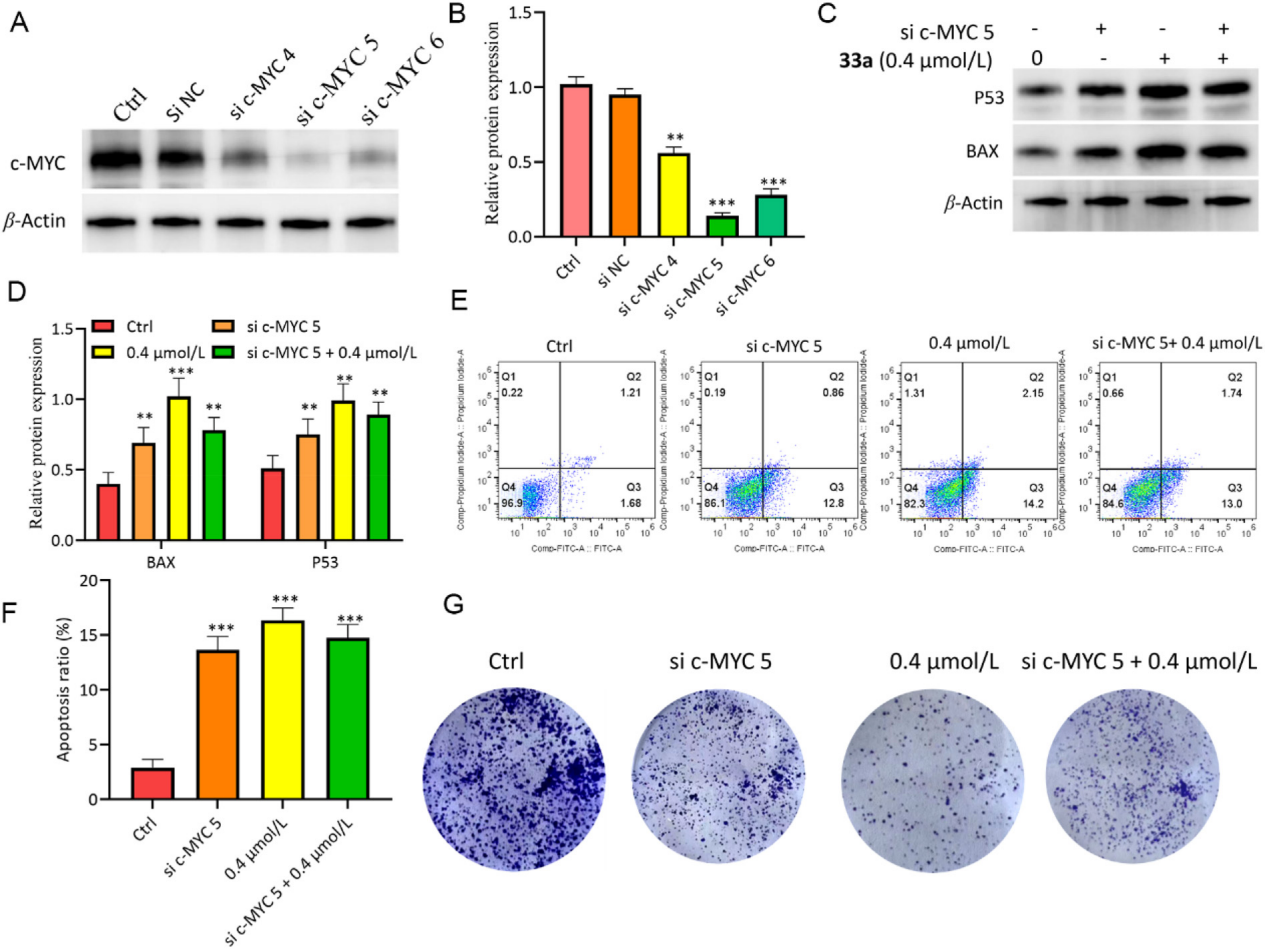
Induction of apoptosis and inhibition of autophagy by knockdown of *c-MYC*. (A,B) Western blot analysis of the expression levels of c-MYC by knockdown of *c-MYC*. (C,D) Western blot analysis of the expression levels of BAX and P53 by knockdown of *c-MYC*. (E,F) Apoptotic in HCT116 cells increased by knockdown of *c-MYC*. (G) The number of colonies decreased by the knockdown of *c-MYC*. Data are mean ± SD (*n* = 3), ∗*P* < 0.05, ∗∗*P* < 0.01, and ∗∗∗*P* < 0.001 compared with the control group.

Dysregulated *c-MYC* expression is strongly implicated in tumorigenesis^32^, so silencing of *c-MYC* is used to test the effect of c-MYC on the proliferation, and apoptosis of HCT116 cells exposed to **33a**. As shown in Fig. 14, the knockdown of *c-MYC* significantly upregulated BAX and P53, induced apoptosis, and decreased the number of colonies, which were not changed with **33a**-treatment after the knockdown of *c-MYC*, suggesting that c-MYC may function as a key effector molecule in the anticancer effects of **33a**. Lower levels of c-MYC alleviated the apoptosis-inducing activity and antiproliferative effects of compound **33a** in HCT116 cells. Taken together, these data indicate that compound **33a** exerts its antitumor activity primarily by inhibiting the c-MYC signaling pathway.

### 2.12 Pharmacokinetic profiles of compounds **33a** and **36b**

Pharmacokinetic (PK) characteristics of compounds **33a** and **36b** were analyzed after intravenous injection (1 mg/kg) and intragastric administration (2 mg/kg) in Sprague–Dawley rats. Plasma concentrations of **33a** and **36b** reached maxima at 4 h (*T*_max_) with maximum plasma concentrations (*C*_max_) of 301.2 ng/mL and 276.3 ng/mL, and half-lives (*T*_1/2_) of 6.8 h and 4.1 h in the i.g. group, respectively (Table 7 and Supporting Information Table S5). **33a** presented a better pharmacokinetic profile than **36b**, compound **33a** was selected for the following *in vivo* experiments.

**Table 7:** Analysis of main pharmacokinetic parameters.

Subject	*T*_1/2_ (h)	*T*_max_ (h)	*C*_max_ (ng/mL)	AUC_0-∞_ (ng/h/L)	CL (mL/min/kg)	Vss (mL/kg)
**33a** (IV)^a^	5.0	**-**	–	3273.0	5.1	2146.42
**33a** (Po)^b^	6.8	4	301.2	3314.2	–	–

‒, not applicable.

a Dosed intravenously at 1 mg/kg.

b Dosed orally at 2 mg/kg.

### 2.13 Acute toxicity of compound **33a**

The safety of compound **33a** in SPF BALB/c mice was evaluated by acute toxicity assay. As shown in Table 8, the LD_50_ of compound **33a** was calculated as 553.4 mg/kg, which was higher than that of 5-FU (230 mg/kg) ^33^. Considering the safety of the drug, 1/10 and 1/30 of the LD_50_ value (55 mg/kg and 20 mg/kg) were applied in subsequent *in vivo* experiments.

**Table 8:** Toxicity results and LD_50_ of compound **33a**.

Group (mg/kg)	Gender	Number of mice	Number of dead mice	Death rate (%)	LD_50_ (mg/kg)
90	♀	5	0	0	553.4
	♂	5	0		
260	♀	5	0	0	
	♂	5	0		
350	♀	5	1	10	
	♂	5	0		
450	♀	5	2	20	
	♂	5	0		
600	♀	5	3	60	
	♂	5	3		
800	♀	5	5	100	
	♂	5	5		

### 2.14 Inhibitory effect of compound **33a** on the growth of xenograft in mice

**Figure 15:**
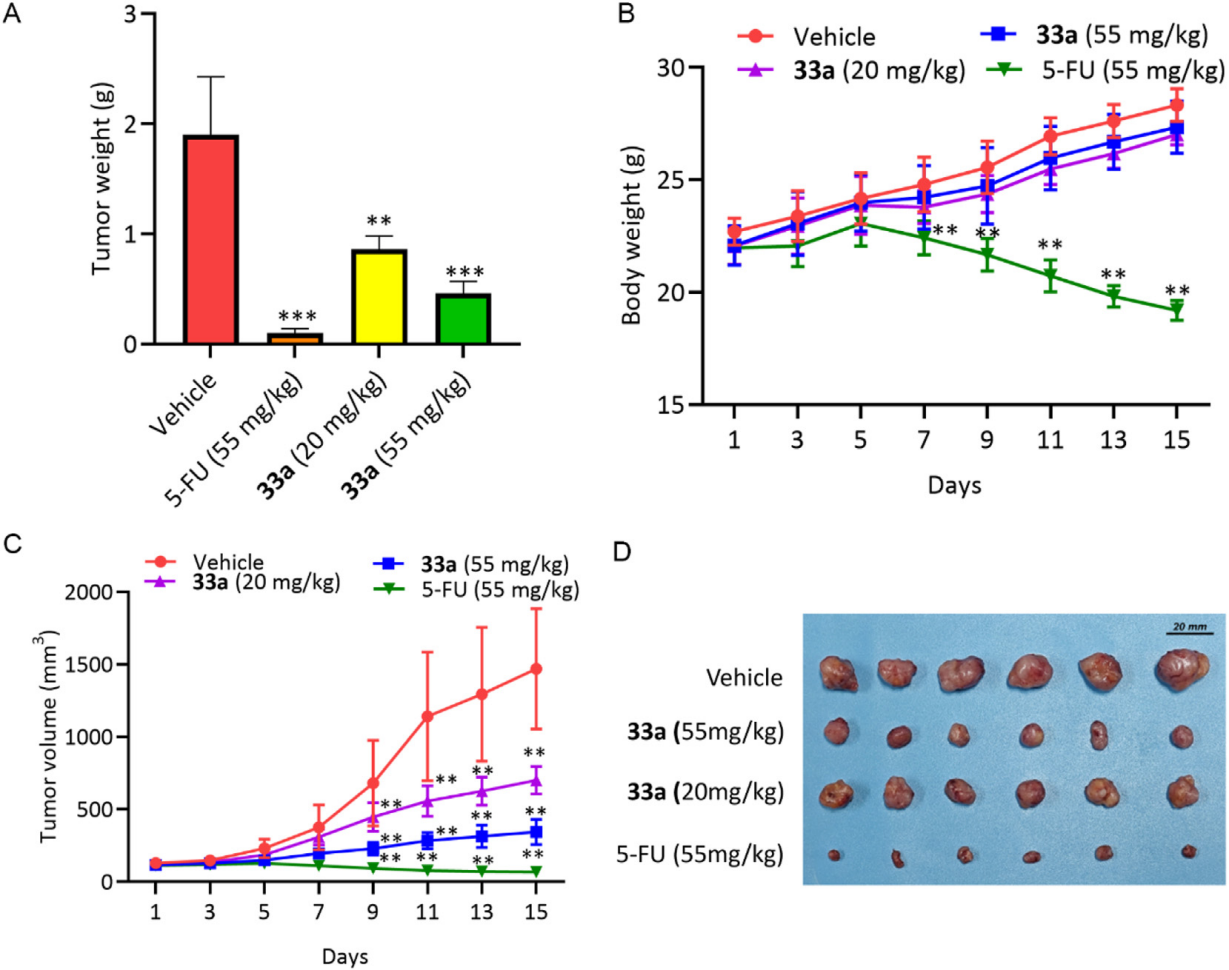
Inhibitory effect on the growth of xenograft in mice. (A) Tumor weight. (B) Body weight. (C) Tumor volume. (D) Tumor gross. Data are mean ± SD (*n* = 3), **∗∗***P* < 0.01, and **∗∗∗***P* < 0.001 compared with the Vehicle group.

To further evaluate the anticancer activity of compound **33a** *in vivo*, a mouse xenograft model of CT26 colon cancer was established. Six BALB/c mice per group were injected intraperitoneally with compound **33a** (at dosages of 55 mg/kg and 20 mg/kg), positive control 5-FU (55 mg/kg), or vehicle, once a day for 14 days. Compound **33a** had a significant dose-dependent inhibitory effect on tumor growth, both in terms of tumor volume and weight, with TGI values of 75.79% and 54.74%, respectively. Compound **33a** did not show an obvious toxic effect on body weight, indicating the safety of **33a** (Fig. 15A‒D).

**Figure 16:**
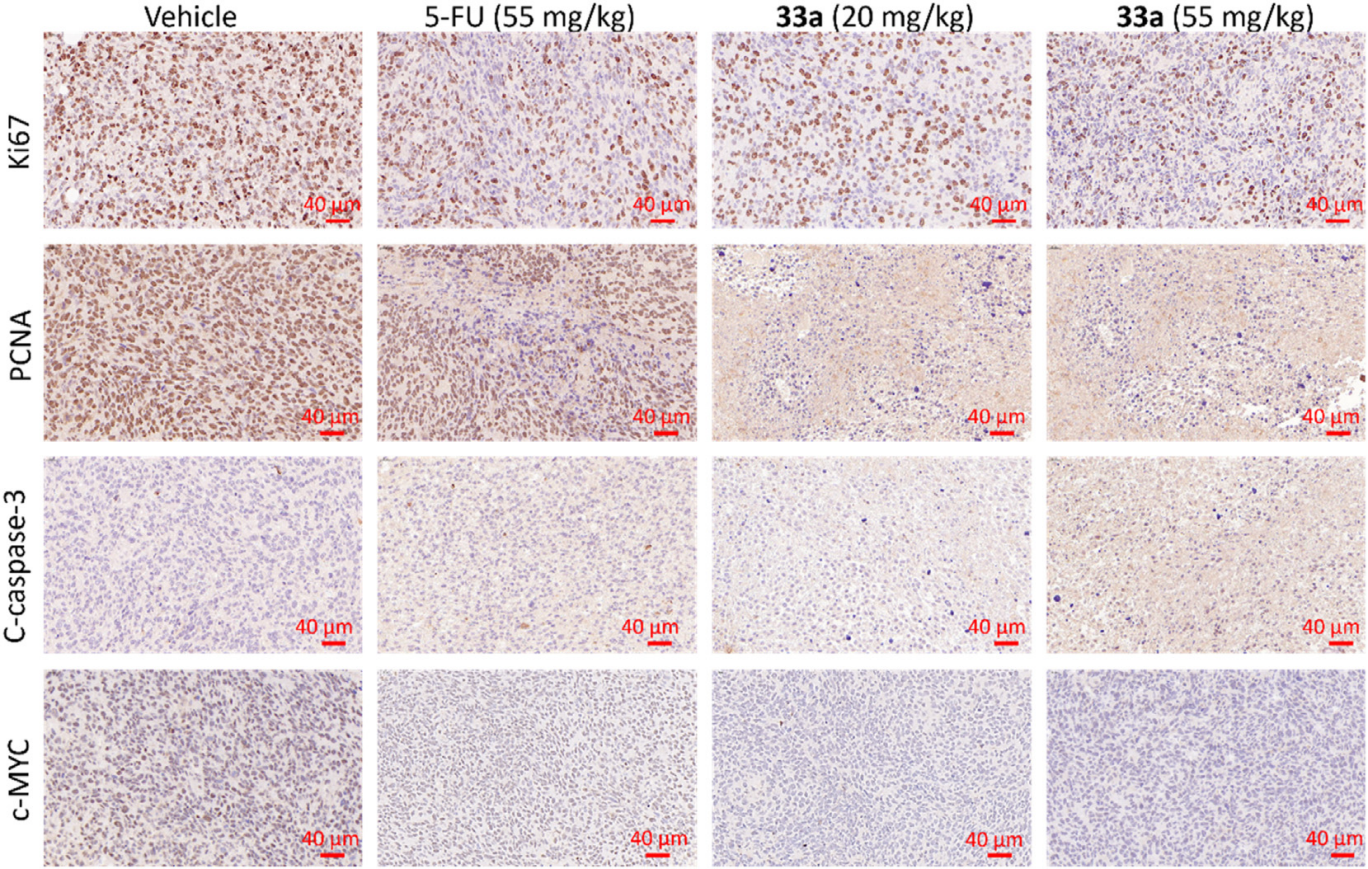
Immunohistochemical (IHC) analysis of the tumor tissue. Scale bar = 40 μm.

Subsequently, the tumor growth inhibition mechanism in mice was analyzed by immunohistochemical (IHC) analysis. Compound **33a** at dosages of 55 and 20 mg/kg could significantly reduce the expression of tumor proliferation markers Ki67 and PCNA, increase the expression of cleaved caspase-3, and decrease the expression of c-MYC, indicating inhibition of tumor growth and apoptosis occurred in the tumor tissue (Fig. 16).

**Figure 17:**
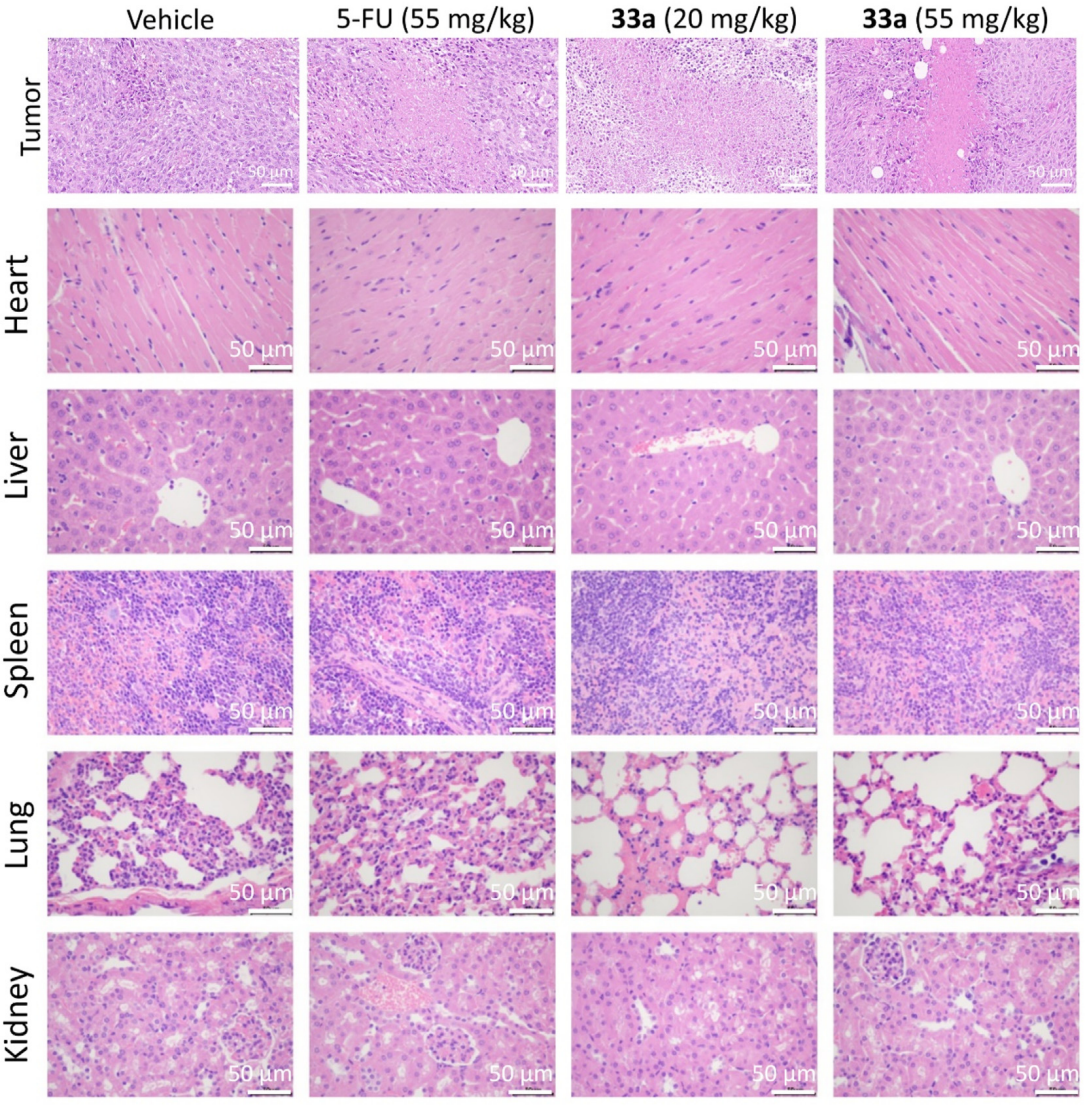
H&E staining of the tumor, heart, liver, spleen, lung, and kidney. Scale bar = 50 μm.

H&E staining of the tumor tissue revealed more necrosis regions in compound **33a**-treated mice. However, despite the observed changes in the tumors, the major organs showed no apparent evidence of injury or abnormality. This suggested that, at least on a macroscopic level, the treatment did not cause harm to these organs (Fig. 17). All these *in vivo* experiments indicated that compound **33a** exhibited potent antitumor efficacy and an acceptable therapy window for colon cancer.

### 2.15 Tissue distribution and metabolism of **33a***in vivo*

The BALB/c mice were injected intraperitoneally with compound **33a** at a dosage of 55 mg/kg, and then LC‒MS/MS was utilized to analyze the tissue distribution of **33a** in various tissues after 4 h. As depicted in Supporting Information Fig. S15, the measured concentrations were found to be 16.86 ± 2.99 ng/mg in heart, 12.47 ± 2.13 ng/mg in liver, 5.49 ± 2.45 ng/mg in spleen, 38.48 ± 4.43 ng/mg in lung, 70.4 ± 5.14 ng/mg in kidney, and 87.87 ± 21.52 ng/mg in brain, indicating that **33a** can readily cross the blood‒brain barrier and enter the brain.

The *in vivo* metabolites of compound **33a** were also analyzed. BALB/c mice were administered compound **33a** intraperitoneally at a dosage of 55 mg/kg, while a control group received an equivalent volume of solvent. Blood samples were collected from the mice in each group at various time points post-administration: 0, 0.25, 0.5, 1, 2, 4, 8, 12, and 24 h. Subsequently, the mixed plasma samples were analyzed using LC‒QTOF/MS to identify and characterize the serum metabolites of the compound. Based on the mass-to-charge ratio (*m*/*z*), major metabolites were identified as: **33a-M1** (*m*/*z* 571.1748, glucuronidation), **33a-M2** (*m*/*z* 367.1114, from ester hydrolysis), **33a-M3** (*m*/*z* 543.1435, ester hydrolysis and glucuronidation). The metabolic pathways for these major metabolites were confirmed to be hydrolysis of carboxylic acid ester and glucuronidation (Supporting Information Fig. S16).

## 3 Conclusions

A series of novel thioheterocyclic nucleosides were designed and synthesized, and their antiproliferative activities were evaluated against cancer cell lines. SAR studies showed that most of the compounds exhibited significant antitumor activity *in vitro*. Among them, compounds **33a** and **36b** showed potent anti-proliferation activities with IC_50_ values of 0.27 and 0.49 μmol/L in HCT116 cells, respectively. Compounds **33a** and **36b** significantly arrested the cell cycle in the G_2_/M phase and induced apoptosis of HCT116 cells. Further studies indicated that **33a** and **36b** could inhibit migration and invasion of HCT116 cells, increase ROS levels, induce DNA damage, ER stress, and mitochondrial damage, and inhibit autophagy. Biological information analysis, RNA-sequencing, GSEA analyses, DARTS assay, CETSA, and SPR experiments identified that **33a** and **36b** showed antitumor activity by suppressing the c-MYC pathway. *In vivo* studies showed that compound **33a** had good PK profiles and low toxicity (LD_50_ value of 553.4 mg/kg). Importantly, **33a** inhibited tumor growth and induced tumor necrosis without obviously affecting the body weight of the mice. Overall, this work identified a series of novel thioheterocyclic nucleoside derivatives as promising antitumor candidates by suppressing the c-MYC pathway, which lays a foundation to further explore the potential of thioheterocyclic nucleosides for anticancer therapy.

## 4 Experimental

### 4.1 Reagents and antibodies

All compounds were dissolved in dimethyl sulfoxide (DMSO, Sigma–Aldrich, St. Louis, MO, USA) at a concentration of 100 mM, and stored at 4 °C. Cell counting kits were obtained from Beijing Lanjie Ke Technology Co., Ltd (Beijing, China). McCoy's 5A medium, Dulbecco's modified Eagle medium (DMEM), RPMI-1640 medium, phosphate buffer (PBS), and fetal bovine serum (FBS) were purchased from Wuhan Servicebio Technology Co., Ltd. (Wuhan, China). Polyvinylidene fluoride (PVDF) membrane was purchased from Merk Millipore Ltd(Ireland). CDK1, Cyclin B1, Caspase-3, BAX, BCL-2, Cleaved-PARP, *γ*-H2AX, P53, ATF4, CHOP, PERK, ATP6, LC3, Beclin 1, P62, *β*-Catenin, c-MYC, GAPDH, *β*-actin, and *β*-Tubulin primary antibodies were purchased from Proteintech Group, Inc (Wuhan, China). Cell cycle assay kit (PI/RNase Staining) and cell apoptosis assay kit (Annexin V-FITC/PI) were purchased from BD Pharmingen^Tm^ (Santiago, USA). 5-Ethynyl-2′-deoxyuridine (EdU) kit was purchased from RiboBio Co., Ltd. (Guangzhou, China). Acridine orange stain (AO), ER-Tracker Red dye, Fluo 4-AM dye, Mito-Tracker Green, 2,7-dichlorodihydrofluorescein diacetate (DCFH-DA), JC-1 staining kit, and BCA protein assay kit were obtained from Beyotime Biological Technology (Shanghai, China). Active c-MYC protein was purchased from Abcam (Aibo Kang (Shanghai) Trading Co., Ltd.). Hc-MYC-435 (si C-MYC-1), Hc-MYC-1464 (si C-MYC-2), Hc-MYC-1484 (si C-MYC-3), FAM NC (si NC), and RNA TransMate were purchased from Sangon Biotech (Shanghai) Co., Ltd. (Shanghai, China).

### 4.2 General chemistry methods

All reagents were purchased from commercial suppliers and used without further purification. ^1^H and ^13^C NMR spectral data were recorded in CDCl_3_, CD_3_OD, or DMSO-*d*_6_ with a Bruker 400 MHz or 600 MHz spectrometer using tetramethylsilane (TMS) as the internal standard. All products were characterized by high-resolution mass spectrometry (HRMS) recorded on an ABI/Sciex QStar mass spectrometer (AB SCIEX, Boston, USA). Chiral resolution of compounds **33a** and **36b** were conducted *via* HPLC columns (Daicel Chiral Technologies (China) Co., LTD.). Chiral HPLC analysis was performed with Agilent Technologies 1260 Infinity apparatus (Santa Clara, USA). Targeted compounds were analyzed by Thermo Scientific UltiMate 3000 high-performance liquid chromatography with a UV/visible detector and an Agilent C_18_ column (5 μmol/L, 4.6 mm × 150 mm). Due to the limited space of the manuscript, general procedures and NMR data of the corresponding intermediates and all final products have been moved into the Supporting Information

### 4.3 CCK-8 cell viability assay

MCF-7, A375, and HeLa cells were cultured in DMEM, HCT116 cells were cultured in McCoy's 5A medium, and DU145 and CT26 were cultured in RPMI 1640 with 10% FBS, 1% penicillin-streptomycin solution (10,000 U/mL penicillin and 10,000 μg/mL streptomycin) at 37 °C under 5% CO_2_. The cells were seeded in 96-well plates (4 × 10^3^ cells/well) overnight and then treated with various concentrations (1–50 μmol/L) of the compound. After 48 h, the cell viability was measured by CCK-8 assay. The absorbance of each well was measured using a microplate reader (Multiskan FC, Thermo Fisher Scientific, Massachusetts, USA) at 450 nm wavelength. Assays were performed in triplicate. Compound concentrations that inhibited cell growth by 50% (IC_50_) were calculated from the dose–response curves of each cell line. Data are mean ± SD values from three independent experiments.

### 4.4 Colony formation

HCT116 cells were seeded in 6-well plates (1.5 × 10^3^ cells/well). Various concentrations of compound **33a** or **36b** were added to the wells for 48 h. The culture medium was changed every 72 h, at the end of 14 days, cells were fixed with methanol for 30 min and stained with 0.1% crystal violet staining solution for 30 min at room temperature, then washed three times in PBS and allowed to dry. Photographs were then taken.

### 4.5 EdU assay

HCT116 cells were seeded in 96-well plates (4 × 10^4^ cells/well) overnight and then the cells were treated with various concentrations of compound **33a** or **36b**. After 48 h, the experiment operation process was performed according to the manufacturer's instructions. Images were obtained with a Leica DMIL fluorescence microscope (Leica Microsystems, Wetzlar, Germany).

### 4.6 Wound healing

HCT116 cells were seeded in 6-well plates (6 × 10^5^ cells/well) to full confluence and then an artificial wound was created in the cell monolayer using a 200 μl pipette tip. Images of wound closure were captured at 0 and 24 h using a Leica DMIL fluorescence microscope (Leica Microsystems). The percentage of wound closure was calculated using Image J software.

### 4.7 Invasion assay

A transwell chamber (24-well) (Corning, New York, USA) was coated with Matrigel (BD Biosciences, New Jersey, USA), and 1 × 10^5^ cells per well were seeded in the upper chamber in serum-free medium and treated with defined doses of compound **33a** or **36b**. The complete medium was placed in the lower chamber. After 24 h, the cells penetrating through the pores were stained with 0.1% crystal violet solution and observed with a Leica DMIL fluorescence microscope (Leica Microsystems).

### 4.8 Cell cycle assay

Cells were seeded in 6-well plates (5 × 10^5^ cells/well). The following day, defined concentrations of compound **33a** or **36b** were added to the wells for 48 h at 37 °C with 5% CO_2_. The cells were collected and fixed in ice-cold 70% alcohol overnight. Then the cells were stained in propidium iodide (PI) dye for 15 min in the dark and the cell DNA contents were analyzed *via* flow cytometry (Becton, Dickinson Company, New Jersey, USA).

### 4.9 Cell apoptosis

HCT116 cells were seeded in 6-well plates (4 × 10^5^ cells/well). The following day, defined concentrations of compound **33a** or **36b** were added to the wells for 48 h at 37 °C with 5% CO_2_. The cells were collected and stained with Annexin V-FITC and PI for 15 min at room temperature. Apoptotic cells were analyzed with flow cytometry (BD Bioscience).

### 4.10 Intracellular ROS measurement and AO stain

HCT116 cells were seeded in 96-well plates overnight and then the cells were treated with defined concentrations of compound **33a** or **36b**. In addition, cells were pretreated with 0.8 mmol/L NAC for 1 h. After 48 h, the cells were washed with PBS. DCFH-DA (100 μL, 100 μmol/L) was added to each well and incubated for 30 min at 37 °C, and the cells were washed twice with PBS. Finally, the cells were cultivated in a 100 μL culture medium and the fluorescence signals were detected with an EnVision® NexusTM Multi-Mode Microplate Reader or flow cytometry (Becton, Dickinson Company).

HCT116 cells were seeded in 6-well plates (2 × 10^5^ cells/well). The following day, defined concentrations of compound **33a** or **36b** were added to the wells for 48 h at 37 °C with 5% CO_2_. The cells were collected and stained with acridine orange stain for 20 min in the dark and were observed with a Leica DMIL fluorescence microscope (Leica Microsystems).

### 4.11 ER measurement, intracellular Ca^2+^, MitoTracker green-stained mitochondria, and determination of mitochondrial membrane potential

HCT116 cells were seeded in 6-well plates (2 × 10^5^ cells/well). The following day, defined concentrations of compound **33a** or **36b** were added to the wells for 48 h at 37 °C with 5% CO_2_. In addition, cells were pretreated with 0.8 mM NAC for 1 h. The ER organelle was stained with ER-Tracker Red (Beyotime) following the instructions and observed with a Leica DMIL fluorescence microscope (Leica Microsystems), and intracellular Ca^2+^ was measured with the Fluo 4-AM dye, The fluorescence intensity of Fluo 4-AM was determined by flow cytometry.

Mitochondrial damage was imaged by Mito-Tracker Green (Beyotime) or cell-permeable JC-1 dye following the instructions and observed with a Leica DMIL fluorescence microscope (Leica Microsystems) or flow cytometry (Becton, Dickinson Company).

### 4.12 Construction of colorectal cancer-related targets

Colorectal cancer-related targets were obtained by searching for keywords “colon cancer” in Disgenet (https://www.disgenet.org/) (score ≥0.01), OMIM (https://omim.org/), Therapeutic Target Database (TTD) (https://db.idrblab.net/ttd/), and GeneCards (https://www. genecards.org/) online database (score ≥24).

### 4.13 Evaluation of the hub genes

Colorectal cancer-related targets were selected with the Venny 2.1.0 tool, then uploaded to the STRING (https://cn.string-db.org/) online database, and degree value was constructed with Cytoscape 3.8.2 software. Genes with the top 30-degree values were recognized as hub genes.

### 4.14 Protein–protein interaction network (PPI) and gene ontology (GO) analyses

Hub genes were submitted to Cytoscape software to construct PPI and analyzed by GO enrichment analyses and KEGG enrichment analyses using the Metascape online database (https://metascape.org/gp/index.html#/main/step1).

### 4.15 RNA-sequencing analysis

Total RNA was isolated from the HCT11 cells using Trizol (Invitrogen Life Technologies Corporation, Carlsbad, USA), cDNA synthesis followed by adaptor ligation and enrichment according to the instructions. The purified library products were evaluated using Illumina NovaSeq 6000 (paired-end 150 bp).

### 4.16 Drug affinity responsive target stability (DARTS) assay

HCT116 cells were lysed with mammalian protein extraction reagent (M-PER), centrifuged, and quantified by BCA protein assay. Lysates were incubated with various concentrations (0, 20, 40, 80, 160, 320, and 640 μmol/L) of compound **33a** or **36b** or DMSO for 30 min. Proteolysis was performed by adding pronase solution at a ratio of 1:3200 for 30 min at room temperature and then stopped by adding SDS-PAGE sample loading buffer and boiling at 100 °C for 5 min. c-MYC protein was analyzed by Western blot.

### 4.17 Cellular thermal shift assay (CETSA)

In short, the lysates were incubated with **33a** or **36b** or DMSO for 30 min. The pyrolysis products were divided equally and individually heated at different temperatures (40–70 °C) for 3 min and then cooled on ice. After freeze-thawing twice with liquid nitrogen, the supernatant was centrifuged, collected, and boiled in a loading buffer. c-MYC protein was analyzed by Western blot.

### 4.18 Surface plasmon resonance (SPR) assay

SPR analyses to evaluate the interaction of thioheterocyclic nucleoside derivatives with c-MYC immobilized onto a CM5 sensor chip (Cytiva, BR100012, Sweden) were performed using a Biacore T200 apparatus (Cytiva, Sweden) at 25 °C. During the analyte–ligand interaction, the change of the refractive index was measured in real-time which allowed interaction results to be plotted as response units (RU) *versus* time.

### 4.19 Western blotting analysis

Whole protein was extracted and separated by polyacrylamide gel electrophoresis (SDS-PAGE) and transferred onto nitrocellulose membranes. Membranes were blotted with CDK1, Cyclin B1, Caspase-3, BAX, BCL-2, PARP-1, *γ*-H2AX, P53, ATF4, CHOP, PERK, ATP6, LC3, Beclin1, P62, *β*-Catenin, c-MYC, MAX, UPP1, GAPDH, *β*-Actin, and *β*-Tubulin primary antibodies. Protein band analyses were performed with ImageJ software and protein expression levels were presented as a percentage difference compared to control.

### 4.20 Molecular docking

PPIA (PDB: 1NKP) was obtained from the Protein Data Bank (PDB). Molecular docking was performed using Autodock Vina 1.1.2 software (The Scripps Research Institute, San Diego, USA). Discovery Studio client software was used to visually analyze the results.

### 4.21 siRNA interference

siRNA interference was performed using a Transfection Kit (Sangon Biotech (Shanghai) Co., Ltd., Shanghai, China) according to the manufacturer's instructions. Western blot analysis, colony assay, and cell apoptosis under the same conditions described above.

### 4.22 Pharmacokinetic analysis

All animal studies and procedures were approved by the Animal Ethics Committee of College of Chemistry and Chemical Engineering, Henan Normal University, China. Male Sprague–Dawley rats (220 ± 10 g, Shanghai Yuanxin Biological Co., Ltd., Shanghai, China) were used to evaluate drug pharmacokinetic properties. Briefly, compound **33a** or **36b** was dissolved in PBS containing 10% DMSO and 20% castor oil. Rats were treated with 1 mg/kg of compound **33a** or **36b** for intravenous injection and 2 mg/kg for oral intragastric administration. Blood samples were collected from each rat at 0, 0.083, 0.25, 0.5, 1, 2, 4, 6, 8, 12, and 24 h. Plasma was isolated from the blood samples by centrifugation (1200 rpm, 4 °C, 10 min. ThermoFisher Scientific, Thermo Electron LED GmbH, Zweigniederlassung Osterode Am Kalkberg, Osterode am Harz, Germany). Sample concentrations were determined by LC-MS/MS (API 4000, AB SCIEX) based on a standard curve. All data were calculated by Excel software.

### 4.23 Acute toxicity assay

SPF BALB/c mice were divided into 8 groups randomly (*n* = 10, 5 male, and 5 female) and injected intraperitoneally with defined dosages of compound **33a** (90, 260, 350, 450, 600, and 800 mg/kg) or dilute (DMSO: castor oil: PBS = 1:2:7, *v*/*v*/*v*). The mice were observed continuously for 14 days after administration. The number of mortalities during the experiment was recorded, and the statistical result of the experimental endpoint was used to calculate LD_50_.

### 4.24 Xenograft tumor growth assay

All animal studies and procedures were approved by the Animal Ethics Committee of College of Chemistry and Chemical Engineering, Henan Normal University, China. CT26 cells (ATCC, Rockville, MD, USA) were used for tumor allograft experiments. SPF BALB/c mice (male, 22 ± 2 g, Changzhou Cavens Laboratory Animal Co. Ltd., Changzhou, China) were randomly divided into four experimental groups (*n* = 6): vehicle, positive control 5-FU (55 mg/kg), and compound **33a** (20 mg/kg and 55 mg/kg). Mice were injected with CT26 cells (1 × 10^7^ per mouse) subcutaneously. The diameter of transplanted mouse tumors was measured with vernier calipers. After the tumor grew to about 120 mm^3^, the mice were injected intraperitoneally with compound **33a** (55 mg/kg or 20 mg/kg), 5-FU (55 mg/kg), or vehicle solvent control (0.2 mL) daily for 14 days. The antitumor activity of the tested compounds was evaluated by measuring the tumor diameter. Tumor diameter and body weight were measured every two days. After 15 days, tumor tissues were surgically removed, weighed, and photographed. The tumor volume (TV) was calculated as in Eq. (1):

(1) $$V=1/2\times a\times b^{2}$$

where *a* and *b* represent the length and width, respectively. Samples were used for histology. The tumor growth inhibition rate TGI (%) was calculated with Eq. (2):

(2) $$\text{TGI}\;\left(\%\right)=\left(1‒T_{w}/C_{w}\right)\times 100$$

where *T*_w_ and *C*_w_ are the average tumor weight on the 15th day for the treatment group and the model group, respectively.

### 4.25 Tissue distribution of **33a***in vivo*

Three SPF BALB/c mice were adaptively fed for one week, and subsequently, 55 mg/kg of **33a** was administered intravenously to each mouse (*n* = 3). 4 h post-administration, the mice were euthanized, and samples of blood, heart, liver, spleen, lung, kidney, and brain were collected. The plasma and drug concentrations in these various tissues were then analyzed using LC-MS/MS, and the drug content in each tissue was calculated based on its respective weight.

#### 4.25.1 Chromatographic conditions

The chromatography was performed using a WATERS ACQUITY UPLC BEH C18 liquid chromatography column (50 mm × 2.1 mm, with an internal diameter of 1.7 μm), maintained at a constant temperature of 40 °C. The mobile phase comprised two components: mobile phase A, consisting of ultrapure water containing 0.1% formic acid, and mobile phase B, which was acetonitrile. The elution gradient was programmed as follows: initially at 0–1 min, A: B (80:20); followed by a linear increase to A: B (10:90) from 1 to 5 min; then maintaining A: B (10:90) until 9 min, with a brief reversion to A: B (80:20) at 9.1 min, lasting for a short duration until 9.1 min; subsequently, the ratio was maintained at A: B (80:20) from 9.1 to 11.5 min. The total runtime of the chromatography was 11.5 min, with a flow rate set at 0.30 mL/min. The retention time for **33a** was recorded as 5.81 min, while the retention time for the internal standard (IS) was 4.75 min.

#### 4.25.2 Mass spectrometry conditions

The measurements were performed utilizing LC–MS/MS equipped with an ESI (Electrospray Ionization) source, operating in positive ion mode. The heated capillary temperature was adjusted to 450 °C. The CAD (Collision Activated Dissociation) energy was set at 4, while the nebulizer gas (N_2_) flow rate, also known as the curtain gas, was maintained at 11. The GS1 (N_2_) and GS2 (N_2_) gas flows were both set to 40. For quantitative analysis, the scanning mode employed was Multiple Reaction Monitoring (MRM). The specific ion transitions monitored were *m*/*z* 396.20 → 222.200 for **33a** and *m*/*z* 360.20 → 244.20 for the internal standard (IS).

### 4.26 Metabolites of **33a** in mice

SPF BALB/c mice were adaptively fed for one week. In the control group, mice received an intravenous injection of an equal volume of solvent. In the Model group, mice received an intravenous injection of 55 mg/kg of **33a**. Following administration at time points of 0, 0.25, 0.5, 1, 2, 4, 8, 12, and 24 h, mixed plasma samples were collected from mice in each group. Subsequently, blood metabolites were detected, identified, and analyzed utilizing LC-QTOF/MS technology.

#### 4.26.1 Liquid chromatography conditions

Chromatographic column: Waters HSS T3 1.8 μm, 2.1 mm × 100 mm Column; Flow rate: 0.3 mL/min; Column oven temperature: 40 °C; Mobile phase: A: Aqueous phase (ultrapure water + 0.1% formic acid), B: Organic Phase (acetonitrile); Gradient as follows: Mobile phase B 5% (0.00–1.50 min), 5%–15% (1.50–2.50 min), 15%–60% (2.50–6.00 min), 60%–95% (6.00–10.00 min), 95% (10.00–12.00 min), 95%–5% (12.00–12.50 min), 5% (12.50–15.50 min).

#### 4.26.2 Mass spectrometry conditions

The ionization mode is Electrospray Ionization in positive mode (ESI+), with an ion source voltage of 5 kV and a temperature set at 550 °C. The collision energy (CE) is 30 eV. The nebulizing gas, gas1 (nebulizer gas), and gas2 (auxiliary gas) are all nitrogen (N_2_) with their pressures set to 55 psi. The curtain gas (CUR) pressure is 35 psi. The parent ion scan range for the first-order mass spectrum is *m*/*z* 3100 to 1000, and the daughter ion scan range is *m*/*z* 50 to 1000. Dynamic Background Subtraction (DBS) is activated. The software used for data acquisition is Analyst® TF 1.8 software.

### 4.27 Statistical analysis

Statistical analyses were determined by *t*-test with SPSS23.0 software, and a *P*-value <0.05 was considered statistically significant. All results were represented as means ± standard deviation (SD). All experiments were repeated at least three times.

## Author contributions

Xian-Jia Li: Writing – review & editing, Writing – original draft, Validation, Methodology, Investigation, Formal analysis, Data curation. Ke-Xin Huang: Data curation. Ke-Xin Wang: Methodology, Formal analysis. Ru Liu: Investigation. Dong-Chao Wang: Data curation. Yu-Ru Liang: Writing – review & editing, Supervision. Er-Jun Hao: Supervision. Yang Wang: Writing – review & editing, Supervision. Hai-Ming Guo: Writing – review & editing, Supervision, Methodology, Investigation.

## Conflicts of interest

The authors have no conflicts of interest to declare.

## Appendix A Supplementary data

The following is the Supporting Information to this article:

## References

[1] Qu S., Mulamoottil V.A., Nayak A., Ryu S., Hou X., Song J. (2016). Design, synthesis, and anticancer activity of C8-substituted-4′-thionucleosides as potential HSP90 inhibitors. Bioorg Med Chem.

[2] Jin Y.P., Qiu J.P., Lu X.F., Li G.W. (2022). c-MYC Inhibited ferroptosis and promoted immune evasion in ovarian cancer cells through NCOA4 mediated ferritin autophagy. Cells.

[3] Khalid K.M., Ratnayake W.S., Apostolatos C.A., Acevedo-Duncan M. (2024). Dual inhibition of atypical PKC signaling and PI3K/Akt signaling dysregulates c-Myc to induce apoptosis in clear cell Renal Cell Carcinoma. Front Oncol.

[4] Ni T.Y., Chu Z.W., Tao L., Zhao Y., Zhu M., Luo Y.Y. (2023). PTBP1 drives c-Myc-dependent gastric cancer progression and stemness. Br J Cancer.

[5] Liang D.L., Yu Y.F., Ma Z.H. (2020). Novel strategies targeting bromodomain-containing protein 4 (BRD4) for cancer drug discovery. Eur J Med Chem.

[6] Liang H.Z., Du J.T., Elhassan R.M., Hou X.B., Fang H. (2021). Recent progress in development of cyclin-dependent kinase 7 inhibitors for cancer therapy. Expet Opin Invest Drugs.

[7] Boffo S., Damato A., Alfano L., Giordano A. (2018). CDK9 inhibitors in acute myeloid leukemia. J Exp Clin Cancer Res.

[8] Medina J.R., Tian X.R., Li W.H., Suarez D., Mack J.F., LaFrance L. (2021). Cell-based drug discovery: identification and optimization of small molecules that reduce c-MYC protein levels in cells. J Med Chem.

[9] Miller L.H., Maxa K.L., Winter S.S., Gossai N.P. (2023). The role of nelarabine in the treatment of T-cell acute lymphoblastic leukemia/lymphoma: challenges, opportunities, and future directions. Expert Rev Anticancer Ther.

[10] Cui Y., Mi R., Chen L., Wang L., Li D.B., Wei X.D. (2024). Case report: Venetoclax plus Azacitidine in treatment of acute undifferentiated leukemia. Hematology.

[11] Ikeda T., Sato K., Kawaguchi S., Izawa J., Takayama N., Hayakawa H. (2024). Forodesine enhances immune responses through Guanosine-mediated TLR7 activation while preventing graft-*versus*-host disease. J Immunol.

[12] Schofield R.C., Scordo M., Shah G., Carlow D.C. (2024). Quantification of clofarabine and fludarabine in plasma by high-performance liquid chromatography-tandem mass spectrometry. Methods Mol Biol.

[13] Creelan B.C., Wang C., Teer J.K., Toloza E.M., Yao J.Q., Kim S. (2021). Tumor-infiltrating lymphocyte treatment for anti-PD-1-resistant metastatic lung cancer: a phase 1 trial. Nat Med.

[14] Zhang B., Tang C., Yao Y., Chen X.H., Zhou C., Wei Z.T. (2021). The tumor therapy landscape of synthetic lethality. Nat Commun.

[15] Perlíková P., Hocek M. (2017). Pyrrolo[2,3- *d*]pyrimidine (7-deazapurine) as a privileged scaffold in design of antitumor and antiviral nucleosides. Med Res Rev.

[16] Fernandez-Bolaños J.G., al-Masoudi N.A., Maya I. (2001). Sugar derivatives having sulfur in the ring. Adv Carbohydr Chem Biochem.

[17] De Souza D., Mariano D.O.C., Nedel F., Schultze E., Campos V.F., Seixas F. (2015). New organochalcogen multitarget drug: synthesis and antioxidant and antitumoral activities of chalcogenozidovudine derivatives. J Med Chem.

[18] Huang K.X., Xie M.S., Zhang Q.Y., Qu G.R., Guo H.M. (2018). Enantioselective synthesis of carbocyclic nucleosides *via* asymmetric [3 + 2] annulation of α-purine-substituted acrylates with MBH carbonates. Org Lett.

[19] Huang K.X., Xie M.S., Sang J.W., Qu G.R., Guo H.M. (2021). Asymmetric synthesis of 3-amine-tetrahydrothiophenes with a quaternary stereocenter *via* Nickel(II)/Trisoxazoline-catalyzed sulfa-Michael/Aldol cascade reaction: divergent access to chiral thionucleosides. Org Lett.

[20] Gu J., Sui Z., Fang C.S., Tan Q.Y. (2017). Stereochemical considerations in pharmacokinetic processes of representative antineoplastic agents. Drug Metab Rev.

[21] Yu Strobykina I., Voloshina A.D., Andreeva O.V., Sapunova A.S., Lyubina A.P., Amerhanova S.K. (2021). Synthesis, antimicrobial activity and cytotoxicity of triphenylphosphonium (TPP) conjugates of 1,2,3-triazolyl nucleoside analogues. Bioorg Chem.

[22] Zhang K.H., Zou Y.J., Shan M.M., Pan Z.N., Ju J.Q., Liu J.C. (2024). Arf1 GTPase regulates Golgi-dependent G2/M transition and spindle organization in oocyte meiosis. Adv Sci.

[23] Wang M.Q., Li L.Y., Yang S.P., Guo F.Y., Zhu G.M., Zhu B. (2023). Synthesis of novel oxazol-5-one derivatives containing chiral trifluoromethyl and isoxazole moieties as potent antitumor agents and the mechanism investigation. Bioorg Chem.

[24] Ly L.D., Xu S., Choi S.K., Ha C.M., Thoudam T., Cha S.K. (2017). Oxidative stress and calcium dysregulation by palmitate in type 2 diabetes. Exp Mol Med.

[25] D'Arcy M.S. (2019). Cell death: a review of the major forms of apoptosis, necrosis and autophagy. Cell Biol Int.

[26] Prerna K., Dubey V.K. (2022). Beclin1-mediated interplay between autophagy and apoptosis: New understanding. Int J Biol Macromol.

[27] Maiuri M.C., Zalckvar E., Kimchi A., Kroemer G. (2007). Self-eating and self-killing: crosstalk between autophagy and apoptosis. Nat Rev Mol Cell Biol.

[28] Mareninova O.A., Jia W.Z., Gretler S.R., Holthaus C.L., Thomas D.D.H., Pimienta M. (2020). Transgenic expression of GFP-LC3 perturbs autophagy in exocrine pancreas and acute pancreatitis responses in mice. Autophagy.

[29] Chen B., Xie K., Zhang J.Z., Yang L.T., Zhou H.S., Zhang L.Y. (2023). Comprehensive analysis of mitochondrial dysfunction and necroptosis in intracranial aneurysms from the perspective of predictive, preventative, and personalized medicine. Apoptosis.

[30] Stine Z.E., Walton Z.E., Altman B.J., Hsieh A.L., Dang C.V. (2015). MYC, Metabolism, and cancer. Cancer Discov.

[31] Okumura M., Iwakiri T., Yoshikawa N., Nagatomo T., Ayabe T., Tsuneyoshi I. (2022). Hepatocyte growth factor enhances antineoplastic effect of 5-fluorouracil by increasing UPP1 expression in HepG2 cells. Int J Mol Sci.

[32] Wu T.Y., Chen X.C., Tang G.X., Shao W., Li Z.C., Chen S.B. (2023). Development and characterization of benzoselenazole derivatives as potent and selective *c-MYC* transcription inhibitors. J Med Chem.

[33] https://go.drugbank.com/drugs/DB00544.

