# Supplementary material for: Design, synthesis, and antitumor activity of novel thioheterocyclic nucleoside derivatives by suppressing the c-MYC pathway
Source: Acta Pharm Sin B. 2025 May 19;15(7):3685–707. doi: 10.1016/j.apsb.2025.05.008 (PMC12278645; doi:10.1016/j.apsb.2025.05.008)
Supplement: Multimedia component 1 [file mmc1.pdf]

Supporting Information for

Original article

Design, Synthesis, and Antitumor Activity of Novel Thioheterocyclic Nucleoside Derivatives by Suppressing the c-MYC Pathway

Xian-Jia Li[a], Ke-Xin Huang[a,d], Ke-Xin Wang[a], Ru Liu[a], Dong-Chao Wang[a], Yu-Ru Liang[c,*], Er-Jun Hao[a,*], Yang Wang[b,*], Hai-Ming Guo[a,*]

[a]*State Key Laboratory of Antiviral Drugs, Pingyuan Laboratory, Key Laboratory of Green Chemical Media and Reactions, Ministry of Education, School of Chemistry and Chemical Engineering, Henan Normal University, Xinxiang 453007, China*

[b]*School of Pharmacy, Fudan University, Shanghai 201203, China*

[c]*Institute of Translation Medicine, Shanghai Jiao Tong University, Shanghai 200240, China*

[d]*School of Biological and Chemical Engineering, Nanyang Institute of Technology, Nanyang 473004, China*

*Corresponding authors. Tel.: +86 21 34206996 (Yu-Ru Liang); +86 373 3326335 (Er-Jun Hao); +86 21 51980115 (Yang Wang); +86 373 3329276 (Hai-Ming Guo).

E-mail addresses: sjtu13122370353@sjtu.edu.cn (Yu-Ru Liang), hej@htu.edu.cn (Er-Jun Hao), wangyang@shmu.edu.cn (Yang Wang), ghm@htu.edu.cn (Hai-Ming Guo).

Contents

1. Chemistry 3

1.1. General experimental procedures...3

1.2. ^{1}H NMR and ^{13}C NMR data 3

1.3. Copies of ^{1}H NMR, ^{13}C NMR and HR-ESIMS spectra of target compounds 28

1.4. HPLC chart for compounds **33a, 36b**...162

2. X-Ray Crystallographic Analysis of **33aa** (SS) (CCDC 2324828)..166

3. X-Ray Crystallographic Analysis of **36ba** (*SR*) (CCDC 2324827) 168

4. Biology..169

4.1 Supplemental Table and Figures 169

1. Chemistry

1.1. General experimental procedures

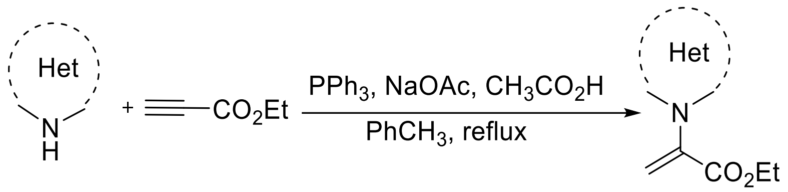

In a round bottom flask, nitrogen heterocyclic (10 mmol), triphenylphosphine (0.0521 g, 0.2 mL), sodium acetate (0.1641 g, 2 mmol), acetic acid (0.3 mL, 2mmol) and ethyl propiolate (1.21 mL, 12 mmol) were added to toluene (100 mL) at 110 °C under N_2. The reaction was stirred for 12 hours. The reaction mixture was extracted with CH_2Cl_2 three times. The organic phase was dried over anhydrous sodium sulfate. The solvent was removed and purified by flash column chromatography on silica gel (PE/EA = 2/1) to afford the product[1].

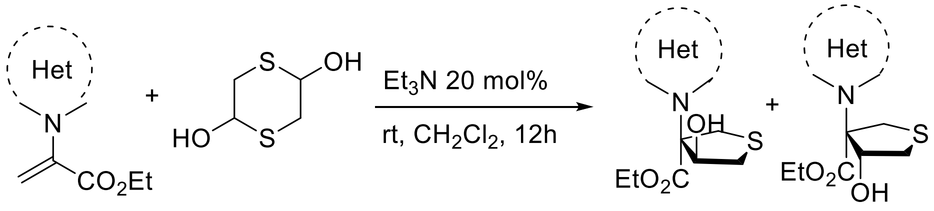

In a round bottom flask, α-N-heterocyclic acrylate A (0.05 mmol) and 1,4-dithiane-2,5-diol B (0.03 mmol) were added. Subsequently, Then, triethylamine (20 mmol%) was added and the mixture was stirred at room temperature for 4 hours. The reaction was detected by TLC, the solvent was removed and purified by flash column chromatography on silica gel (PE/EA = 2/1) to afford the product[2].

1.2. ^1H NMR and ^{13}C NMR data

*1.2.1. (±)Ethyl-4-hydroxy-3-(9H-purin-9-yl)tetrahydrothiophene-3-carboxylate (**1a, 1b**)*

Ethyl 2-(9*H*-purin-9-yl) acrylate (43.6mg, 0.2 mmol), 1,4-dithiane-2,5-diol (18.2 mg, 0.12 mmol), triethylamine (4.0 mg, 20 mol%) and dichloromethane (4 mL) were added to a reaction tube along with a magnetic stirring bar. Which was monitored by TLC, and

deemed complete after 12 hours. The mixture was then subjected to flash chromatography using a PE/EA solvent system (PE/EA = 2/1) for purification, resulting in the isolation of the products. **1a**: white solids, 41.2% yield. ^{1}H NMR (600 MHz, $CDCl_3$) δ 9.18 (s, 1H), 8.95 (s, 1H), 8.42 (s, 1H), 5.42 (d, J = 42.6 Hz, 2H), 4.24–4.16 (m, 2H), 3.96 (d, J = 12.6 Hz, 1H), 3.59 (d, J =12 Hz, 1H), 3.19 (dd, J = 4.8 Hz, J = 7.2 Hz, 1H), 2.86 (dd, J = 4.8 Hz, J = 11.4 Hz, 1H), 1.08 (t, J = 6.6 Hz, 3H).^{13}C NMR (150 MHz, $CDCl_3$) δ 169.3, 152.4, 151.5, 149.6,144.0, 134.3, 78.9, 74.0, 63.3, 35.5, 35.4, 13.9. ESI-HRMS (m/z) calcd $C_{12}H_{15}N_4O_3S$ (M + H)$^+$, 295.0859; found, 295.0856. HPLC purity: 97.7%. **1b**: white solids, 52.4% yield. ^{1}H NMR (600 MHz, $CDCl_3$) δ 9.06 (s, 1H), 8.83 (s, 1H), 8.51 (s, 1H), 5.49 (t, J = 6.0 Hz, 1H), 4.22–4.15 (m, 2H), 3.74 (dd, J =12 Hz, J = 30.0 Hz, 2H), 3.34 (q, J = 5.4 Hz, 1H), 2.89(q, J = 6, 1H), 1.12 (t, J =7.2 Hz, 3H), ^{13}C NMR (150 MHz, $CDCl_3$) δ 168.1, 152.1, 151.7, 148.9, 145.0, 133.7, 76.3, 73.8, 63.1, 34.7, 34.0, 13.8; ESI-HRMS (*m/z*) calcd $C_{12}H_{14}N_4O_3SNa$ (M + Na)$^+$, 317.0679; found, 317.0678. HPLC purity: 98.0%.

*1.2.2. (±)Ethyl-3-(6-chloro-9H-purin-9-yl)-4-hydroxytetrahydrothiophene-3-carboxylate (**2a**, **2b**)*

Compounds **2a,** and **2b** were prepared as described for the preparation of **1a**, and **1b** except ethyl 2-(6-chloro-9*H*-purin-9-yl) acrylate was used in place of ethyl 2-(9*H*-purin-9-yl) acrylate. **2a**: colorless 1iquid, 42.4% yield. ^{1}H NMR (600 MHz, $CDCl_3$) δ 8.70 (s, 1H), 8.56(s, 1H), 5.46 (s, 1H), 4.23 (t, J =7.2 Hz,2H), 3.79 (d, J = 11.4 Hz, 1H), 3.64 (d, J = 11.4 Hz, 1H), 3.32 (q, J = 6.0 Hz, 1H), 2.83 (dd, J = 6.6 Hz, J = 11.4 Hz, 1H)1.17 (t, J = 7.2 Hz, 3H).^{13}C NMR (150 MHz, $CDCl_3$) δ 167.9, 152.2, 152.0, 151.8, 144.7, 131,7, 74.0, 63.5, 34.9, 33.7, 14.0; ESI-HRMS (*m/z*) calcd $C_{12}H_{13}N_4ClO_3SNa$ (M + Na)$^+$, 351.0281; found, 351.0289. HPLC purity: 98.3%. **2b**: colorless 1iquid, 58.3% yield. ^{1}H NMR (600 MHz, $CDCl_3$) δ 8.71 (s, 1H), 8.48 (s, 1H), 5.34(s, 1H), 5.14 (s, 1H), 4.20 (d, J = 6.6 Hz, 2H), 3.97 (d, J = 12.6 Hz, 1H), 3.15 (dd, J = 4.2 Hz, J = 12, 1H), 2.82 (dd, J = 4.8 Hz, J = 11.4 Hz, 1H), 1.09 (t, J = 7.2 Hz,3H).^{13}C NMR (150 MHz, $CDCl_3$) δ 169.1, 152.1, 151.8, 151.7, 143.8,132.0, 78.8, 74.2, 63.5, 53.6, 35.5, 35.4, 14.0; ESI-HRMS (*m/z*) calcd $C_{12}H_{13}N_4ClO_3SNa$ (M + Na)$^+$, 351.0289; found, 351.0294. HPLC purity: 98.8%.

*1.2.3. (±)Ethyl-3-(6-bromo-9H-purin-9-yl)-4-hydroxytetrahydrothiophene-3-carboxylate (**3a**, **3b**)*

Compounds **3a**, and **3b** were prepared as described for the preparation of **1a**, and **1b** except ethyl 2-(6-bromo-9*H*-purin-9-yl) acrylate was used in place of ethyl 2-(9*H*-purin-9-yl) acrylate. **3a**: white solid, 30.1% yield. ^{1}H NMR (400 MHz, $CDCl_3$) δ 8.61 (s, 1H), 8.58 (s, 1H), 5.46 (s, 1H), 4.27–4.17 (m,2H), 4.15–4.12 (m, 1H), 3.73 (dd, *J* = 12.0 Hz, *J* = 28.4 Hz, 2H), 3.33 (q, *J* = 5.6 Hz, 1H), 2.84 (dd, *J* = 6.4 Hz, *J* = 11.2 Hz, 1H), 1.14 (t, *J* = 7.2 Hz, 3H). ^{13}C NMR (100 MHz, $CDCl_3$) δ 167.9, 151.7, 150.9, 144.8, 143.9, 134.1,74.1, 63.4, 34.7, 33.9, 13.9. ESI-HRMS (*m/z*) calcd $C_{12}H_{13}N_4BrO_3SNa$ $(M + Na)^+$, 394.9789; found, 394.9779. HPLC purity: 95.5%. **3b**: white solid, 41.2% yield. ^{1}H NMR (400 MHz, $CDCl_3$) δ 8.66 (s, 1H), 8.50 (s, 1H), 5.34 (s, 1H), 5.12(s, 1H), 4.20 (q, *J* = 7.2 Hz, 2H), 3.96 (d, *J* = 12.4 Hz, 1H), 3.58 (d, *J* = 12.8 Hz, 1H), 3.15 (dd, *J* = 4.0 Hz, *J* = 11.6 Hz, 1H), 2.81 (dd, *J* = 4.8 Hz, *J* = 11.6 Hz, 1H), 1.10 (t, *J* = 7.2 Hz, 3H).^{13}C NMR (100 MHz, $CDCl_3$) δ 169.0, 151.7, 150.4, 144.1, 143.7, 134.5, 78.7, 74.3, 63.5, 35.5, 35.3, 13.9. ESI-HRMS (*m/z*) calcd $C_{12}H_{13}N_4BrO_3SNa$ $(M + Na)^+$, 394.9789; found, 394.9784. HPLC purity: 98.1%.

1.2.4. (±)*Ethyl-3-(6-(dimethylamino)-9H-purin-9-yl)-4-hydroxytetrahydrothiophene-3-carboxylate (**4a**, **4b**)*

Compounds **4a**, and **4b** were prepared as described for the preparation of **1a**, and **1b** except ethyl 2-(6-(dimethylamino)-9*H*-purin-9-yl) acrylate was used in place of ethyl 2-(9*H*-purin-9-yl) acrylate. **4a**: white solid, 38.1% yield.^{1}H NMR (400 MHz, $CDCl_3$) δ 8.49 (s, 1H), 8.19 (s, 1H), 5.84 (s, 1H), 5.32 (t, *J* = 5.2 Hz, 1H), 4.23-4.15 (m, 8H), 3.93 (d, *J* = 12.4 Hz, 1H), 3.57 (d, *J* = 12.0 Hz, 1H), 3.21 (q, *J* = 5.2 Hz, 1H), 2.89 (dd, *J* = 5.2 Hz, *J* = 11.6 Hz, 1H), 1.10 (t, *J* = 7.2 Hz, 3H). ^{13}C NMR (100 MHz, $CDCl_3$) δ 69.4, 161.6, 152.0, 151.9, 141.0, 121.8, 79.3, 74.1, 63.1, 54.6, 35.6, 35.3, 14.0. ESI-HRMS (*m/z*) calcd $C_{14}H_{20}N_5O_3S$ $(M + H)^+$, 338.1281; found, 338.1272. HPLC purity: 98.9%. **4b**: white solid, 59.2% yield.^{1}H NMR (600 MHz, $CDCl_3$) δ 8.22 (s, 1H), 8.00 (s, 1H), 6.28 (s, 1H), 5.40 (t, *J* =6.6,1H), 4.25-4.18 (m, 2H), 3.74(d, *J* = 12 Hz, 1H), 3.54-3.44 (m, 7H), 3.22 (dd, *J* = 6. 6Hz, *J* = 10.8 Hz, 1H), 2.81 (dd, *J* = 7.2 Hz, *J* = 10.8 Hz, 1H), 1.18(t, *J* =7.2 Hz, 3H). ^{13}C NMR (150 MHz, $CDCl_3$) δ 168.6, 155.2, 151.8, 150.4, 137.8, 120.1, 73.9, 63.0, 35.3, 33.3, 14.0. ESI-HRMS (*m/z*) calcd $C_{14}H_{20}N_5O_3S$ $(M + H)^+$, 338.1281; found, 338.1280. HPLC purity: 95.2%.

*1.2.5. (±)Ethyl-4-hydroxy-3-(6-(piperidin-1-yl)-9H-purin-9-yl)tetrahydrothiophene-3-carboxylate (**5a, 5b**)*

Compounds **5a**, and **5b** were prepared as described for the preparation of **1a**, and **1b** except ethyl 2-(6-(piperidin-1-yl)-9*H*-purin-9-yl) acrylate was used in place of ethyl 2-(9*H*-purin-9-yl) acrylate. **5a**: white solid, 41.7% yield.^{1}H NMR (400 MHz, $CDCl_3$) δ 8.24 (s, 1H), 7.90 (s, 1H), 5.25 (t, *J* = 5.6 Hz, 1H), 4.28–4.15 (m, 6H), 3.87 (d, *J* = 12.0 Hz, 1H), 3.53 (d, *J* = 12.0 Hz, 1H), 3.25 (dd, *J* = 6.0, *J* = 11.2 Hz, 1H), 2.94 (dd, *J* = 11.2, *J* = 5.2 Hz, 1H), 1.76–1.69 (m, 6H), 1.15 (t, *J* = 7.2 Hz, 3H).^{13}C NMR (100 MHz, $CDCl_3$) δ 169.8, 154.1, 151.9, 150.9, 136.8, 119.8, 79.7, 74.0, 63.0, 35.9, 35.1, 26.3, 24.9, 14.1. ESI-HRMS (*m/z*) calcd $C_{17}H_{24}N_5O_3S$ $(M + H)^+$, 378.1594; found, 378.1597. HPLC purity: 98.7%. **5b**: white solid, 51.1% yield. ^{1}H NMR (600 MHz, $CDCl_3$) δ 8.19 (s, 1H), 7.99 (s, 1H), 5.39(t, *J* = 6.6, 1H), 4.24 - 4.18 (m, 6H), 3.72 (d, *J* = 12, 1H), 3.44 (d, *J* = 12, 1H), 3.21 (q, *J* = 6,1H), 2.80 (q, *J* = 7.2, 1H), 1.74-1.67(m, 6H), 1.18 (t, *J* = 6.6, 3H).^{13}C NMR (150 MHz, $CDCl_3$) δ 168.6, 154.1, 151.9, 150.7, 137.5, 119.6, 73.8, 63.0, 35.2, 33.4, 26.3, 24.8, 14.0. ESI-HRMS (*m/z*) calcd $C_{17}H_{23}N_5O_3SNa$ $(M + Na)^+$, 400.1414; found, 400.1410. HPLC purity: 95.8%.

*1.2.6. (±)Ethyl-3-(6-ethoxy-9H-purin-9-yl)-4-hydroxytetrahydrothiophene-3-carboxylate (**6a, 6b**)*

Compounds **6a**, and **6b** were prepared as described for the preparation of **1a**, and **1b** except ethyl 2-(6-ethoxy-9H-purin-9-yl) acrylate was used in place of ethyl 2-(9*H*-purin-9-yl) acrylate. **6a**: white solid, 39.1% yield. ^{1}H NMR (600 MHz, $CDCl_3$) δ 8.47 (s, 1H), 8.17 (s, 1H), 5.90 (d, *J* = 2.4 Hz, 1H), 5.31 (t, *J* = 5.4 Hz, 1H), 4.69-4.63 (m, 2H), 4.23–4.15 (m, 2H), 3.92 (d, *J* = 12.6 Hz, 1H), 3.67 (d, *J* = 12.0 Hz, 1H), 3.21 (q, *J* = 5.4 Hz, 1H), 2.89 (dd, *J* = 4.8 Hz, *J* = 11.4 Hz, 1H), 1.51 (t, *J* = 7.2 Hz, 3H) , 1.10 (t, *J* = 7.2 Hz, 3H). ^{13}C NMR (100 MHz, $CDCl_3$) δ 169.8, 161.7, 152.4,152.4, 141.3, 122.1, 79.7, 74.5, 64.0, 63.5, 36.1, 35.7, 15.0, 14.4. ESI-HRMS (*m/z*) calcd $C_{14}H_{18}N_4O_4SNa$ $(M + Na)^+$, 361.0941; found, 361.0938. HPLC purity: 95.4%. **6b**: white solid, 53.2% yield.^{1}H NMR (600 MHz, $CDCl_3$) δ 8.43 (s, 1H), 8.22 (s, 1H), 5.46(t, *J* = 6.0 Hz, 1H), 4.64 (q, *J* = 7.2 Hz, 2H), 4.23–4.63 (m, 2H), 4.23–4.16 (m, 2H), 3.74 (d, *J* = 12.0 Hz, 1H), 3.59 (d, *J* = 11.4 Hz, 1H), 3.30 (q, *J* = 6.0 Hz, 1H), 2.87 (dd, *J* = 6.0 Hz, J = 7.2 Hz, 1H), 1.51(t, *J* = 7.2 Hz, 3H),1.15 (t, *J* = 7.2 Hz, 3H).^{13}C NMR (100 MHz, $CDCl_3$) δ 168.3, 161.2, 152.0, 151.9, 141.8, 121.4, 76.7, 74.1,

63.6, 63.1, 35.0, 33.8, 14.6, 13.9. ESI-HRMS (*m/z*) calcd $C_{14}H_{18}N_4O_4SNa$ (M + Na)$^+$, 361.0941; found, 361.0936. HPLC purity: 96.0%.

*1.2.7. (±)Ethyl-4-hydroxy-3-(6-methoxy-9H-purin-9-yl)tetrahydrothiophene-3-carboxylate (**7a**, **7b**)*

Compounds **7a**, and **7b** were prepared as described for the preparation of **1a**, and **1b** except ethyl 2-(6-methoxy-9*H*-purin-9-yl) acrylate was used in place of ethyl 2-(9*H*-purin-9-yl) acrylate. **7a**: light yellow solid, 32.8% yield. ^{1}H NMR (600 MHz, $CDCl_3$) δ 8.49 (s, 1H), 8.19 (s, 1H), 5.31 (t, *J* = 7.8 Hz, 1H), 4.21–4.15 (m, 5H), 3.92 (d, *J* =18.6 Hz, 1H), 3.57 (d, *J* = 18 Hz,1H), 3.20 (dd, *J* = 7.8 Hz, *J* = 17.4 Hz, 1H), 2.89 (dd, *J* = 7.2 Hz, *J* = 17.4 Hz, 1H), 1.09 (t, *J* = 10.2 Hz, 3H).^{13}C NMR (150 MHz, $CDCl_3$) δ 169.8, 155.2, 151.9, 150.7, 137.1, 120.1, 79.7, 74.0, 62.9, 53.6, 35.9, 35.1,1 4.1. ESI-HRMS (m/z) calcd $C_{13}H_{16}N_4O_4SNa$ (M + Na)$^+$, 347.0784; found, 347.0786. HPLC purity: 95.1%. **7b**: light yellow solid, 54.1% yield. ^{1}H NMR (400 MHz, $CDCl_3$) δ 8.47 (s, 1H), 8.25 (s, 1H), 5.46 (t, *J* = 6.4 Hz, 1H), 4.21(t, *J* = 7.2 Hz, 5H), 3.76 (d, *J* =11.6 Hz, 1H), 3.57 (d, *J* = 11.2 Hz, 1H), 3.29 (q, *J* = 5.6 Hz, 1H), 2.86 (dd, *J* = 6.4 Hz, *J* =11.2 Hz, 1H), 1.16 (t, *J* = 7.2 Hz, 3H).^{13}C NMR (100 MHz, $CDCl_3$) δ 168.3, 161.6, 152.0, 151.9, 141.9, 74.0, 63.2, 54.6, 35.1, 33.7, 14.0. ESI-HRMS (*m/z*) calcd $C_{13}H_{16}N_4O_4SNa$ (M + Na)$^+$,347.0784; found, 347.0778. HPLC purity: 95.7%.

*1.2.8. (±)Ethyl-4-hydroxy-3-(6-(propylthio)-9H-purin-9-yl)tetrahydrothiophene-3-carboxylate (**8a**, **8b**)*

Compounds **8a**, and **8b** were prepared as described for the preparation of **1a**, and **1b** except ethyl 2-(6-(propylthio)-9*H*-purin-9-yl) acrylate was used in place of ethyl 2-(9*H*-purin-9-yl) acrylate. **8a**: yellow solid, 31.2% yield. ^{1}H NMR (400 MHz, $CDCl_3$) δ 8.63 (s, 1H), 8.28 (s, 1H), 5.48 (t, *J* = 6.0 Hz, 1H), 4.22(q, *J* =7.2 Hz, 2H), 3.78 (d, *J* =12.0 Hz, 1H),3.56 (d, *J* = 11.6 Hz, 2H), 3.37(t, *J* = 7.2 Hz, 2H), 3.29 (dd, *J* =6.0 Hz, *J* = 11.2, 1H),2.86 (dd, *J* = 6.8 Hz, *J* = 11.2 Hz, 1H), 1.81.78 (m,2H), 1.18 (t, *J* =7.2 Hz, 3H),1.09 (t, *J* =7.6, 3H). ^{13}C NMR (100 MHz, $CDCl_3$) δ 168.3, 163.0, 151.5, 148.4, 142.2, 131.3, 74.0, 63.2, 35.1, 33.6, 30.9, 22.9, 14.0, 13.6. ESI-HRMS (*m/z*) calcd $C_{15}H_{20}N_4O_3S_2Na$ (M + Na)$^+$, 391.0869; found, 391.0870. HPLC purity: 98.0%. **8b**: yellow solid, yield: 54.1%. ^{1}H NMR (400 MHz, $CDCl_3$) δ 8.64 (s, 1H), 8.22 (s, 1H),5.32 (t, *J* = 4.8 Hz, 1H), 4.25-4.14 (m, 2H),

3.92 (d, *J* = 12.4 Hz, 1H), 3.56 (d, *J* = 12.4 Hz, 1H), 3.42-3.31 (m, 2H), 3.19 (dd, *J* = 5.2 Hz, *J* = 11.6 Hz, 1H), 2.87 (dd, *J* = 4.8 Hz, *J* = 11.6 Hz, 1H), 1.86–1.77 (m, 2H), 1.13–1.06 (m, 6H). ^{13}C NMR (100 MHz, $CDCl_3$) *δ* 169.4, 162.9, 151.5, 148.3, 141.2, 131.5, 79.2, 74.0, 63.2, 35.6, 35.3, 30.9, 22.9, 14.0, 13.5. ESI-HRMS (m/z) calcd $C_{15}H_{20}N_4O_3S_2Na$ (M + Na)$^+$, 391.0869; found, 391.0865. HPLC purity: 96.4%.

*1.2.9. (±)Ethyl-4-hydroxy-3-(6-morpholino-9H-purin-9-yl) tetrahydrothiophene-3-carboxylate (**9a**, **9b**)*

Compounds **9a**, and **9b** were prepared as described for the preparation of **1a**, and **1b** except ethyl 2-(6-morpholino-9*H*-purin-9-yl) acrylate was used in place of ethyl 2-(9*H*-purin-9-yl) acrylate.**9a**: white solid, 24.4% yield. ^{1}H NMR (400 MHz, $CDCl_3$) *δ* 8.27 (s, 1H), 7.94 (s, 1H), 5.26 (t, *J* = 5.6 Hz, 1H), 4.31 (s, 4H), 4.26–4.15 (m, 2H), 3.88 (d, *J* = 12.4 Hz, 1H), 3.83 (d, *J* = 4.0 Hz, 4H), 3.53 (d, *J* = 12.0 Hz, 1H), 3.23 (q, *J* = 6.0 Hz, 1H), 2.92 (dd, *J* = 5.2 Hz, *J* = 11.6 Hz, 1H), 1.15 (t, *J* = 6.8 Hz, 3H). ^{13}C NMR (100 MHz, $CDCl_3$) *δ* 169.7, 154.1, 151.9, 151.1, 137.3. 120.0, 79.6, 74.0, 67.1, 63.0, 35.8, 35.2, 14.1.ESI-HRMS (*m/z*) calcd $C_{16}H_{22}N_5O_4S$ (M +H)$^+$, 380.1387; found, 380.1390. HPLC purity: 97.9%. **9b:** white solid, 45.4% yield. ^{1}H NMR (400 MHz, $CDCl_3$) *δ* 8.23 (s, 1H), 8.02 (s, 1H), 5.94(s, 1H), 5.40 (t, *J* = 6.4 Hz, 1H), 4.29 (s, 4H), 4.21 (q, *J* = 7.2 Hz, 2H), 3.81 (t, *J* = 4.8 Hz, 4H), 3.73 (d, *J* = 11.6 Hz, 1H), 3.46 (d, *J* = 11.6 Hz, 1H), 3.23 (dd, *J* = 6.0 Hz, *J* = 10.8 Hz, 1H), 2.80 (dd, *J* = 7.2 Hz, *J* = 10.8 Hz, 1H), 1.18 (t, *J* = 6.8 Hz, 3H). ^{13}C NMR (100 MHz, $CDCl_3$) *δ* 168.5, 154.1, 151.8, 150.9, 138.1, 119.8, 77.3, 73.8, 67.1, 63.0, 35.2, 33.4, 14.0.ESI-HRMS (*m/z*) calcd $C_{16}H_{21}N_5O_4SNa$ (M + Na)$^+$, 402.1206; found, 402.1196. HPLC purity: 99.4%.

1.2.10. (±)*Ethyl-4-hydroxy-3-(6-thiomorpholino-9H-purin-9-yl)tetrahydrothiophene-3-carboxylate (**10a**, **10b**)*

Compounds **10a**, and **10b** were prepared as described for the preparation of **1a**, and **1b** except ethyl 2-(6-thiomorpholino-9*H*-purin-9-yl) acrylate was used in place of ethyl 2-(9*H*-purin-9-yl) acrylate. **10a**: white solid, 30.4% yield. ^{1}H NMR (400 MHz, $CDCl_3$) *δ* 8.27 (s, 1H) 7.94 (s, 1H), 5.27 (t, *J* = 5.6 Hz, 1H), 4.59(s, 4H), 4.26–4.18 (m, 2H), 3.88 (d, *J* = 12 Hz, 1H), 3.54 (d, *J* = 12 Hz, 1H), 3.24 (q, *J* = 6 Hz, 1H), 2.93 (dd, *J* = 5.2 Hz, *J* = 11.2 Hz, 1H), 2.76 (t, *J* = 5.2 Hz, 4H), 1.16 (t, *J* = 7.2 Hz, 3H). ^{13}C NMR (100 MHz, $CDCl_3$) *δ*

160.7, 153.9, 151.9, 151.2, 137.3, 120.0, 79.6, 74.0, 63.0, 35.9, 35.2, 27.5, 14.1.ESI-HRMS (*m/z*) calcd $C_{16}H_{22}ClN_5O_3S_2$ (M +H)$^+$, 396.1159; found, 396.1149. HPLC purity: 96.1%. **10b**: white solid, 45.2% yield. ^{1}H NMR (600 MHz, CDCl$_3$) δ 8.27 (s, 1H) ,7.94 (s, 1H), 6.46 (s, 1H), 5.27 (t, *J* = 6 Hz, 1H) 4.59 (s, 4H), 4.25–4.19 (m, 2H), 3.88 (d, *J* = 12 Hz, 1H), 3.54 (d, *J* = 12 Hz, 1H), 3.24 (q, *J* = 6 Hz, 1H), 2.93 (q, *J* = 5.4 Hz, 1H), 2.77 (t, *J* = 4.8 Hz, 4H), 1.16 (t, *J* = 7.2 Hz, 3H).^{13}C NMR (150 MHz, CDCl$_3$) δ 169.7, 153.9, 151.9, 151.2, 120.0, 79.6, 74.0, 63.0, 35.9, 35.2, 27.5, 14.1. ESI-HRMS (*m/z*) calcd $C_{16}H_{21}ClN_5O_3S_2Na$ (M + Na)$^+$, 418.0978; found, 418.0980. HPLC purity: 97.4%.

*1.2.11. (±)Ethyl-4-hydroxy-3-(6-(4-methylpiperidin-1-yl)-9H-purin-9yl) tetrahydrothiophene-3-carboxylate (**11a, 11b**)*

Compounds **11a**, and **11b** were prepared as described for the preparation of **1a**, and **1b** except ethyl 2-(6-(4-methylpiperidin-1-yl)-9*H*- purin-9-yl) acrylate was used in place of ethyl 2-(9*H*-purin-9-yl) acrylate. **11a**: white solid, 32.7% yield. ^{1}H NMR (600 MHz, CDCl$_3$) δ 8.23 (s, 1H), 7.89 (s, 1H), 6.66 (s, 1H), 5.41 (s, 2H), 5.24 (t, *J* = 8.4 Hz, 1H), 4.24-4.16 (m, 2H), 3.86 (d, *J* = 18 Hz, 1H), 3.52 (d, *J* = 17.4 Hz, 1H) 3.24 (dd, *J* = 6.6 Hz, *J* = 9 Hz, 1H), 3.08 (s, 2H), 2.93 (q, *J* = 7.8 Hz, 1H), 1.82–1.66 (m, 2H), 1.31–1.19 (2H), 1.14 (t, *J* = 10.2 Hz, 3H), 0.97 (d, *J* = 10.2 Hz, 3H). ^{13}C NMR (150 MHz, CDCl$_3$) δ 169.8, 154.0, 151.9, 150.9, 136.8, 119.8, 82.3, 79.6, 73.9, 62.9, 35.9, 35.1, 34.4, 31.3, 22.0, 14.0. ESI-HRMS (*m/z*) calcd $C_{18}H_{25}ClN_5O_3SNa$ (M + Na)$^+$, 392.1751; found, 392.1744. HPLC purity: 95.4%. **11b**: white solid, 31.5% yield. ^{1}H NMR (400 MHz, CDCl$_3$) δ 8.21 (s, 1H), 8.00 (s, 1H), 5.40 (t, *J* = 6.8 Hz, 4H), 4.22 (dd, *J* = 7.2 Hz, *J* = 15.6 Hz, 2H), 3.74 (d, *J* = 12 Hz, 1H), 3.43 (d, *J* = 12 Hz, 1H), 3.22 (dd, *J* = 6.0 Hz, *J* = 10.8 Hz, 1H), 3.09 (s, 2H), 2.81 (dd, *J* = 7.2 Hz, *J* = 10.8 Hz, 1H), 1.83-1.70 (m, 2H), 1.32-1.27 (m, 2H), 1.19 (t, *J* = 7.2 Hz, 3H), 0.98 (d, *J* = 6.4 Hz, 3H).^{13}C NMR (100 MHz, CDCl$_3$) δ 168.7, 154.1, 151.9, 150.7, 137.5, 119.7, 73.8, 63.0, 35.3, 34.5, 33.3, 31.4, 22.0, 14.0. ESI-HRMS (*m/z*) calcd $C_{18}H_{25}ClN_5O_3SNa$ (M + Na)$^+$, 392.1751; found, 392.1746. HPLC purity: 96.7%.

*1.2.12. (±)Ethyl-3-(6-(4-fluoropiperidin-1-yl)-9H-purin-9-yl)-4-hydroxytetrahydrothiophene-3-carboxylate (**12a, 12b**)*

Compounds **12a**, and **12b** were prepared as described for the preparation of **1a**, and **1b** except ethyl 2-(6-(4-fluoropiperidin-1-yl)-9*H*-purin-9-yl) acrylate was used in place

of ethyl 2-(9*H*-purin-9-yl) acrylate. **12a**: white solid, 42.3% yield. ^{1}H NMR (600 MHz, $CDCl_3$) δ 8.21 (s, 1H), 7.90 (s, 1H), 6.48 (s, 1H), 5.21 (t, J = 6 Hz, 1H), 4.93-4.84 (m, 1H), 4.46 (s, 2H) 4.19-4.13 (m, 4H), 3.83 (d, J = 12 Hz, 1H), 3.50 (d, J = 12.6 Hz, 1H), 3.18 (q, J = 6 Hz, 1H), 2.89 (dd, J = 4.8 Hz, J = 11.4 Hz, 1H), 1.99-1.90 (m, 4H), 1.10 (t, J = 7.2 Hz, 3H). ^{13}C NMR (150 MHz, $CDCl_3$) δ 169.5, 153.8, 151.8, 150.9, 137.0, 119.7, 88.6, 87.5, 79.4, 73.9, 62.8, 42.3, 35.7, 35.0, 31.4 ($Jc\text{-}F$ = 19.7), 13.9.^{19}F NMR (376MHz, $CDCl_3$) δ -181.58. ESI-HRMS (**m/z**) calcd $C_{17}H_{22}FN_5O_3SNa$ $(M + Na)^+$, 396.1500; found, 396.1506. HPLC purity: 98.9%. **12b**: white solid, 53.4% yield. ^{1}H NMR (400 MHz, $CDCl_3$) δ 8.24 (s, 1H), 8.03 (s, 1H), 6.10 (s, 1H), 5.41 (t. J = 6.0 Hz, 1H), 5.03-4.86 (m,1H), 4,54 (s, 2H), 4.22 (q, J = 7.2 Hz, 4H), 3.75 (d, J = 12 Hz, 1H), 3.44 (d, J = 11.6 Hz, 1H), 3.23 (dd, J = 10.8 Hz, J = 6.0 Hz, 1H), 2.81 (dd, J = 10.8 Hz, J = 7.2 Hz, 1H), 2.05-1.95 (m, 4H), 1.20 (t, J = 7.2 Hz, 3H).^{13}C NMR (100 MHz, $CDCl_3$) δ 168.6, 154.1, 151.9, 150.9, 137.9, 119.8, 87.3, 73.8, 63.1, 35.3, 33.3, 31.6 (d, $J_{C\text{-}F}$ = 20.2 Hz), 14.0.^{19}F NMR (376MHz, $CDCl_3$) δ -181.68.ESI-HRMS (*m/z*) calcd $C_{17}H_{22}FN_5O_3SNa$ $(M + Na)^+$, 396.1500; found, 396.1508. HPLC purity: 97.2%.

*1.2.13. (±)Ethyl-4-hydroxy-3-(6-(4-(trifluoromethyl) piperidin-1-yl)-9H-purin-9-yl)tetrahydrothiophene-3-carboxylate (**13a**, **13b**)*

Compounds **13a**, and **13b** were prepared as described for the preparation of **1a**, and **1b** except ethyl 2-(6-(4-(trifluoromethyl) piperidin-1-yl)-9*H*-purin-9-yl) acrylate was used in place of ethyl 2-(9*H*-purin-9-yl) acrylate. **13a**: yellow solid, 42.1% yield. ^{1}H NMR (400 MHz, $CDCl_3$) δ 8.25 (s, 1H), 7.93 (s, 1H), 6.44 (s, 1H), 5.60 (s, 2H), 5.24 (t. J = 5.6 Hz, 1H), 4.23–4.15(m, 2H), 3.86 (d, J = 12 Hz, 1H), 3.53 (d, J = 12 Hz, 1H), 3.20 (q, J = 6 Hz, 1H), 3.08–2.99 (m, 2H) 2.95 (q, J = 5.6 Hz, 1H), 2.42-2.33 (m, 1H), 2.00 (d, J = 12.8 Hz, 2H), 1.69–1.56 (m, 2H), 1.13 (t. J = 6.8 Hz, 3H).^{13}C NMR (100 MHz, $CDCl_3$) δ 169.6, 153.9, 151.8, 151.0, 137.2, 128.5, 125.8, 119.8, 79.5, 73.9, 62.9, 44.2, 40.7 (q, $J_{C\text{-}F}$ = 27.4 Hz), 35.8, 35.1, 24.7, 14.0.^{19}F NMR (376MHz, $CDCl_3$)δ -73.90. ESI-HRMS (*m/z*) calcd $C_{18}H_{22}F_3N_5O_3SNa$ $(M + Na)^+$, 446.1468; found, 446.1468. HPLC purity: 95.6%. **13b**: yellow solid, 37.5% yield. ^{1}H NMR (400 MHz, $CDCl_3$) δ 8.24 (s, 1H) 8.03 (s, 1H), 5.97 (s, 1H), 5.63 (s, 2H), 5.41 (t. J = 6.4 Hz, 1H), 4.22, (q, J = 6.8 Hz, 2H), 3.75 (d, J = 12 Hz, 1H), 3.45 (d, J = 12 Hz, 1H), 3.23 (dd, J = 11.2 Hz, J = 6 Hz, 1H), 3.07 (t, J = 11.6 Hz, 2H), 2.81

(dd, J = 10.8 Hz, J = 7.2 Hz, 1H), 2.46-2.34 (m, 1H), 2.03 (d, J = 12.8 Hz, 2H), 1.72-1.61 (m, 2H), 1.25-1.15 (m, 3H). ^{13}C NMR (100 MHz, $CDCl_3$) δ 168.6, 154.0, 151.9, 151.0, 138.1, 119.8, 73.9, 63.1, 40.8 (d, $J_{C\text{–}F}$ = 27.3), 35.3, 33.3, 24.8, 14.0.^{19}F NMR (376MHz, $CDCl_3$) δ -73.90.ESI-HRMS (*m/z*) calcd $C_{18}H_{22}F_3N_5O_3SNa$ $(M + Na)^+$, 446.1468; found, 446.1467. HPLC purity: 96.7%.

*1.2.14. (±)Ethyl-3-(6-(4-chloropiperidin-1-yl)-9H-purin-9-yl)-4-hydroxytetrahydrothiophene-3-carboxylate (**14a**, **14b**)*

Compounds **14a**, and **14b** were prepared as described for the preparation of **1a**, and **1b** except ethyl 2-(6-(4-chloropiperidin-1-yl)-9*H*-purin-9-yl) acrylate was used in place of ethyl 2-(9*H*-purin-9-yl) acrylate. White solid; **14a**: light yellow solid, 51.7% yield. ^{1}H NMR (600 MHz, $CDCl_3$) δ 8.23 (s, 1H), 8.02(s, 1H), 6.03 (s, 1H), 5.40 (t, J = 6.6 Hz, 1H), 4.60 (s, 2H) 4.38-4.35 (m, 1H), 4.22 (q, J = 7.2 Hz, 4H), 3.74 (d, J = 12 Hz, 1H), 3.45 (d, J = 12 Hz, 1H), 3.23 (dd, J = 4.8 Hz, J = 10.8 Hz, 1H), 2.80 (dd, J = 7.2 Hz, J = 10.8 Hz, 1H), 2.22–2.19 (m, 2H), 2.00–1.96 (m, 2H), 1.19 (t, J = 7.2 Hz, 3H). ^{13}C NMR (100 MHz, $CDCl_3$) δ 168.5, 154.0, 151.8, 150.9, 137.9, 119.8, 73.8, 63.1, 57.0, 35.4, 35.2, 33.4, 14.0.ESI-HRMS (*m/z*) calcd $C_{17}H_{23}ClN_5O_3S$ $(M +H)^+$, 412.1205; found, 412.1198. HPLC purity: 96.1%. **14b**: light yellow solid, 31.0% yield. ^{1}H NMR (400 MHz, $CDCl_3$) δ 8.27 (s, 1H), 7.93 (s, 1H), 6.50 (s, 1H), 5.26 (t, J = 5.6 Hz, 1H), 4.62(s, 2H), 4.39-4.34 (m, 1H), 4.27–4.16 (m, 4H), 3.88 (d, J = 12.0 Hz, 1H), 3.54 (d, J = 12.0 Hz, 1H), 3.24 (q, J = 6 Hz, 1H), 2.93 (dd, J = 5.2 Hz, J = 11.6 Hz, 1H), 2.25-2.17 (m, 2H), 2.02-1.93 (m, 2H), 1.15 (t, J = 7.2 Hz, 3H).^{13}C NMR (100 MHz, $CDCl_3$) δ 169.7, 154.0, 151.9, 151.1, 137.2, 119.9, 79.6, 74.0, 63.0, 57.0, 35.9, 35.4, 35.2, 14.1. ESI-HRMS (*m/z*) calcd $C_{17}H_{23}ClN_5O_3S$ $(M +H)^+$, 412.1205; found, 412.1203. HPLC purity: 95.0%.

*1.2.15. (±)Ethyl-4-hydroxy-3-(6-(4-morpholinopiperidin-1-yl)-9H-purin-9-yl)tetrahydrothiophene-3-carboxylate (**15a**, **15b**)*

Compounds **15a**, and **15b** were prepared as described for the preparation of **1a**, and **1b** except ethyl 2-(6-(4-morpholinopiperidin-1- yl)-9*H*-purin-9-yl) acrylate was used in place of ethyl 2-(9*H*-purin-9-yl) acrylate. **15a**: white solid, 37.7% yield. ^{1}H NMR (600 MHz, $CDCl_3$) δ 8.22 (s, 1H), 8.02 (s, 1H), 5.50 (s, 1H), 5.40 (t, J = 9.6 Hz, 1H), 4.22 (q, J = 10.8 Hz, 2H), 3.76 (s, 1H), 3.72 (t, J = 6.6 Hz, 4H),3.44 (d, J = 18 Hz, 1H), 3.22 (dd, J =9

Hz, *J* = 16.2 Hz, 1H), 3.13 (s, 2H), 2.80 (dd, *J* = 11.4 Hz, *J* = 16.2 Hz, 1H), 2.59-2.52 (m, 5H), 2.01 (d, *J* = 19.2 Hz, 2H), 1.62-1.52 (m, 4H), 1.19 (t, *J* = 10.8 Hz, 3H).^{13}C NMR (150 MHz, $CDCl_3$) δ 169.6, 154.0, 150.8, 137.8, 119.8, 73.8, 67.4, 63.1, 62.2, 49.9, 35.3, 33.3, 28.6, 14.0.ESI-HRMS (*m/z*) calcd $C_{21}H_{31}N_6O_4S$ (M +H)$^+$, 463.2111; found, 463.2113. HPLC purity: 97.9%. **15b**: white solid, 38.6% yield. ^{1}H NMR (600 MHz, $CDCl_3$) δ 8.25 (s, 1H), 7.92 (s, 1H), 6.55 (s, 1H) 5.49 (s, 1H), 5.25 (t, *J* = 8.4 Hz, 1H), 4.26-4.17 (m, 2H), 3.88 (d, *J* = 18.6 Hz, 1H), 3.73 (t, *J* = 6.6 Hz, 4H), 3.53 (d, *J* = 18 Hz, 1H), 3.24 (dd, *J* = 9 Hz, *J* = 16.8 Hz, 1H), 3.12 (s, 2H), 2.93 (q *J* = 7.8 Hz, 1H), 2.60-2.53 (s, 5H), 2.02 (d, *J* = 18 Hz, 2H), 1.63-1.52 (m, 4H), 1.15 (t, *J* = 10.8 Hz, 3H).^{13}C NMR (100 MHz, $CDCl_3$) δ 169.8, 153.9, 151.9, 151.0, 137.0, 119.9, 79.6, 74.0, 67.3, 63.0, 62.2, 49.9, 35.9, 35.2, 29.8, 28.6, 14.1.ESI-HRMS (*m/z*) calcd $C_{21}H_{31}N_6O_4S$ (M +H)$^+$, 463.2111; found, 463.2120. HPLC purity: 95.9%.

*1.2.16. (±)Ethyl-3-(6-(4-benzylpiperidin-1-yl)-9H-purin-9-yl)-4-hydroxytetrahydrothiophene-3-carboxylate (**16a, 16b**)*

Compounds **16a**, and **16b** were prepared as described for the preparation of **1a**, and **1b** except ethyl 2-(6-(4-methylpiperidin-1-yl)-9*H*-purin-9-yl) acrylate was used in place of ethyl 2-(9*H*-purin-9-yl) acrylate. **16a**: white solid, 32.4% yield. ^{1}H NMR (600 MHz, $CDCl_3$) δ 8.18 (s, 1H), 7.97 (s, 1H), 7.28–7.11 (m, 5H), 5.37 (t, *J* = 10.0 Hz. 3H), 4.22-4.16 (m, 2H), 3.71 (d, *J* = 17.4 Hz, 1H), 3.41 (d, *J* = 17.4 Hz, 1H) 3.21 (dd, *J* = 9.6 Hz, *J* = 16.8 Hz, 1H) , 2.99 (s, 2H), 2.78 (dd, *J* = 10.8 Hz, *J* = 16.2 Hz, 1H) 2.55 (d, *J* = 10.8 Hz, 2H), 1.90-1.78 (m, 4H), 1.35-1.22 (m, 1H), 1.16 (t, *J* = 10.8 Hz, 3H).^{13}C NMR (150 MHz, $CDCl_3$) δ 168.6, 154.0, 151.9, 150.7, 140.2, 137.6, 129.3, 128.4, 126.1, 119.7, 73.8,63.0, 43.3, 38.5, 35.3, 33.3, 32.4, 14.0.ESI-HRMS (*m/z*) calcd $C_{24}H_{29}N_5O_3SNa$ (M + Na)$^+$, 490.1883; found, 490.1886. HPLC purity: 97.9%. **16b**: white solid, 31.8% yield. ^{1}H NMR (600 MHz, $CDCl_3$) δ 8.21 (s, 1H), 7.87 (s, 1H), 7.28–7.11 (m, 5H), 5.43 (s, 2H), 5.23 (t, *J* = 8.4 Hz, 1H), 4.23–4.14 (m, 2H), 3.84 (d, *J* = 18 Hz, 1H), 3.50 (d, *J* = 18 Hz, 1H), 3.21 (dd, *J* =9 Hz, *J* = 16.8 Hz, 1H), 3.00 (s, 2H), 2.91 (dd, *J* = 7.8 Hz, *J* = 16.8 Hz, 1H), 2.55 (d, *J* = 10.8 Hz, 2H), 1.90–1.78 (m, 4H), 1.36-1.21 (m, 1H), 1.13 (t, *J* = 10.8 Hz, 3H).^{13}C NMR (150 MHz, $CDCl_3$) δ 169.8, 154.0, 151.9, 150.9, 140.2, 136.8, 129.3, 128.4, 126.1, 119.8, 79.7, 74.0, 62.9, 43.3, 38.5, 35.9, 35.1, 32.4, 14.1. ESI-HRMS (*m/z*) calcd $C_{24}H_{30}N_5O_3S$ (M +H)$^+$,

468.2064; found, 468.2067. HPLC purity: 99.7%.

*1.2.17. (±)Ethyl-3-(6-(4-(2-fluorobenzyl) piperidin-1-yl)-9H-purin-9-yl)-4-hydroxytetrahydrothiophene-3-carboxylate (**17a, 17b**)*

Compounds **17a**, and **17b** were prepared as described for the preparation of **1a**, and **1b** except ethyl 2-(6-(4-(2- fluorobenzyl) piperidin-1-yl)-9*H*-purin-9-yl) acrylate was used in place of ethyl 2-(9*H*-purin-9-yl) acrylate. **17a**: white solid, 24.6% yield. ^{1}H NMR (600 MHz, $CDCl_3$) *δ* 8.24 (s, 1H), 7.90 (s, 1H), 7.20-7.12 (m, 2H), 7.07-7.00 (m, 2H), 6.63 (s, 1H), 5.45 (s, 2H), 5.25 (t, *J* = 6 Hz, 1H), 4.26–4.18 (m, 1H), 3.87 (d, *J* = 11.4 Hz, 1H), 3.53 (d, *J* = 12 Hz, 1H), 3.24 (dd, *J* = 6 Hz, *J* = 10.8 Hz, 1H), 3.03 (s, 2H), 2.94 (q, *J* = 4.8 Hz, 1H), 2.62 (d, *J* = 7.2 Hz, 2H), 1.96-1.92 (m, 1H), 1.82 (d, *J* = 12.6 Hz, 2H), 1.38-1.34 (m, 2H), 1.15 (t, *J* = 7.2 Hz, 3H).^{13}C NMR (150 MHz, $CDCl_3$) *δ* 169.8, 162.2, 160.6, 154.0, 151.9, 136.8, 131.7 ($J_{C–F}$ = 5.0 Hz), 128.0 (Jc_{-F} = 7.7 Hz), 127.1 ($Jc_{–F}$ = 11.4 Hz), 123.9 ($J_{C–F}$ = 3.9 Hz), 119.8, 115.4, ($J_{C–F}$ = 22.1 Hz), 79.7, 73.9, 62.9, 37.4, 36.1, 35.9, 35.1, 32.4,14.1.^{19}F NMR (376MHZ, $CDCl_3$) δ -117.84.ESI-HRMS (*m/z*) calcd $C_{24}H_{29}FN_5O_3S$ (M + H)$^+$, 486.1970; found, 486.1980. HPLC purity: 96.4%. **17b**: white solid, 37.8% yield. ^{1}H NMR (400 MHz, $CDCl_3$) *δ* 8.24 (s, 1H), 7.90, (s, 1H), 7.20-7.00 (m, 4H), 5.47(s, 2H), 5.26 (t, *J* = 5.6 Hz, 1H), 4.26–4.17 (m, 2H), 3.87 (d, *J* = 12.4 Hz, 1H), 3.53 (d, *J* = 12 Hz, 1H), 3.24 (dd, *J* = 6.4 Hz, *J* = 11.6 Hz, 1H), 3.03 (s, 2H), 2.94 (dd, *J* = 11.2 Hz, *J* = 5.2 Hz, 1H), 2.62 (d, *J* = 7.2 Hz, 2H), 1.95 (s, 2H), 1.82 (d, *J* = 13.2 Hz, 2H), 1.64 (s, 1H), 1.15(t, *J* = 17.2 Hz, 3H). ^{13}C NMR (100 MHz, $CDCl_3$) *δ* 169.8, 154.0, 152.0, 150.9, 136.8, 131.7, 128.0 (d, J_{C-F} = 7.9), 127.1 (d, $J_{C–F}$ = 16.1 Hz), 123.9, 119.8, 115.4 (d, $J_{C–F}$ = 21.7 Hz), 79.7, 74.0, 63.0, 37.4, 36.2, 35.2, 32.3, 29.8, 14.1.^{19}F NMR (376 MHZ, $CDCl_3$) *δ* -117.86. ESI-HRMS (*m/z*) calcd $C_{24}H_{29}FN_5O_3S$ (M + H)$^+$, 486.1970; found, 486.1978. HPLC purity: 99.6%.

*1.2.18. (±)Ethyl-4-hydroxy-3-(6-(4-methylpiperazin-1-yl)-9H-purin-9-yl)tetrahydrothiophene-3-carboxylate (**18a, 18b**)*

Compounds **18a,** and **18b** were prepared as described for the preparation of **1a,** and **1b** except ethyl 2-(6-(4-methylpiperazin-1-yl)- 9*H*-purin-9-yl) acrylate was used in place of ethyl 2-(9*H*-purin-9-yl) acrylate. **18a**: white solid, 29.5% yield. ^{1}H NMR (600 MHz, $CDCl_3$) *δ* 8.23 (s, 1H), 8.02 (s, 1H), 5.40 (t, *J* = 6.6 Hz, 1H), 4.35 (s, 2H), 4.24–4.20 (m,

2H), 3.74 (d, *J* = 11.4 Hz, 1H), 3.44 (d, *J* = 11.4 Hz, 1H), 3.23 (dd, *J* = 6 Hz, *J* = 10.8 Hz, 1H), 2.80 (dd, *J* = 7.2 Hz, *J* = 10.8 Hz, 1H), 2.55(t, *J* = 5.4 Hz, 4H), 2.35 (s, 3H) 1.77 (s, 2H), 1.19 (t, *J* = 7.2 Hz, 3H). ^{13}C NMR (100 MHz, $CDCl_3$) δ 168.6, 154.1, 151.9, 150.9, 137.9, 119.8, 73.8, 63.1, 55.2, 46.2, 35.3, 33.4, 14.0.ESI-HRMS (*m/z*) calcd $C_{17}H_{24}ClN_6O_3SNa$ (M + Na)$^+$, 393.1703; found, 393.1701. HPLC purity: 98.1%. **18b**: white solid, 41.6% yield. ^{1}H NMR (600 MHz, $CDCl_3$) δ 8.26 (s, 1H), 7.93 (s, 1H), 5.26(t, *J* = 6 Hz,1H), 4.34 (s, 2H), 4.25-4.18 (m, 2H), 3.88 (d, *J* = 12 Hz, 1H), 3.54 (d, *J* = 12 Hz, 1H), 3.24 (q, *J* = 5.4 Hz, 1H), 2.93(q, *J* = 5.4 Hz, 1H), 2.56 (s, 4H) , 2.36 (s, 3H), 1.75 (s, 2H), 1.15 (t, *J* = 7.2 Hz, 3H). ^{13}C NMR (150 MHz, $CDCl_3$) δ 169.8, 154.1, 151.9, 151.0, 137.1, 119.9, 79.6, 74.0, 63.0, 55.2, 46.3, 35.9, 35.2, 14.1. ESI-HRMS (*m/z*) calcd $C_{17}H_{24}ClN_6O_3SNa$ (M + Na)$^+$,393.1703; found, 393.1699. HPLC purity: 95.6%.

*1.2.19. (±)Ethyl-3-(6-(azepan-1-yl)-9H-purin-9-yl)-4-hydroxytetrahydrothiophene-3-carboxylate (**19a, 19b**)*

Compounds **19a**, and **19b** were prepared as described for the preparation of **1a**, and **1b** except ethyl 2-(6-(azepan-1-yl)-9*H*-purin-9-yl) acrylate was used in place of ethyl 2-(9*H*-purin-9-yl) acrylate. **19a**: white solid, 31.8% yield. ^{1}H NMR (600 MHz, $CDCl_3$) δ 8.25 (s, 1H), 7.88 (s, 1H), 6.84(s, 1H) 5.30-5.24 (m, 1H), 4.45 (s, 1H), 4.28-4.20 (m, 3H), 3.99 (s, 1H), 3.88 (d, *J* = 17.4 Hz, 2H), 3.55 (d, *J* = 18 Hz, 1H), 3.27 (dd, *J* = 9.6 Hz, *J* = 16.8 Hz, 1H), 2.98 (q, *J* = 7.8 Hz, 1H), 1.89 (s, 4H), 1.62–1.57 (m, 4H), 1.17 (t, *J* = 10.8 Hz, 3H).^{13}C NMR (100 MHz, $CDCl_3$) δ 169.7, 154.5, 151.8, 150.6, 136.9, 119.6, 79.7, 73.9, 62.8, 35.9, 35.0, 14.0.ESI-HRMS (*m/z*) calcd $C_{18}H_{26}N_5O_3S$ (M +H)$^+$, 392.1751; found, 392.1744. HPLC purity: 95.7%. **19b**: white solid, 52.4% yield. ^{1}H NMR (600 MHz, $CDCl_3$) δ 8.22 (s, 1H), 7.99 (s, 1H), 6.42 (s, 1H), 5.41 (t, *J* = 6.6 Hz, 1H), 4.44 (s, 1H) 4.30–4.20 (m, 3H), 3.97 (s, 1H), 3.85 (s, 1H), 3.75 (d, *J* = 11.4 Hz, 1H), 3.44 (d, *J* = 11.4 Hz, 1H), 3.22 (dd, *J* = 6 Hz, *J* = 10.8 Hz, 1H), 2.83 (t, *J* = 7.2 Hz, 1H), 1.89 (s, 4H), 1.60 (s, 1H), 1.20 (t, *J* = 6.6 Hz, 3H).^{13}C NMR (100 MHz, $CDCl_3$) δ 168.7, 154.7, 151.9, 150.5, 137.8, 119.6, 73.8, 63.0, 50.0, 48.6, 35.3, 33.3, 29.1, 27.3, 27.0, 14.0. ESI-HRMS (*m/z*) calcd $C_{18}H_{26}N_5O_3S$ (M +H)$^+$, 392.1751; found, 392.1758. HPLC purity: 95.3%.

1.2.20. (±)*Ethyl-4-hydroxy-3-(6-(pyrrolidin-1-yl)-9H-purin-9-yl) tetrahydrothiophene-3-carboxylate (**20a, 20b**)*

Compounds **20a**, and **20b** were prepared as described for the preparation of **1a**, and **1b** except ethyl 2-(6-(pyrrolidin-1-yl)-9*H*- purin-9-yl) acrylate was used in place of ethyl 2-(9*H*-purin-9-yl) acrylate. **20a**: white solid, 26.5% yield. ^{1}H NMR (600 MHz, $CDCl_3$) δ 8.27 (s, 1H), 7.87 (s, 1H), 5.26 (t, *J* = 6.0 Hz, 1H), 4.26-4.16 (m, 4H), 3.88 (d, *J* = 12.0 Hz, 1H), 3.76 (s, 2H), 3.55 (d, *J* = 12.0 Hz, 1H), 3.27 (dd, *J* = 6.4 Hz, *J* = 11.2 Hz, 1H), 2.96 (dd, *J* = 5.2 Hz, *J* = 11.2 Hz, 1H), 2.04 (d, *J* = 26 Hz, 4H), 1.16 (t, *J* = 7.2 Hz, 3H). ^{13}C NMR (100 MHz, $CDCl_3$) δ 169.7, 153.4, 152.3, 150.3, 137.6, 120.3, 79.9, 74.0, 62.9, 36.0, 35.0, 14.1.ESI-HRMS (*m/z*) calcd $C_{16}H_{22}N_5O_3S$ (M +H)$^+$, 364.1438; found, 364.1437. HPLC purity: 98.4%. **20b**: white solid, 58.0% yield. ^{1}H NMR (600 MHz, $CDCl_3$) δ 8.22 (s, 1H), 7.96 (s, 1H), 6.43 (s, 1H), 5.41(t, *J* = 6.4 Hz, 1H), 4.30-4.15 (m, 4H), 3.72 (d, *J* = 9.2 Hz, 3H) ,3.47 (d, *J* = 11.6 Hz, 1H), 3.24 (dd, *J* = 6 Hz, *J* = 10.8 Hz, 1H), 2.84 (dd, *J* = 6.8 Hz, *J* = 10.8 Hz, 1H), 2.03 (t, *J* = 25.6 Hz, 4H), 1.18 (t, *J* = 6.8 Hz, 3H).^{13}C NMR (100 MHz, $CDCl_3$) δ 168.7, 153.3, 152.3, 150.0, 138.3, 120.1, 74.0, 63.0, 49.1, 47.7, 35.2, 33.5, 26.4, 24.4, 14.0. ESI-HRMS (*m/z*) calcd $C_{16}H_{22}N_5O_3S$ (M +H)$^+$, 364.1438; found, 364.1435. HPLC purity: 95.3%.

*1.2.21. (±)Ethyl-4-hydroxy-3-(6-(methylthio)-9H-purin-9-yl) tetrahydrothiophene-3-carboxylate (**21a, 21b**)*

Compounds **21a**, and **21b** were prepared as described for the preparation of **1a**, and **1b** except ethyl 2-(6-(methylthio)-9*H*-purin-9-yl) acrylate was used in place of ethyl 2-(9*H*-purin-9-yl) acrylate. **21a**: yellowish solid, 33.4% yield. ^{1}H NMR (400 MHz, $CDCl_3$) δ 8.63 (s, 1H), 8.22 (s, 1H), 5.74 (s, 1H), 5.30 (t, *J* = 5.2 Hz, 1H), 4.18-4.12 (m, 2H), 3.91 (d, *J* = 12.4 Hz, 1H), 3.56 (d, *J* = 12.0 Hz, 1H), 3.15 (dd, *J* = 5.2 Hz, =11.6 Hz, 1H), 2.86 (dd, *J* = 4.8 Hz, *J* = 11.6 Hz, 1H), 2.68 (s, 3H), 1.07 (t, *J* = 7.2 Hz, 3H).^{13}C NMR (150 MHz, $CDCl_3$) δ 169.1, 162.7, 151.4, 148.2, 141.3, 131.4, 79.0, 74.0, 63.0, 35.5, 35.2, 13.9, 11.8. ESI-HRMS (*m/z*) calcd $C_{13}H_{16}N_4O_3S_2Na$ (M + Na)$^+$, 363.0556; found, 363.0546. HPLC purity: 99.8%. **21b**: yellowish solid, 40.5% yield.^{1}H NMR (600 MHz, $CDCl_3$) δ 8.61 (s, 1H), 8.28 (s, 1H), 5.45 (t, *J* = 6.0 Hz, 1H), 5.04 (s, 1H), 4.23-4.14 (m, 2H), 3.69 (dd, *J* = 11.6 Hz, *J* = 36.8 Hz, 2H),3.30 (q, *J* = 6.0 Hz, 1H), 2.87 (q, *J* = 6.0 Hz, 1H), 2.67 (s, 3H), 1.12 (t, *J* = 7.2 Hz, 3H).^{13}C NMR (100 MHz, $CDCl_3$) δ 168.2, 162.6, 151.5, 148.3, 142.3, 131.1, 76.5, 74.0, 63.1, 34.8, 34.0, 13.9, 11.9. ESI-HRMS (*m/z*) calcd $C_{13}H_{16}N_4O_3S_2Na$

(M + Na)$^+$, 363.0556; found, 363.0564. HPLC purity: 99.6%.

*1.2.22. (±)Ethyl-3-(6-(ethylthio)-9H-purin-9-yl)-4-hydroxytetrahydrothiophene-3-carboxylate (**22a, 22b**)*

Compounds **22a**, and **22b** were prepared as described for the preparation of **1a**, and **1b** except ethyl 2-(6-(ethylthio)-9*H*-purin-9-yl) acrylate used in place of ethyl 2-(9*H*-purin-9-yl) acrylate. **22a**: yellow solid, 21.5% yield. ^{1}H NMR (600 MHz, CDCl$_3$) *δ* 8.58 (s, 1H), 8.28 (s, 1H), 5.44 (q, *J* = 6 Hz, 1H), 5.12 (d, *J* = 5.4 Hz, 1H), 4.18-4.13 (m, 2H), 3.73 (d, *J* = 12 Hz, 1H), 3.65 (d, *J* = 11.4 Hz, 1H), 3.34–3.28 (m, 3H), 2.86 (q, *J* = 5.4 Hz, 1H), 1.41(t, *J* = 7.8 Hz, 3H), 1.12(t, *J* = 7.2 Hz, 3H). ^{13}C NMR (150 MHz, CDCl$_3$) *δ* 168.2, 162.3, 151.5, 148.4, 142.3, 130.9, 76.5, 74.0, 63.0, 34.8, 33.9, 23.4, 14.7, 13.9.ESI-HRMS (*m/z*) calcd $C_{14}H_{18}N_4O_3S_2Na$ (M + Na)$^+$, 377.0713; found, 377.0697. HPLC purity: 99.8%. **22b**: yellow solid, 22.6% yield.^{1}H NMR (600 MHz, CDCl$_3$) *δ* 8.63 (s, 1H), 8,28 (s, 1H), 5.48(q, *J* = 6 Hz,1H), 4.81(d, *J* = 5.4 Hz, 1H), 4.23–4.19 (m, 2H), 3.77 (d, *J* = 12 Hz, 1H), 3.55 (d, *J* = 12 Hz, 1H), 3.38 (q, *J* = 7.2 Hz, 2H), 3.28 (q, *J* = 6 Hz, 1H), 2.85 (dd, *J* = 6.6 Hz, *J* = 11.4 Hz, 1H), 1.45 (t, *J* = 7.2 Hz, 3H), 1.17 (t, *J* = 7.2 Hz, 3H). ^{13}C NMR (150 MHz, CDCl$_3$) *δ* 168.2, 162.9, 151.5, 148.5, 142.2, 131.3, 74.0, 63.2, 35.1, 33.6, 23.5, 14.8, 14.0. ESI-HRMS (*m/z*) calcd $C_{14}H_{18}N_4O_3S_2Na$ (M + Na)$^+$, 377.0713; found, 377.0704. HPLC purity: 99.3%.

1.2.23. (±)*Ethyl-4-hydroxy-3-(6-(isopropylthio)-9H-purin-9-yl) tetrahydrothiophene-3-carboxylate (**23a, 23b**)*

Compounds **23a**, and **23b** were prepared as described for the preparation of **1a**, and **1b** except ethyl 2-(6-(isopropylthio)-9*H*- purin-9-yl) acrylate used in place of ethyl 2-(9*H*-purin-9-yl) acrylate. **23a**: white solid, 51.2% yield. ^{1}H NMR (600 MHz, CDCl$_3$) *δ* 8.63 (s, 1H), 8.27(s, 1H), 5.48 (s, 1H), 4.81 (s, 1H) 4.37–4.33 (m, 1H), 4.24-4.20 (m, 2H) 3.78 (d, *J* = 12 Hz, 1H), 3.53 (d, *J* = 12 Hz, 1H), 3.28 (q, *J* = 6 Hz, 1H), 2.85 (dd, *J* = 6.6 Hz, *J* = 10.8 Hz, 1H), 1.50 (d, *J* = 7.2 Hz, 6H), 1.18(t, *J* = 7.2 Hz, 3H). ^{13}C NMR (150 MHz, CDCl$_3$) *δ* 168.3, 163.2, 151.5, 148.5, 142.1, 131.2, 74.0, 63.3, 35.1, 34.7, 33.6, 23.4, 23.3, 14.0. ESI-HRMS (m/z) calcd $C_{15}H_{20}N_4O_3S_2Na$ (M + Na)$^+$, 391.0869; found, 391.0871. HPLC purity: 99.8%. **23b**: white solid, 22.4% yield. ^{1}H NMR (600 MHz, CDCl$_3$) *δ* 8.61 (s, 1H), 8.20(s, 1H), 5.76(s, 1H), 5.30 (s, 1H), 4.34-4.29 (m, 1H), 4.19-4.15 (m, 2H) 3.91 (d, *J* =

12.6 Hz, 1H), 3.56 (d, *J* = 12.6 Hz, 1H), 3.16 (dd, *J* = 4.8 Hz, *J* = 11.4 Hz, 1H), 2.86 (dd, *J* = 5.4 Hz, *J* = 12 Hz, 1H), 1.46 (d, *J* = 6.0 Hz, 6H), 1.09(t, *J* = 6.6 Hz, 3H). ^{13}C NMR (150 MHz, $CDCl_3$) *δ* 169.2, 162.8, 151.4, 148.4, 141.2, 131.2, 74.0, 63.1, 35.5, 35.2, 34.6, 23.3, 23.2, 13.9. ESI-HRMS (*m/z*) calcd $C_{15}H_{20}N_4O_3S_2Na$ $(M + Na)^+$, 391.0869; found, 391.0868. HPLC purity: 95.1%.

*1.2.24. (±)Ethyl-4-hydroxy-3-(6-(prop-2-yn-1-ylthio)-9H-purin-9-yl)tetrahydrothiophene-3-carboxylate (**24a, 24b**)*

Compounds **24a**, and **24b** were prepared as described for the preparation of **1a**, and **1b** except ethyl 2-(6-(prop-2-yn-1-ylthio)-9*H*- purin-9-yl) acrylate used in place of ethyl 2-(9*H*-purin-9-yl) acrylate. **24a**: white solid, 41.2% yield.^{1}H NMR (600 MHz, $CDCl_3$) *δ* 8.68 (s, 1H), 8.32 (s, 1H), 5.46 (s, 1H), 4.65 (s, 1H), 4.23–4.18 (m, 2H), 4.16 (d, *J* = 2.4 Hz, 2H), 3.76 (d, *J* = 12 Hz, 1H), 3.60 (d, *J* = 12 Hz, 1H), 3.29 (q, *J* = 6 Hz, 1H) , 2.85(q, *J* = 6 Hz, 1H), 2.22 (t, *J* = 2.4 Hz, 1H), 1.16(t, *J* = 7.2 Hz, 3H).^{13}C NMR (150 MHz, $CDCl_3$) *δ* 168.1, 160.2, 151.6, 148.9, 142.6, 131.0, 79.3, 74.0, 71.3, 63.2, 35.0, 33.7, 17.3, 14.0.ESI-HRMS (*m/z*) calcd $C_{15}H_{16}N_4O_3S_2Na$ $(M + Na)^+$, 387.0556; found, 387.0550. HPLC purity: 98.6%. **24b**: white solid, 49.7% yield.^{1}H NMR (600 MHz, $CDCl_3$) *δ* 8.71 (s, 1H), 8.27 (s, 1H), 5.54 (s, 1H), 5.33 (s, 1H), 4.23–4.19 (m, 4H), 3.93 (d, *J* = 12.6 Hz, 1H), 3.57 (d, *J* = 12.6 Hz, 1H), 3.19 (dd, *J* = 4.8 Hz, *J* = 12 Hz, 1H), 2.83 (dd, *J* = 4.8 Hz, *J* = 11.4 Hz, 1H), 2.23 (t, *J* = 2.4 Hz, 1H), 1.12(t, *J* = 7.2 Hz, 3H). ^{13}C NMR (150 MHz, $CDCl_3$) *δ* 169.4, 160.3, 151.6, 148.7, 141.7, 131.4, 79.3, 79.1, 74.1, 71.3, 63.3, 35.6, 35.4, 17.3, 14.0. ESI-HRMS (m/z) calcd $C_{15}H_{16}N_4O_3S_2Na$ $(M + Na)^+$, 387.0556; found,387.0566. HPLC purity: 97.2%.

*1.2.25. (±)Ethyl-3-(6-(butylthio)-9H-purin-9-yl)-4-hydroxytetrahydrothiophene-3-carboxylate (**25a, 25b**)*

Compounds **25a**, and **25b** were prepared as described for the preparation of **1a**, and **1b** except ethyl 2-(6-(butylthio)-9*H*-purin-9-yl) acrylate used in place of ethyl 2-(9*H*-purin-9-yl) acrylate. **25a**: white solid, 45.1% yield. ^{1}H NMR (400 MHz, DMSO) *δ* 8.72 (s, 1H), 8.68 (s, 1H), 5.93 (d, *J* = 5.2 Hz, 1H), 5.23–5.20 (m, 1H), 4.17–4.07 (m, 2H), 4.00 (d, *J* = 11.6 Hz, 1H), 3.62 (d, *J* = 11.2 Hz, 1H), 3.41-3.30 (m, 3H), 2.90 (dd, *J* = 3.6 Hz, *J* = 11.2 Hz, 1H), 1.73-1.66 (m, 2H), 1.49-1.39 (m, 2H), 1.04 (t, *J* = 7.2 Hz, 3H) , 0.91 (t, *J* =

7.2 Hz, 3H).^{13}C NMR (100 MHz, DMSO) *δ* 167.9, 159.8, 151.3, 148.6, 143.8, 130.5, 73.9, 73.8, 62.0, 35.5, 33.7, 31.2, 27.5, 21.4, 13.6, 13.5.ESI-HRMS (*m/z*) calcd $C_{16}H_{22}N_4O_3S_2$ (M + Na)$^+$, 405.1026; found, 405.1020. HPLC purity: 99.6%. **25b**: white solid, 30.2% yield.^{1}H NMR (400 MHz, $CDCl_3$) *δ* 8.62 (s, 1H), 8.21 (s, 1H), 5.74(s, 1H), 5.33-5.30 (m, 1H), 4.22-4.15 (m, 2H), 3.91 (d, *J* = 12 Hz, 1H), 3.56 (d, *J* = 12.4 Hz, 1H), 3.40–3.35 (m, 1H), 3.18 (q, *J* = 4.8 Hz, 1H), 2.87 (q = 3.2, 1H), 1.80-1.72 (m, 2H), 1.54–1.45 (m, 2H), 1.10 (t, *J* = 7.2 Hz, 3H) , 0.94 (t, *J* = 7.6 Hz, 3H).^{13}C NMR (100 MHz, $CDCl_3$) *δ* 169.3, 162.9, 151.5, 148.3, 141.2, 131.4, 79.1, 74.0, 63.0, 35.6, 35.3, 31.5, 28.6, 22.1, 14.0, 13.7.ESI-HRMS (*m/z*) calcd $C_{16}H_{22}N_4O_3S_2$ (M + Na)$^+$, 405.1026; found, 405.1031. HPLC purity: 98.6%.

*1.2.26. (±)Ethyl-4-hydroxy-3-(6-(pentylthio)-9H-purin-9-yl) tetrahydrothiophene-3-carboxylate (**26a**, **26b**)*

Compounds **26a**, and **26b** were prepared as described for the preparation of **1a**, and **1b** except ethyl 2-(6-(pentylthio)-9*H*-purin-9-yl) acrylate used in place of ethyl 2-(9*H*-purin-9-yl) acrylate. **26a**: white solid, 43.3% yield. ^{1}H NMR (400 MHz, $CDCl_3$) *δ* 8.62 (s, 1H), 8.27 (s, 1H), 5.48 (q, *J* = 6.0 Hz, 1H), 4.80(d, *J* = 6.0 Hz, 1H), 4.21(q, *J* = 7.2 Hz, 2H), 3.78 (d, *J* = 12 Hz, 1H), 3.54 (d, *J* = 12.0 Hz, 1H), 3.38 (t. *J* = 7.2 Hz, 2H), 3.28 (q, *J* = 6.0 Hz, 1H), 2.85 (dd, *J* = 6.4 Hz, *J* = 11.2 Hz, 1H), 1.83-1.75 (m, 2H), 1.51-1.33 (m, 4H), 1.17 (t, *J* = 6.8 Hz, 3H), 0.91 (t, *J* = 7.2 Hz, 3H).^{13}C NMR (100 MHz, $CDCl_3$) *δ* 168.3, 163.1, 151.5, 148.4, 142.2, 131.3, 74.0, 63.2, 35.1, 33.6, 31.1, 29.2, 29.0, 22.4, 14.1, 14.0.ESI-HRMS (*m/z*) calcd $C_{17}H_{25}N_4O_3S_2$ (M + H)$^+$,397.1363; found, 397.1354. HPLC purity: 99.6%. **26b**: white solid, 25.1% yield. ^{1}H NMR (600 MHz, $CDCl_3$) *δ* 8.62 (s, 1H), 8.21 (s, 1H), 5.75 (s, 1H), 5.32-5.30(m, 1H), 4.21–4.14 (m, 2H), 3.91 (d, *J* = 12.0 Hz, 1H), 3.56 (d, *J* = 12.0 Hz, 1H), 3.39-3.32 (m, 2H), 3.17 (dd, *J* = 4.8 Hz, *J* = 11.4 Hz, 1H), 2.87 (dd, *J* = 4.8 Hz, *J* = 11.4 Hz, 1H), 1.79-1.74 (m, 2H), 1.47-1.42 (m, 2H), 1.37-1.31 (m, 2H), 1.09 (t, *J* = 7.2 Hz, 3H), 0.88 (t, *J* = 7.2 Hz, 3H).^{13}C NMR (100 MHz, $CDCl_3$) *δ* 169.1, 162.7, 151.4, 148.2, 141.3, 131.3, 79.0, 74.0, 63.0, 35.5, 35.2, 31.0, 29.0, 28.8, 22.2, 14.0, 14.0.ESI-HRMS (*m/z*) calcd $C_{17}H_{25}N_4O_3S_2$ (M + H)$^+$,397.1363; found, 397.1361. HPLC purity: 95.3%.

1.2.27. (±)Ethy-3-(6-(benzylthio)-9H-purin-9-yl)-4-hydroxytetrahydrothiophene-3-

*carboxylate (**27a, 27b**)*

Compounds **27a**, and **27b** were prepared as described for the preparation of **1a**, and **1b** except ethyl 2-(6-(methylthio)-9*H*-purin-9-yl) acrylate used in place of ethyl 2-(9*H*-purin-9-yl) acrylate. **27a**: white solid, 43.2% yield. ^{1}H NMR (400 MHz, DMSO) *δ*8.73 (s, 1H), 8.68 (s, 1H), 7.44 (d, *J* = 7.2 Hz, 1H), 7.31–7.20 (m, 3H), 6.40 (d, *J* = 5.6 Hz,1H), 5.43 (dd, *J* = 5.6 Hz, *J* = 9.6 Hz, 1H), 4.64 (dd, *J* = 13.6 Hz, *J* = 16 Hz, 2H), 4.12-4.00 (m, *J* = 13.6 Hz 2H), 3.90 (d, *J* = 12.4 Hz, 1H), 3.78 (d, *J* = 12.4 Hz, 1H), 3.26-3.22 (m, 1H), 2.96 (dd, *J* = 4 Hz, *J* = 12 Hz, 1H), 1.00 (t, *J* = 7.2 Hz, 3H).^{3}C NMR (100 MHz, DMSO) *δ* 167.9, 159.0, 151.2, 148.8, 144.0, 137.8, 130.3, 129.0, 128.5, 127.2, 73.9, 62.0, 35.5, 33.7, 31.6, 13.7.ESI-HRMS (*m/z*) calcd $C_{19}H_{20}N_4O_3S_2$ (M + Na)$^+$, 439.0869; found, 439.0872. HPLC purity: 99.7%. **27b**: white solid, 42.4% yield. ^{1}H NMR (400 MHz, DMSO) *δ* 8.75 (s, 1H), 8.74 (s, 1H), 7.47 (d, *J* = 7.2 Hz, 2H), 7.34–7.22 (m, 3H), 5.94 (d, *J* = 5.2 Hz, 1H), 5.24-5.21 (m, 1H), 4.66 (dd, *J* = 13.6 Hz, *J* = 18.4 Hz, 2H), 4.15-4.07 (m, 2H), 4.00 (d, *J* = 11.6 Hz, 1H), 3.62 (d, *J* = 11.2 Hz, 1H), 3.40–3.38 (m, 1H), 2.90 (dd, *J* = 3.2 Hz, *J* = 11.2 Hz, 1H), 1.04 (t, *J* = 6.8 Hz, 3H).^{13}C NMR (100 MHz, DMSO) *δ* 167.3, 159.3, 151.3, 148.7, 143.0, 137.7, 130.5, 129.0, 128.5, 127.2, 76.6, 75.4, 61.8, 35.8, 34.6, 31.6, 13.8. ESI-HRMS (*m/z*) calcd $C_{19}H_{20}N_4O_3S_2$ (M + Na)$^+$, 439.0869; found, 439.0863. HPLC purity: 98.3%.

*1.2.28. (±)Ethyl-3-(6-((3-fluorophenyl) thio)-9H-purin-9-yl)-4-Hydroxytetrahydrothiophene-3-carboxylate (**28a, 28b**)*

Compounds **28a**, and **28b** were prepared as described for the preparation of **1a**, and **1b** except ethyl 2-(6-((3-fluorophenyl) thio)-9*H*-purin-9-yl) acrylate used in place of ethyl 2-(9*H*-purin-9-yl) acrylate. **28a**: white solid, 26.4% yield. ^{1}H NMR (600 MHz, $CDCl_3$) *δ* 8.21 (s, 1H), 7.90 (s, 1H), 6.48 (s, 1H), 5.21(t, *J* = 6 Hz, 1H), 4.93-4.84(m, 1H),4.46 (s, 2H) 4.19-4.13 (m, 4H), 3.83 (d, *J* = 12 Hz, 1H), 3.50 (d, *J* = 12.6 Hz, 1H), 3.18 (q, *J* = 6 Hz, 1H), 2.89 (dd, *J* = 4.8 Hz, *J* = 11.4 Hz, 1H), 1.99-1.90 (m, 4H), 1.10(t, *J* = 7.2 Hz, 3H). ^{13}C NMR (150 MHz, $CDCl_3$) *δ* 169.5, 153.8, 151.8, 150.9, 137.0, 119.7, 88.6, 87.5, 79.4, 73.9, 62.8, 42.3, 35.7, 35.0, 31.4 (J_{C-F} = 19.7), 13.9. ^{19}F NMR (376MHz, $CDCl_3$) *δ* -111.38.ESI-HRMS (*m/z*) calcd $C_{18}H_{17}FN_4O_3S_2$ (M + Na)$^+$, 443.0618; found, 443.0613. HPLC purity: 99.0%. **28b**: white solid, 31.3% yield. ^{1}H NMR (400 MHz, $CDCl_3$) *δ* 8.58 (s, 1H), 8.31 (s,

1H), 7.46-7.40 (m, 3H), 7.21–7.16 (m, 1H), 5.33 (t, *J* = 4.8 Hz, 1H), 4.24-4.18 (m, 2H), 3.94(d, *J* = 12.4 Hz, 1H), 3.58 (d, *J* = 12.4 Hz, 1H) 3.19 (dd, *J* = 4.8 Hz, *J* = 11.6 Hz, 1H), 2.86 (dd, *J* = 4.8 Hz, *J* = 11.6 Hz, 1H), 1.12(t, *J* = 7.2 Hz, 3H).^{13}C NMR (100 MHz, $CDCl_3$) δ 169.4, 164.0, 161.4 (d, $J_{C\text{–}F}$ = 38 Hz), 151.9, 149.0, 142.0, 131.3 (d, $J_{C\text{-}F}$ = 3.4 Hz), 130.9, 130.7 (d, $J_{C\text{-}F}$ = 8.1 Hz), 128.8 (d, $J_{C\text{–}F}$ = 8.0 Hz), 122.8, 122.6, 117.2, 117.0, 79.1, 74.1, 63.3, 35.6, 35.4, 14.0.^{19}F NMR (376MHz, $CDCl_3$) δ -111.35. ESI-HRMS (*m/z*) calcd $C_{18}H_{18}FN_4O_3S_2$ (M + H)$^+$, 421.0799; found, 421.0790. HPLC purity: 95.7%.

*1.2.29. (±)Ethyl-3-(6-((4-(tert-butyl) phenyl) thio)-9H-purin-9-yl)-4-hydroxytetrahydrothiophene-3-carboxylate (**29a**, **29b**)*

Compounds **29a**, and **29b** were prepared as described for the preparation of **1a**, and **1b** except ethyl 2-(6-((4-(tert-butyl) phenyl) thio)-9*H*-purin-9-yl) acrylate used in place of ethyl 2-(9*H*-purin-9-yl) acrylate. **29a**: white solid, 33.4% yield. ^{1}H NMR (400 MHz, DMSO) δ 8.80 (s, 1H), 8.55 (s, 1H), 7.57-7.51 (m, 4H), 5.95 (d, *J* = 5.2 Hz, 1H), 5.24–5.20 (m, 1H), 4.15-4.08 (m, 2H), 4.01 (d, *J* = 11.2 Hz, 1H), 3.63 (d, *J* = 11.2 Hz, 1H), 3.38 (dd, *J* = 5.6 Hz, *J* = 11.2 Hz, 1H), 2.91 (dd, *J* = 3.6 Hz, *J* = 11.2 Hz, 1H), 1.33 (s, 9H), 1.04 (t, *J* = 7.2 Hz, 3H).^{13}C NMR (100 MHz, DMSO) δ 167.9, 159.2, 152.3, 151.5, 149.0, 144.4, 135.3, 129.8, 126.4, 123.1, 73.9, 62.0, 35.5, 34.5, 33.7, 31.0, 13.6.ESI-HRMS (m/z) calcd $C_{22}H_{27}N_4O_3S_2$ (M + H)$^+$, 459.1519; found, 459.1515. HPLC purity: 96.4%. **29b**: white solid, 37.4% yield. ^{1}H NMR (400 MHz, DMSO) δ 8.76 (s, 1H), 8.56 (s, 1H), 7.57-7.51 (m, 4H), 6.42 (d, *J* = 5.2 Hz, 1H), 5.49–5.45 (m, 1H), 4.13–4.03 (m, 2H), 3.92 (d, *J* = 12.4 Hz, 1H), 3.80 (d, *J* = 12.0 Hz, 1H), 3.28 (dd, *J* = 5.2 Hz, *J* = 12 Hz, 1H), 2.99 (dd, *J* = 3.6 Hz, *J* = 12.0 Hz, 1H), 1.32 (s, 10H), 1.03 (t, *J* = 6.8 Hz, 3H).^{13}C NMR (100 MHz, DMSO) δ 167.3, 159.5, 152.3, 151.5, 148.9, 143.3, 135.3, 130.0, 126.4, 123.0, 76.6, 75.5, 61.8, 35.8, 34.6, 34.5, 31.0, 13.8. ESI-HRMS (m/z) calcd $C_{22}H_{27}N_4O_3S_2$ (M + H)$^+$, 459.1519; found, 459.1514. HPLC purity: 96.3%.

*1.2.30. (±)Ethyl-3-(6-((3-fluoropropyl) thio)-9H-purin-9-yl)-4-hydroxytetrahydrothiophene-3-carboxylate (**30a**, **30b**)*

Compounds **30a**, and **30b** were prepared as described for the preparation of **1a**, and **1b** except ethyl 2-(6-((3-fluoropropyl) thio)-9*H*-purin-9-yl) acrylate used in place of ethyl 2-(9*H*-purin-9-yl) acrylate. **30a**: white solid, 31.4% yield. ^{1}H NMR (400 MHz, $CDCl_3$)

δ 8.63 (s, 1H), 8.30 (s, 1H), 5.47 (t, J = 6.4 Hz, 1H), 4.71 (s, 1H), 4.67 (t, J = 6 Hz, 1H), 4.55 (t, J = 6.0 Hz, 1H), 4.21 (q, J = 6.8 Hz, 2H) 3.77 (d, J = 11.6 Hz, 1H), 3.56 (d, J = 11.6 Hz, 1H), 3.50 (t, J = 6.8 Hz, 2H), 3.29 (q, J = 5.6 Hz, 1H), 2.84 (dd, J = 6.8 Hz, J = 11.2 Hz, 1H), 2.27-2.14 (m, 2H), 1.17 (t, J = 7.2 Hz, 3H). ^{13}C NMR (100 MHz, $CDCl_3$) δ 168.2, 162.0, 151.5. 148.6 142.4, 131.4, 83.3, 81.6, 74.0, 63.2, 35.1, 33.6, 30.6 (d, $J_{C\text{–}F}$=20Hz), 24.8 (d, $J_{C\text{–}F}$=5.2 Hz), 14.0.^{19}F NMR (376MHz, $CDCl_3$) δ 20.41. ESI-HRMS (m/z) calcd $C_{15}H_{19}FN_4O_3S_2Na$ (M + Na) $^+$, 409.0775; found, 409.0774. HPLC purity: 97.1%. **30b**: white solid, 30.2% yield. ^{1}H NMR (400 MHz, $CDCl_3$) δ 8.65 (s, 1H), 8.24 (s, 1H), 5.66(s, 1H), 5.32 (t, J = 4.8 Hz, 1H), 4.66(t, J = 5.6 Hz, 1H), 4.54 (t, J = 6.0 Hz, 1H), 4.20 (dd, J = 6.4 Hz, J = 13.6 Hz, 2H), 3.92 (d, J = 12.4 Hz, 1H), 3.56 (d, J = 12.0 Hz, 1H), 3.50 (t, J = 6.8 Hz, 2H), 3.18 (dd, J = 11.2 Hz, J = 4.8 Hz, 1H), 2.86 (dd, J = 11.6 Hz, J = 5.2 Hz, 1H), 2.26-2.14 (m, 2H), 1.11 (t, J = 7.2 Hz, 3H).^{13}C NMR (100 MHz, $CDCl_3$) δ 169.3, 161.9, 151.5, 148.5, 141.5, 131.5, 83.3, 81.6, 79.1, 74.1, 63.2, 35.42 (d, $J_{C\text{–}F}$=25.4 Hz), 30.6(d, $J_{C\text{–}F}$=20.1 Hz), 24.8 (d, $J_{C\text{–}F}$=5.1 Hz), 14.0.^{19}F NMR (376MHz, $CDCl_3$)δ -20.45. ESI-HRMS (m/z) calcd $C_{15}H_{19}FN_4O_3S_2Na$ (M + Na)$^+$, 409.0775; found, 409.0768. HPLC purity: 97.7%.

*1.2.31. (±)Ethyl-3-(6-((3-chloropropyl) thio)-9H-purin-9-yl)-4-hydroxytetrahydrothiophene-3-carboxylate (**31a, 31b**)*

Compounds **31a**, and **31b** were prepared as described for the preparation of **1a**, and **1b** except ethyl 2-(6-((3-chloropropyl) thio)-9*H*-purin-9-yl) acrylate used in place of ethyl 2-(9*H*-purin-9-yl) acrylate. **31a**: white solid, 42.4% yield. ^{1}H NMR (400 MHz, $CDCl_3$) δ 8.59 (s, 1H), 8.31 (s, 1H), 5.44 (s, 1H), 5.01(s, 1H), 4.20-4.13 (m, 2H), 3.75-3.63 (m, 4H), 3.47 (t, J = 6.8 Hz, 2H), 3.29 (q, J = 5.6 Hz, 1H), 2.85 (dd, J = 6.4 Hz, J = 11.2 Hz, 1H), 2.26-2.20 (m, 2H), 1.13 (t, J = 7.2 Hz, 3H). ^{13}C NMR (100 MHz, $CDCl_3$) δ 168.1, 161.4, 151.5, 148.6, 142.5, 131.0, 73.9, 63.1, 43.5, 34.8, 33.9, 32.2, 26.0, 13.9. ESI-HRMS (m/z) calcd $C_{15}H_{19}ClN_4O_3S_2Na$ (M + Na)$^+$, 425.0479; found, 425.0470. HPLC purity: 96.4%. **31b**: white solid, 21.7% yield. ^{1}H NMR (400 MHz, $CDCl_3$) δ 8.62 (s, 1H), 8.24 (s, 1H), 5.67 (s, 1H), 5.31(s, 1H), 4.176 (q, J = 7.2 Hz, 2H), 3.92 (d, J = 12 Hz, 1H), 3.68 (t, J = 6.4 Hz, 2H), 3.56 (d, J = 12.4 Hz, 1H), 3.50 (t, J = 6.8 Hz, 2H), 3.16 (dd, J = 5.2 Hz, J = 11.6 Hz, 1H), 2.86 (dd, J = 4.8 Hz, J = 11.6 Hz, 1H), 2.28-2.21 (m, 2H), 1.094 (t, J = 7.2 Hz, 3H).^{13}C NMR

(100 MHz, $CDCl_3$) δ 169.2, 161.7, 151.4, 148.4, 141.5, 131.4, 79.0, 74.1, 63.1, 43.5, 35.5, 35.3, 32.2, 26.0, 13.9. ESI-HRMS (m/z) calcd $C_{15}H_{19}ClN_4O_3S_2Na$ (M + Na)$^+$, 425.0479; found, 425.0474. HPLC purity: 95.2%.

*1.2.32. (±)Ethyl-3-(2-amino-6-(piperidin-1-yl)-9H-purin-9-yl)-4-hydroxytetrahydrothiophene-3-carboxylate (**32a, 32b**)*

Compounds **32a**, and **32b** were prepared as described for the preparation of **1a**, and **1b** except ethyl 2-(2-chloro-6-(piperidin-1-yl)-9*H*-purin-9-yl) acrylate was used in place of ethyl 2-(9*H*-purin-9-yl) acrylate. **32a**: yellow solid, 43.0% yield. ^{1}H NMR (600 MHz, $CDCl_3$) δ 7.60 (s, 1H), 5.18 (t, J = 5.4 Hz, 1H), 4.60 (s, 2H), 4.27–4.16 (m, 4H), 3.80 (d, J = 12 Hz, 1H), 3.49 (d, J = 12 Hz, 1H), 3.24 (dd, J = 6.6 Hz, J = 11.4 Hz, 1H), 2.97 (q, J = 5.4 Hz, 1H), 1.71-1.64(m, 6H), 1.18 (t, J = 7.2 Hz, 3H).^{13}C NMR (150 MHz, $CDCl_3$) δ 169.9, 152.8, 134.5, 114.7, 79.5, 73.6, 62.7, 58.6, 36.0, 34.9, 29.8, 26.3, 24.9, 18.6, 14.1. ESI-HRMS (*m/z*) calcd $C_{17}H_{25}N_6O_3S$ (M + H)$^+$, 393.1703; found, 393.1701. HPLC purity: 99.0%.**32b**: yellow solid, 32.6% yield.^{1}H NMR (600 MHz, $CDCl_3$) δ 7.72 (s, 1H), 5.34 (t, J = 6.6 Hz, 1H), 4.59 (s, 2H), 4.26–4.11 (m, 4H), 3.68 (d, J = 12 Hz, 1H) 3.37 (d, J = 12 Hz, 1H), 3.17 (dd, J = 6 Hz, J = 10.8 Hz, 1H), 2.81 (dd, J = 7.2 Hz, J = 10.8 Hz, 1H), 1.72–1.65 (m, 6H), 1.20(t, J = 7.2 Hz, 3H).^{13}C NMR (150 MHz, $CDCl_3$) δ 168.8, 158.5, 154.5, 152.4, 135.1, 114.7, 73.5, 62.8, 35.2, 33.4, 26.3, 24.9, 14.1. ESI-HRMS (*m/z*) calcd $C_{17}H_{25}N_6O_3S$ (M + H)$^+$, 393.1703; found, 393.1701. HPLC purity: 98.2%.

*1.2.33. (±)Ethyl-3-(2-fluoro-6-(piperidin-1-yl)-9H-purin-9-yl)-4-hydroxytetrahydrothiophene-3-carboxylate (**33a, 33b**)*

Compounds **33a**, and **33b** were prepared as described for the preparation of **1a**, and **1b** except ethyl 2-(2-fluoro-6-(piperidin-1-yl)-9*H*-purin-9-yl) acrylate was used in place of ethyl 2-(9*H*-purin-9-yl) acrylate. **33a**: yellow solid, 36.4% yield. ^{1}H NMR (600 MHz, $CDCl_3$) δ 8.01 (s, 1H), 5.44 (s, 1H),4.35 (s, 2H), 4.23 (q, J=7.2,2H), 3.94-3.68(m, 3H), 3.47 （d, J =11.6,1H), 3.26 (dd, J = 6, J =11.2, 1H), 2.84 (dd, J = 6.4, J =11.2, 1H), 1.72 (s, 6H), 1.21 (t, J = 7.6.3H) ^{13}C NMR (100 MHz, $CDCl_3$) δ 168.3, 159.6, 157.5, 154. 9(d, J_{C-F} = 19.6), 152.4(d, J_{C-F} = 11.5), 137.5 (d, $J_{C–F}$=2.9), 117. 8 (d, $J_{C–F}$ = 4.3), 76.3, 73.6, 63.1, 58.6, 35.2, 33.9, 26.2, 24.5, 18.5, 14.0. ^{19}F NMR (376MHz, $CDCl_3$) δ -50.27. ESI-HRMS (*m/z*) calcd $C_{17}H_{23}FN_5O_3S$ (M + H)$^+$, 396.1500; found, 396.1499. HPLC purity: 99.4%. **33b**: yellow

solid, 43.6% yield. ^{1}H NMR (600 MHz, $CDCl_3$) δ 7.96 (s, 1H), 5.38 (s, 1H), 5.26–5.24 (m, 1H), 4.60–4.45 (m, 2H), 4.29–4.19 (m, 2H), 3.97 (s, 1H), 3.86 (d, *J* = 12,2H), 3.49 (d, *J* = 12.6, 1H), 3.13 (dd, *J* = 4.8, *J* = 12, 1H), 3.83（dd, *J* = 3.6, *J* = 11.4, 1H), 1.16（t, *J* = 7.2, 3H). ^{13}C NMR (150 MHz, $CDCl_3$) δ 169.7, 159.1, 157.7, 154.9 ($J_{C–F}$ = 19.8), 152.3 (J_{C-F} = 18.6), 136.6 ($J_{C–F}$=3), 118.0 ($J_{C–F}$ = 4.4), 78.9, 7 3.6, 63.1, 35.6 ($J_{C–F}$ = 6.5)， 24.7, 14.0.. ^{19}F NMR (376MHz, $CDCl_3$) δ -50.14. ESI-HRMS (*m/z*) calcd $C_{17}H_{22}FN_5O_3S$ (M + Na)$^+$, 418.1320; found, 418.1322. HPLC purity: 96.6%.

*1.2.34. (±)Ethyl-3-(2-chloro-6-(piperidin-1-yl)-9H-purin-9-yl)-4-hydroxytetrahydrothiophene-3-carboxylate (**34a**, **34b**)*

Compounds **34a**, and **34b** were prepared as described for the preparation of **1a**, and **1b** except ethyl 2-(2-chloro-6-(piperidin-1-yl)-9*H*-purin-9-yl) acrylate was used in place of ethyl 2-(9*H*-purin-9-yl) acrylate. **34a**: white solid, 17.6% yield. ^{1}H NMR (600 MHz, $CDCl_3$) δ 8.01 (s, 1H), 5.41 (t, *J* = 6 Hz, 1H), 4.62 (s, 2H), 4.29-4.20 (m, 4H), 3.69 (d, *J* = 12 Hz, 1H), 3.45 (d, *J* = 11.4 Hz, 1H), 3.25 (q, *J* = 5.4 Hz, 1H), 2.82 (dd, *J* = 7.2 Hz, *J* = 11.4 Hz, 1H), 1.74-1.68 (m, 6H), 1.22 (t, *J* = 7.2 Hz, 3H).^{13}C NMR (100 MHz, $CDCl_3$) δ 168.4, 154.0, 153.7, 152.1, 137.6, 118.4, 73.6, 63.2, 35.3, 33.7, 29.8, 26.3, 24.7, 14.0. ESI-HRMS (*m/z*) calcd $C_{17}H_{23}ClN_5O_3S$ (M + H)$^+$, 412.1205; found, 412.1210. HPLC purity: 96.1%. **34b**: white solid, 21.6% yield ^{1}H NMR (400 MHz, $CDCl_3$) δ 7.93 (s, 1H), 5.65(s, 1H),5.24 (t, *J* = 4.8 Hz, 1H), 4.31–4.16 (m, 6H), 3.86 (d, *J* = 12,6 Hz, 1H), 3.48 (d, *J* = 12 Hz, 1H), 3.16 (dd, *J* = 4.8 Hz, *J* = 11.4 Hz, 1H), 2.86 (dd, *J* = 4.8 Hz, *J* = 11.4 Hz, 1H), 1.73-1.67 (m, 6H), 1.18(t, *J* = 7.2 Hz, 3H).^{13}C NMR (100 MHz, $CDCl_3$) δ 169.6, 154.0, 153.6, 152.0, 136.8, 118.6, 79.2, 73.7, 63.1, 35.7, 35.4, 26.2, 24.7, 14.0.ESI-HRMS (*m/z*) calcd $C_{17}H_{23}ClN_5O_3S$ (M + H)$^+$, 412.1205; found, 412.1206. HPLC purity: 98.7%.

*1.2.35. (±)Ethyl-3-(2-amino-6-(propylthio)-9H-purin-9-yl)-4-hydroxytetrahydrothiophene-3-carboxylate (**35a**, **35b**)*

Compounds **35a**, and **35b** were prepared as described for the preparation of **1a**, and **1b** except ethyl 2-(2-amino-6-(propylthio)-9*H*-purin-9-yl) acrylate used in place of ethyl 2-(9*H*-purin-9-yl) acrylate. **35a**: white solid, 41.3% yield. ^{1}H NMR (400 MHz, $CDCl_3$) δ 7.90 (s, 1H), 5.42 (t, *J* = 5.6 Hz, 1H), 4.96 (s, 2H), 4.23–4.16 (m, 2H), 3.71 (d, *J* = 11.6 Hz, 1H), 3.44 (d, *J* = 11.2 Hz, 1H), 3.27–3.21 (m, 3H), 2.86 (dd, *J* = 6.0 Hz, *J* = 10.8 Hz, 1H),

1.82-1.73 (m, 2H), 1.19 (t, J = 7.2 Hz, 3H), 1.05(t, J = 7.2 Hz, 3H). ^{13}C NMR (100 MHz, $CDCl_3$) δ 168.5, 163.8, 158.3, 150.0, 139.3, 125.5, 76.7, 73.8, 63.0, 35.1, 33.7, 30.7, 23.0, 14.0, 13.6. ESI-HRMS (*m/z*) calcd $C_{15}H_{22}N_5O_3S_2$ (M + H)$^+$,384.1159; found, 384.1150. HPLC purity: 99.7%. **35b**: white solid, 43.5% yield. ^{1}H NMR (400 MHz, $CDCl_3$) δ 7.84 (s, 1H), 5.98 (s, 1H), 5.24 (t, J = 5.2 Hz, 1H), 4.95 (s, 2H), 4.27–4.14 (m, 2H), 3.82 (d, J = 12 Hz, 1H), 3.50 (d, J = 12.0 Hz, 1H), 3.28–3.24 (m, 2H), 3.17 (q, J = 5.6 Hz, 1H), 2.90 (dd, J = 4.8 Hz, J = 11.2 Hz, 1H), 1.82–1.73 (m, 2H), 1.15 (t, J = 7.2 Hz, 3H), 1.05 (t, J = 7.2 Hz, 3H).^{13}C NMR (100 MHz, $CDCl_3$) δ 169.7, 163.4, 158.2, 150.3, 138.4, 125.6, 79.0, 73.6, 63.0, 35.6, 35.1, 30.6, 23.0, 14.0, 13.6. ESI-HRMS (*m/z*) calcd $C_{15}H_{22}N_5O_3S_2$ (M + H)$^+$, 384.1159; found, 384.1149. HPLC purity: 98.4%.

*1.2.36. (±)Ethyl-3-(2-fluoro-6-(propylthio)-9H-purin-9-yl)-4-hydroxytetrahydrothiophene-3-carboxylate (**36a**, **36b**)*

Compounds **36a**, and **36b** were prepared as described for the preparation of **1a**, and **1b** except ethyl 2-(2-fluoro-6-(propylthio)-9*H*-purin-9-yl) acrylate used in place of ethyl 2-(9*H*-purin-9-yl) acrylate. **36a**: white solid, 38.5% yield. ^{1}H NMR (400 MHz, $CDCl_3$) δ 8.31, (s, 1H) 5.44, (t, J = 6 Hz, 1H), 4.24 (q, J = 13.6 Hz, 2H), 3.72(d, J = 11.6 Hz, 1H), 3.61(d, J = 11.6 Hz, 1H), 3.34-3.29 (m, 2H), 2.85 (dd, J = 11.2, J = 6 Hz, 1H), 1.86–1.77 (m, 2H), 1.19 (t, J = 6.8 Hz, 3H) 1.08 (t, J = 7.6 Hz, 3H).^{13}C NMR (100 MHz, $CDCl_3$) δ 169.3, 166.0 (d, $J_{C\text{–}F}$ = 16.5 Hz), 159.7, 158.3, 152.2 (d, $J_{C\text{–}F}$ = 17.6 Hz), 145.2 (d, $J_{C\text{–}F}$ = 3.2 Hz), 76.4, 75.7, 36.0, 31.9, 23.9, 14.1, 13.6.^{19}F NMR (376MHz, $CDCl_3$) δ -49.78. ESI-HRMS (*m/z*) calcd $C_{15}H_{19}FN_4O_3S_2Na$ (M + Na)$^+$, 409.0775; found, 409.0775. HPLC purity: 99.4%. **36b**: white solid, 41.5% yield. ^{1}H NMR (600 MHz, $CDCl_3$) δ 8.27 (s, 1H), 5.29 (t, J = 4.2 Hz, 1H), 4.25–4.19 (m, 2H) 3.92 (d, J = 12.6 Hz, 1H) 3.54 (d, J = 12.6 Hz, 1H), 3.36–3.29 (m, 2H), 3.11 (dd, J = 4.2 Hz, J = 12 Hz, 1H), 3.81 (dd, J = 4.8 Hz, J = 12 Hz, 1H), 1.84–1.78(m, 2H), 1.14 (t, J = 7.2 Hz, 3H), 1.07 (t, J = 7.2 Hz, 3H).^{13}C NMR (100 MHz, $CDCl_3$) δ 169.1, 166.1 ($J_{C\text{–}F}$ = 16.5 Hz), 157.6 (d, $J_{C\text{–}F}$ = 215.0 Hz), 150.1 ($J_{C\text{–}F}$ = 17.3 Hz), 141.4 ($J_{C\text{–}F}$ = 3.2 Hz), 129.8 ($J_{C\text{–}F}$ = 4.2 Hz), 78.5, 73.8, 63.4, 35.4 ($J_{C\text{–}F}$ = 45 Hz), 31.3, 22.7, 14.0, 13.5.^{19}F NMR (376 MHz, $CDCl_3$) δ -49.51. ESI-HRMS (m/z) calcd $C_{15}H_{19}FN_4O_3S_2Na$ (M + Na)$^+$, 409.0775; found, 409.0769. HPLC purity: 99.4%.

1.2.37. (±)Ethyl-3-(2-chloro-6-(propylthio)-9H-purin-9-yl)-4-

*hydroxytetrahydrothiophene-3-carboxylate (**37a**, **37b**)*

Compounds **37a**, and **37b** were prepared as described for the preparation of **1a**, and **1b** except ethyl 2-(2-chloro-6-(propylthio)-9*H*-purin-9-yl) acrylate used in place of ethyl 2-(9*H*-purin-9-yl) acrylate. **37a**: white solid, 22.4% yield. ^{1}H NMR (400 MHz, DMSO) *δ* 7.57 (s, 1H), 5.90 (d, *J* = 5 Hz, 2H), 5.17-5.14 (m, 1H), 4.14 (q, *J* = 7.2 Hz, 2H), 3.92 (*J* = 11.2 Hz, 1H), 3.57 (d, *J* = 11.2 Hz, 1H), 3.37 (q, *J* = 5.6 Hz, 1H), 3.33-3.29 (m, 2H), 2.88 (dd, *J* = 3.6 Hz, 11.6, 1H), 1.78-1.69 (m, 2H), 1.07 (t, *J* = 7.2 Hz, 3H), 1.00 (t, *J* = 7.2 Hz, 3H). ^{13}C NMR (100 MHz, DMSO) *δ* 167.6, 162.4, 151.8, 150.0, 144.6, 130.0, 74.0, 73.6, 62.1, 22.2,13.6, 13.1.ESI-HRMS (*m/z*) calcd $C_{15}H_{19}ClN_4O_3S_2Na$ (M + Na)$^+$, 425.0479; found, 425.0471. HPLC purity: 99.8%. **37b**: white solid, 51.6% yield. ^{1}H NMR (400 MHz, $CDCl_3$) *δ* 8.25 (s, 1H), 5.30-5.27 (m, 1H), 5.12–5.11 (m, 1H), 4.28–4.12 (m, 2H), 3.91 (d, *J* = 12.4 Hz, 1H), 3.53 (d, *J* = 12.4 Hz, 1H), 3.37-3.27 (m, 2H), 3.12 (dd, *J* = 4.4 Hz, *J* = 11.6 Hz, 1H), 2.85–2.80 (m, 1H), 1.84-1.75 (m, 1H), 1.13 (q, *J* = 7.2 Hz, 3H), 1.05 (t, *J* = 7.2 Hz, 3H). ^{13}C NMR (100 MHz, $CDCl_3$) *δ* 168.9, 164.7, 153.3, 149.6, 141.5, 130.2, 78.6, 73.9, 63.3, 35.4 ($J_{C–F}$ = 13.7 Hz), 31.2, 22.6, 13.9, 13.4. ESI-HRMS (m/z) calcd $C_{15}H_{19}ClN_4O_3S_2Na$ (M + Na)$^+$, 425.0479; found, 425.0478. HPLC purity: 97.9%.

1.2.38. (±)*Ethyl-3-(2-amino-6-chloro-9H-purin-9-yl)-4-hydroxytetrahydrothiophene-3-carboxylate (**38a**, **38b**)*

Compounds 3**8a**, and **38b** were prepared as described for the preparation of **1a**, and **1b** except ethyl 2-(2-amino-6-chloro-9H-purin-9-yl) acrylate was used in place of ethyl 2-(9*H*-purin-9-yl) acrylate. 3**8a**: white solid, 71.0% yield. ^{1}H NMR (600 MHz, MeOD) *δ* 8.31 (s, 1H), 5.35 (t, *J* = 5.4 Hz, 1H), 4.23-4.15 (m, 2H), 4.02 (d, *J* = 12.6 Hz, 1H), 3.73 (d, *J* = 12.6 Hz, 1H), 3.33 (s, 1H), 3.21 (q, *J* = 5.4 Hz, 1H), 3.10 (dd, *J* = 4.8 Hz, *J* = 11.4 Hz, 1H), 1.13 (t, *J* = 6.6 Hz, 3H). ^{13}C NMR (150 MHz, $CDCl_3$) *δ* 69.5, 158.5, 153.4, 152.3, 140.4, 125.3, 78.4, 73.5, 63.1, 35.5, 35.3, 13.9. ESI-HRMS (*m/z*) calcd $C_{12}H_{14}ClN_5O_3SNa$ (M + Na)$^+$, 366.0398; found, 366.0399. HPLC purity: 97.1%. 3**8b**: white solid, 16.7% yield. ^{1}H NMR (600 MHz, MeOD) *δ* 8.43 (s, 1H), 5.33 (t, *J* = 5.4 Hz, 1H), 4.23-4.19 (m, 2H),4.01 (d, *J* = 11.4 Hz, 1H), 3.67 (d, *J* = 11.4 Hz, 1H), 3.43 (q, *J* = 6.0 Hz, 1H), 2.94 (dd, *J* = 4.2 Hz, *J* = 10.8 Hz, 1H), 1.16 (t, *J* = 6.6 Hz, 3H). ^{13}C NMR (100 MHz, DMSO) *δ* 168.2, 159.5, 154.4, 149.7, 142.4, 123.2, 74.1, 73.7, 62.1, 35.5, 33.6, 13.8. ESI-HRMS (*m/z*)

calcd $C_{12}H_{14}ClN_5O_3SNa$ (M + Na)$^+$, 366.0398; found, 366.0398. HPLC purity: 98.8%.

*1.2.39. (±)Ethy-3-(6-chloro-2-fluoro-9H-purin-9-yl)-4-hydroxytetrahydrothiophene-3-carboxylate (**39a, 39b**)*

Compounds **39a**, and **39b** were prepared as described for the preparation of **1a**, and **1b** except ethyl 2-(6-chloro-2-fluoro-9*H*-purin-9-yl) acrylate was used in place of ethyl 2-(9*H*-purin-9-yl) acrylate.; **39a**: brown solid, 21.5% yield. ^{1}H NMR (600 MHz, $CDCl_3$) δ 8.61 (s, 1H), 5.40 (t, J = 6.6 Hz, 1H), 4.28–4.21 (m, 2H), 3.72(dd, J = 12.0 Hz, J = 29.4 Hz, 2H), 3.59 (s, 1H), 3.32 (q, J = 6.0 Hz, 1H), 2.82 (dd, J = 11.4 Hz, J = 6.6 Hz, 1H), 1.18 (t, J = 7.2 Hz, 3H).^{13}C NMR (100 MHz, $CDCl_3$) δ 167.6, 145.2, 76.6, 73.8, 63.6,5 3.6, 34.7, 33.7 (dd, J=100.6), 14.0. ^{19}F NMR (376 MHz, $CDCl_3$) δ -48.89. ESI-HRMS (*m/z*) calcd $C_{12}H_{12}ClFN_4O_3SNa$ (M + Na)$^+$, 369.0195; found, 369.0187. HPLC purity: 97.5%. **39b**: brown solid, 32.5% yield. ^{1}H NMR (400 MHz, $CDCl_3$) δ 8.51 (s, 1H), 5.30 (s, 2H), 4.55 (s, 1H), 4.29–4.21 (m, 2H), 3.97 (d, J = 12.8 Hz, 1H), 3.56 (d, J = 12.4 Hz, 1H), 3.12 (dd, J = 3.2 Hz, J = 11.6 Hz, 1H), 2.78 (dd, J = 4.4 Hz, J = 11.6 Hz, 1H), 1.14 (t, J =7.2 Hz,3H).^{13}C NMR (100 MHz, $CDCl_3$) δ 168.7, 158.0, 155.8, 153.8, 144.2 (d, $J_{C\text{-}F}$=3.6), 130.6, 78.3, 74.1, 63.8, 53.6, 35.6, 35.1, 14.0. ^{19}F NMR (376 MHz, $CDCl_3$) δ -48.89. ESI-HRMS (*m/z*) calcd $C_{12}H_{12}ClFN_4O_3SNa$ (M + Na)$^+$, 369.0195; found, 369.0192. HPLC purity: 99.0%.

*1.2.40. (±)Ethyl-3-(2,6-dichloro-9H-purin-9-yl)-4-hydroxytetrahydrothiophene-3-carboxylate (**40a, 40b**)*

Compounds **40a**, and **40b** were prepared as described for the preparation of **1a**, and **1b** except ethyl 2-(2,6-dichloro-9*H*-purin-9-yl) acrylate was used in place of ethyl 2-(9*H*-purin-9-yl) acrylate. **40a**: white solid, 22.6% yield. ^{1}H NMR (600 MHz, $CDCl_3$) δ ^{1}H NMR (600 MHz, $CDCl_3$) δ 8.61 (s, 1H), 5.40 (q, J = 6 Hz, 1 H),4.25 (q, J =7.2 Hz,2H), 3.71(dd, J =12.0 Hz, J= 31.2 Hz, 1H), 3.58 (d, J = 4.8 Hz, 1H), 3.32 (q, J = 6.0 Hz, 1H), 2.81(q, J = 6.6 Hz, J =11.4 Hz, 1H), 1.19 (t, J =7.2 Hz, 3H).^{13}C NMR (100 MHz, $CDCl_3$) δ 167.6, 153.6, 152.8, 152.2, 145.4, 130.5, 76.5, 73.9, 63.6, 34.6, 33.7, 14.0. ESI-HRMS (m/z) calcd $C_{12}H_{12}N_4ClO_3SNa$ (M + Na)$^+$, 384.9905; found, 384.9909. HPLC purity: 99.0%. **40b**: white solid, 35.6% yield.^{1}H NMR (600 MHz, $CDCl_3$) δ 8.51 (s, 1H), 5.30 (q, J = 3.0 Hz, 1H), 4.65 (q, J =1.8 Hz, 1H), 4.32–4.19 (m, 2H), 3.9 6(d, J = 12.6 Hz, 1H), 3.55 (d, J = 12.6 Hz, 1H), 3.13 (dd, J = 3.0, J =12.0 Hz, 1H), 2.78(dd, J = 4.2 Hz, J = 12.0

Hz, 1H), 1.15 (t, *J* = 7.2 Hz,3H).^{13}C NMR (100 MHz, $CDCl_3$) *δ* 168.6, 163.1, 151.6, 148.3, 141.2, 131.5, 79.3, 74.1, 53.7, 35.7, 35.2, 30.9, 22.9, 13.6. ESI-HRMS (m/z) calcd $C_{12}H_{12}N_4ClO_3SNa$ (M + Na)$^+$, 384.9905; found, 384.9899. HPLC purity: 96.0%.

*1.2.41. (±)Ethyl-3-(2-chloro-9H-purin-9-yl)-4-hydroxytetrahydrothiophene-3-carboxylate (**41a, 41b**)*

Compounds **41a**, and **41b** were prepared as described for the preparation of **1a**, and **1b** except ethyl 2-(2-chloro-9*H*-purin-9-yl) acrylate was used in place of ethyl 2-(9*H*-purin-9-yl) acrylate. **41a**: white solid, 54.6% yield. ^{1}H NMR (600 MHz, $CDCl_3$) *δ* 8.96 (s, 1H), 8.56 (s, 1H), 5.44 (t, *J* =6.0 Hz, 1H), 4.29–4.21 (m, 2H), 3.71(dd, *J* = 12.0 Hz, *J* = 43.8 Hz, 2H), 3.33 (q, *J* = 6.0 Hz, 1H), 2.83 (dd, *J* = 6.6 Hz, *J* =11.4 Hz, 1H), 1.18 (t, *J* =7.2 Hz, 3H).^{13}C NMR (150 MHz, $CDCl_3$) *δ* 167.9, 154.2, 153.7, 150.6, 145.5, 132.9, 76.4, 73.6, 63.5, 34.8, 33.8, 14.0; ESI-HRMS (*m/z*) calcd $C_{12}H_{13}N_4ClO_3SNa$ (M + Na)$^+$, 351.0289; found, 351.0295. HPLC purity: 99.5%. **41b**: white solid, 27.1% yield. ^{1}H NMR (600 MHz, $CDCl_3$) *δ* 8.99 (s, 1H), 8.48 (s, 1H), 5.34 (t, *J* = 4.4 Hz, 1H), 4.89 (s, 1H), 4.31–4.14 (m, 2H),3.96 (d, *J* = 12.4 Hz, 1H), 3.56 (d, *J* = 12.4 Hz, 1H), 3.14 (dd, *J* = 3.6 Hz, *J* = 11.6 Hz, 1H), 2.81 (dd, *J* = 4.8 Hz, *J* = 11.6 Hz, Hz, 1H), 1.12 (t, *J* = 7.2 Hz, 3H).^{13}C NMR (100 MHz, $CDCl_3$) *δ* 168.9, 154.3, 153.2, 150.8, 144.5, 133.3, 78.4, 73.9, 63.5, 35.6, 35.2, 13.9; ESI-HRMS (*m/z*) calcd $C_{12}H_{13}N_4ClO_3SNa$ (M + Na)$^+$, 351.0289; found, 351.0290. HPLC purity: 99.7%.

*1.2.42. (±)Ethy-3-(2-chloro-6-morpholino-9H-purin-9-yl)-4-hydroxytetrahydrothiophene-3-carboxylate (**42a, 42b**)*

Compounds **42a**, and **42b** were prepared as described for the preparation of **1a**, and **1b** except ethyl 2-(2-chloro-6-morpholino-9*H*-purin-9-yl) acrylate was used in place of ethyl 2-(9*H*-purin-9-yl) acrylate. **42a**: white solid, 18.4% yield. ^{1}H NMR (400 MHz, $CDCl_3$) *δ* 8.06 (s, 1H), 5.42 (q, *J* = 6 Hz, 1H), 4.33 (d *J* = 5.2 Hz, 3H), 4.34-4.19 (m, 6H), 3.82 (t, *J* = 5.2 Hz, 4H), 3.70 (d, *J* = 12.0 Hz, 1H), 3.46 (d, *J* = 11.6 Hz, 1H) 3.26 (dd, *J* = 6.0 Hz, *J* = 10.8 Hz, 1H),2.81 (dd, *J* = 6.8 Hz, *J* = 11.2 Hz, 1H), 1.23 (t, *J* = 6.8 Hz, 3H).^{13}C NMR (100 MHz, $CDCl_3$) *δ* 168.3, 154.2, 153.7, 152.4, 138.1, 118.6, 76.6, 73.6, 67.1, 63.3, 35.3, 33.7, 14.0.ESI-HRMS (*m/z*) calcd $C_{16}H_{20}ClN_5O_4SNa$ (M + Na)$^+$, 436.0817; found, 436.0824. HPLC purity: 97.6%. **42b**: white solid, 53.2% yield. ^{1}H NMR (400 MHz, $CDCl_3$)

δ 7.98 (s, 1H), 5.49 (s, 1H), 5.26 (t, *J* = 4.4 Hz, 1H), 4.32-4.17 (m, 6H), 3.88 (d, *J* = 12.4 Hz, 1H), 3.83 (t, *J* = 4.8 Hz, 4H), 3.49 (d, *J* = 12.4 Hz, 1H), 3.17 (dd, *J* = 4.8Hz, *J* = 11.6 Hz, 1H), 2.85 (dd, *J* = 4.4 Hz, *J* = 11.6 Hz, 1H) 1.18 (t, *J* = 7.2 Hz, 3H). ^{13}C NMR (100 MHz, $CDCl_3$) δ 169.5, 154.1, 153.6, 152.2, 137.4, 118.8, 79.1, 73.8, 67.1, 63.3, 35.7, 35.5, 14.1.ESI-HRMS (*m/z*) calcd $C_{16}H_{20}ClN_5O_4SNa$ (M + Na)$^+$, 436.0817; found, 436.0817. HPLC purity: 96.9%.

1.3. Copies of ^{1}H NMR, ^{13}C NMR and HR-ESIMS spectra of target compounds

(±)Ethyl-4-hydroxy-3-(9*H*-purin-9-yl)tetrahydrothiophene-3-carboxylate (**1a**)

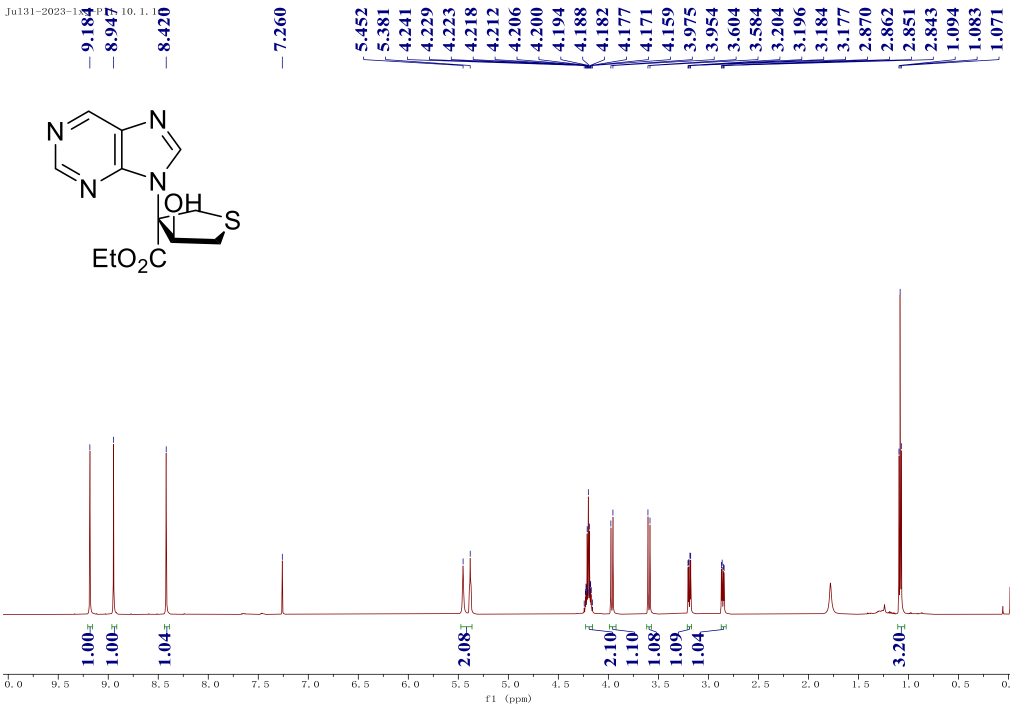

^{1}H NMR spectrum

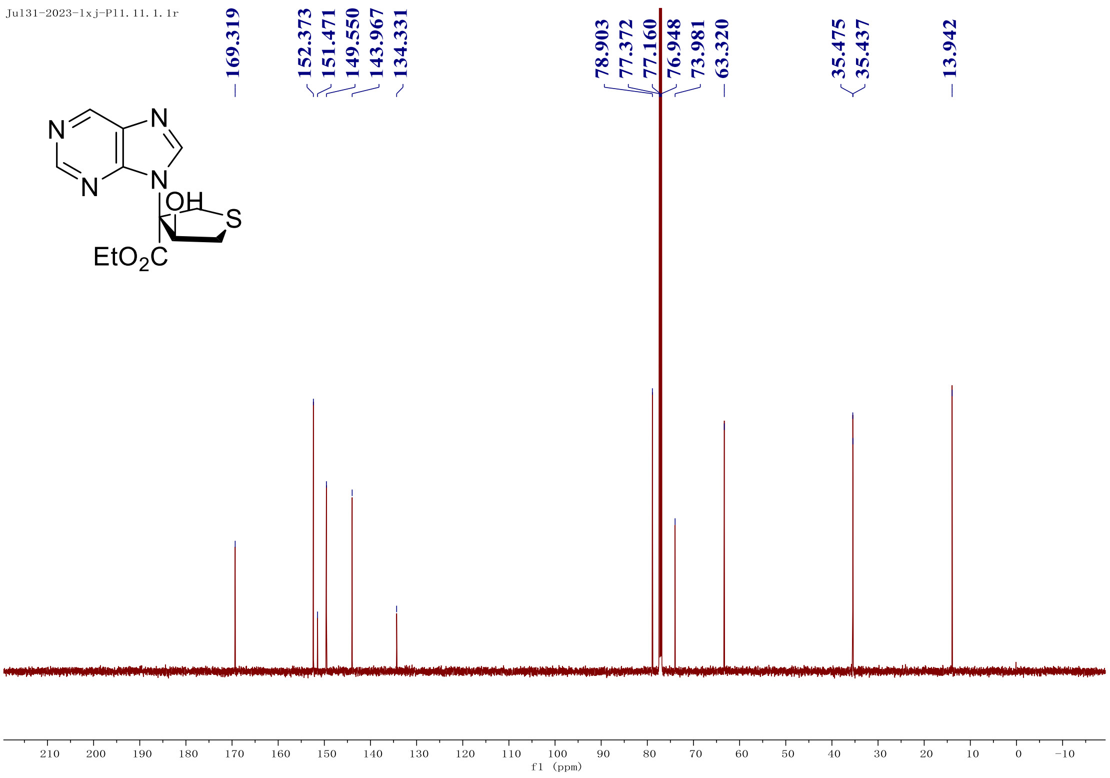

^{13}C NMR spectrum

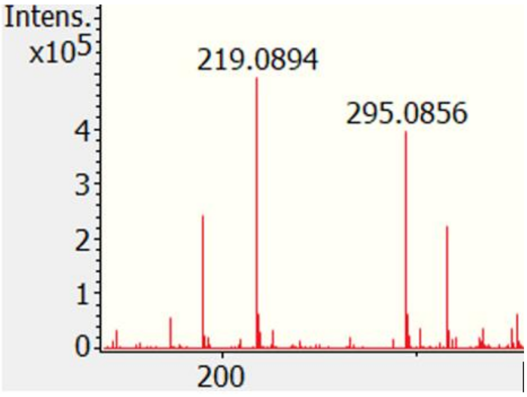

HR-ESIMS spectrum

(±)Ethyl-4-hydroxy-3-(9*H*-purin-9-yl)tetrahydrothiophene-3-carboxylate (**1b**)

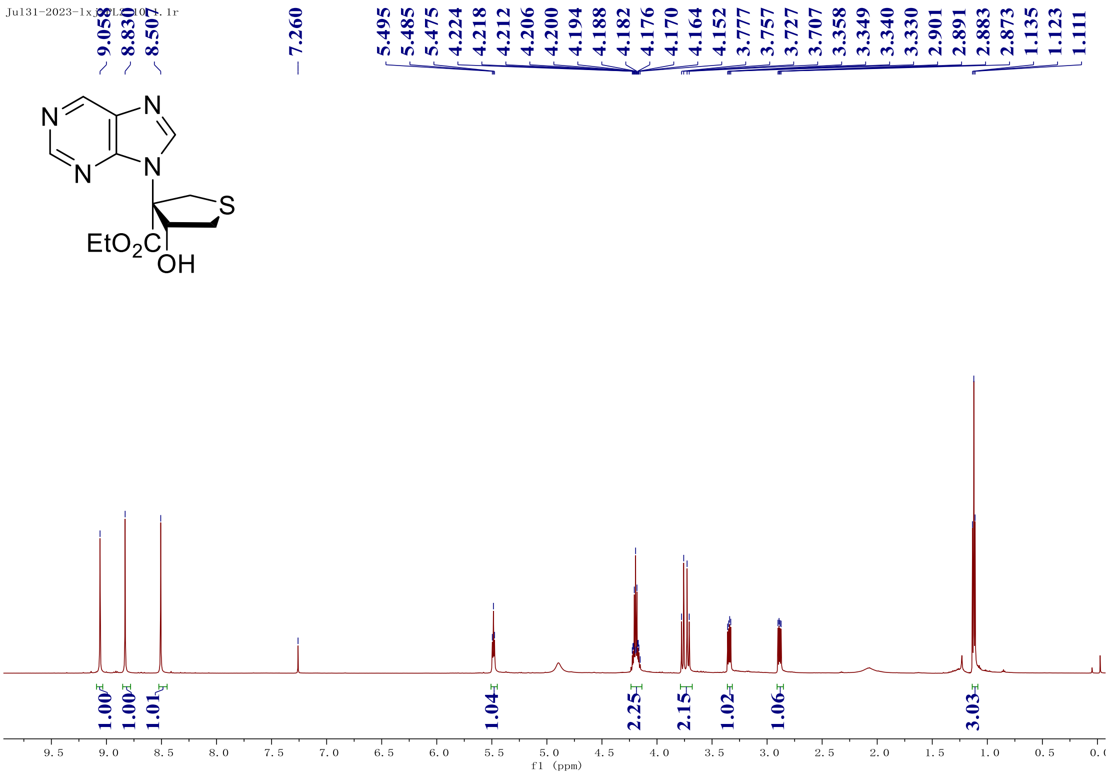

^{1}H NMR spectrum

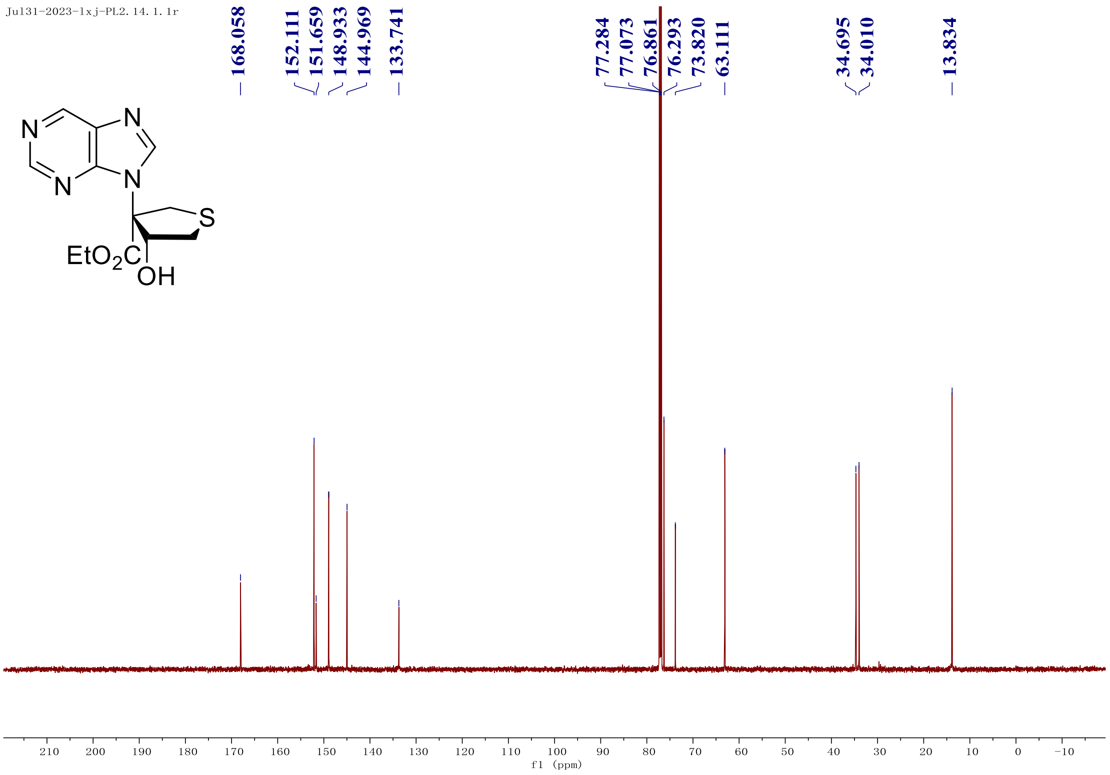

^{13}C NMR spectrum

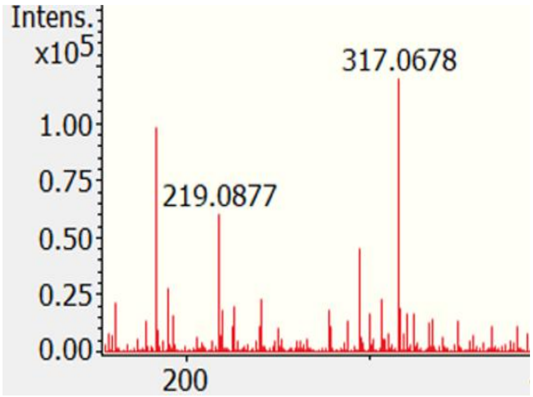

HR-ESIMS spectrum

(±)Ethyl-3-(6-chloro-9*H*-purin-9-yl)-4-hydroxytetrahydrothiophene-3-carboxylate (**2a**)

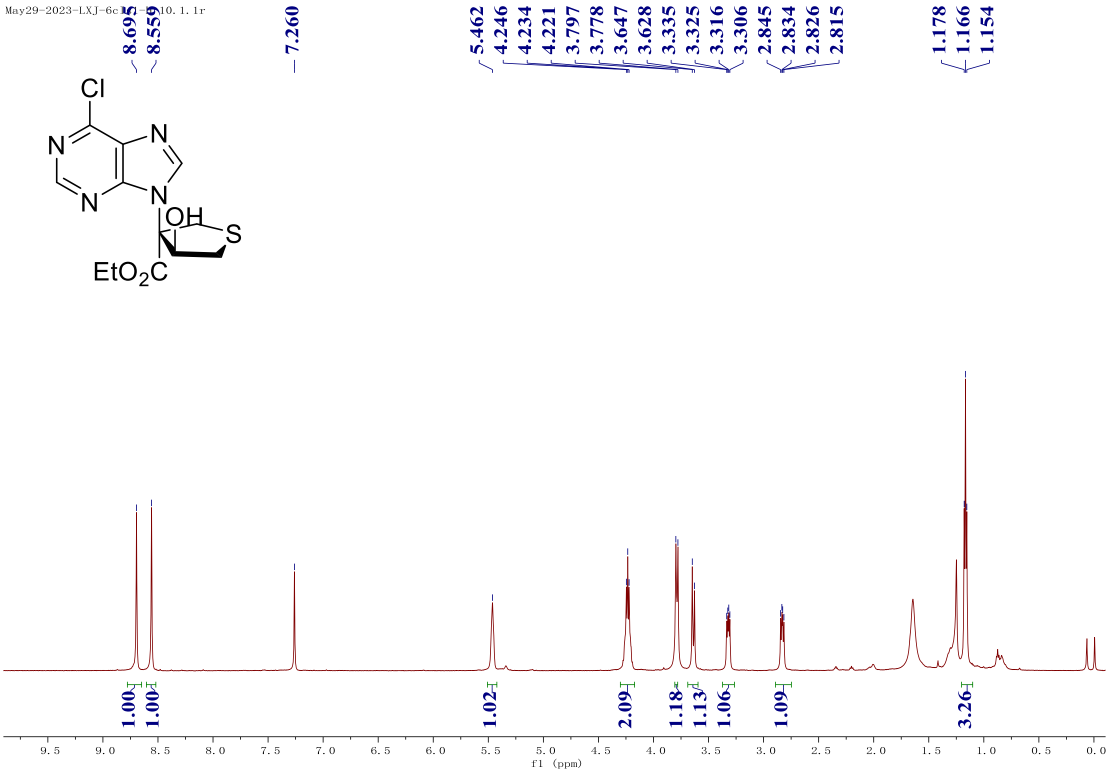

^{1}H NMR spectrum

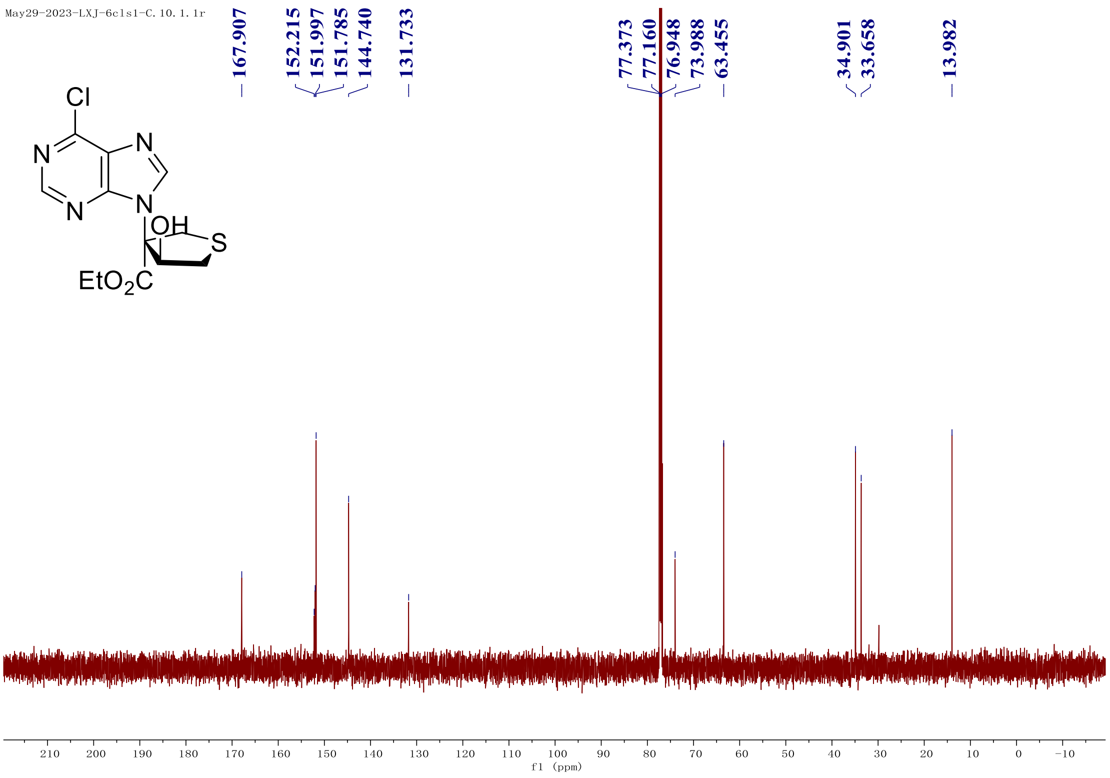

^{13}C NMR spectrum

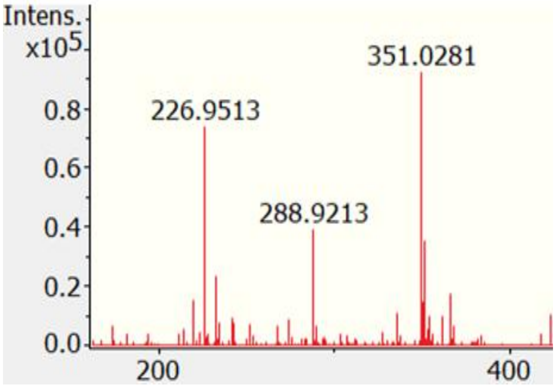

HR-ESIMS spectrum

(±)Ethyl-3-(6-chloro-9*H*-purin-9-yl)-4-hydroxytetrahydrothiophene-3-carboxylate (**2b**)

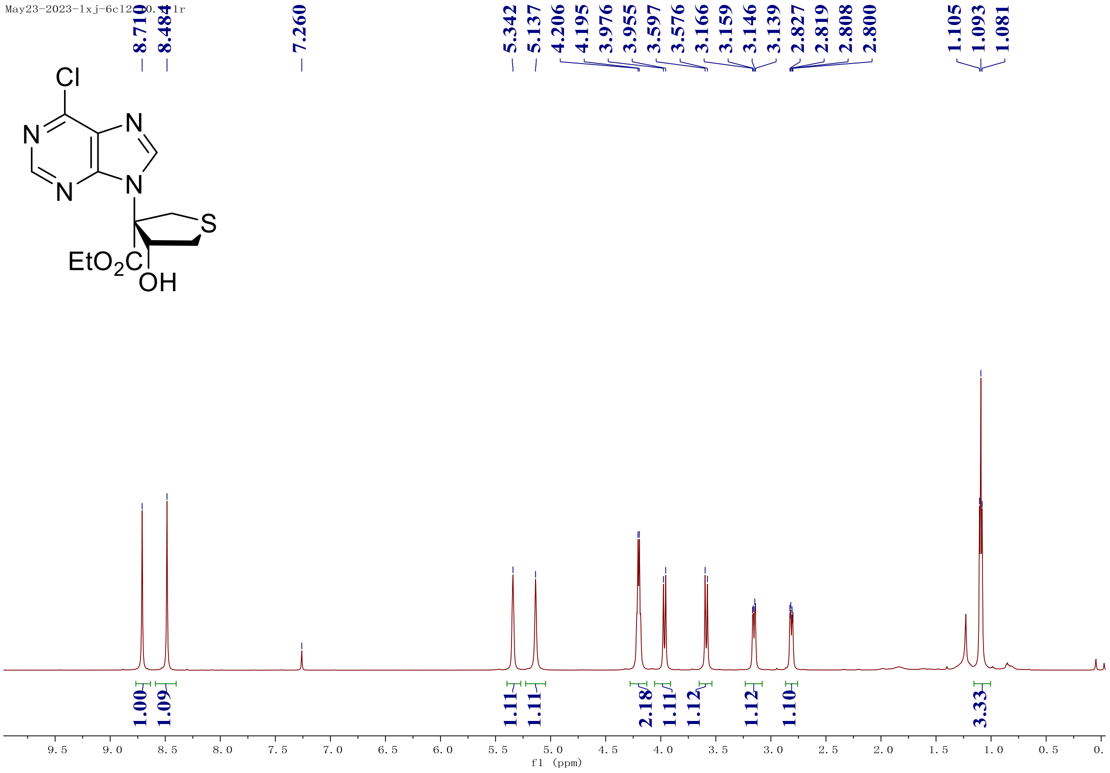

^{1}H NMR spectrum

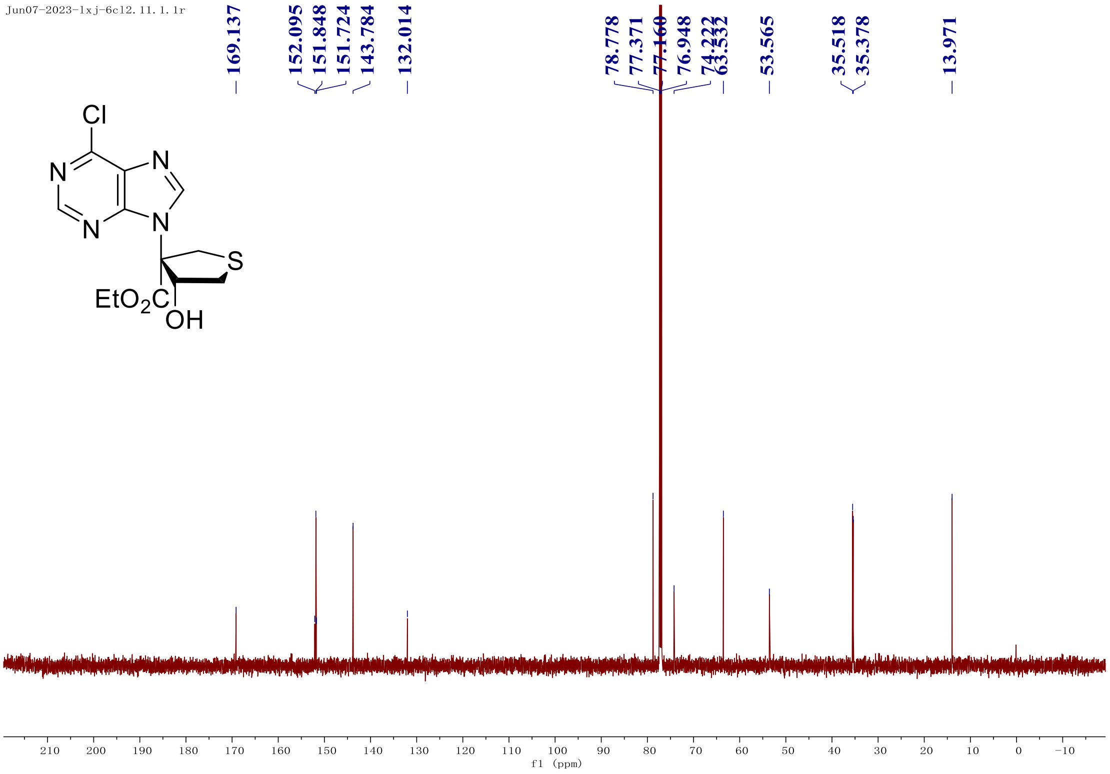

^{13}C NMR spectrum

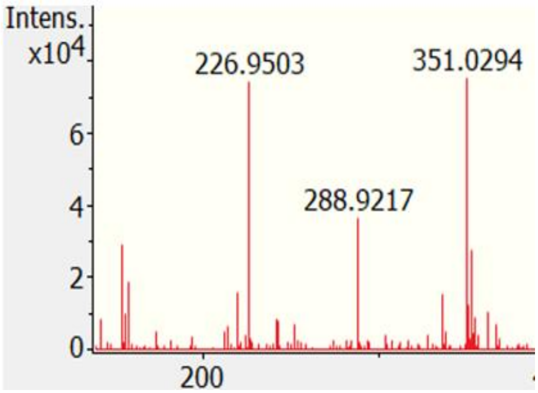

HR-ESIMS spectrum

(±)Ethyl-3-(6-bromo-9*H*-purin-9-yl)-4-hydroxytetrahydrothiophene-3-carboxylate (**3a**)

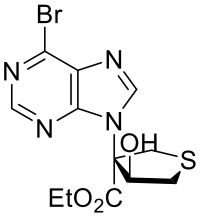

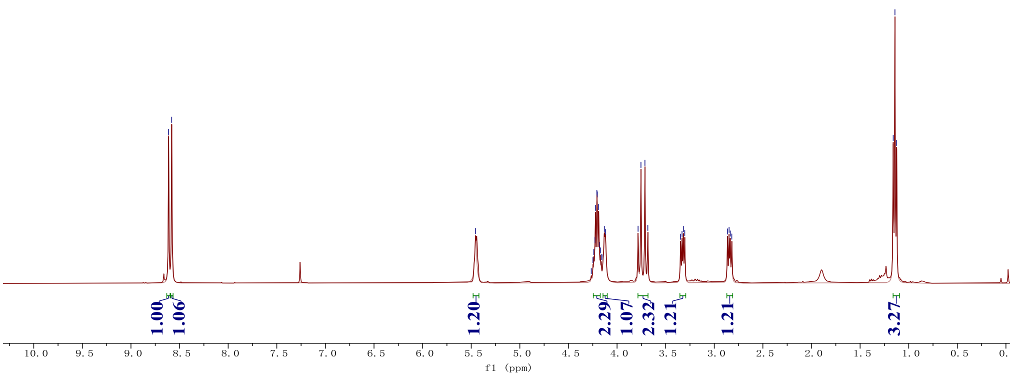

^{1}H NMR spectrum

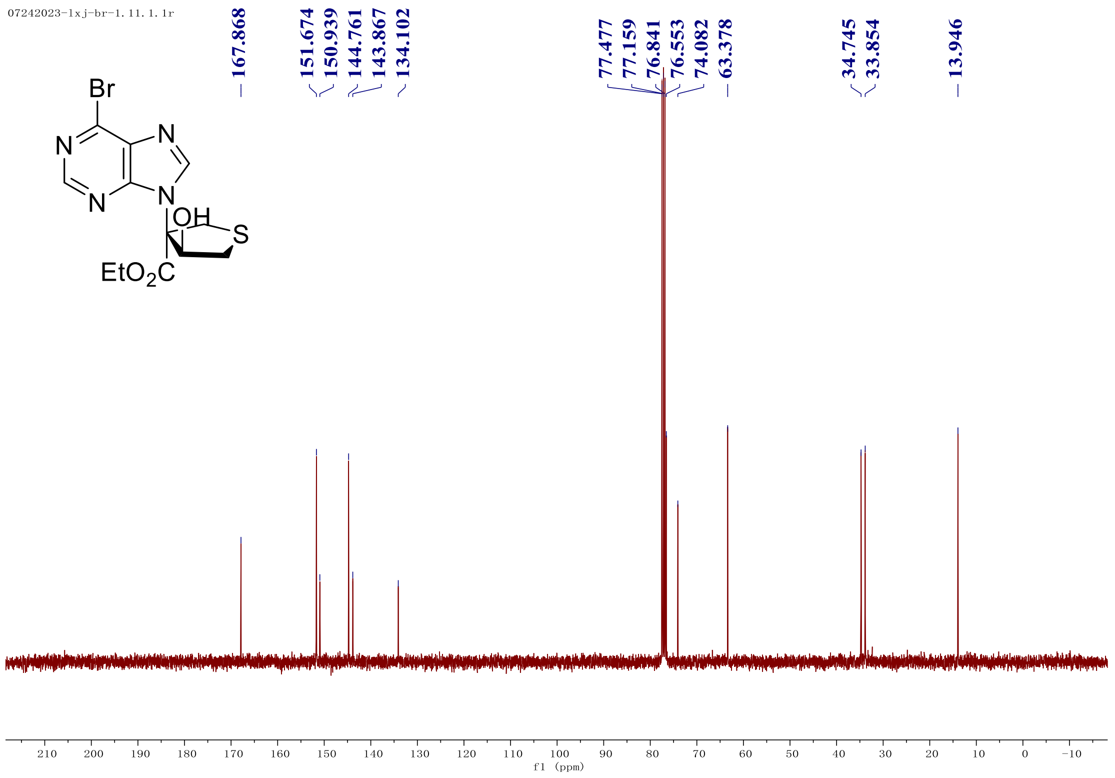

^{13}C NMR spectrum

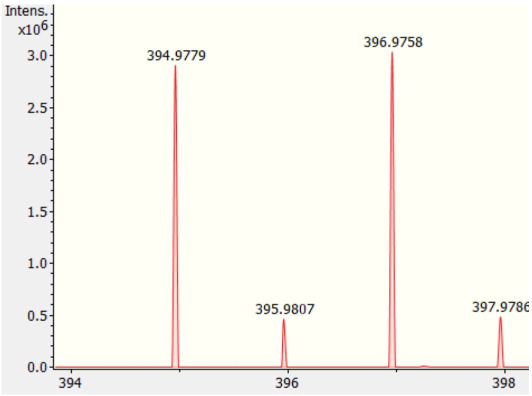

HR-ESIMS spectrum

(±)-Ethyl-3-(6-bromo-9*H*-purin-9-yl)-4-hydroxytetrahydrothiophene-3-carboxylate (**3b**)

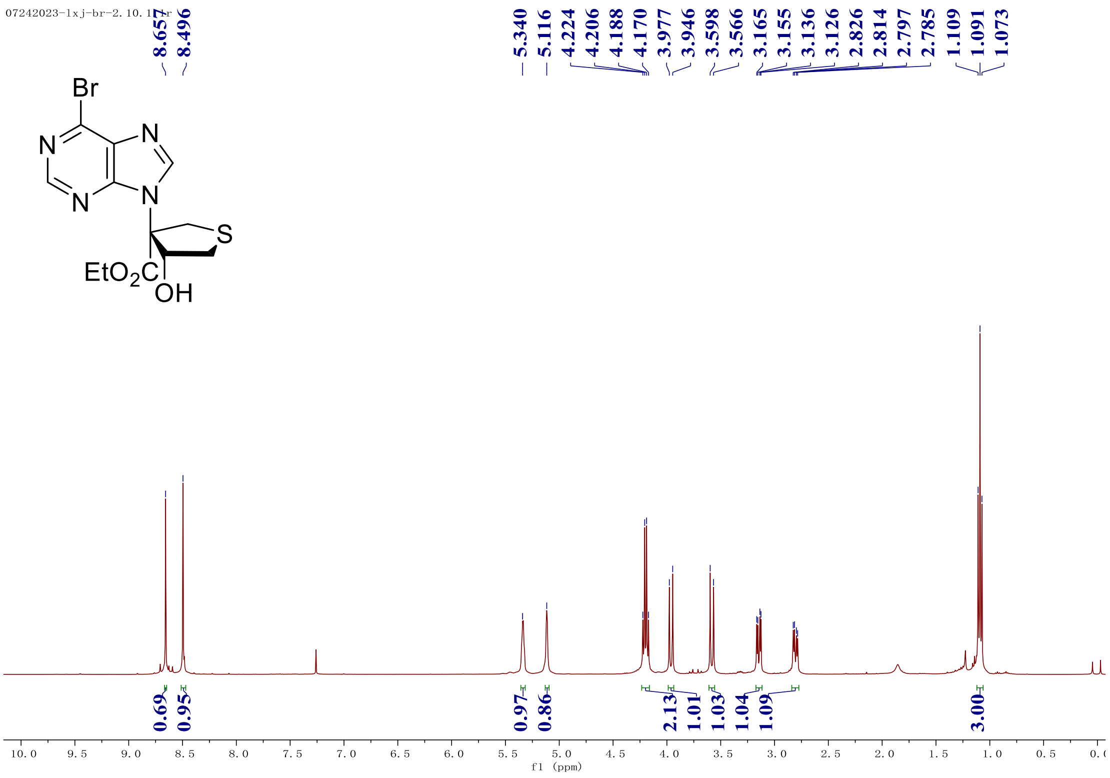

^1H NMR spectrum

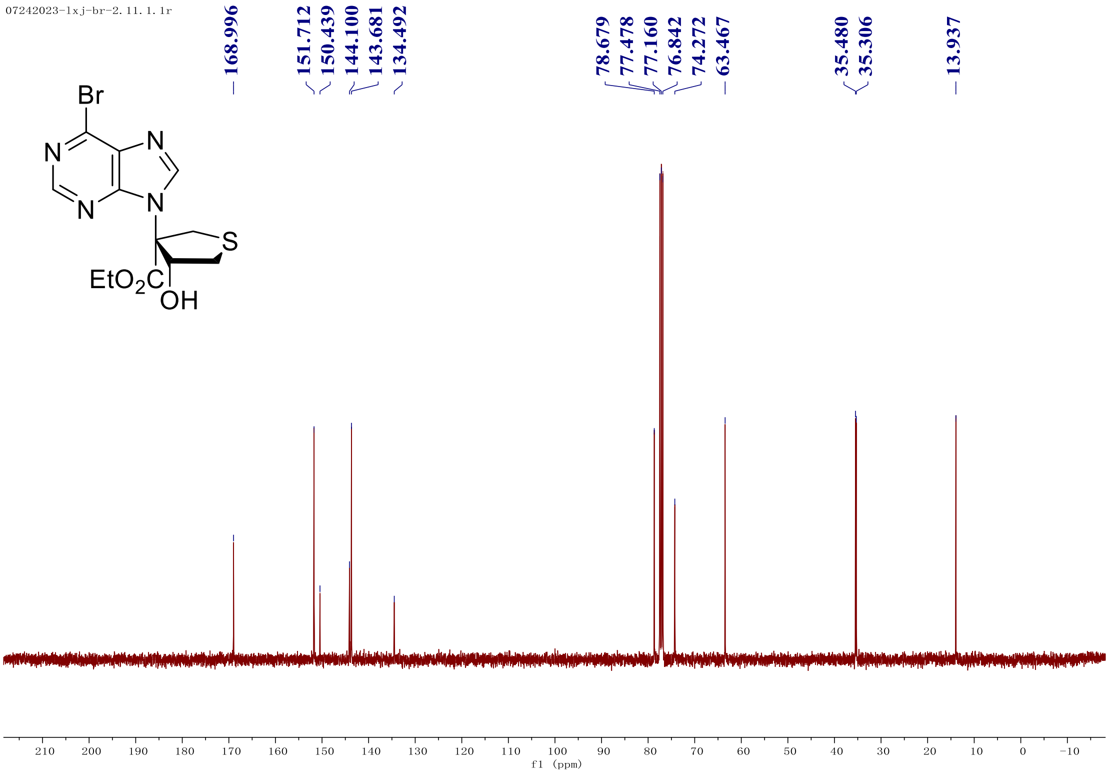

^{13}C NMR spectrum

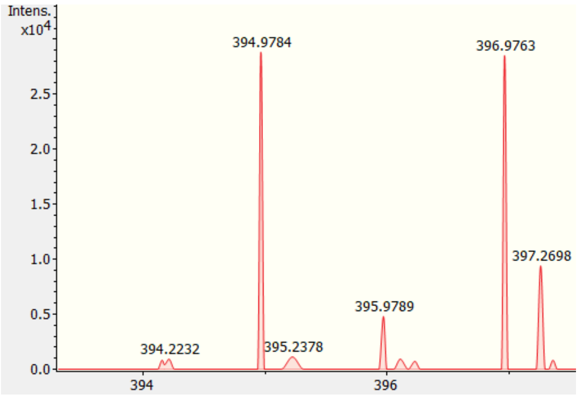

HR-ESIMS spectrum

(±)Ethyl-3-(6-(dimethylamino)-9*H*-purin-9-yl)-4-hydroxytetrahydrothiophene-3-carboxylate (**4a**)

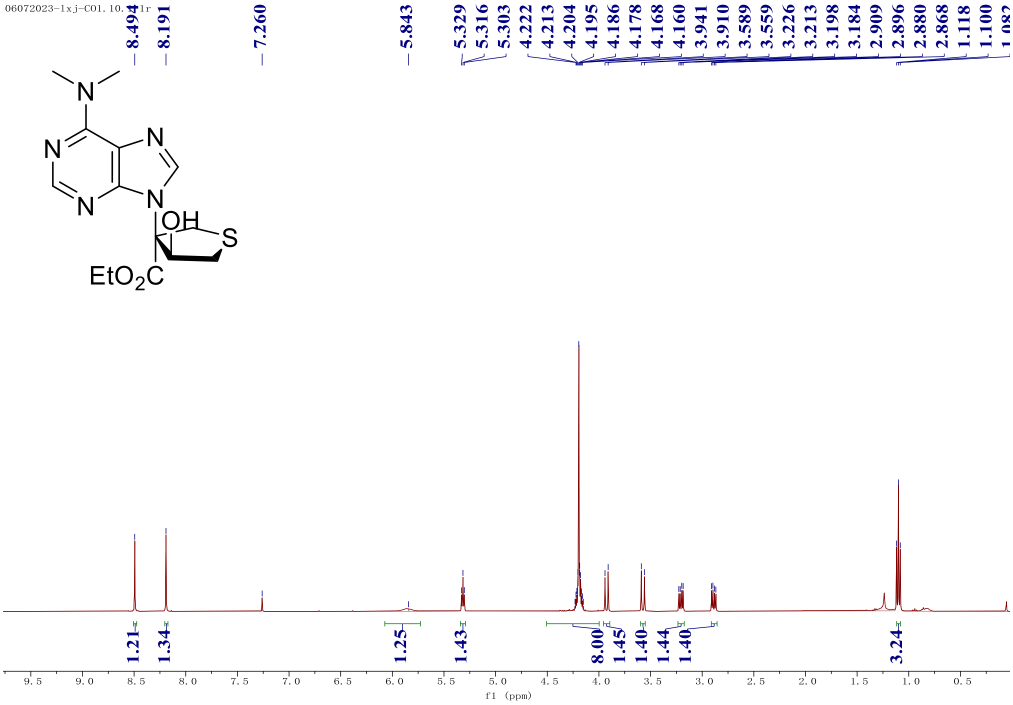

^{1}H NMR spectrum

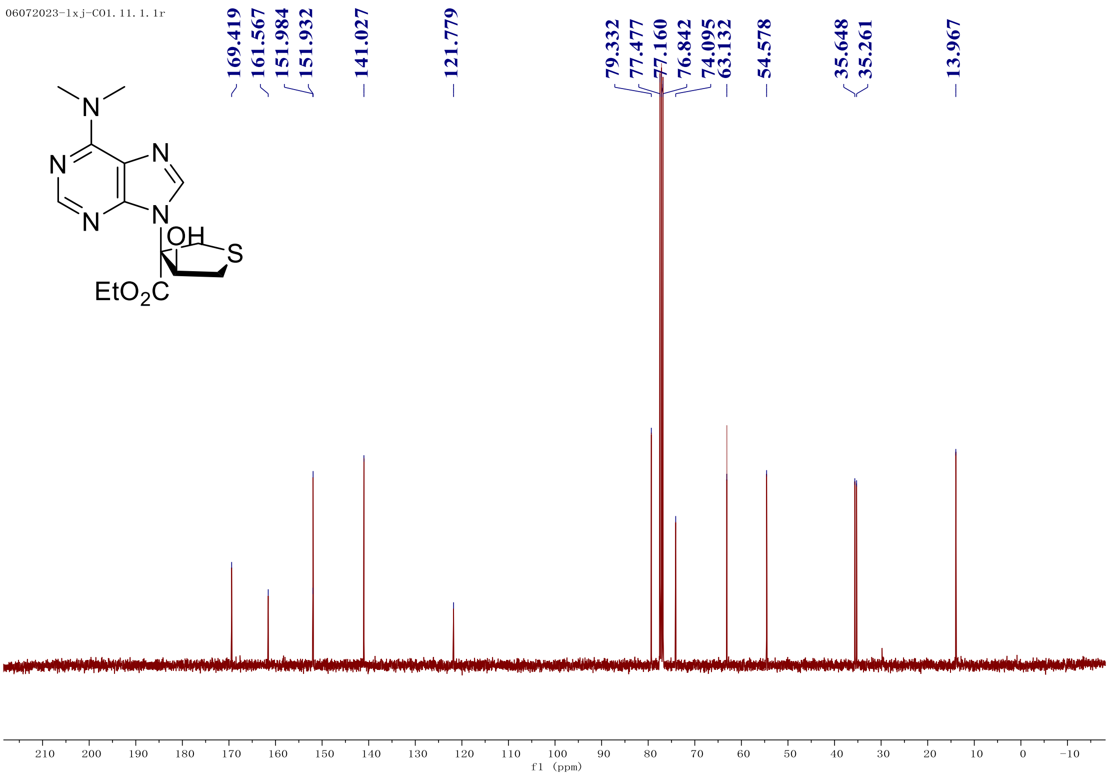

^{13}C NMR spectrum

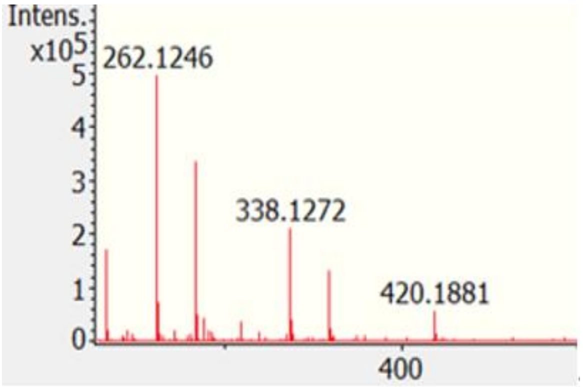

HR-ESIMS spectrum

(±)Ethyl-3-(6-(dimethylamino)-9*H*-purin-9-yl)-4-hydroxytetrahydrothiophene-3-carboxylate (**4b**)

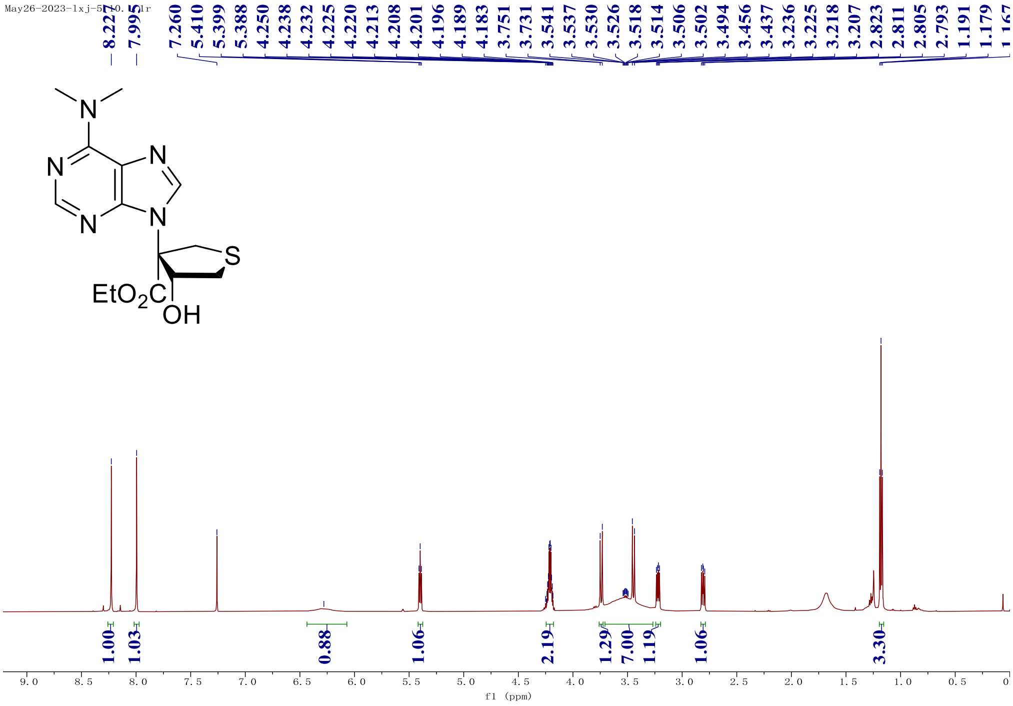

^{1}H NMR spectrum

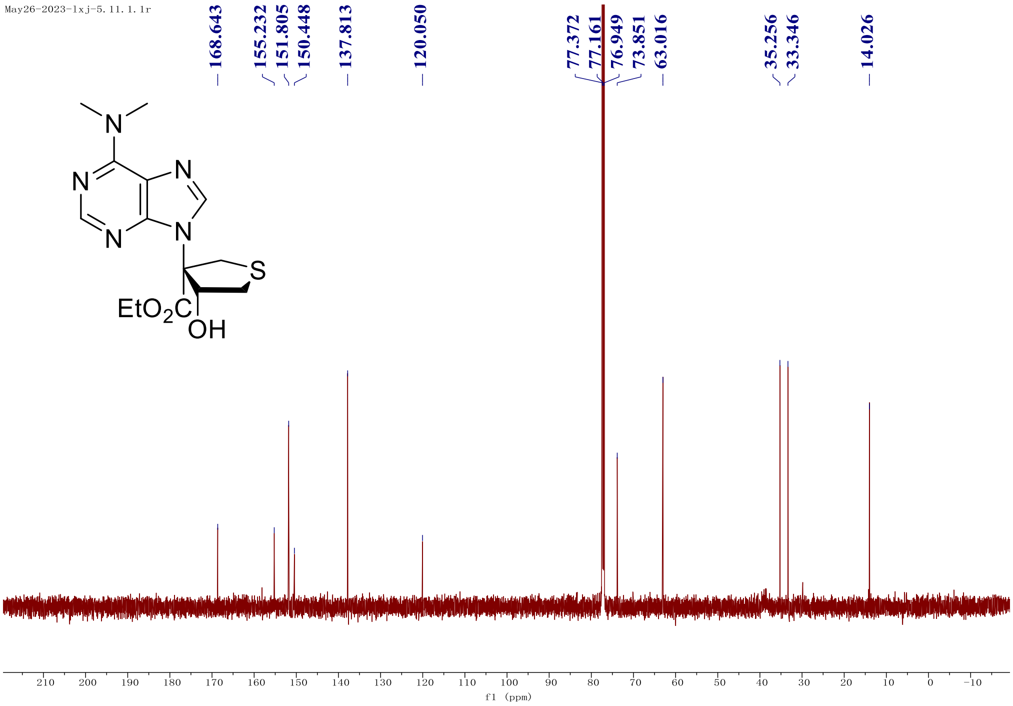

^{13}C NMR spectrum

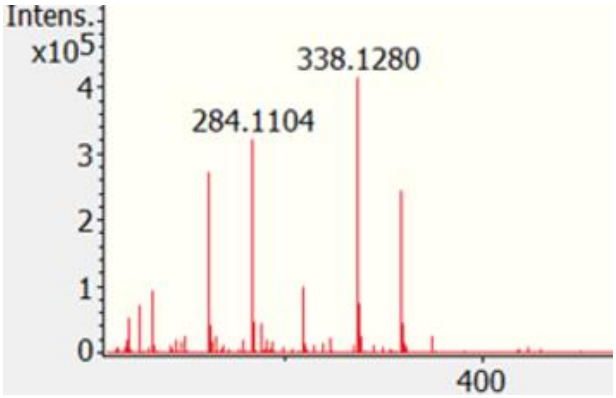

HR-ESIMS spectrum

(±)Ethyl-4-hydroxy-3-(6-(piperidin-1-yl)-9*H*-purin-9-yl)tetrahydrothiophene-3-carboxylate (**5a**)

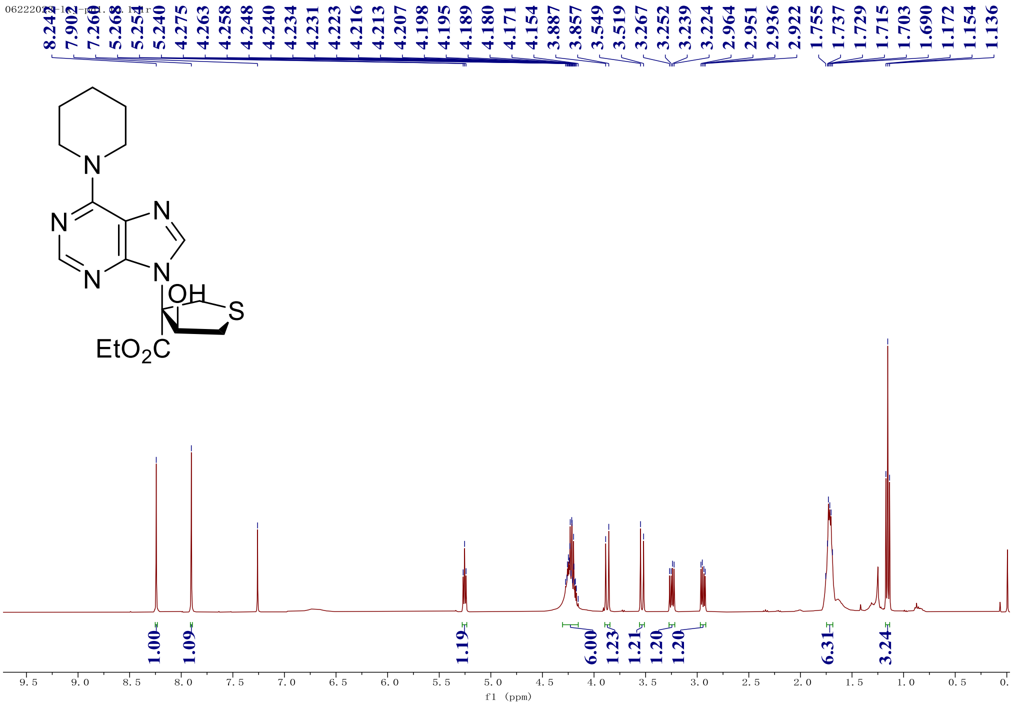

^{1}H NMR spectrum

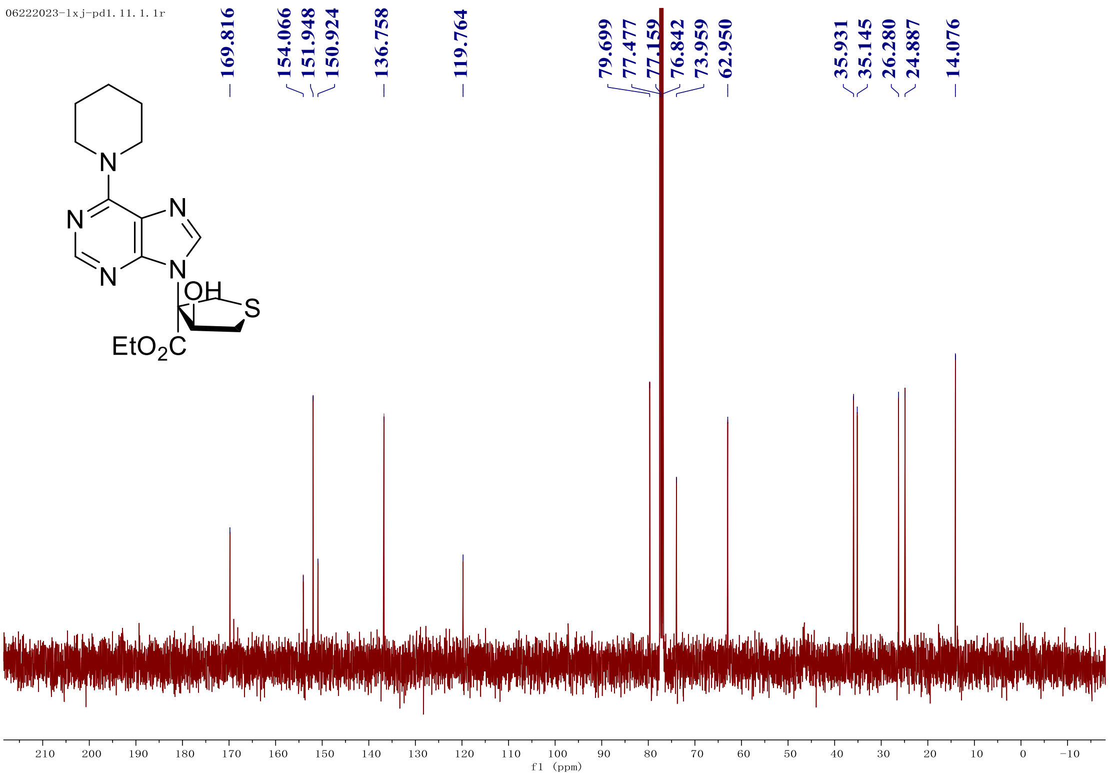

^{13}C NMR spectrum

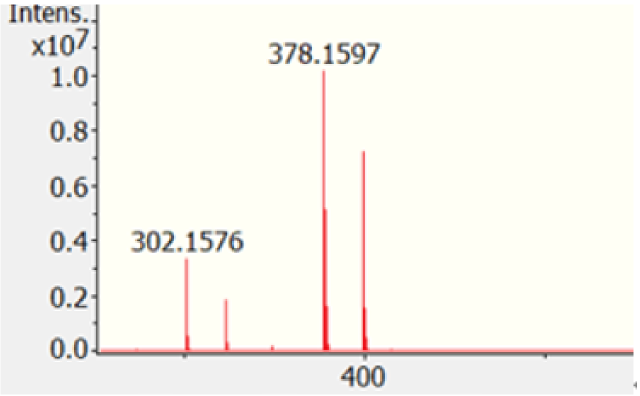

HR-ESIMS spectrum

(±)Ethyl-4-hydroxy-3-(6-(piperidin-1-yl)-9*H*-purin-9-yl)tetrahydrothiophene-3-carboxylate (**5b**)

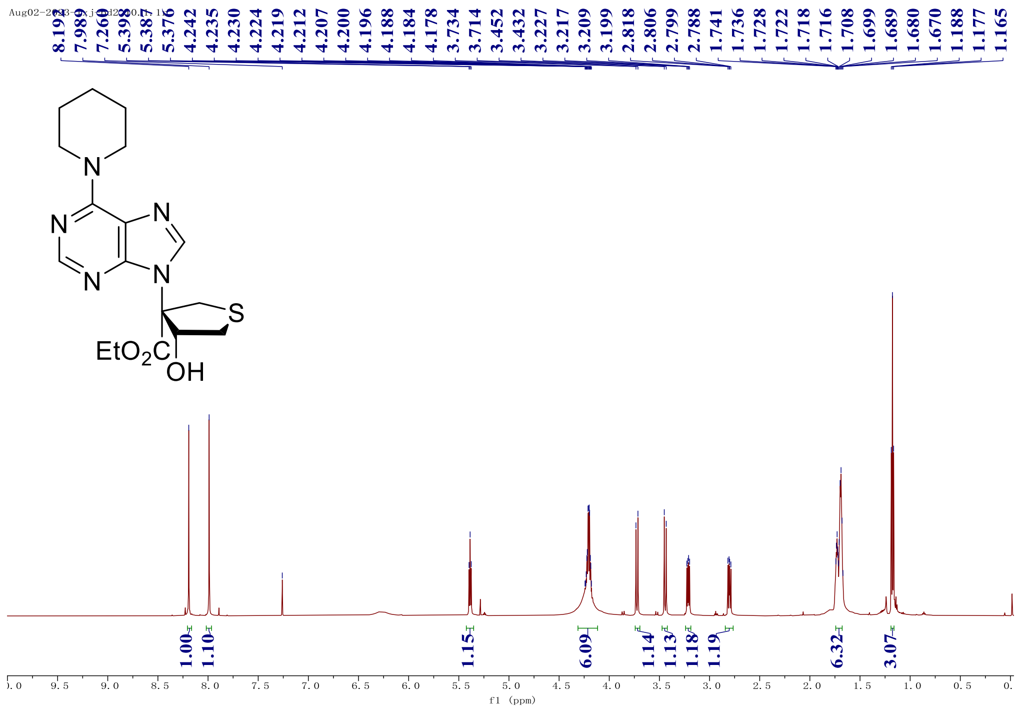

^1H NMR spectrum

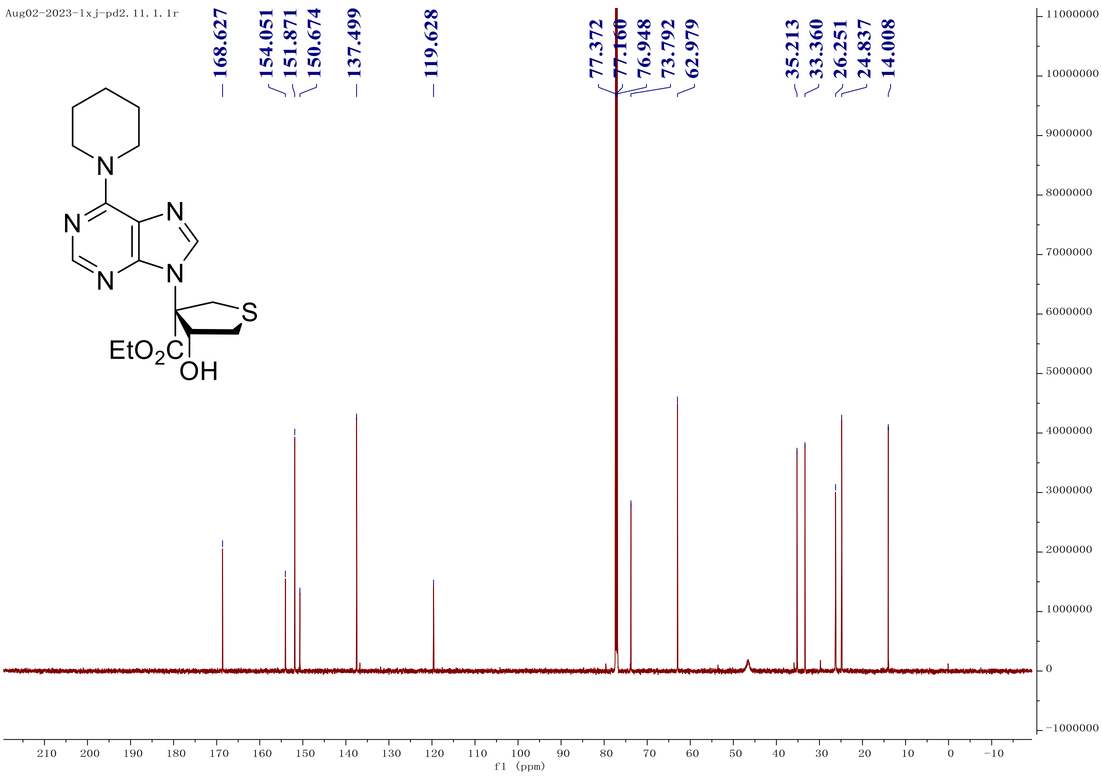

^{13}C NMR spectrum

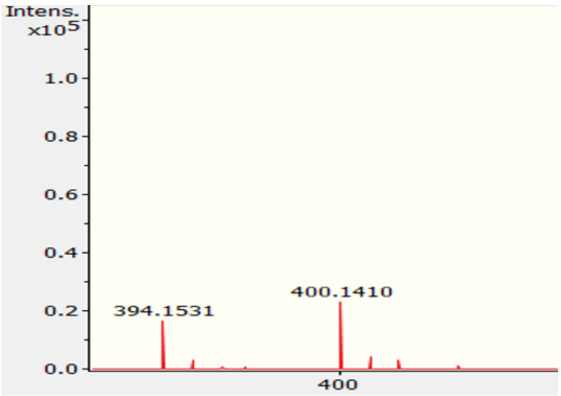

HR-ESIMS spectrum

(±)Ethyl-3-(6-ethoxy-9*H*-purin-9-yl)-4-hydroxytetrahydrothiophene-3-carboxylate (**6a**)

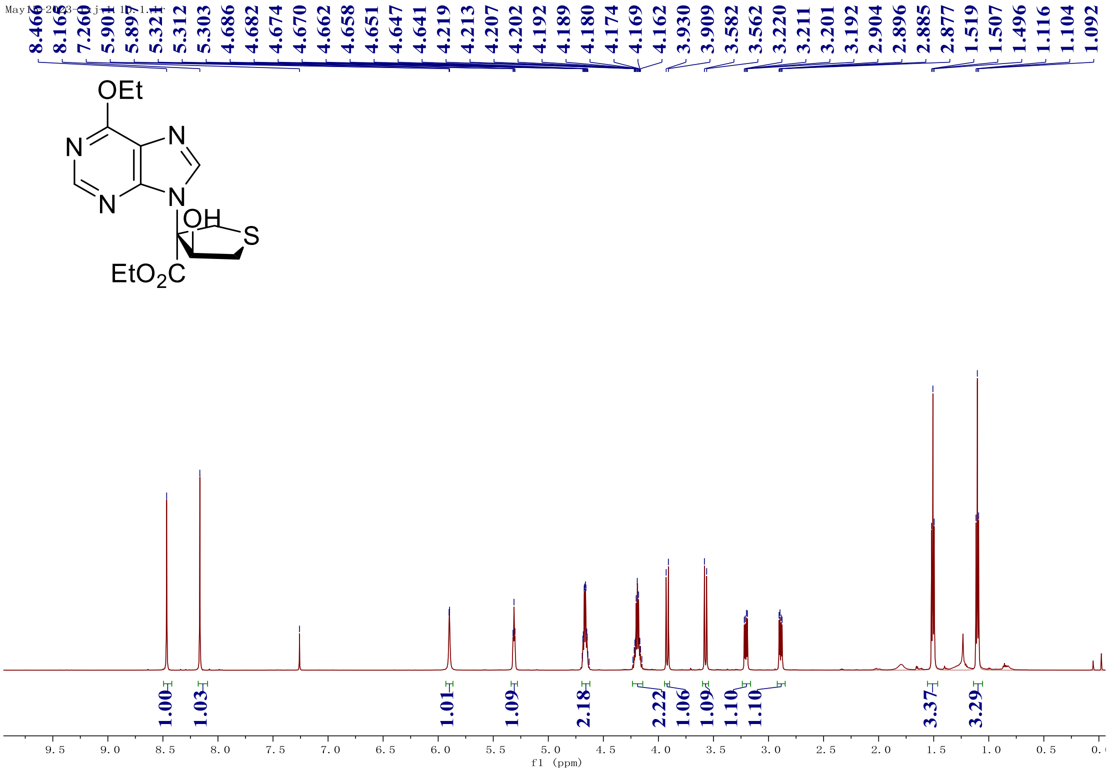

^{1}H NMR spectrum

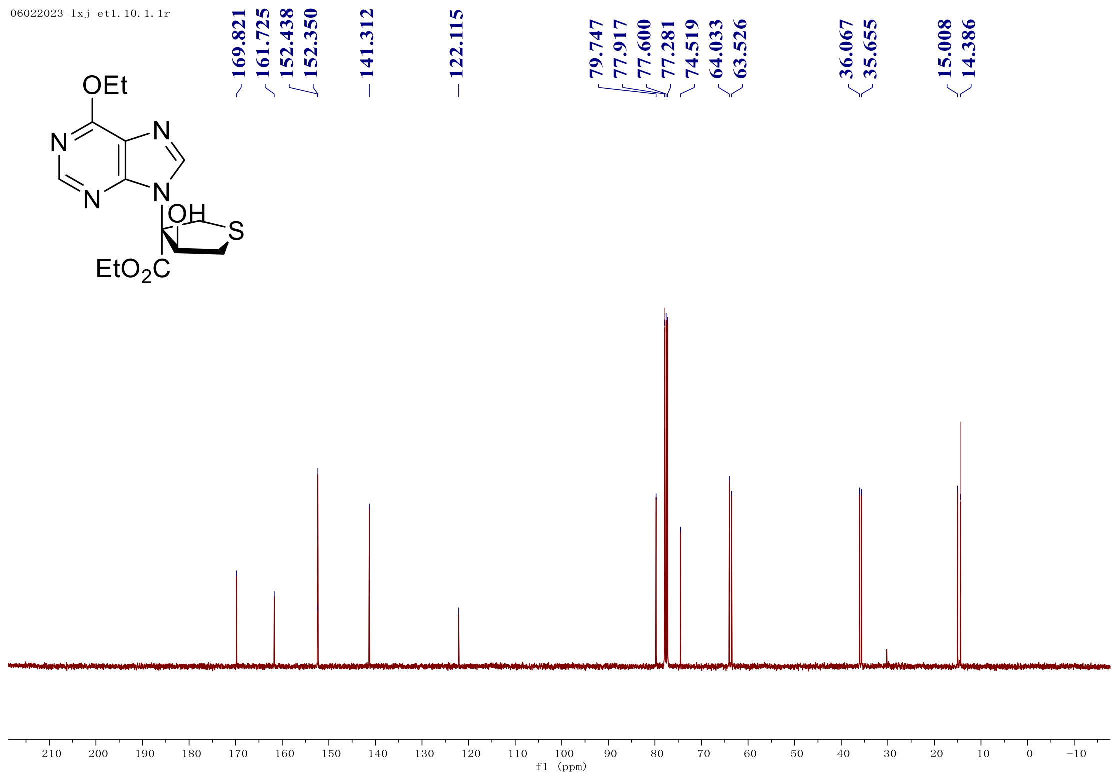

^{13}C NMR spectrum

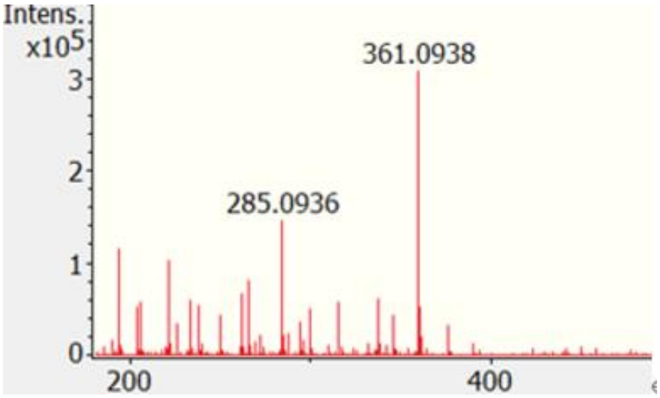

HR-ESIMS spectrum

(±)Ethyl-3-(6-ethoxy-9*H*-purin-9-yl)-4-hydroxytetrahydrothiophene-3-carboxylate (**6b**)

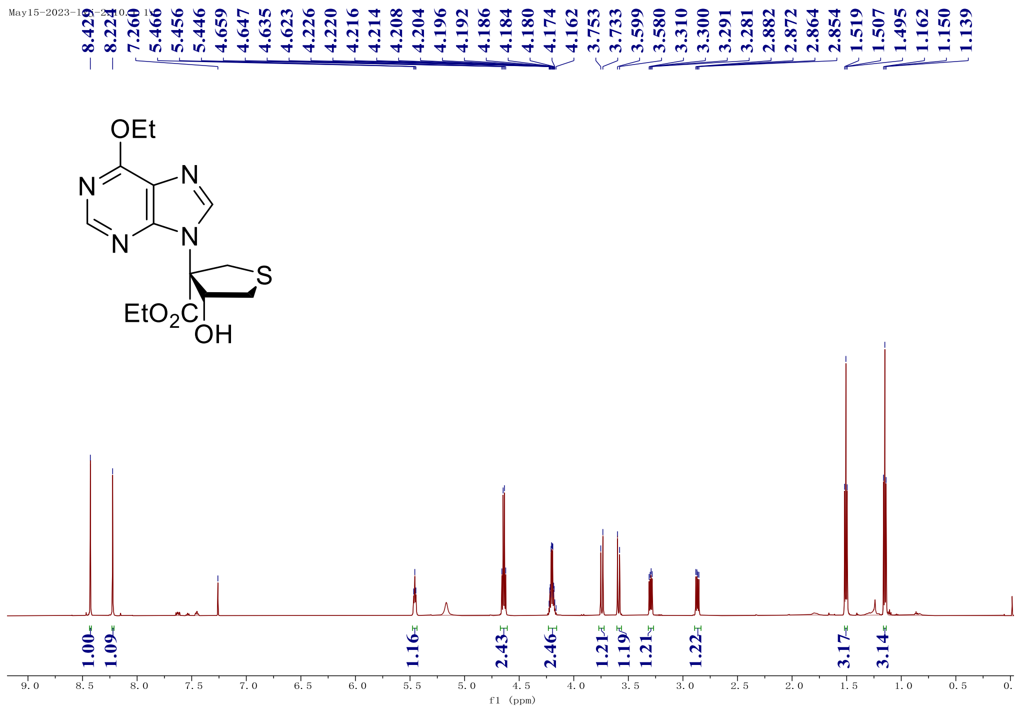

^1H NMR spectrum

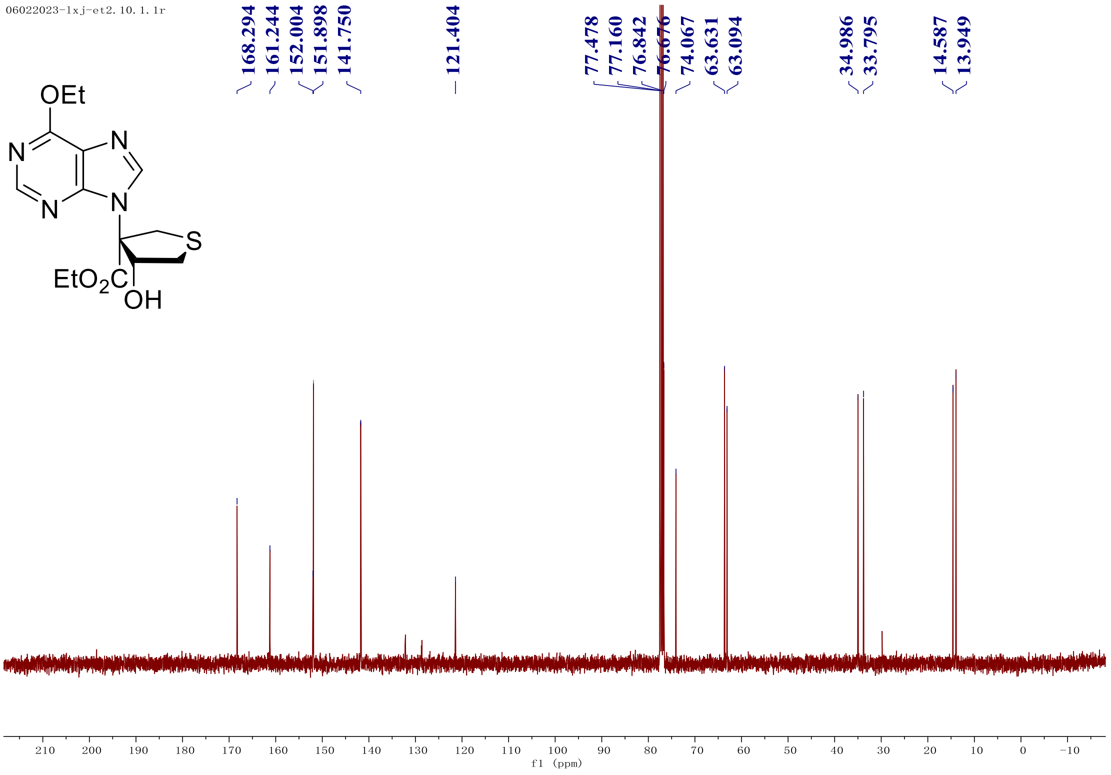

^{13}C NMR spectrum

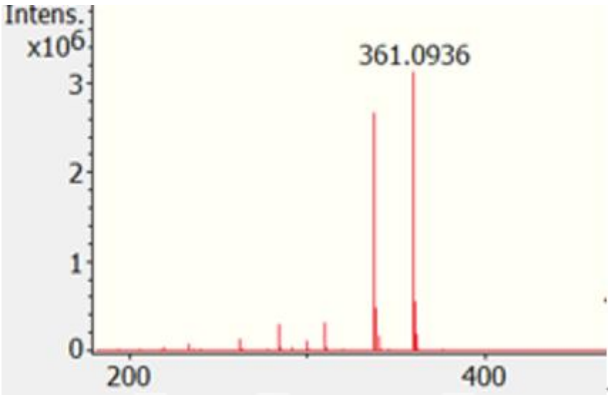

HR-ESIMS spectrum

(±)Ethyl-4-hydroxy-3-(6-methoxy-9*H*-purin-9-yl)tetrahydrothiophene-3-carboxylate (**7a**)

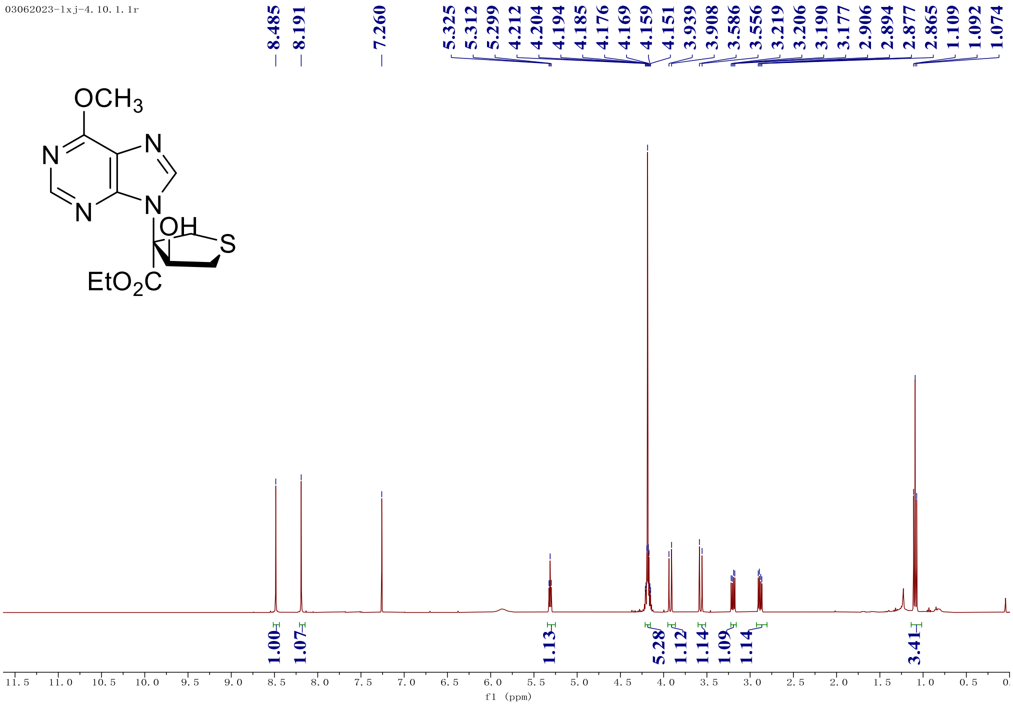

^{1}H NMR spectrum

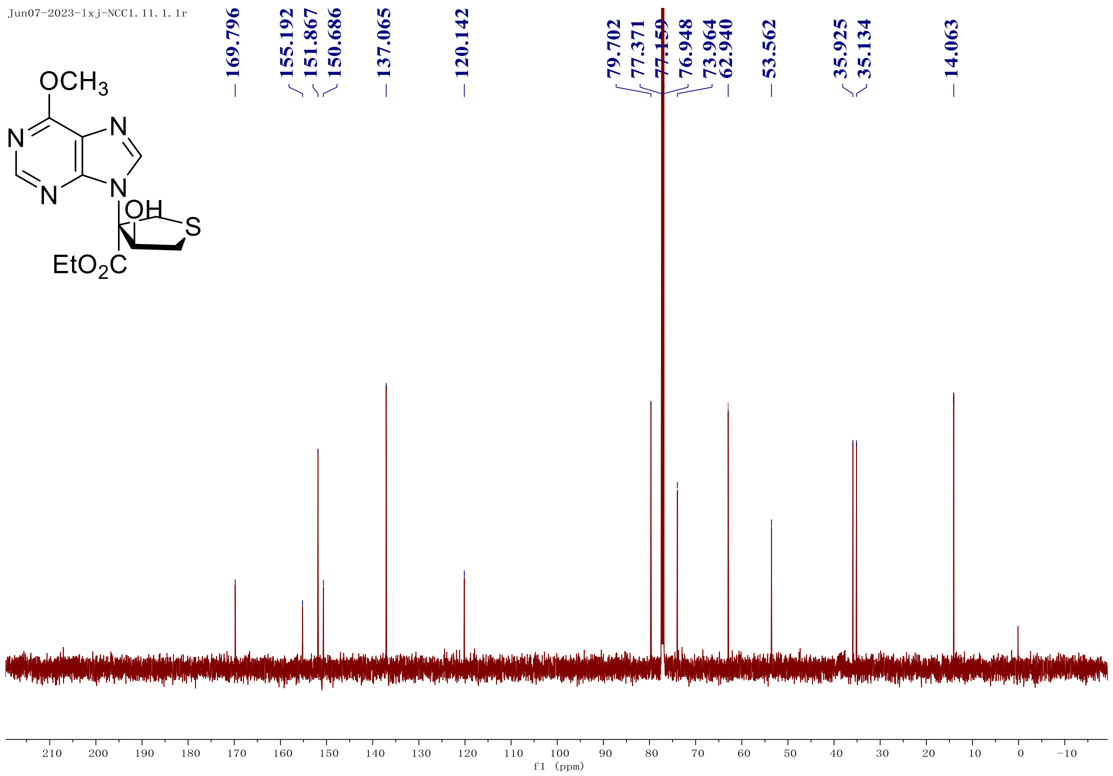

^{13}C NMR spectrum

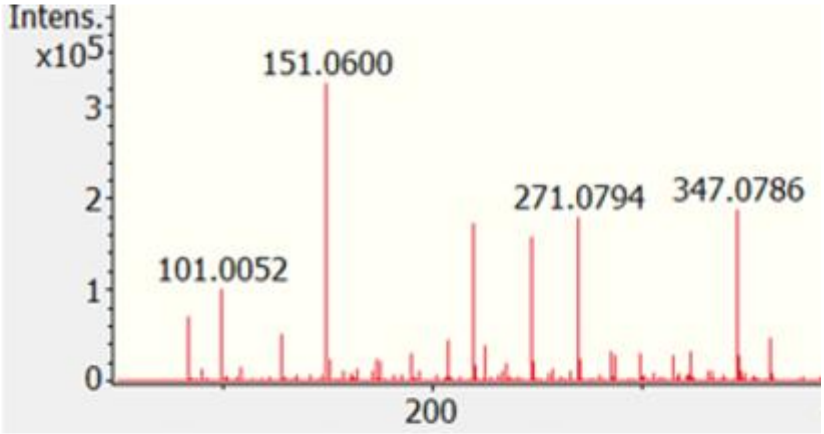

HR-ESIMS spectrum

(±)Ethyl-4-hydroxy-3-(6-methoxy-9*H*-purin-9-yl)tetrahydrothiophene-3-carboxylate (**7b**)

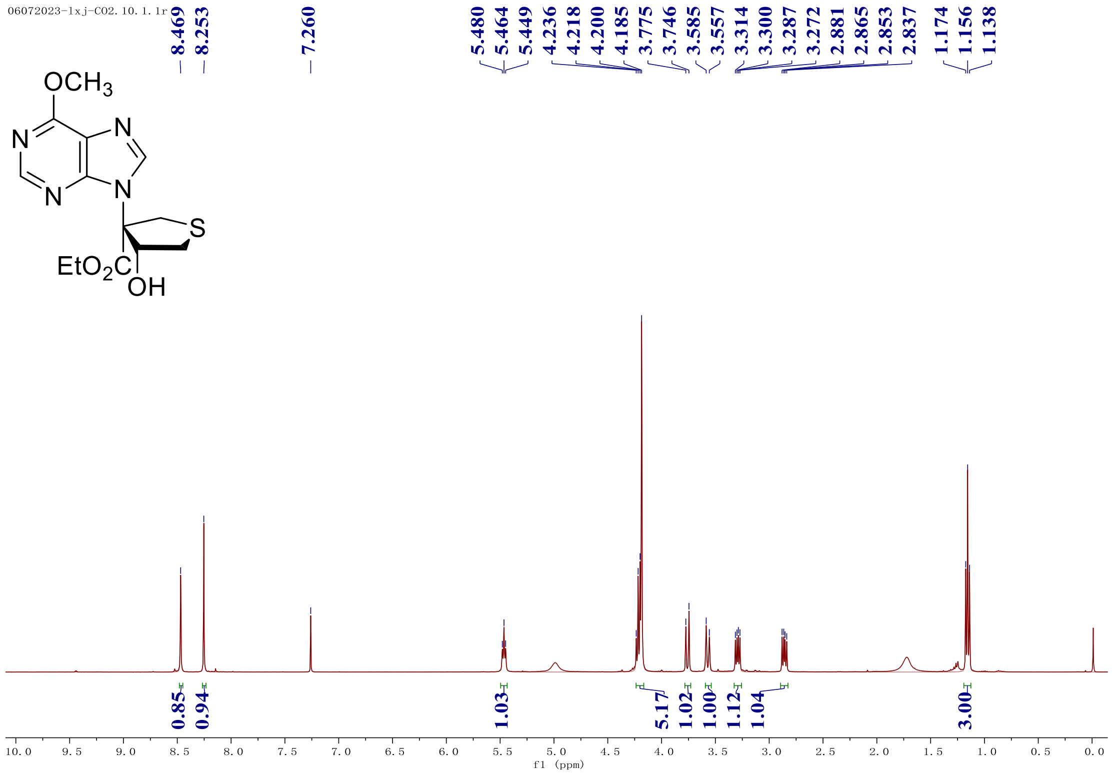

^{1}H NMR spectrum

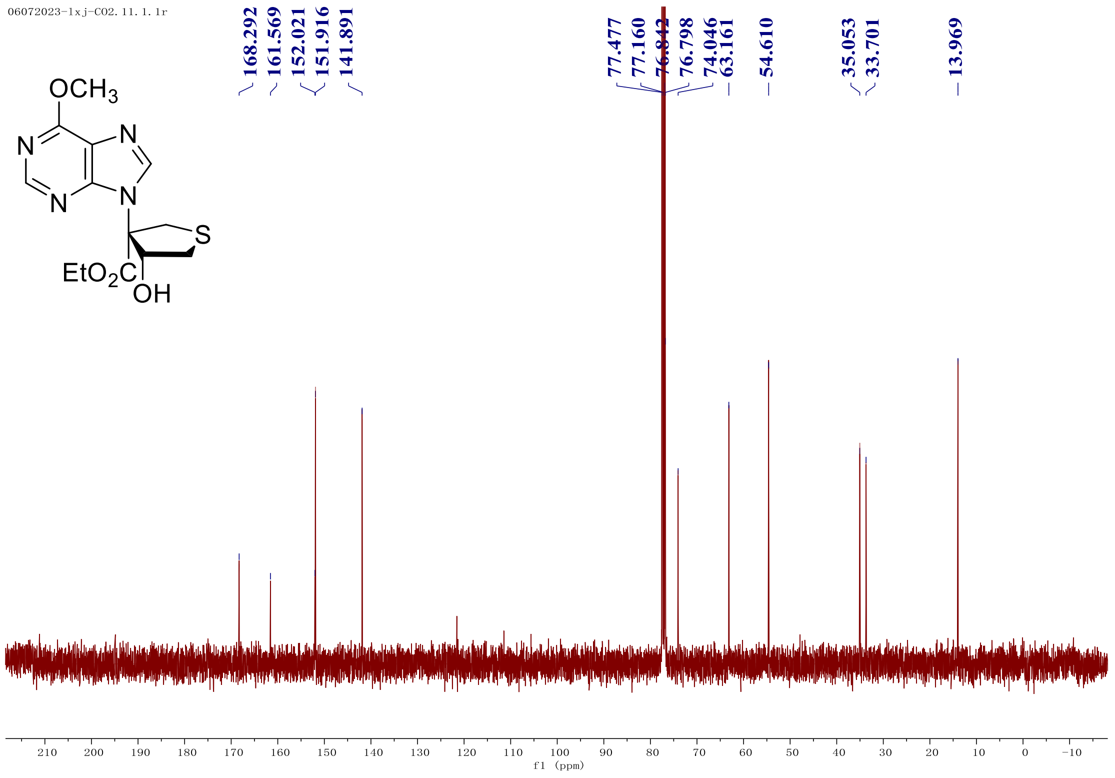

^{13}C NMR spectrum

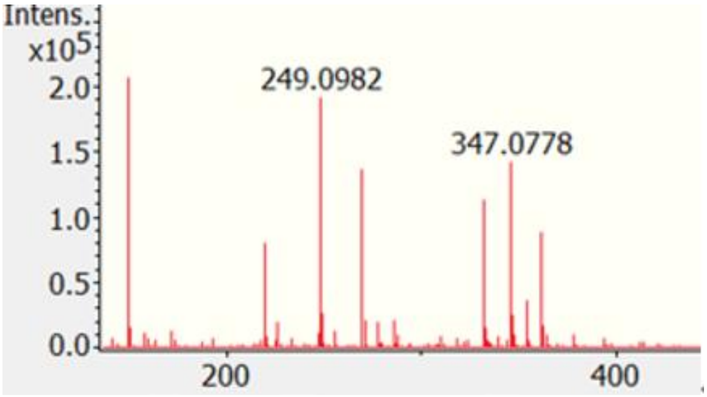

HR-ESIMS spectrum

(±)Ethyl-4-hydroxy-3-(6-(propylthio)-9*H*-purin-9-yl) tetrahydrothiophene-3-carboxylate (**8a**)

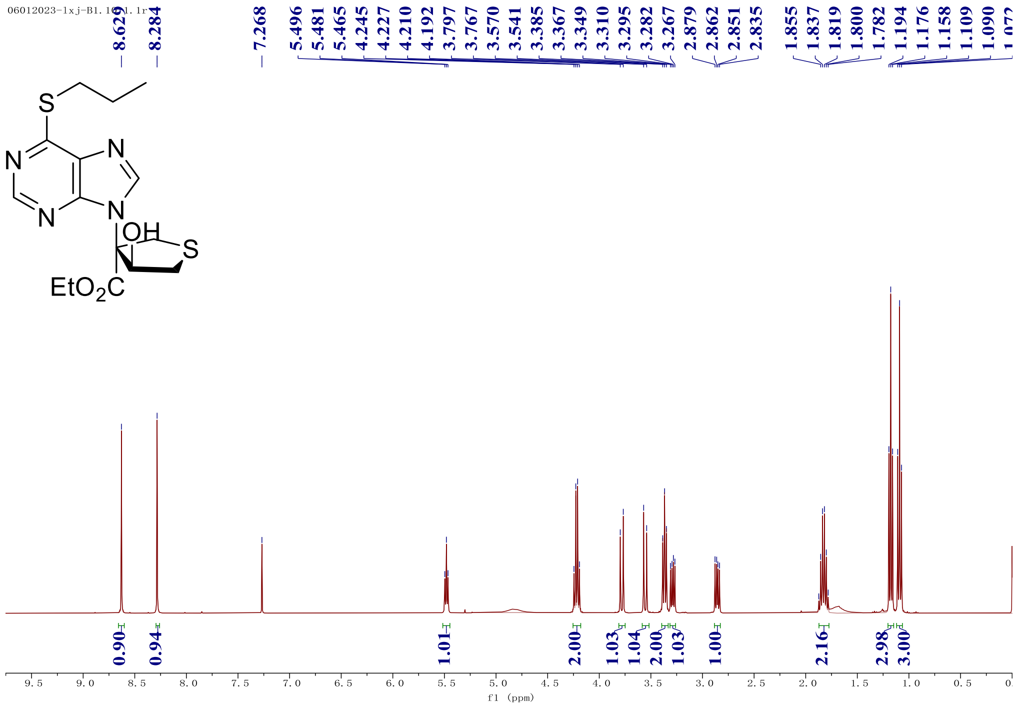

^{1}H NMR spectrum

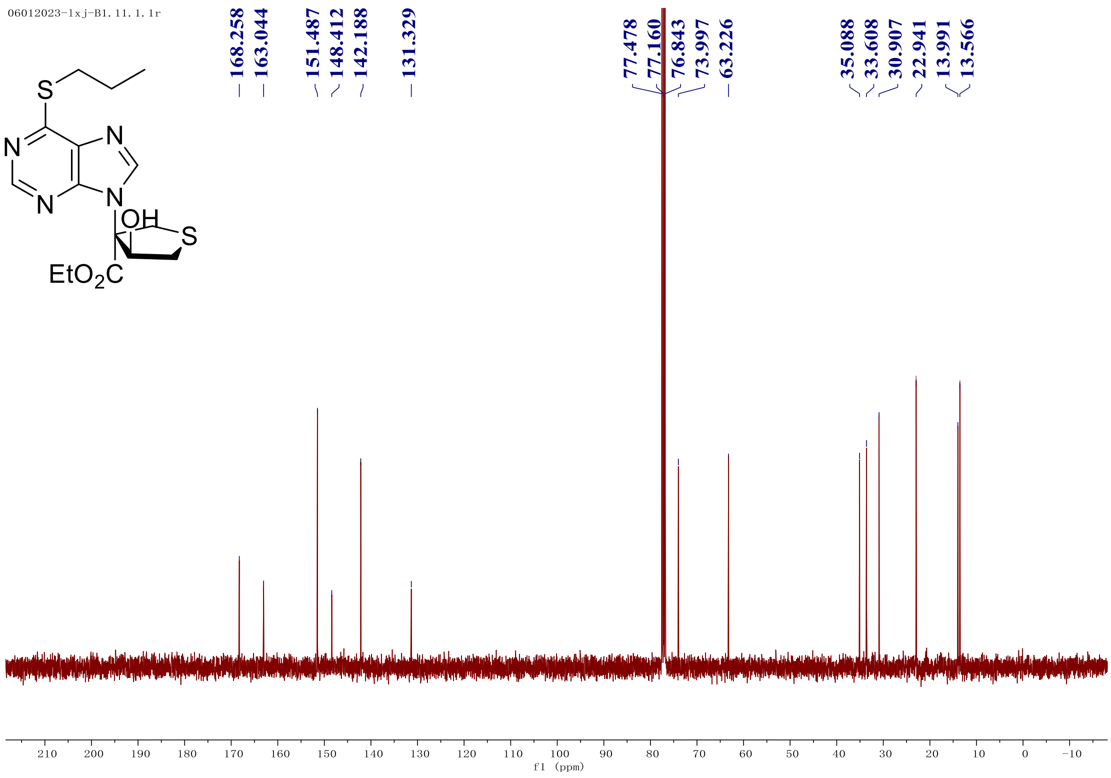

^{13}C NMR spectrum

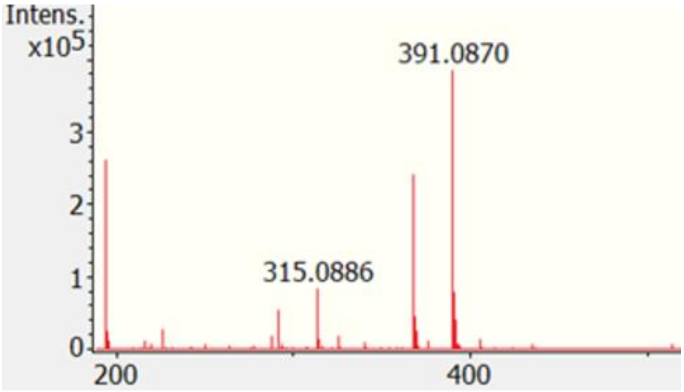

HR-ESIMS spectrum

(±)Ethyl-4-hydroxy-3-(6-(propylthio)-9*H*-purin-9-yl)tetrahydrothiophene-3-carboxylate (**8b**)

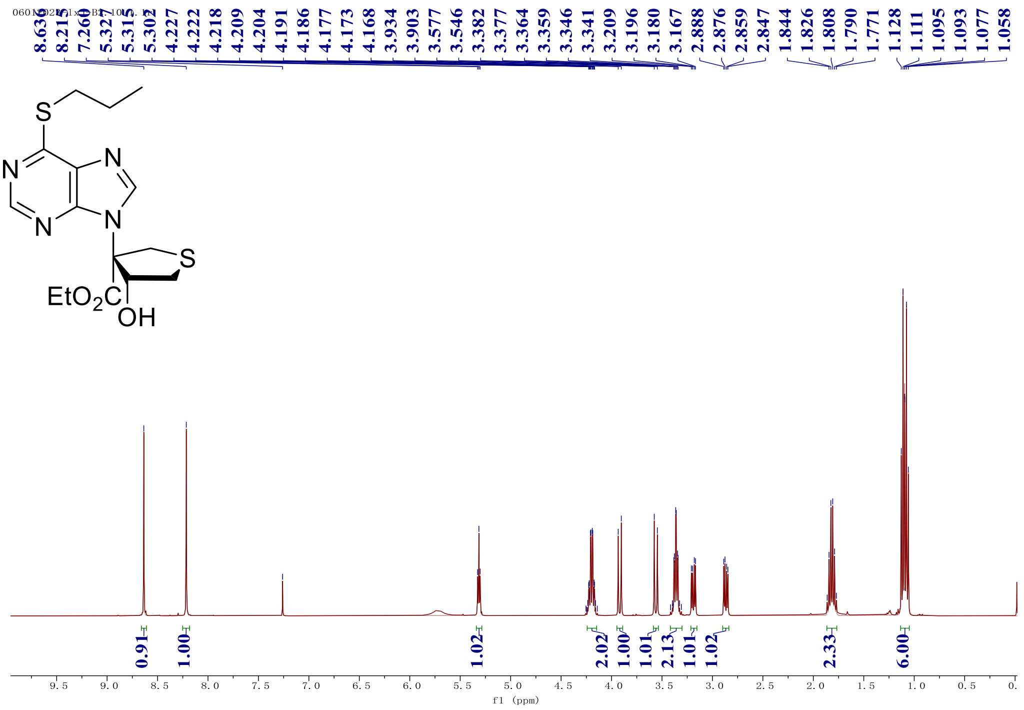

^1H NMR spectrum

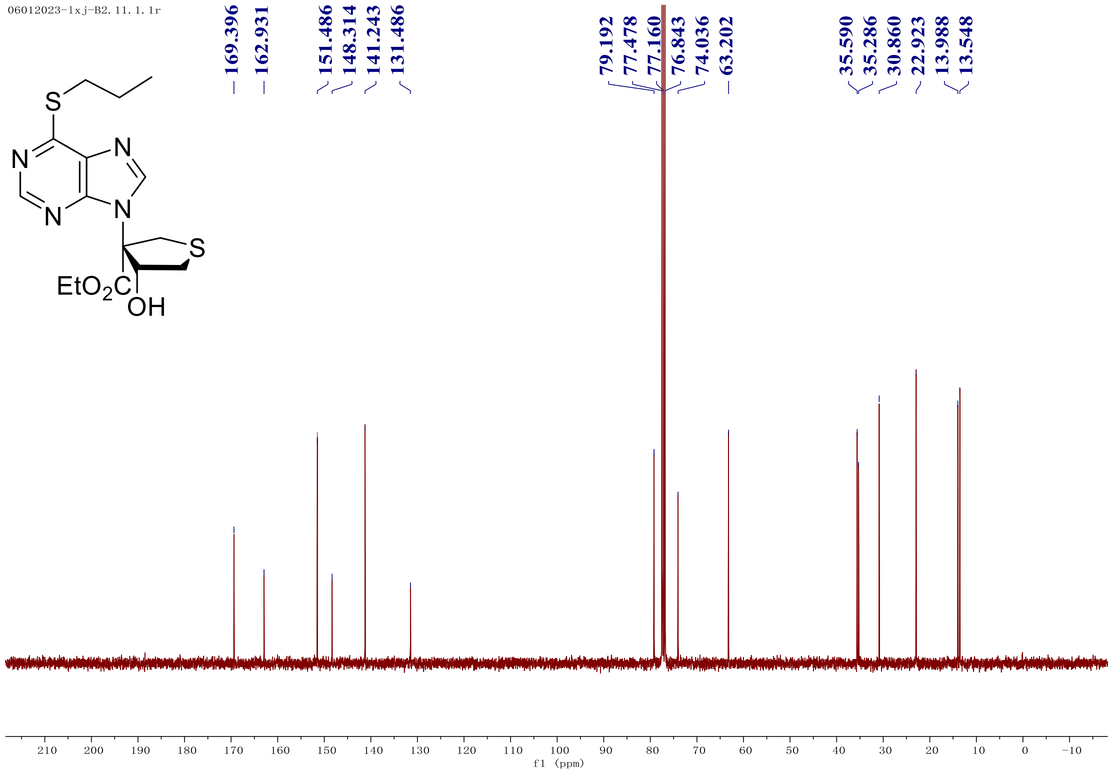

^{13}C NMR spectrum

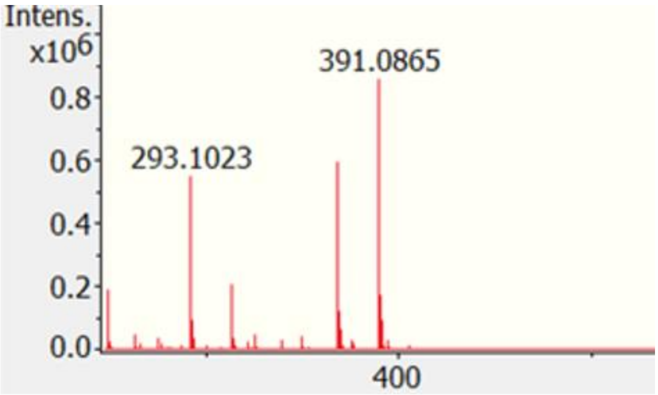

HR-ESIMS spectrum

(±)Ethyl-4-hydroxy-3-(6-morpholino-9*H*-purin-9-yl)tetrahydrothiophene-3-carboxylate (**9a**)

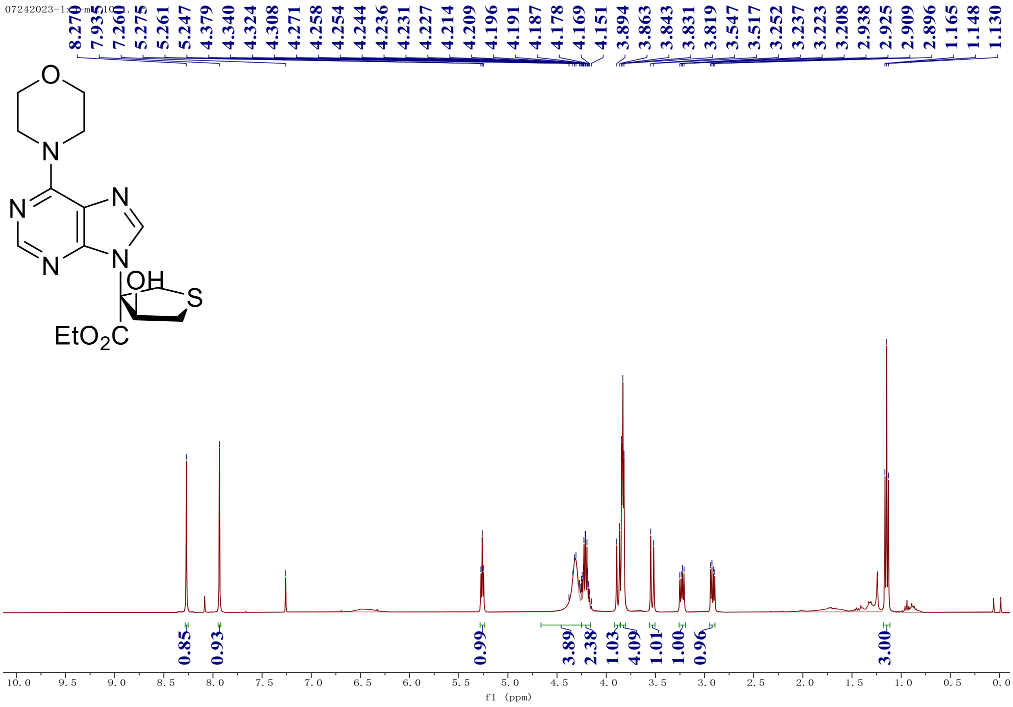

^{1}H NMR spectrum

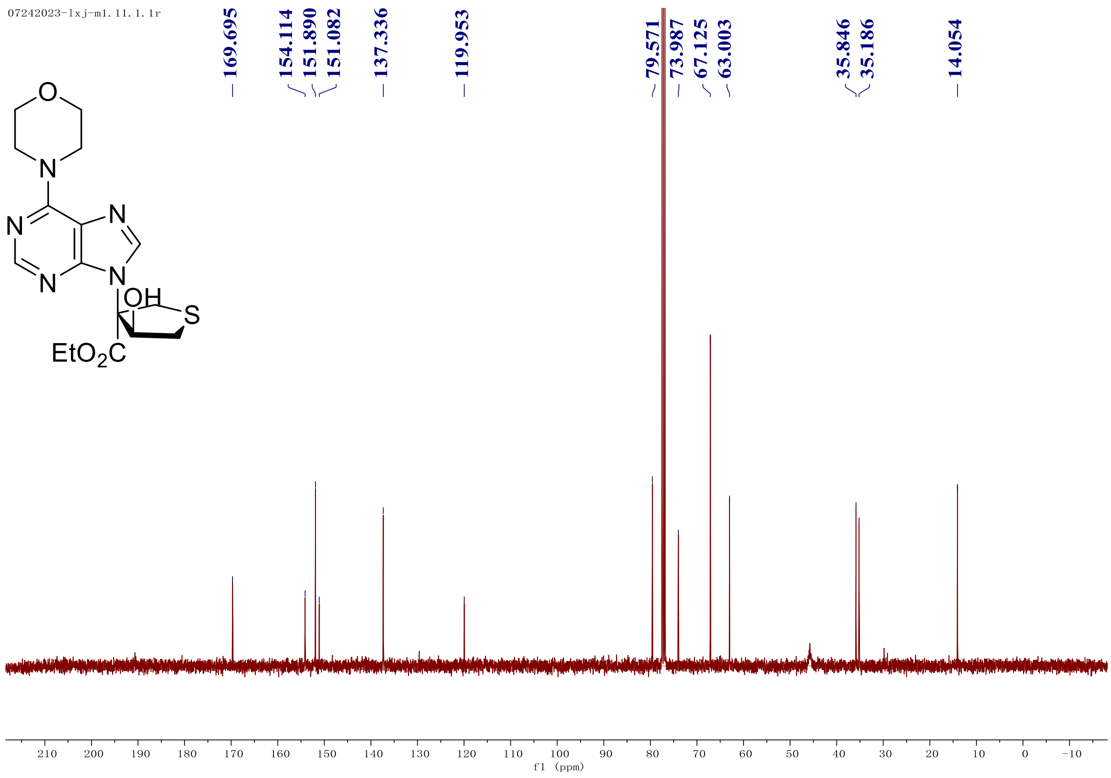

^{13}C NMR spectrum

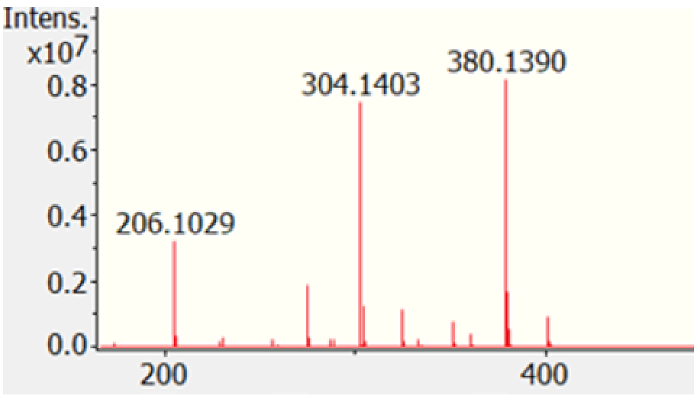

HR-ESIMS spectrum

(±)Ethyl-4-hydroxy-3-(6-morpholino-9*H*-purin-9-yl)tetrahydrothiophene-3-carboxylate (**9b**)

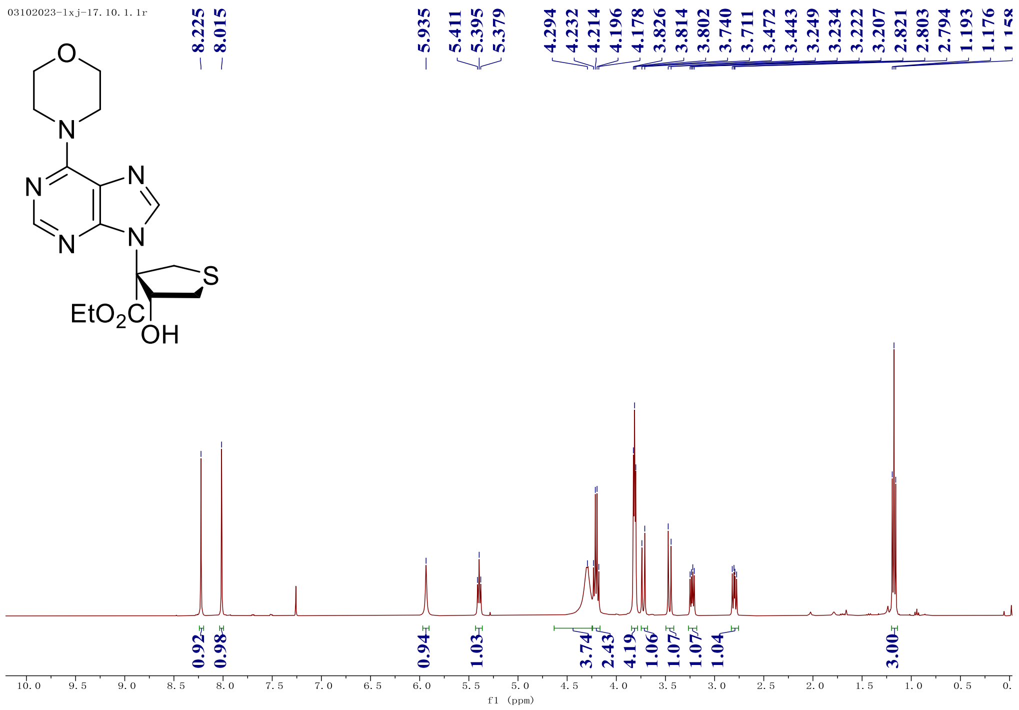

^{1}H NMR spectrum

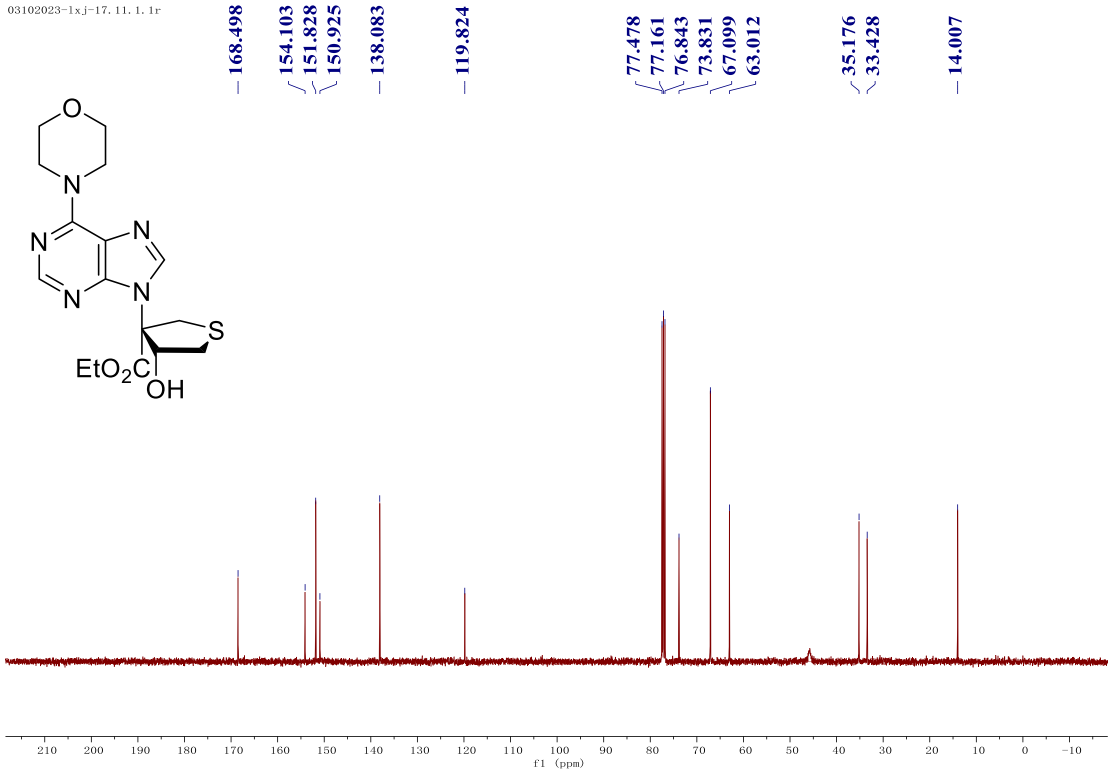

^{13}C NMR spectrum

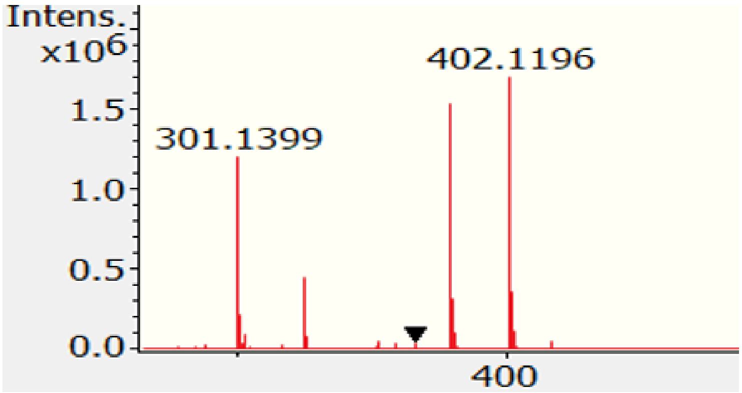

HR-ESIMS spectrum

(±)Ethyl-4-hydroxy-3-(6-thiomorpholino-9*H*-purin-9-yl)tetrahydrothiophene-3-carboxylate (**10a**)

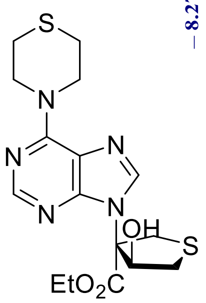

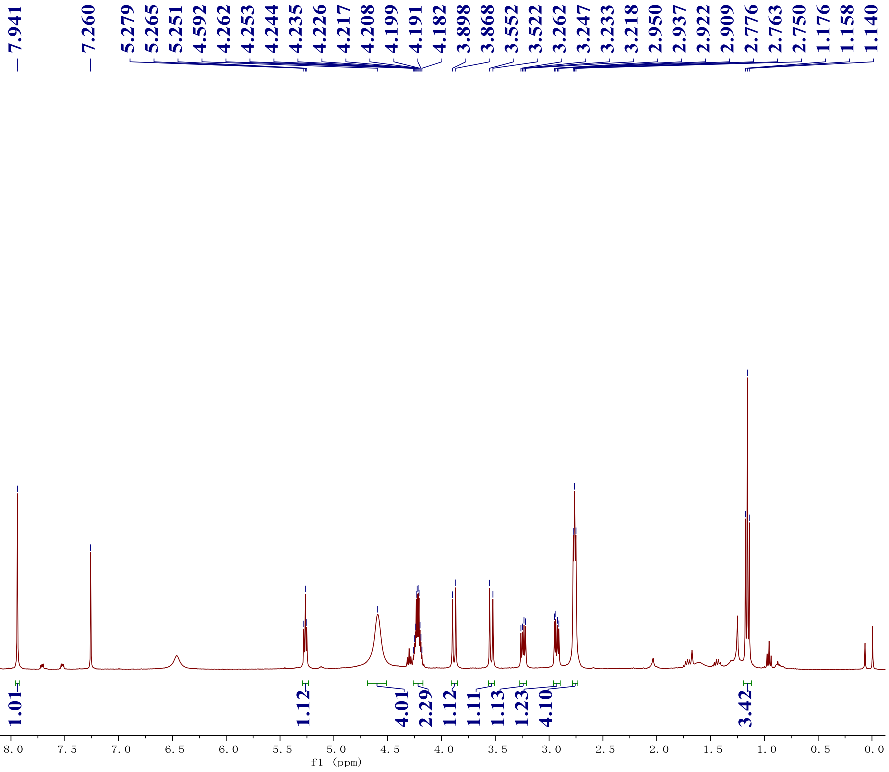

^{1}H NMR spectrum

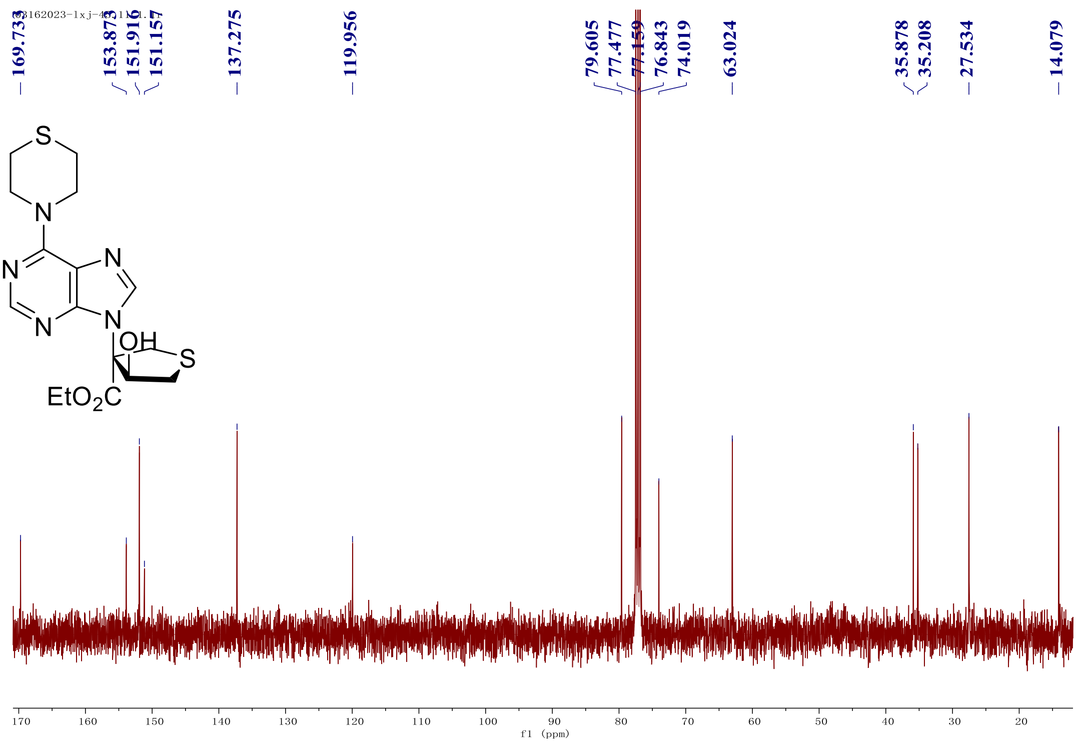

^{13}C NMR spectrum

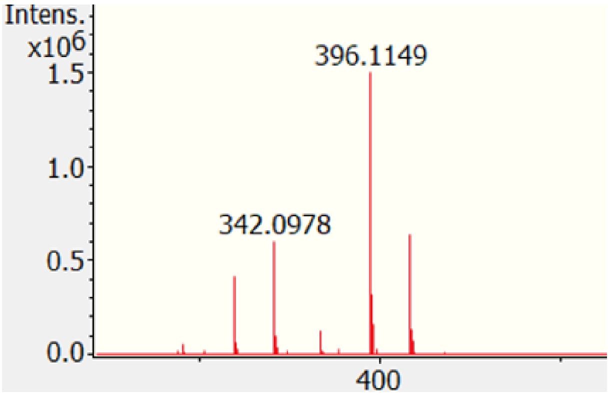

HR-ESIMS spectrum

(±)Ethyl-4-hydroxy-3-(6-thiomorpholino-9*H*-purin-9-yl)tetrahydrothiophene-3-carboxylate (**10b**)

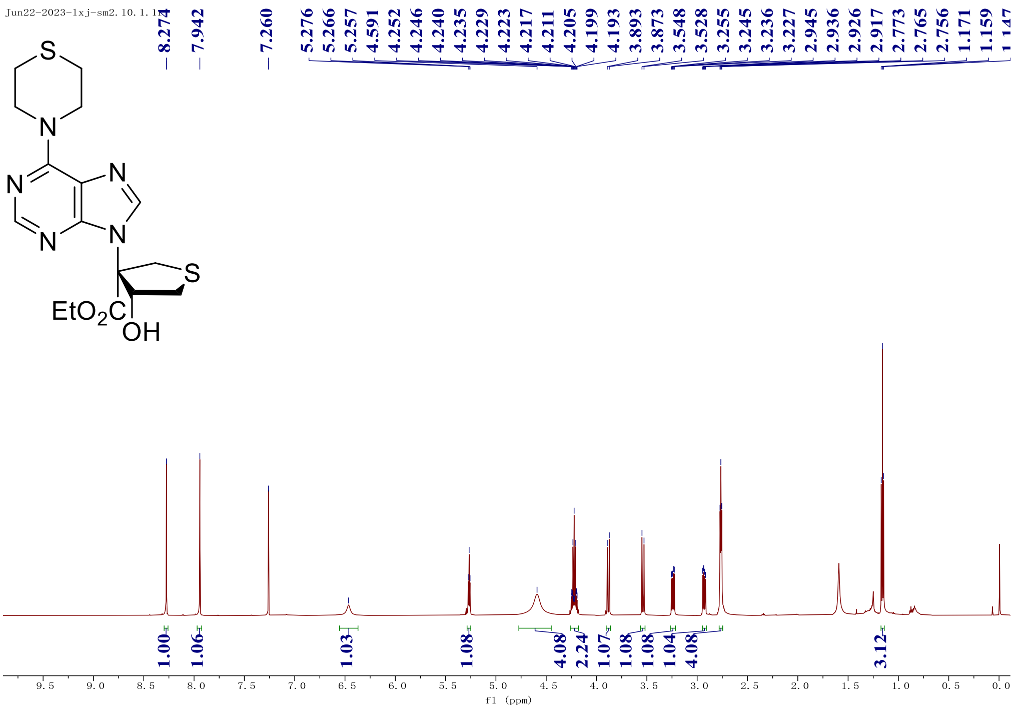

^{1}H NMR spectrum

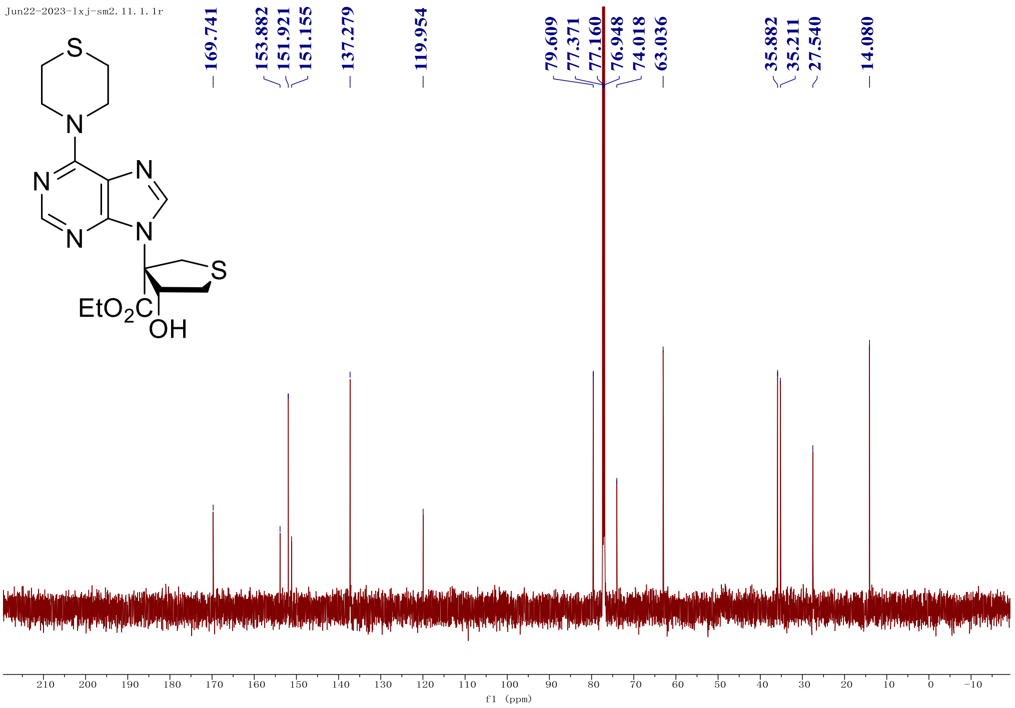

^{13}C NMR spectrum

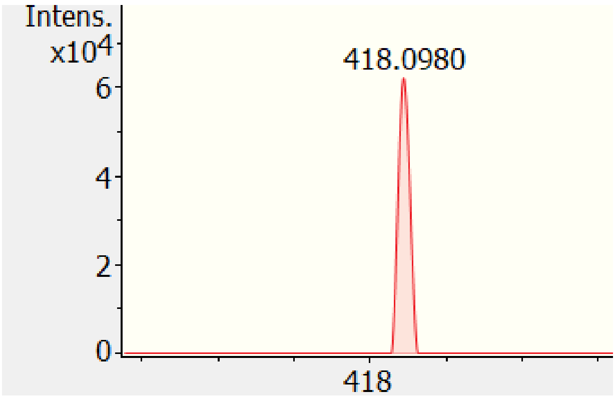

HR-ESIMS spectrum

(±)Ethyl-4-hydroxy-3-(6-(4-methylpiperidin-1-yl)-9*H*-purin-9-yl)tetrahydrothiophene-3-carboxylate (**11a**)

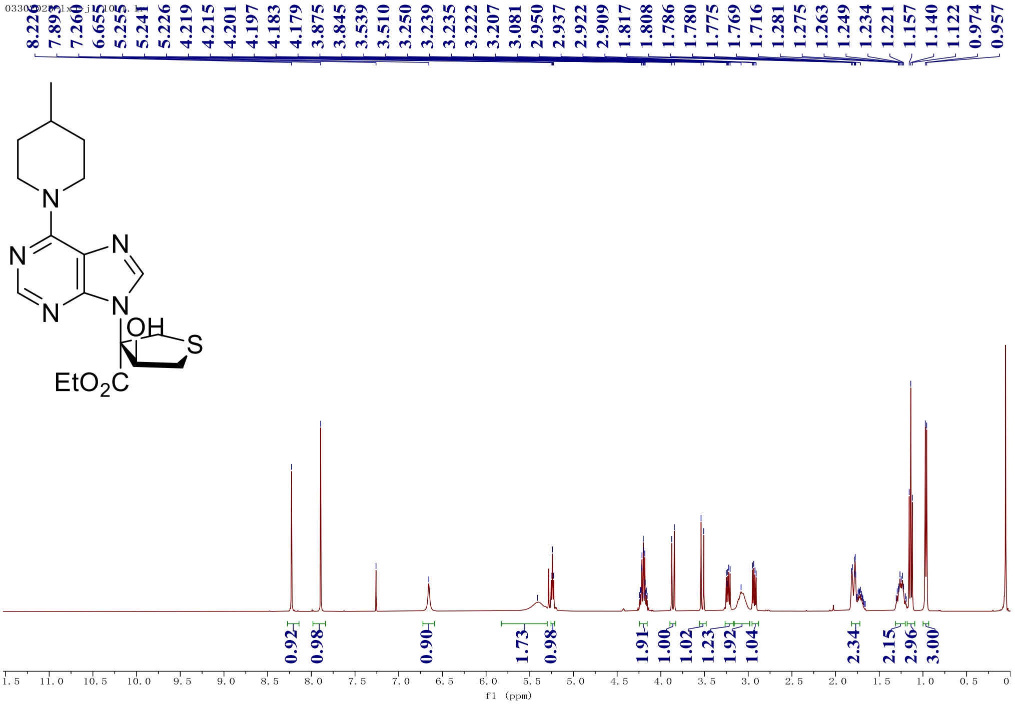

^{1}H NMR spectrum

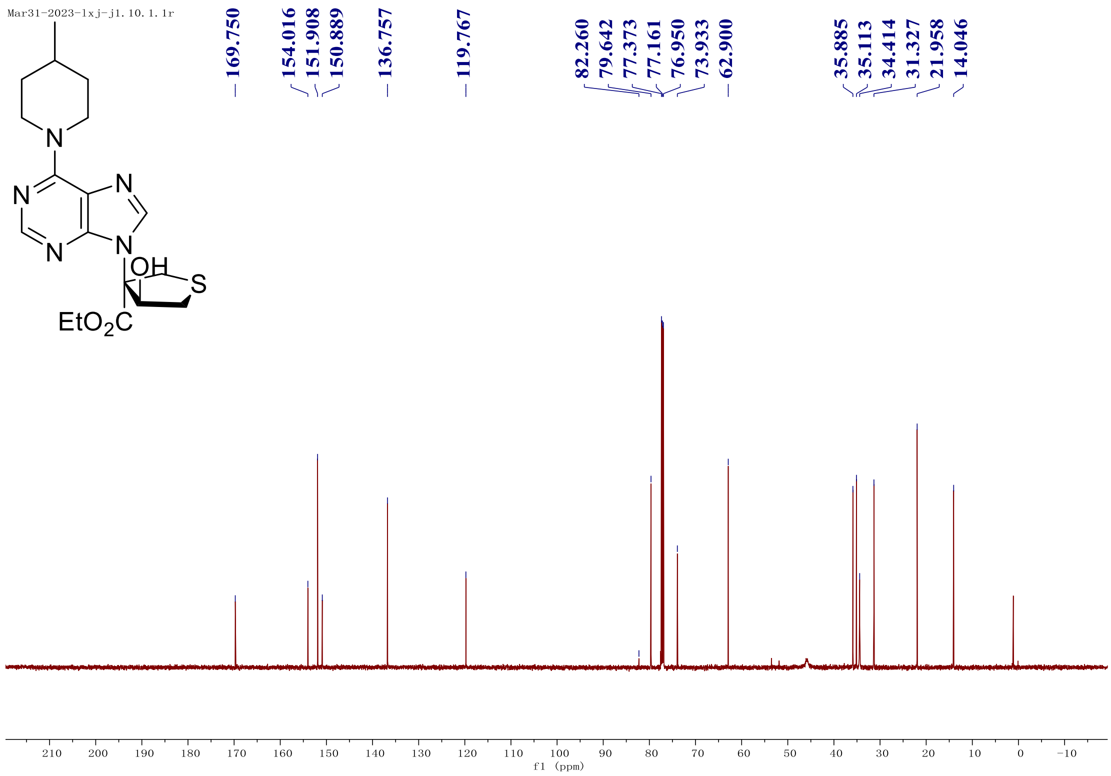

^{13}C NMR spectrum

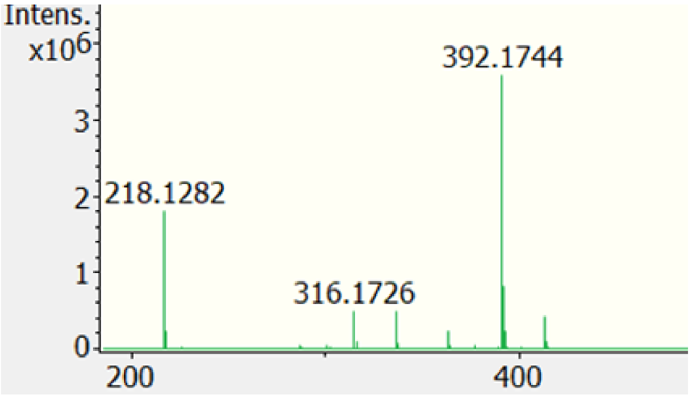

HR-ESIMS spectrum

(±)Ethyl-4-hydroxy-3-(6-(4-methylpiperidin-1-yl)-9*H*-purin-9-yl)tetrahydrothiophene-3-carboxylate (**11b**)

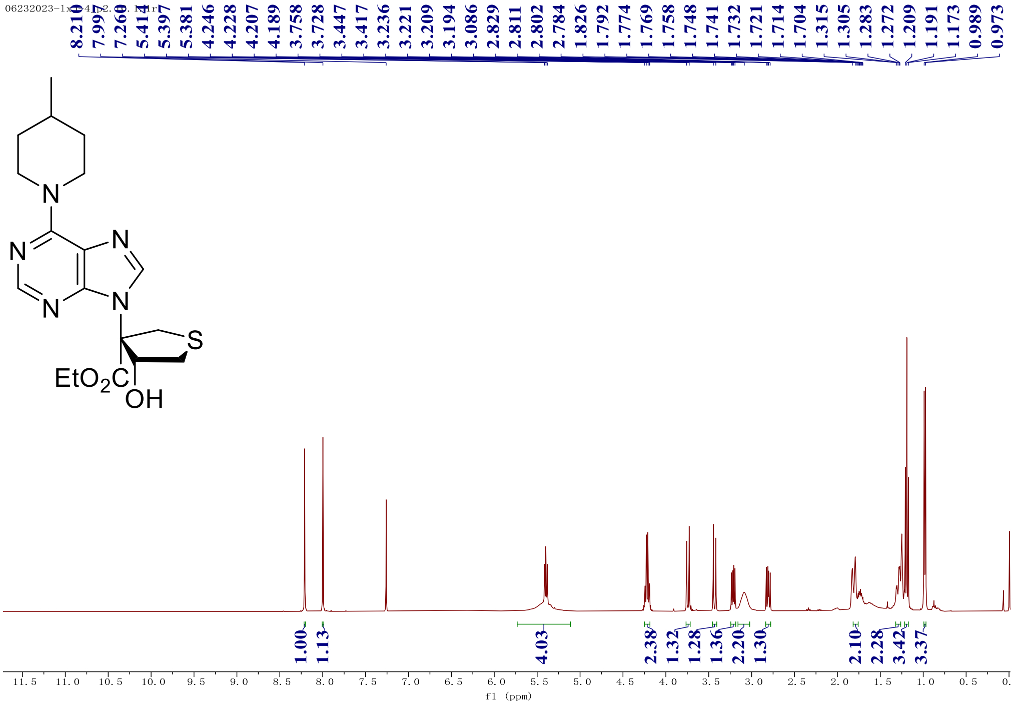

^{1}H NMR spectrum

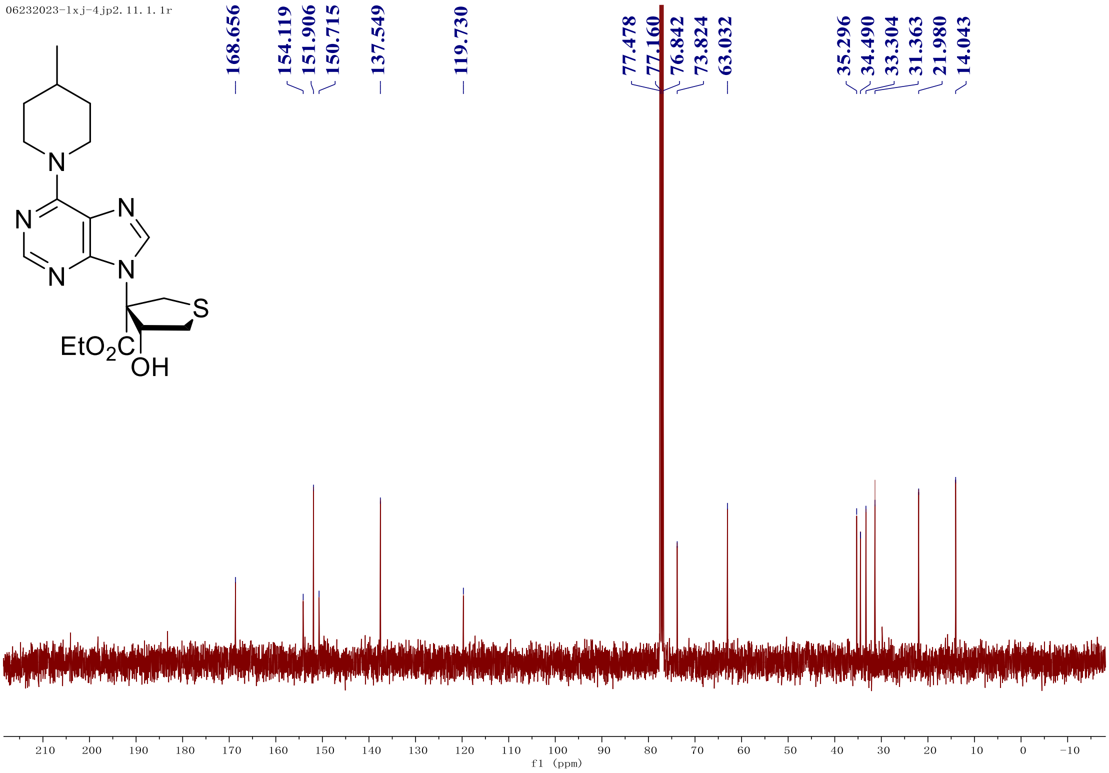

^{13}C NMR spectrum

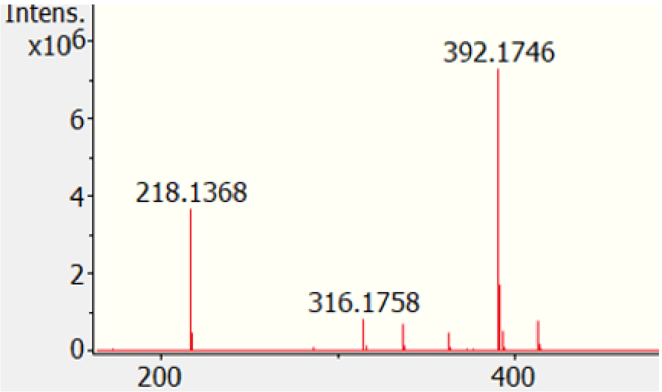

HR-ESIMS spectrum

(±)Ethyl-3-(6-(4-fluoropiperidin-1-yl)-9*H*-purin-9-yl)-4-hydroxytetrahydrothiophene-3-carboxylate (**12a**)

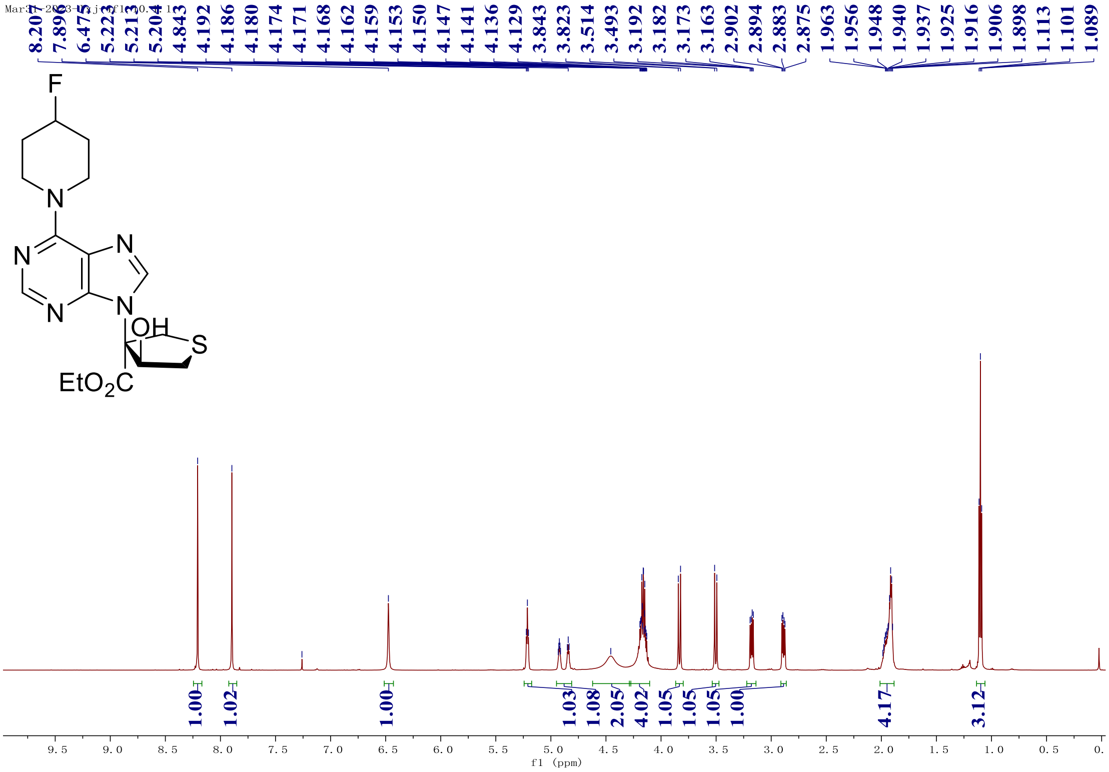

^{1}H NMR spectrum

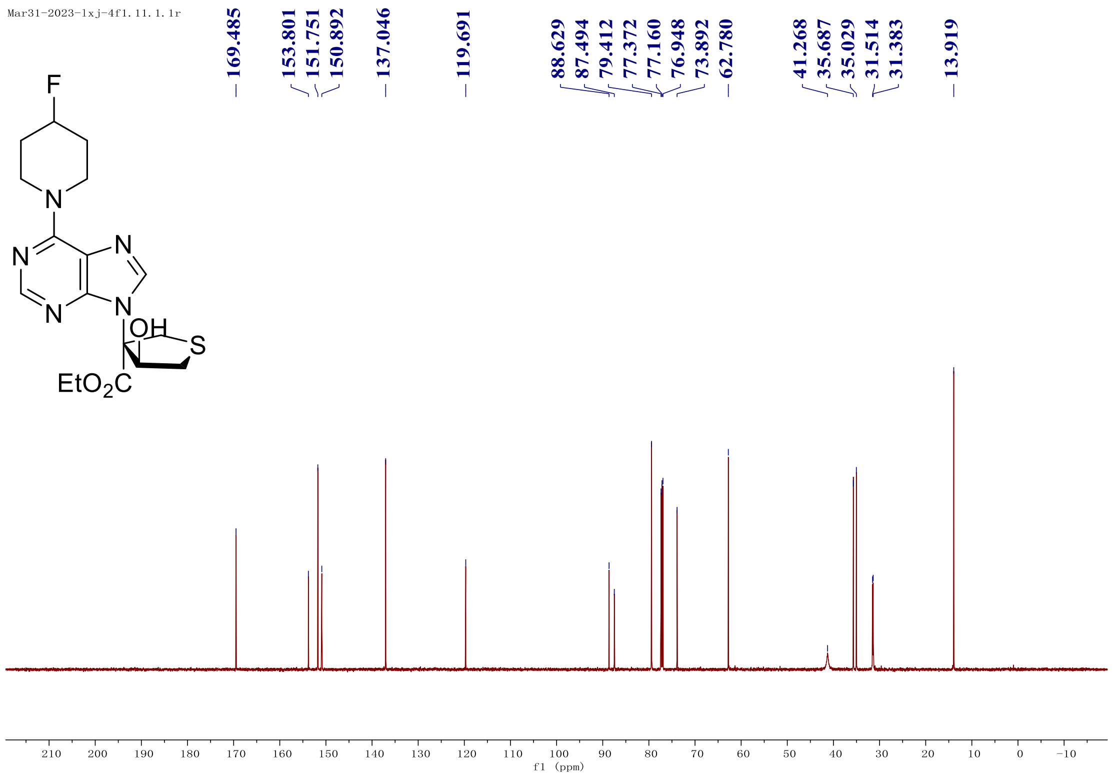

^{13}C NMR spectrum

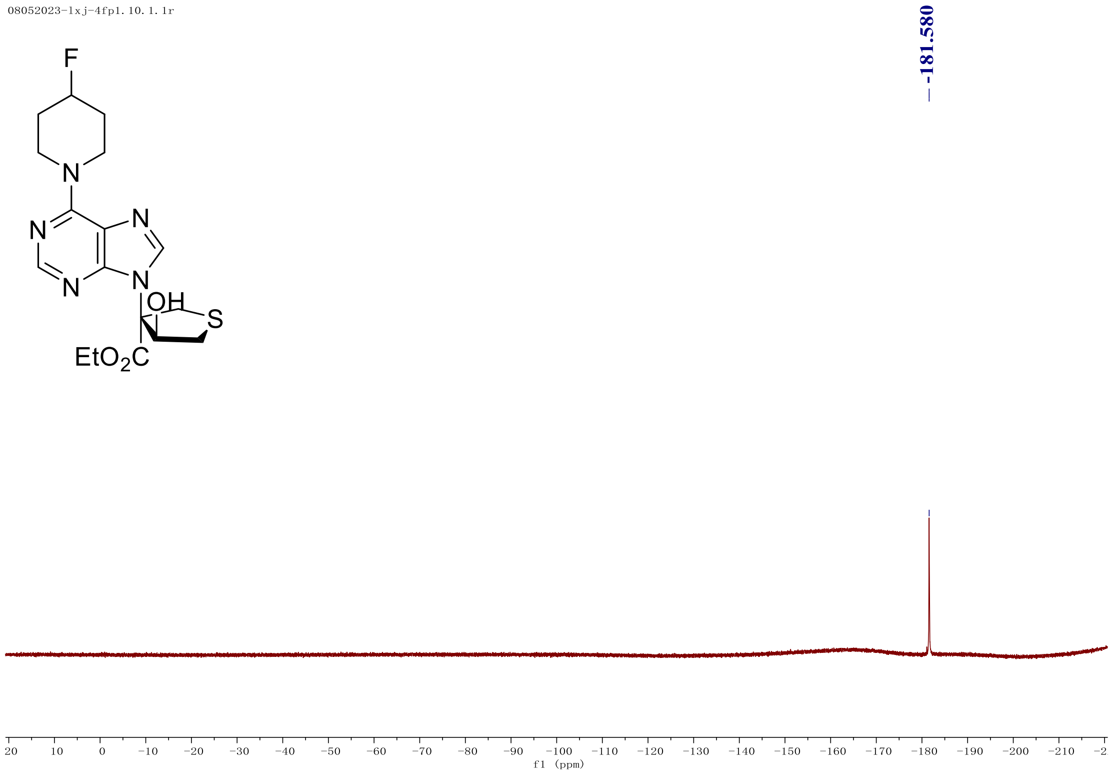

^{19}F NMR spectrum

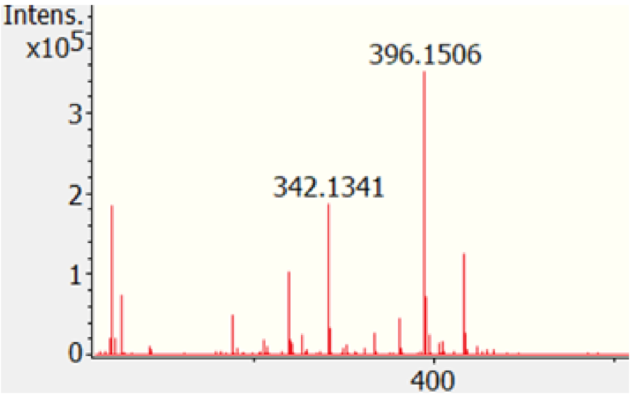

HR-ESIMS spectrum

(±)Ethyl-3-(6-(4-fluoropiperidin-1-yl)-9*H*-purin-9-yl)-4-hydroxytetrahydrothiophene-3-carboxylate (**12b**)

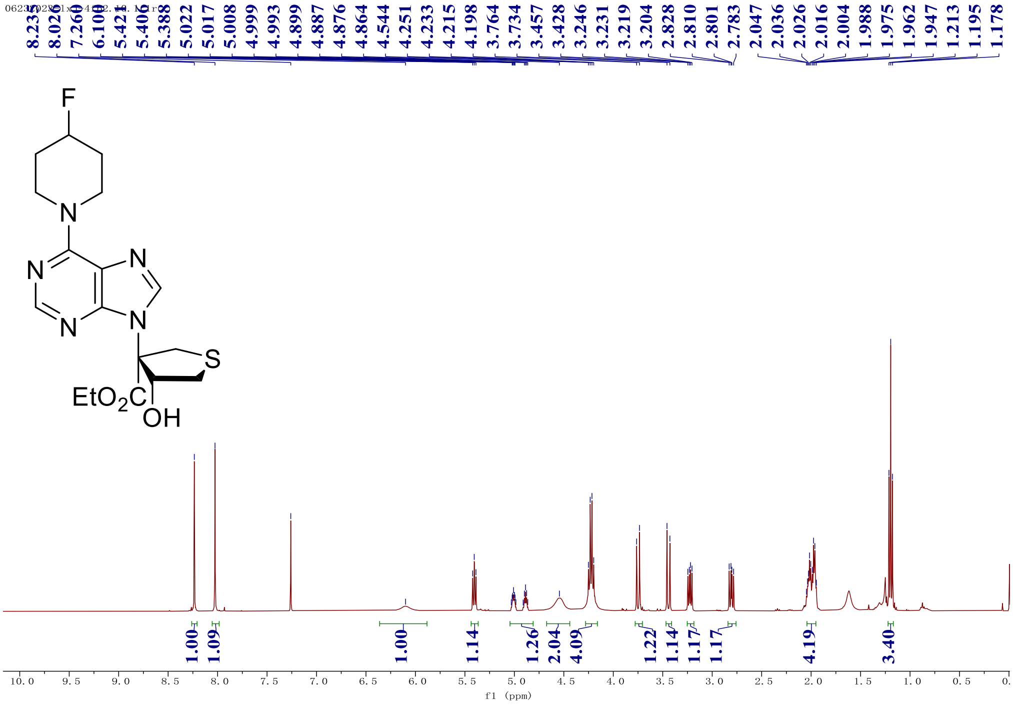

^{1}H NMR spectrum

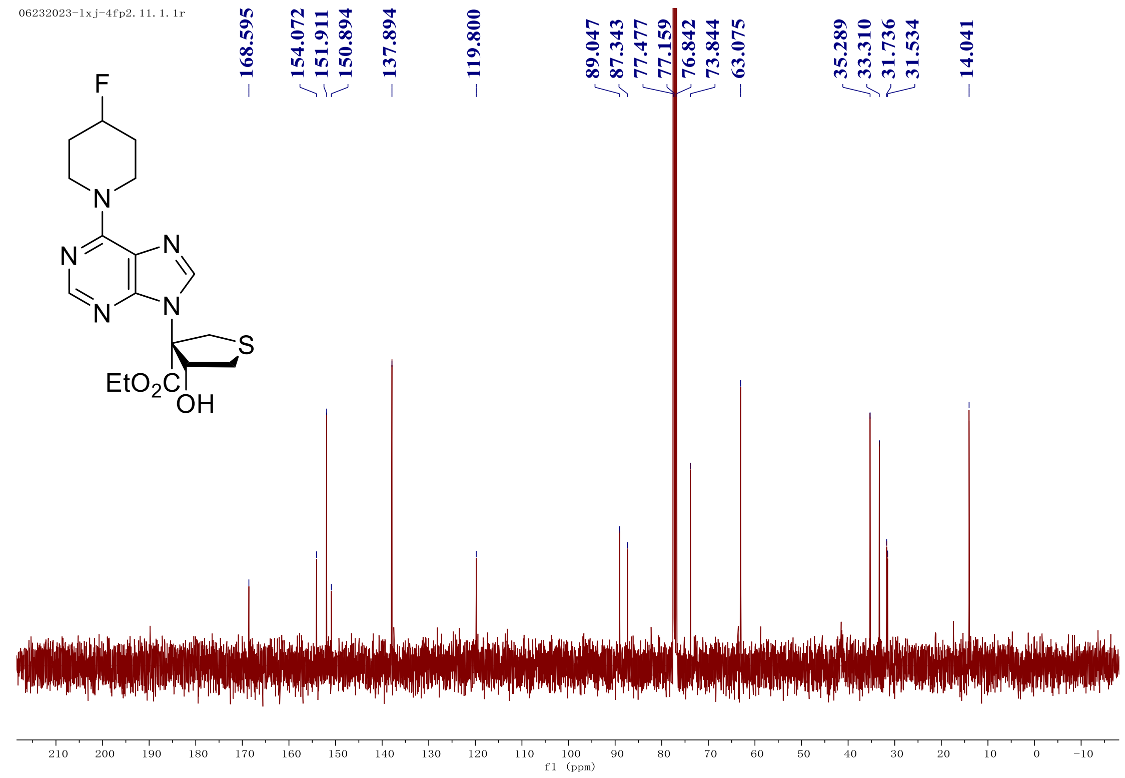

^{13}C NMR spectrum

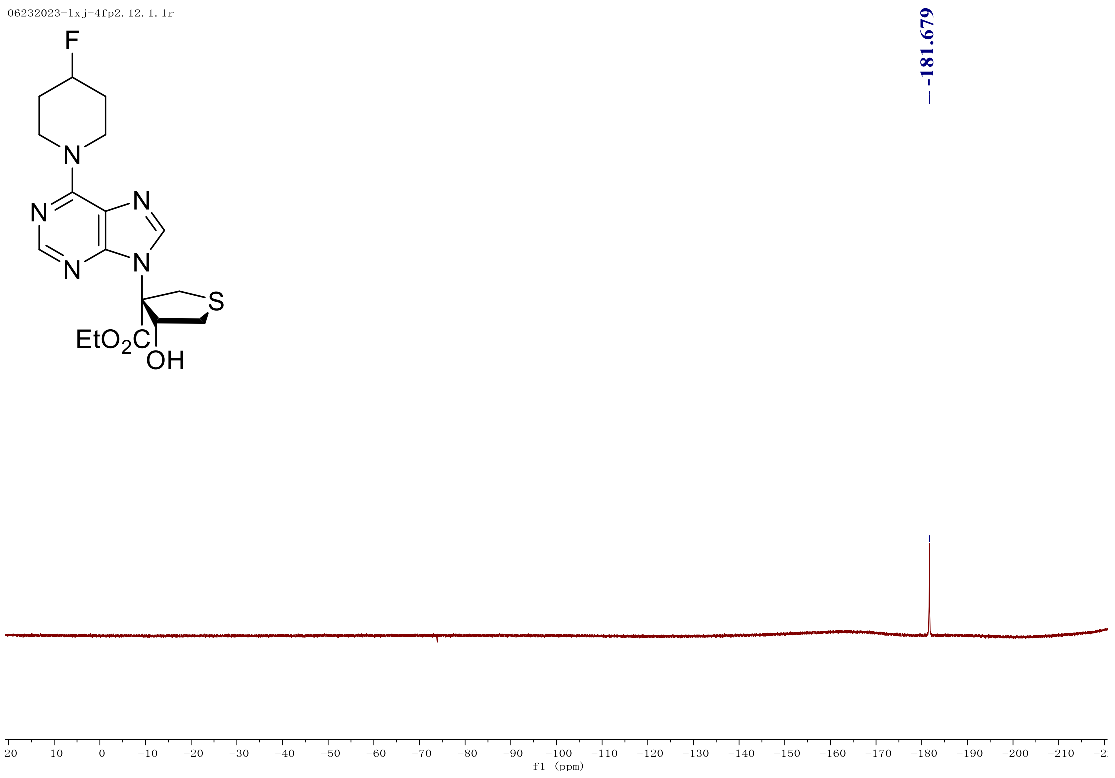

^{19}F NMR spectrum

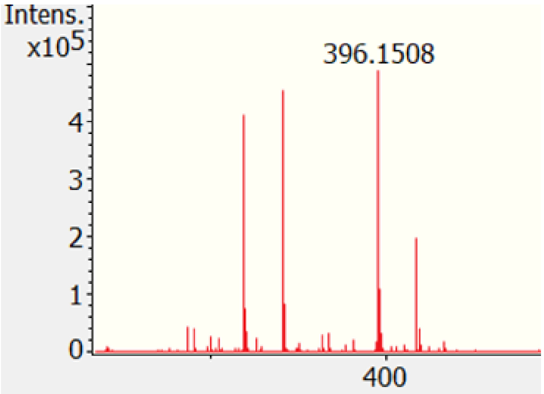

HR-ESIMS spectrum

(±)Ethyl-4-hydroxy-3-(6-(4-(trifluoromethyl)piperidin-1-yl)-9*H*-purin-9-yl)tetrahydrothiophene-3-carboxylate (**13a**)

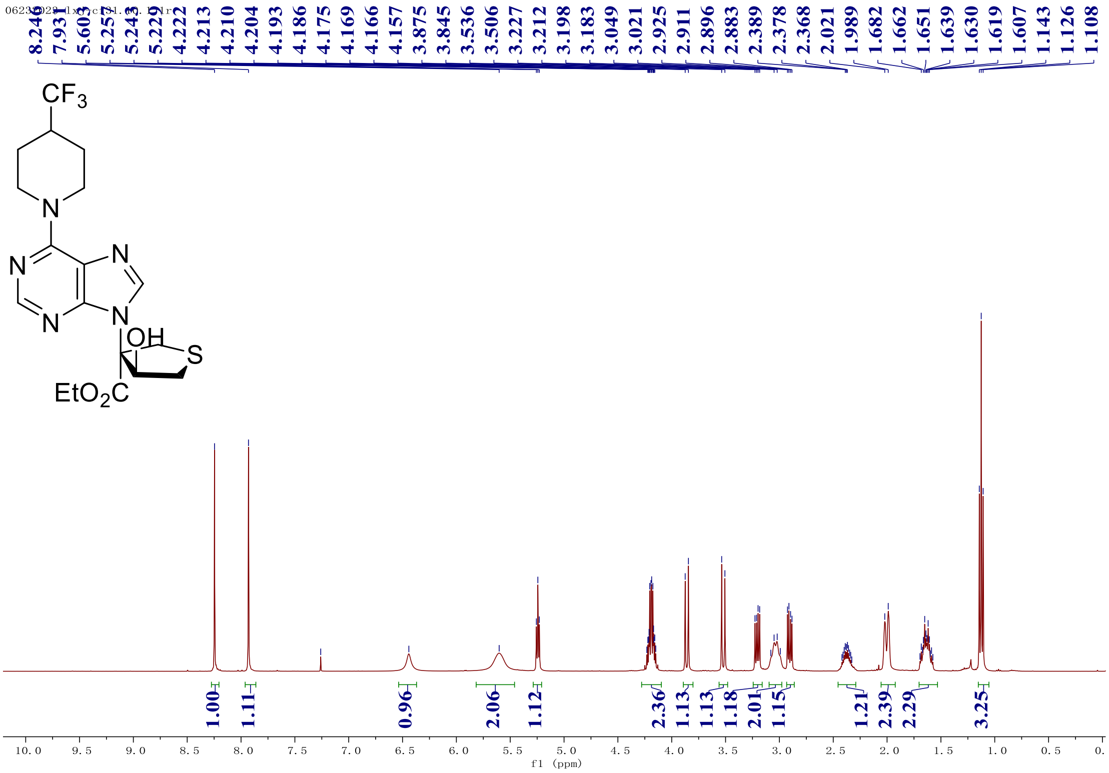

^{1}H NMR spectrum

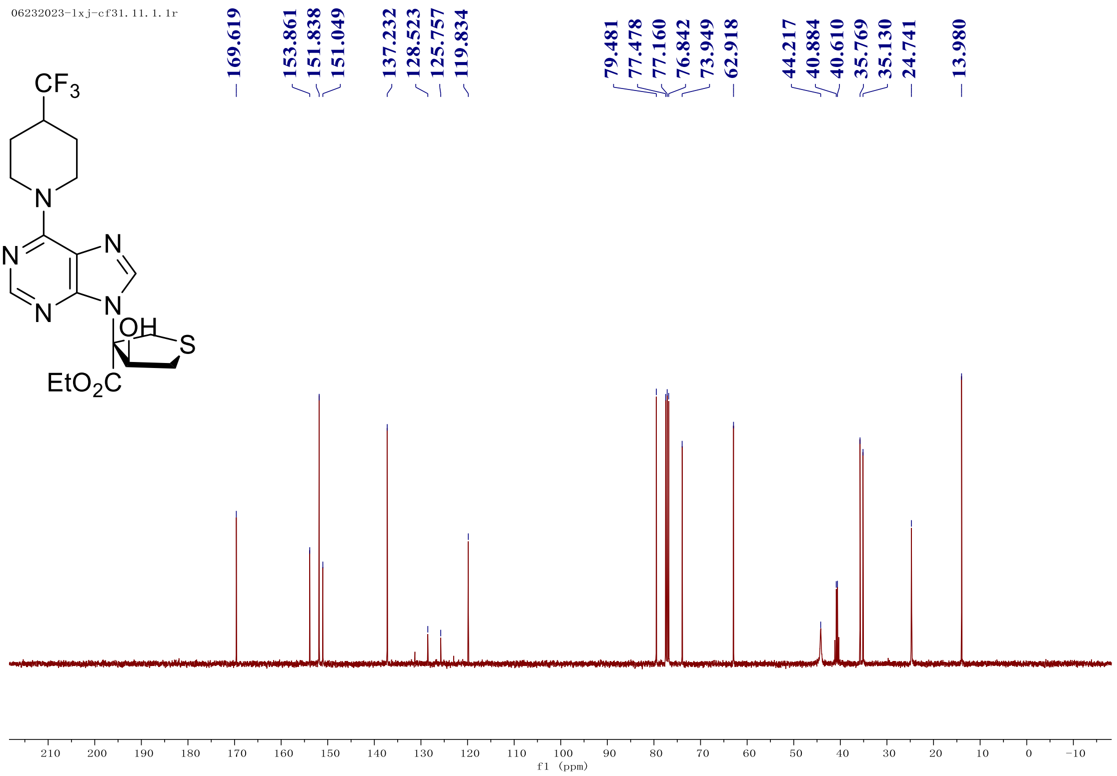

^{13}C NMR spectrum

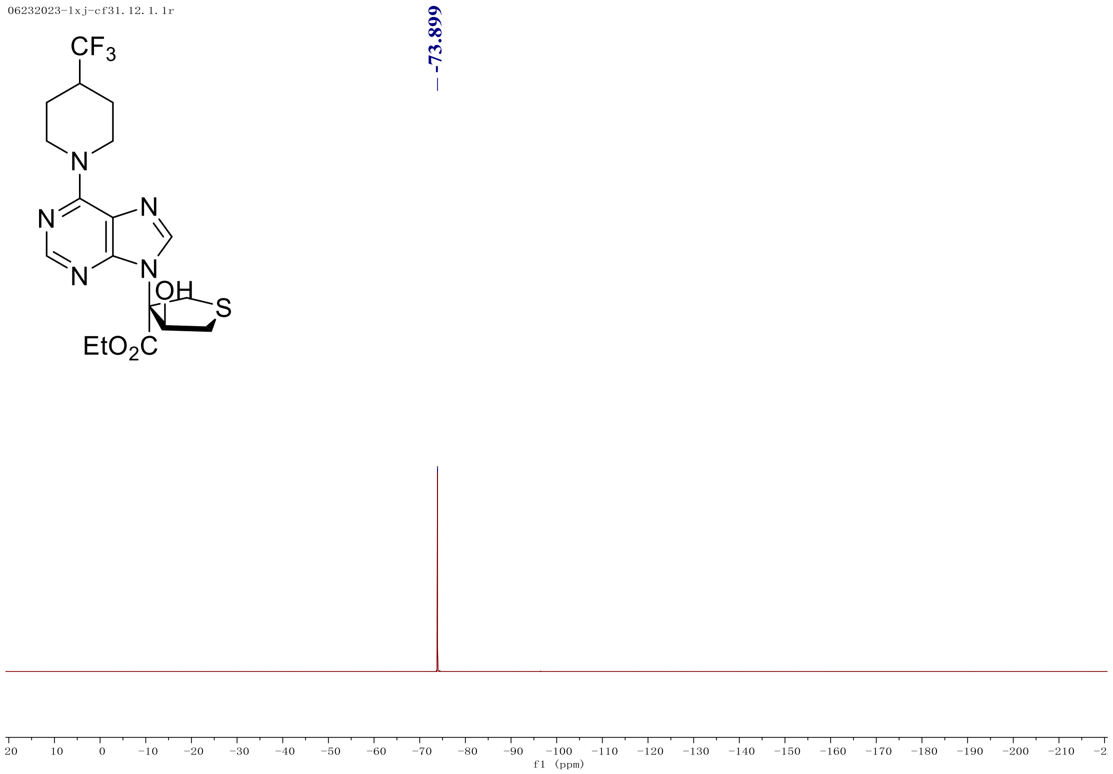

^{19}F NMR spectrum

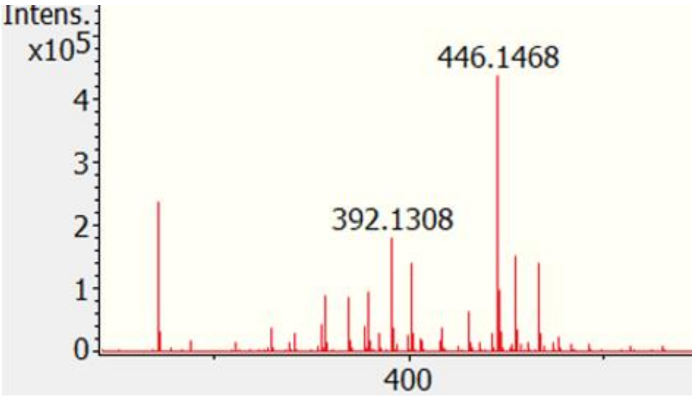

HR-ESIMS spectrum

(±)Ethyl-4-hydroxy-3-(6-(4-(trifluoromethyl)piperidin-1-yl)-9*H*-purin-9-yl)tetrahydrothiophene-3-carboxylate (**13b**)

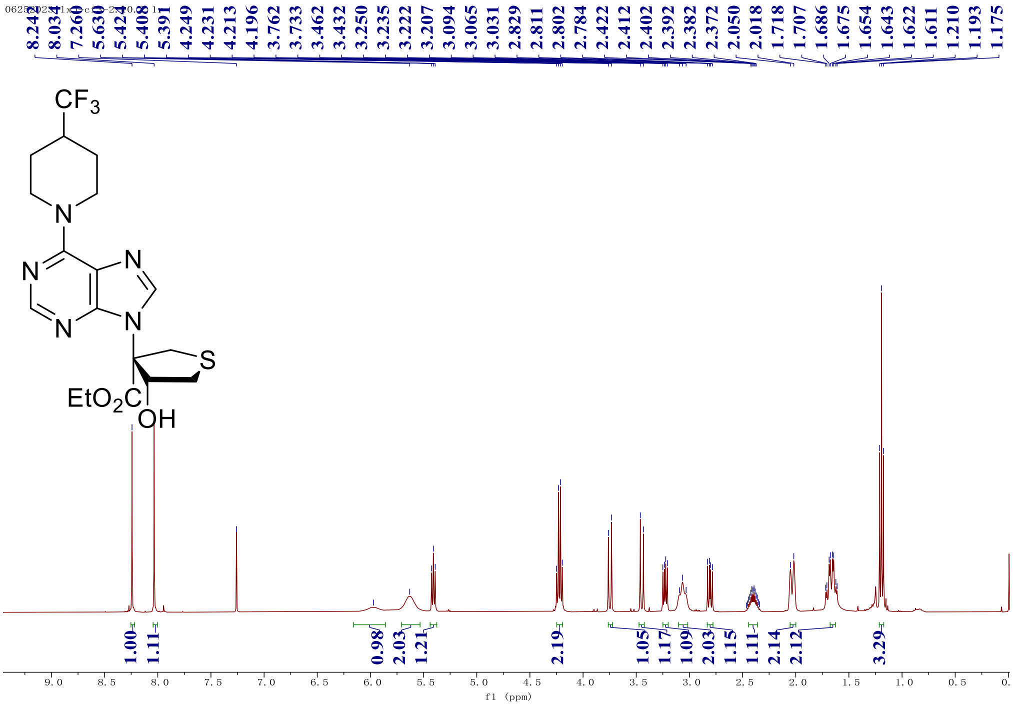

^{1}H NMR spectrum

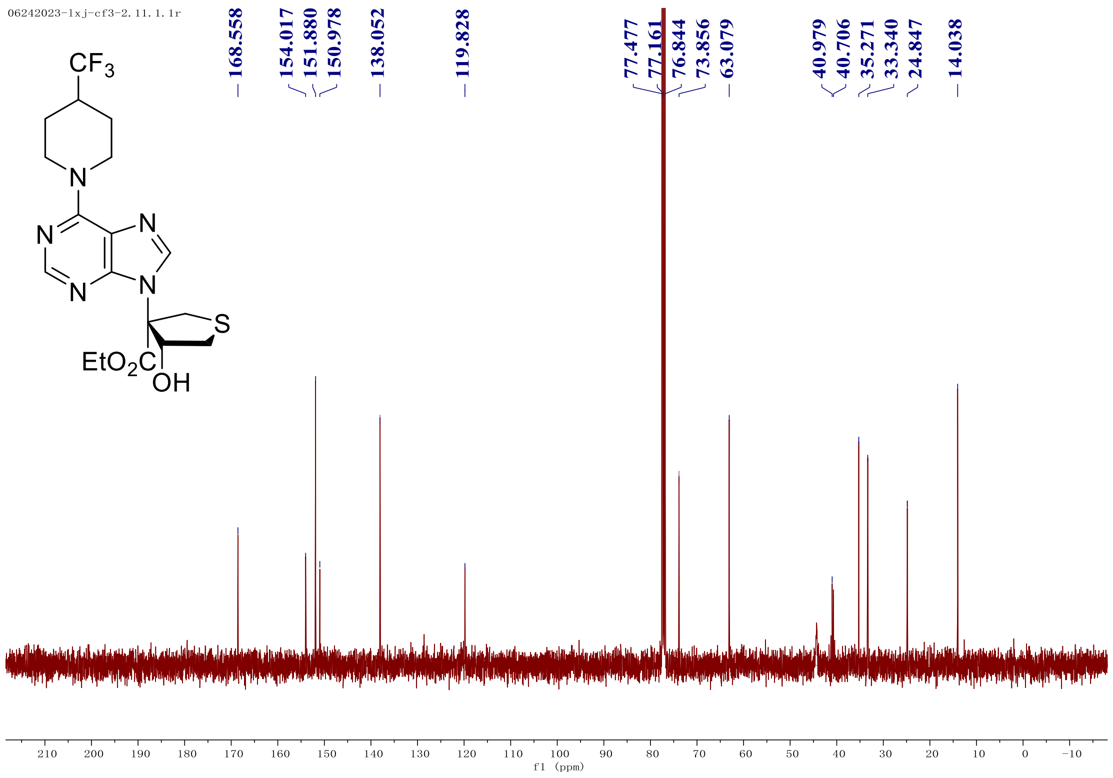

^{13}C NMR spectrum

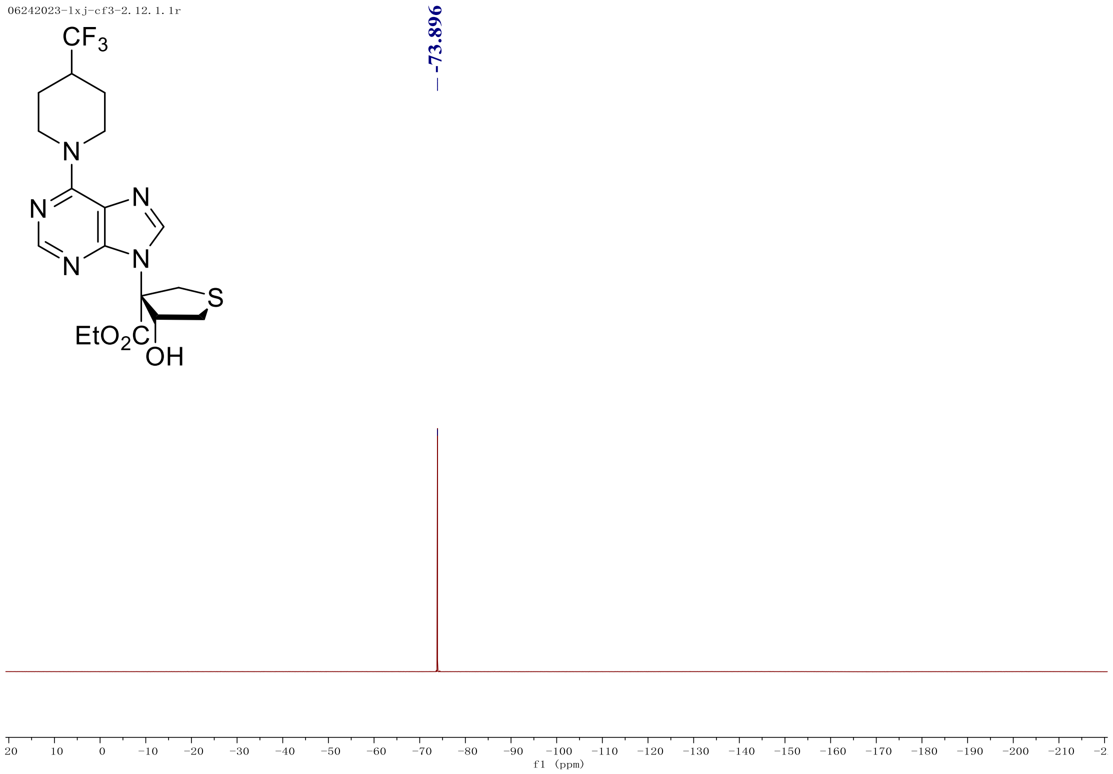

^{19}F NMR spectrum

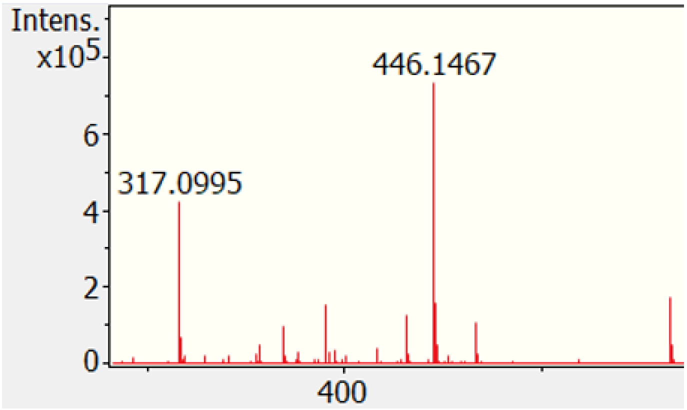

HR-ESIMS spectrum

(±)Ethyl-3-(6-(4-chloropiperidin-1-yl)-9*H*-purin-9-yl)-4-hydroxytetrahydrothiophene-3-carboxylate (**14a**)

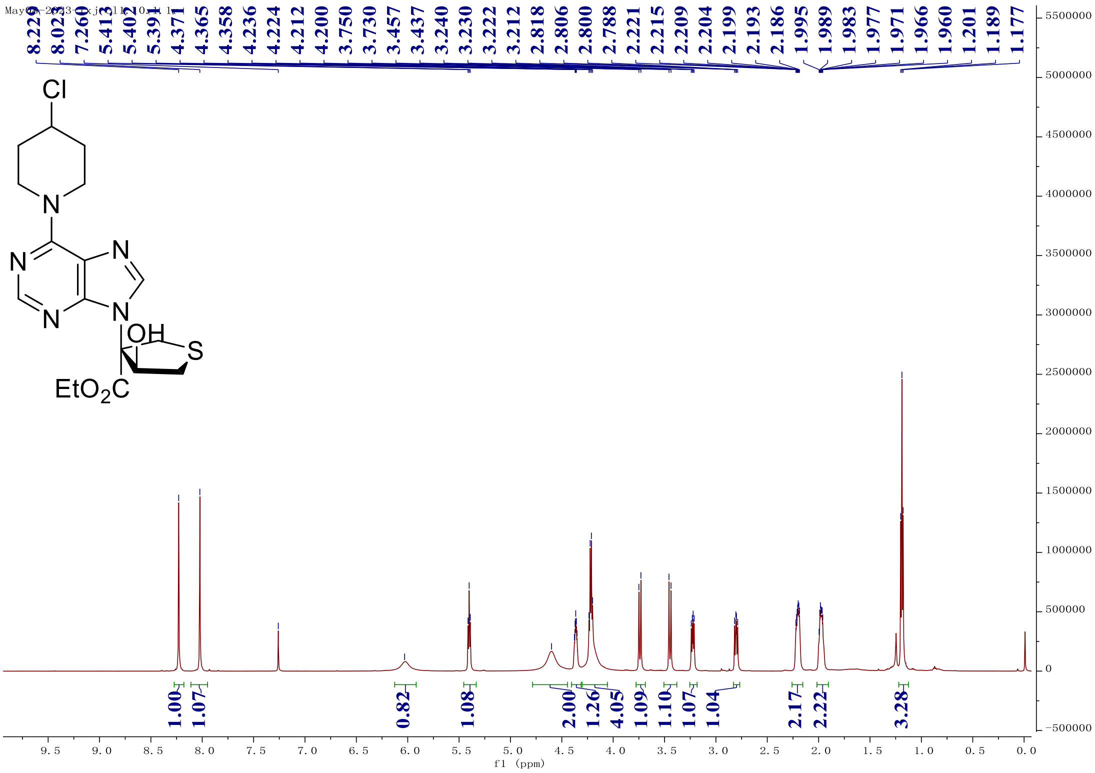

^{1}H NMR spectrum

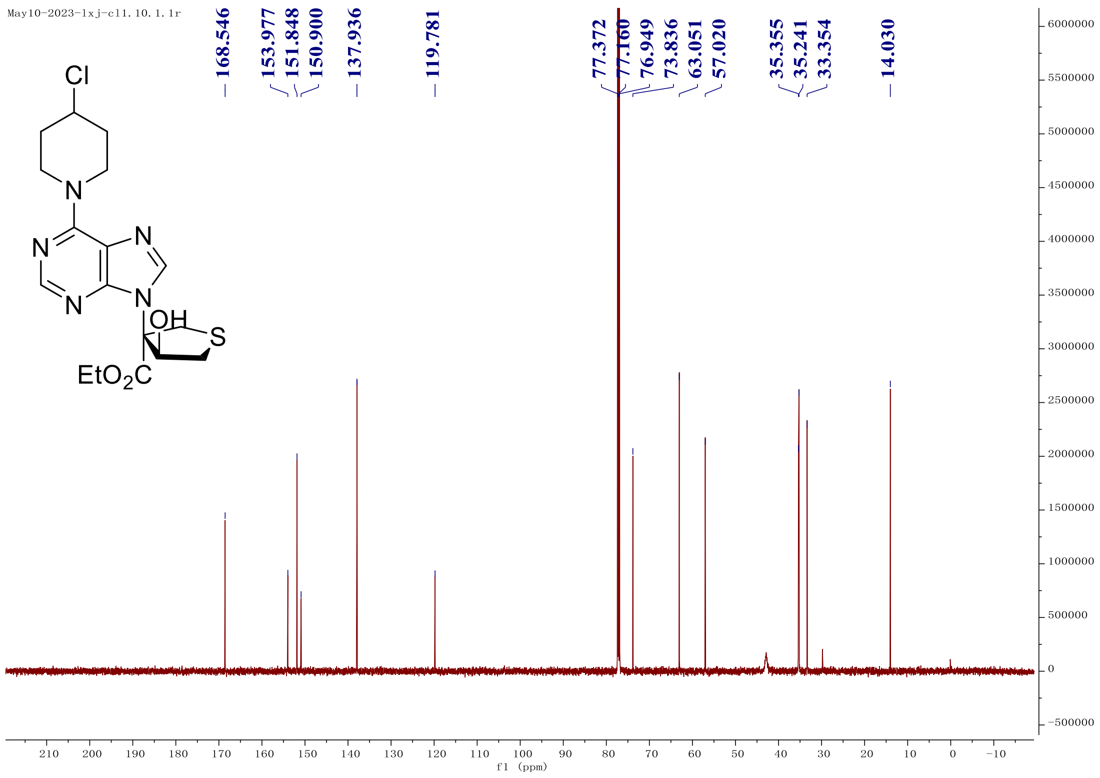

^{13}C NMR spectrum

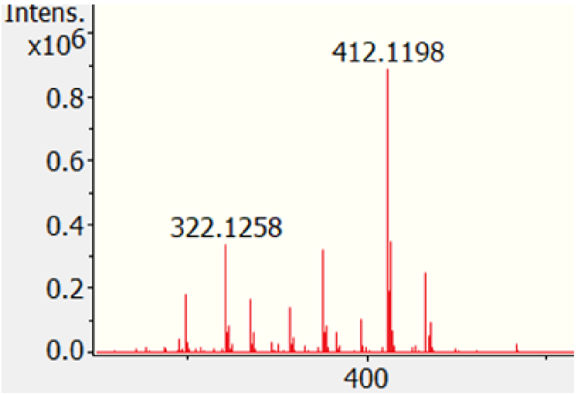

HR-ESIMS spectrum

(±)Thyl-3-(6-(4-chloropiperidin-1-yl)-9*H*-purin-9-yl)-4-hydroxytetrahydrothiophene-3-carboxylate (**14b**)

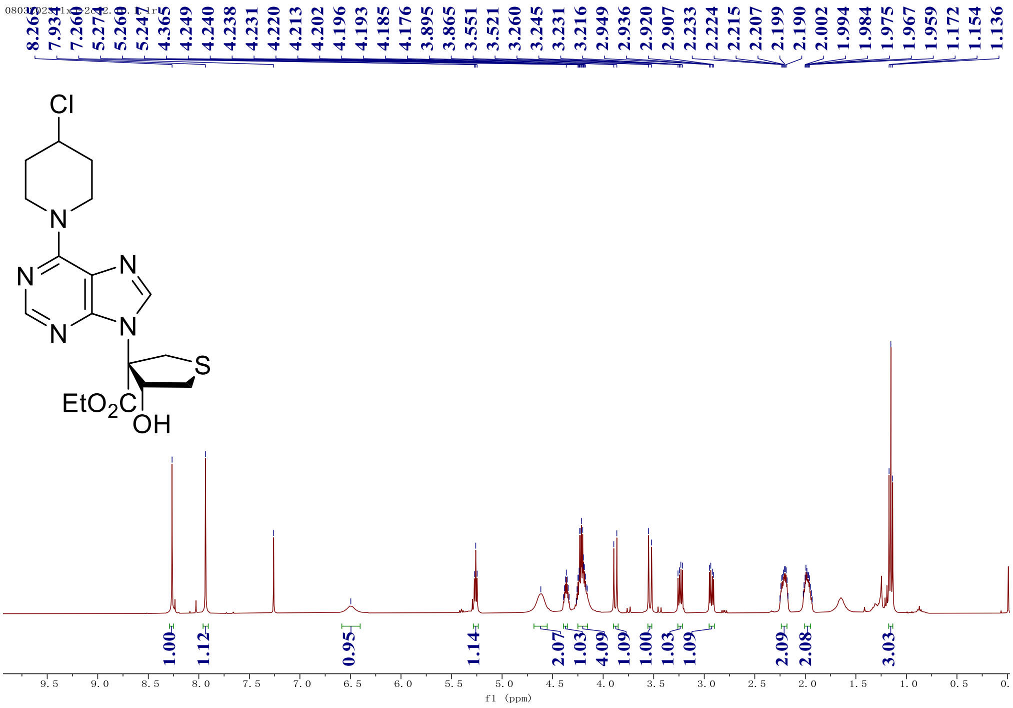

^{1}H NMR spectrum

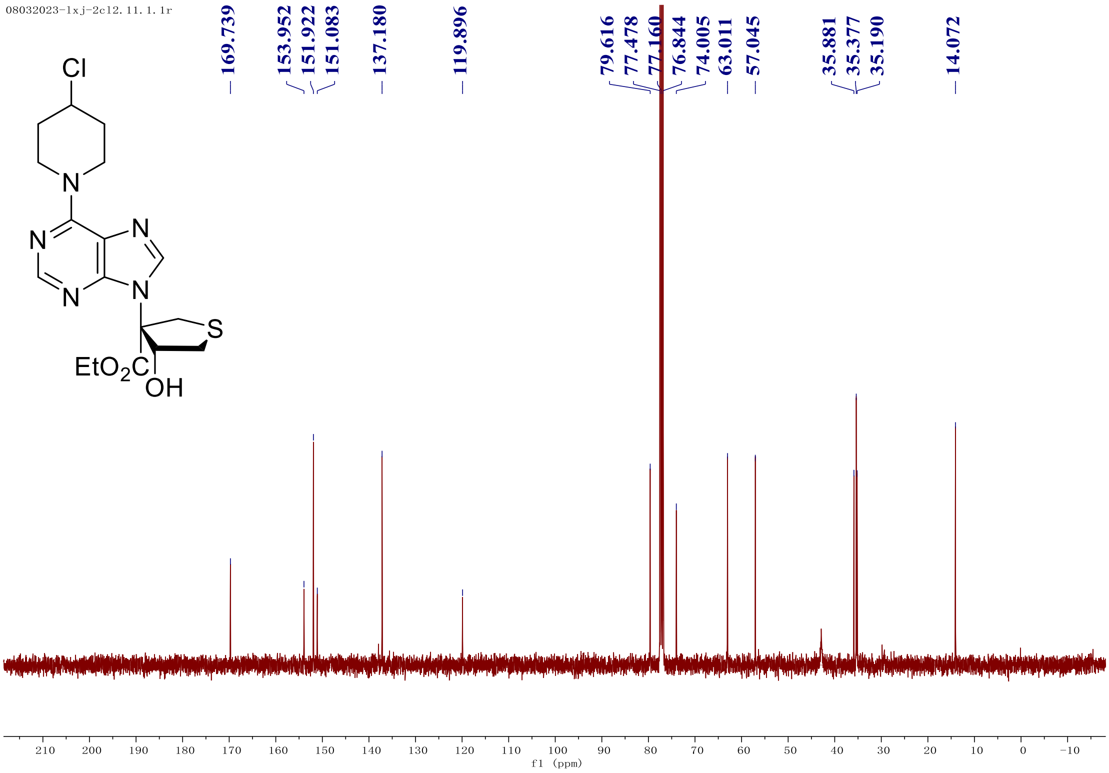

^{13}C NMR spectrum

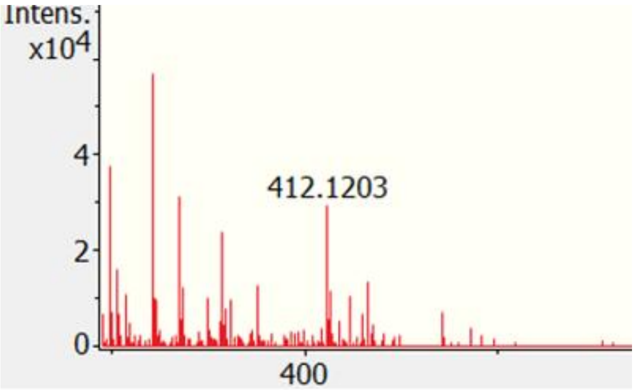

HR-ESIMS spectrum

(±)Ethyl-4-hydroxy-3-(6-(4-morpholinopiperidin-1-yl)-9*H*-purin-9-yl)tetrahydrothiophene-3-carboxylate (**15a**)

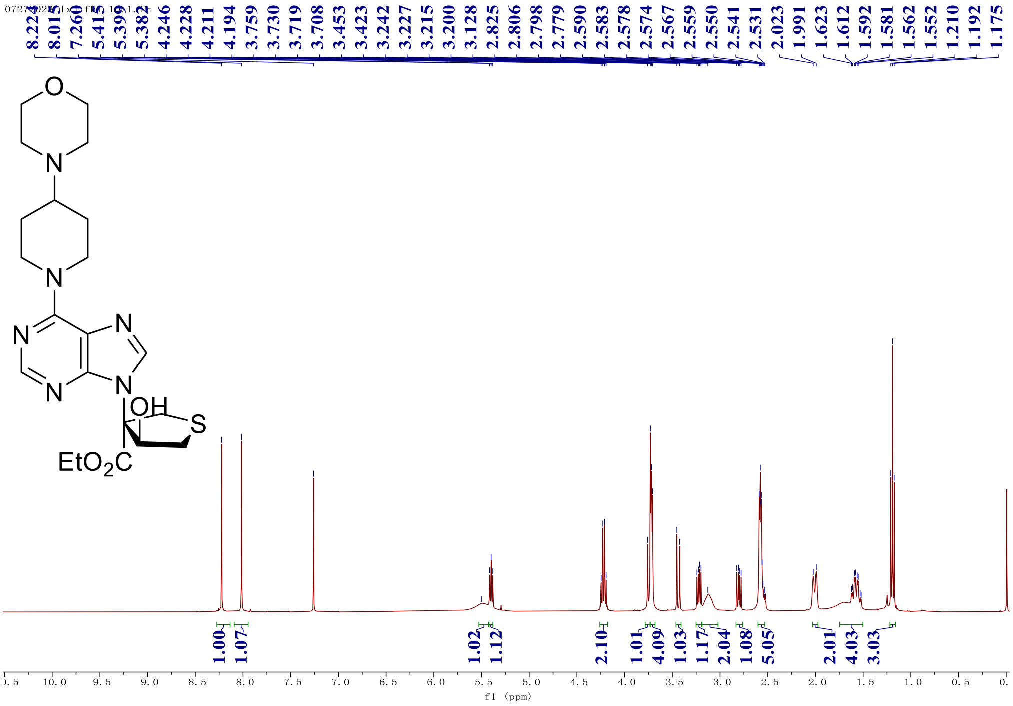

^{1}H NMR spectrum

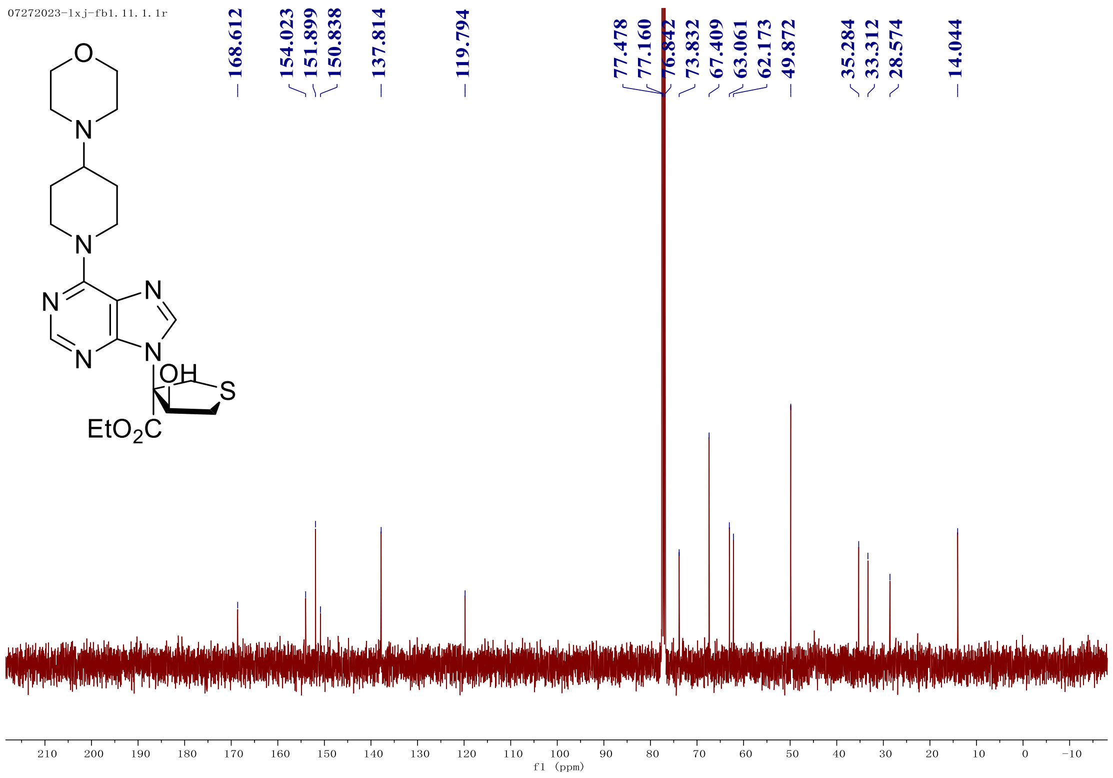

^{13}C NMR spectrum

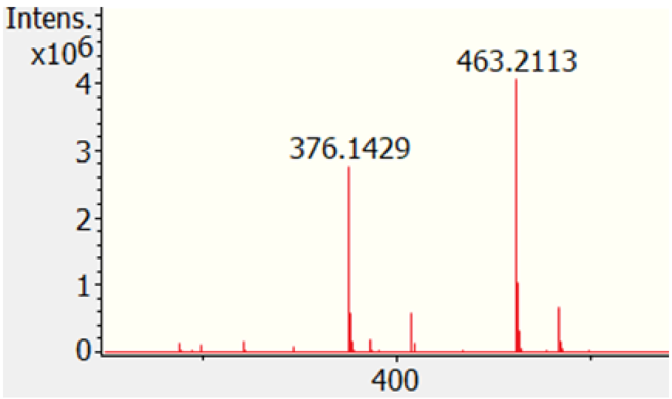

HR-ESIMS spectrum

(±)Ethyl-4-hydroxy-3-(6-(4-morpholinopiperidin-1-yl)-9*H*-purin-9-yl)tetrahydrothiophene-3-carboxylate (**15b**)

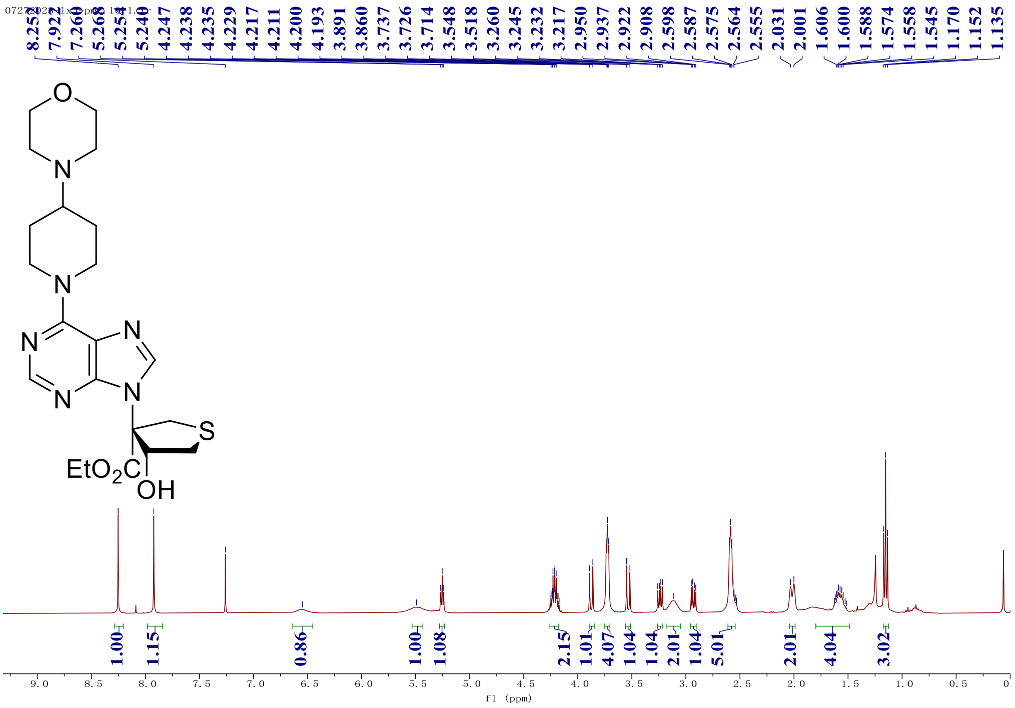

^{1}H NMR spectrum

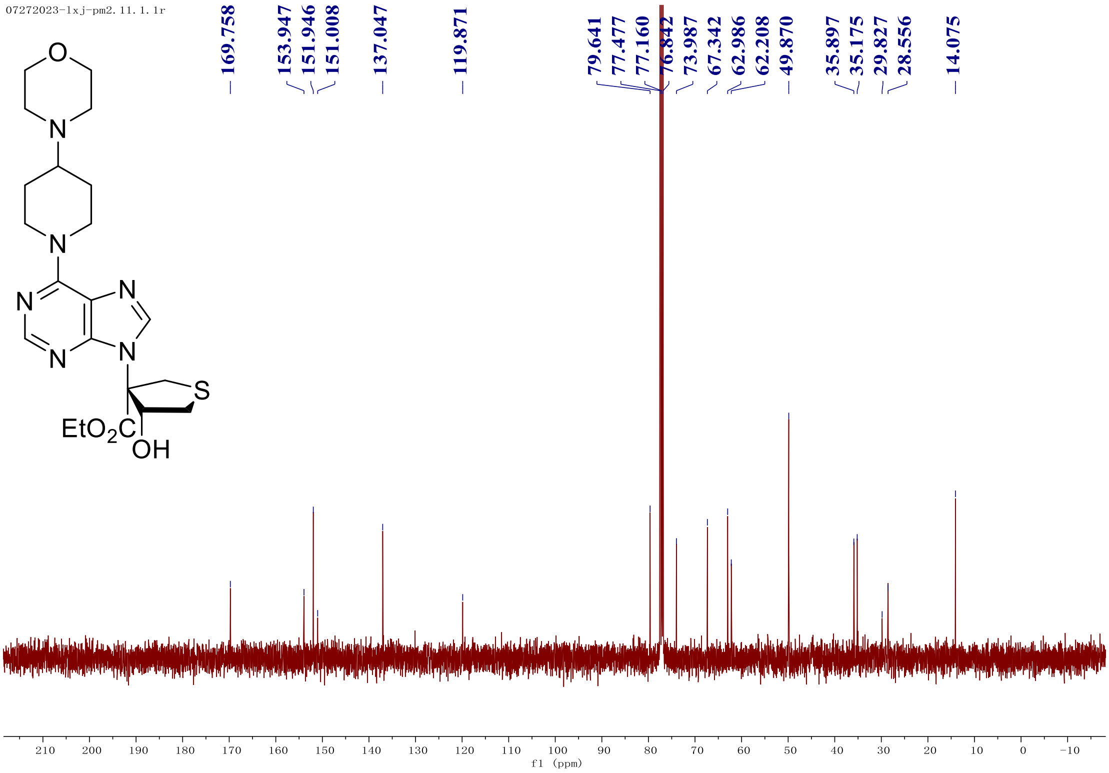

^{13}C NMR spectrum

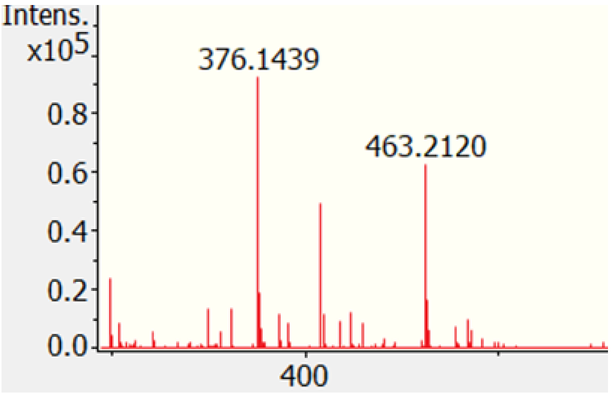

HR-ESIMS spectrum

(±)Thyl-3-(6-(4-benzylpiperidin-1-yl)-9*H*-purin-9-yl)-4-hydroxytetrahydrothiophene-3-carboxylate (**16a**)

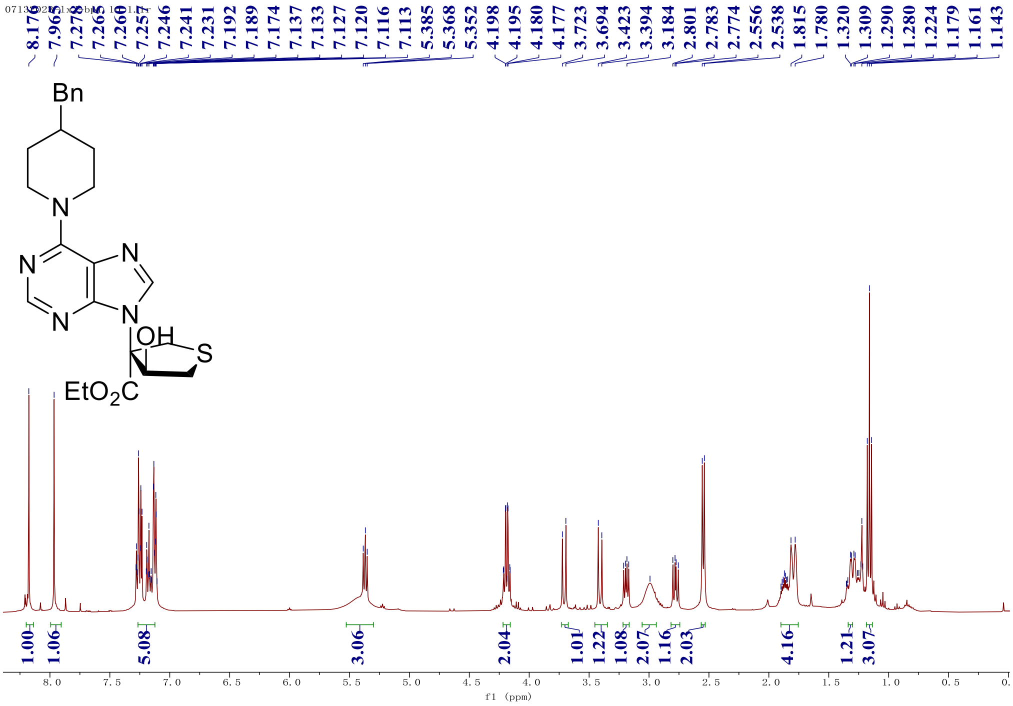

^{1}H NMR spectrum

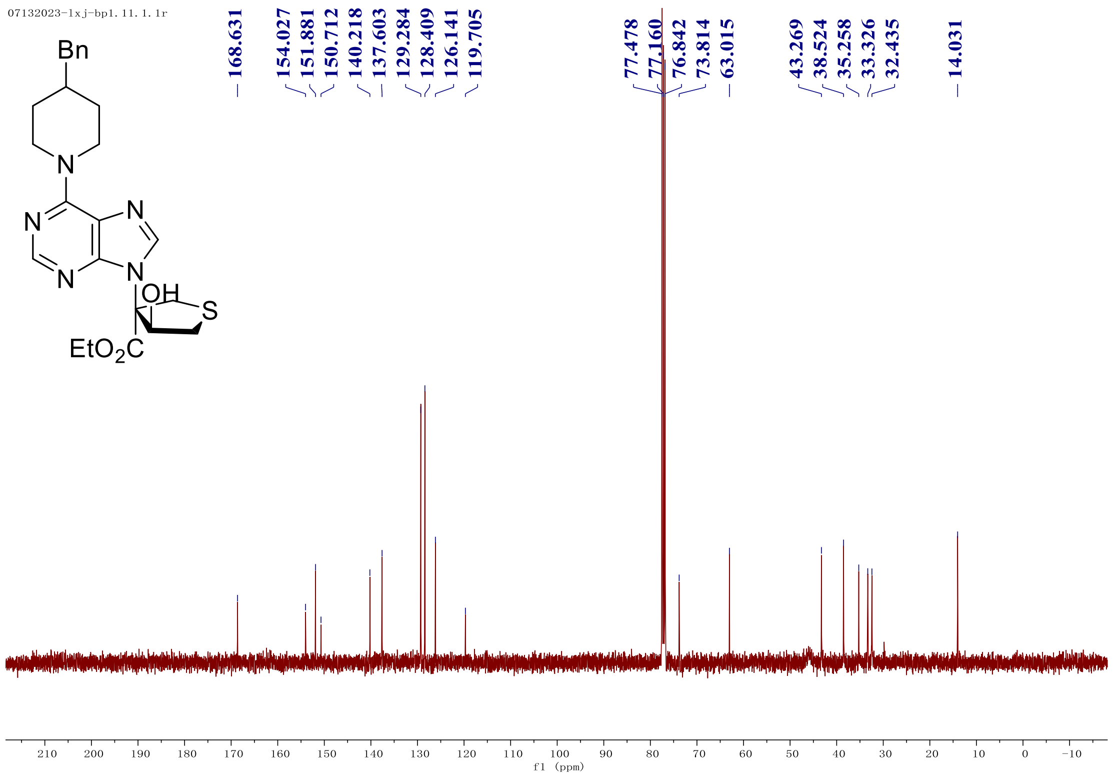

^{13}C NMR spectrum

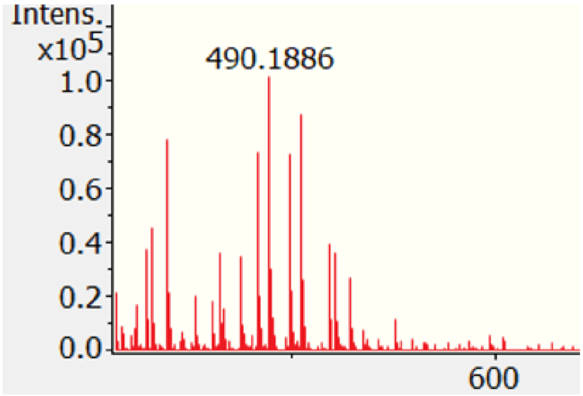

HR-ESIMS spectrum

(±)Ethyl-3-(6-(4-benzylpiperidin-1-yl)-9*H*-purin-9-yl)-4-hydroxytetrahydrothiophene-3-carboxylate (**16b**)

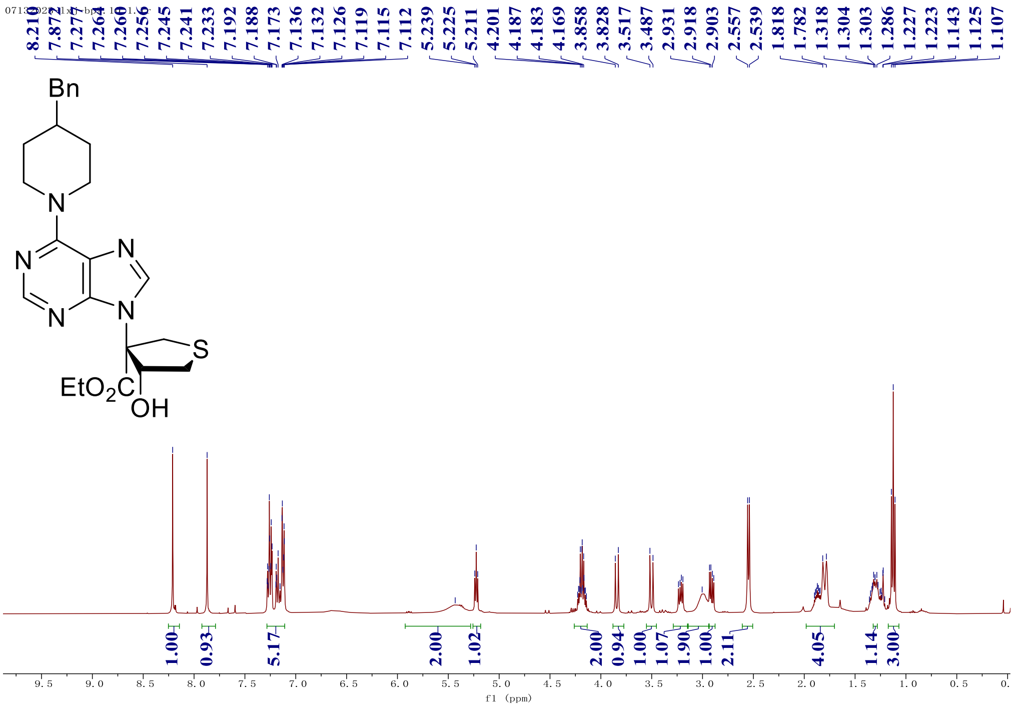

^{1}H NMR spectrum

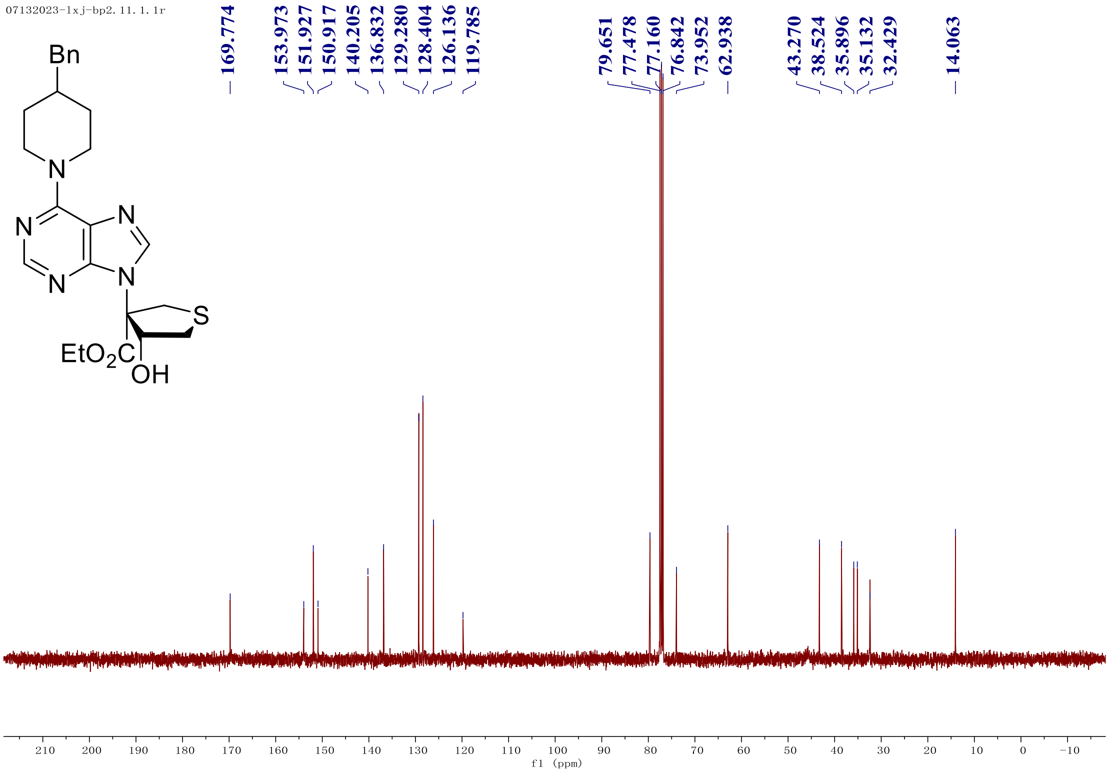

^{13}C NMR spectrum

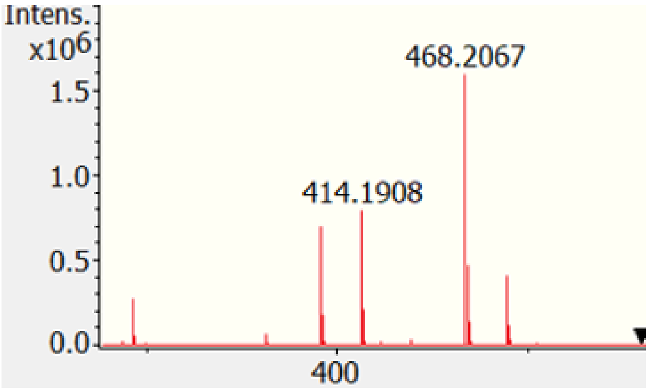

HR-ESIMS spectrum

(±)Ethyl-3-(6-(4-(2-fluorobenzyl) piperidin-1-yl)-9*H*-purin-9-yl)-4-hydroxytetrahydrothiophene-3-carboxylate (**17a**)

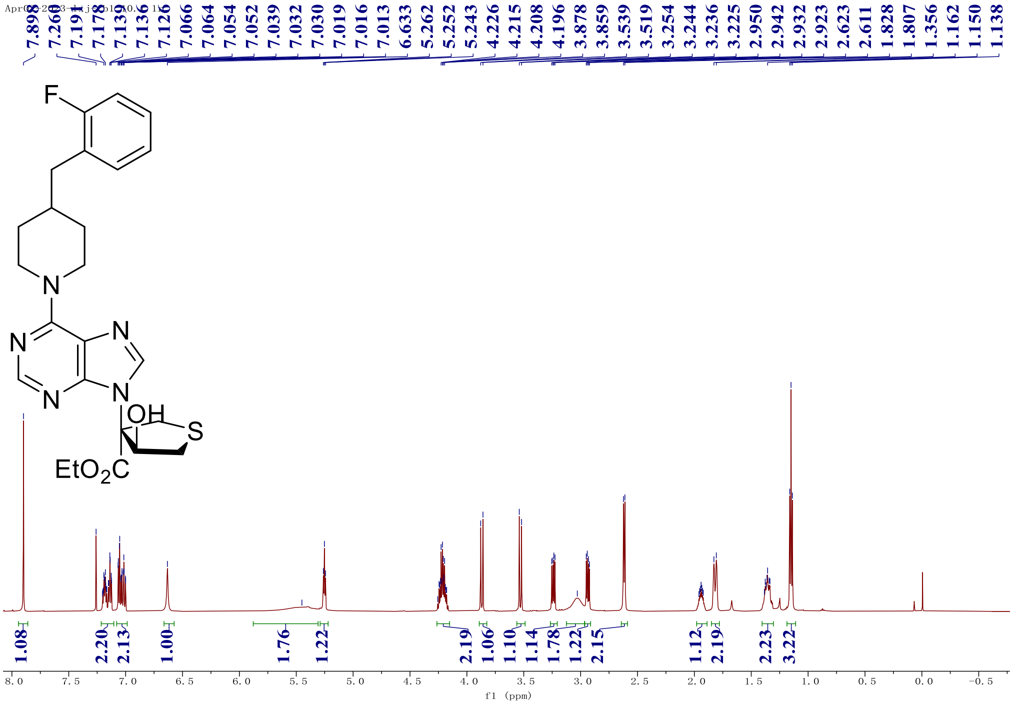

^{1}H NMR spectrum

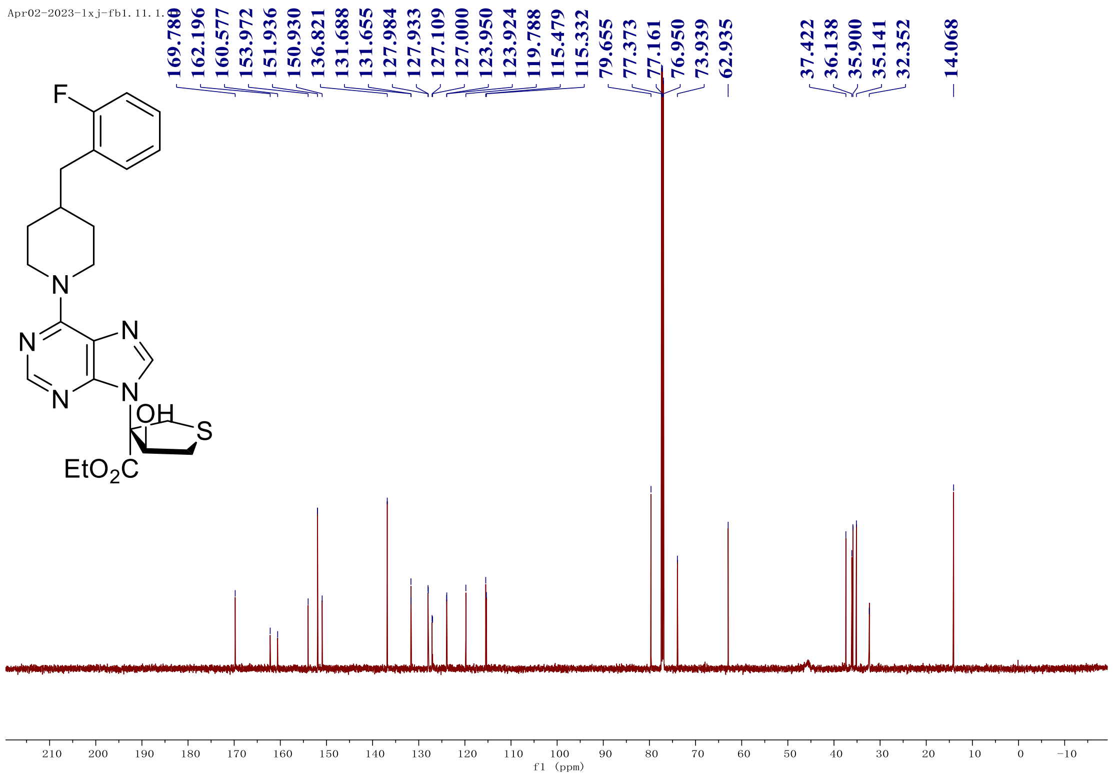

^{13}C NMR spectrum

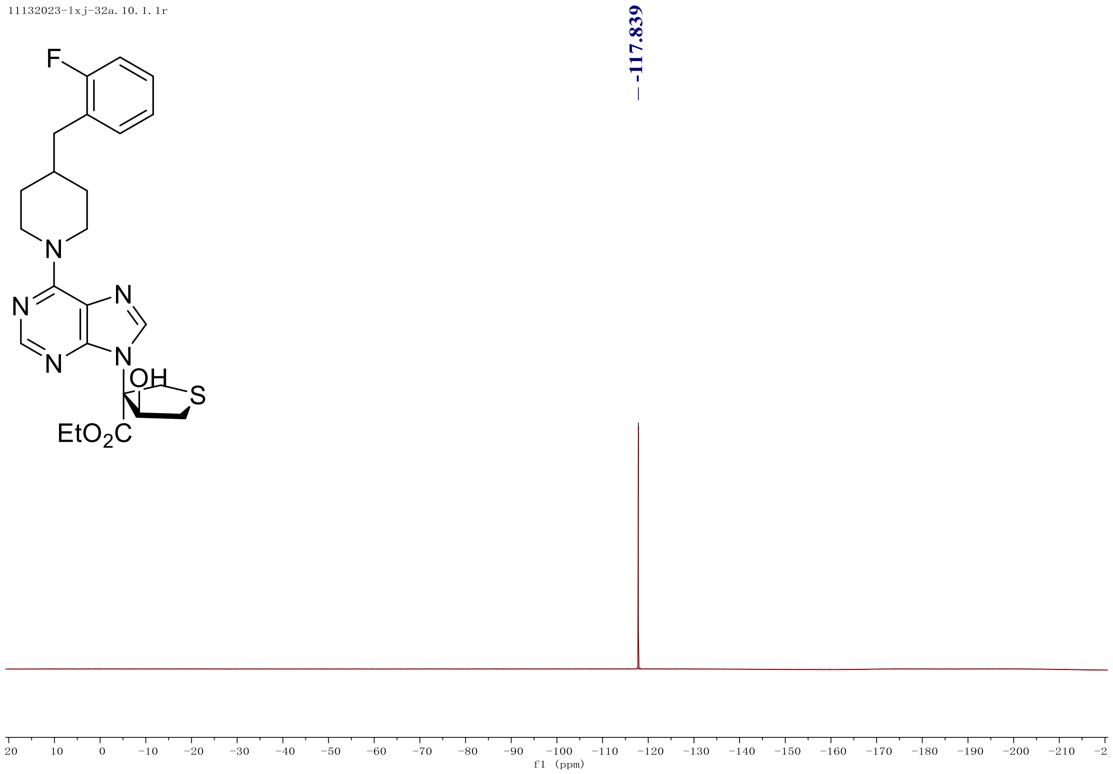

^{19}F NMR spectrum

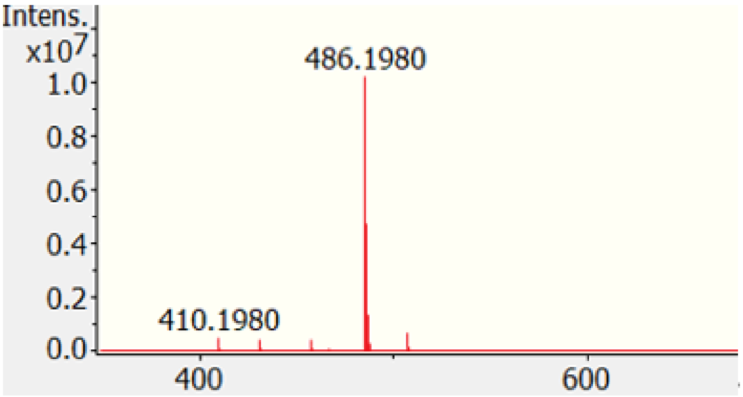

HR-ESIMS spectrum

(±)Ethyl-3-(6-(4-(2-fluorobenzyl) piperidin-1-yl)-9*H*-purin-9-yl)-4-hydroxytetrahydrothiophene-3-carboxylate (**17b**)

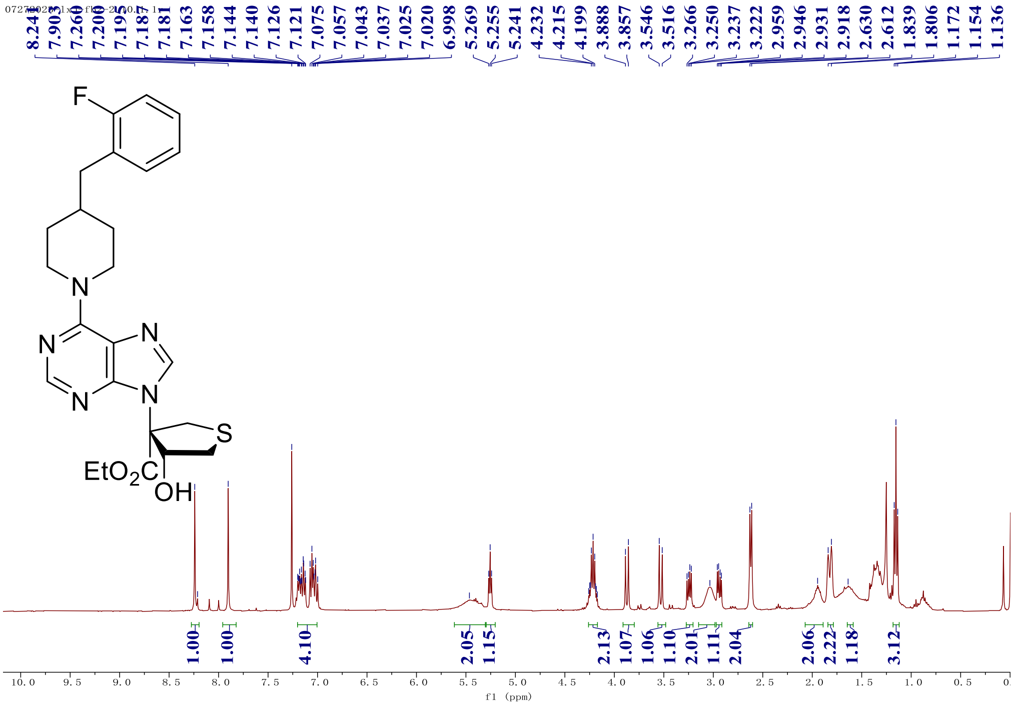

^{1}H NMR spectrum

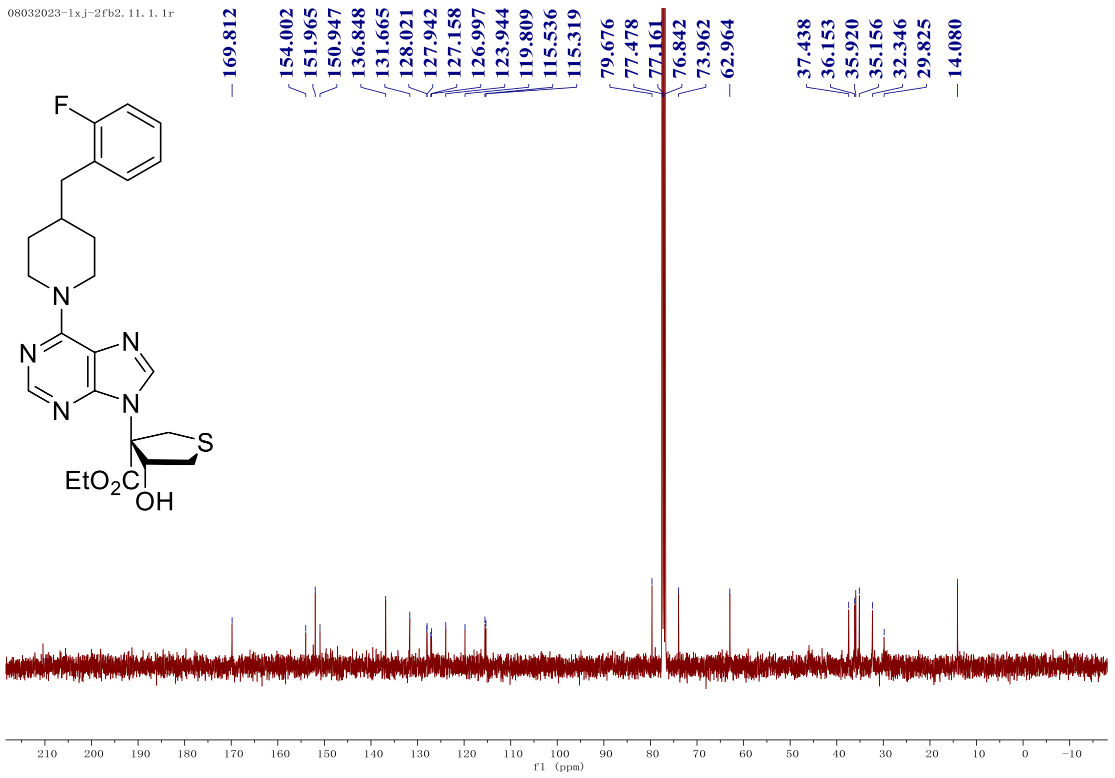

^{13}C NMR spectrum

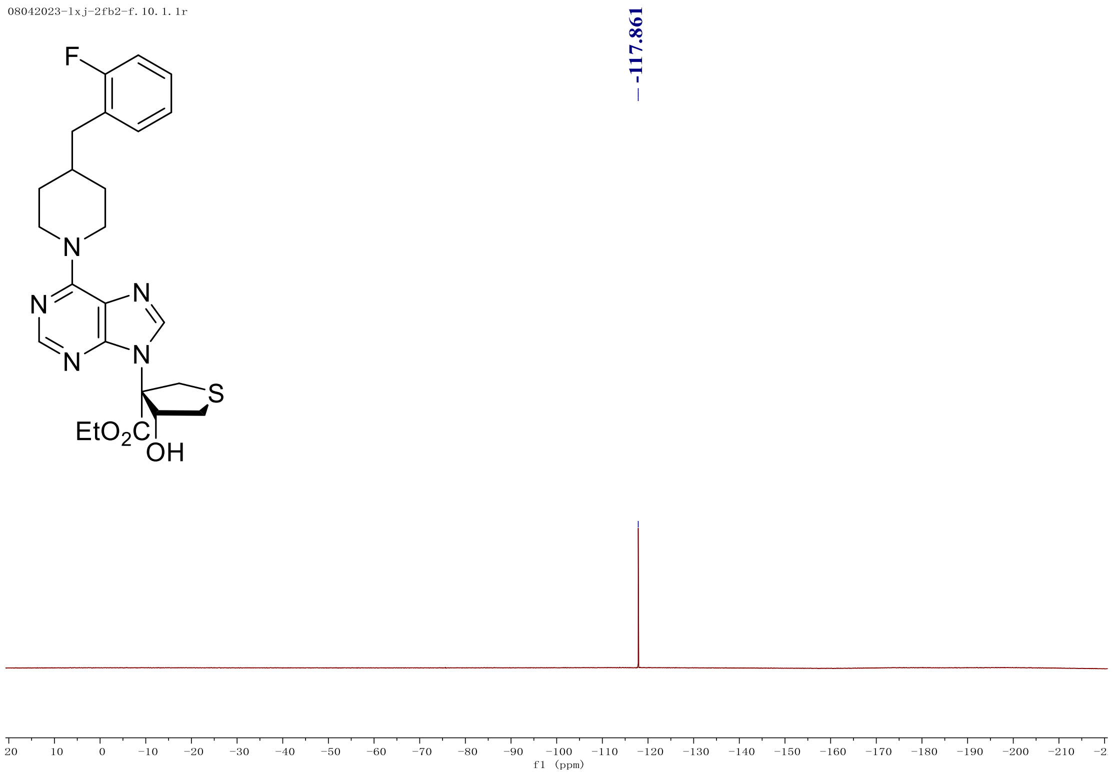

^{19}F NMR spectrum

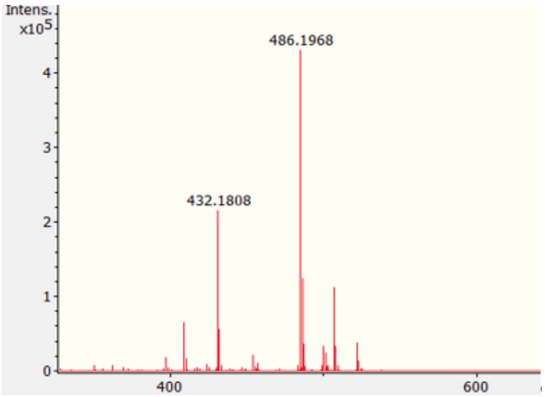

HR-ESIMS spectrum

(±)Ethyl-4-hydroxy-3-(6-(4-methylpiperazin-1-yl)-9*H*-purin-9-yl)tetrahydrothiophene-3-carboxylate (**18a**)

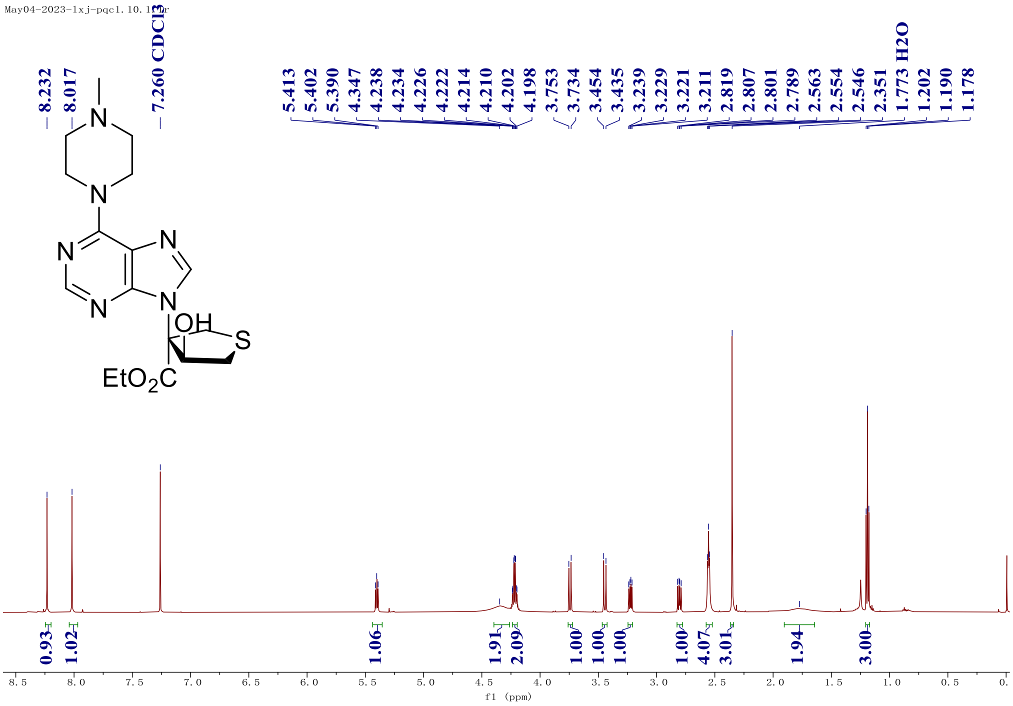

^{1}H NMR spectrum

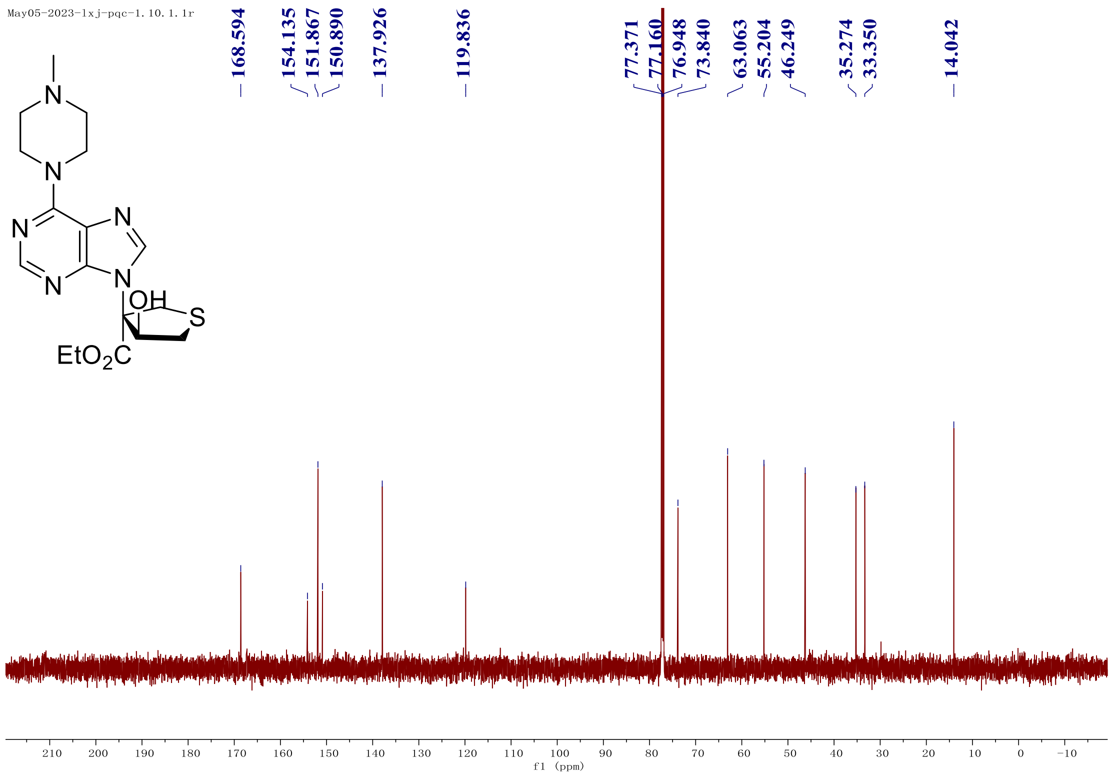

^{13}C NMR spectrum

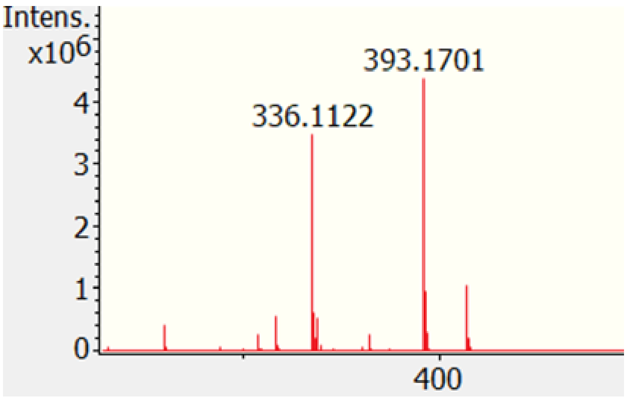

HR-ESIMS spectrum

(±)Ethyl-4-hydroxy-3-(6-(4-methylpiperazin-1-yl)-9*H*-purin-9-yl)tetrahydrothiophene-3-carboxylate (**18b**)

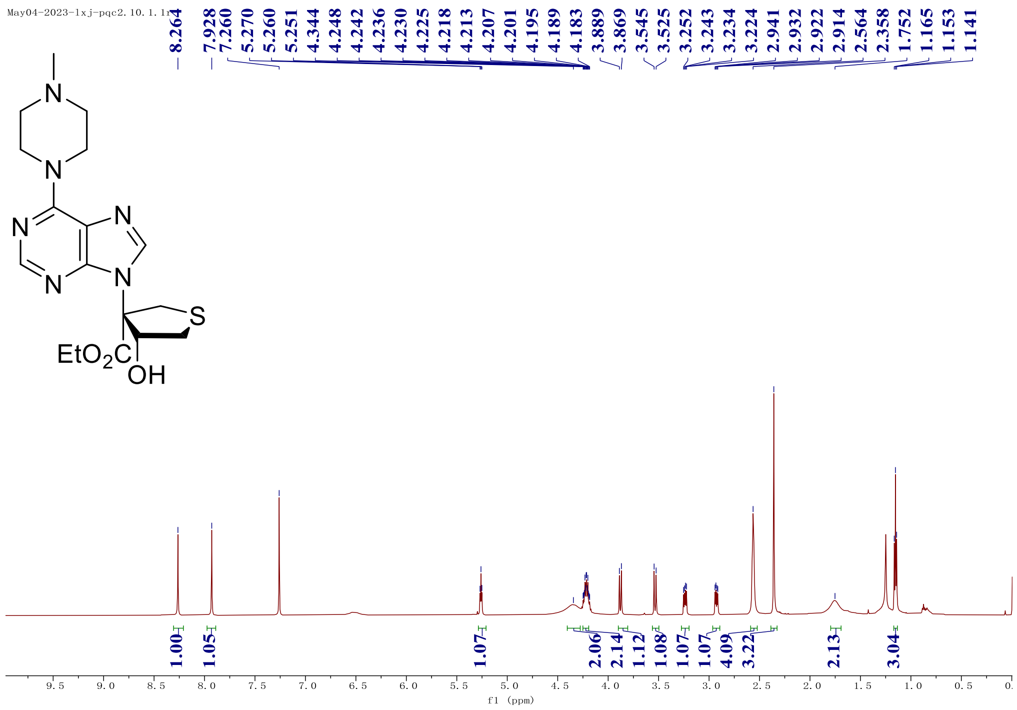

^{1}H NMR spectrum

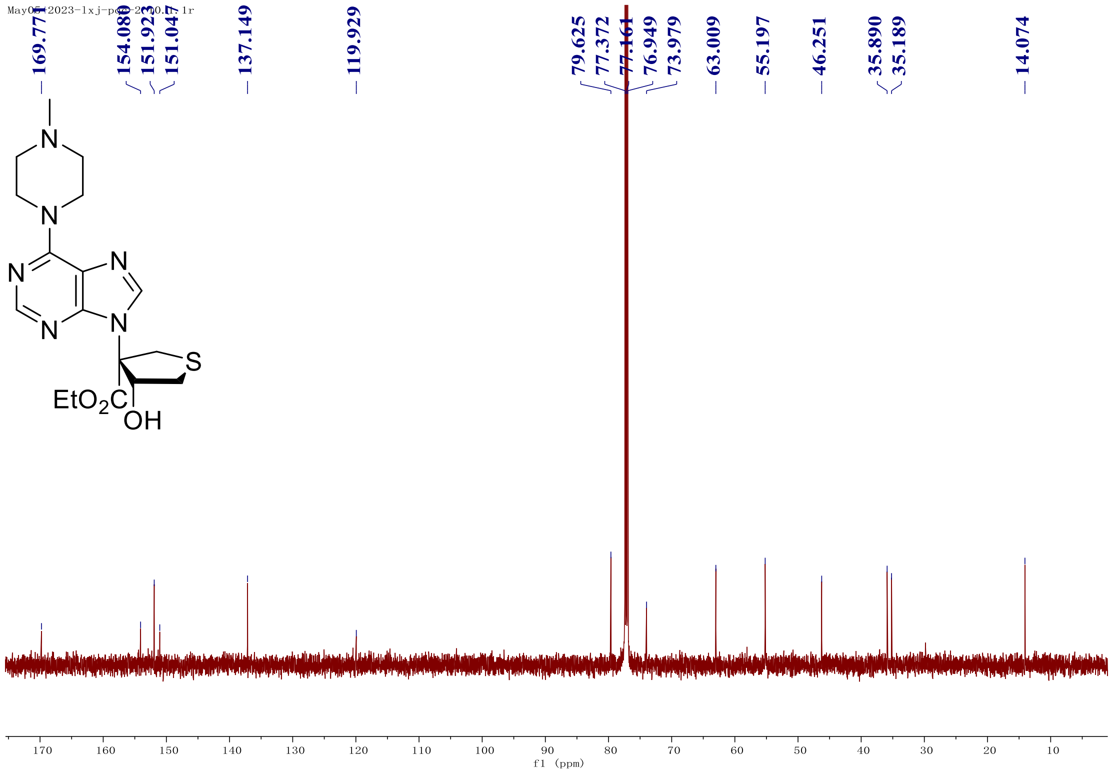

^{13}C NMR spectrum

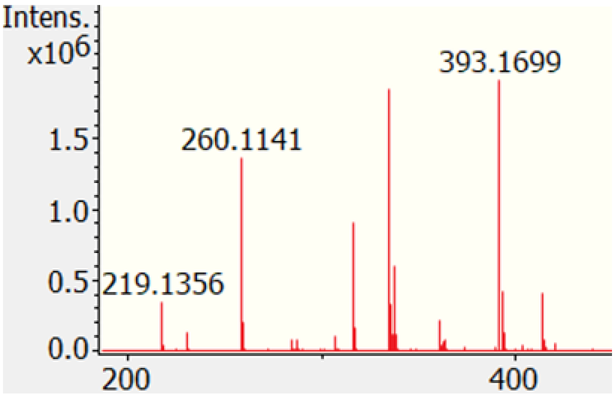

HR-ESIMS spectrum

(±)Ethyl-3-(6-(azepan-1-yl)-9*H*-purin-9-yl)-4-hydroxytetrahydrothiophene-3-carboxylate (**19a**)

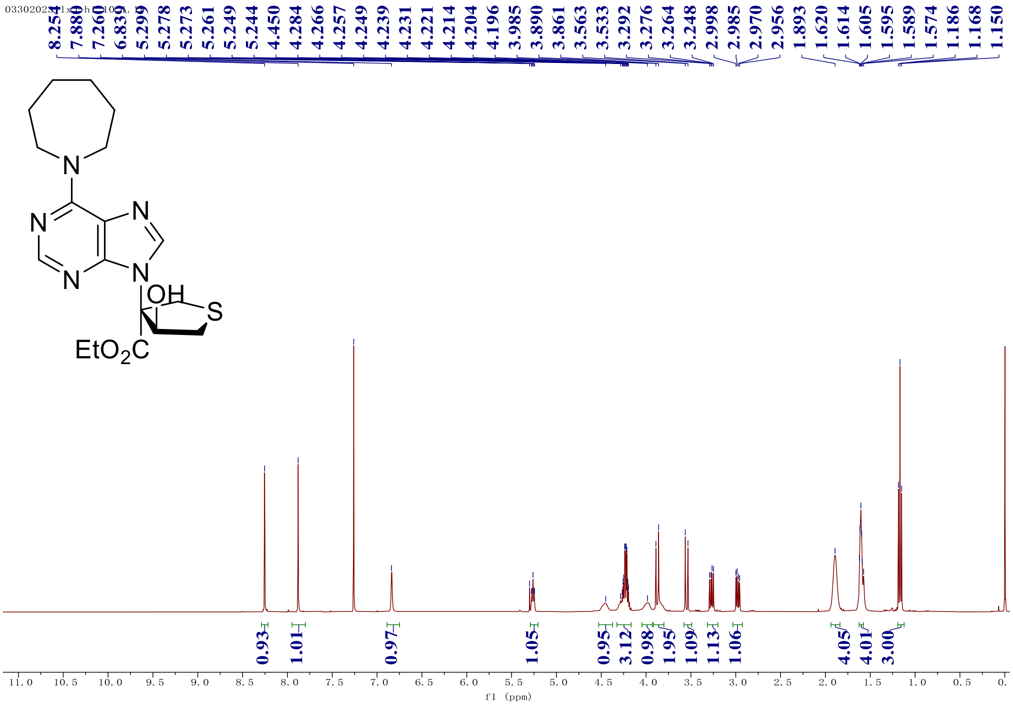

^{1}H NMR spectrum

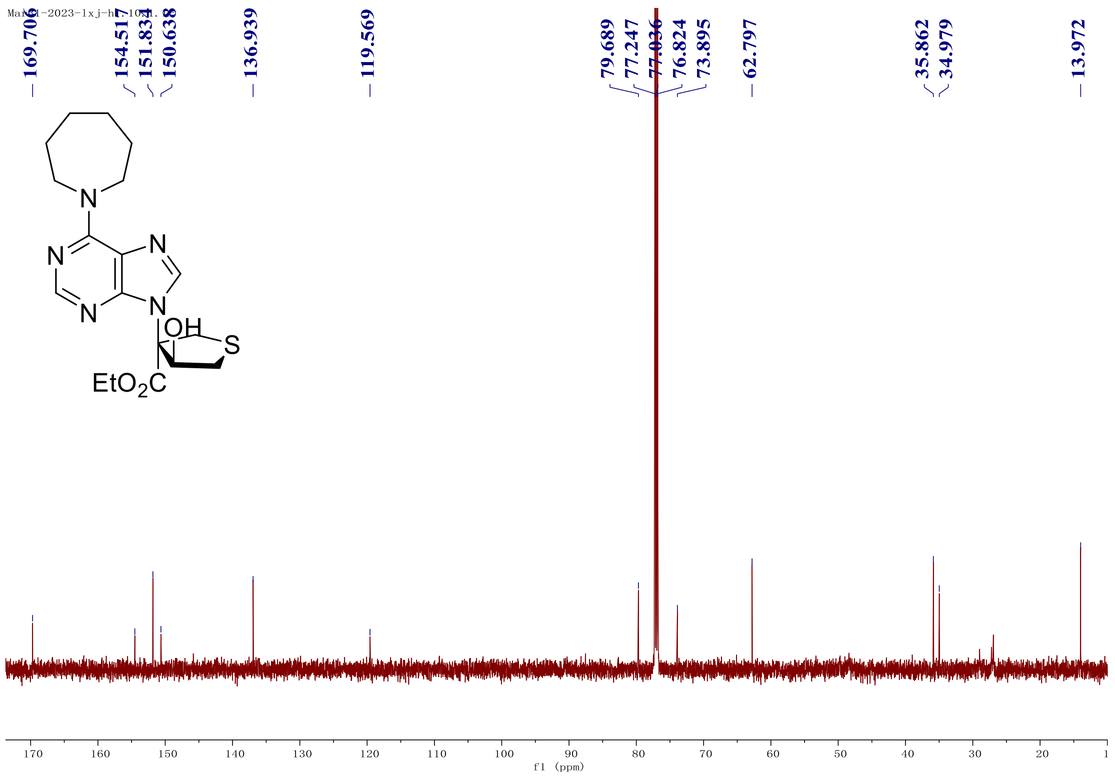

^{13}C NMR spectrum

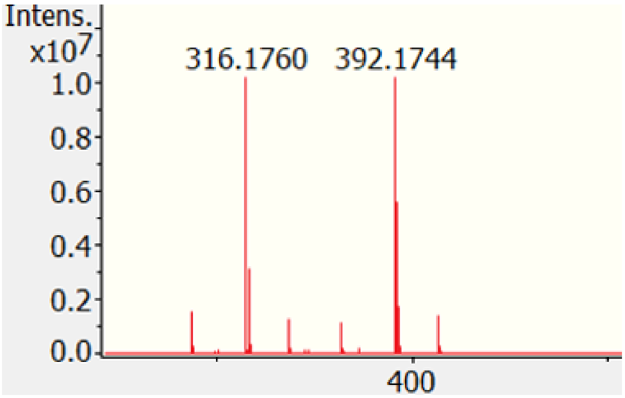

HR-ESIMS spectrum

(±)Ethyl-3-(6-(azepan-1-yl)-9*H*-purin-9-yl)-4-hydroxytetrahydrothiophene-3-carboxylate (**19b**)

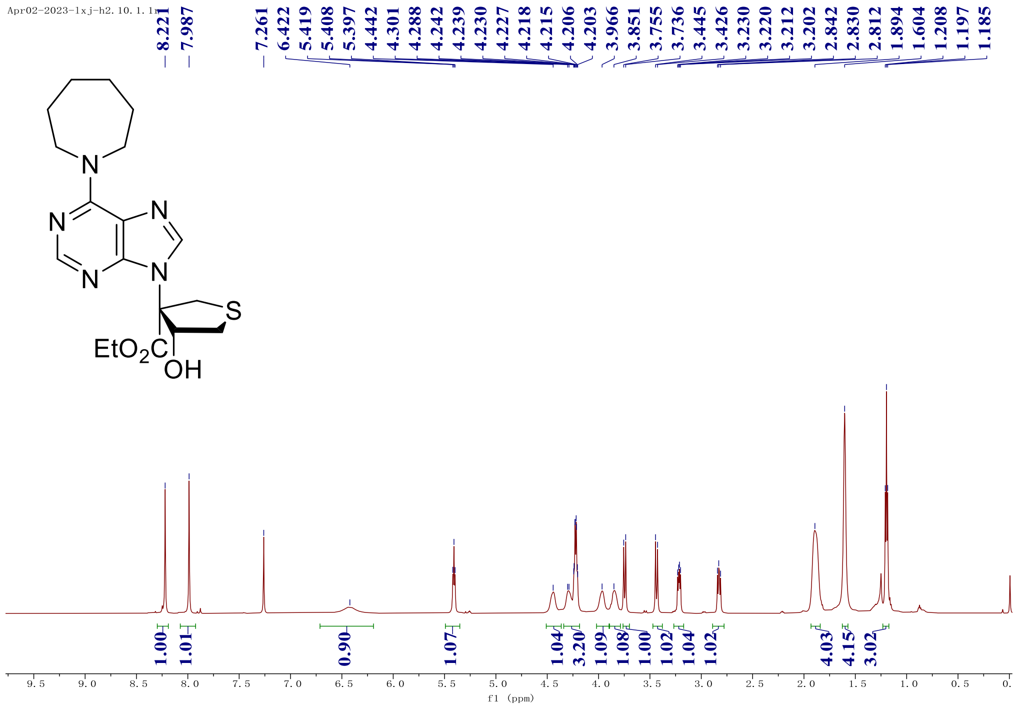

^{1}H NMR spectrum

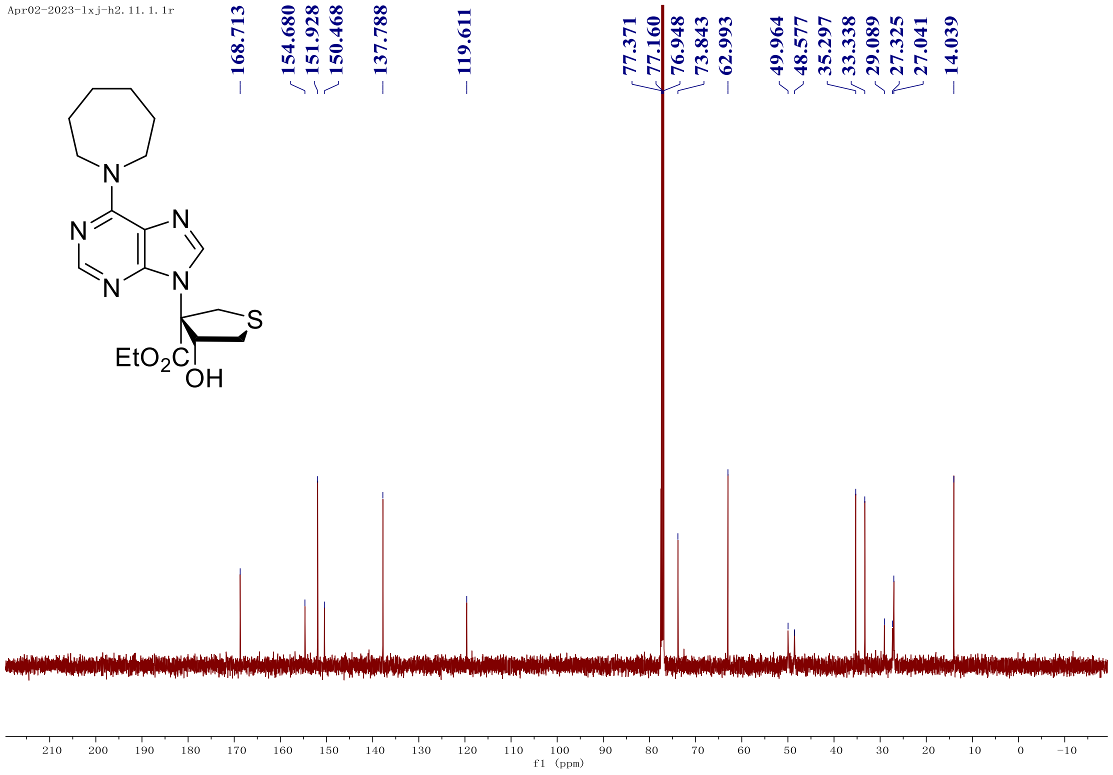

^{13}C NMR spectrum

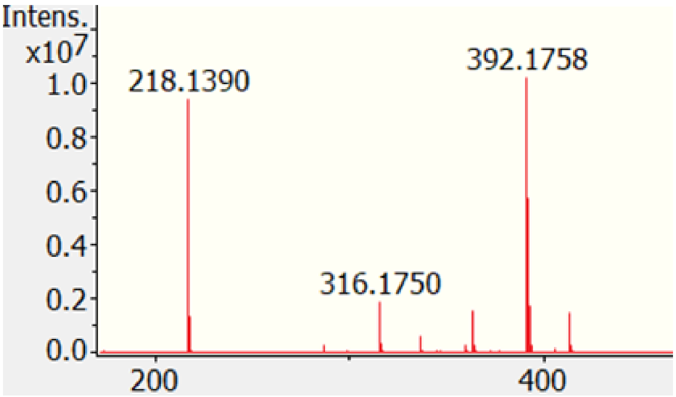

HR-ESIMS spectrum

(±)Ethyl-4-hydroxy-3-(6-(pyrrolidin-1-yl)-9*H*-purin-9-yl)tetrahydrothiophene-3-carboxylate (**20a**)

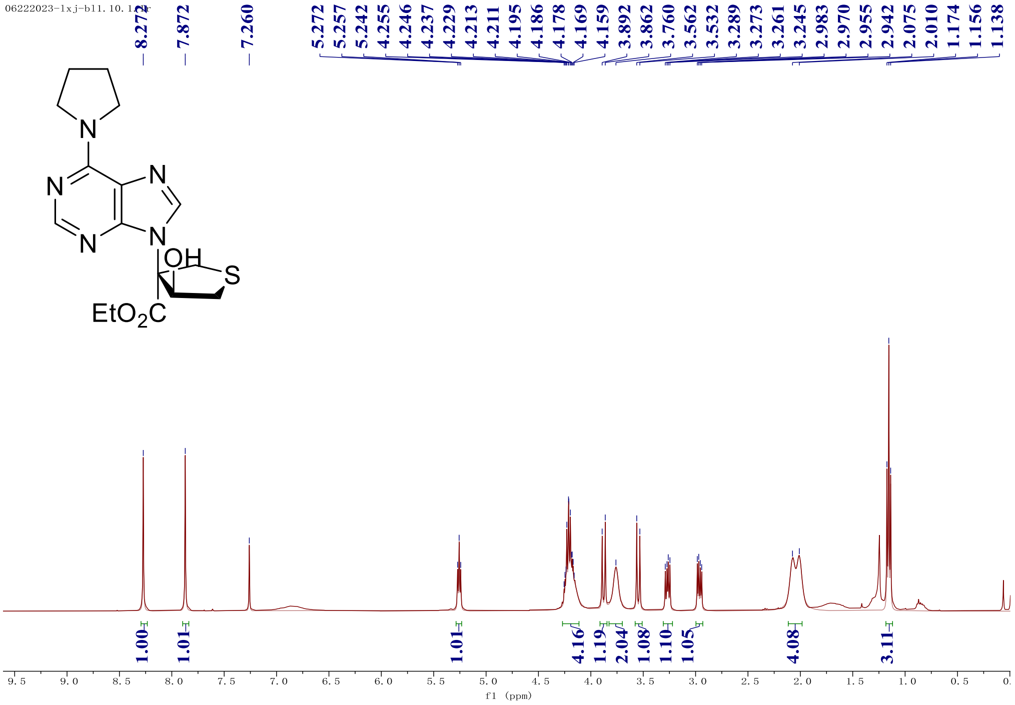

^{1}H NMR spectrum

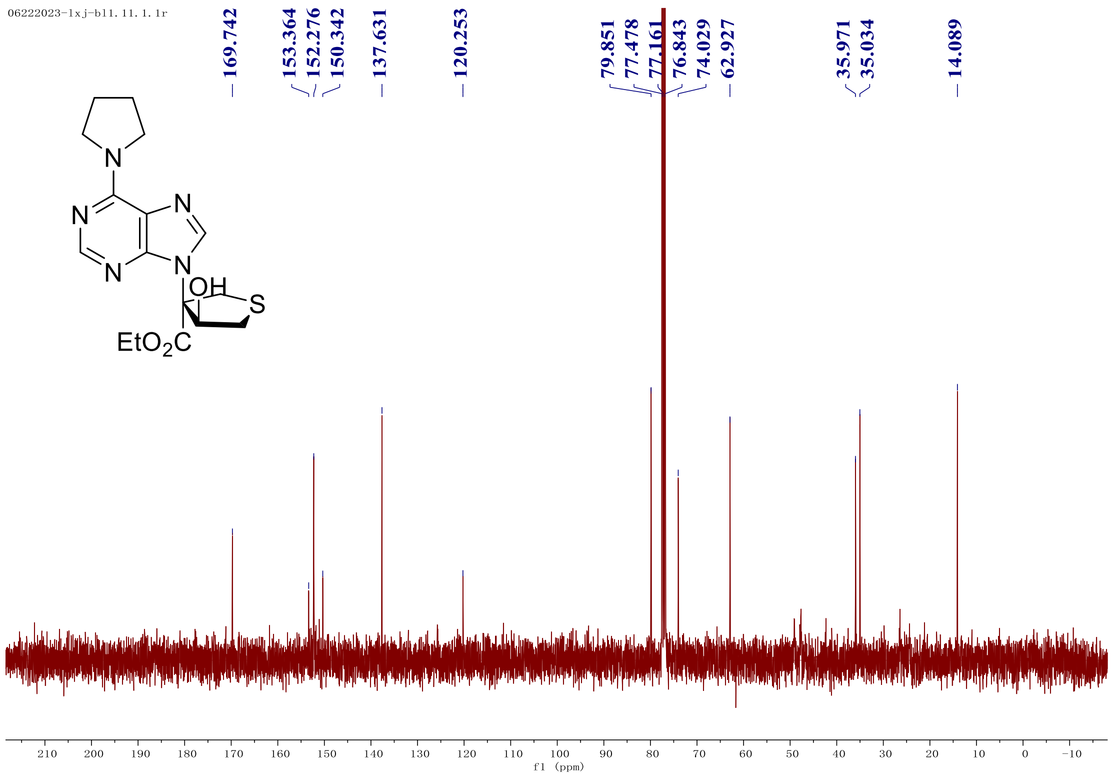

^{13}C NMR spectrum

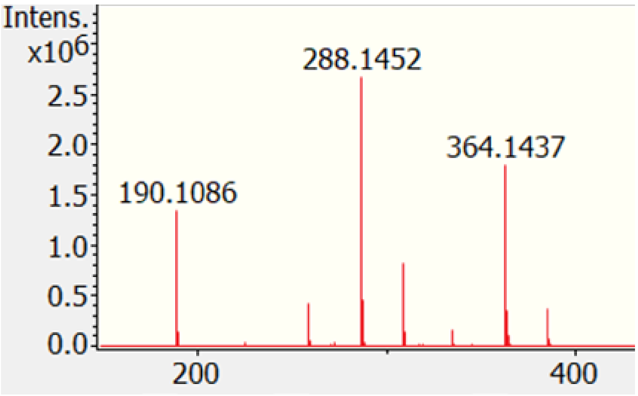

HR-ESIMS spectrum

(±)Ethyl-4-hydroxy-3-(6-(pyrrolidin-1-yl)-9*H*-purin-9-yl)tetrahydrothiophene-3-carboxylate (**20b**)

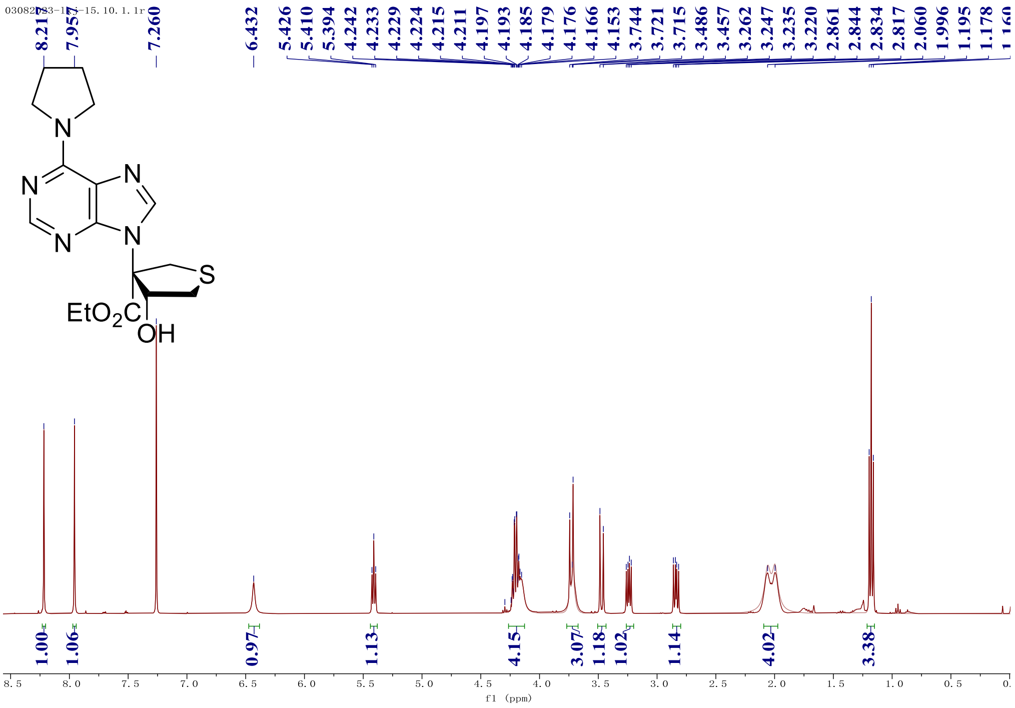

^{1}H NMR spectrum

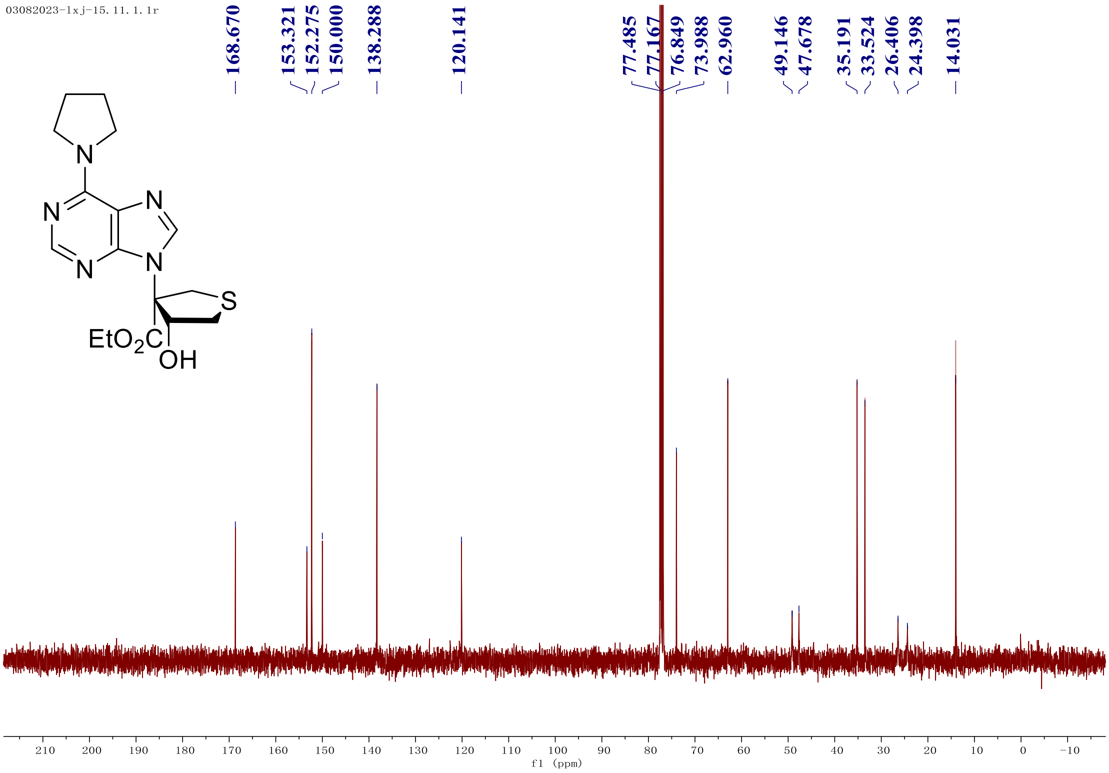

^{13}C NMR spectrum

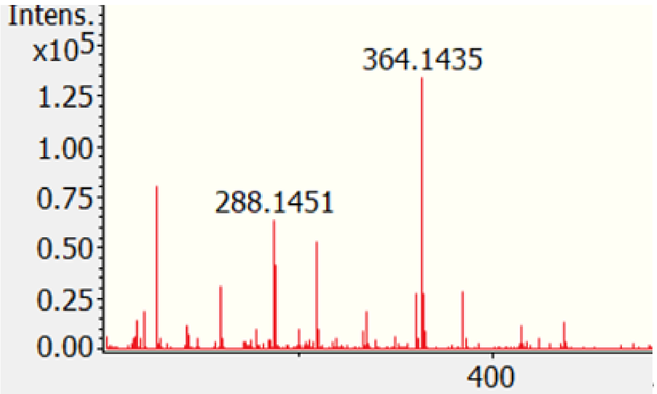

HR-ESIMS spectrum

(±)Ethyl-4-hydroxy-3-(6-(methylthio)-9*H*-purin-9-yl)tetrahydrothiophene-3-carboxylate (**21a**)

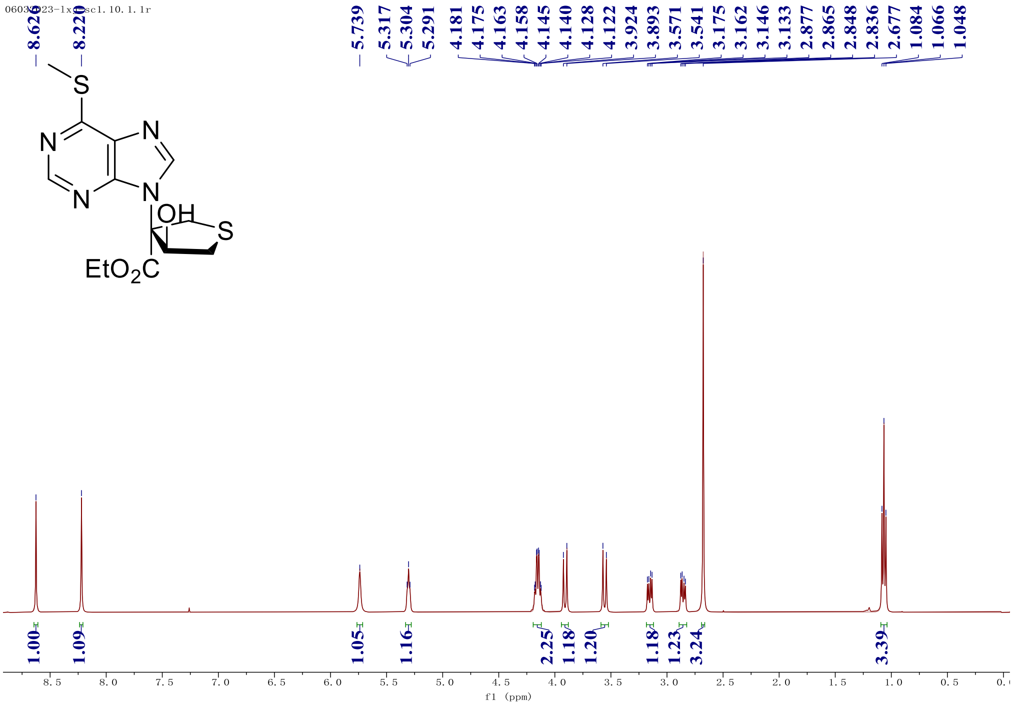

^{1}H NMR spectrum

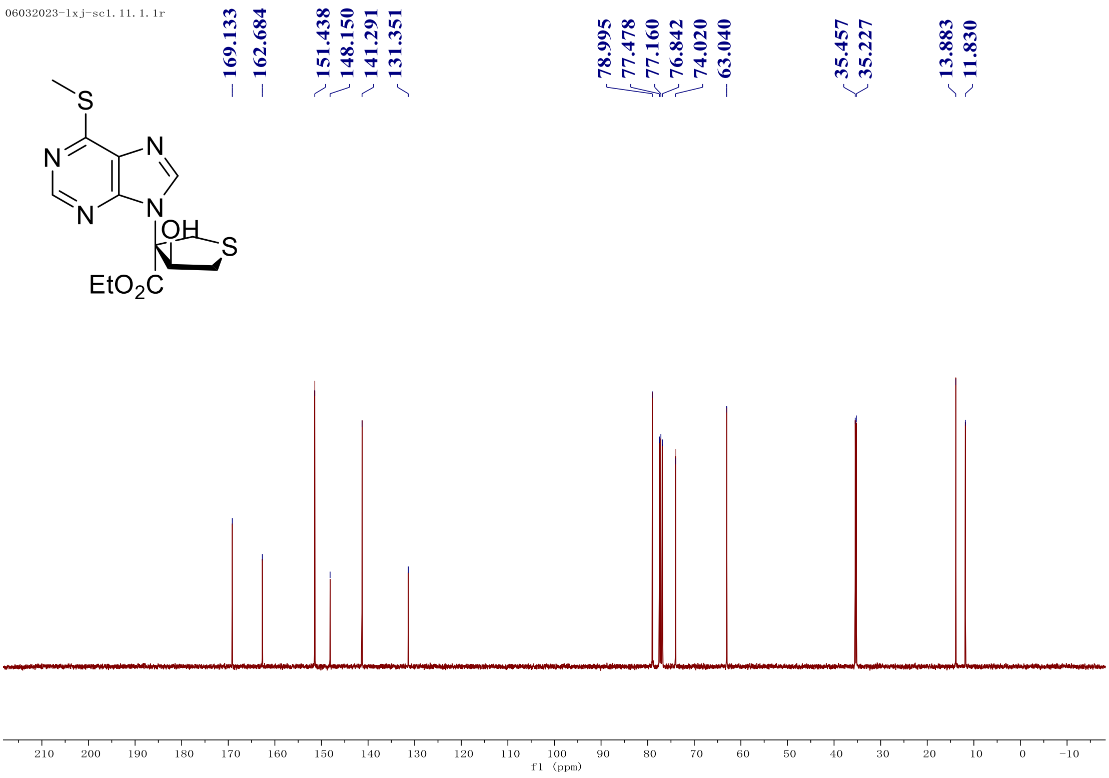

^{13}C NMR spectrum

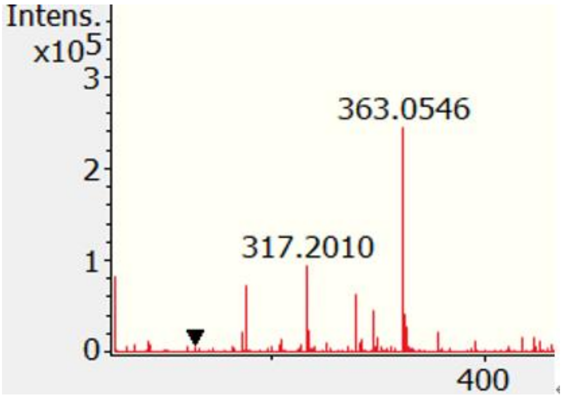

HR-ESIMS spectrum

(±)Ethyl-4-hydroxy-3-(6-(methylthio)-9*H*-purin-9-yl)tetrahydrothiophene-3-carboxylate (**21b**)

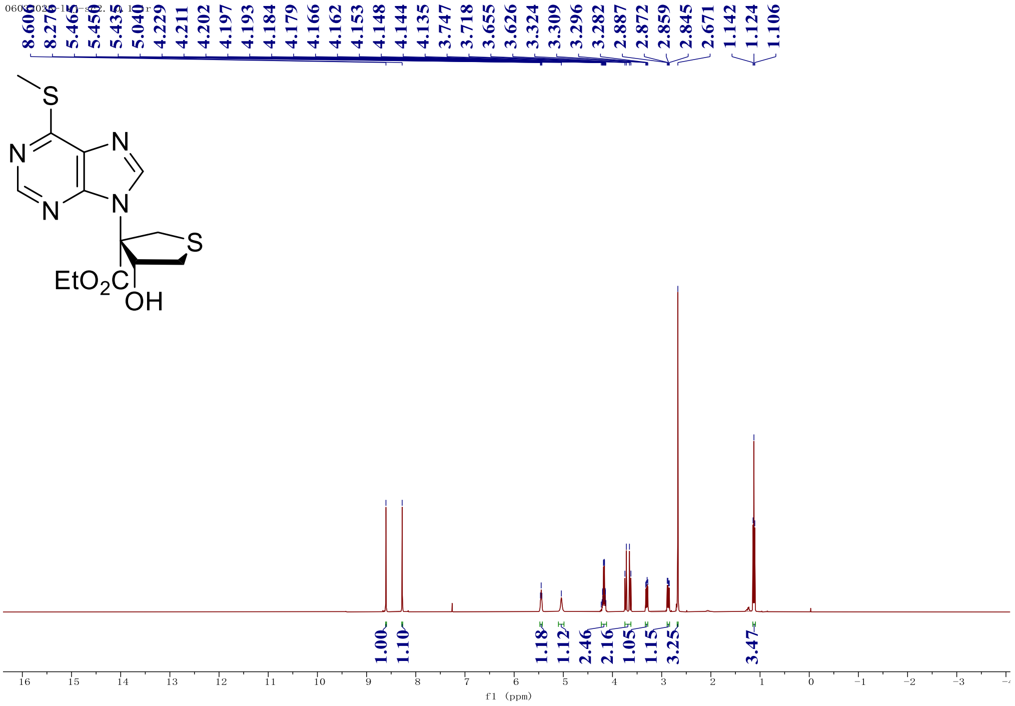

^{1}H NMR spectrum

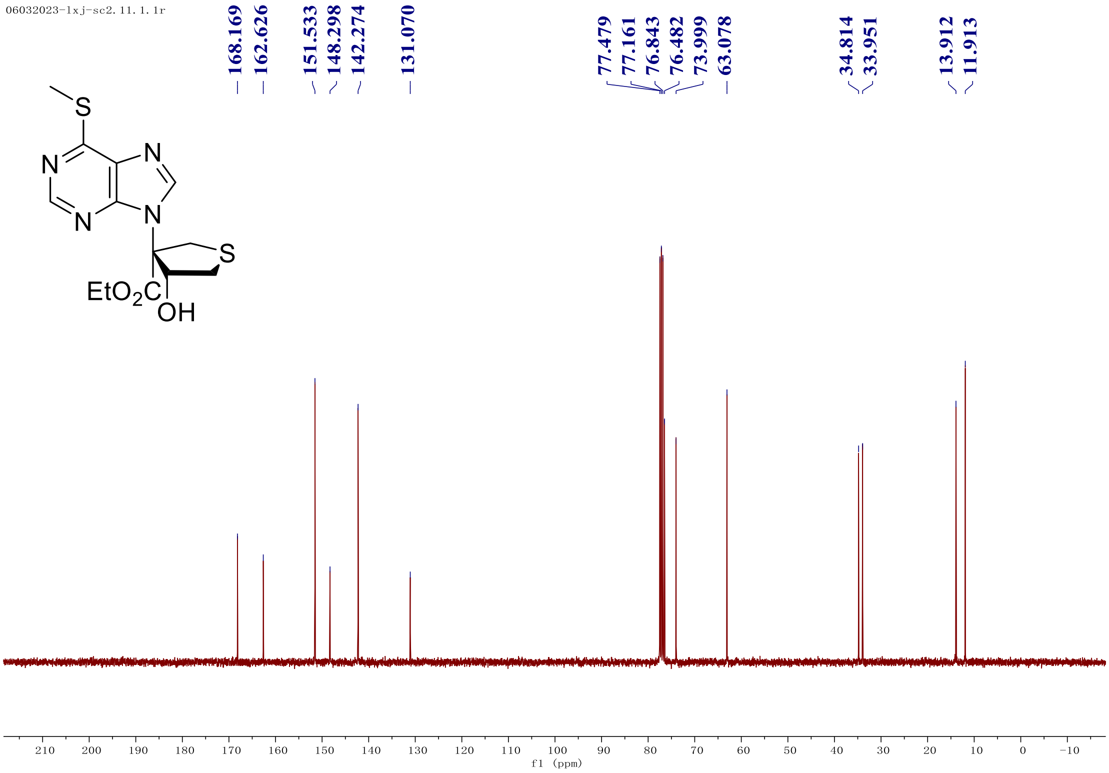

^{13}C NMR spectrum

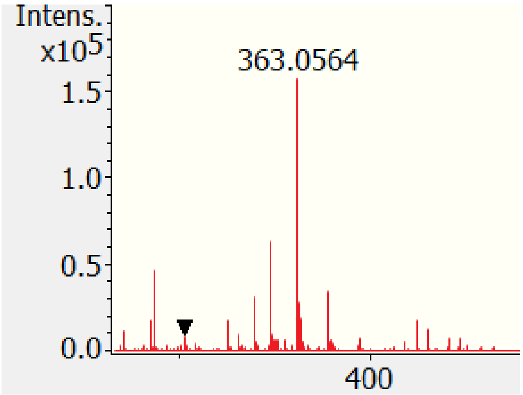

HR-ESIMS spectrum

(±)Ethyl-3-(6-(ethylthio)-9*H*-purin-9-yl)-4-hydroxytetrahydrothiophene-3-carboxylate (**22a**)

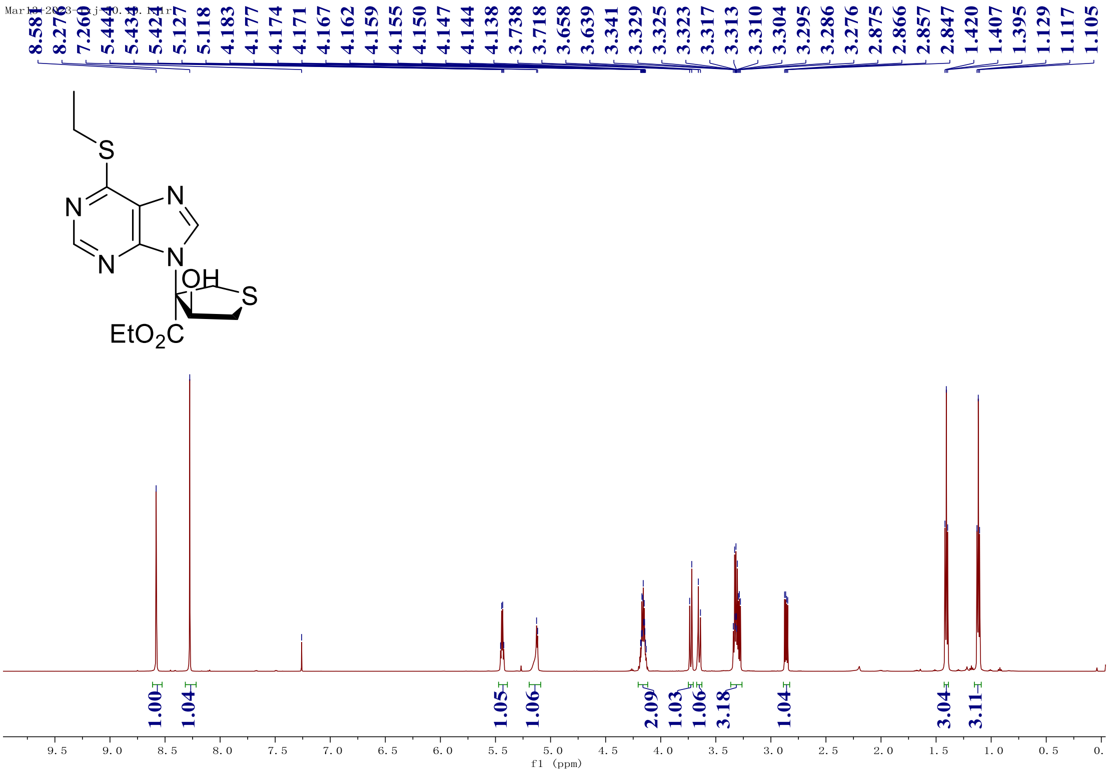

^{1}H NMR spectrum

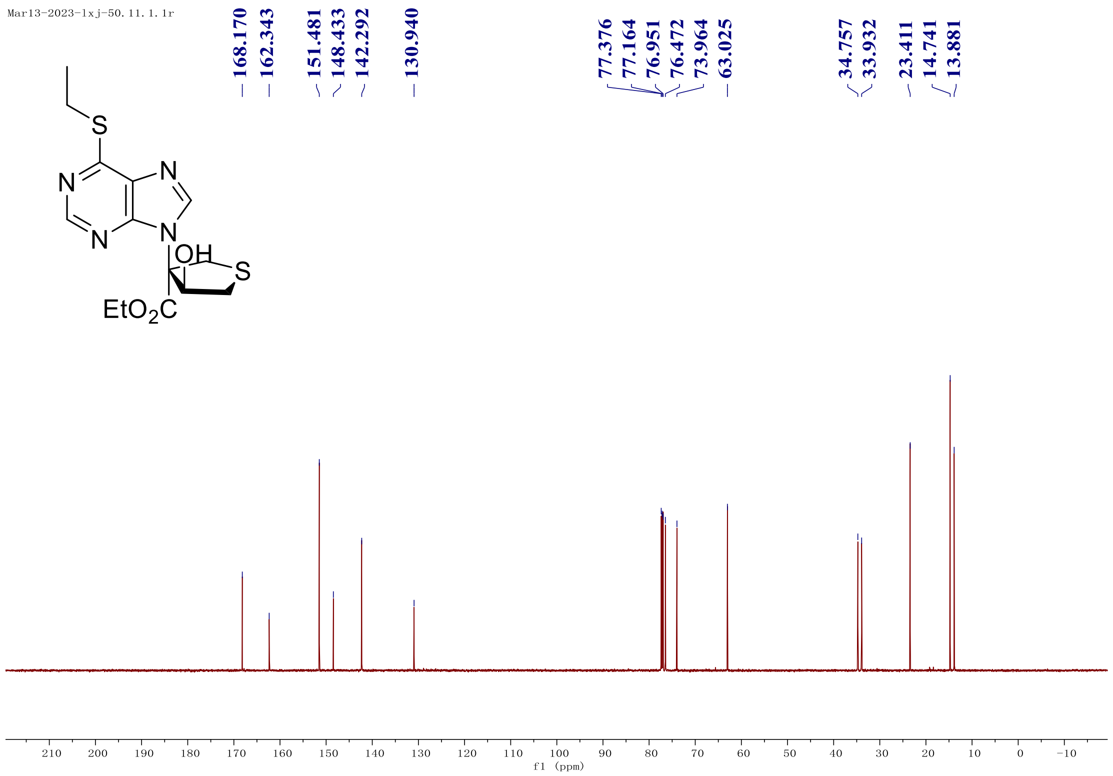

^{13}C NMR spectrum

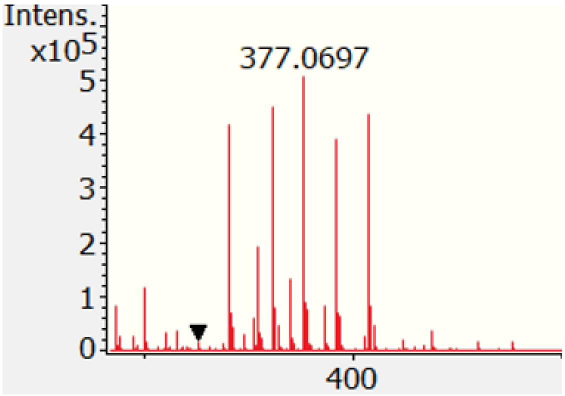

HR-ESIMS spectrum

(±)Ethyl-3-(6-(ethylthio)-9*H*-purin-9-yl)-4-hydroxytetrahydrothiophene-3-carboxylate (**22b**)

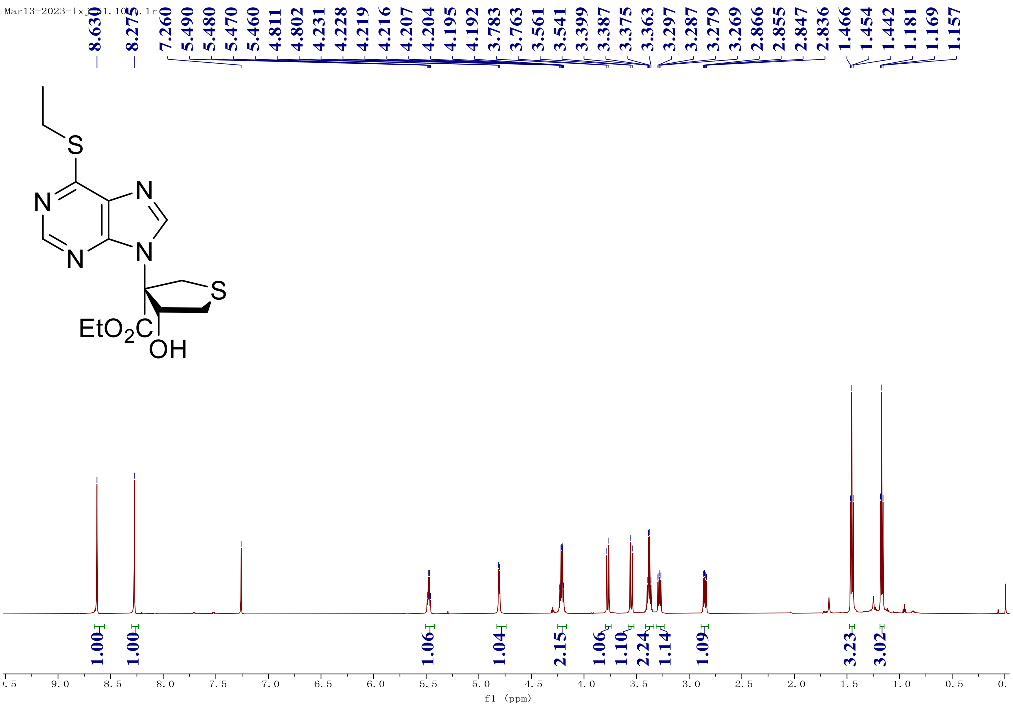

^{1}H NMR spectrum

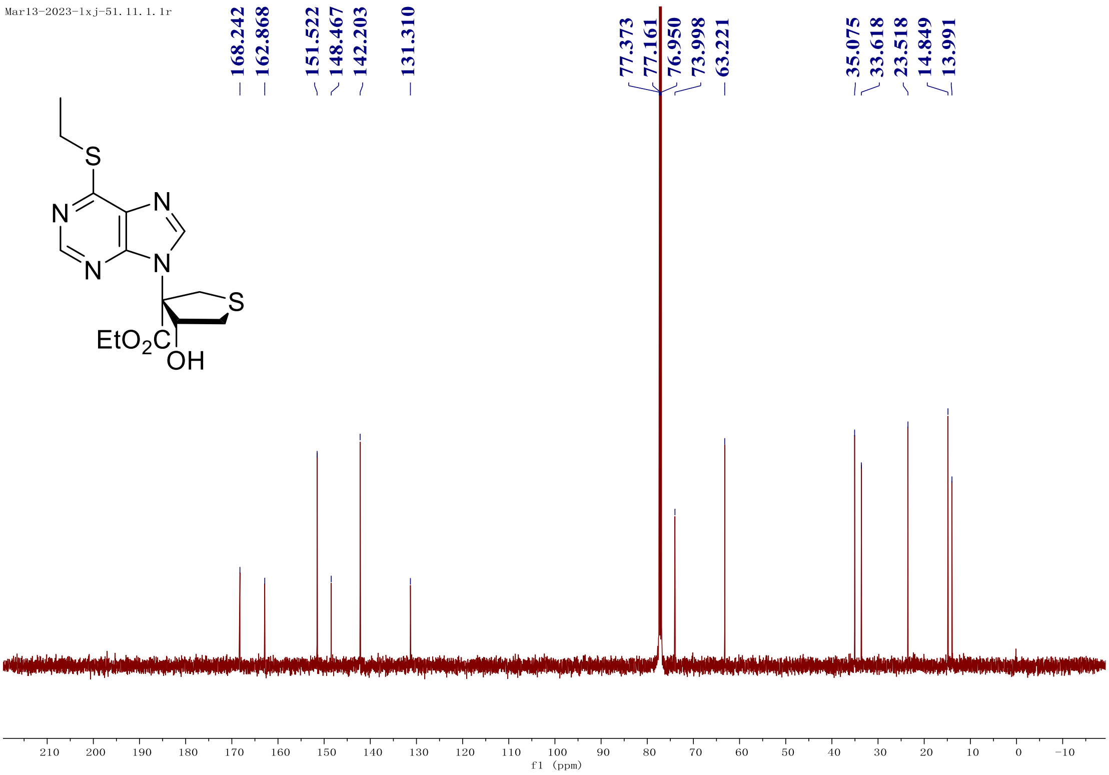

^{13}C NMR spectrum

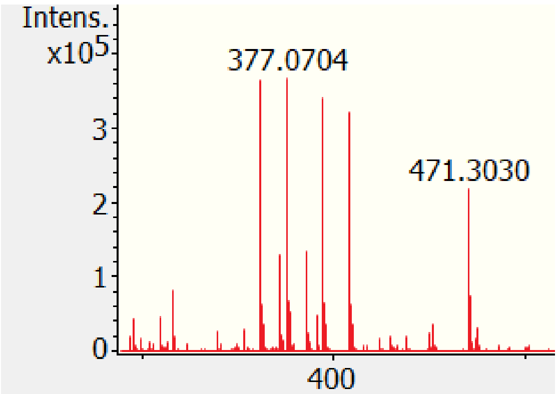

HR-ESIMS spectrum

(±)Ethyl-4-hydroxy-3-(6-(isopropylthio)-9*H*-purin-9-yl) tetrahydrothiophene-3-carboxylate (**23a**)

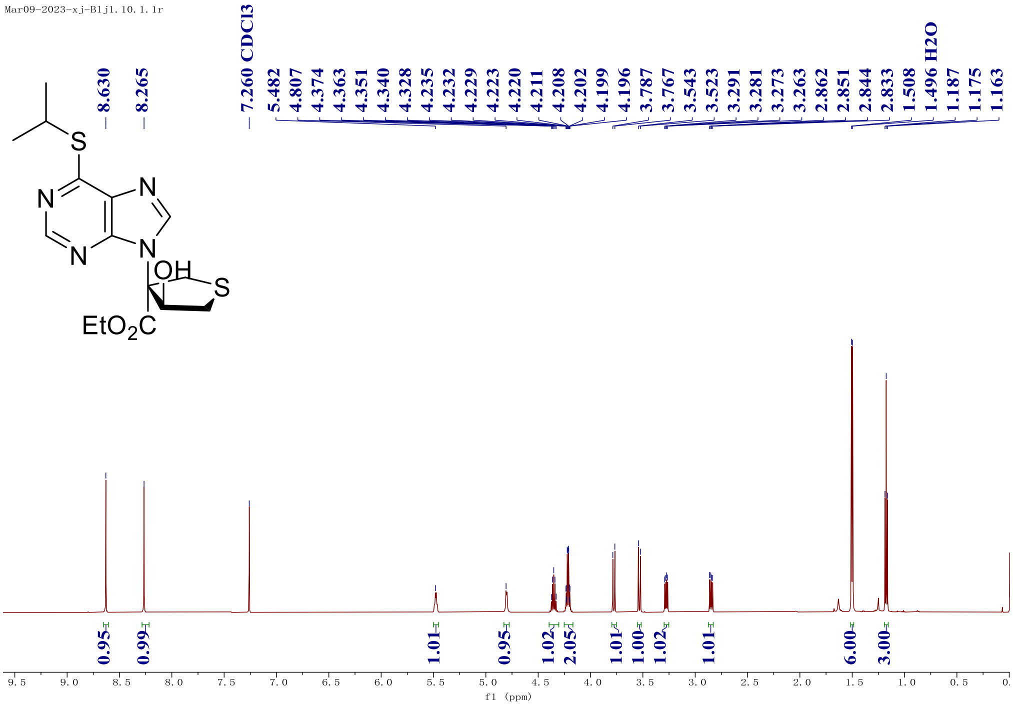

^{1}H NMR spectrum

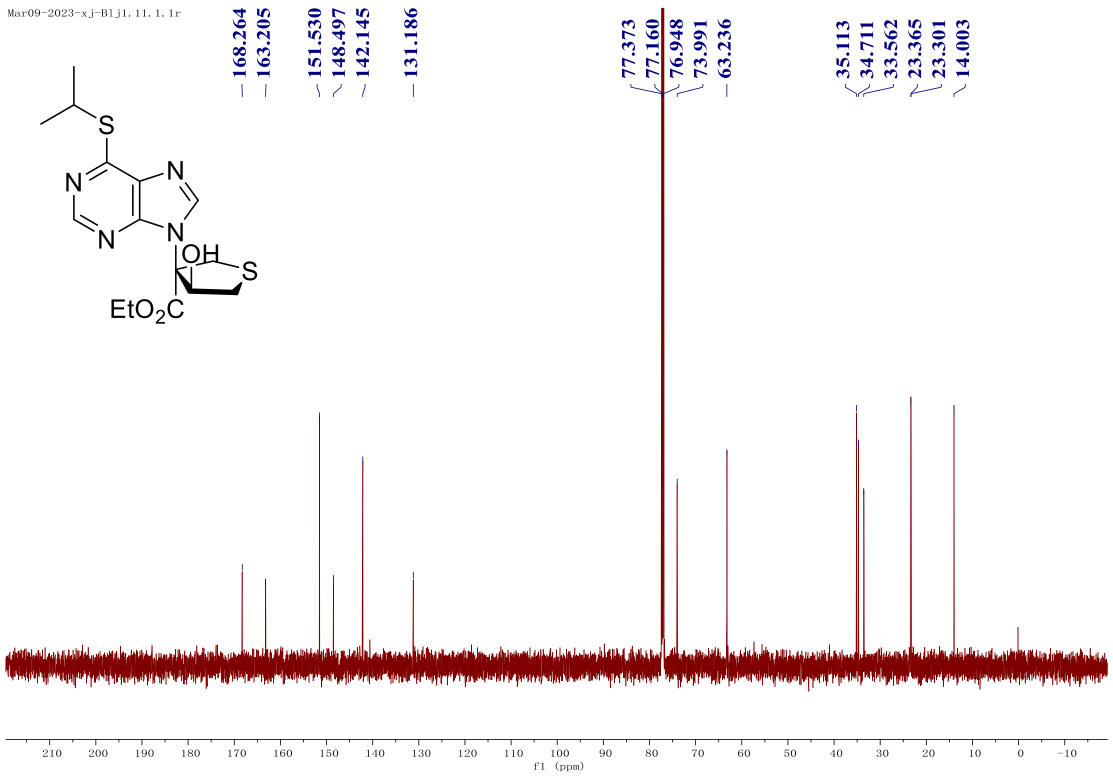

^{13}C NMR spectrum

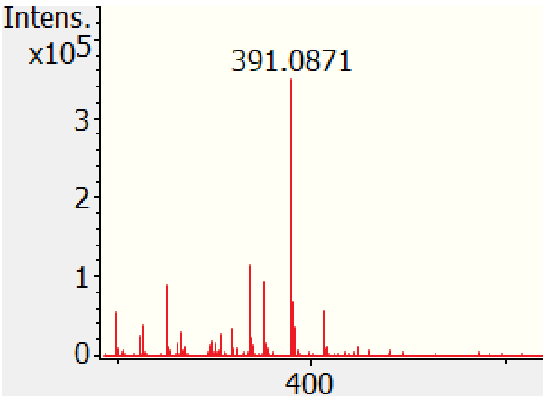

HR-ESIMS spectrum

(±)Ethyl-4-hydroxy-3-(6-(isopropylthio)-9*H*-purin-9-yl) tetrahydrothiophene-3-carboxylate (**23b**)

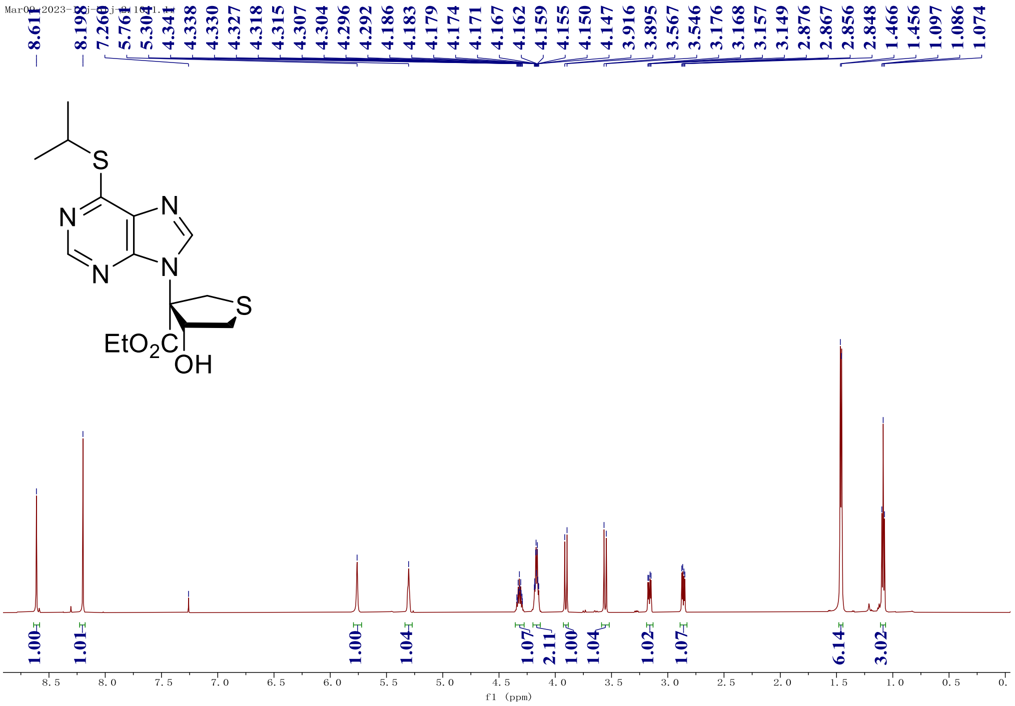

^{1}H NMR spectrum

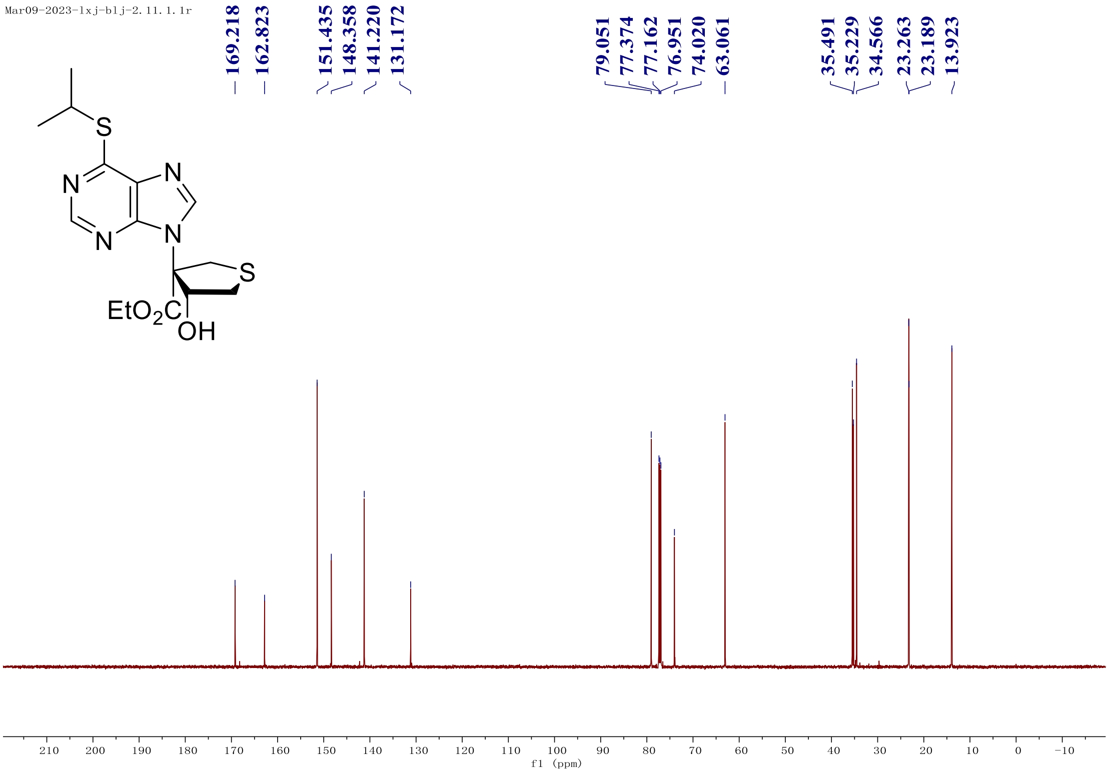

^{13}C NMR spectrum

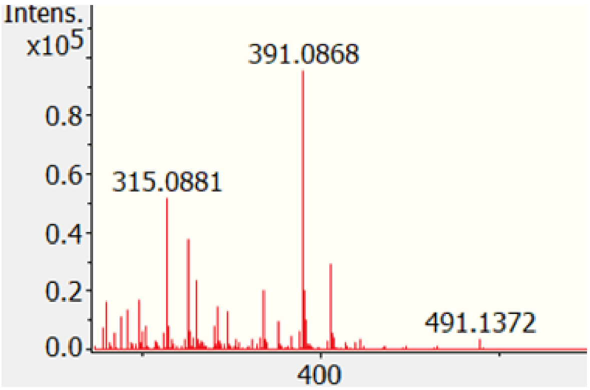

HR-ESIMS spectrum

(±)Ethyl-4-hydroxy-3-(6-(prop-2-yn-1-ylthio)-9*H*-purin-9-yl)tetrahydrothiophene-3-carboxylate (**24a**)

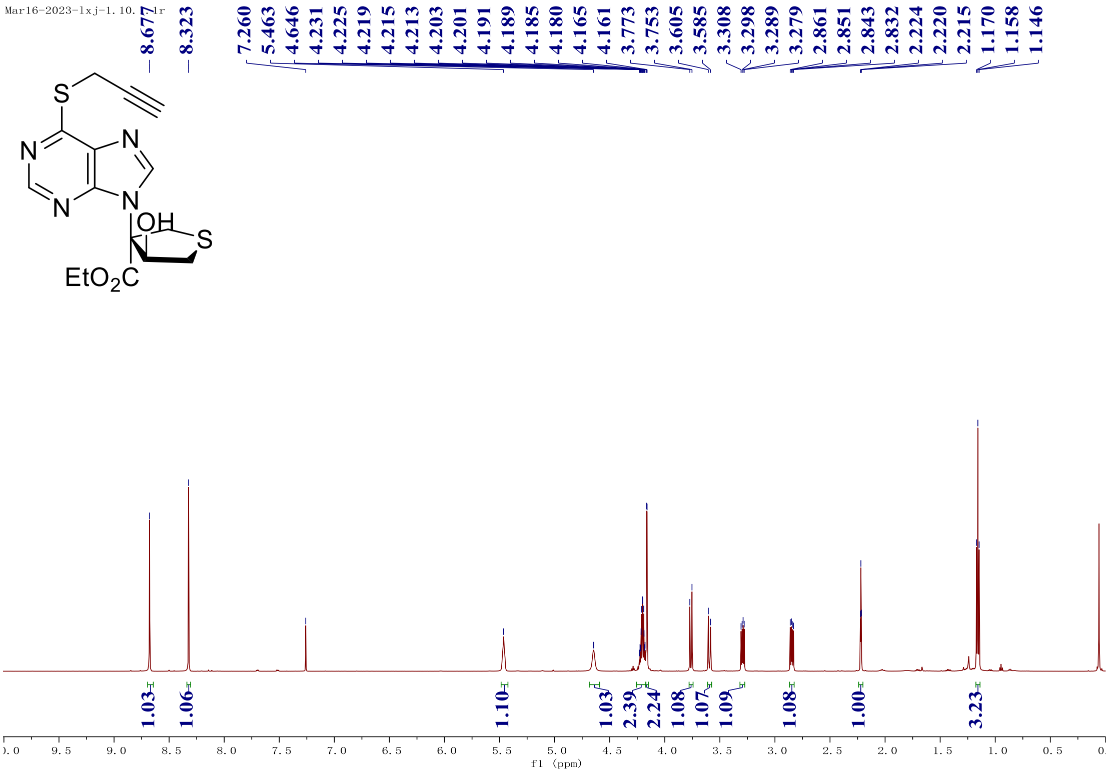

^{1}H NMR spectrum

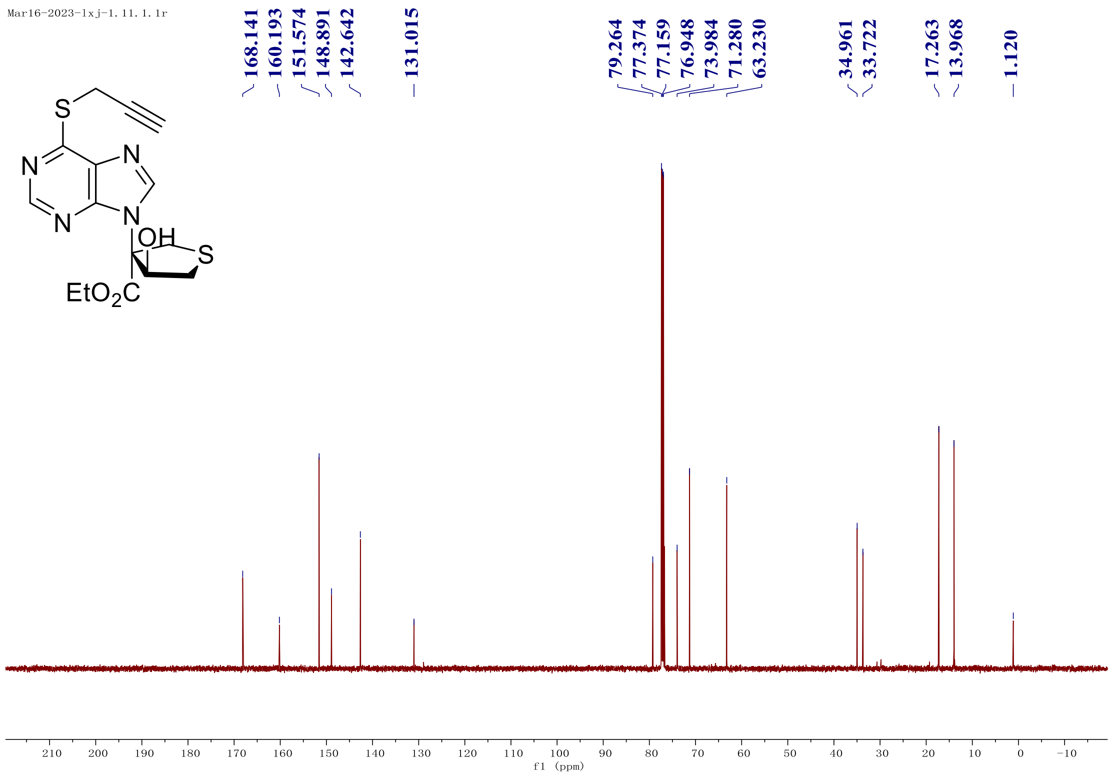

^{13}C NMR spectrum

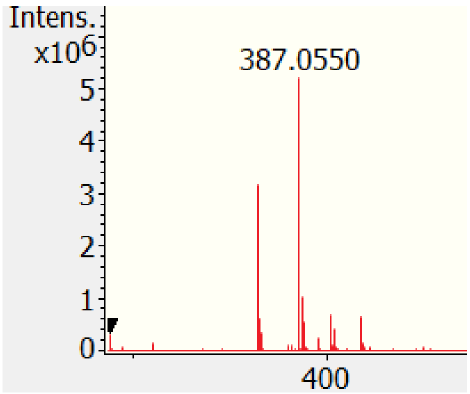

HR-ESIMS spectrum

(±)Ethyl-4-hydroxy-3-(6-(prop-2-yn-1-ylthio)-9*H*-purin-9-yl)tetrahydrothiophene-3-carboxylate (**24b**)

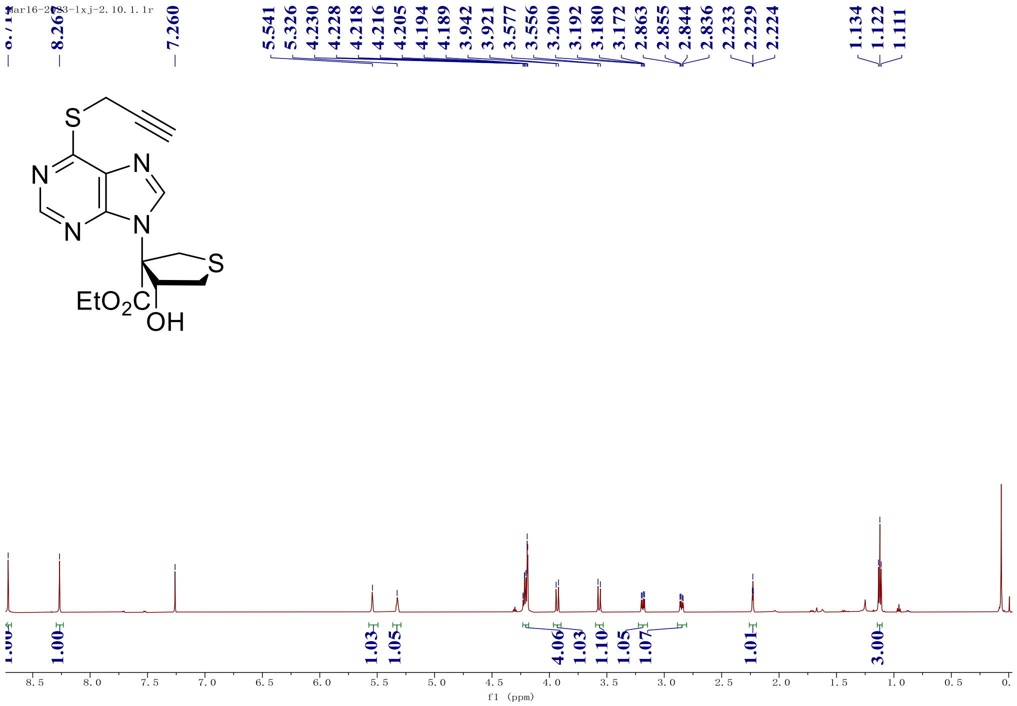

^{1}H NMR spectrum

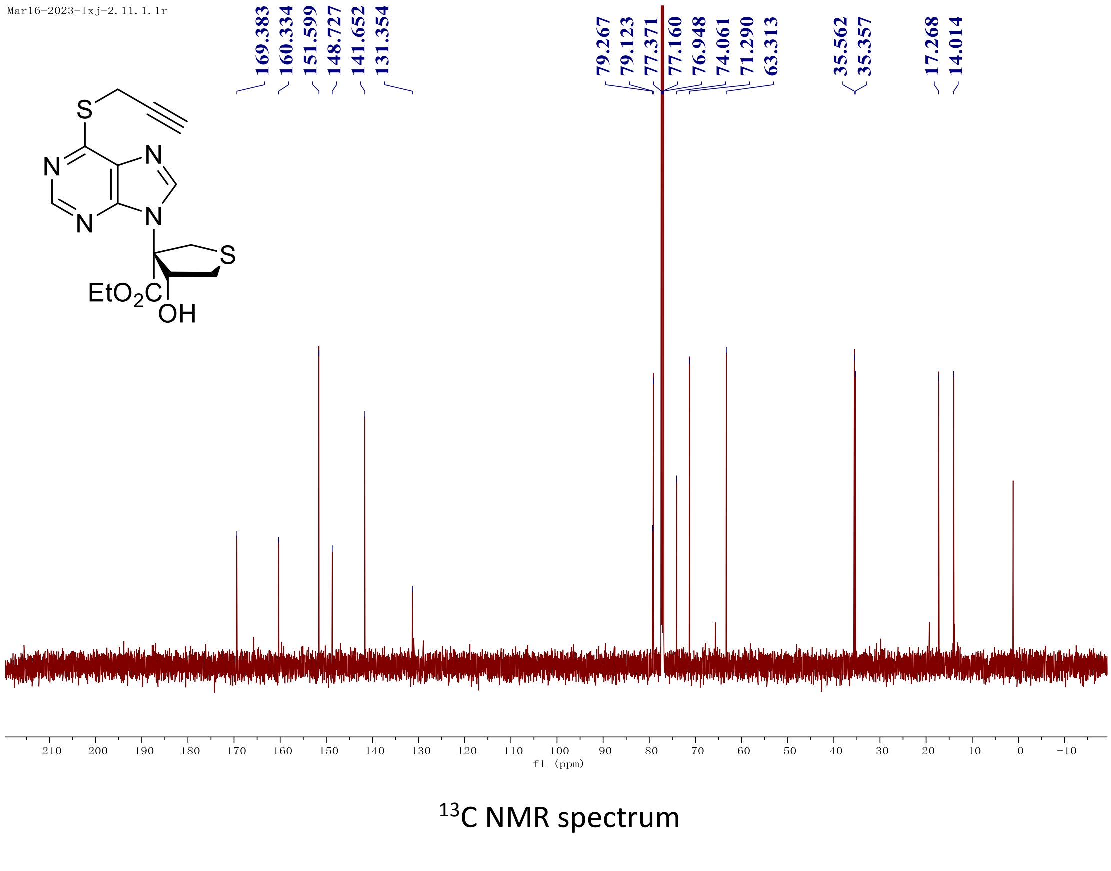

^{13}C NMR spectrum

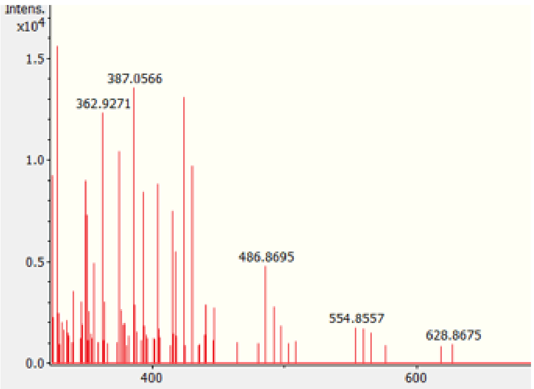

HR-ESIMS spectrum

(±)Ethyl-3-(6-(butylthio)-9*H*-purin-9-yl)-4-hydroxytetrahydrothiophene-3-carboxylate (**25a**)

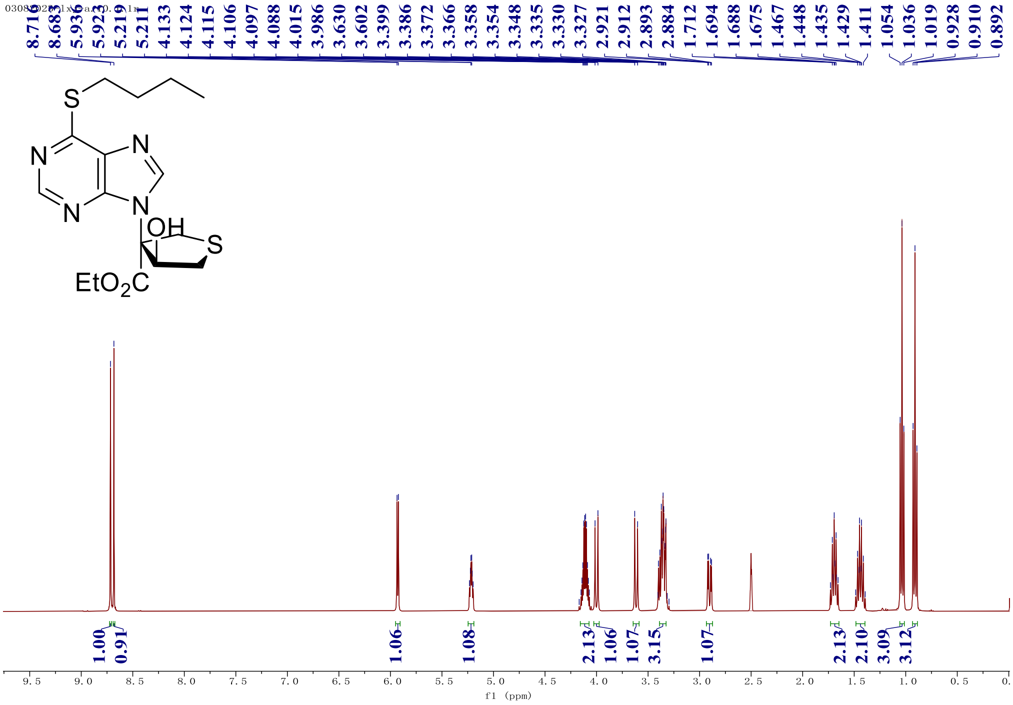

^{1}H NMR spectrum

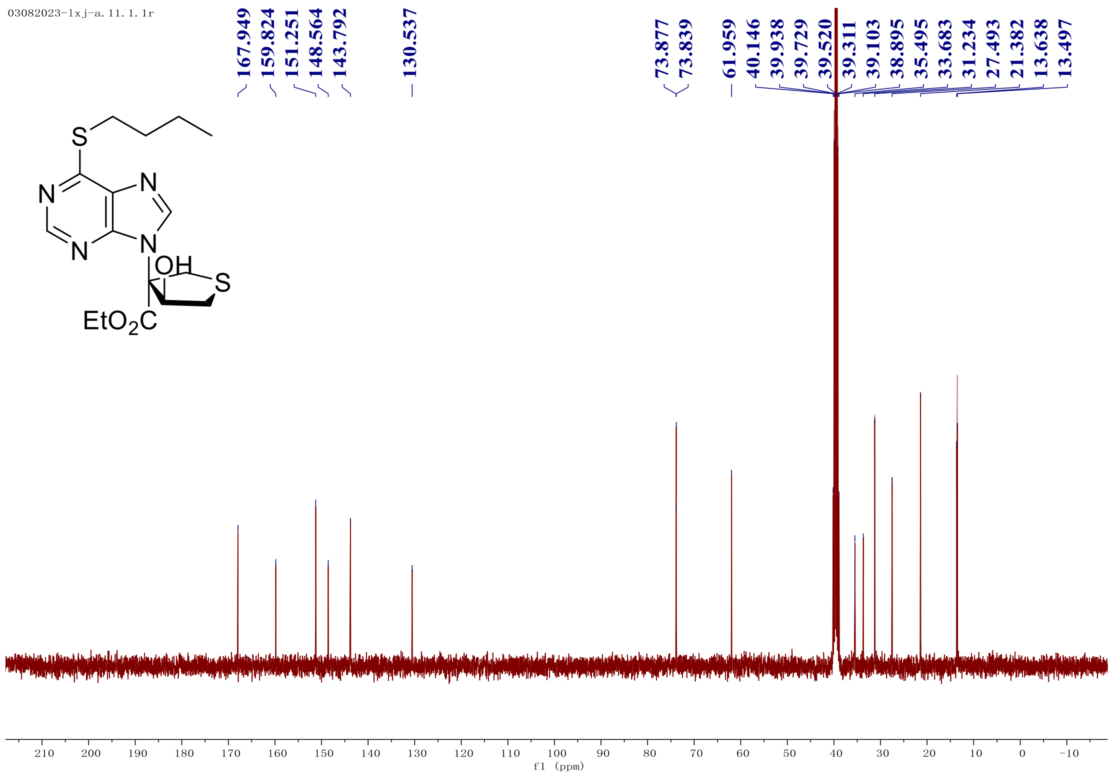

^{13}C NMR spectrum

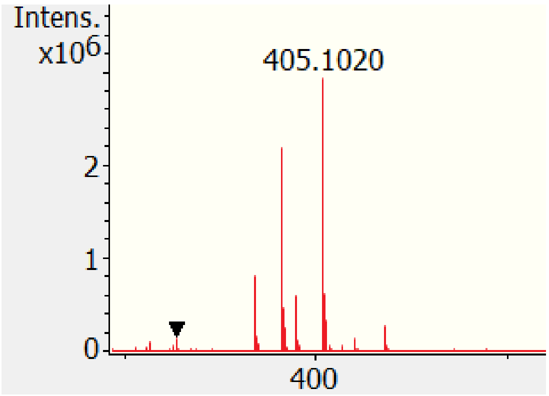

HR-ESIMS spectrum

(±)Ethyl-3-(6-(butylthio)-9*H*-purin-9-yl)-4-hydroxytetrahydrothiophene-3-carboxylate (**25b**)

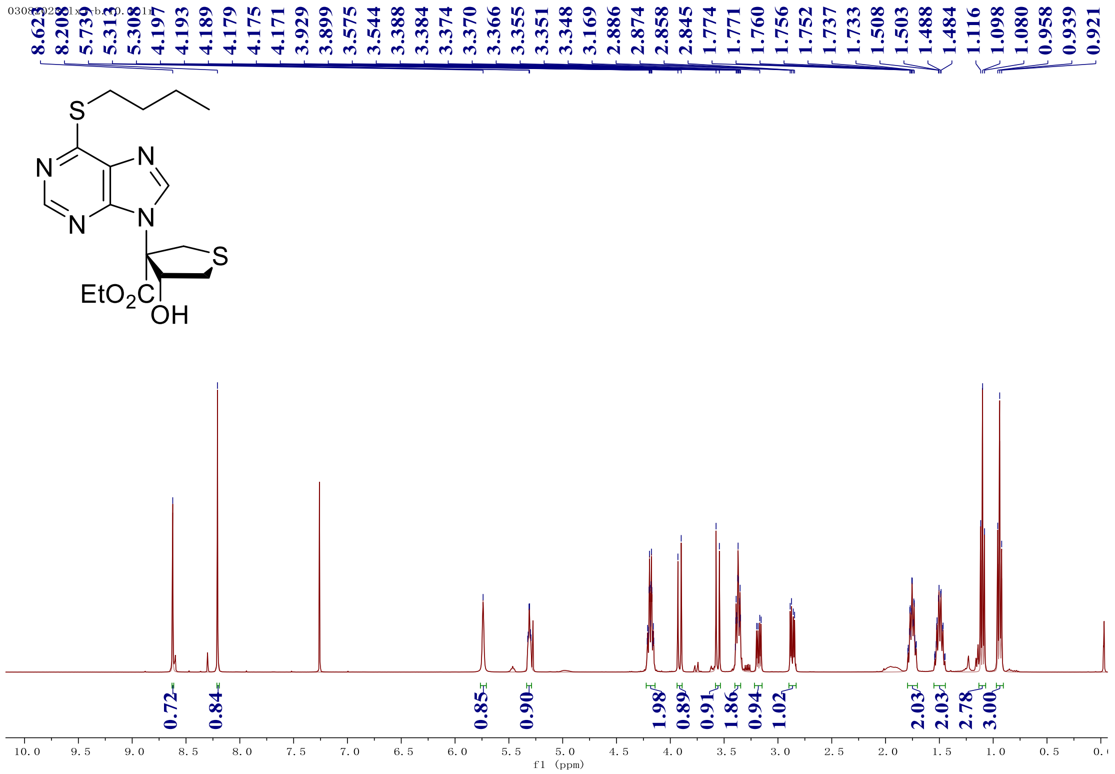

^{1}H NMR spectrum

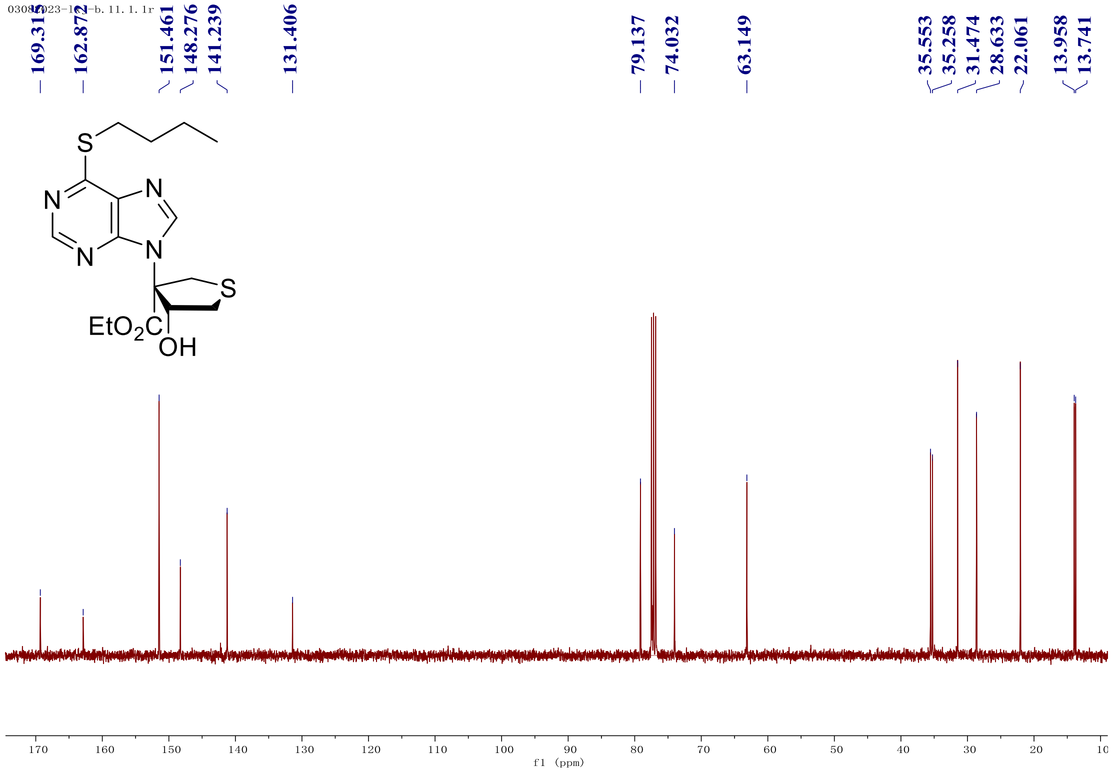

^{13}C NMR spectrum

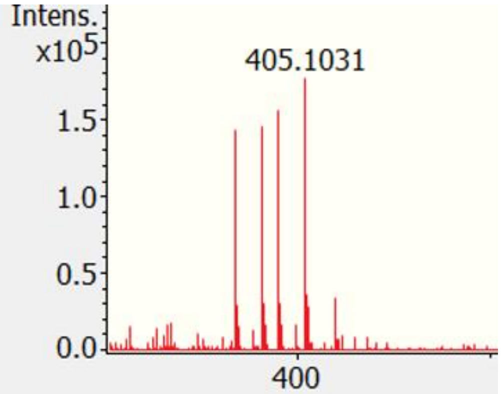

HR-ESIMS spectrum

(±)Ethyl-4-hydroxy-3-(6-(pentylthio)-9*H*-purin-9-yl)tetrahydrothiophene-3-carboxylate (**26a**)

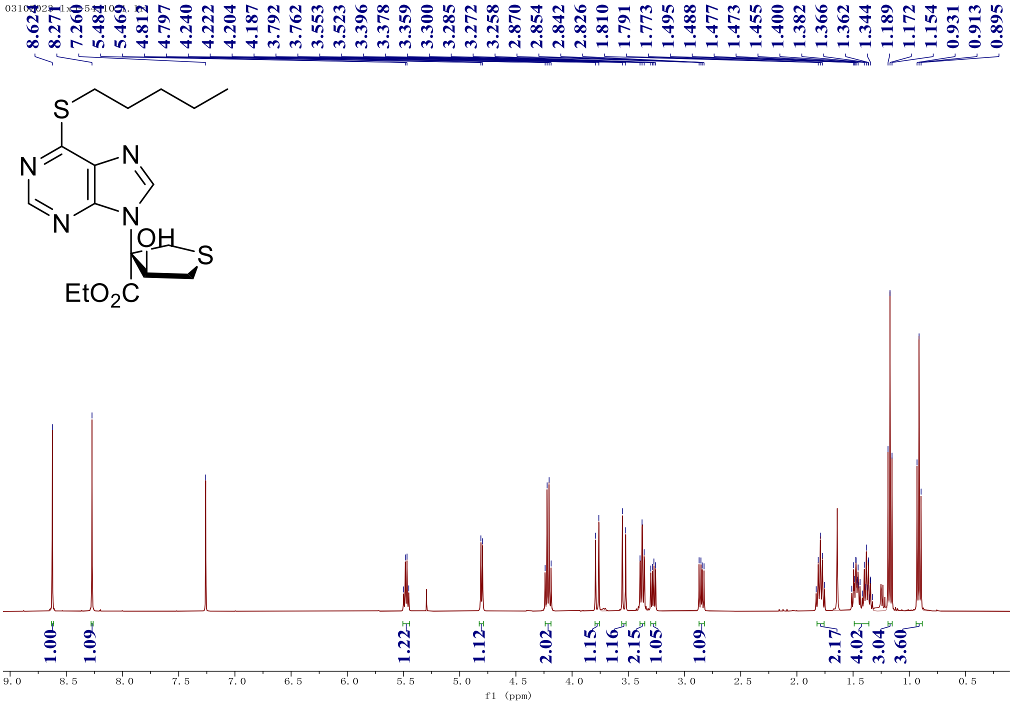

^1H NMR spectrum

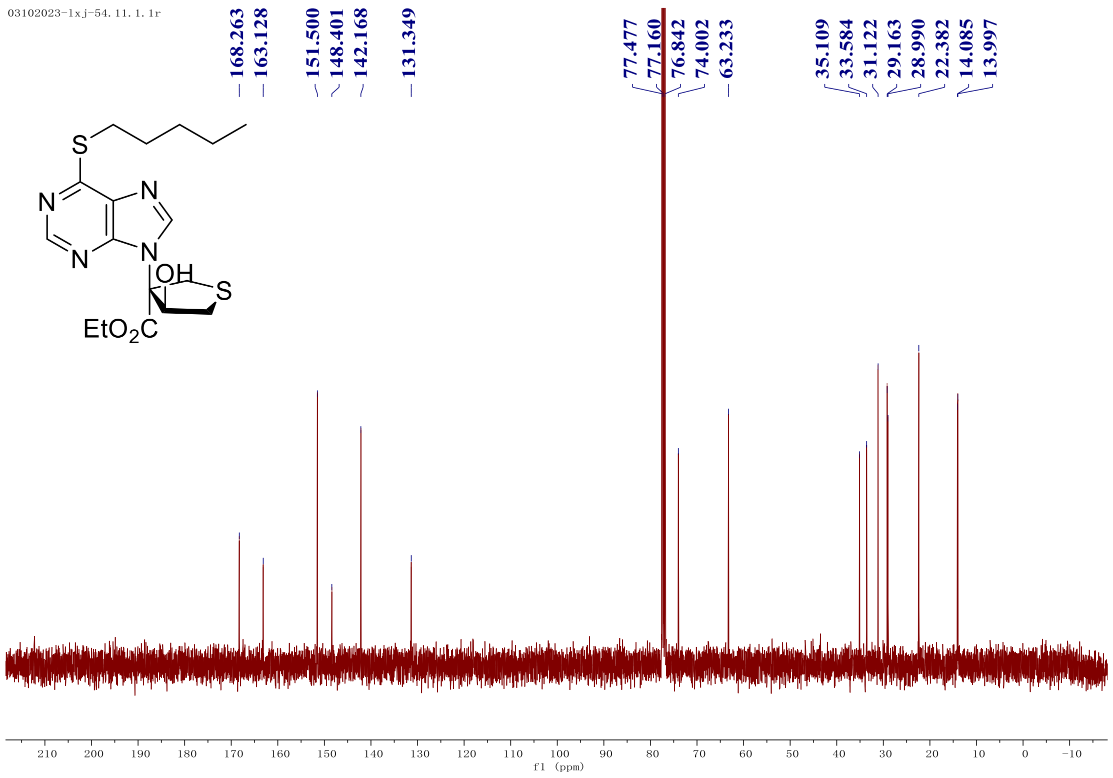

^{13}C NMR spectrum

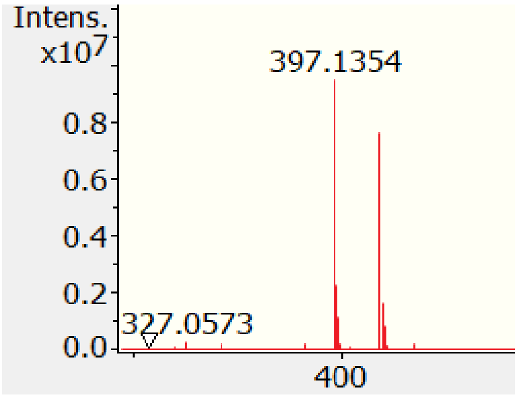

HR-ESIMS spectrum

(±)Ethyl-4-hydroxy-3-(6-(pentylthio)-9*H*-purin-9-yl)tetrahydrothiophene-3-carboxylate (**26b**)

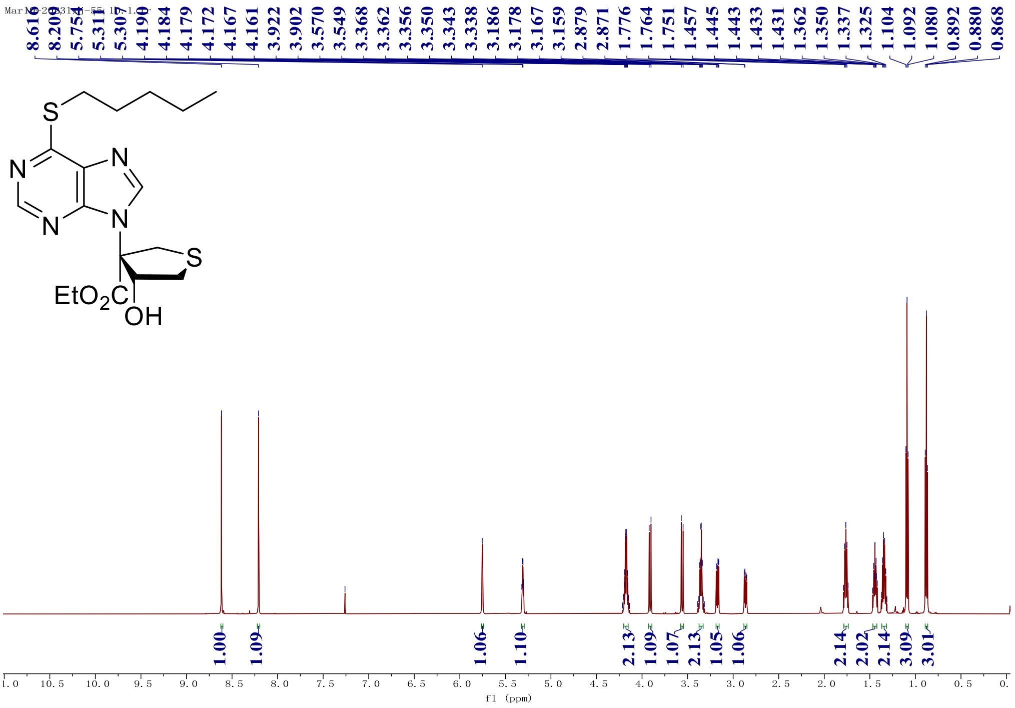

^{1}H NMR spectrum

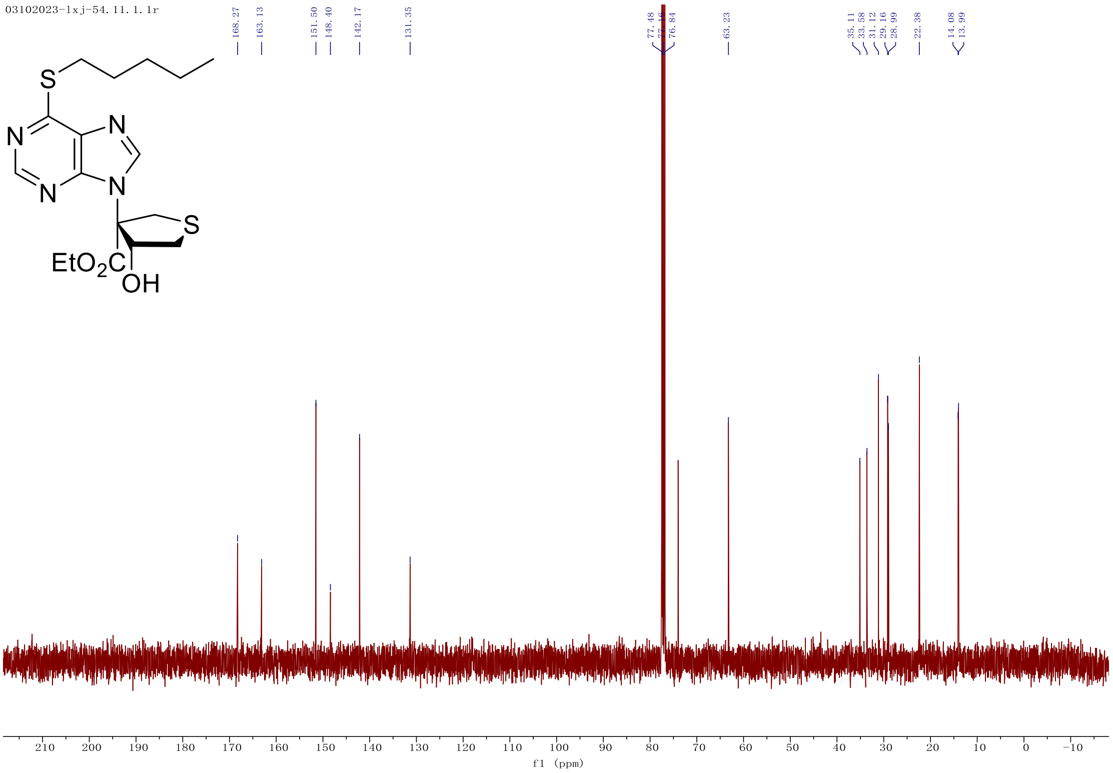

^{13}C NMR spectrum

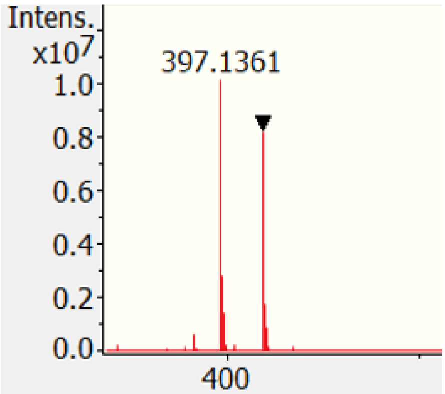

HR-ESIMS spectrum

(±)Ethy-3-(6-(benzylthio)-9*H*-purin-9-yl)-4-hydroxytetrahydrothiophene-3-carboxylate (**27a**)

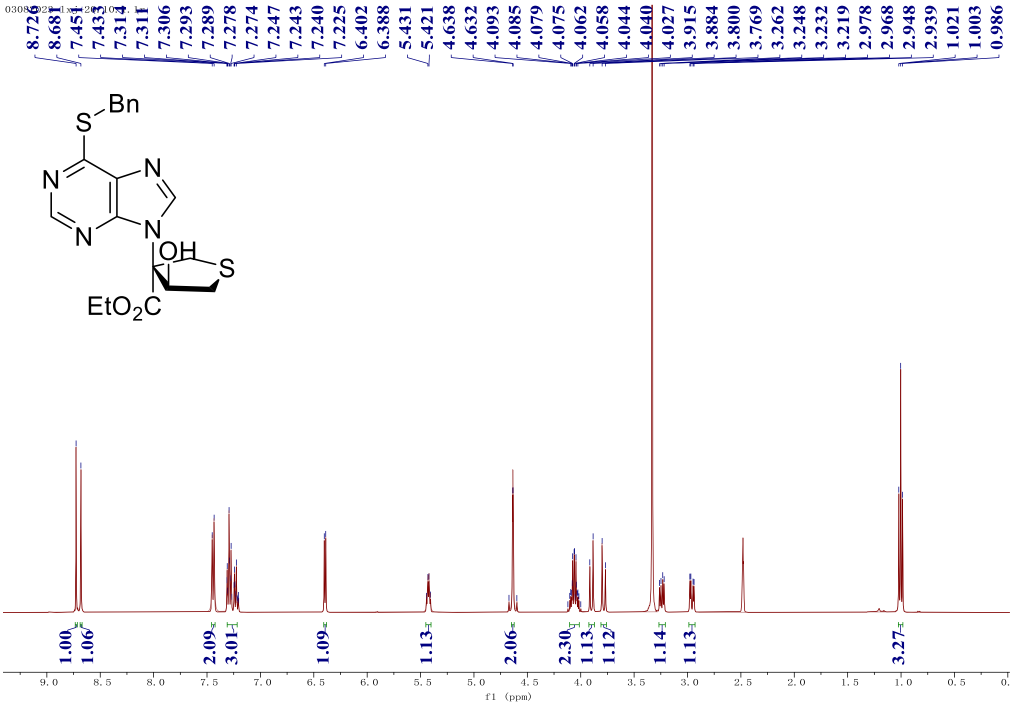

^{1}H NMR spectrum

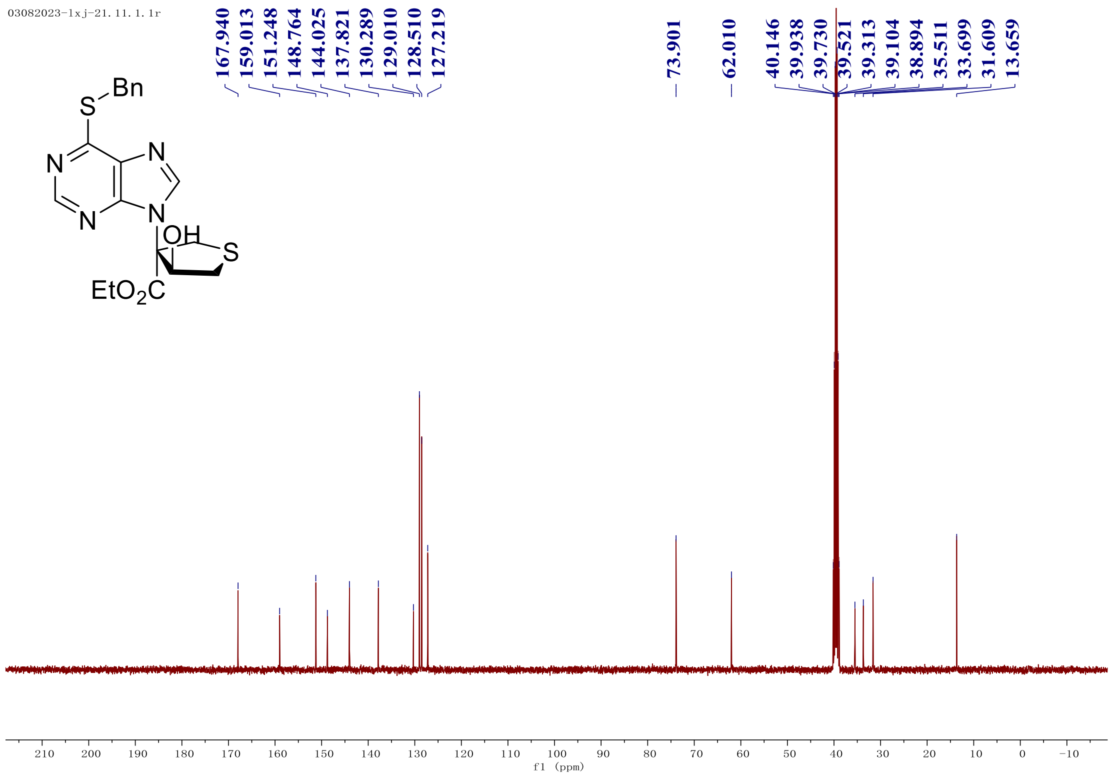

^{13}C NMR spectrum

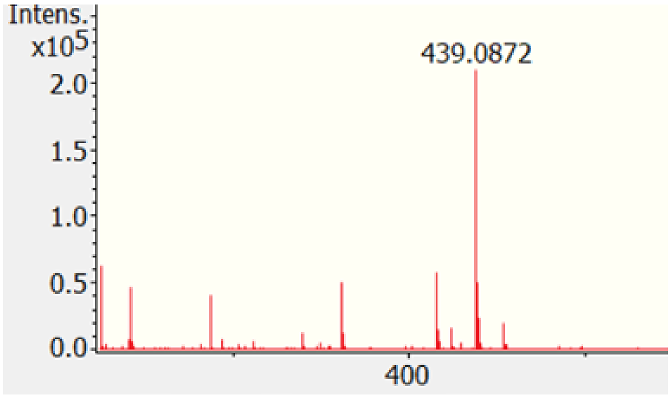

HR-ESIMS spectrum

(±)Ethy-3-(6-(benzylthio)-9*H*-purin-9-yl)-4-hydroxytetrahydrothiophene-3-carboxylate (**27b**)

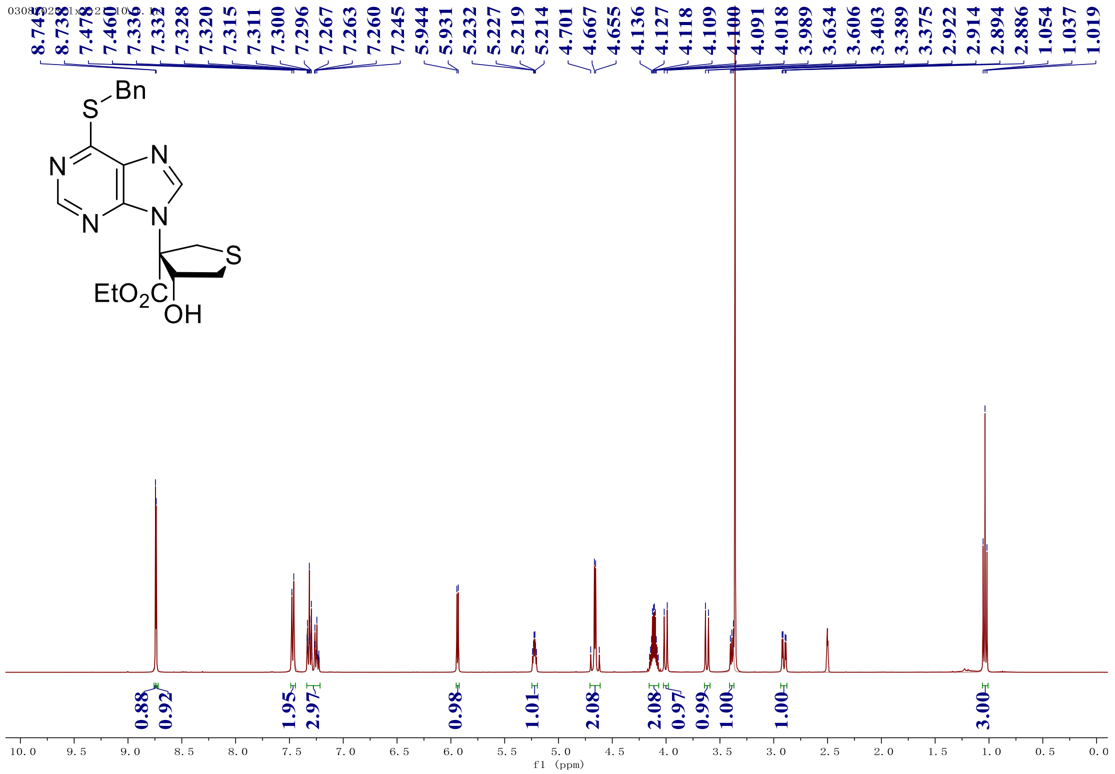

^1H NMR spectrum

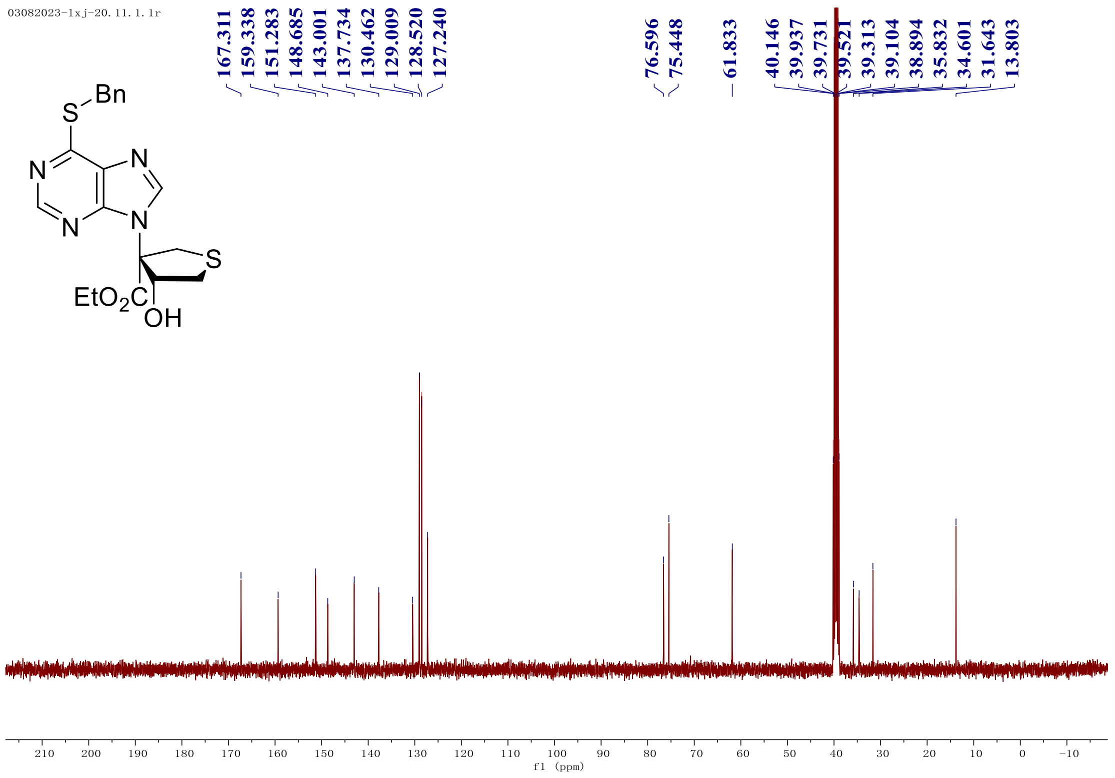

^{13}C NMR spectrum

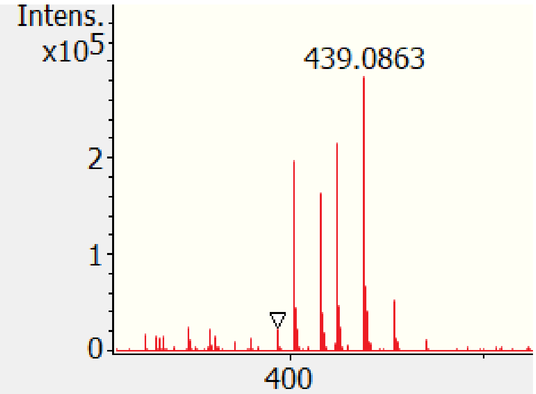

HR-ESIMS spectrum

(±)Ethyl-3-(6-((3-fluorophenyl)thio)-9*H*-purin-9-yl)-4-hydroxytetrahydrothiophene-3-carboxylate (**28a**)

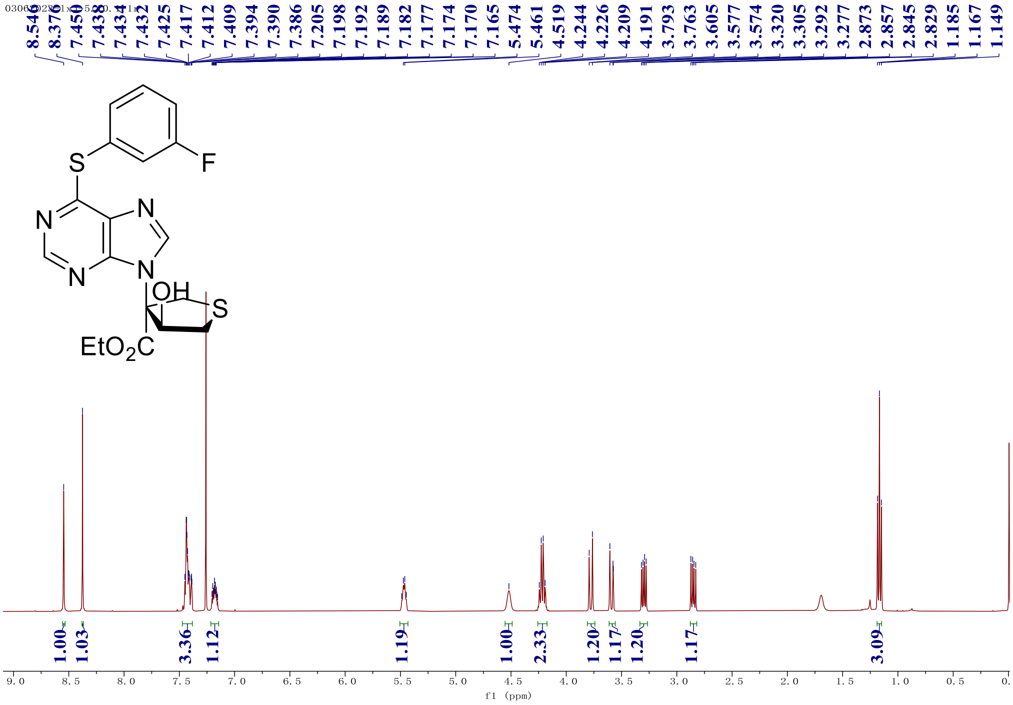

^{1}H NMR spectrum

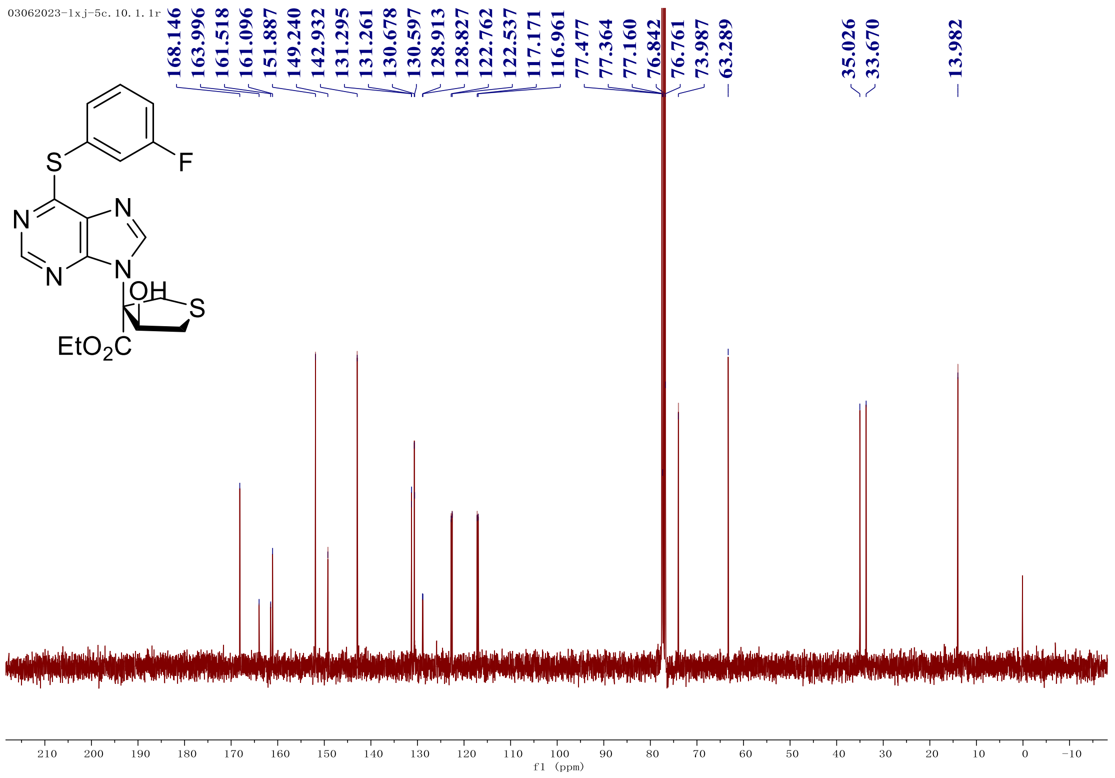

^{13}C NMR spectrum

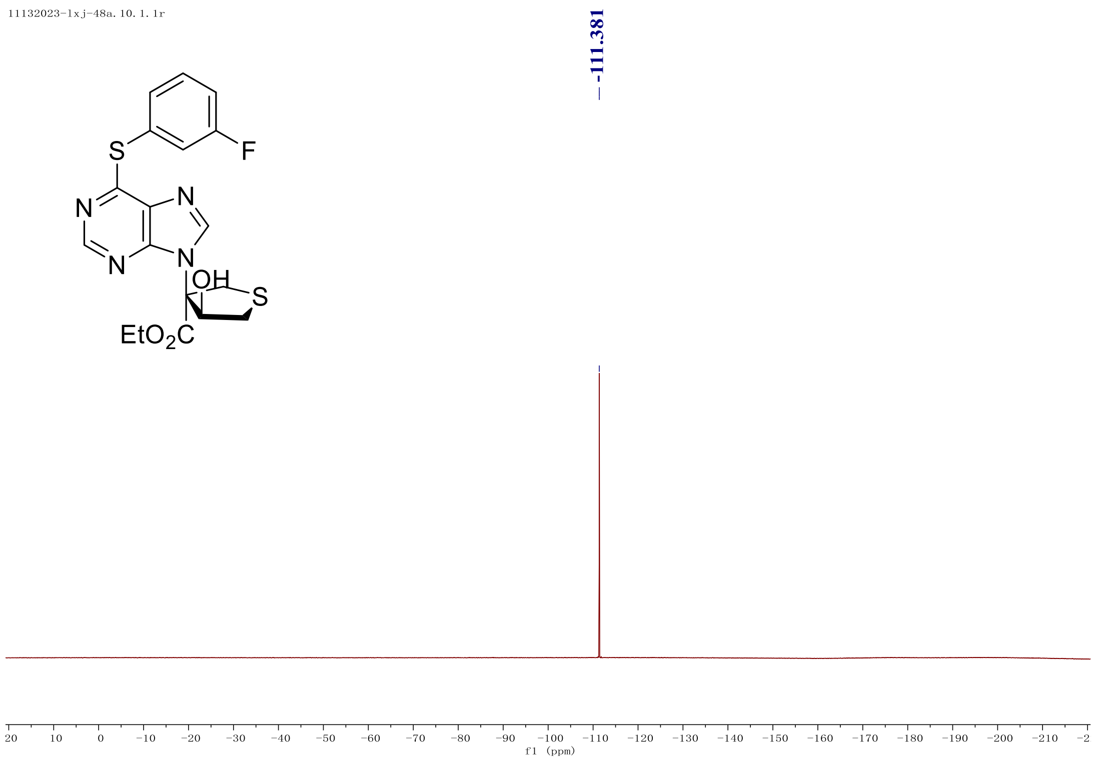

^{19}F NMR spectrum

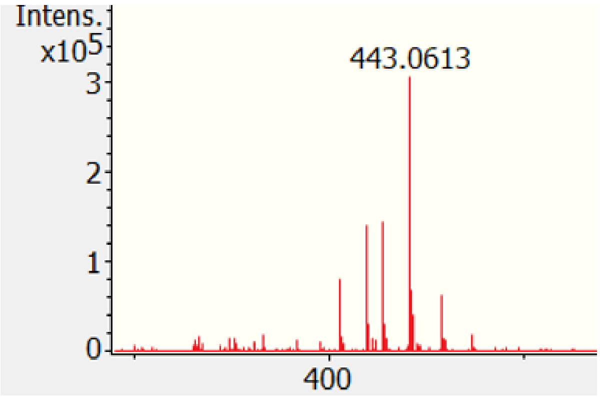

HR-ESIMS spectrum

(±)Ethyl-3-(6-((3-fluorophenyl)thio)-9*H*-purin-9-yl)-4-hydroxytetrahydrothiophene-3-carboxylate (**28b**)

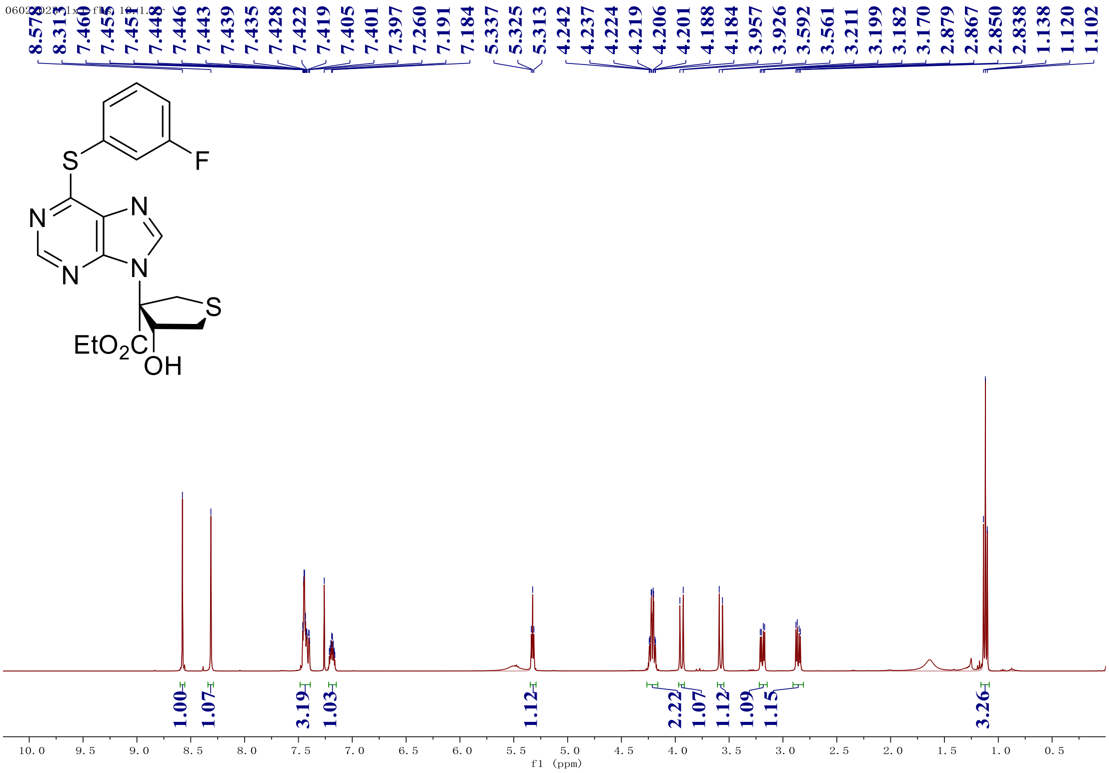

^{1}H NMR spectrum

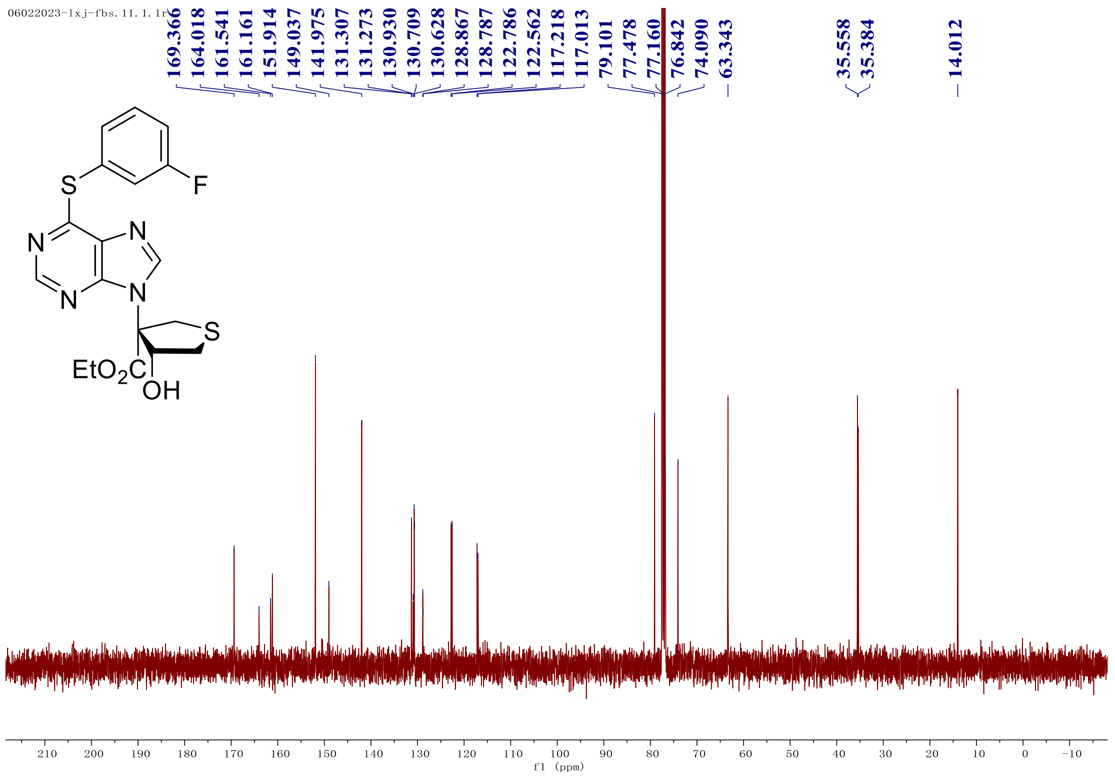

^{13}C NMR spectrum

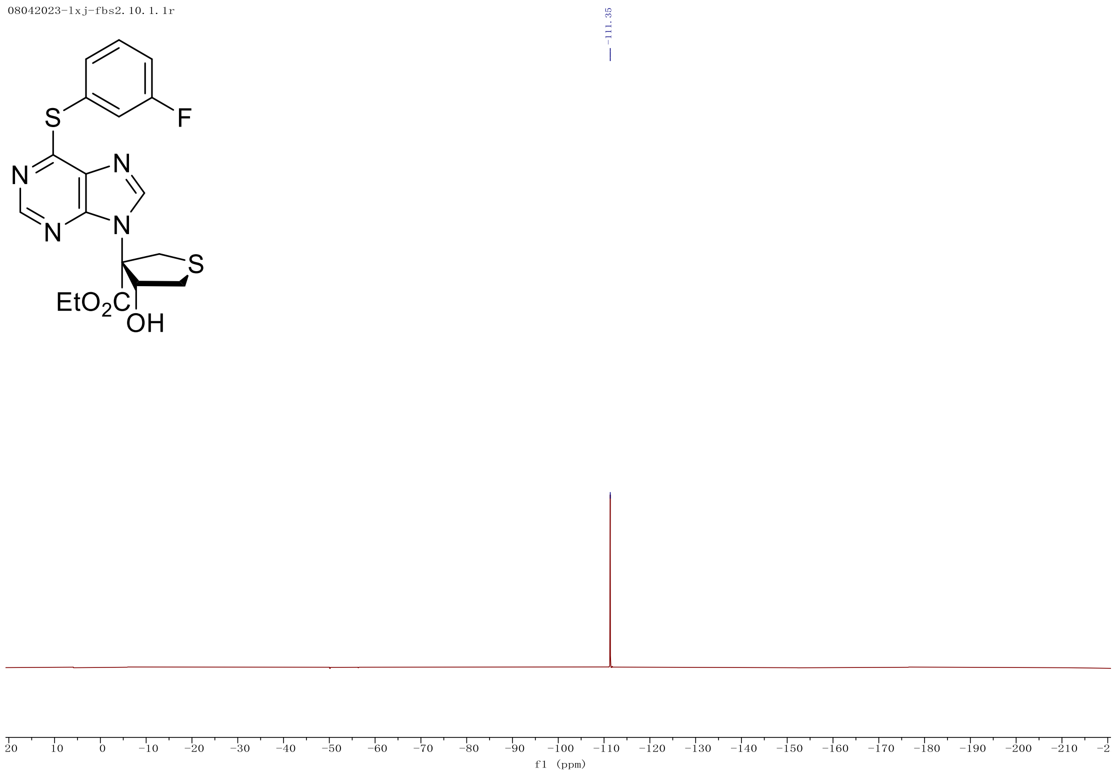

^{19}F NMR spectrum

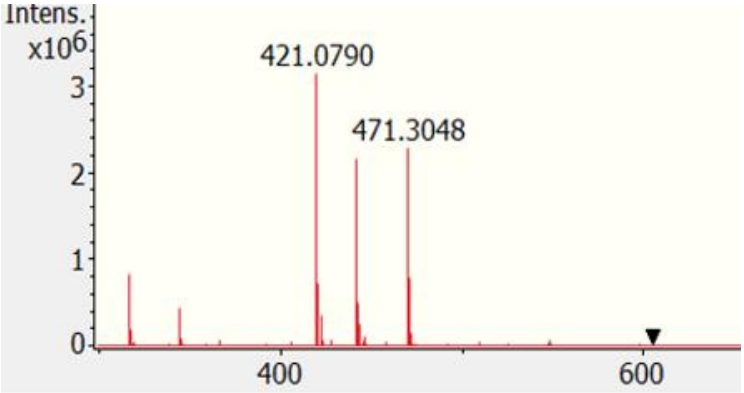

HR-ESIMS spectrum

(±)Ethyl-3-(6-((4-(tert-butyl) phenyl) thio)-9*H*-purin-9-yl)-4-hydroxytetrahydrothiophene-3-carboxylate (**29a**)

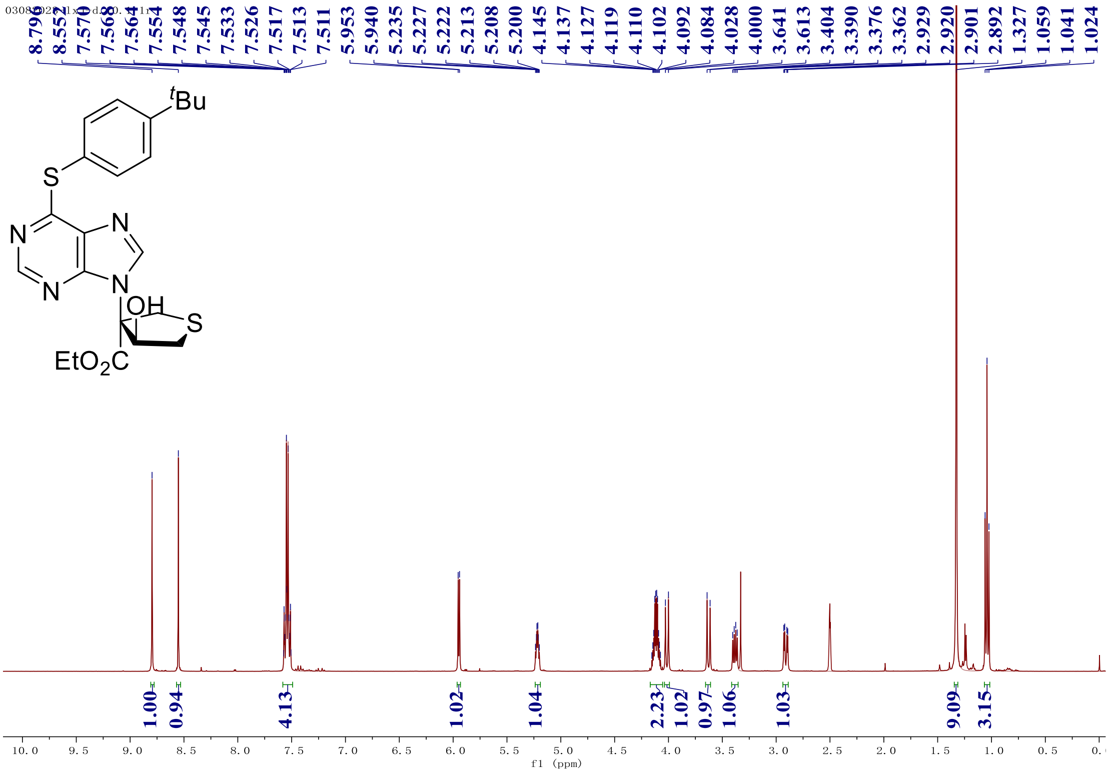

^{1}H NMR spectrum

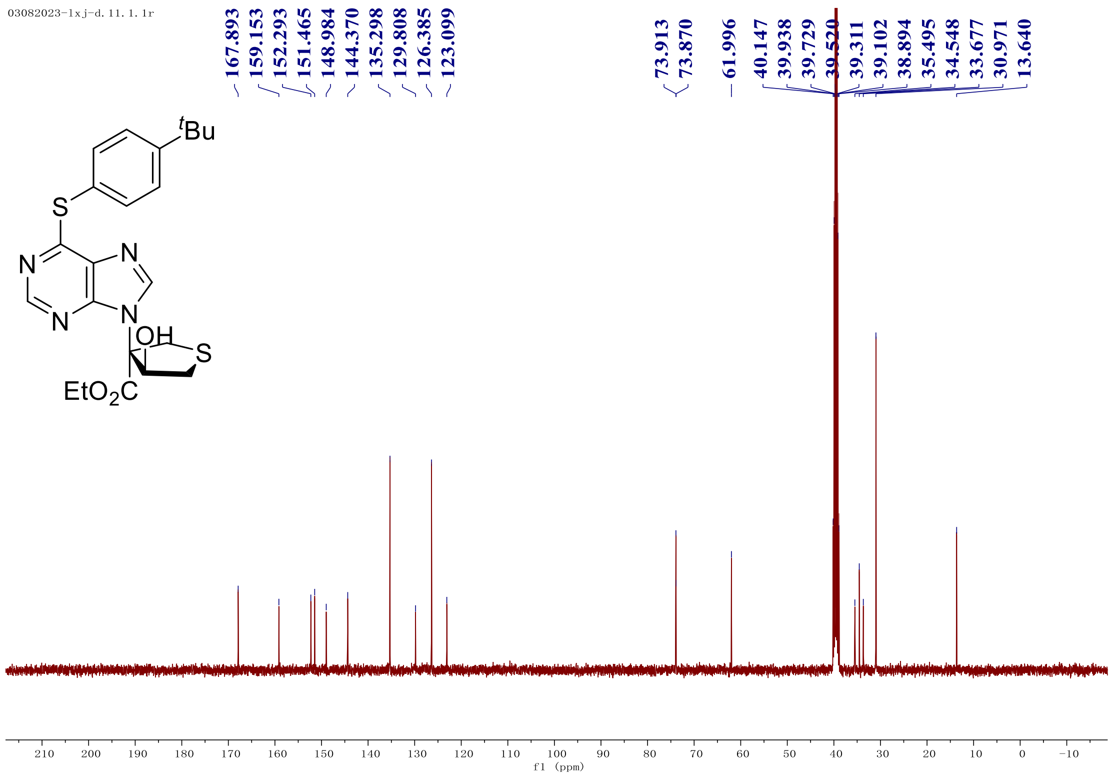

^{13}C NMR spectrum

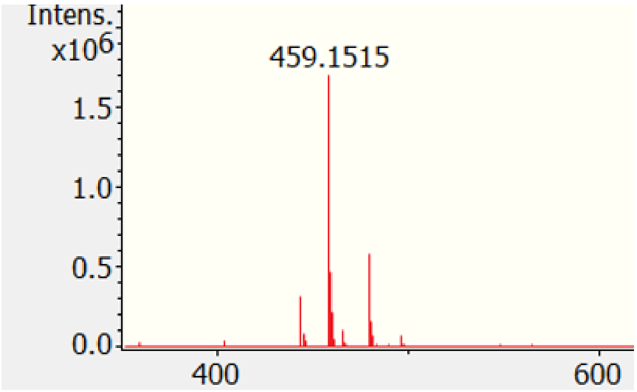

HR-ESIMS spectrum

(±)Ethyl-3-(6-((4-(tert-butyl) phenyl) thio)-9*H*-purin-9-yl)-4-hydroxytetrahydrothiophene-3-carboxylate (**29b**)

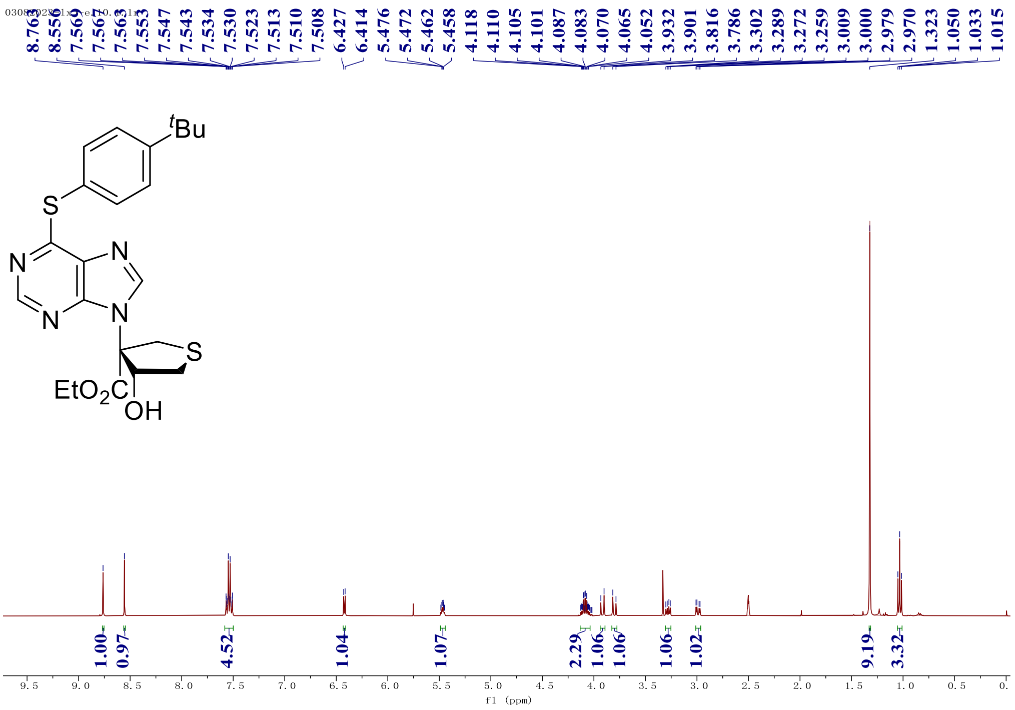

^{1}H NMR spectrum

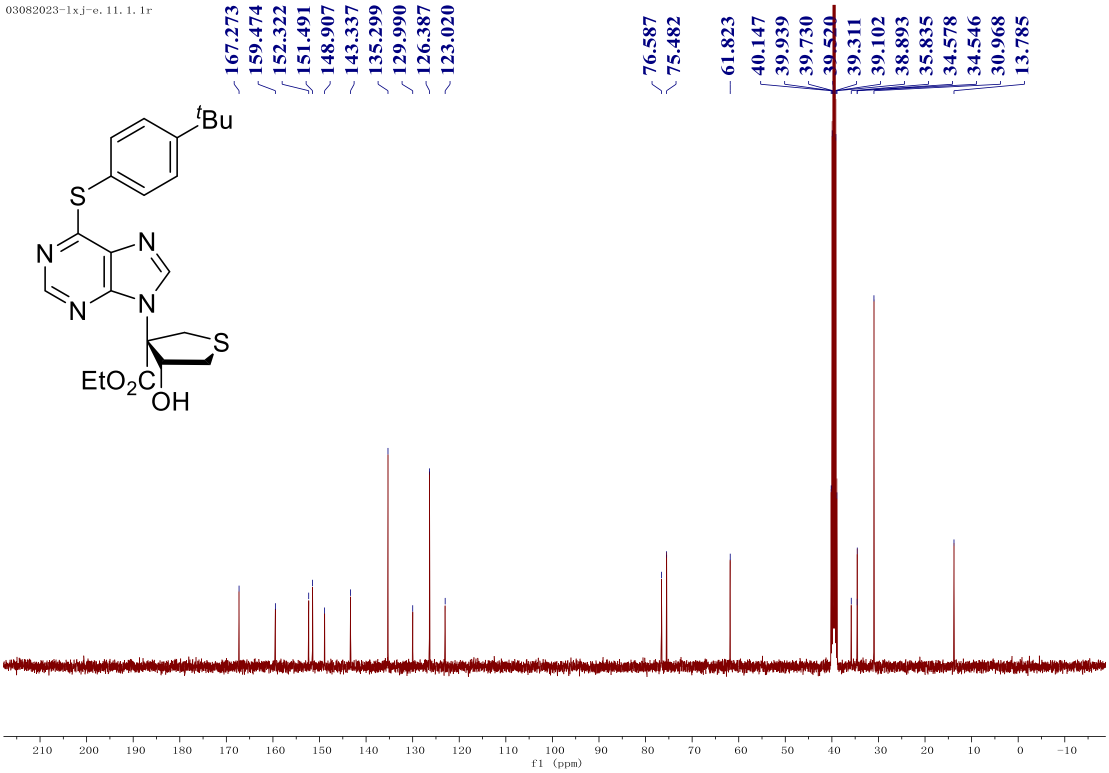

^{13}C NMR spectrum

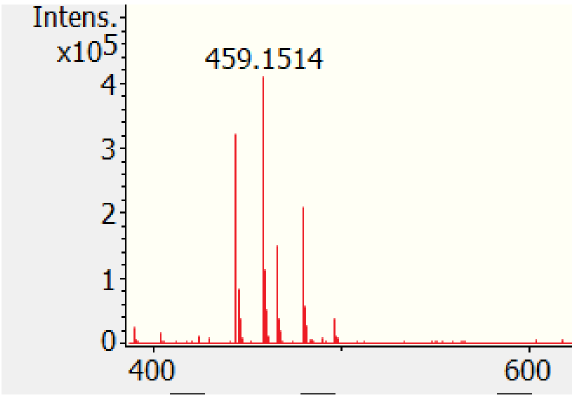

HR-ESIMS spectrum

(±)Ethyl-3-(6-((3-fluoropropyl) thio)-9*H*-purin-9-yl)-4-hydroxytetrahydrothiophene-3-carboxylate (**30a**)

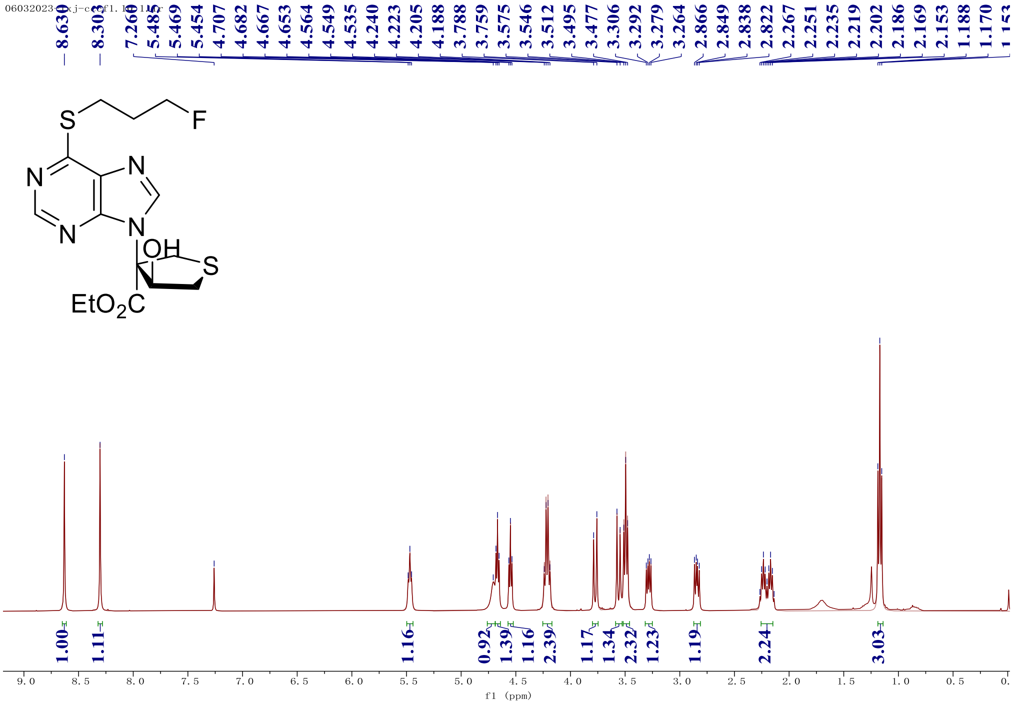

^{1}H NMR spectrum

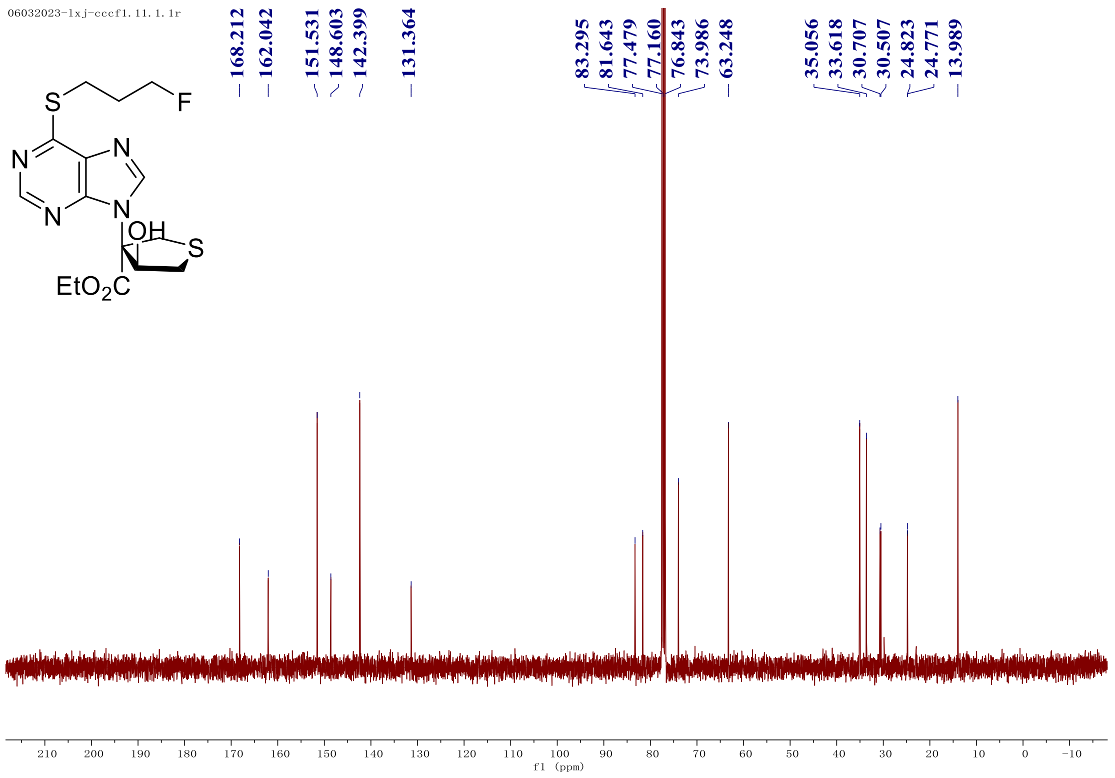

^{13}C NMR spectrum

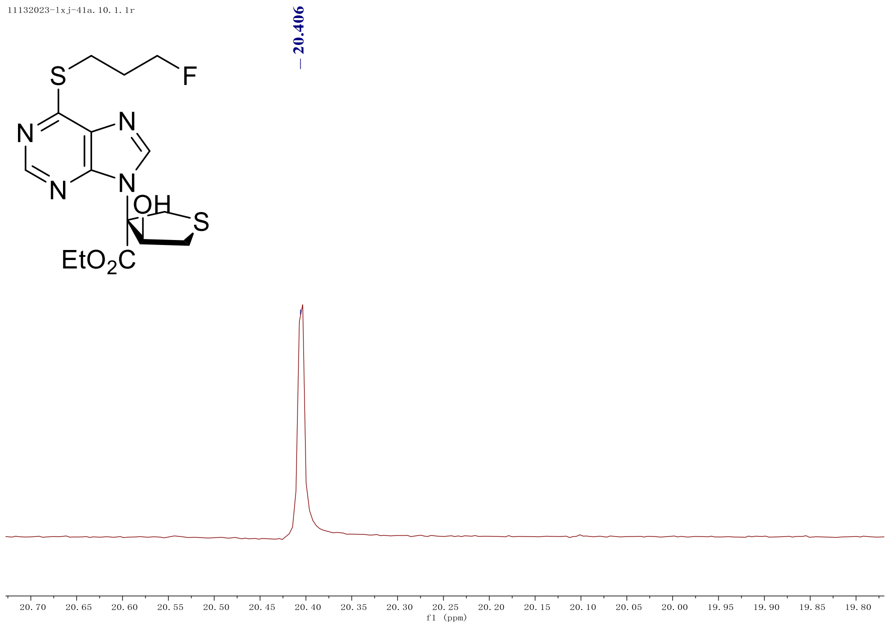

^{19}F NMR spectrum

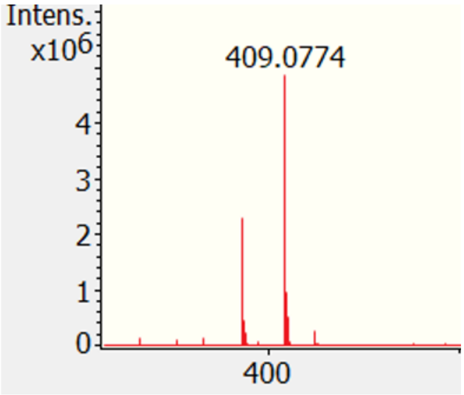

HR-ESIMS spectrum

(±)Ethyl-3-(6-((3-fluoropropyl) thio)-9*H*-purin-9-yl)-4-hydroxytetrahydrothiophene-3-carboxylate (**30b**)

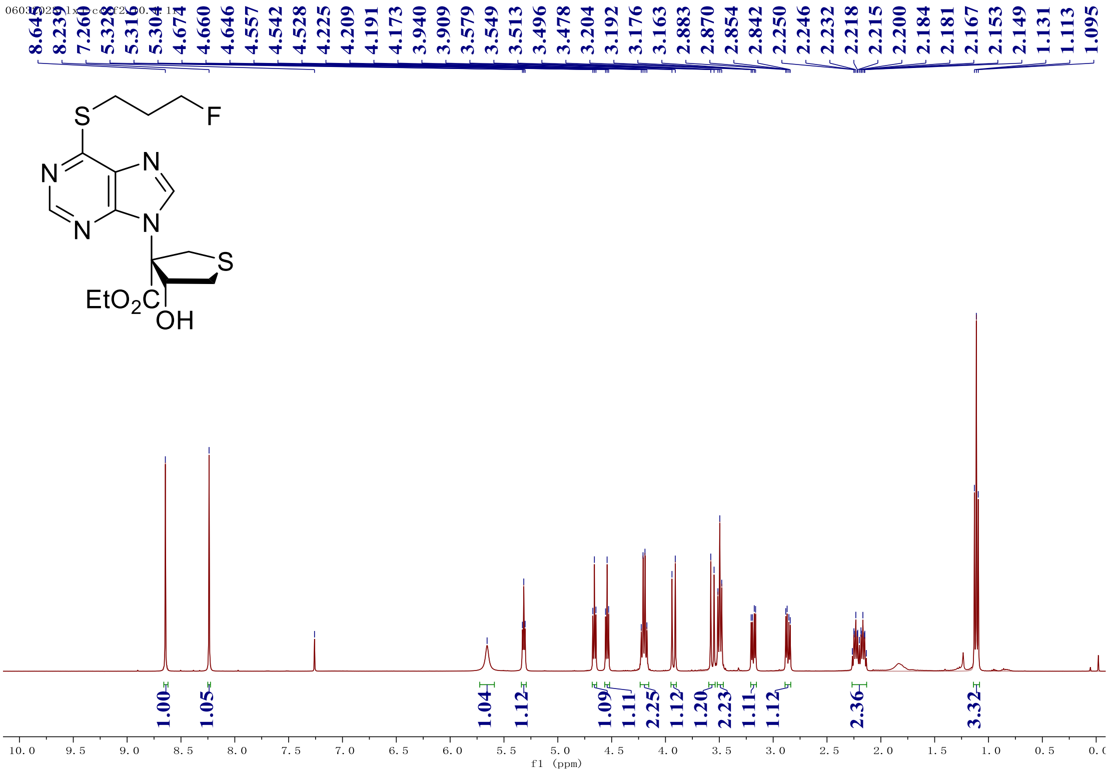

^{1}H NMR spectrum

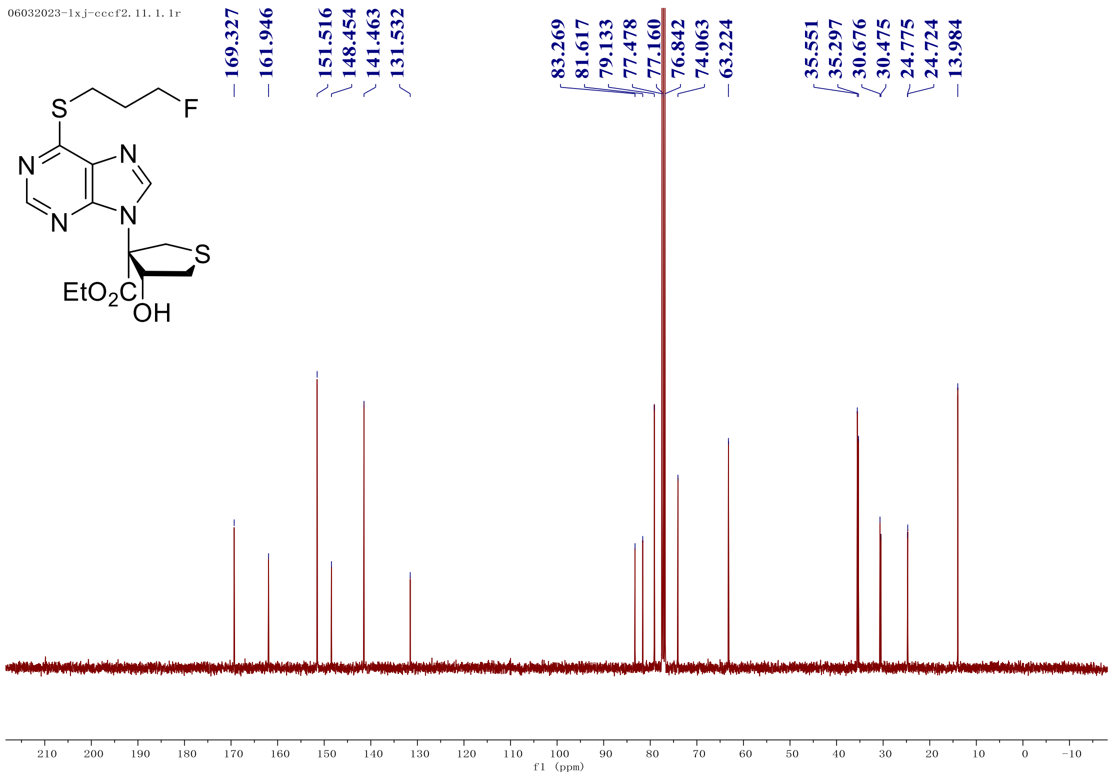

^{13}C NMR spectrum

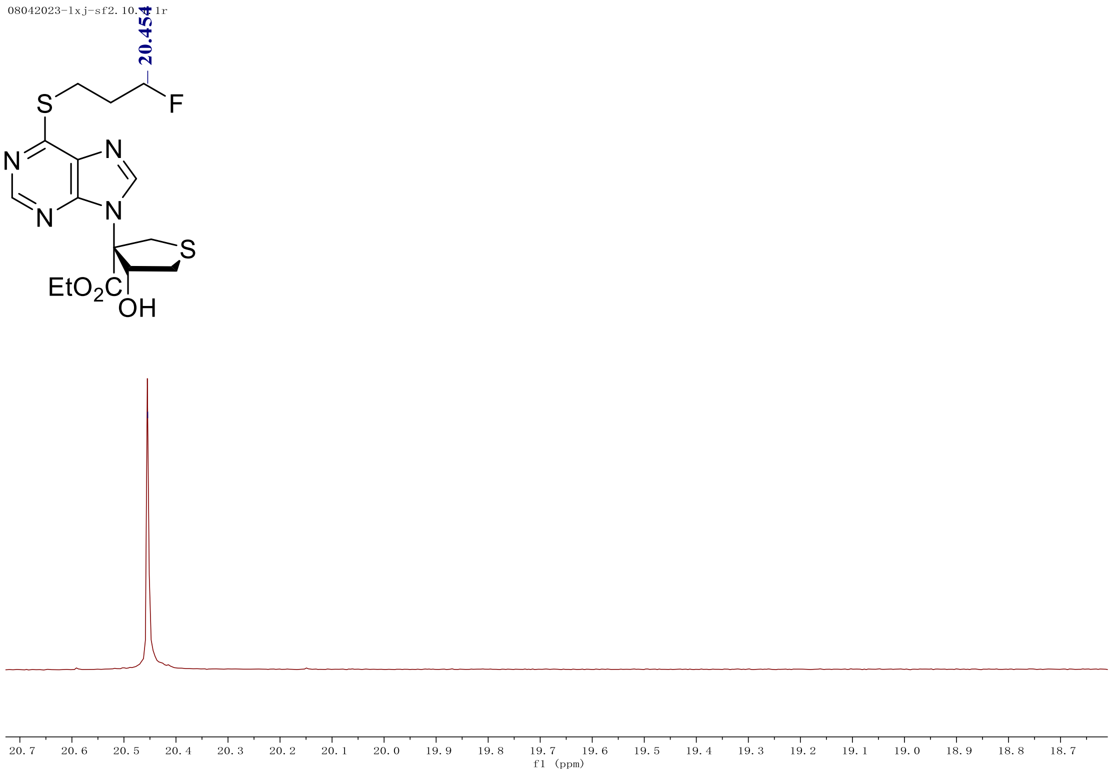

^{19}F NMR spectrum

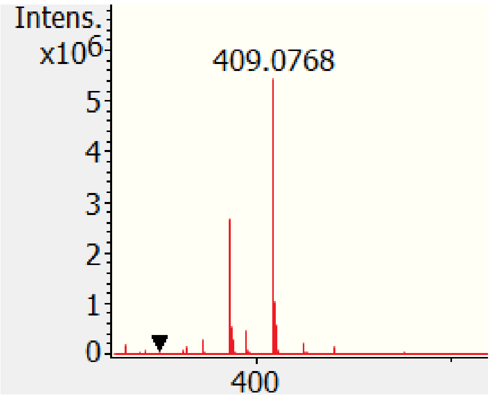

HR-ESIMS spectrum

(±)Ethyl-3-(6-((3-chloropropyl) thio)-9*H*-purin-9-yl)-4-hydroxytetrahydrothiophene-3-carboxylate (**31a**)

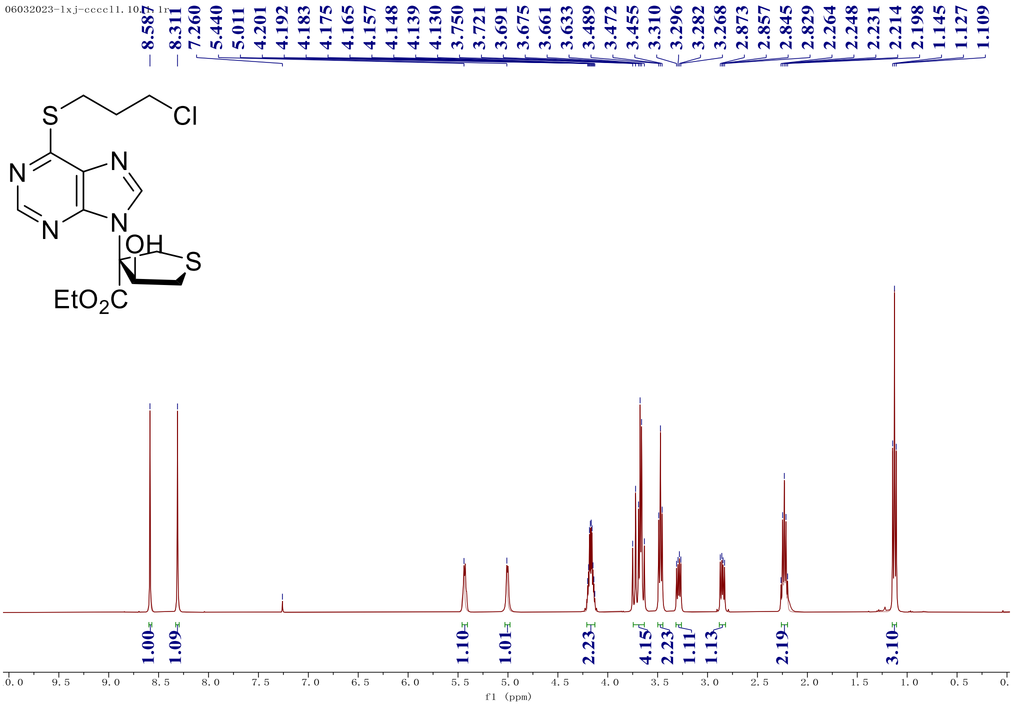

^{1}H NMR spectrum

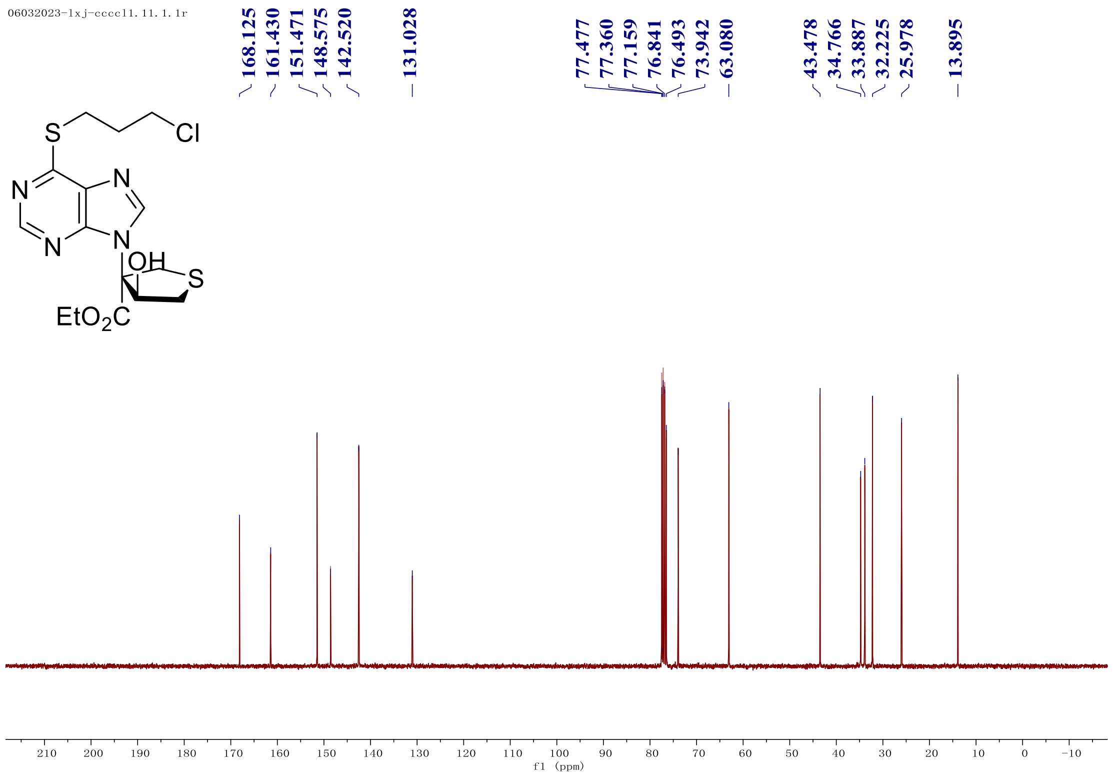

^{13}C NMR spectrum

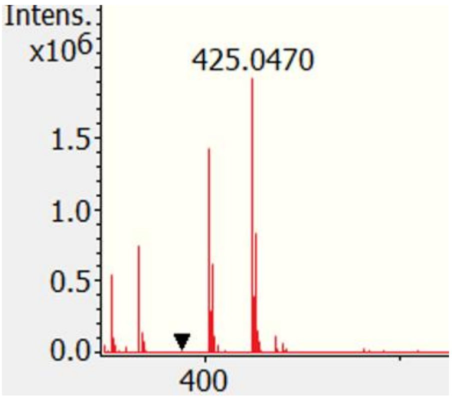

HR-ESIMS spectrum

(±)Ethyl-3-(6-((3-chloropropyl) thio)-9*H*-purin-9-yl)-4-hydroxytetrahydrothiophene-3-carboxylate (**31b**)

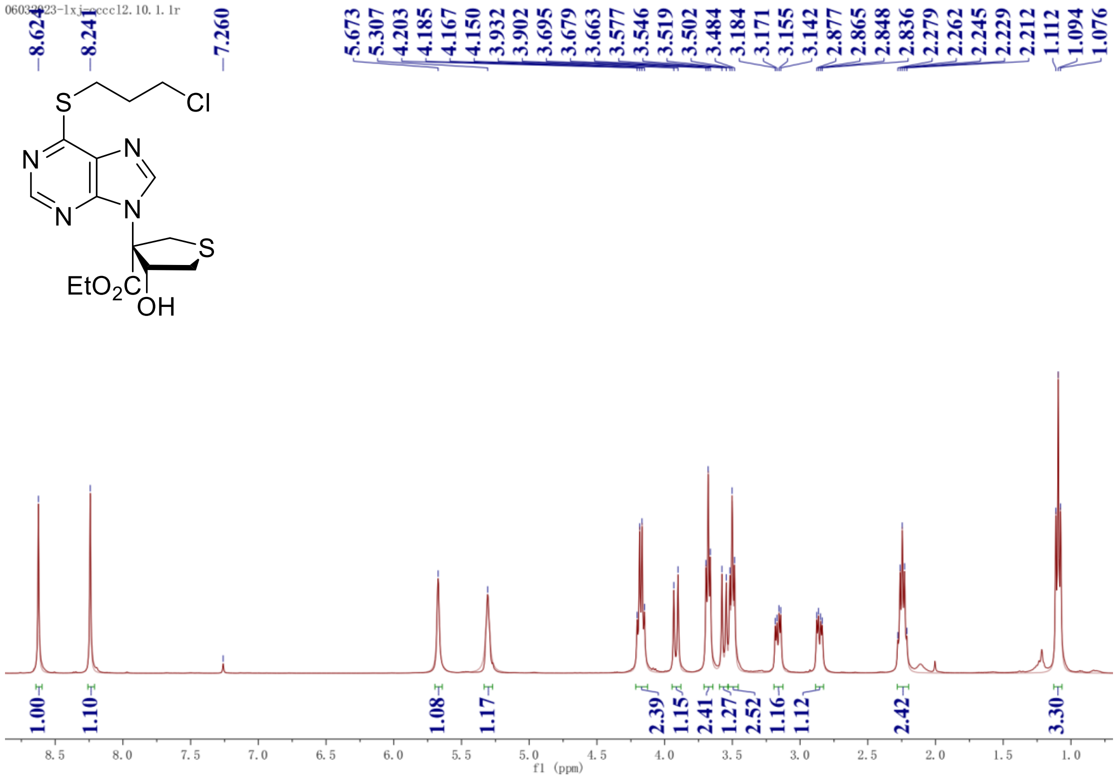

^{1}H NMR spectrum

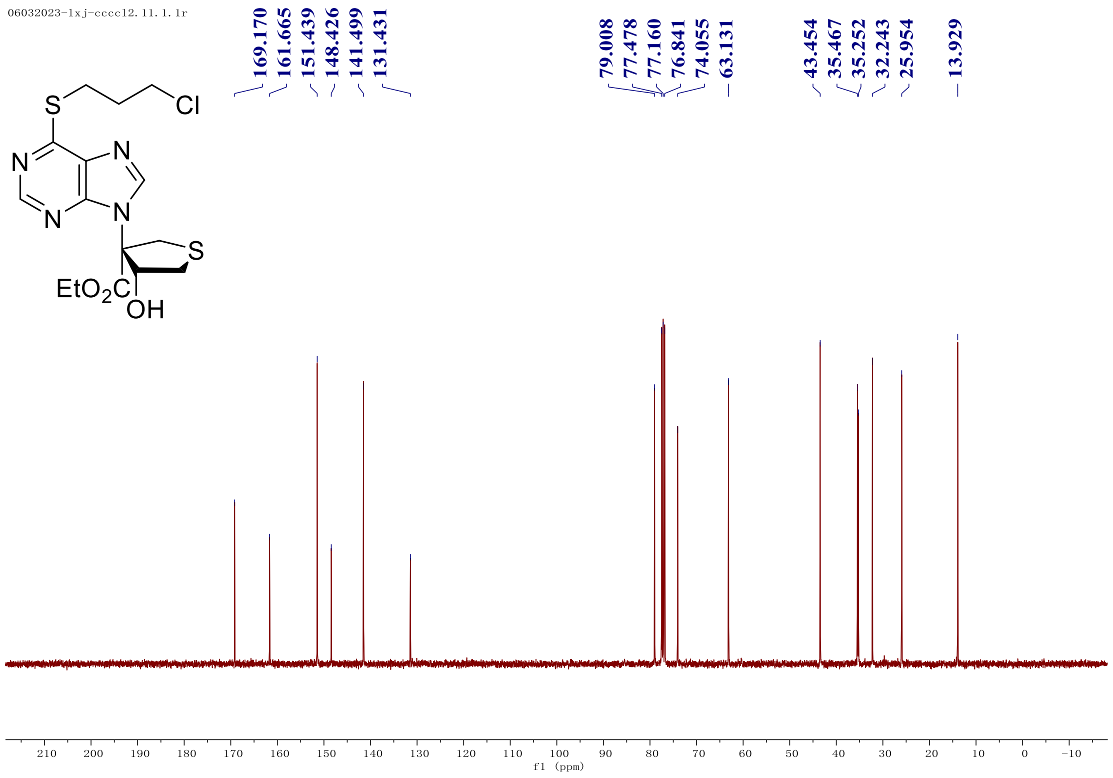

^{13}C NMR spectrum

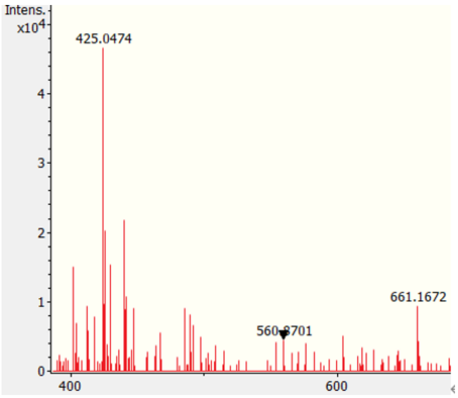

HR-ESIMS spectrum

(±)Ethyl-3-(2-amino-6-(piperidin-1-yl)-9*H*-purin-9-yl)-4-hydroxytetrahydrothiophene-3-carboxylate (**32a**)

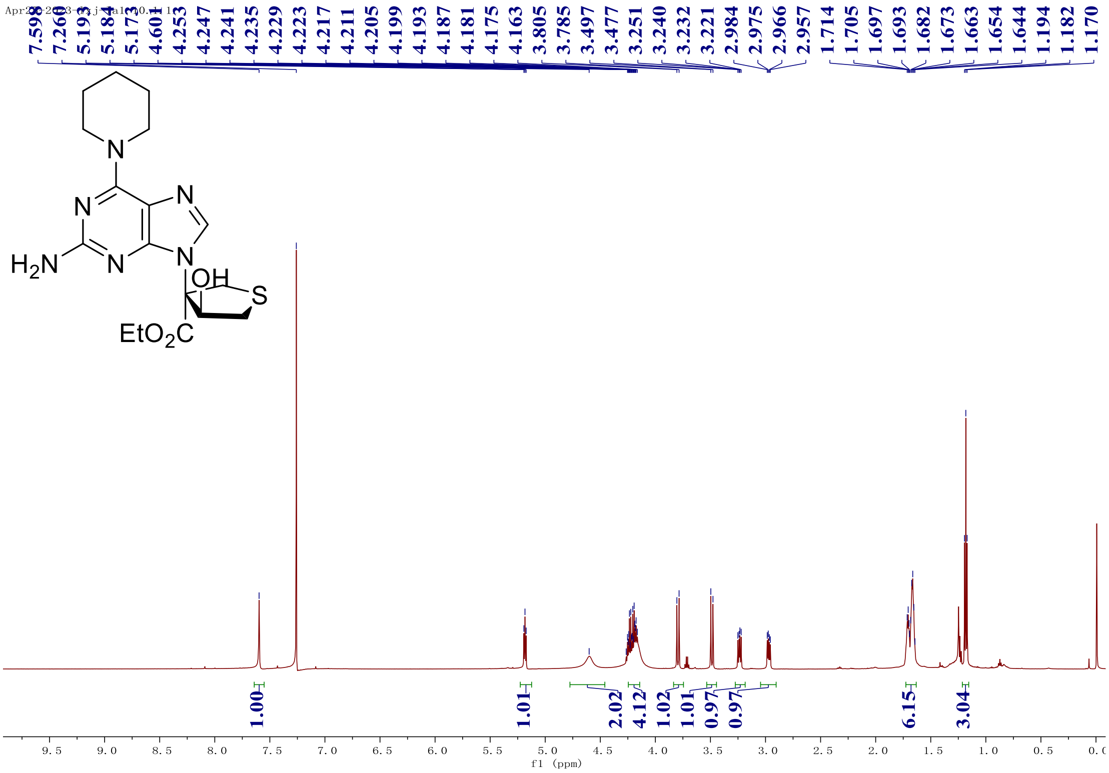

^{1}H NMR spectrum

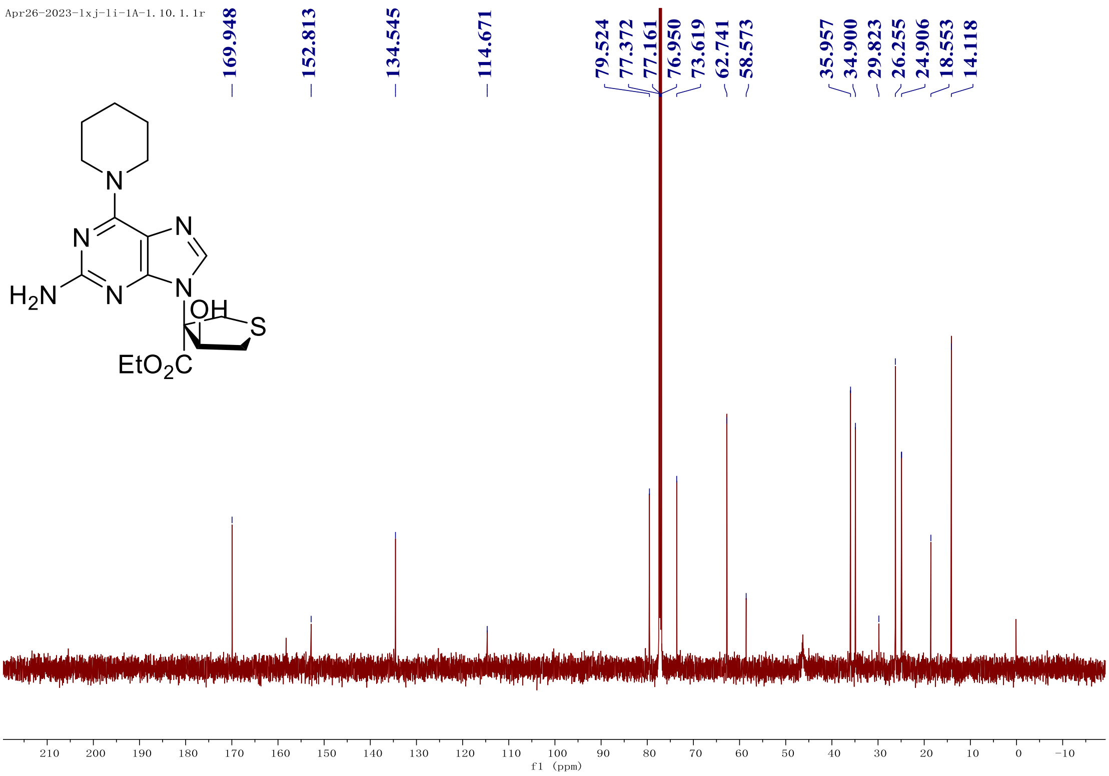

^{13}C NMR spectrum

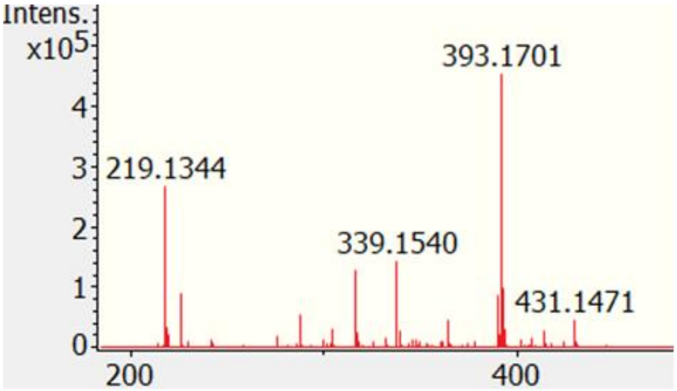

HR-ESIMS spectrum

(±)Ethyl-3-(2-amino-6-(piperidin-1-yl)-9*H*-purin-9-yl)-4-hydroxytetrahydrothiophene-3-carboxylate (**32b**)

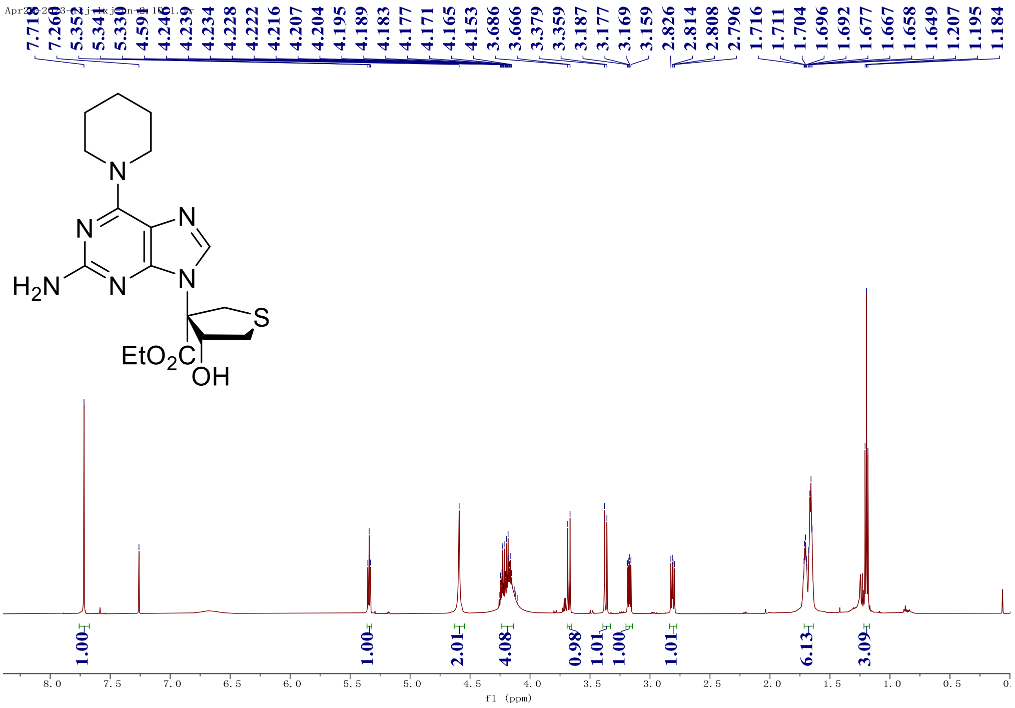

^{1}H NMR spectrum

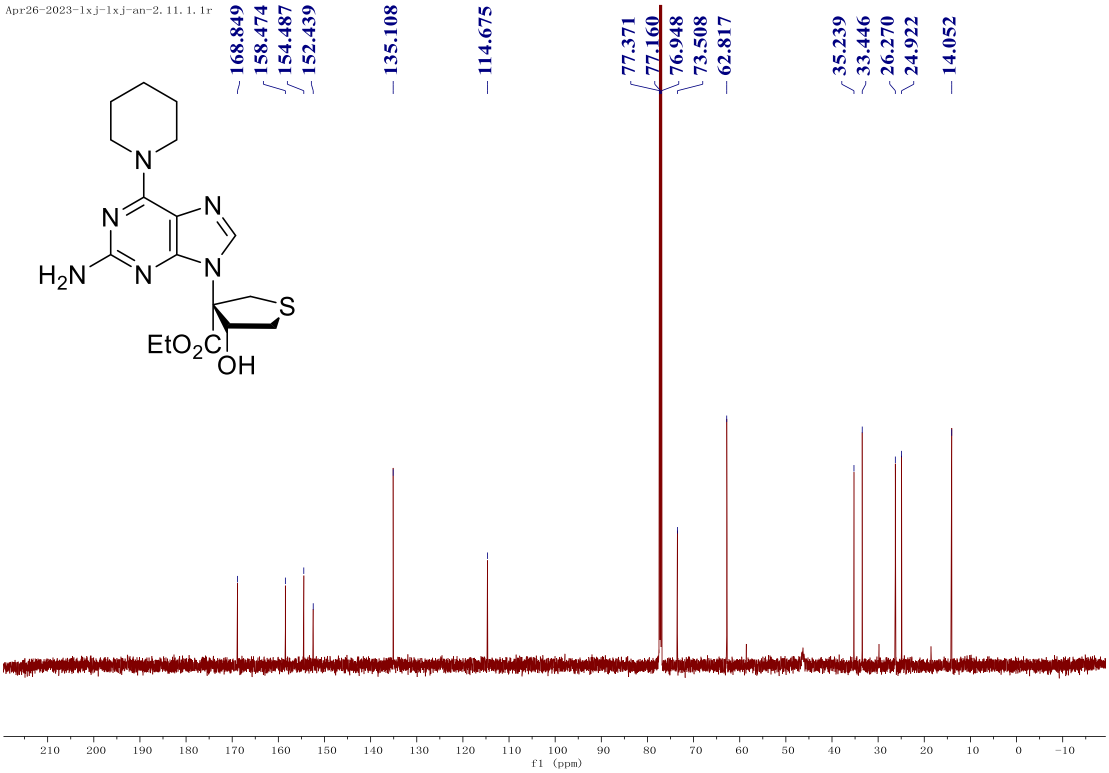

^{13}C NMR spectrum

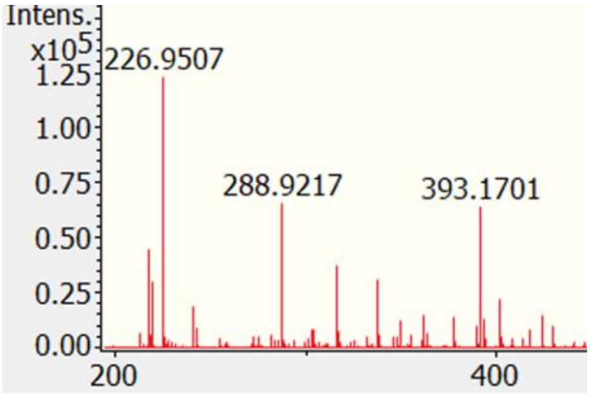

HR-ESIMS spectrum

(±)Ethyl-3-(2-fluoro-6-(piperidin-1-yl)-9*H*-purin-9-yl)-4-hydroxytetrahydrothiophene-3-carboxylate (**33a**)

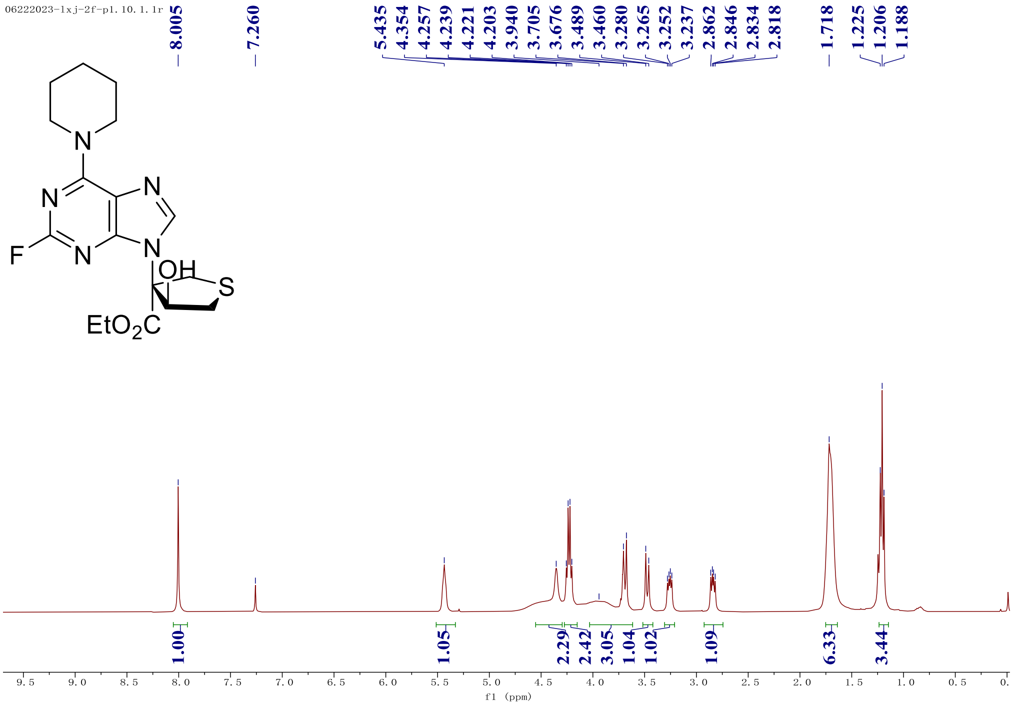

^{1}H NMR spectrum

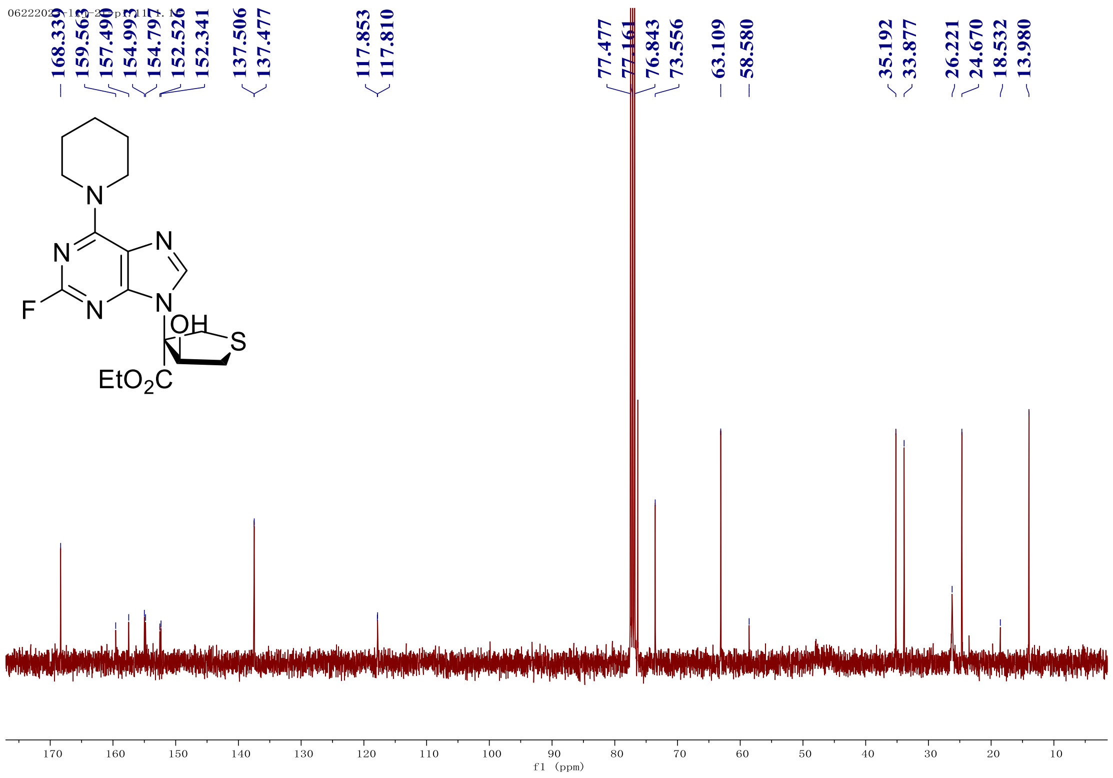

^{13}C NMR spectrum

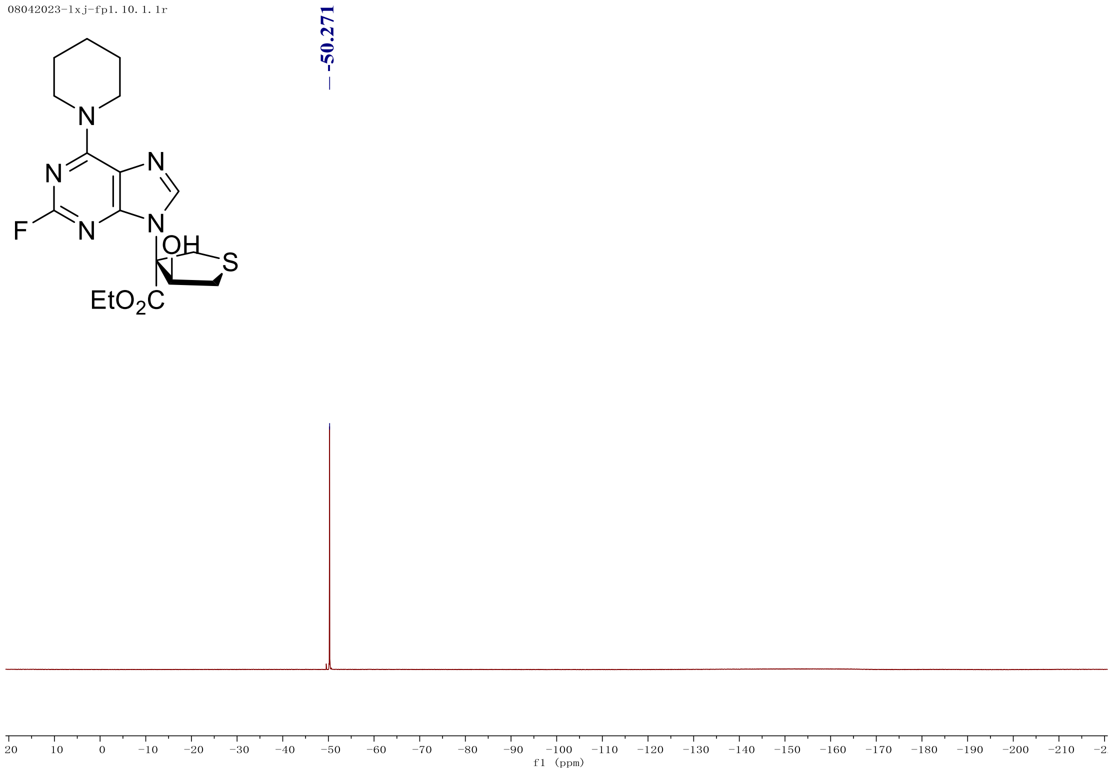

^{19}F NMR spectrum

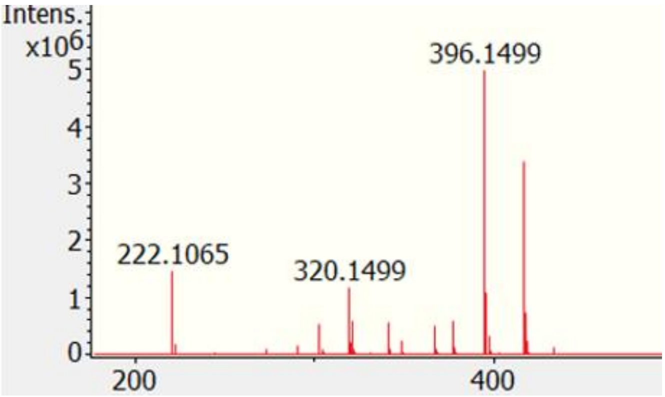

HR-ESIMS spectrum

(±)Ethyl-3-(2-fluoro-6-(piperidin-1-yl)-9*H*-purin-9-yl)-4-hydroxytetrahydrothiophene-3-carboxylate (**33b**)

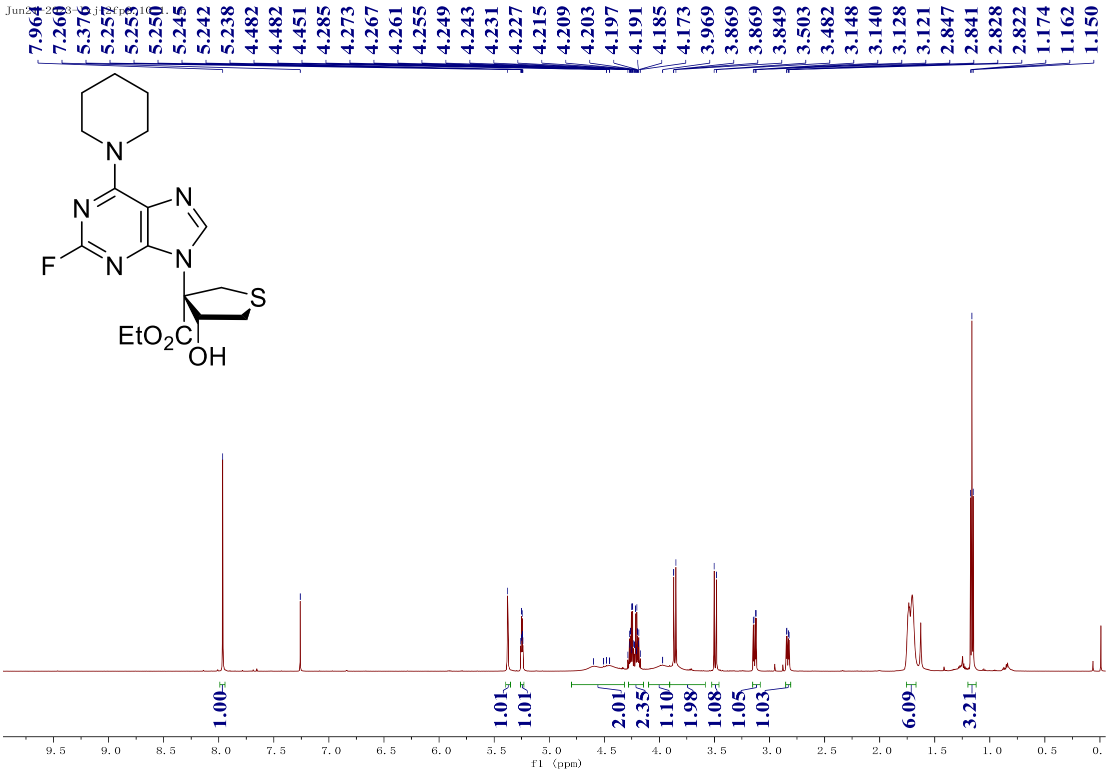

^{1}H NMR spectrum

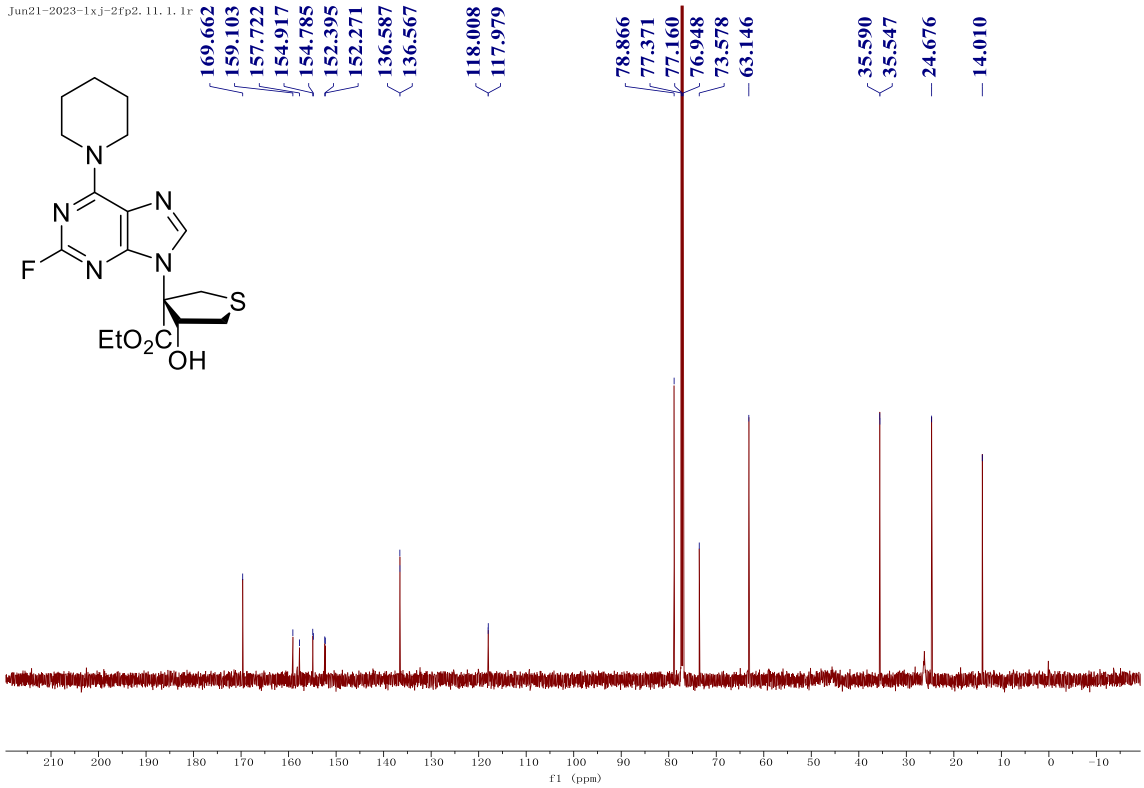

^{13}C NMR spectrum

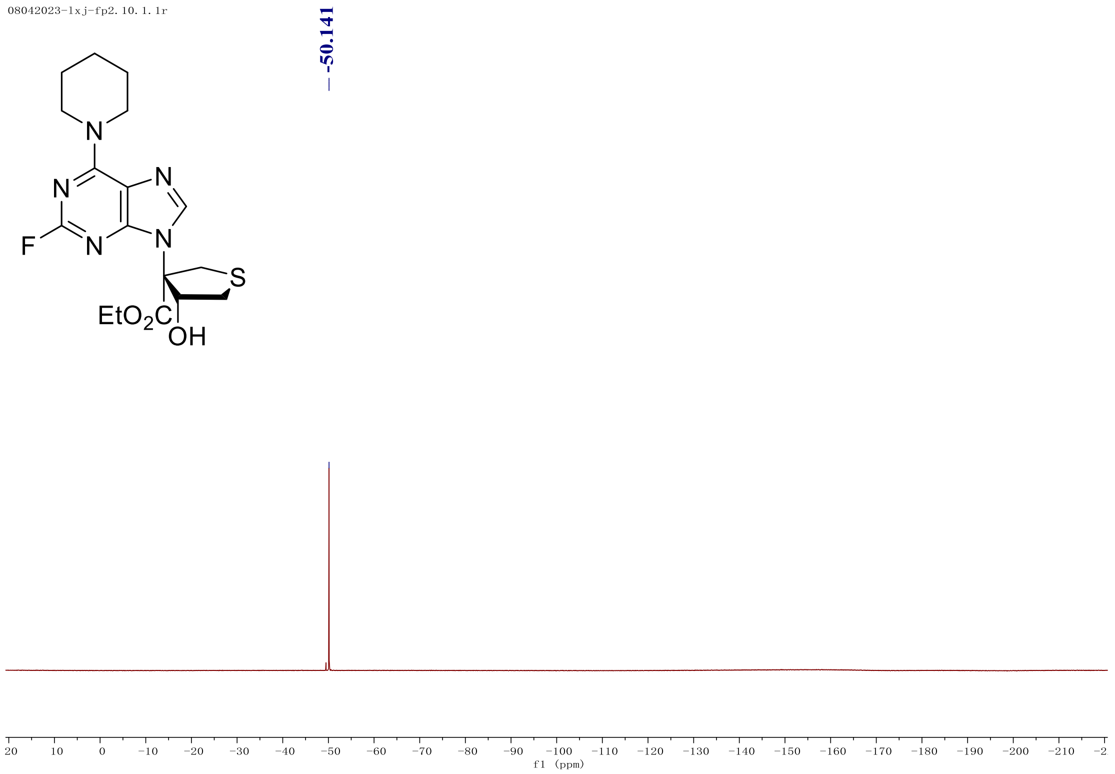

^{19}F NMR spectrum

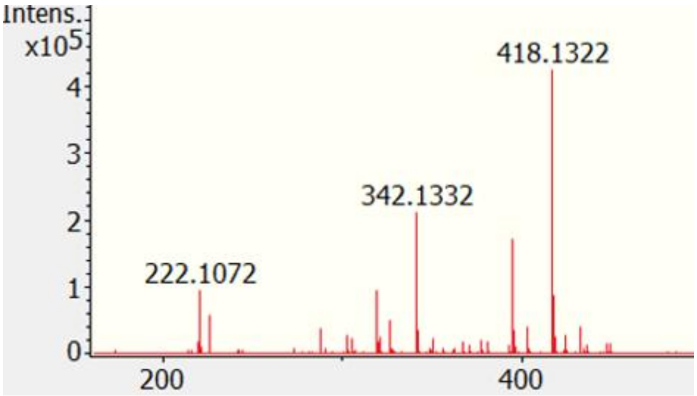

HR-ESIMS spectrum

(±)Ethyl-3-(2-chloro-6-(piperidin-1-yl)-9*H*-purin-9-yl)-4-hydroxytetrahydrothiophene-3-carboxylate (**34a**)

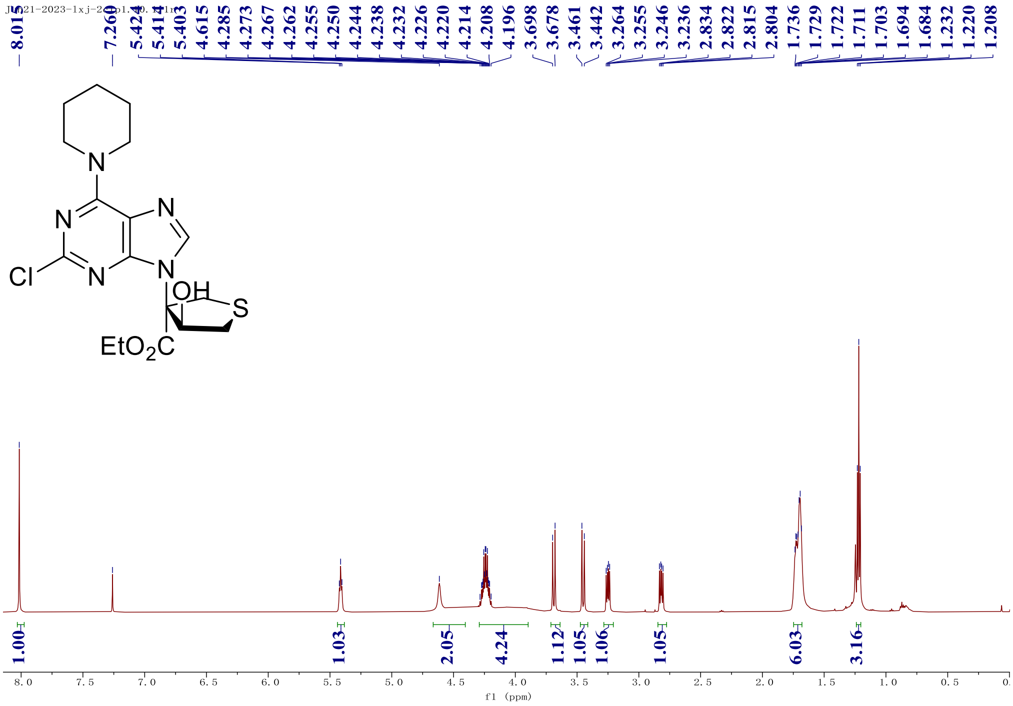

^{1}H NMR spectrum

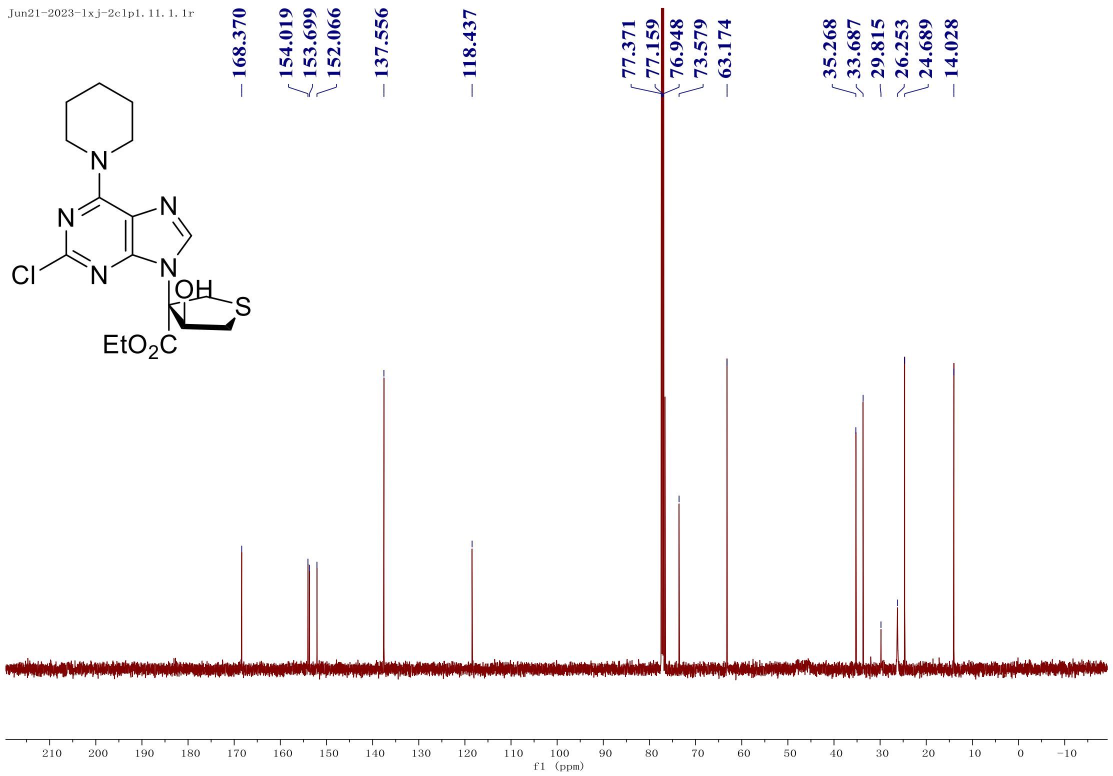

^{13}C NMR spectrum

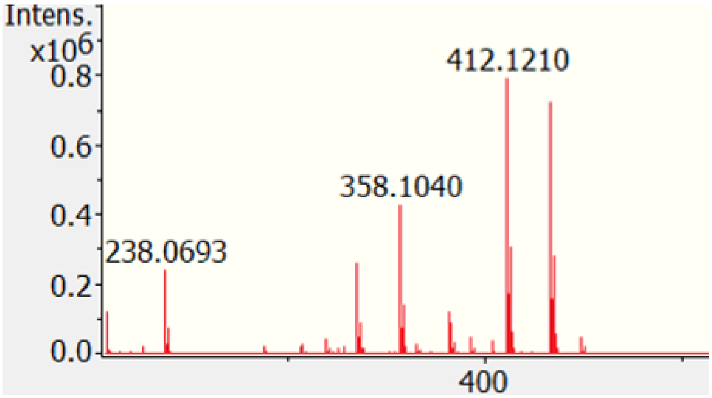

HR-ESIMS spectrum

(±)Ethyl-3-(2-chloro-6-(piperidin-1-yl)-9*H*-purin-9-yl)-4-hydroxytetrahydrothiophene-3-carboxylate (**34b**)

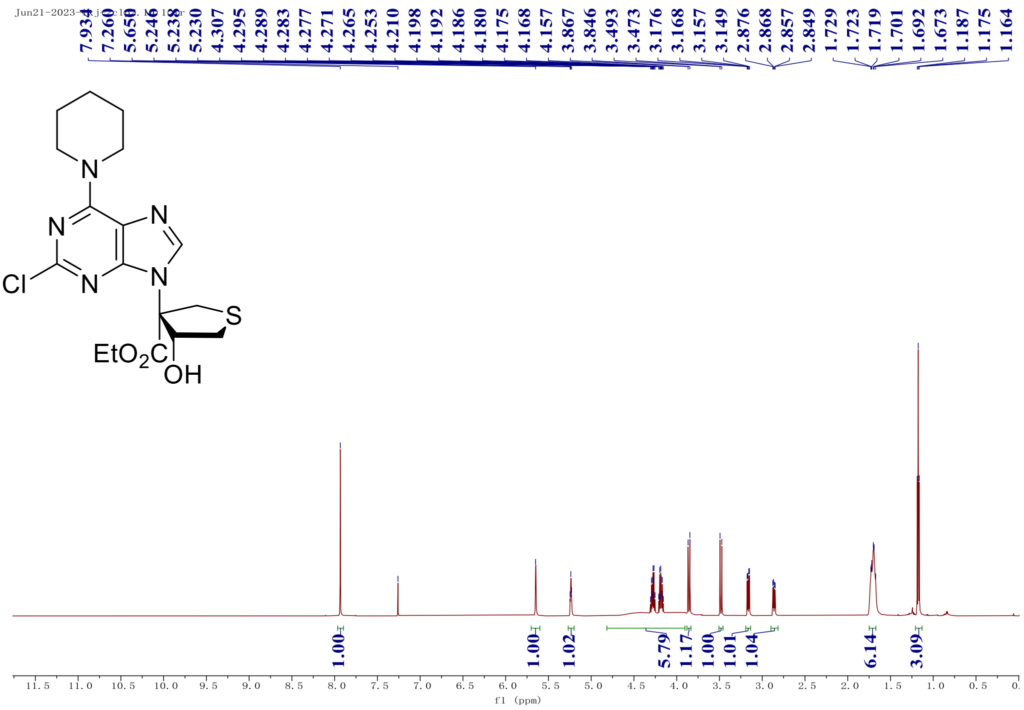

^{1}H NMR spectrum

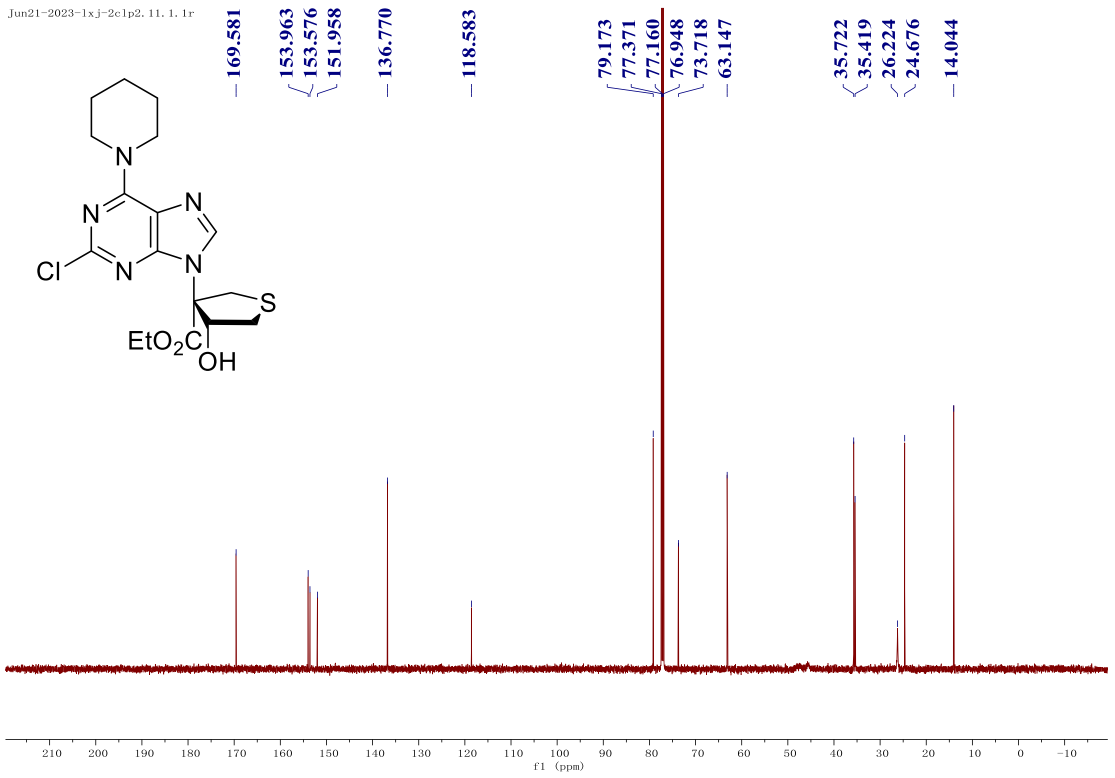

^{13}C NMR spectrum

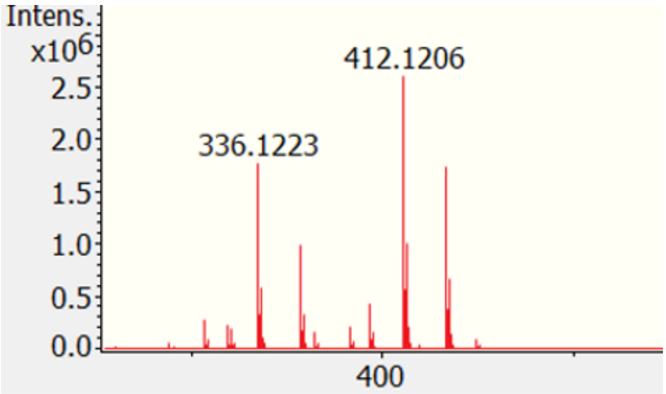

HR-ESIMS spectrum

(±)Ethyl-3-(2-amino-6-(propylthio)-9*H*-purin-9-yl)-4-hydroxytetrahydrothiophene-3-carboxylate (**35a**)

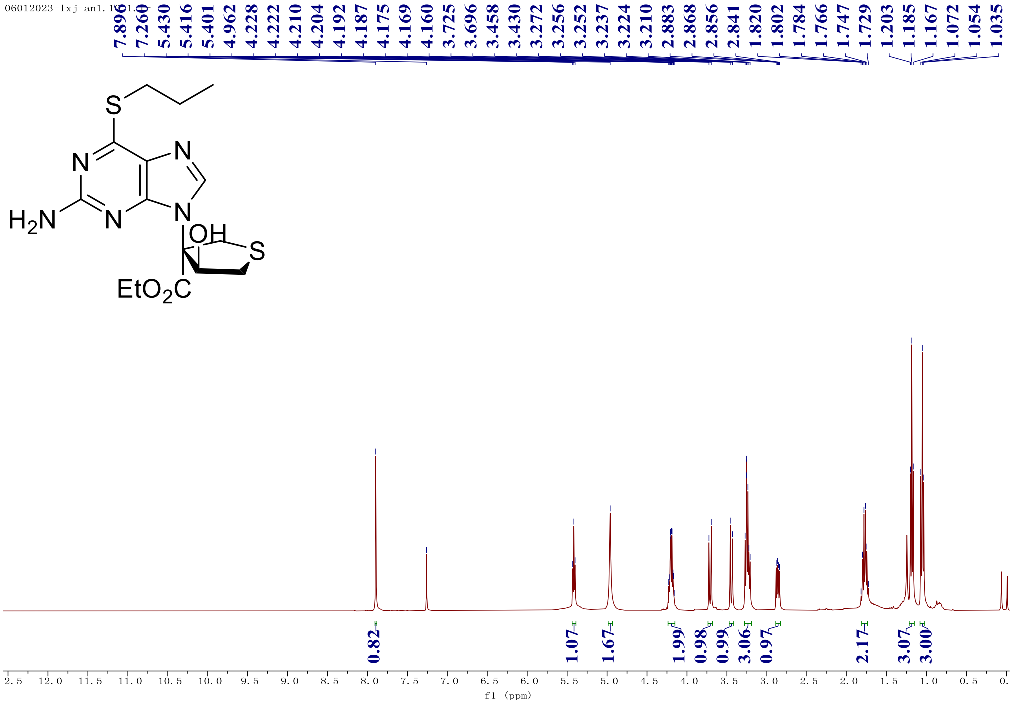

^{1}H NMR spectrum

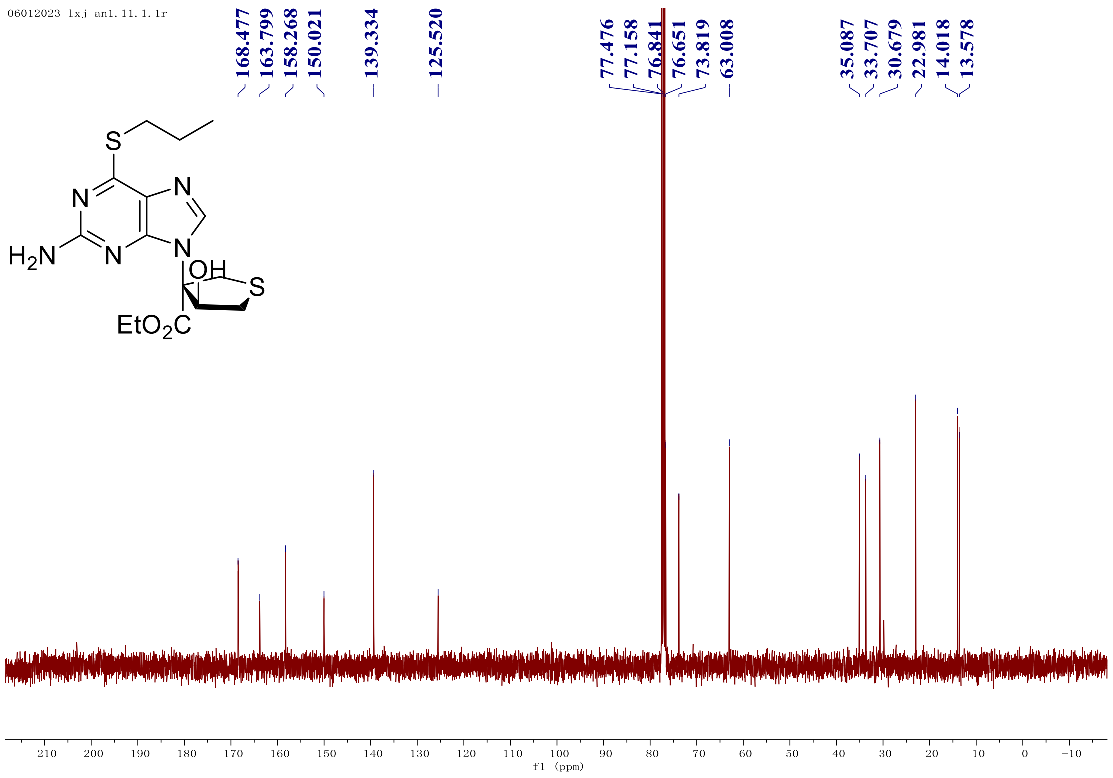

^{13}C NMR spectrum

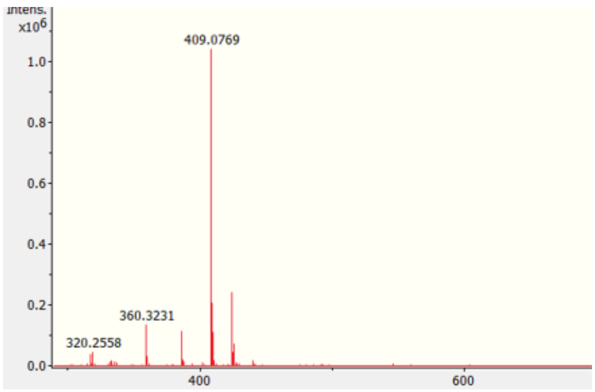

HR-ESIMS spectrum

(±)Ethyl-3-(2-amino-6-(propylthio)-9*H*-purin-9-yl)-4-hydroxytetrahydrothiophene-3-carboxylate (**35b**)

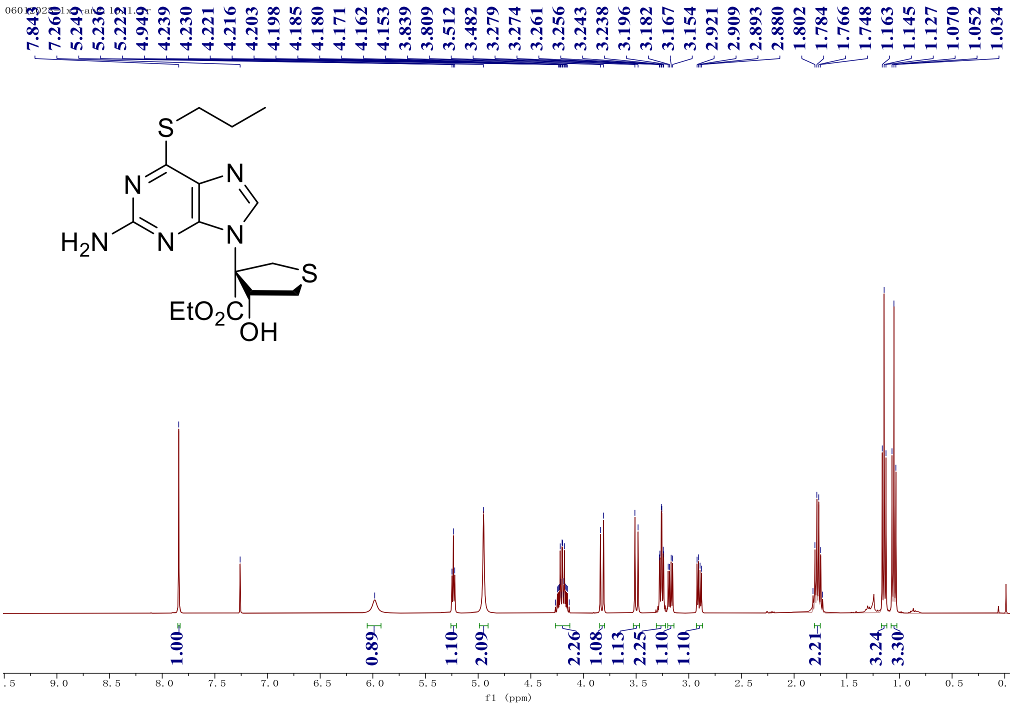

^{1}H NMR spectrum

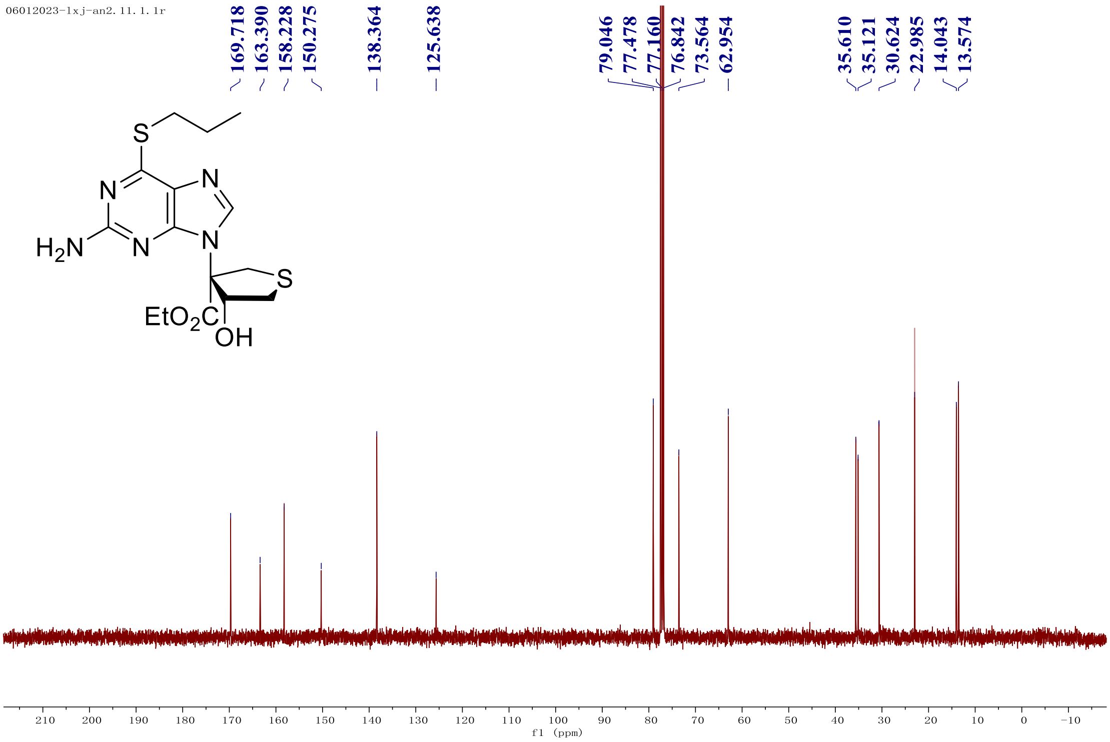

^{13}C NMR spectrum

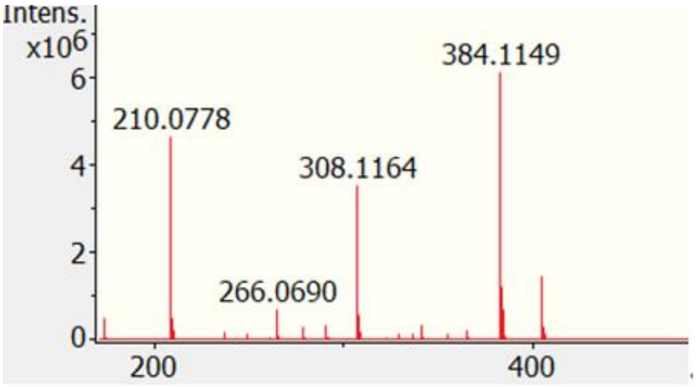

HR-ESIMS spectrum

(±)Ethyl-3-(2-fluoro-6-(propylthio)-9*H*-purin-9-yl)-4-hydroxytetrahydrothiophene-3-carboxylate (**36a**)

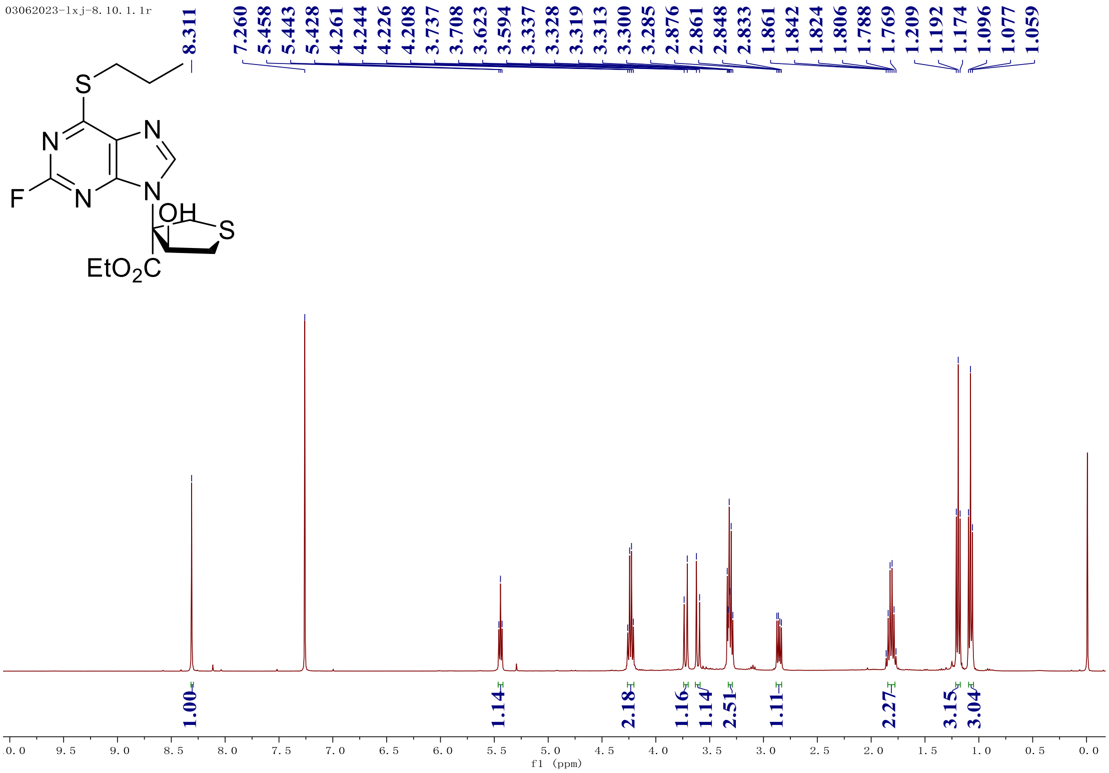

^{1}H NMR spectrum

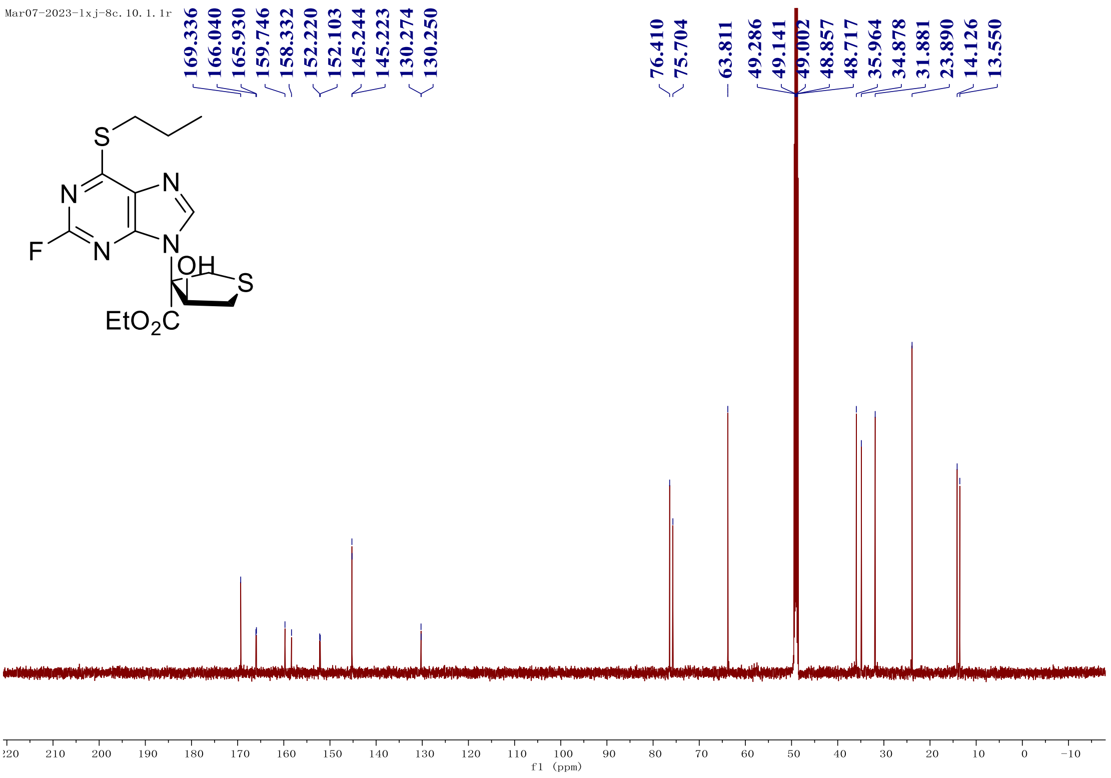

^{13}C NMR spectrum

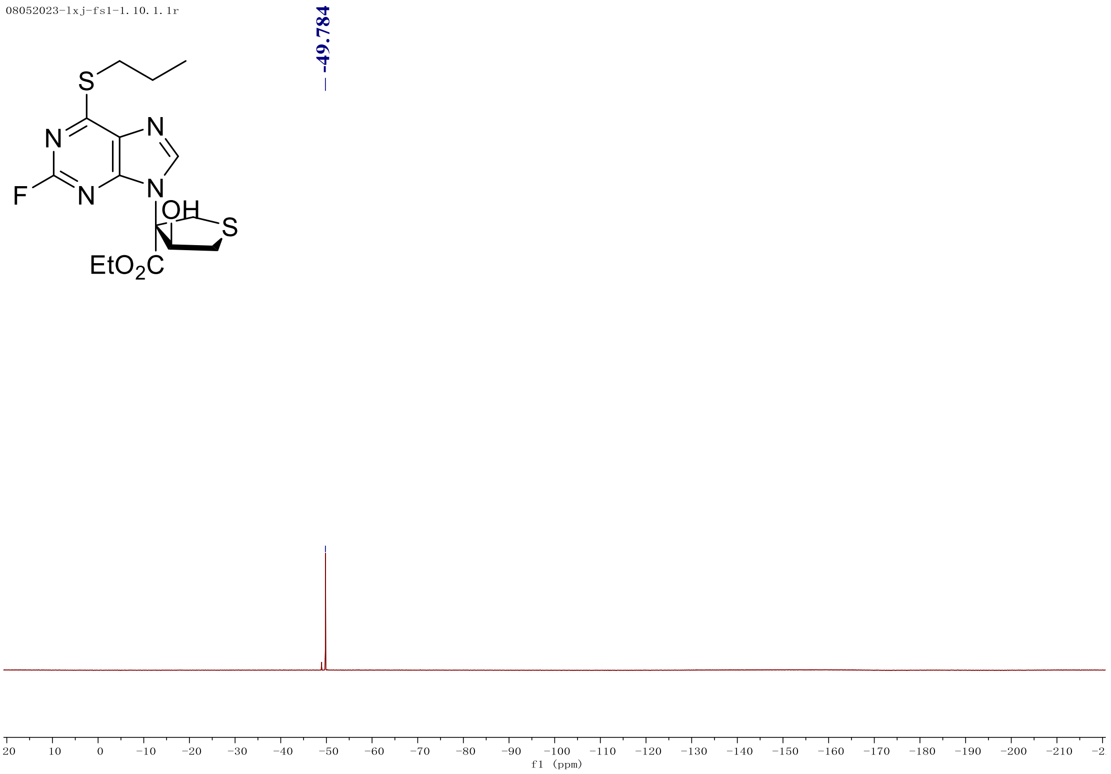

^{19}F NMR spectrum

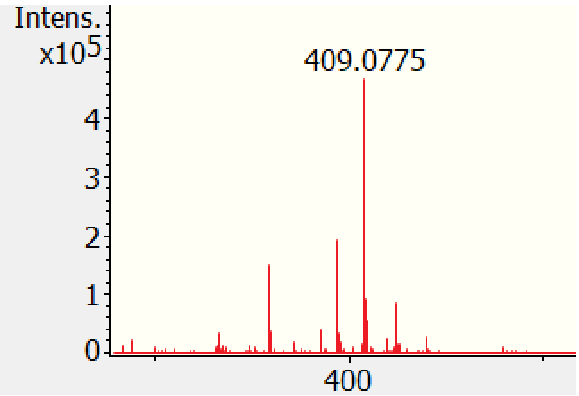

HR-ESIMS spectrum

(±)Ethyl-3-(2-fluoro-6-(propylthio)-9*H*-purin-9-yl)-4-hydroxytetrahydrothiophene-3-carboxylate (**36b**)

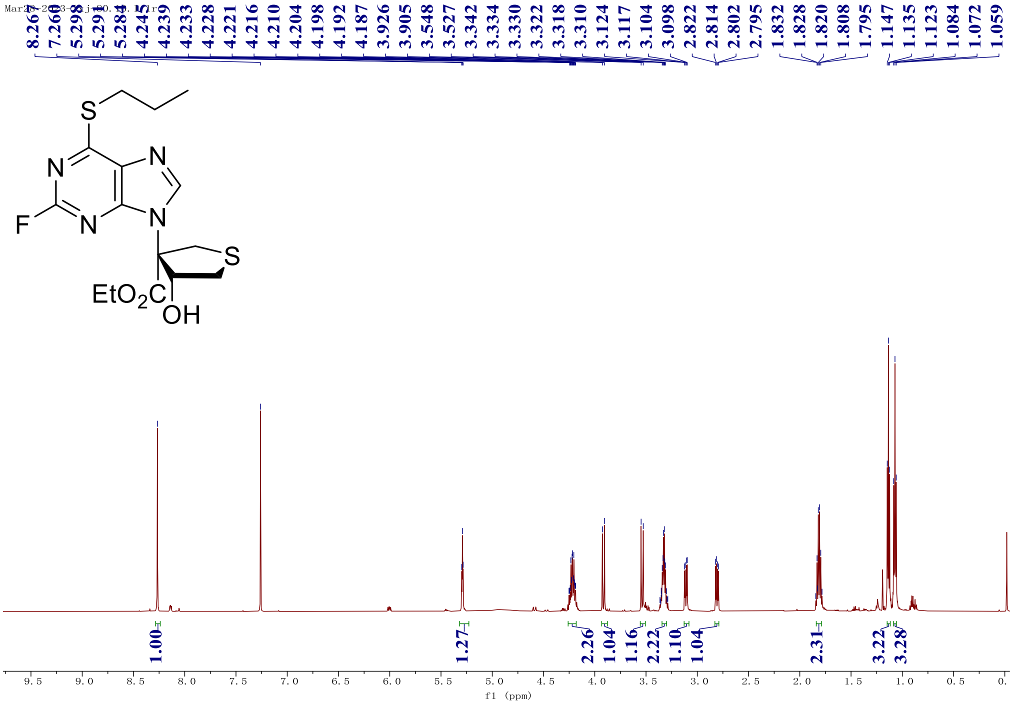

^{1}H NMR spectrum

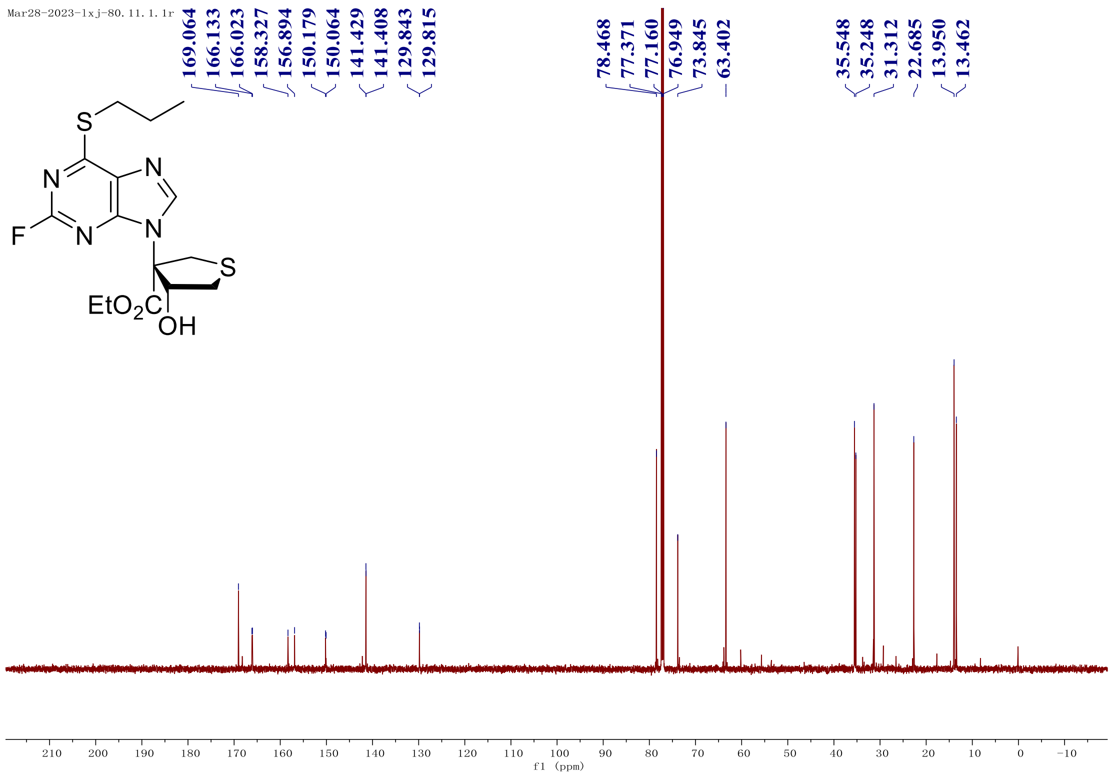

^{13}C NMR spectrum

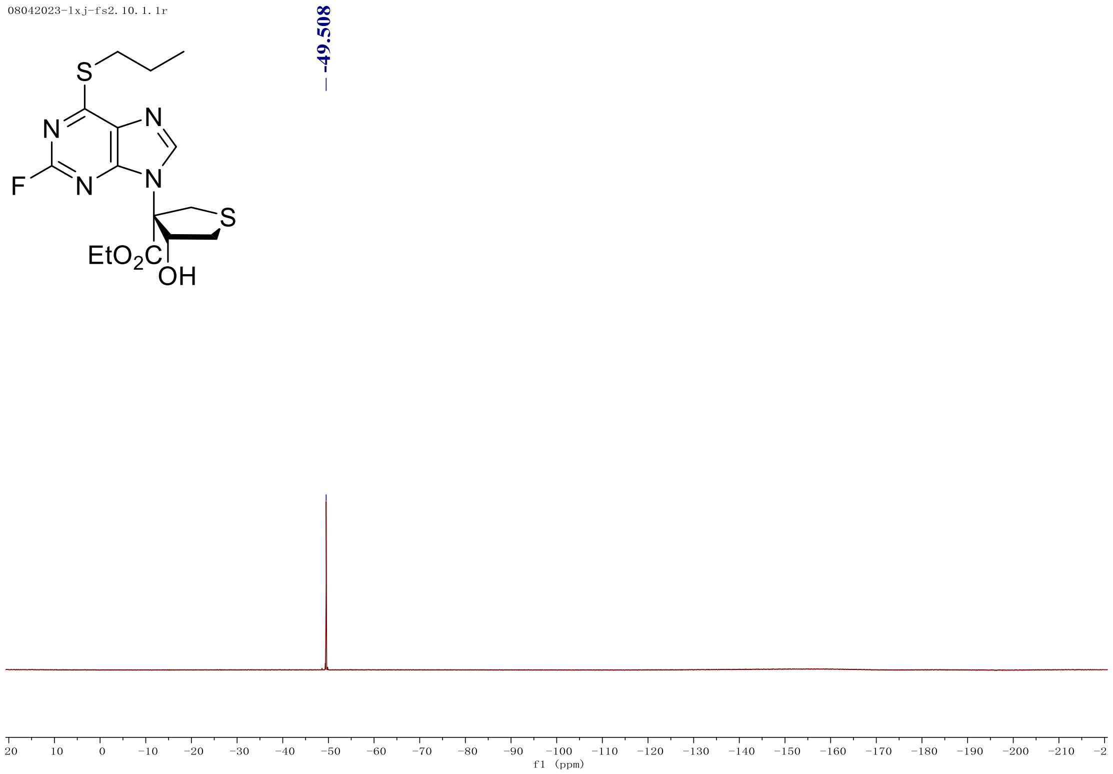

^{19}F NMR spectrum

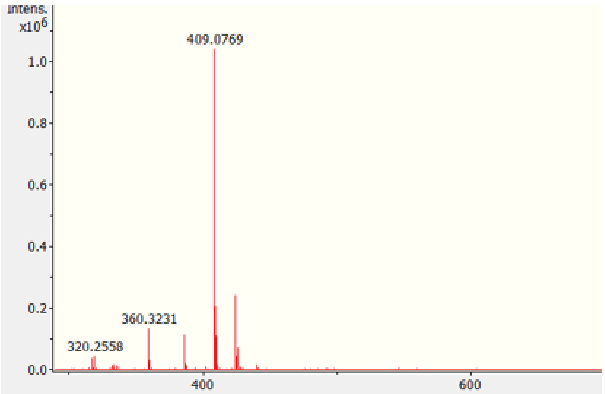

HR-ESIMS spectrum

(±)Ethyl-3-(2-chloro-6-(propylthio)-9*H*-purin-9-yl)-4-hydroxytetrahydrothiophene-3-carboxylate (**37a**)

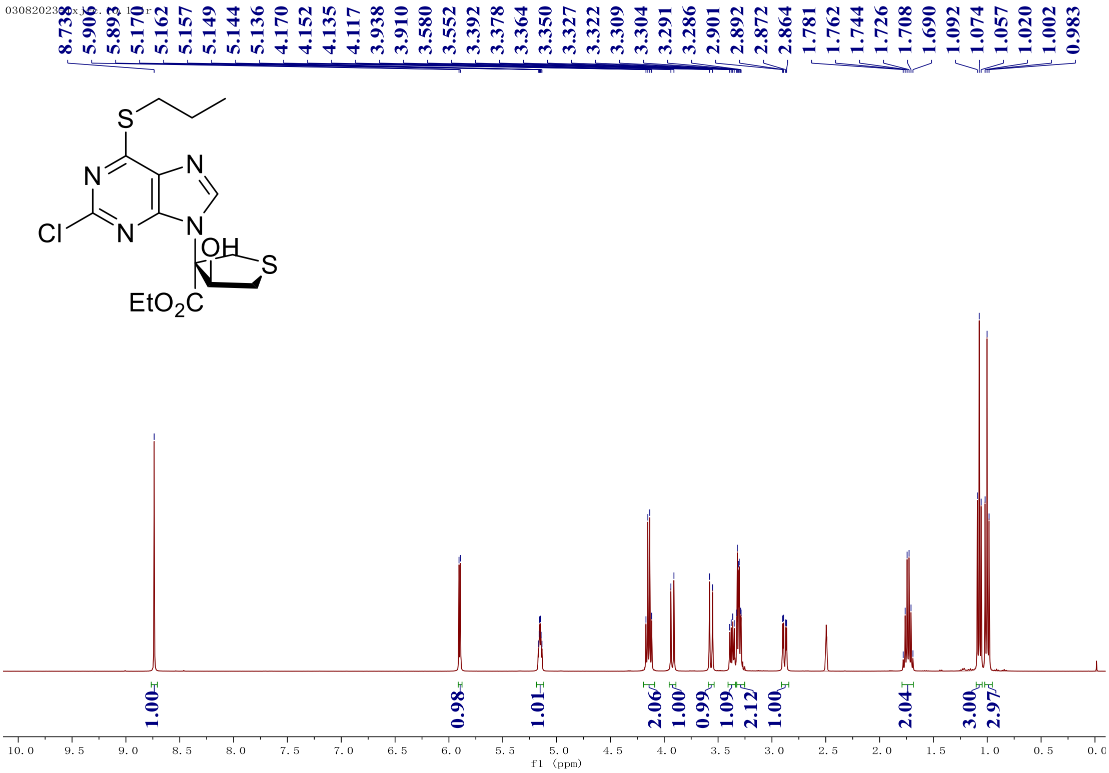

^{1}H NMR spectrum

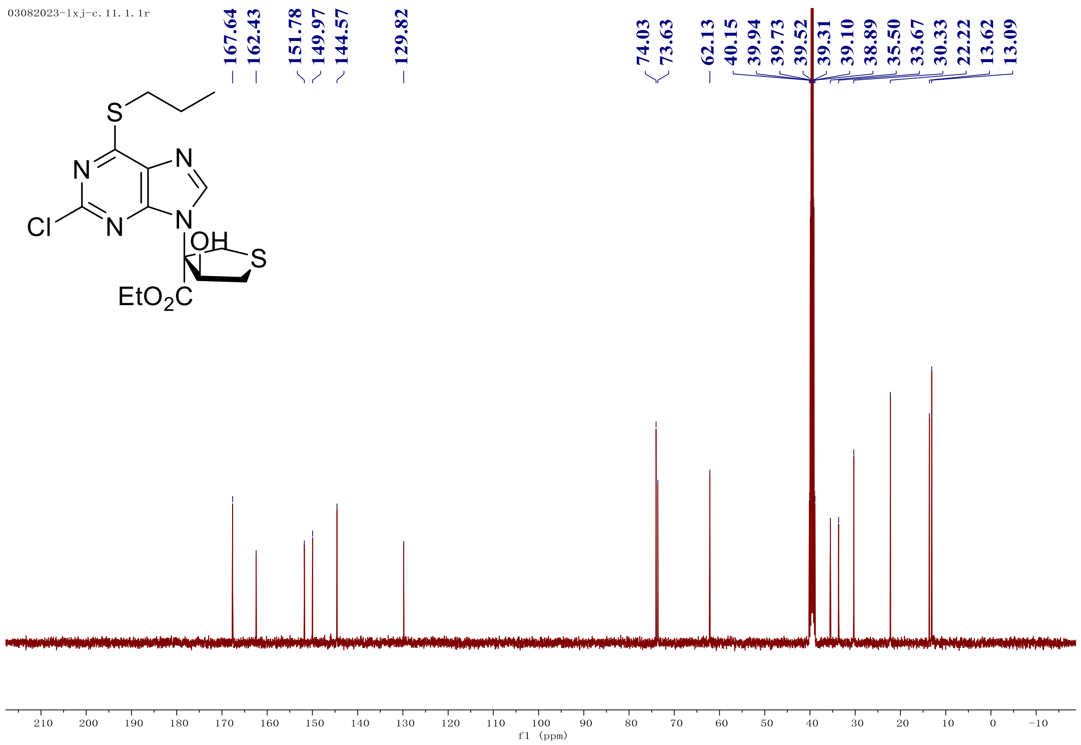

^{13}C NMR spectrum

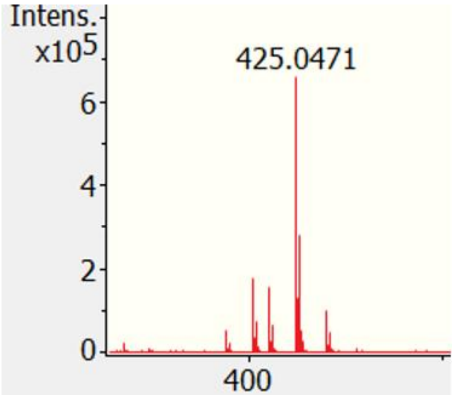

HR-ESIMS spectrum

(±)Ethyl-3-(2-chloro-6-(propylthio)-9*H*-purin-9-yl)-4-hydroxytetrahydrothiophene-3-carboxylate (**37b**)

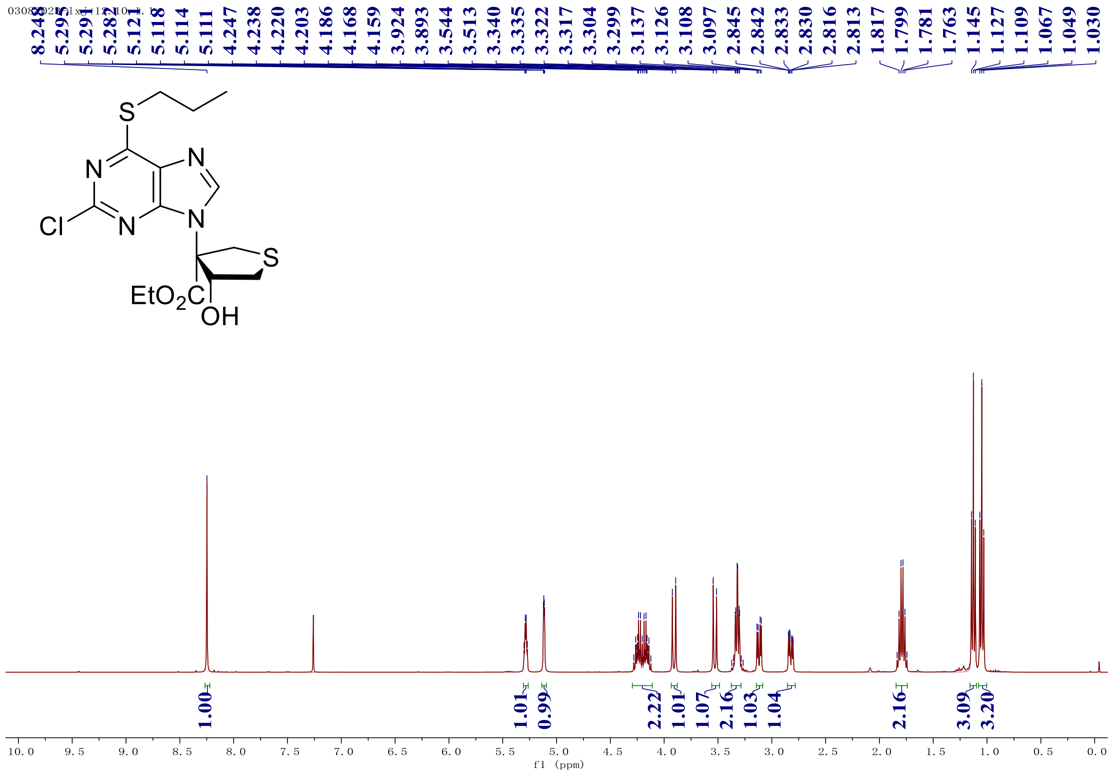

^{1}H NMR spectrum

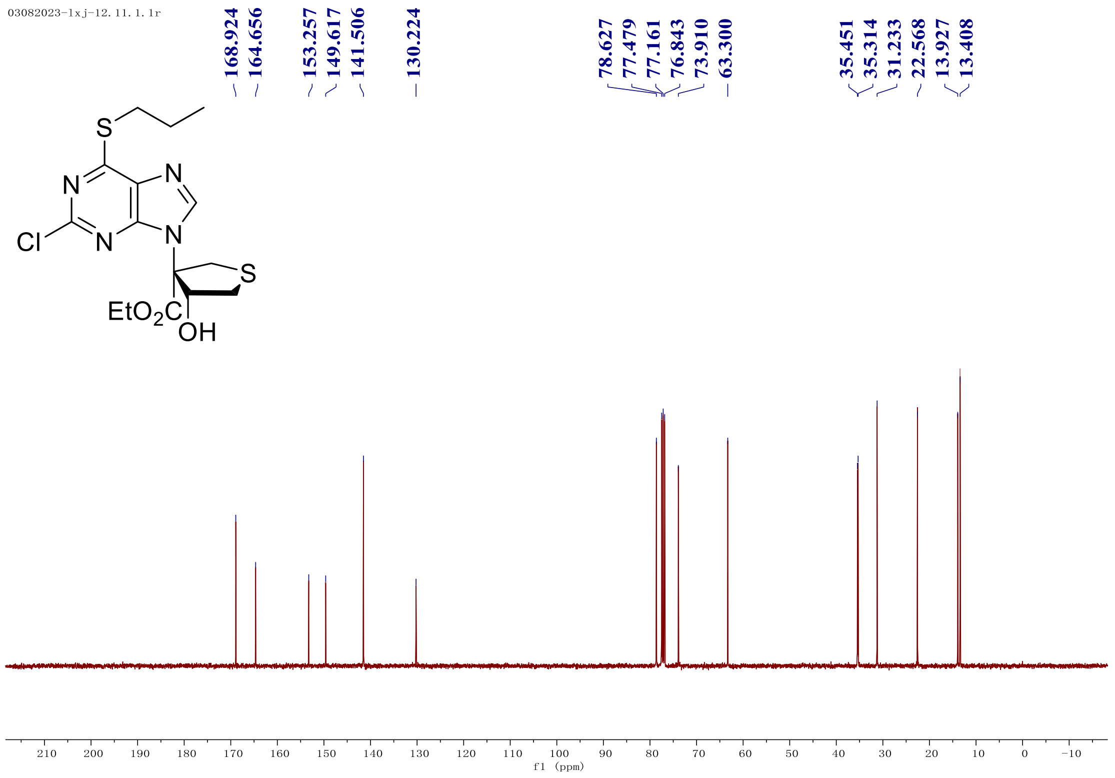

^{13}C NMR spectrum

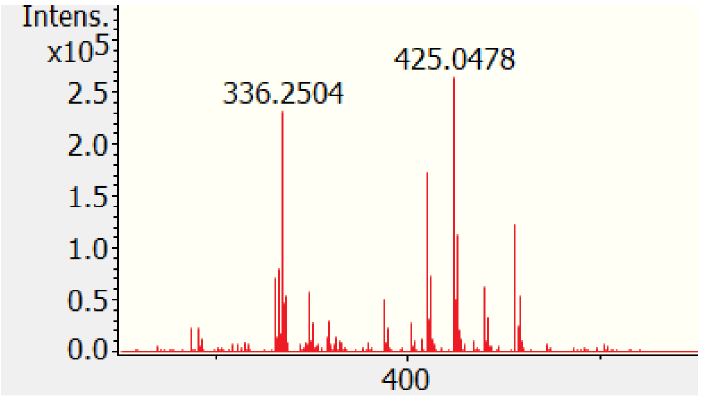

HR-ESIMS spectrum

(±)Ethyl-3-(2-amino-6-chloro-9*H*-purin-9-yl)-4-hydroxytetrahydrothiophene-3-carboxylate (**38a**)

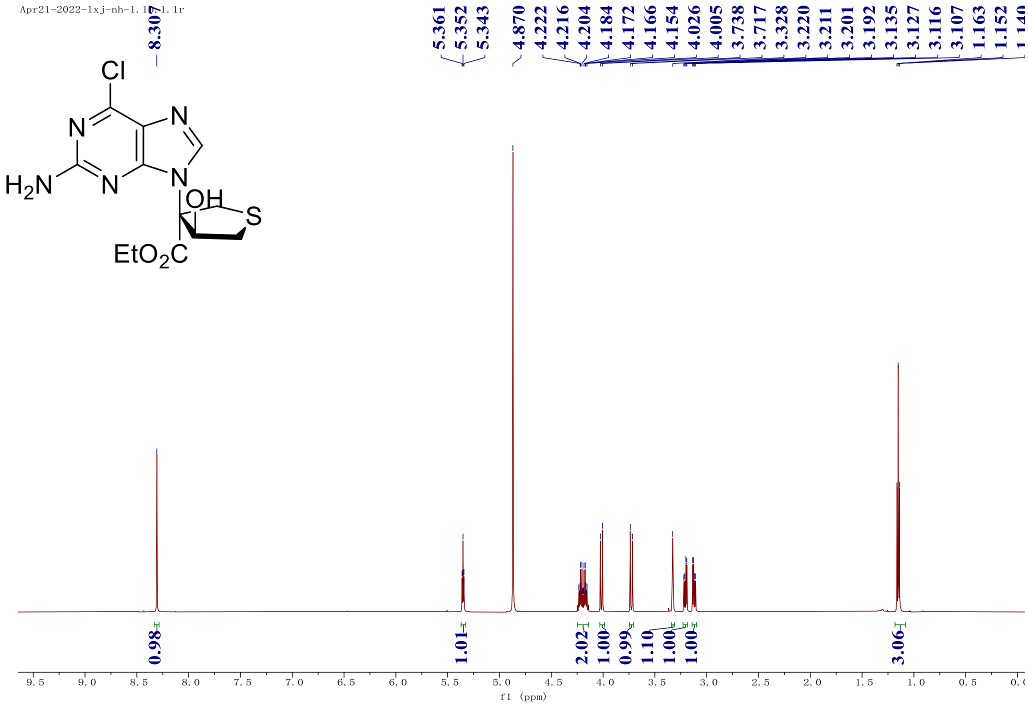

^{1}H NMR spectrum

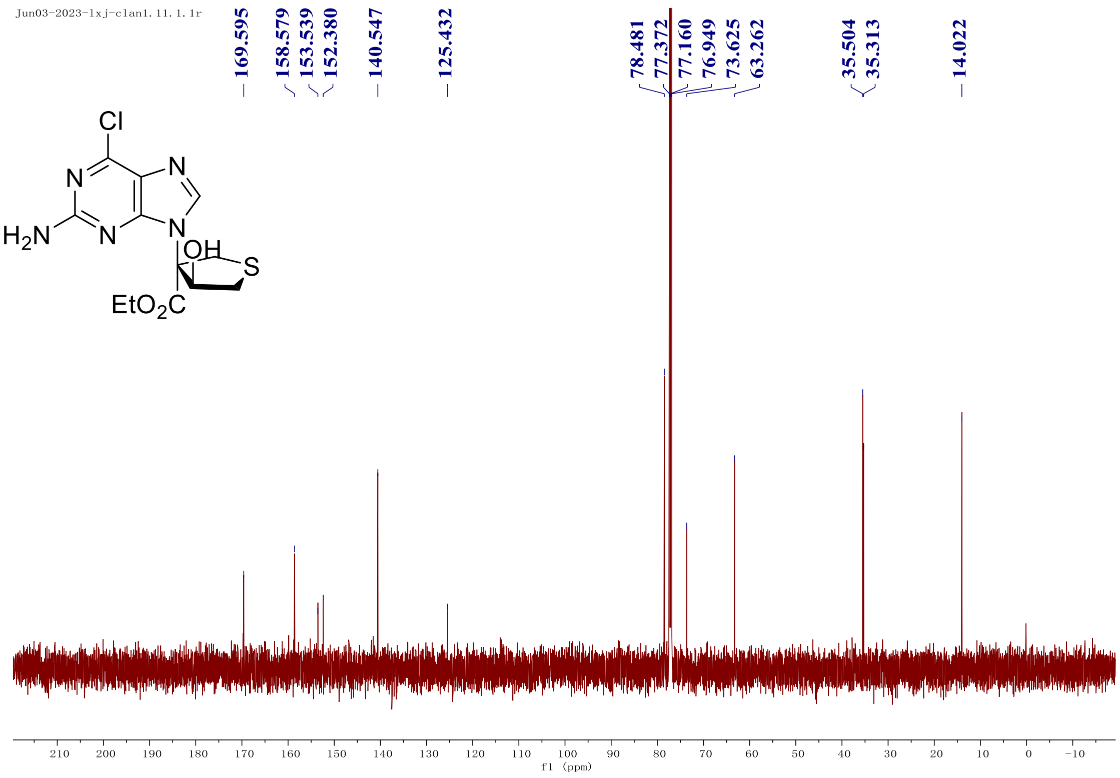

^{13}C NMR spectrum

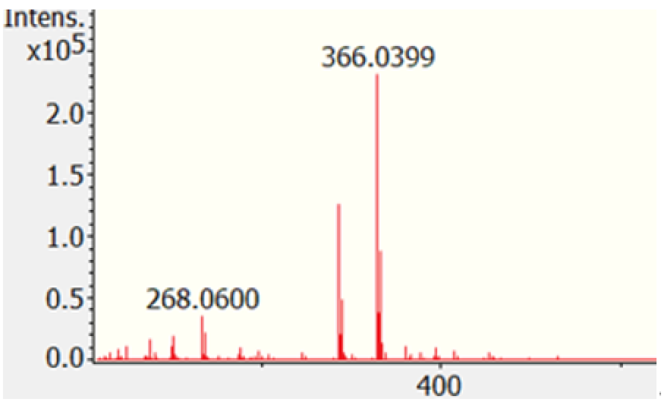

HR-ESIMS spectrum

(±)Ethyl-3-(2-amino-6-chloro-9*H*-purin-9-yl)-4-hydroxytetrahydrothiophene-3-carboxylate (**38b**)

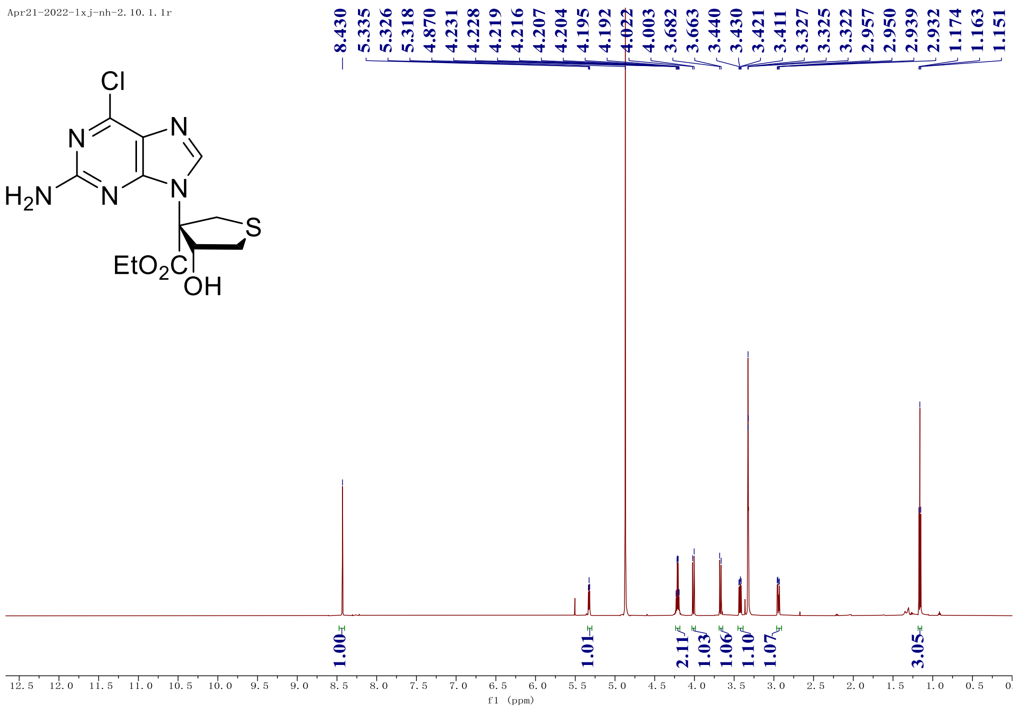

^{1}H NMR spectrum

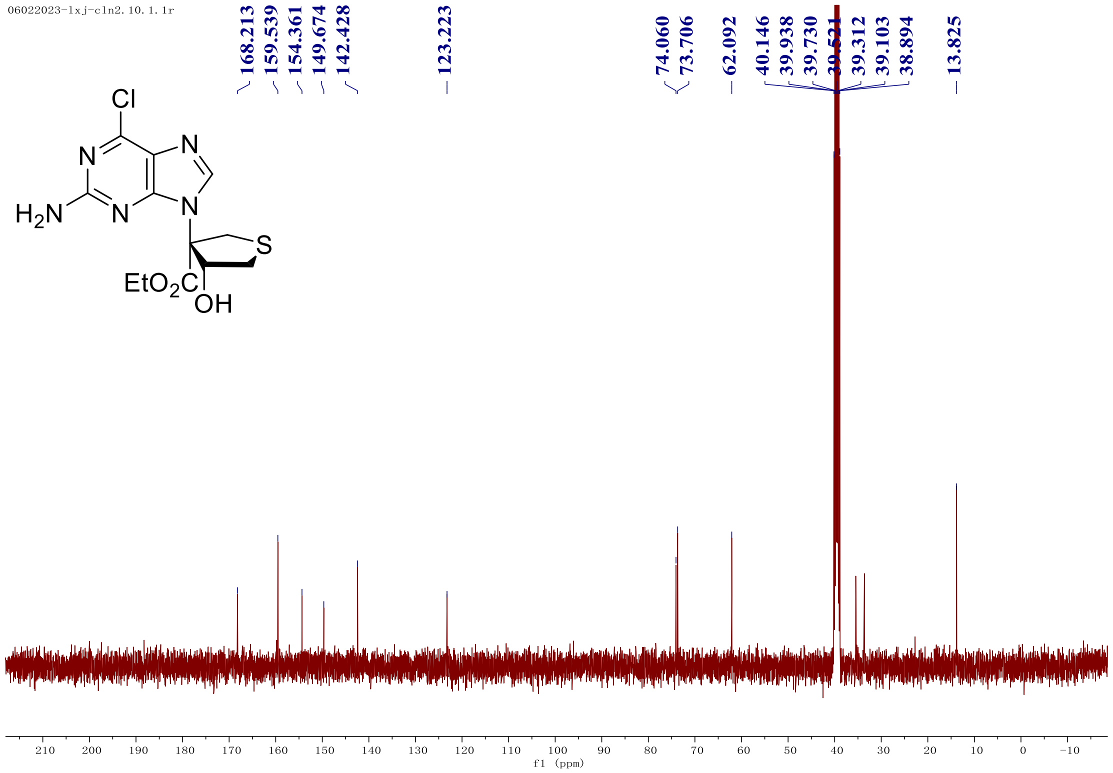

^{13}C NMR spectrum

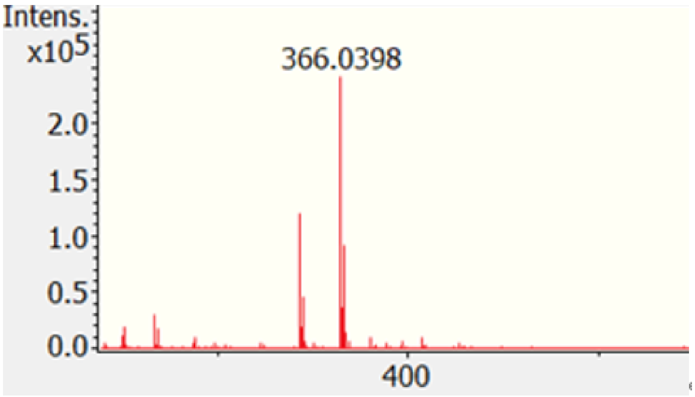

HR-ESIMS spectrum

±)Ethy-3-(6-chloro-2-fluoro-9*H*-purin-9-yl)-4-hydroxytetrahydrothiophene-3-carboxylate (**39a**)

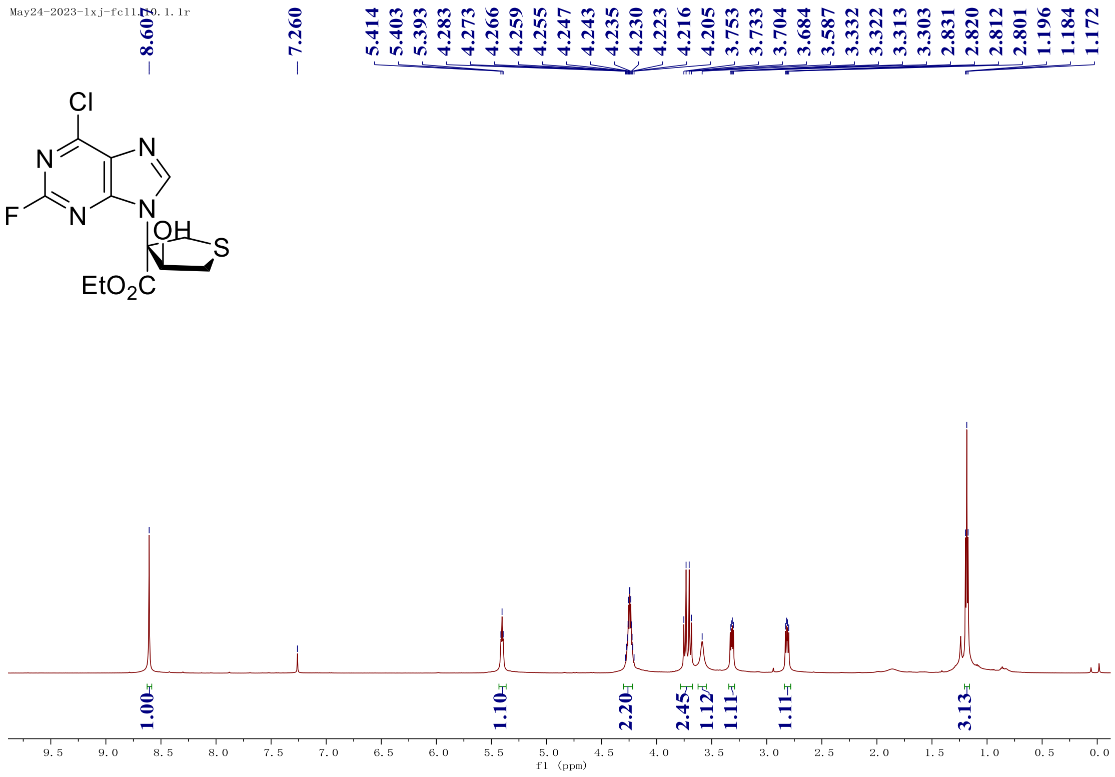

^{1}H NMR spectrum

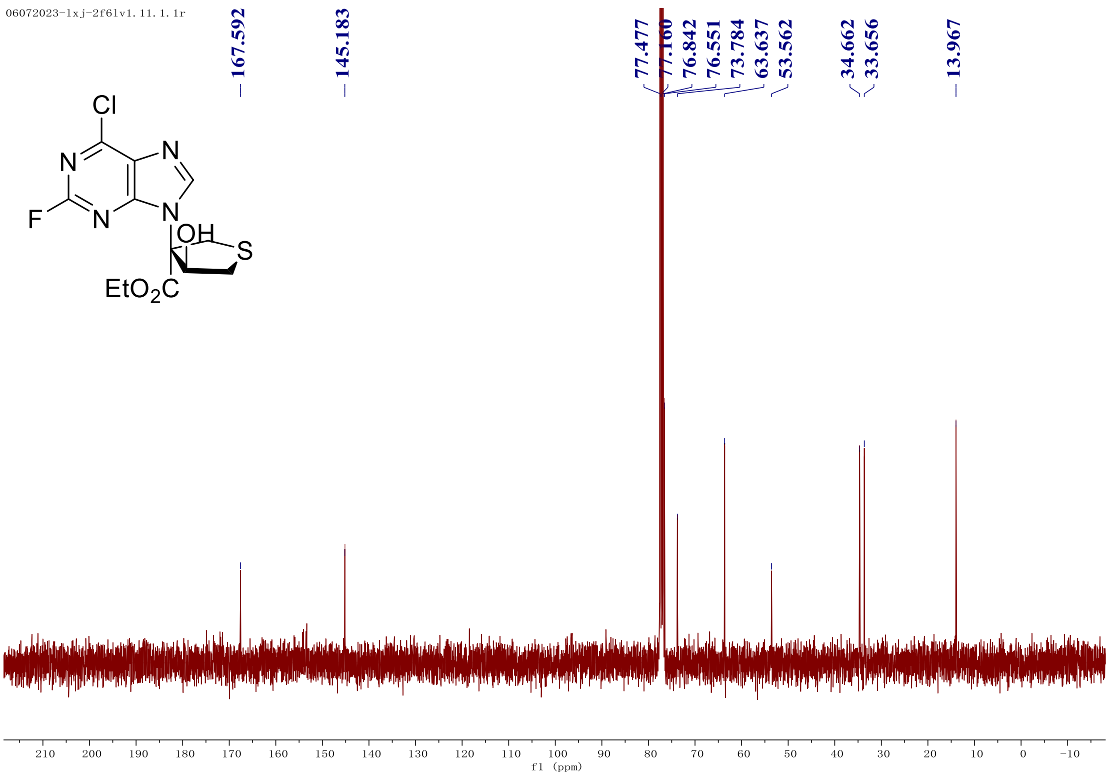

^{13}C NMR spectrum

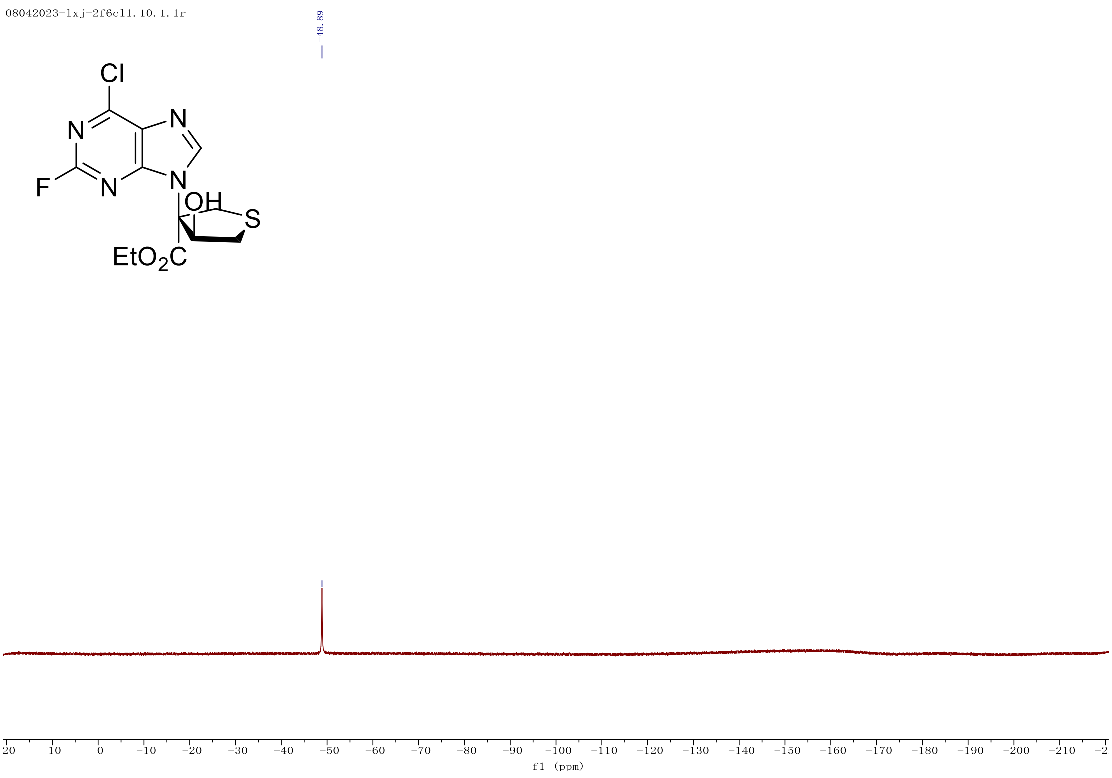

^{19}F NMR spectrum

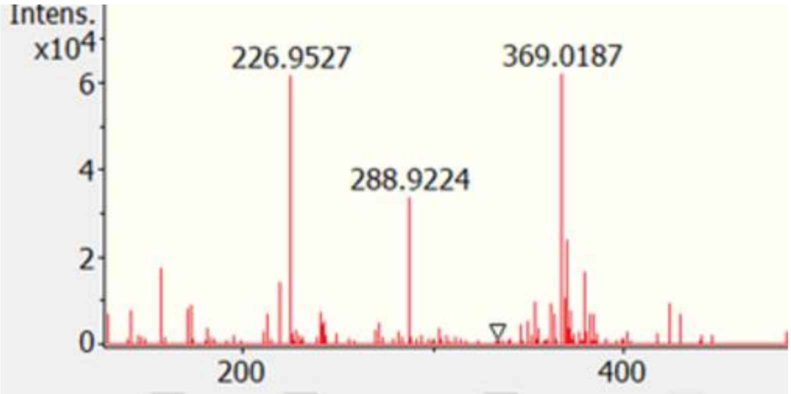

HR-ESIMS spectrum

(±)Ethy-3-(6-chloro-2-fluoro-9*H*-purin-9-yl)-4-hydroxytetrahydrothiophene-3-carboxylate(**39b**)

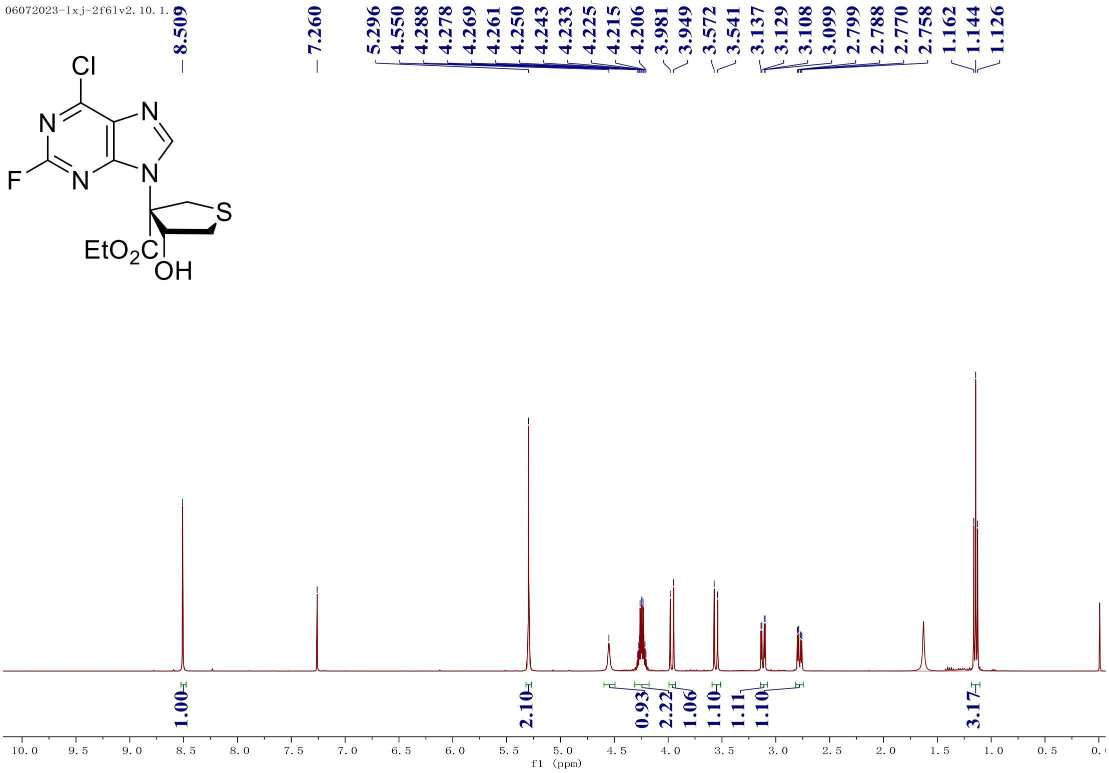

^{1}H NMR spectrum

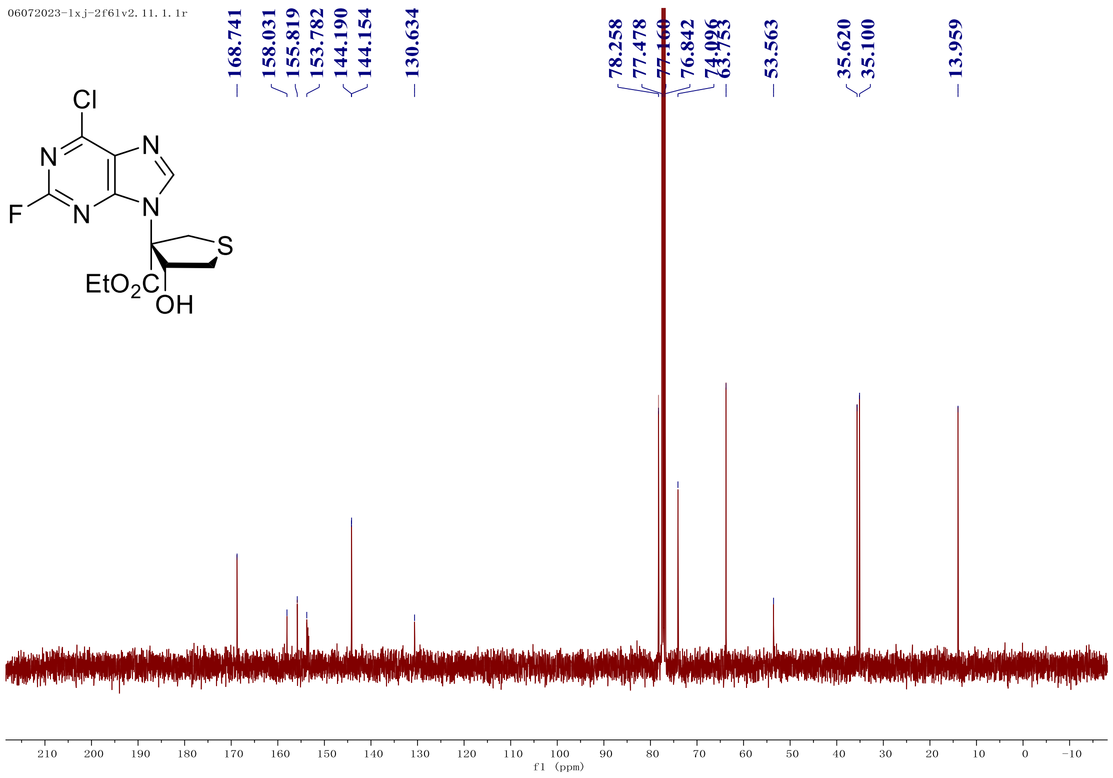

^{13}C NMR spectrum

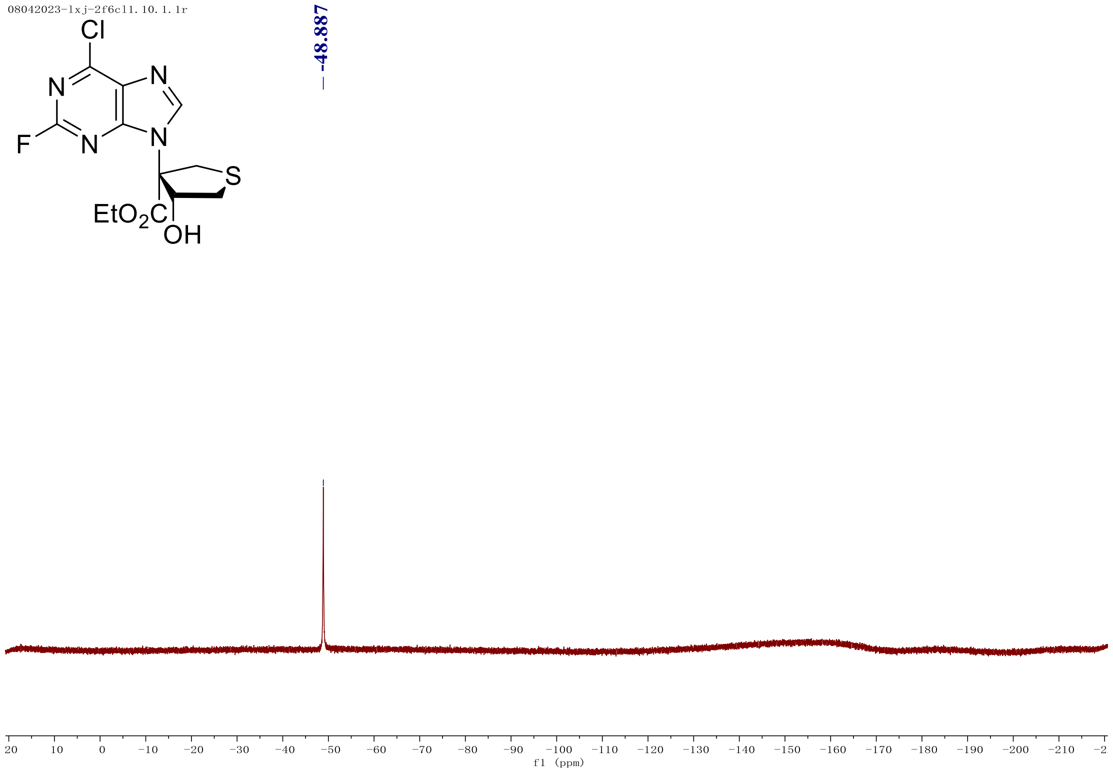

^{19}F NMR spectrum

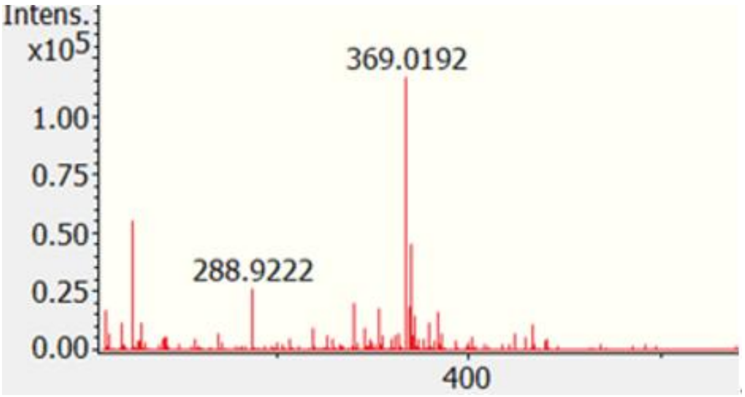

HR-ESIMS spectrum

(±)Ethyl-3-(2,6-dichloro-9H-purin-9-yl)-4-hydroxytetrahydrothiophene-3-carboxylate (**40a**)

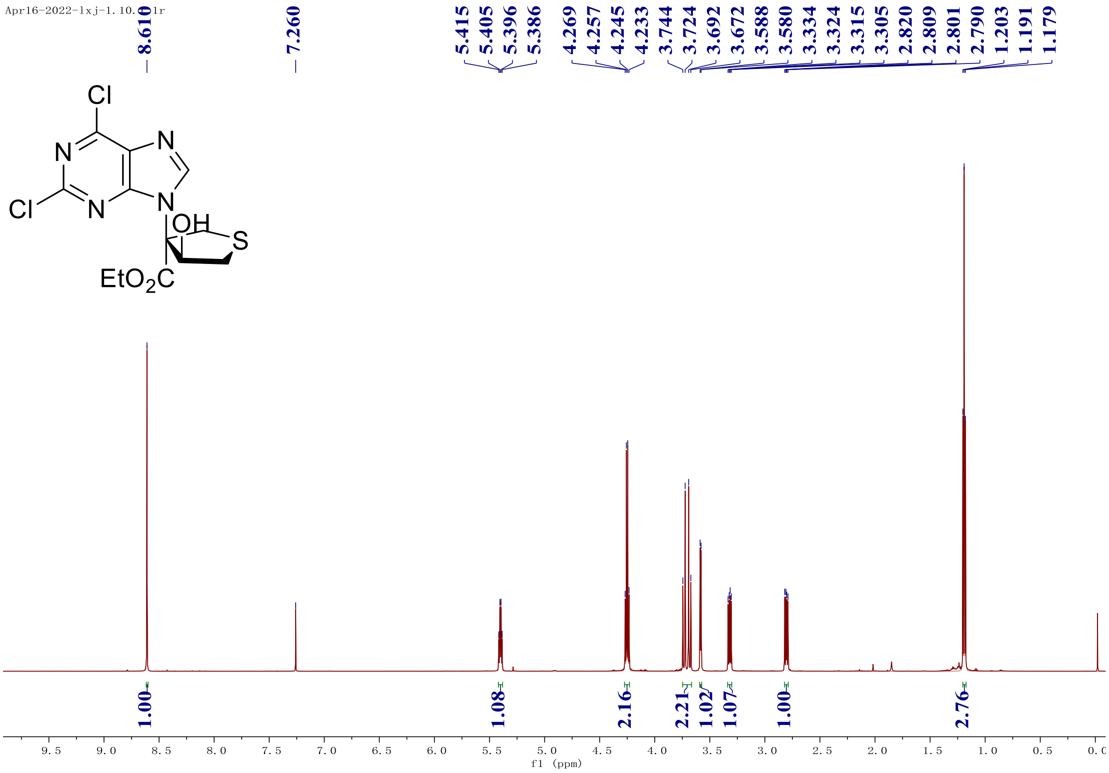

^{1}H NMR spectrum

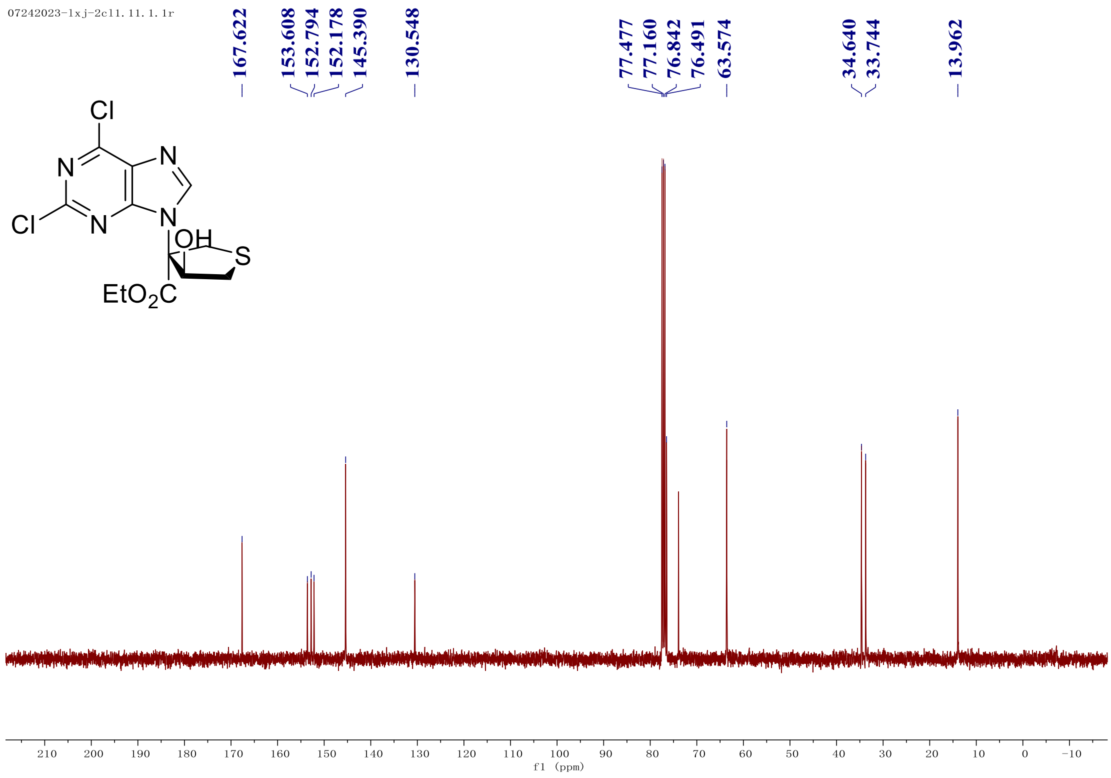

^{13}C NMR spectrum

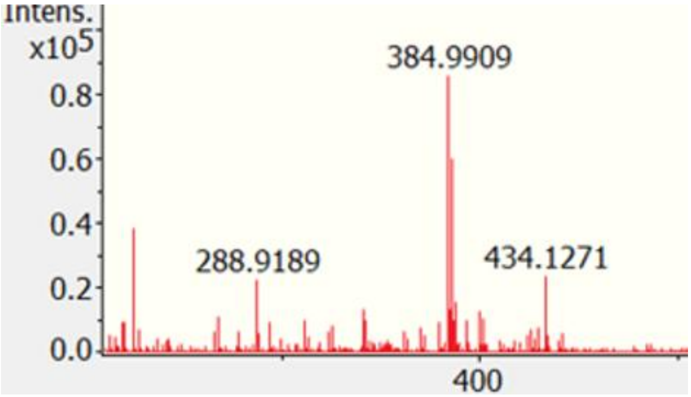

HR-ESIMS spectrum

(±)Ethyl-3-(2,6-dichloro-9H-purin-9-yl)-4-hydroxytetrahydrothiophene-3-carboxylate (**40b**)

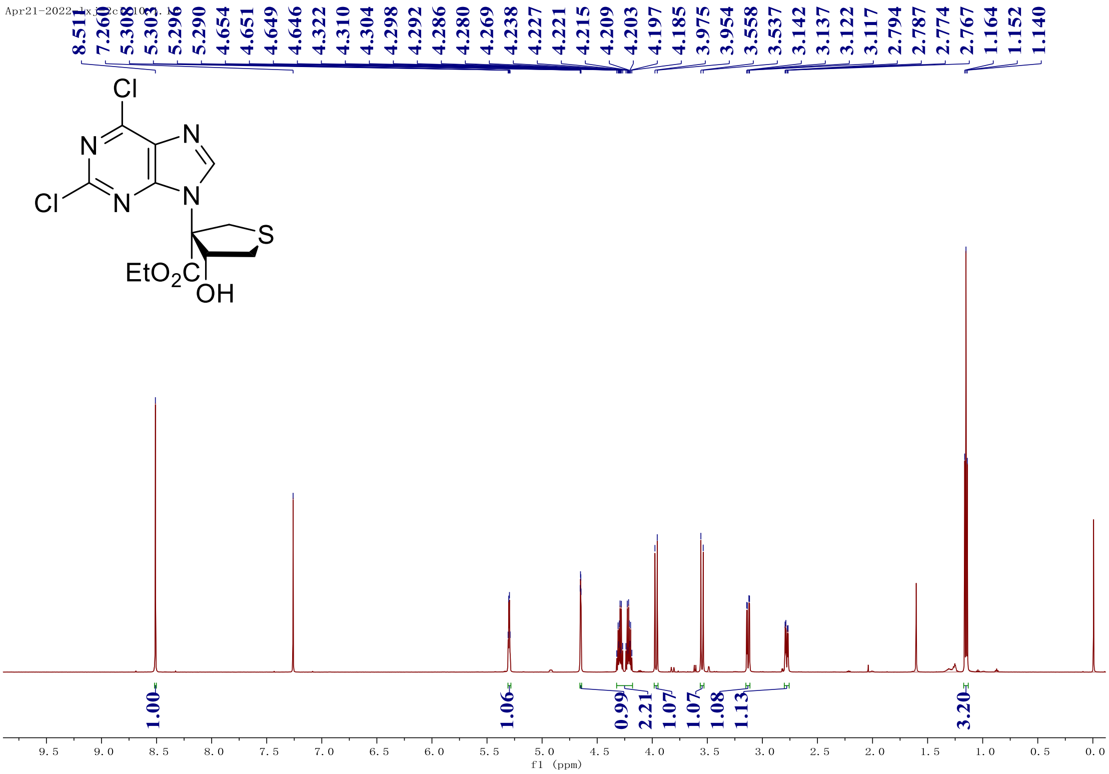

^{1}H NMR spectrum

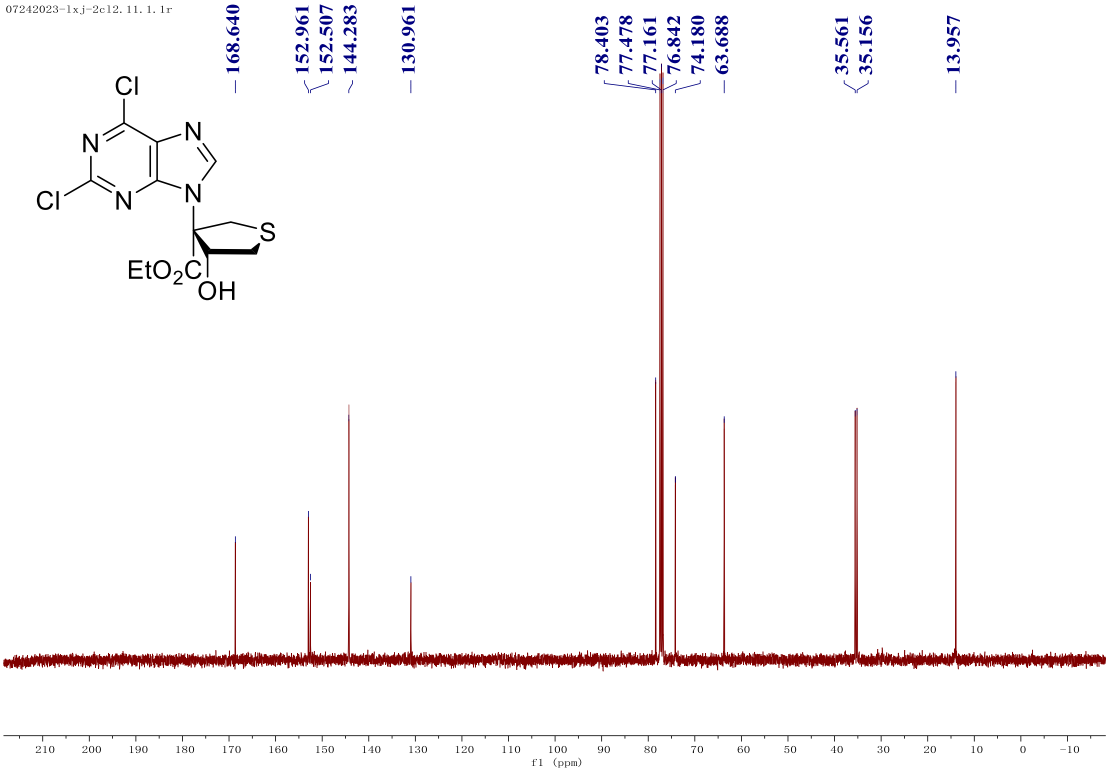

^{13}C NMR spectrum

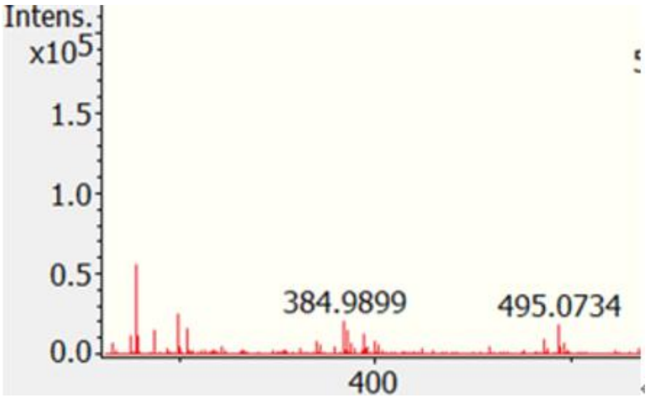

HR-ESIMS spectrum

(±)Ethyl-3-(2-chloro-9H-purin-9-yl)-4-hydroxytetrahydrothiophene-3-carboxylate (**41a**)

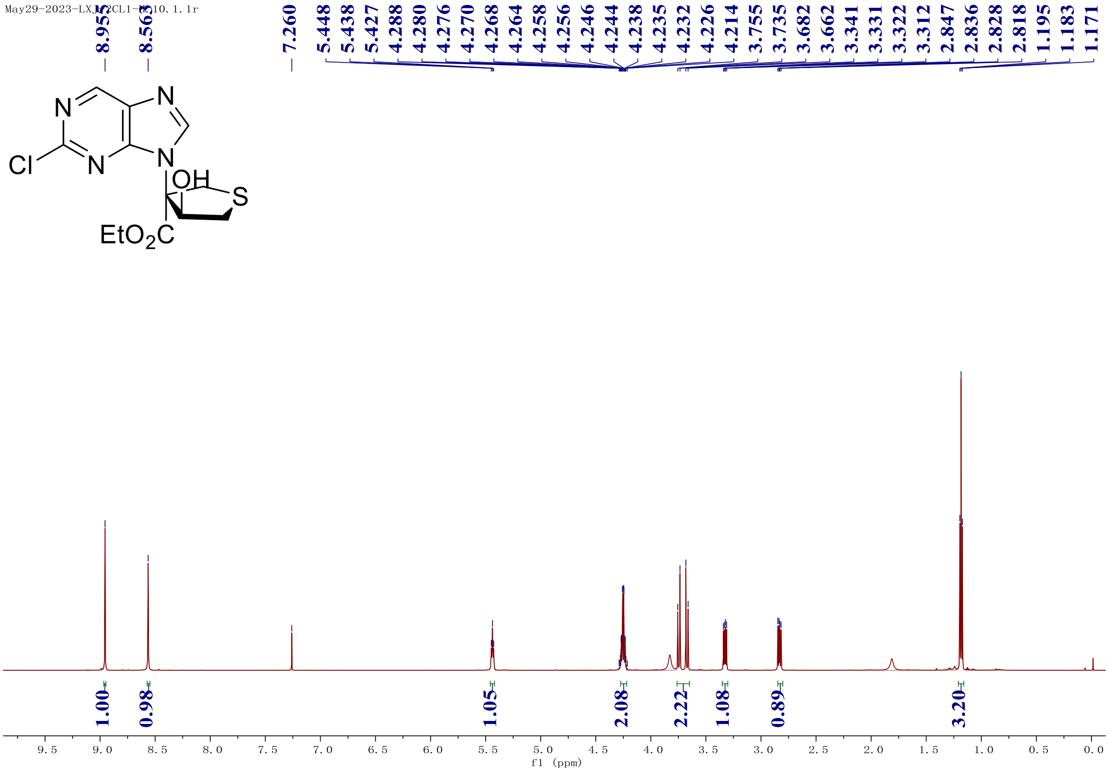

^{1}H NMR spectrum

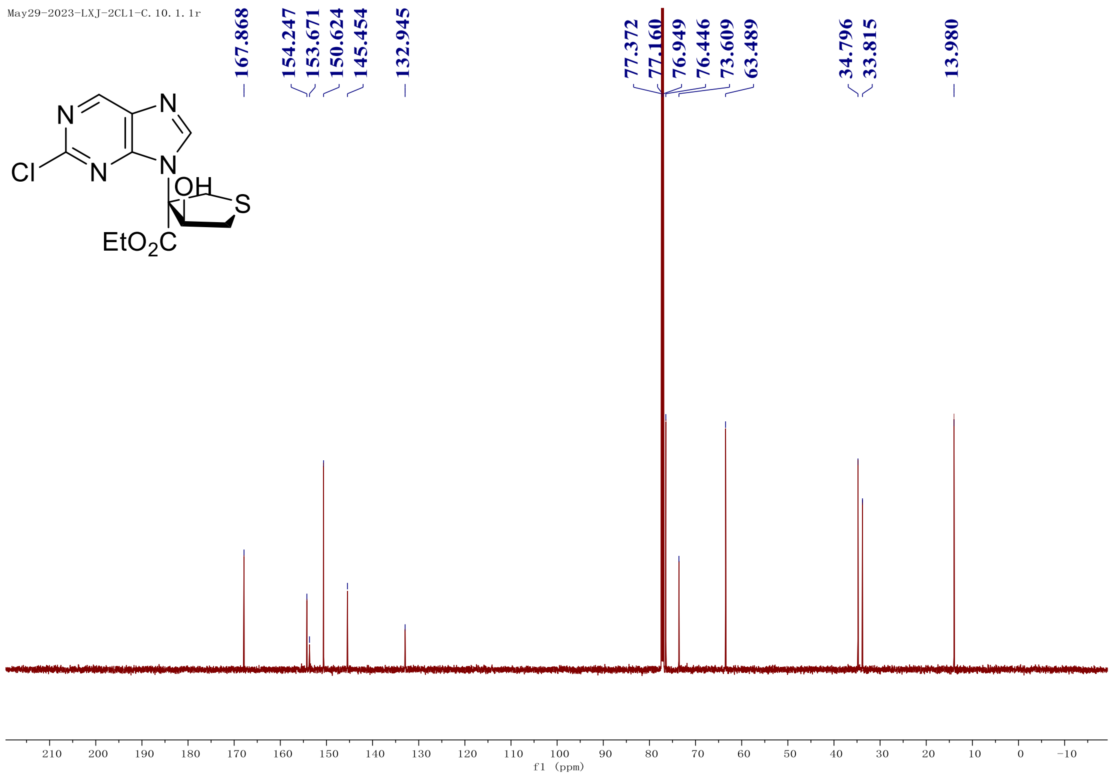

^{13}C NMR spectrum

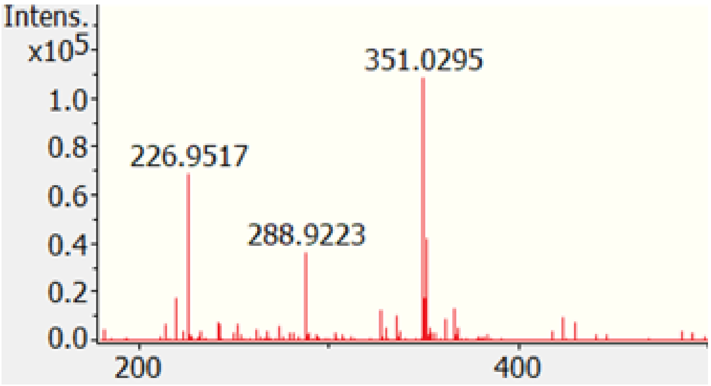

HR-ESIMS spectrum

(±)Ethyl-3-(2-chloro-9H-purin-9-yl)-4-hydroxytetrahydrothiophene-3-carboxylate

(**41b**)

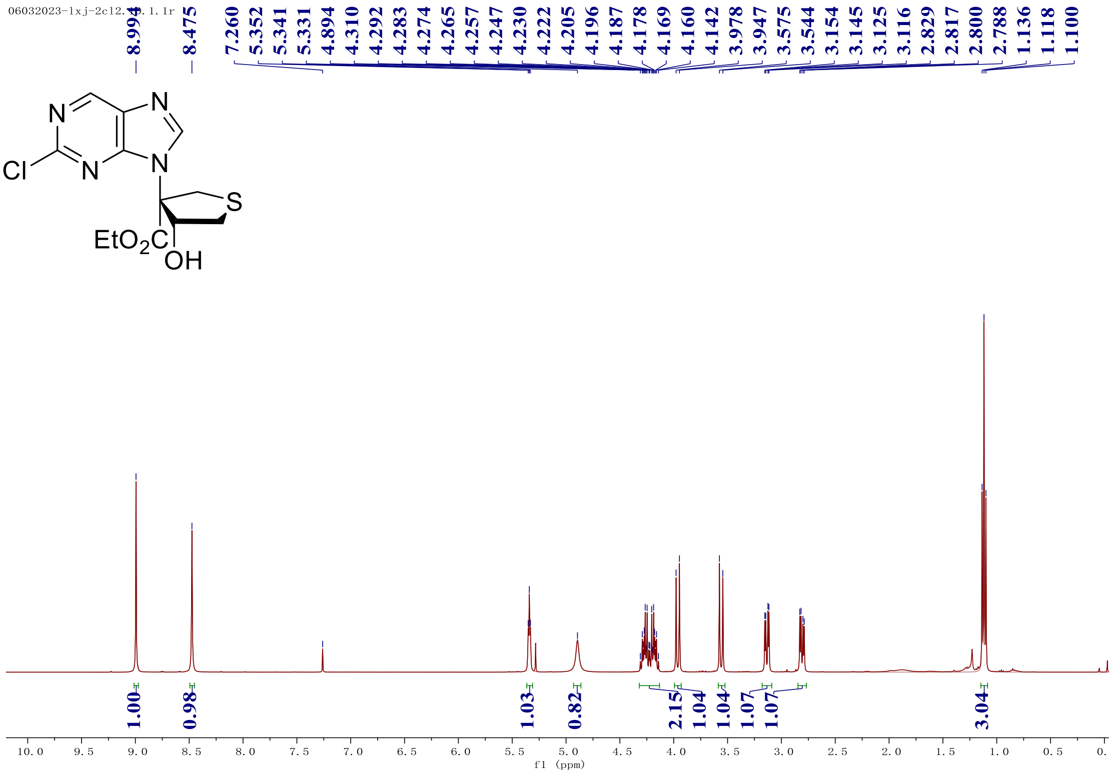

^{1}H NMR spectrum

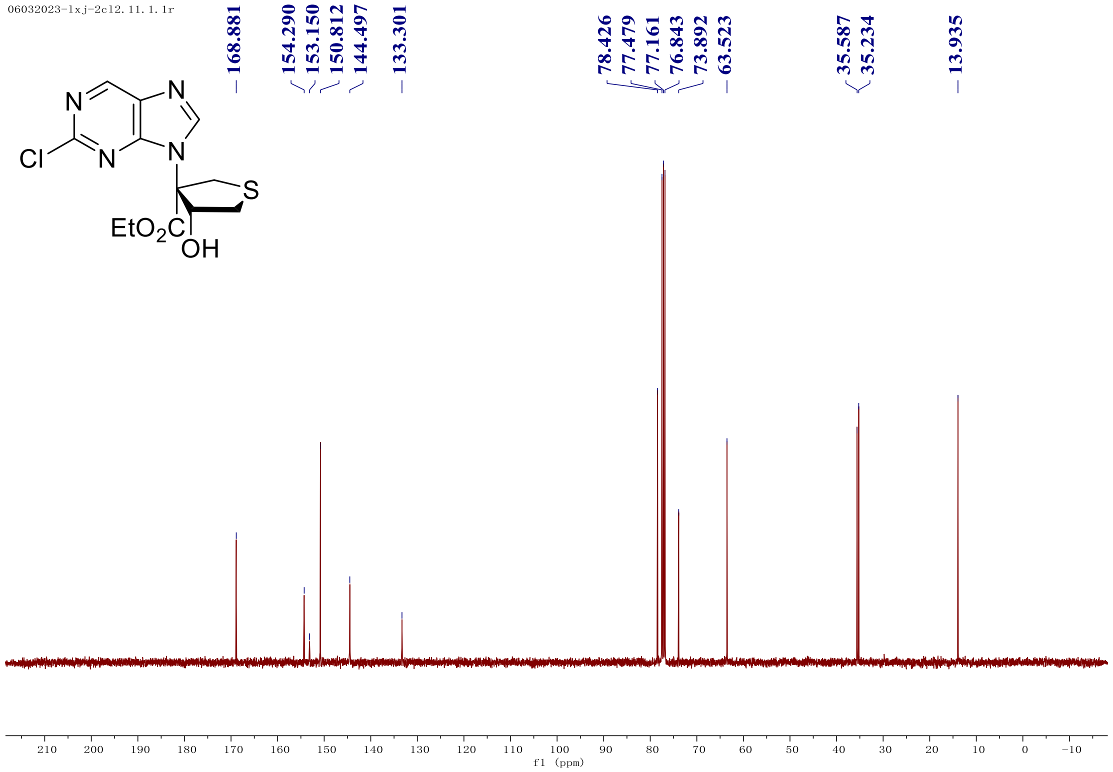

^{13}C NMR spectrum

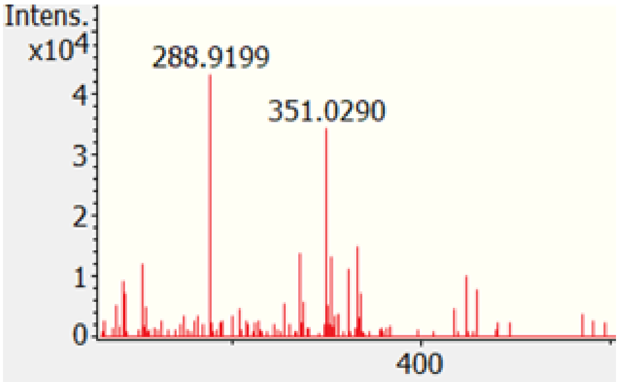

HR-ESIMS spectrum

(±)Ethy-3-(2-chloro-6-morpholino-9*H*-purin-9-yl)-4-hydroxytetrahydrothiophene-3-carboxylate (**42a**)

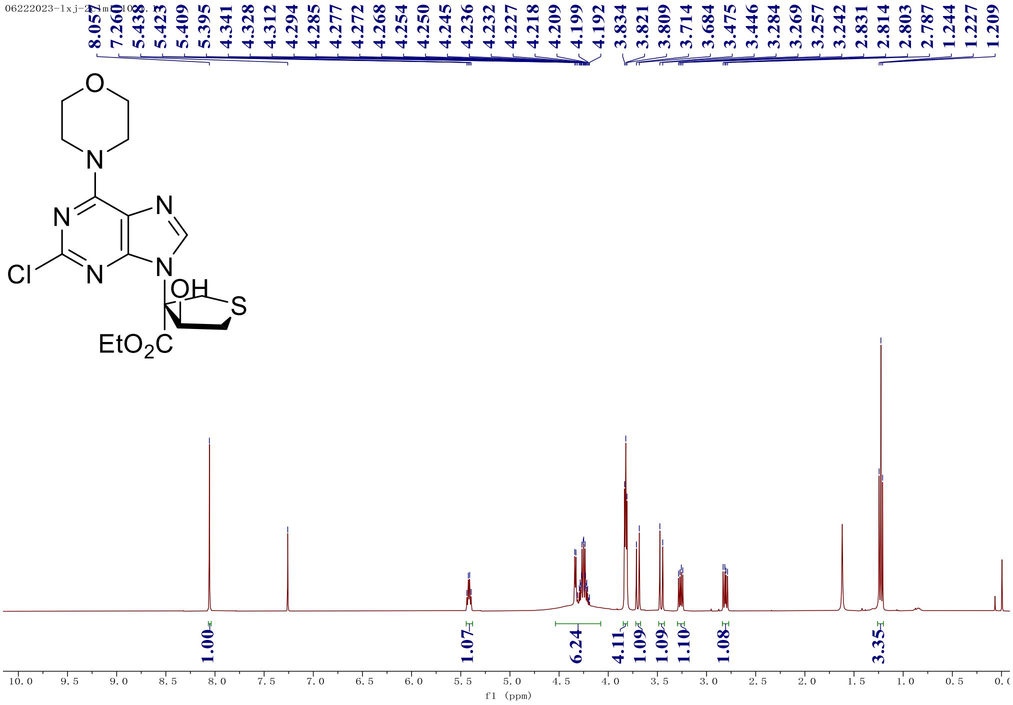

^{1}H NMR spectrum

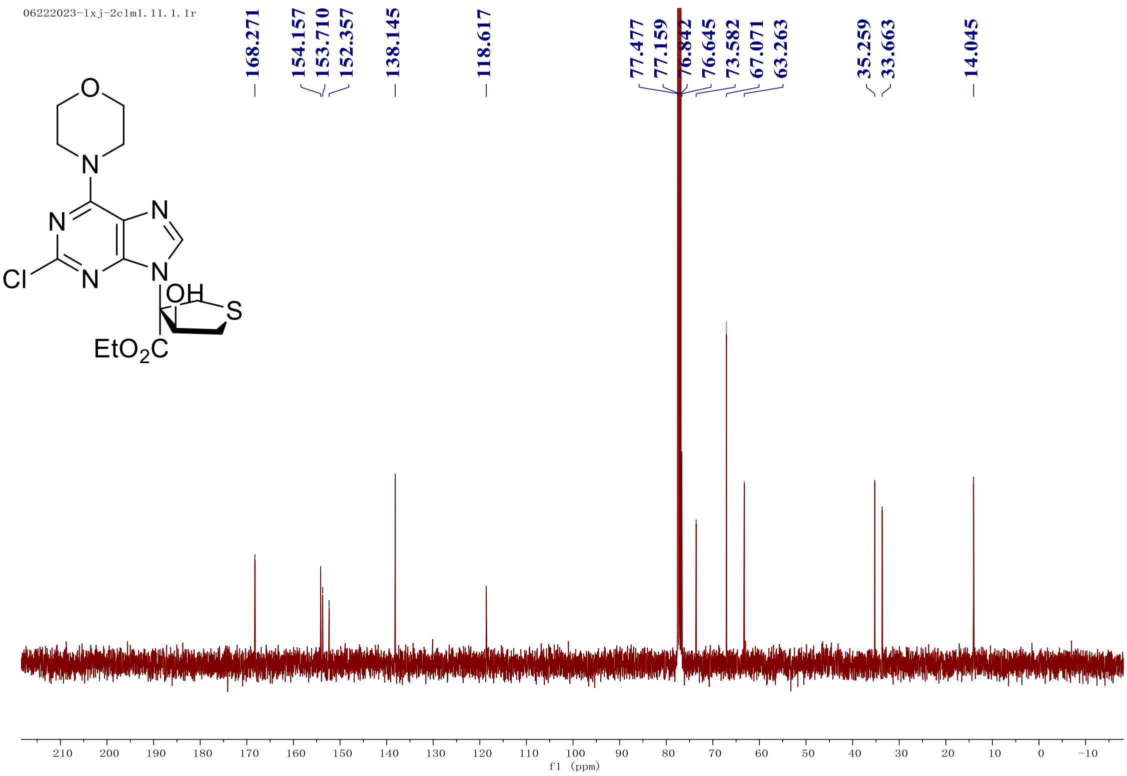

^{13}C NMR spectrum

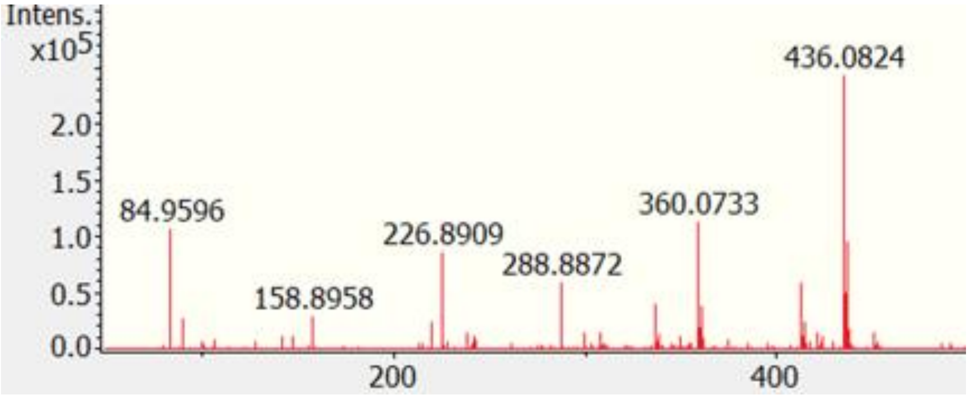

HR-ESIMS spectrum

(±)Ethy-3-(2-chloro-6-morpholino-9*H*-purin-9-yl)-4-hydroxytetrahydrothiophene-3-carboxylate (**42b**)

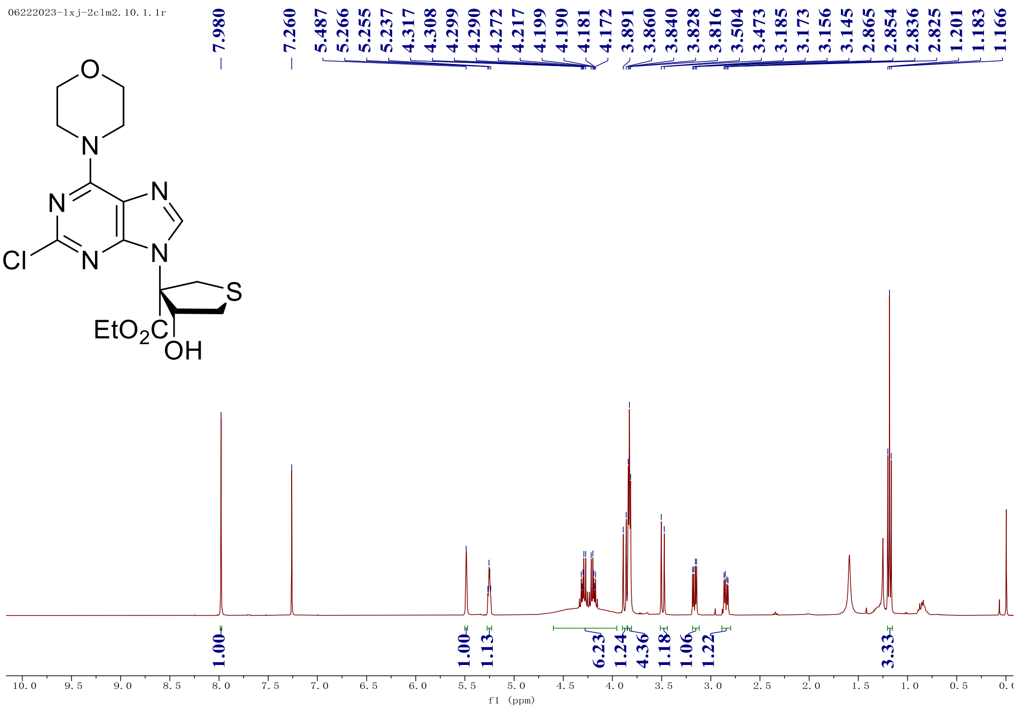

^{1}H NMR spectrum

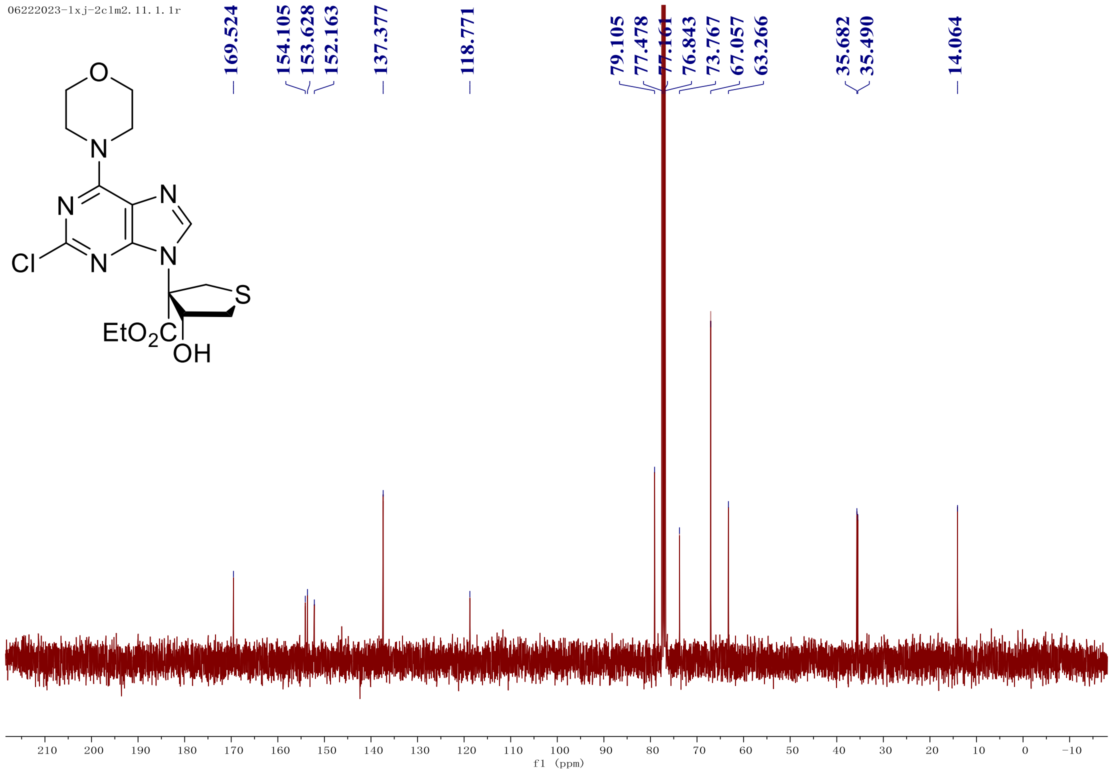

^{13}C NMR spectrum

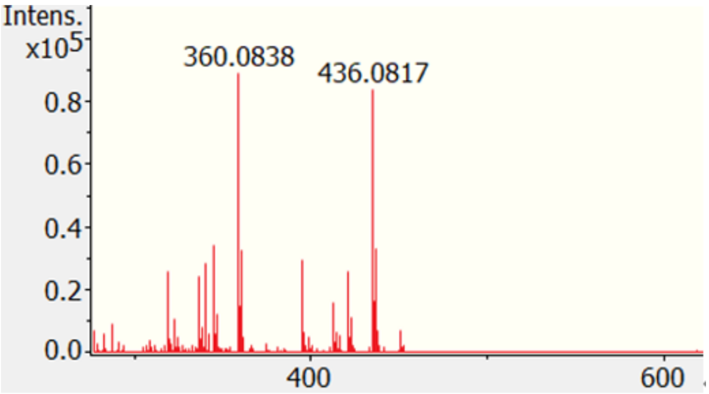

HR-ESIMS spectrum

1.4. HPLC chart for compounds ***33a, 36b***

*1.4.1. Ethyl-3-(2-fluoro-6-(piperidin-1-yl)-9H-purin-9-yl)-4-hydroxytetrahydrothiophene-3-carboxylate (**33a**)*

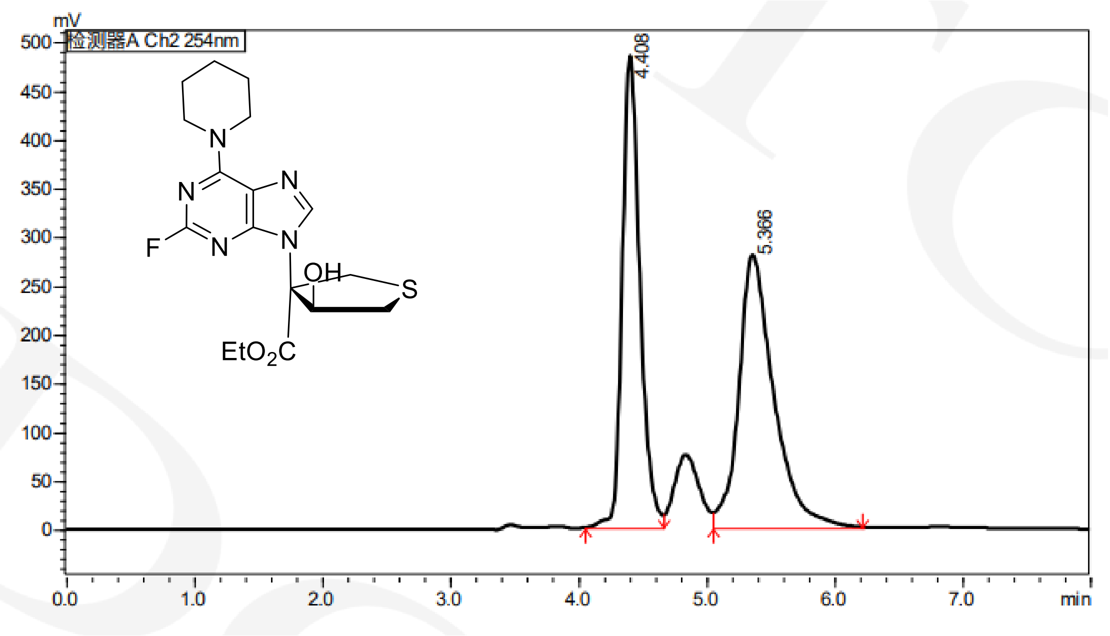

<Peak Table>

Peak#	Ret. Time	Area	Area%	T.Plate#	Tailing F.	Resolution
1	4.408	4688617	47.076	4672	1.297	–
2	5.366	5271072	52.924	2095	–	2.635

–, not applicable.

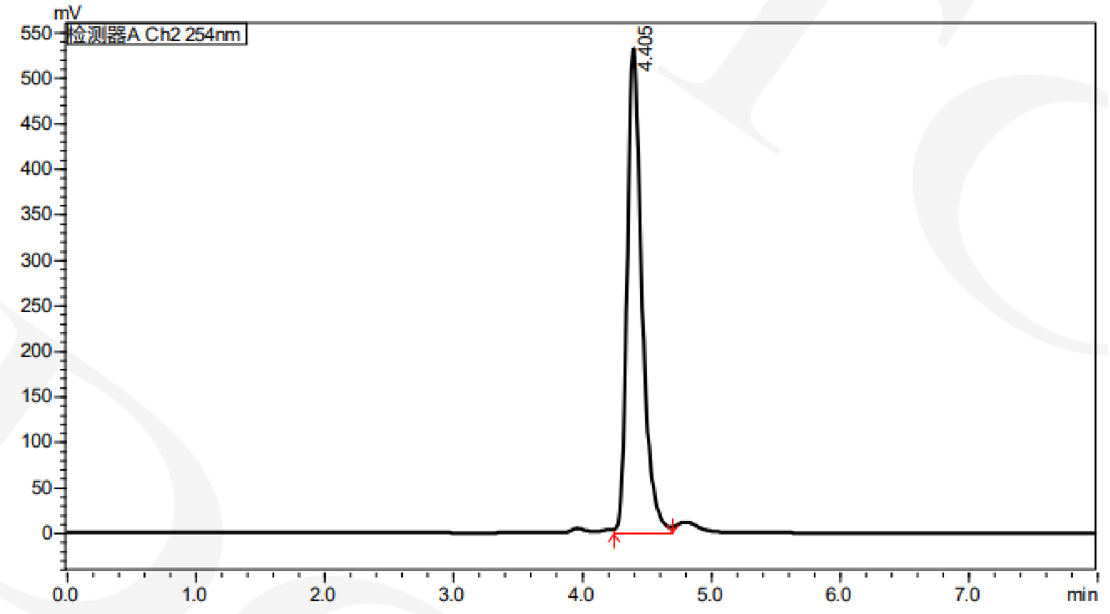

<Peak Table>

Peak#	Ret. Time	Area	Area%	T.Plate#	Tailing F.	Resolution
1	4.405	4245370	100.000	6817	1.390	–

–, not applicable.

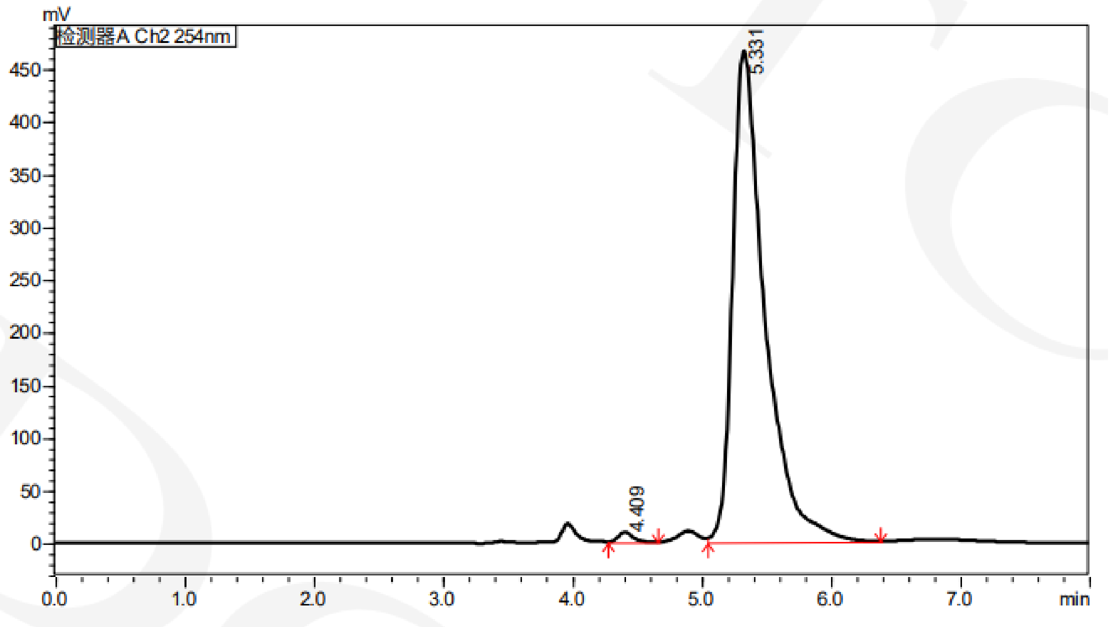

<Peak Table>

Peak#	Ret. Time	Area	Area%	T.Plate#	Tailing F.	Resolution
1	4.409	68584	0.841	7239	1.241	–
2	5.331	8082227	99.159	2065	1.716	2.951

*1.4.2. Ethyl-3-(2-fluoro-6-(propylthio)-9H-purin-9-yl)-4-hydroxytetrahydrothiophene-3-carboxylate (**36b**)*

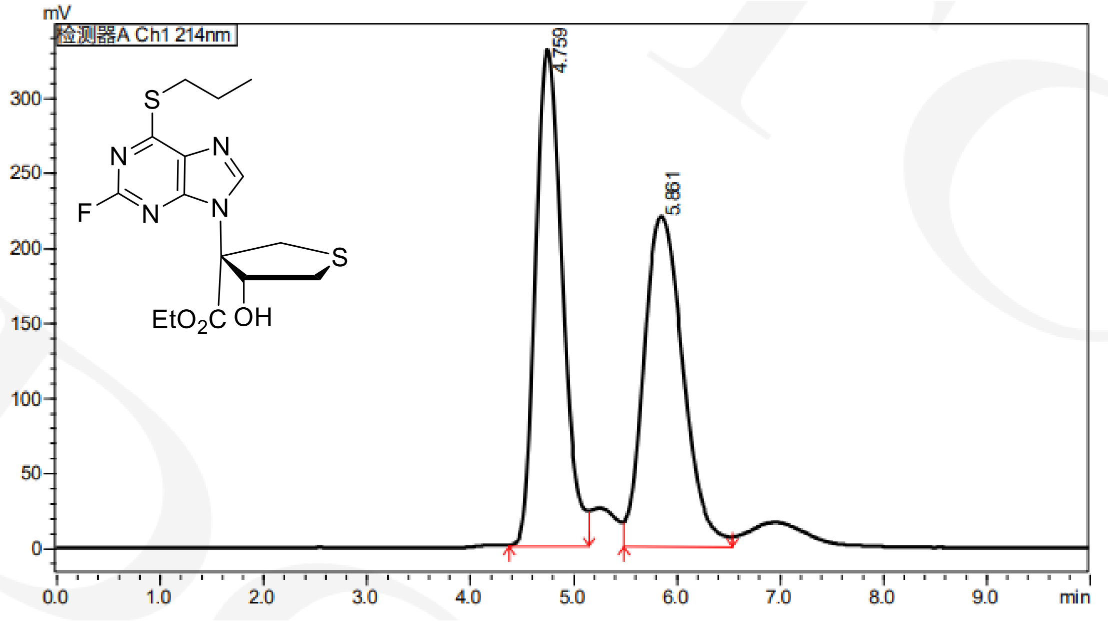

<Peak Table>

Peak#	Ret. Time	Area	Area%	T.Plate#	Tailing F.	Resolution
1	4.759	5982170	50.846	1593	–	–
2	5.861	5783201	49.154	1167	–	1.895

–, not applicable.

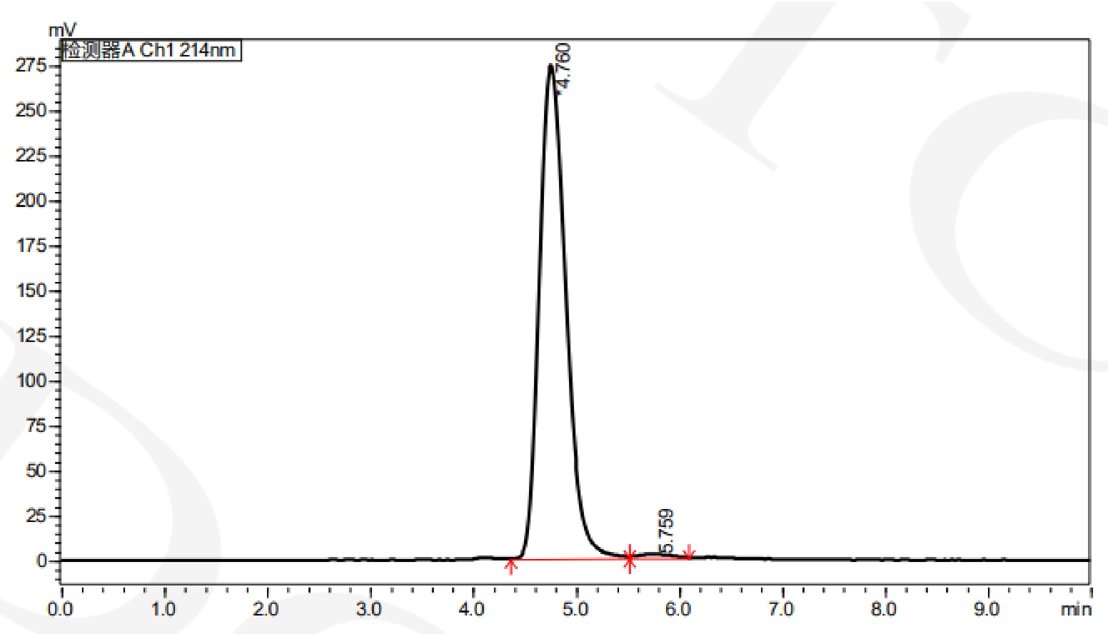

<Peak Table>

Peak#	Ret. Time	Area	Area%	T.Plate#	Tailing F.	Resolution
1	4.760	4914093	99.179	1636	1.269	–
2	5.759	40700	0.821	1625	–	1.918

–, not applicable.

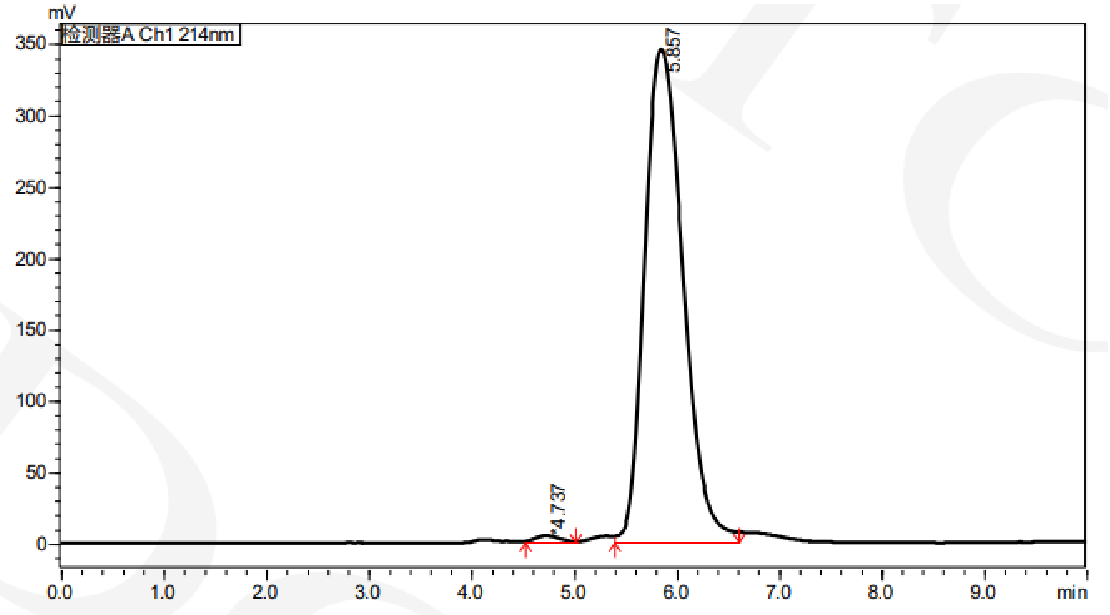

<Peak Table>

Peak#	Ret. Time	Area	Area%	T.Plate#	Tailing F.	Resolution
1	4.737	6520	0.690	2141	1.126	–
2	5.857	8997813	99.310	1182	1.279	2.054

–, not applicable.

2. X-Ray Crystallographic Analysis of 33aa (SS) (CCDC 2324828)

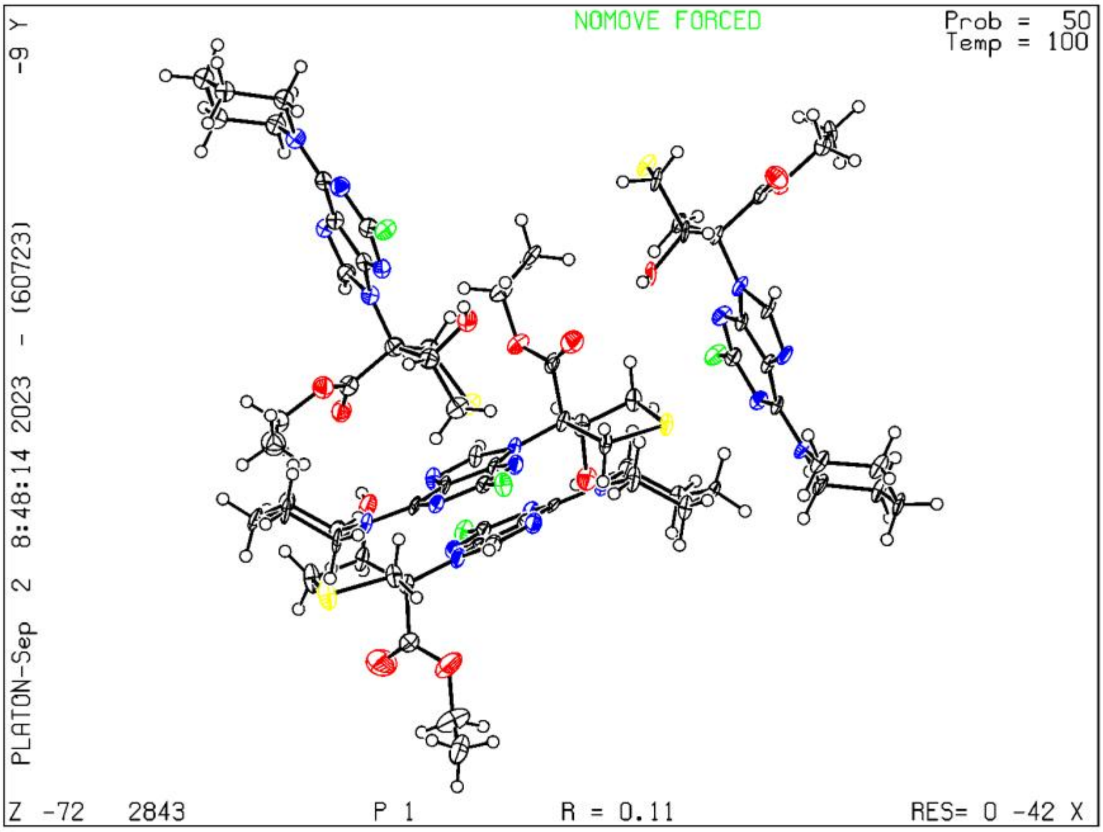

Figure S1 X-ray crystal structure of **33aa** (*SS*)

Table S1 Crystal data and structure refinement for **33aa** (*SS*)

Identification code	**33aa**
Empirical formula	$C_{17}H_{22}FN_5O_3S$
Formula weight	395.45
Temperature/K	99.98(11)
Crystal system	triclinic
Space group	27a
a/Å	11.6588(6)
b/Å	11.8931(5)
c/Å	12.8609(4)
α/°	94.579(3)
β/°	91.329(3)

γ/°	91.346(4)
Volume/Å^3	1776.55(13)
Z	4
ρ_{calc}g/cm^3	1.479
μ/mm^{-1}	1.979
F(000)	832.0
Crystal size/mm^3	0.16 × 0.14 × 0.12
Radiation	Cu Kα (λ = 1.54184)
2Θ range for data collection/°	6.898 to 150.696
Index ranges	-14 ≤ h ≤ 12, -14 ≤ k ≤ 13, -15 ≤ l ≤ 15
Reflections collected	9667
Independent reflections	9667 [R_{int} = ?, R_{sigma} = 0.0394]
Data/restraints/parameters	9667/1037/982
Goodness-of-fit on F^2	1.097
Final R indexes [I>=2σ (I)]	R_1 = 0.1121, wR_2 = 0.3242
Final R indexes [all data]	R_1 = 0.1141, wR_2 = 0.3255
Largest diff. peak/hole / e Å^{-3}	1.36/-0.96
Flack/Hooft parameter	0.04(3)/0.020(5)

3. X-Ray Crystallographic Analysis of 36ba (*SR*) (CCDC 2324827)

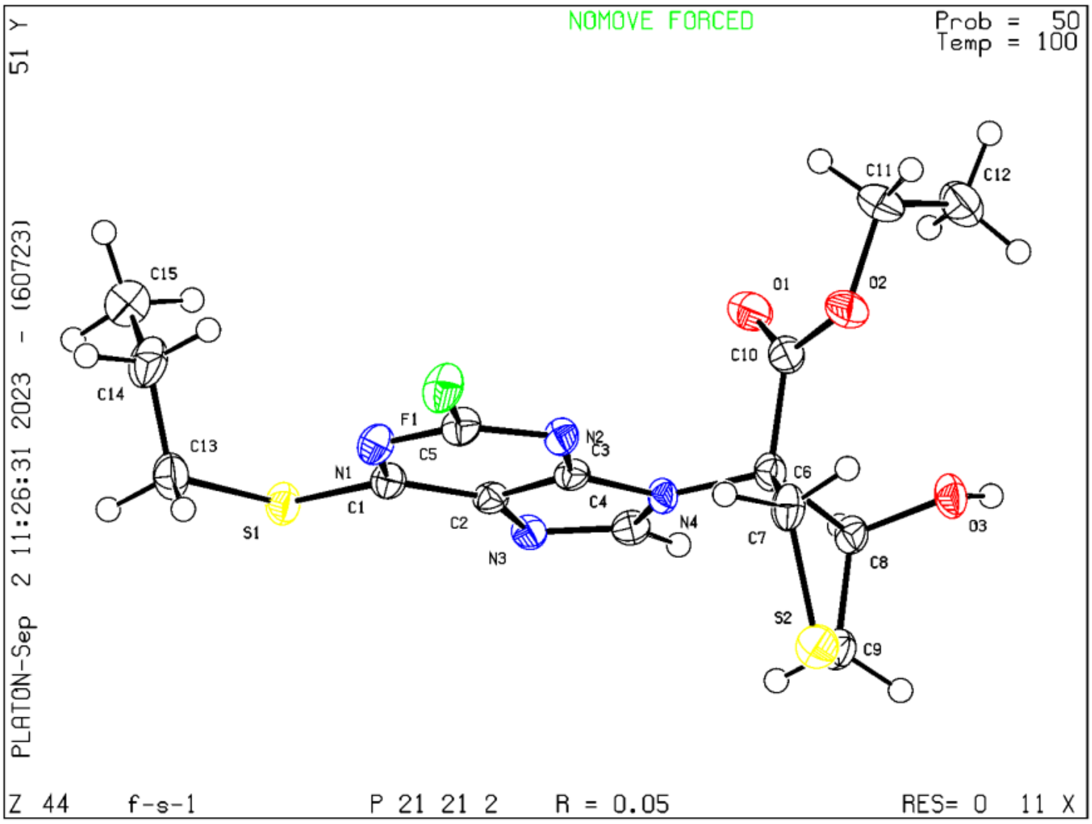

Figure S2 X-ray crystal structure of **36ba** (*SR*)

Table S2 Crystal data and structure refinement for **36ba** (SR)

Identification code	**36ba**
Empirical formula	$C_{15}H_{19}FN_4O_3S_2$
Formula weight	386.46
Temperature/K	99.95(14)
Crystal system	orthorhombic
Space group	$P2_12_12$
a/Å	11.0981(2)
b/Å	12.2885(3)
c/Å	12.5913(3)
α/°	90
β/°	90

γ/°	90
Volume/Å^3	1717.19(7)
Z	4
ρ_{calc}g/cm^3	1.495
μ/mm^{-1}	3.122
F(000)	808.0
Crystal size/mm^3	0.14 × 0.12 × 0.1
Radiation	Cu Kα (λ = 1.54184)
2Θ range for data collection/°	7.02 to 148.712
Index ranges	-8 ≤ h ≤ 13, -15 ≤ k ≤ 13, -13 ≤ l ≤ 15
Reflections collected	9375
Independent reflections	3413 [R_{int} = 0.0413, R_{sigma} = 0.0435]
Data/restraints/parameters	3413/0/229
Goodness-of-fit on F^2	1.090
Final R indexes [I>=2σ (I)]	R_1 = 0.0545, wR_2 = 0.1432
Final R indexes [all data]	R_1 = 0.0564, wR_2 = 0.1448
Largest diff. peak/hole / e Å^{-3}	0.85/-0.37
Flack/Hooft parameter	-0.009(12)/0.006(9)

4. Biology

4.1 Supplemental Table and Figures

Table S3 Inhibitory effects of compounds **38a/38b** to **42a/42b** on cancer cell proliferation[a]

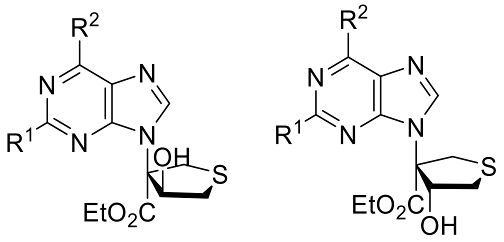

(±)–**a** (±)–**b**

Compd.	R^1	R^2	IC$_{50}$ (μM)

			HeLa	HCT116
**38a**	NH_2	Cl	25.64±2.13	30.53±1.42
**38b**	NH_2	Cl	22.50±2.17	10.59±1.02
**39a**	F	Cl	11.23±1.32	3.42±0.42
**39b**	F	Cl	9.33±1.17	2.01±0.24
**40a**	Cl	Cl	8.21±1.25	5.37±1.02
**40b**	Cl	Cl	4.45±1.07	2.13±0.46
**41a**	Cl	H	>50	>50
**41b**	Cl	H	>50	>50
**42a**	Cl	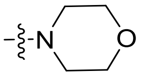	6.32±1.02	24.15±1.32
**42b**	Cl	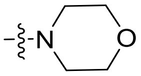	3.21±0.65	4.48±0.56
5-FU	–	–	4.93±1.02	9.84±2.32
Cisplatin	–	–	11.25±0.81	5.29±0.25

[a]Data are mean ± SD values from three independent experiments.

–, not applicable.

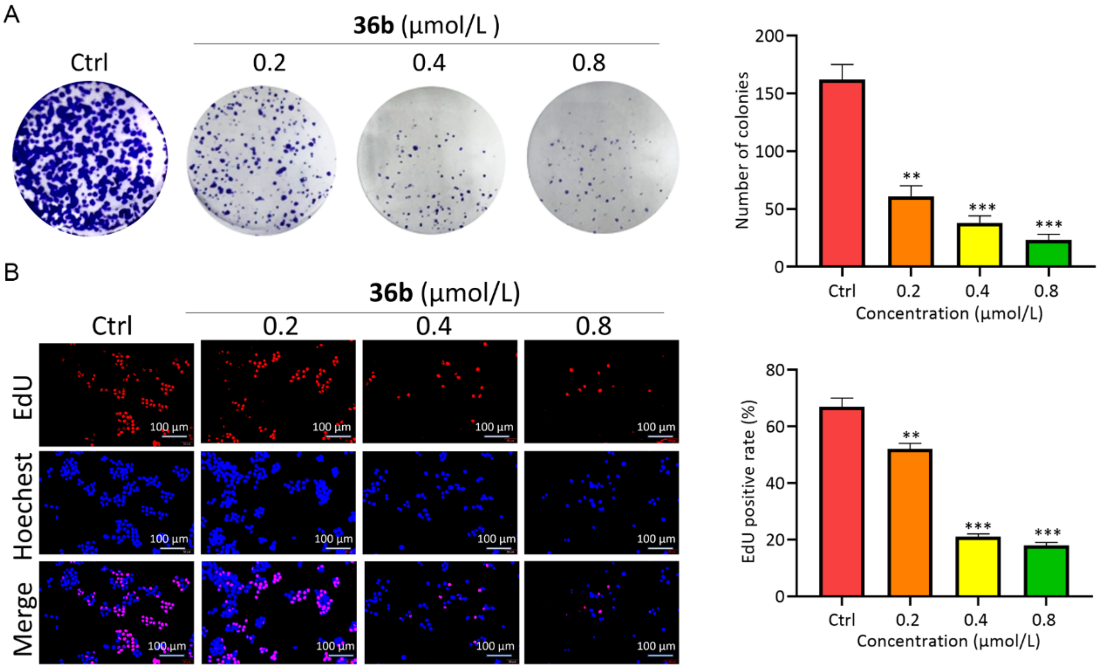

Figure S3 Inhibition of the proliferation by compounds **36b**. (A) Colony formation

analysis of **36b** in HCT116 cell line (cells were incubated for 48 h with each respective compound, followed by incubation in fresh media for an additional 14 days). (B) EdU assays analysis of **36b** in HCT116 cell line for 48 h. Scale bar = 100 μm. Data are mean ± SD (*n* = 3), ***P* < 0.01, and ****P* < 0.001 compared with the control cells.

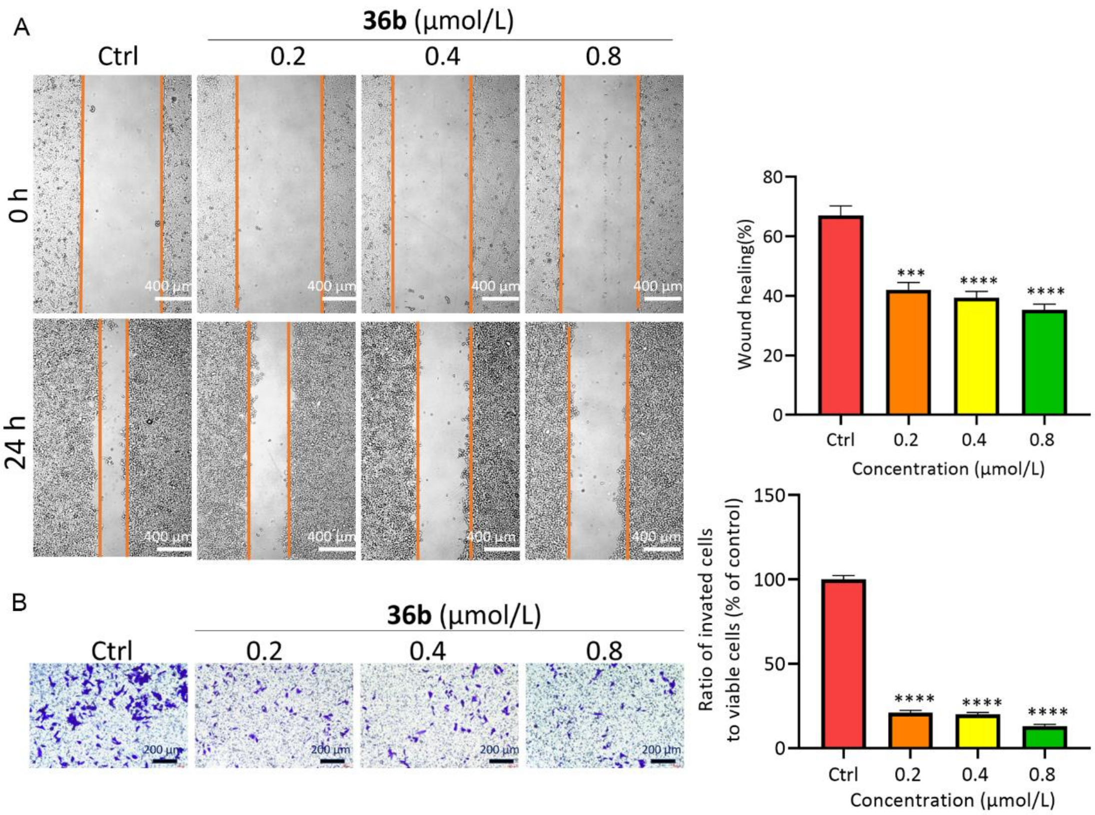

Figure S4 Migration and invasion inhibition of compound **36b**. (A) Wound healing assay analysis of **36b** in HCT116 cell line for 24 h. Scale bar = 400 μm. (B) Transwell assays analysis of **36b** in HCT116 cell line for 24 h. Scale bar = 400 μm. Data are mean ± SD (*n* = 3), ****P* < 0.001 and *****P* < 0.0001 compared with the control group.

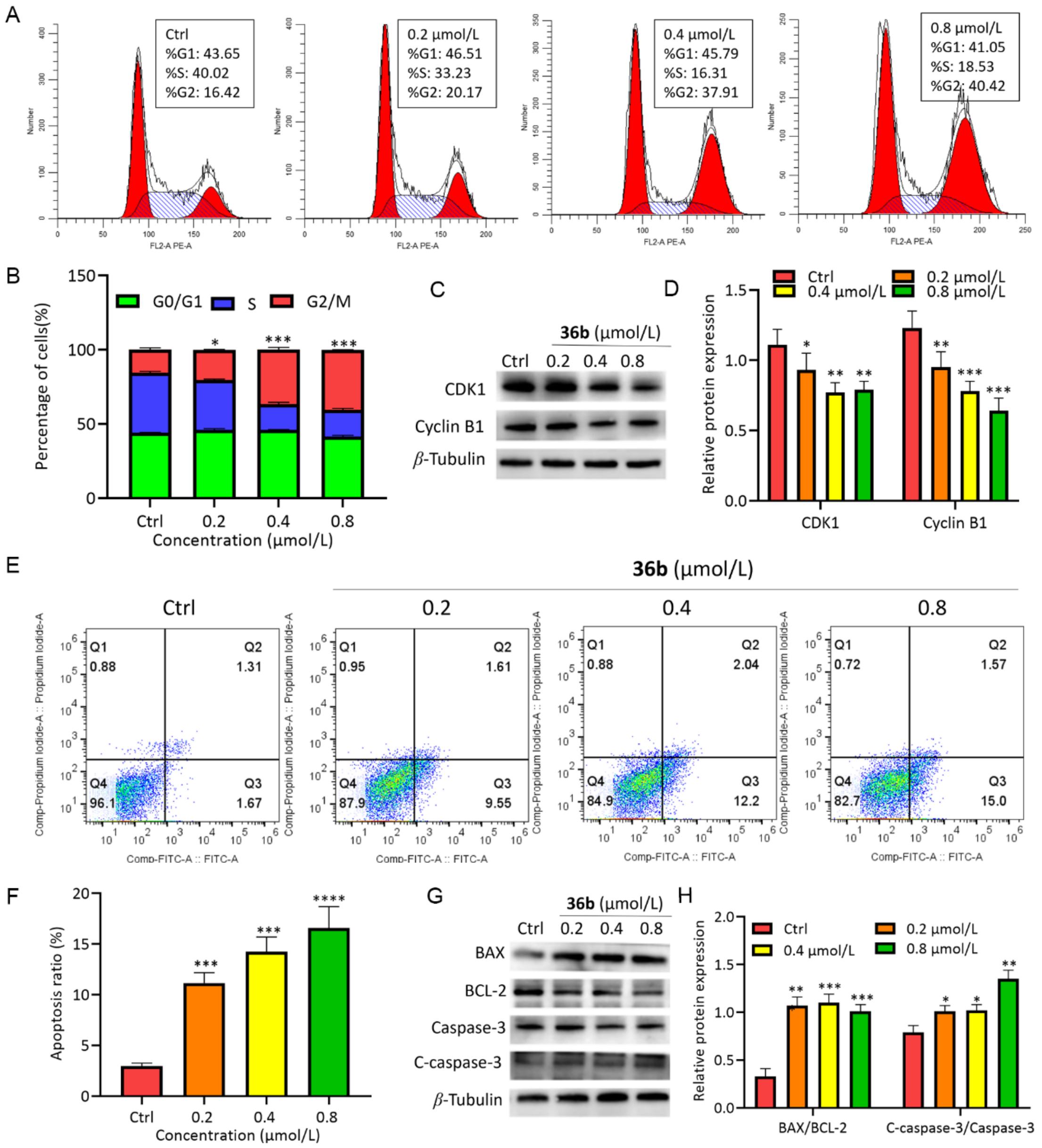

Figure S5 Cell cycle arrest and apoptosis induced by compound **36b** in HCT116 for 48 h. (A–B) G2/M block induced by compound **36b** treated for 48 h. (C–D) Western blotting analysis of cell cycle-related proteins. (E–F) Apoptosis induced by compound **36b**. (G-H) Western blotting analysis of cell apoptosis-related proteins. Data are mean ± SD (n = 3), **P* < 0.05, ***P* < 0.01, ****P* < 0.001, and *****P* < 0.0001 compared with the control group.

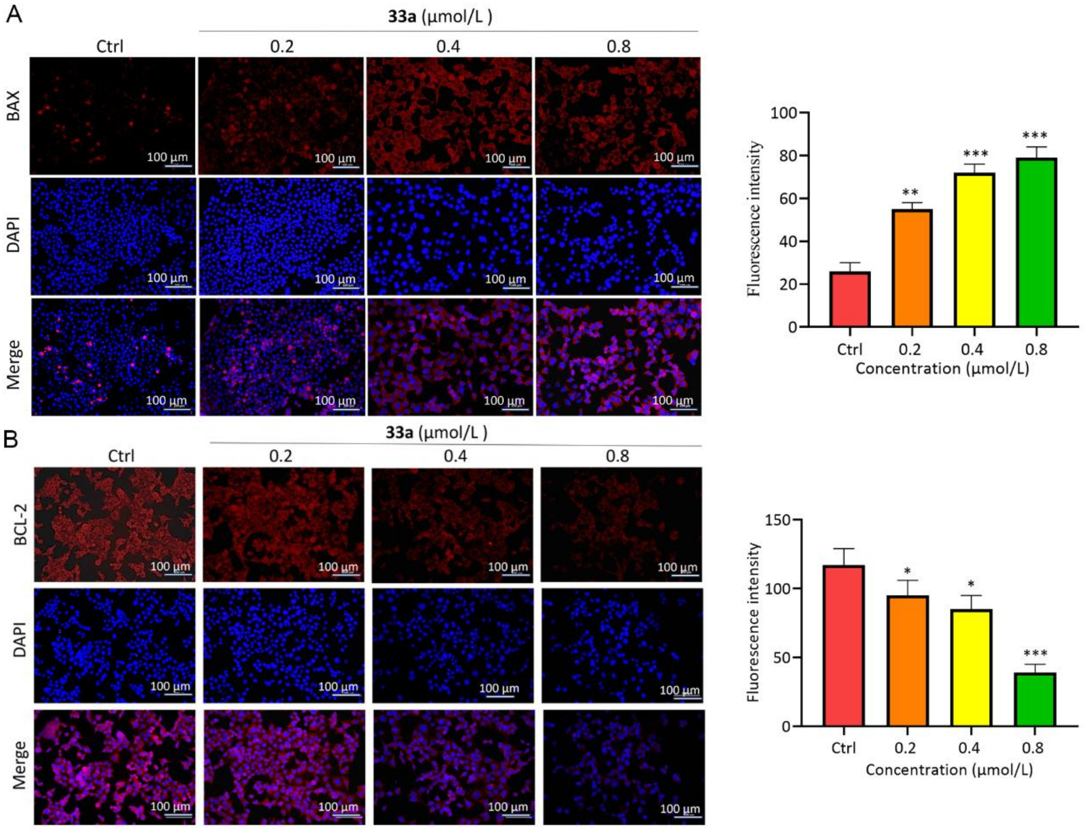

Figure S6 Immunofluorescence staining analysis of cell apoptosis-related proteins BAX and BCL-2. Scale bar = 100 μm. Data are mean ± SD (*n* = 3), *P < 0.05, **P < 0.01, and ***P < 0.001 compared with the control group.

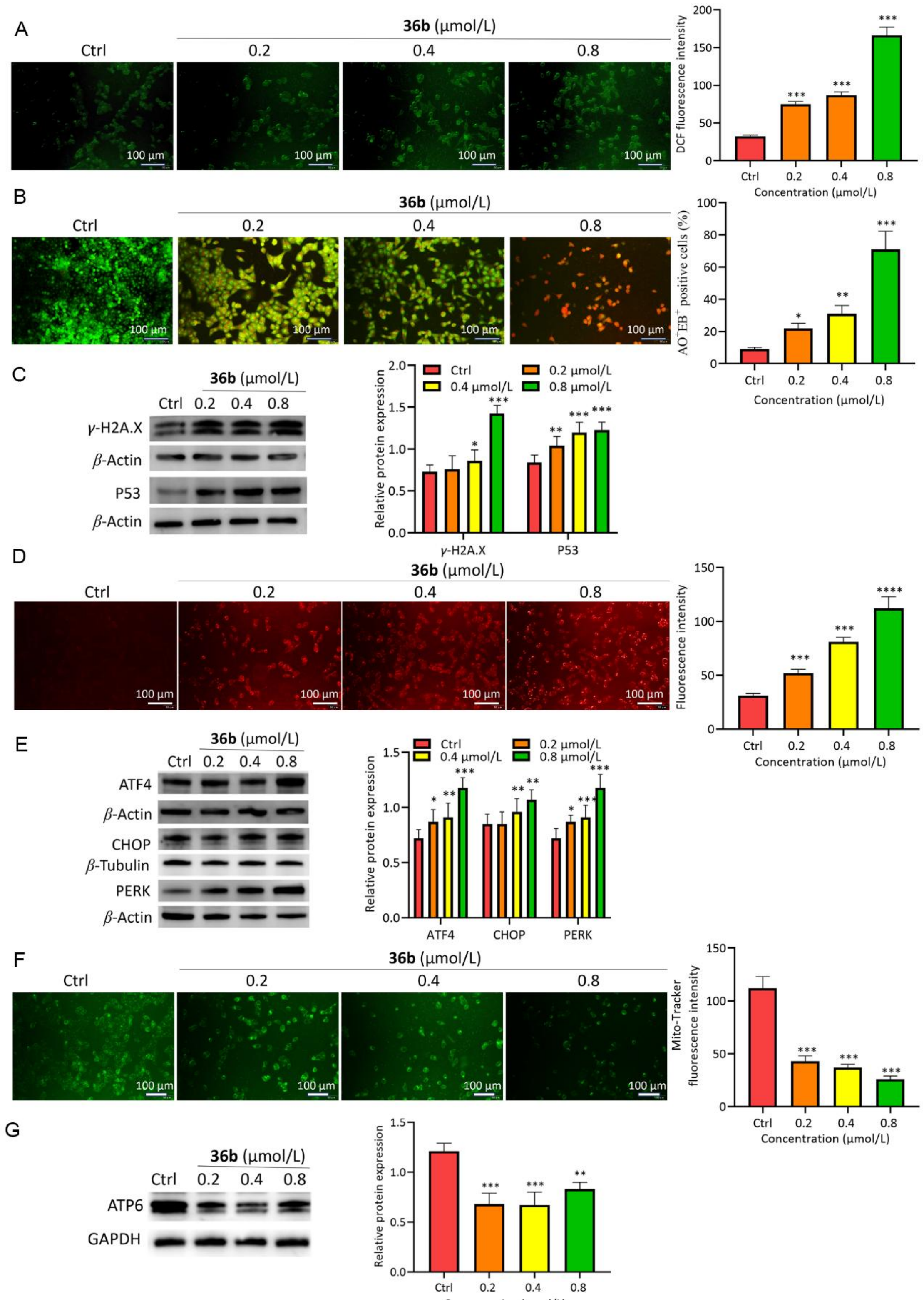

Figure S7 ROS production, DNA damage, ER stress, and mitochondrial cell death pathways induced by compound **36b** for 48 h. (A) ROS levels were examined by DCFH-DA. Scale bar = 100 μm. (B) AO staining assessment of **36b**-treated HCT116 cells. Scale bar = 100 μm. (C) Western blotting analysis of the expression levels of γ-H2AX

and P53. (D) ER stress was evaluated by ER-Tracker Red. Scale bar = 100 μm. (E) Western blotting analysis of PERK, ATF4, and CHOP expression. (F) Mitochondrial damage found by Mito-Tracker Green probe. Scale bar = 100 μm. and (G) Western blotting analysis of ATP6 expression level. Data are mean ± SD (*n* = 3), **P* < 0.05, ***P* < 0.01, ****P* < 0.001, and *****P* < 0.0001 compared with the control group.

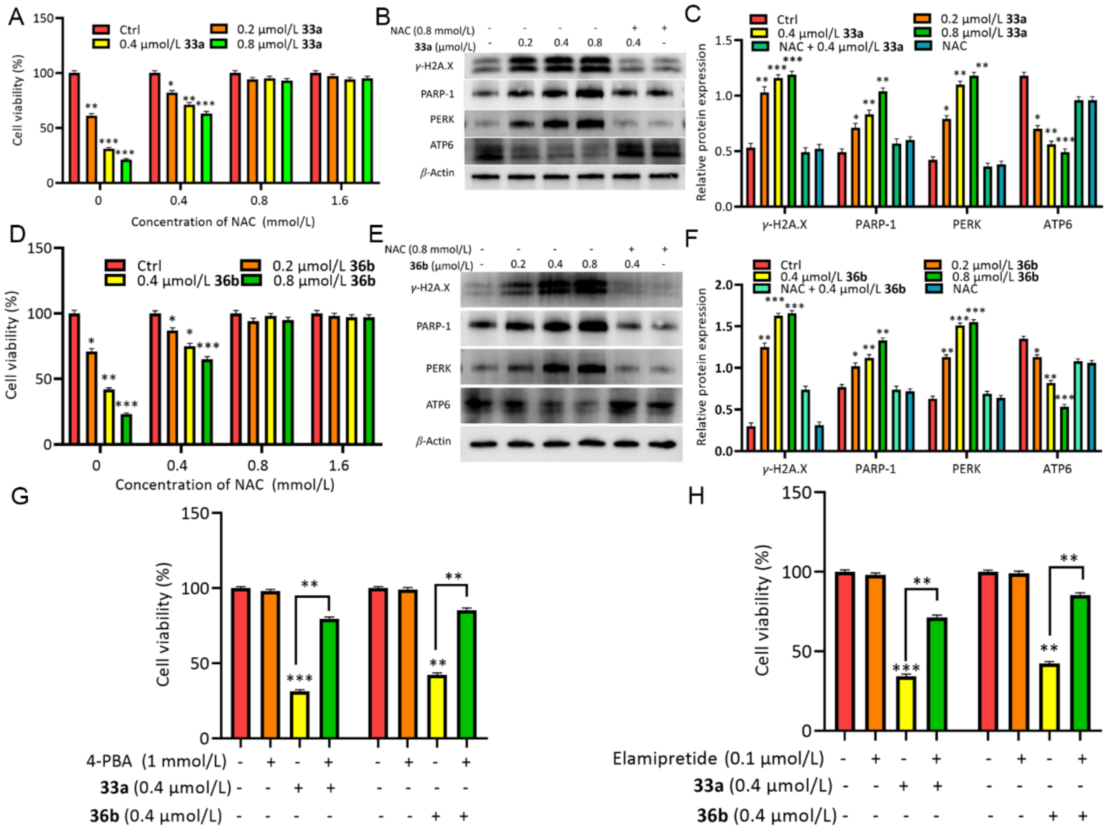

Figure S8 Compounds **33a** and **36b** trigger DNA damage, ER stress, and mitochondrial damage in HCT116 cells by boosting ROS. (A) The effect of NAC on the viability of HCT116 cells treated with **33a**. (B-C) The effect of NAC on *γ*-H2A.X, PARP-1, PERK, and ATP6 expressions of HCT116 cells treated with **33a**. (D) The effect of NAC on the viability of HCT116 cells treated with **36b**. (E-F) The effect of NAC on *γ*-H2A.X, PARP-1, PERK, and ATP6 expressions of HCT116 cells treated with **36b**. (G) The effect of 4-PBA on the viability of HCT116 cells treated with **33a** or **36b**. (H) The effect of elamipretide on the viability of HCT116 cells treated with **33a** or **36b**. Data are mean ± SD (*n* = 3), **P* < 0.05, ***P* < 0.01, and ****P* < 0.001.

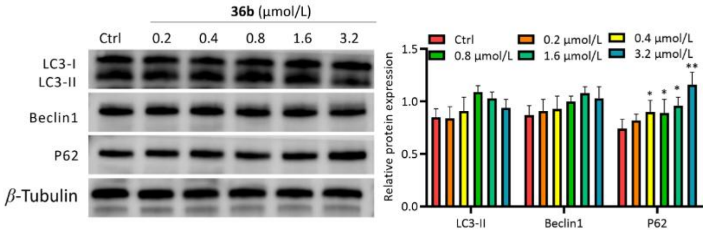

Figure S9 Autophagy inhibition of **36b**-treated HCT116 cells for 48 h. The data were mean ±SD (n = 3), $^{*}P$ <0.05, $^{**}P$ < 0.01 compared with the control cells.

Table S4 The top 10 genes with the most significant fold change.

ID	logFC	logCPM	PValue	FDR
NGFR	2.801281	7.808034	7.03E-57	1.65E-52
SLC44A2	1.969041	7.942247	5.99E-34	7.01E-30
SLC43A2	2.040802	5.492692	5.13E-28	4.01E-24
C5AR1	1.861943	4.696883	1.88E-27	1.10E-23
SLC16A6	1.719096	5.269314	1.01E-25	4.72E-22
SDSL	1.840979	5.702371	6.36E-25	2.48E-21
MYC	-1.85278	3.579885	2.11E-20	7.05E-17
PNCK	2.368382	2.863811	8.23E-20	2.41E-16
SULF2	1.560743	4.692587	5.17E-19	1.24E-15
DCLK1	-1.48808	7.19861	5.95E-19	1.27E-15

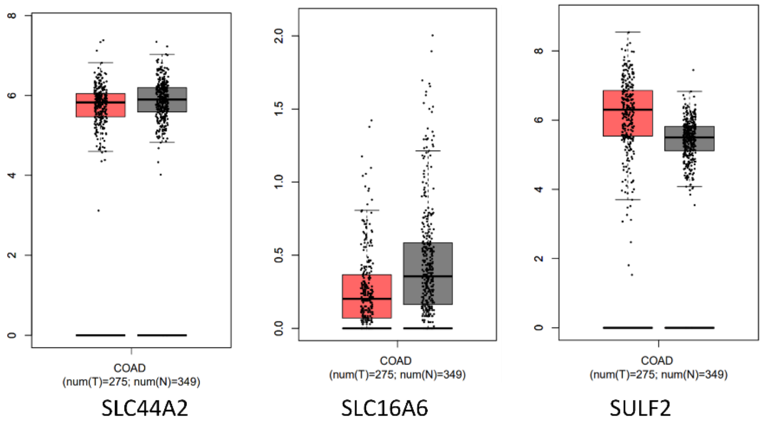

Figure S10 Expression levels of SLC44A2, SLC16A6, and SULF2 in colon cancer and normal tissue.

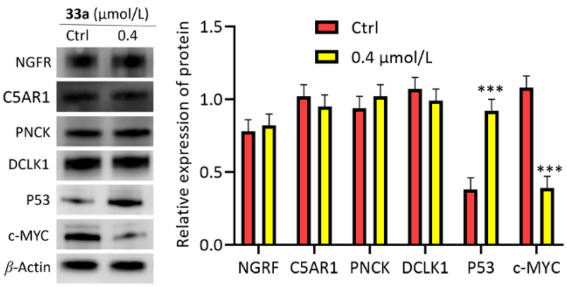

Figure S11 Western blotting analysis of NGFR, C5AR1, PNCK, DCLK1, P53, and c-MYC expression levels. The data were mean ±SD (n = 3), ***P < 0.001 compared with the control cells.

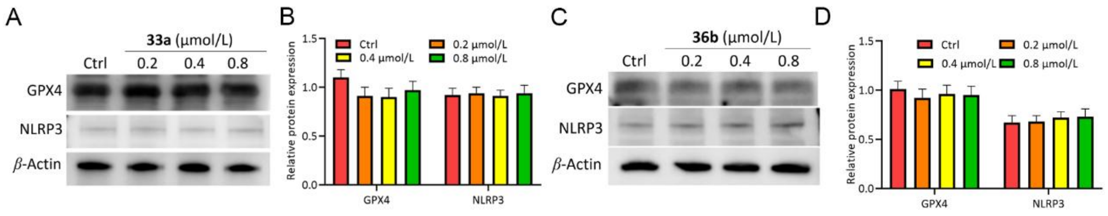

Figure S12 Western blotting analysis of GPX4 and NLRP3 expression levels. The data were mean ±SD (n = 3).

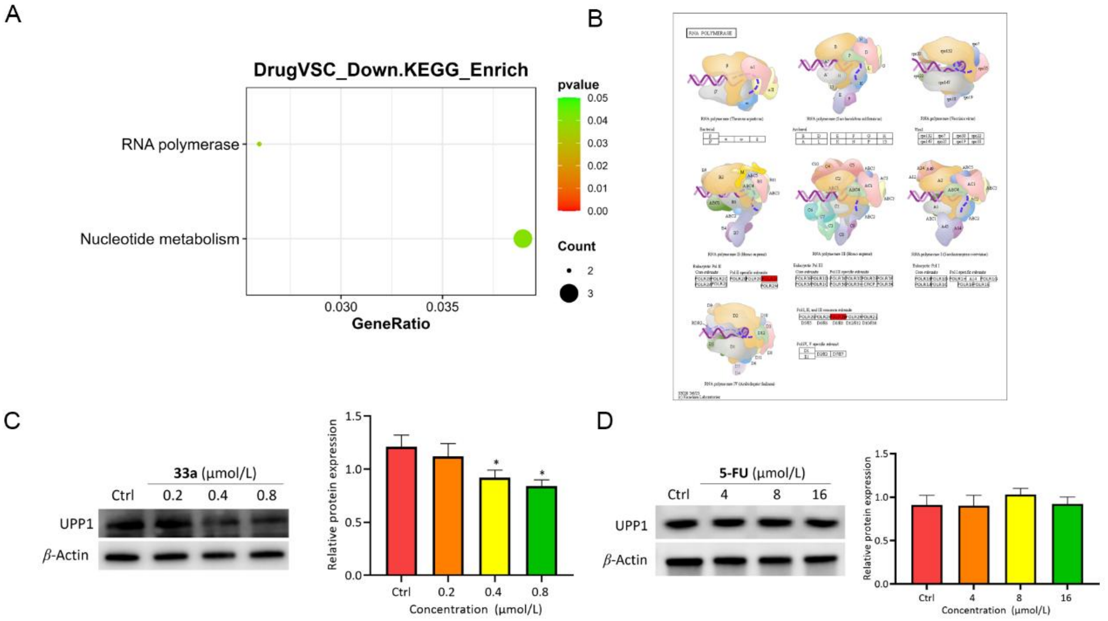

Figure S13 The impact of **33a** on transcription regulation and nucleic acid metabolic regulation**.** (A) KEGG pathway enrichment analysis. (B) Pathwaymaps. (C–D) Western blot analysis of the expression levels of UPP1. The data were mean ±SD (n = 3). *P

<0.05 compared with the control cells.

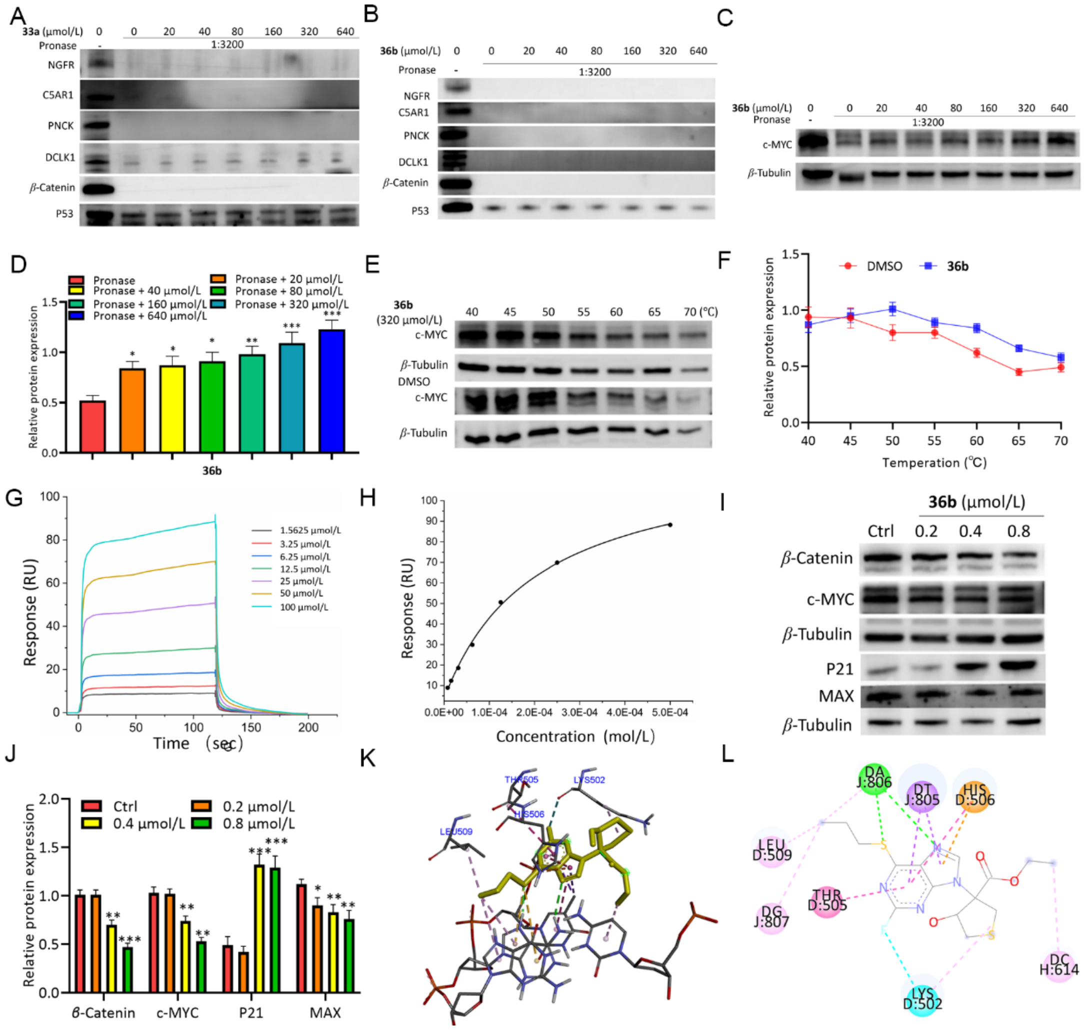

Figure S14 Interaction of compound **36b** and c-MYC (PDB code: 1NKP). (A–B) NGFR, C5AR1, PNCK, DLCK1, β-Catenin, and P53 are protected by **33a** or 36b against proteolysis in DARTS assays. (C–D) c-MYC protected by compound **36b** against proteolysis in DARTS assays. (E-F) Compound **36b** binding with c-MYC in CETSA experiment. (G–H) KD value between compound **36b** and c-MYC determined by SPR analysis. (I–J) The expression levels of β-Catenin, c-MYC, P21, and MAX. (K-L) 3D and 2D (hydrogen bonding (green) and hydrophobic bond (purple)) molecular interactions of compound **36b** with c-MYC. Data are mean ± SD (n = 3), *P < 0.05, **P < 0.01, and ***P < 0.001 compared with the control group.

Table S5 Analysis of Main Pharmacokinetic Parameters

Subject	$T_{1/2}$ (h)	T_{max} (h)	C_{max}	$AUC_{0-\infty}$	CL	Vss

			(ng/mL)	(ng·h/L)	(mL/min/ kg)	(mL/kg)
**36b** (IV)[a]	4.9	–	–	8801.4	1.89	3541.5
**36b** (Po)[b]	4.1	4	276.3	1804.8	–	–

–, not applicable.

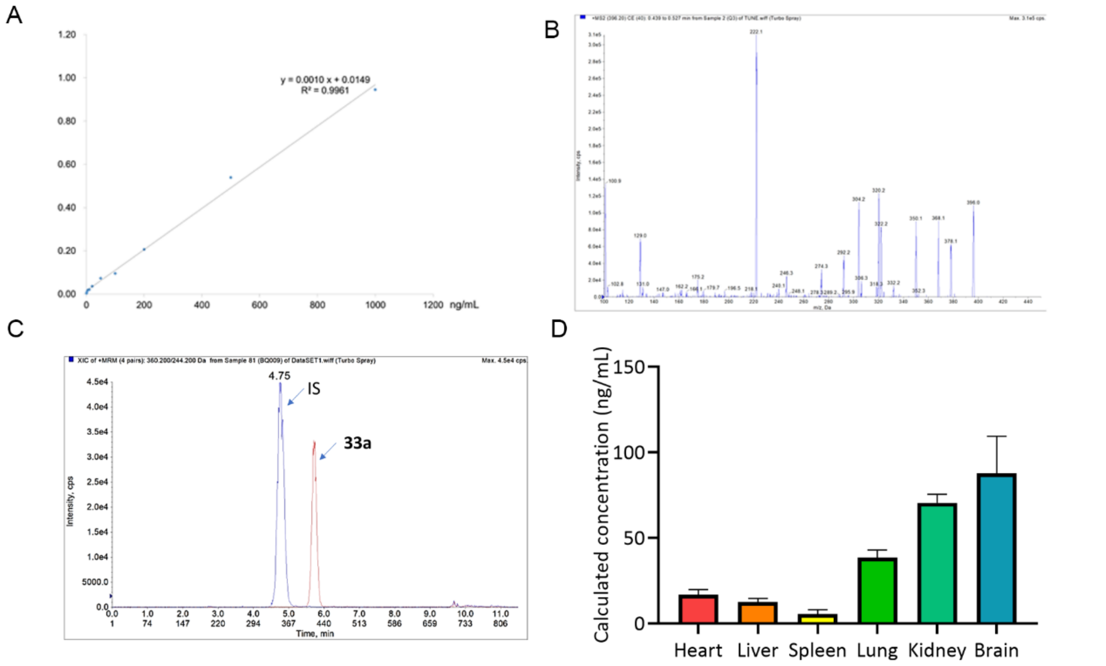

Figure S15 Tissue distribution of **33a** in BALB/c mice. (A) Standard curve. (B) Typical mass spectrometry diagram of **33a**. (C) Typical chromatogram of **33a**. (D) Calculated concentrations of **33a** in different tissues of mice. The data were mean ±SD (n = 3).

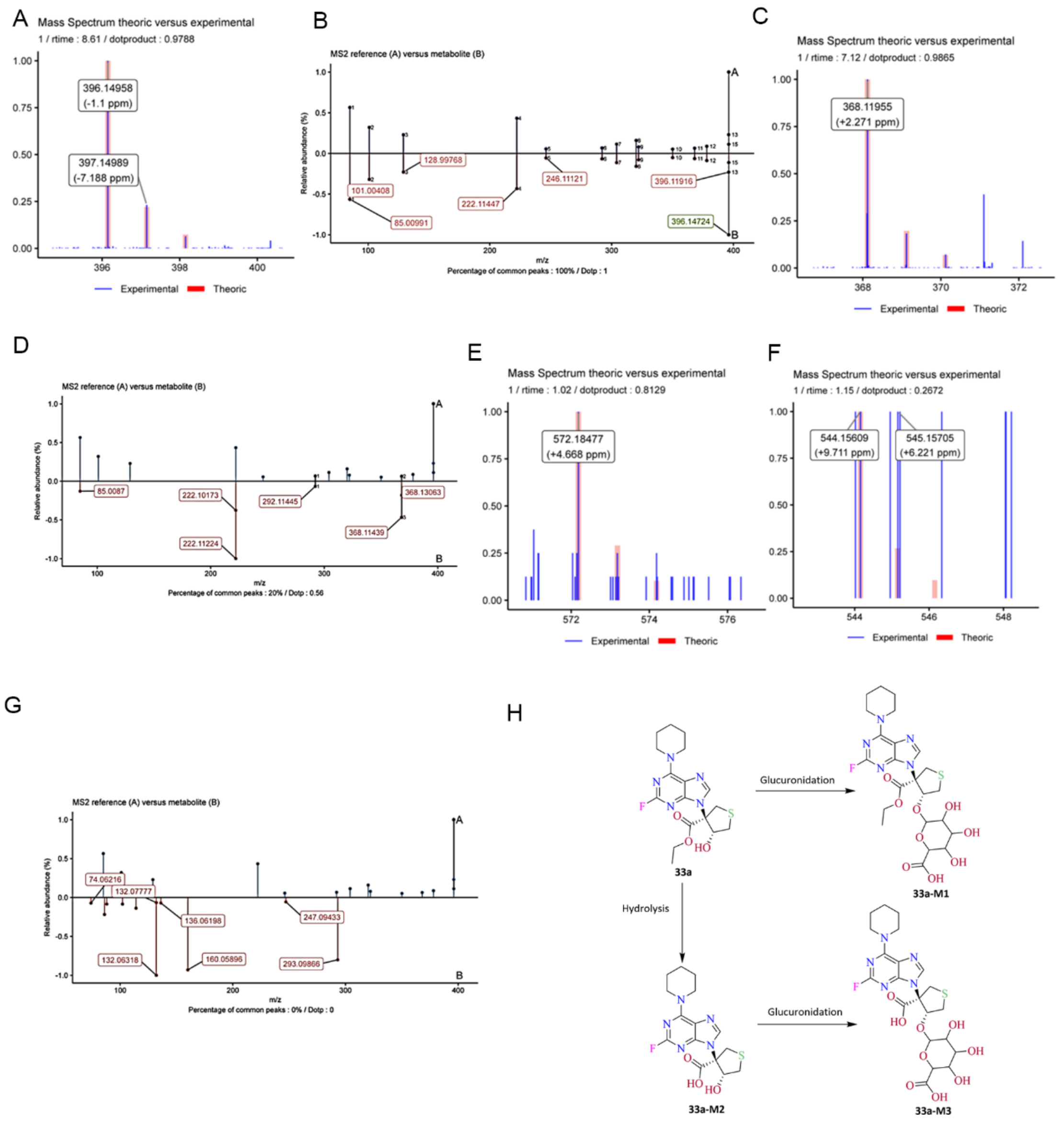

Figure S16 The metabolites of **33a** in mice. (A–G) The chromatography and mass spectrography of the metabolites of **33a**. (H) The main metabolic pathway of **33a**.

References

1. Huang KX, Xie MS, Zhang QY, Qu GR, Guo HM. Enantioselective synthesis of carbocyclic nucleosides *via* asymmetric [3 + 2] annulation of α-purine-substituted acrylates with MBH carbonates. *Org Lett* 2018;**20**:389–92.
2. Huang KX, Xie MS, Sang JW, Qu GR, Guo HM. Asymmetric synthesis of 3-amine-tetrahydrothiophenes with a quaternary stereocenter *via* Nickel(II)/Trisoxazoline-catalyzed sulfa-Michael/Aldol cascade reaction: Divergent access to chiral thionucleosides. *Org Lett* 2021;**23**:81–6.
